# Supplementary figures and images for: Decitabine cytotoxicity is promoted by dCMP deaminase DCTD and mitigated by SUMO-dependent E3 ligase TOPORS (part 1 of 3)
Source: EMBO J. 2024 May 17;43(12):6. doi: 10.1038/s44318-024-00108-2 (PMC11183266; doi:10.1038/s44318-024-00108-2)

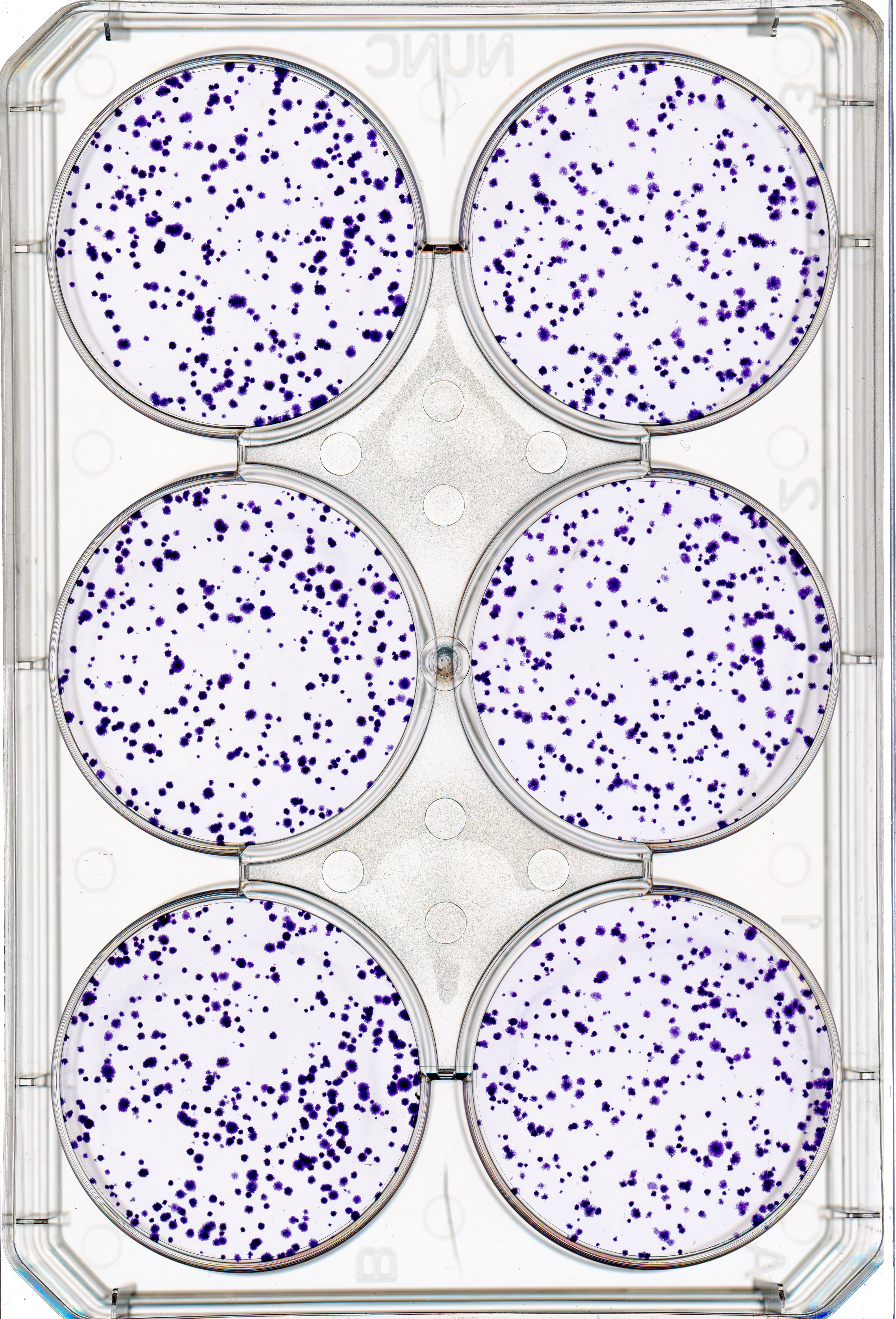

Supplement: Supplementary file 4 — Source data Fig. 1 [file 44318_2024_108_MOESM4_ESM.zip › EMBOJ-2023-115654_Fig1_sourcedata/Figure1E/E231005 DCTD 5dC0-100.jpg]

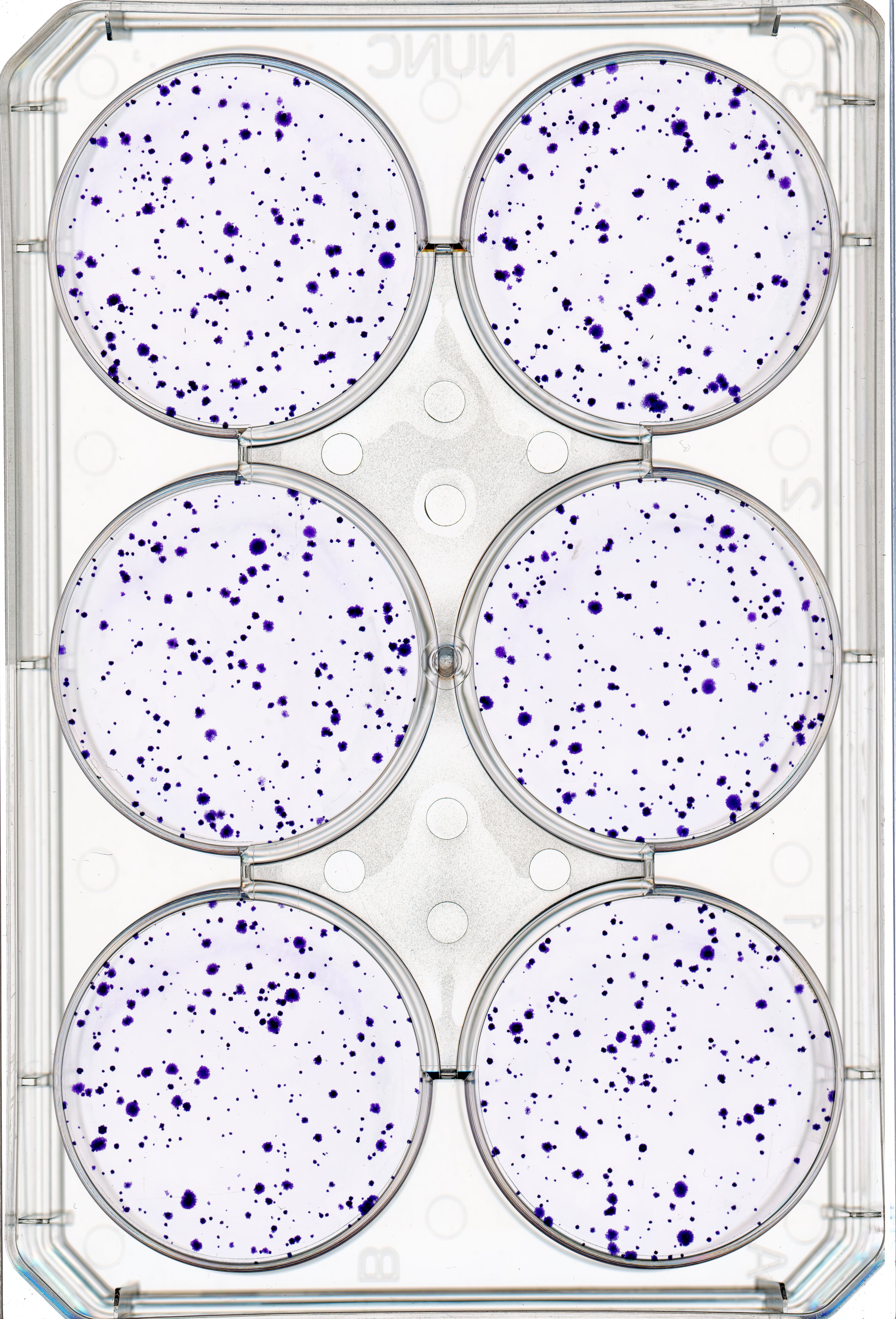

Supplement: Supplementary file 4 — Source data Fig. 1 [file 44318_2024_108_MOESM4_ESM.zip › EMBOJ-2023-115654_Fig1_sourcedata/Figure1E/E231005 DCK 5dC200-400.jpg]

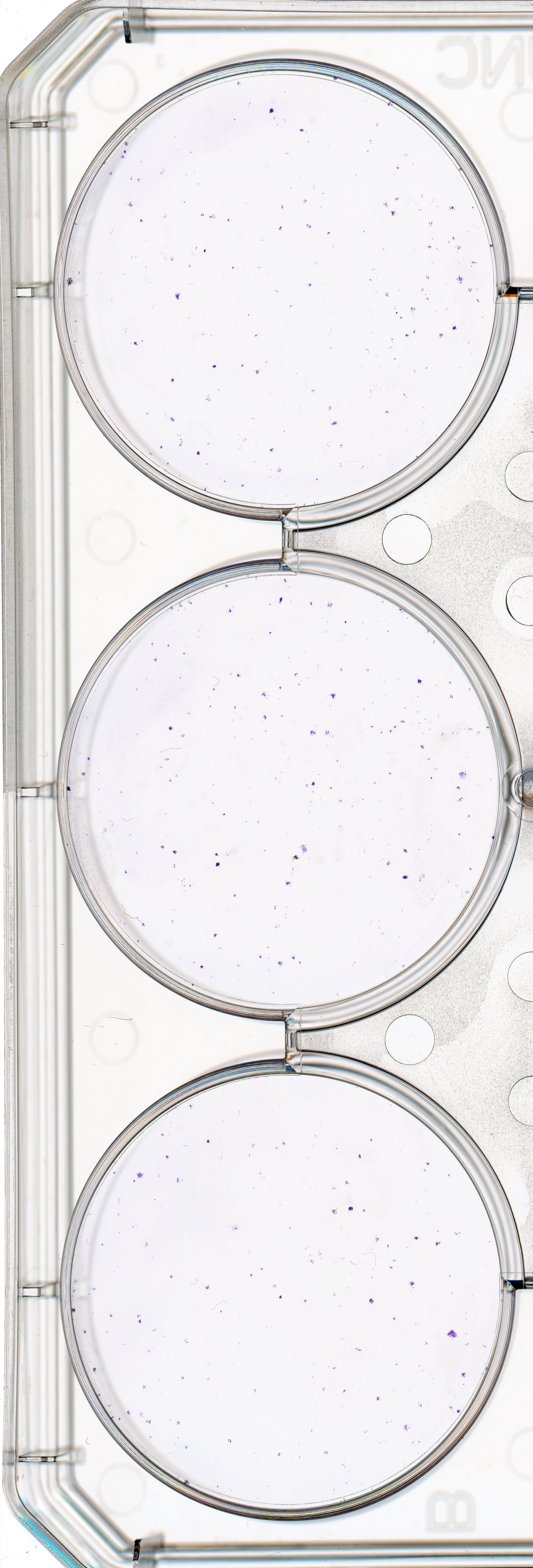

Supplement: Supplementary file 4 — Source data Fig. 1 [file 44318_2024_108_MOESM4_ESM.zip › EMBOJ-2023-115654_Fig1_sourcedata/Figure1E/E231005 DCTD 5dC1000.jpg]

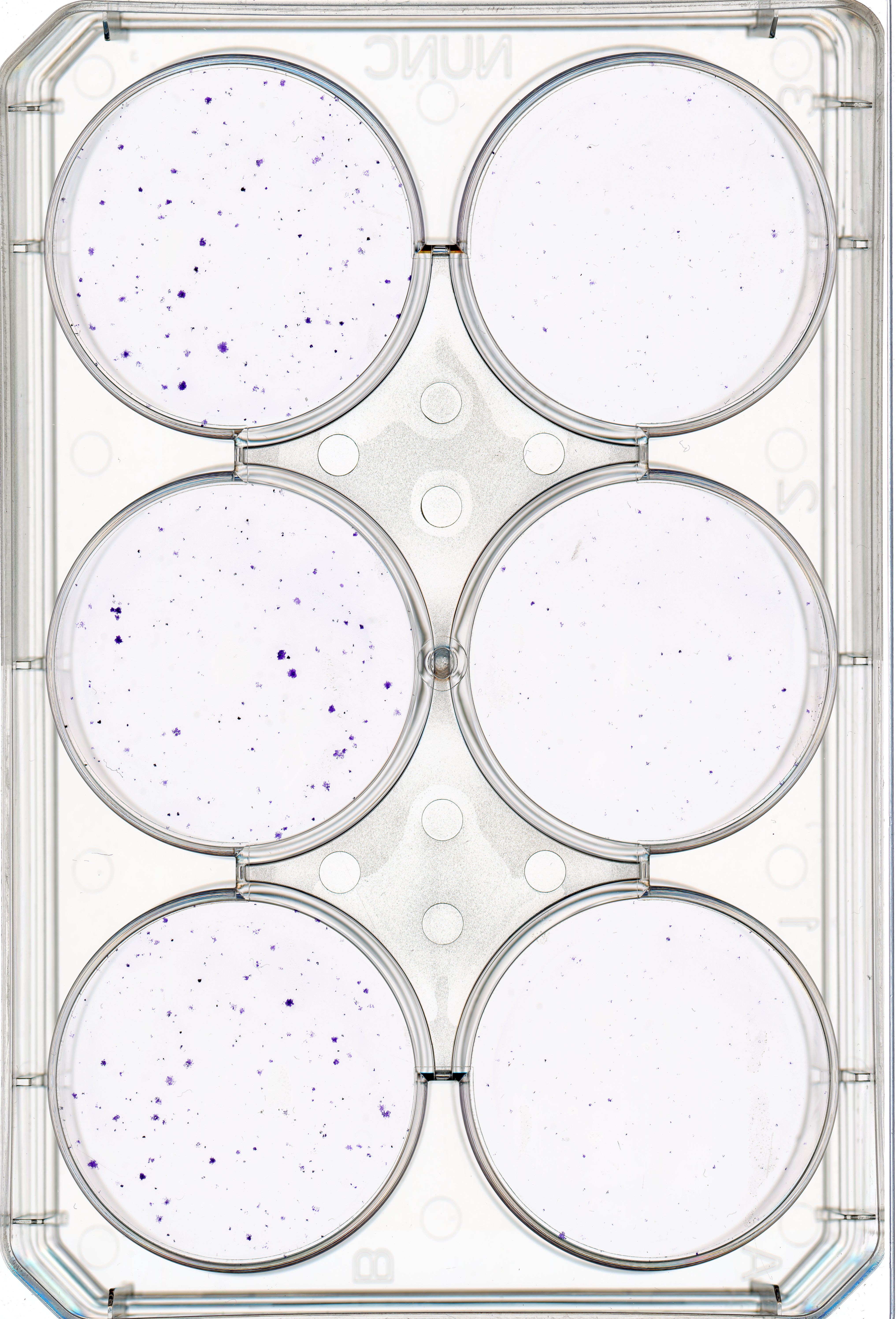

Supplement: Supplementary file 4 — Source data Fig. 1 [file 44318_2024_108_MOESM4_ESM.zip › EMBOJ-2023-115654_Fig1_sourcedata/Figure1E/E231005 WT 5dC200-400.jpg]

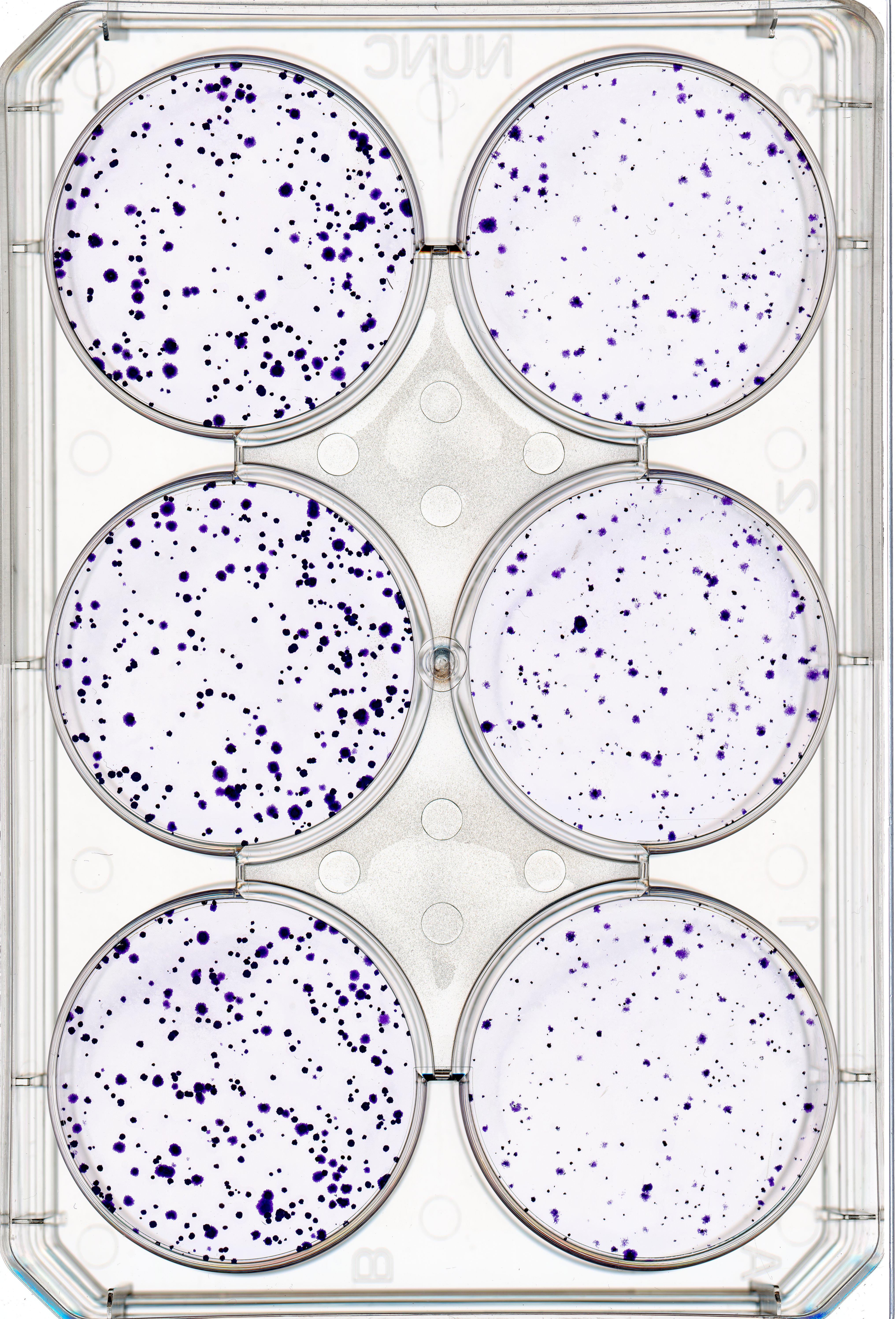

Supplement: Supplementary file 4 — Source data Fig. 1 [file 44318_2024_108_MOESM4_ESM.zip › EMBOJ-2023-115654_Fig1_sourcedata/Figure1E/E231005 WT 5dC0-100.jpg]

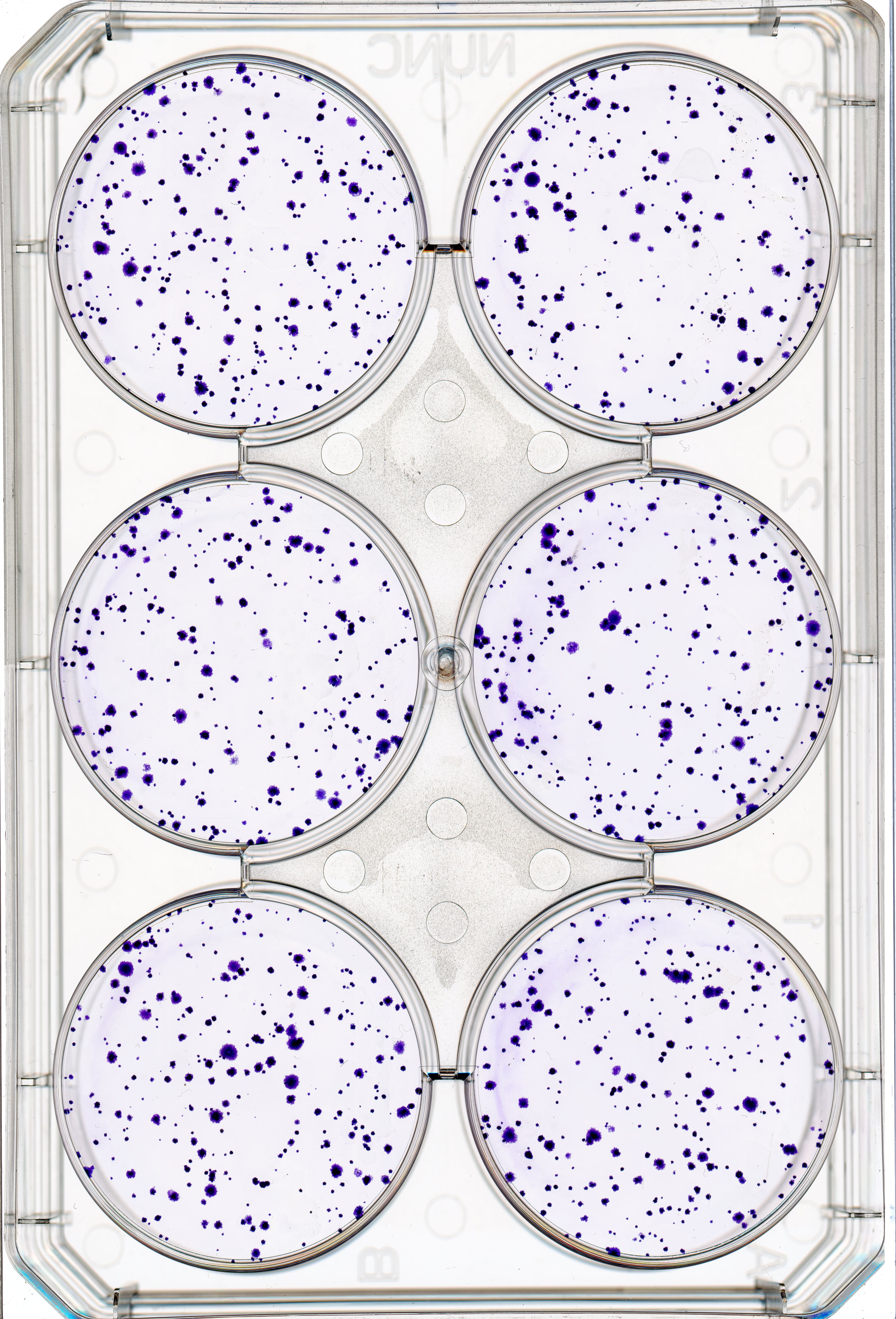

Supplement: Supplementary file 4 — Source data Fig. 1 [file 44318_2024_108_MOESM4_ESM.zip › EMBOJ-2023-115654_Fig1_sourcedata/Figure1E/E231005 DCK 5dC600-800.jpg]

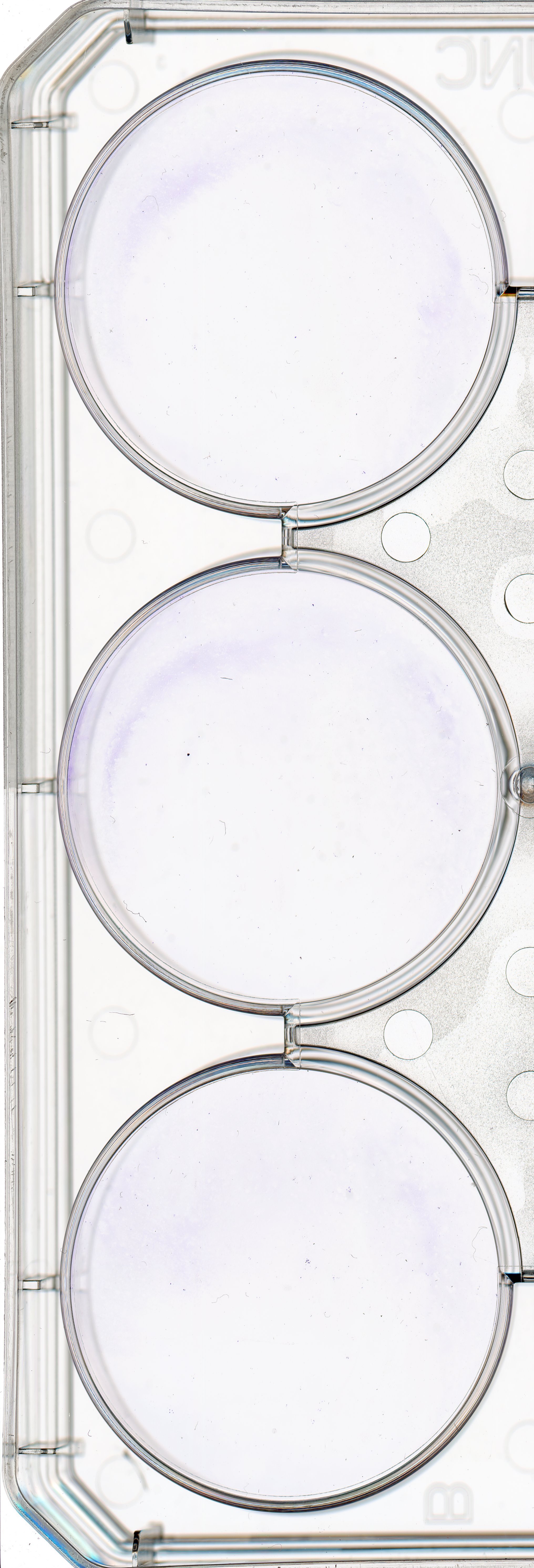

Supplement: Supplementary file 4 — Source data Fig. 1 [file 44318_2024_108_MOESM4_ESM.zip › EMBOJ-2023-115654_Fig1_sourcedata/Figure1E/E231005 WT 5dC1000.jpg]

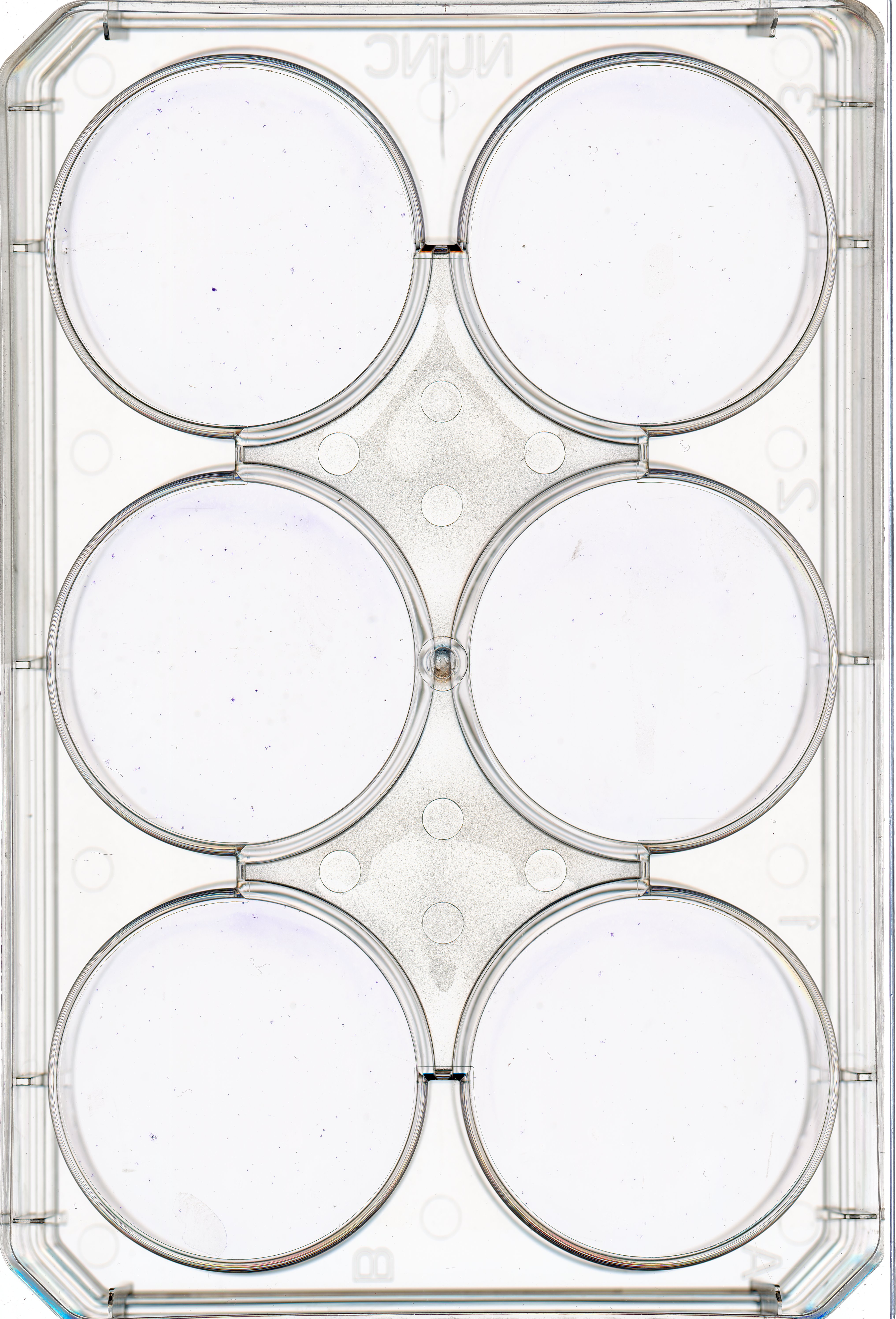

Supplement: Supplementary file 4 — Source data Fig. 1 [file 44318_2024_108_MOESM4_ESM.zip › EMBOJ-2023-115654_Fig1_sourcedata/Figure1E/E231005 WT 5dC600-800.jpg]

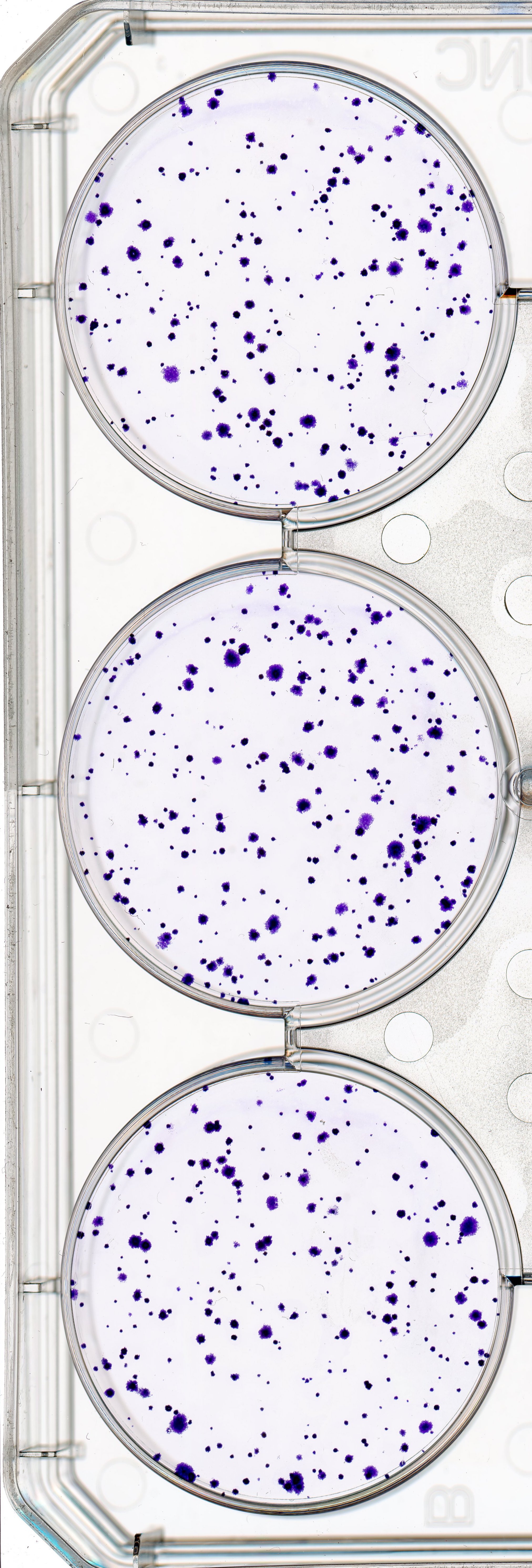

Supplement: Supplementary file 4 — Source data Fig. 1 [file 44318_2024_108_MOESM4_ESM.zip › EMBOJ-2023-115654_Fig1_sourcedata/Figure1E/E231005 DCK 5dC1000.jpg]

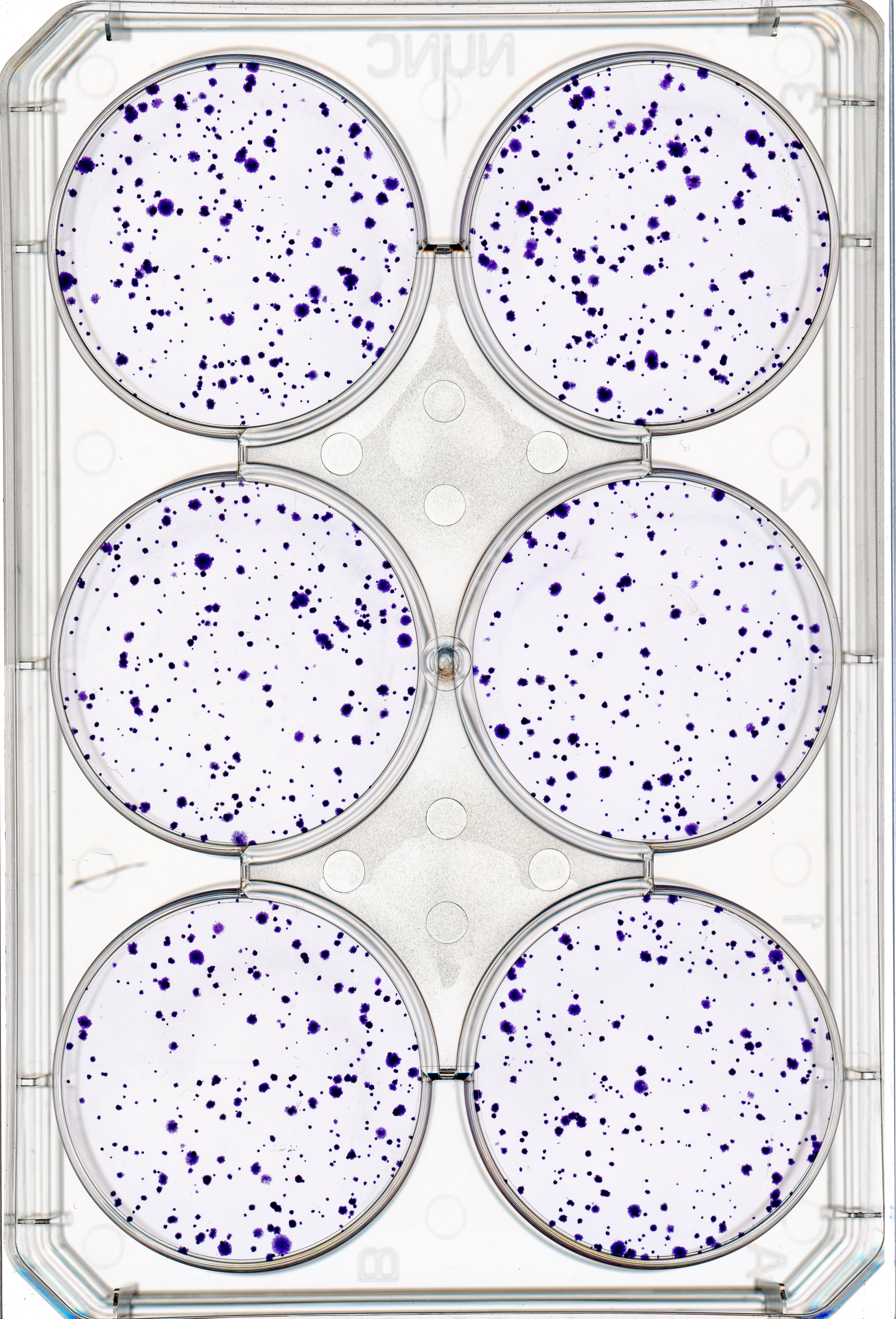

Supplement: Supplementary file 4 — Source data Fig. 1 [file 44318_2024_108_MOESM4_ESM.zip › EMBOJ-2023-115654_Fig1_sourcedata/Figure1E/E231005 DCK 5dC0-100.jpg]

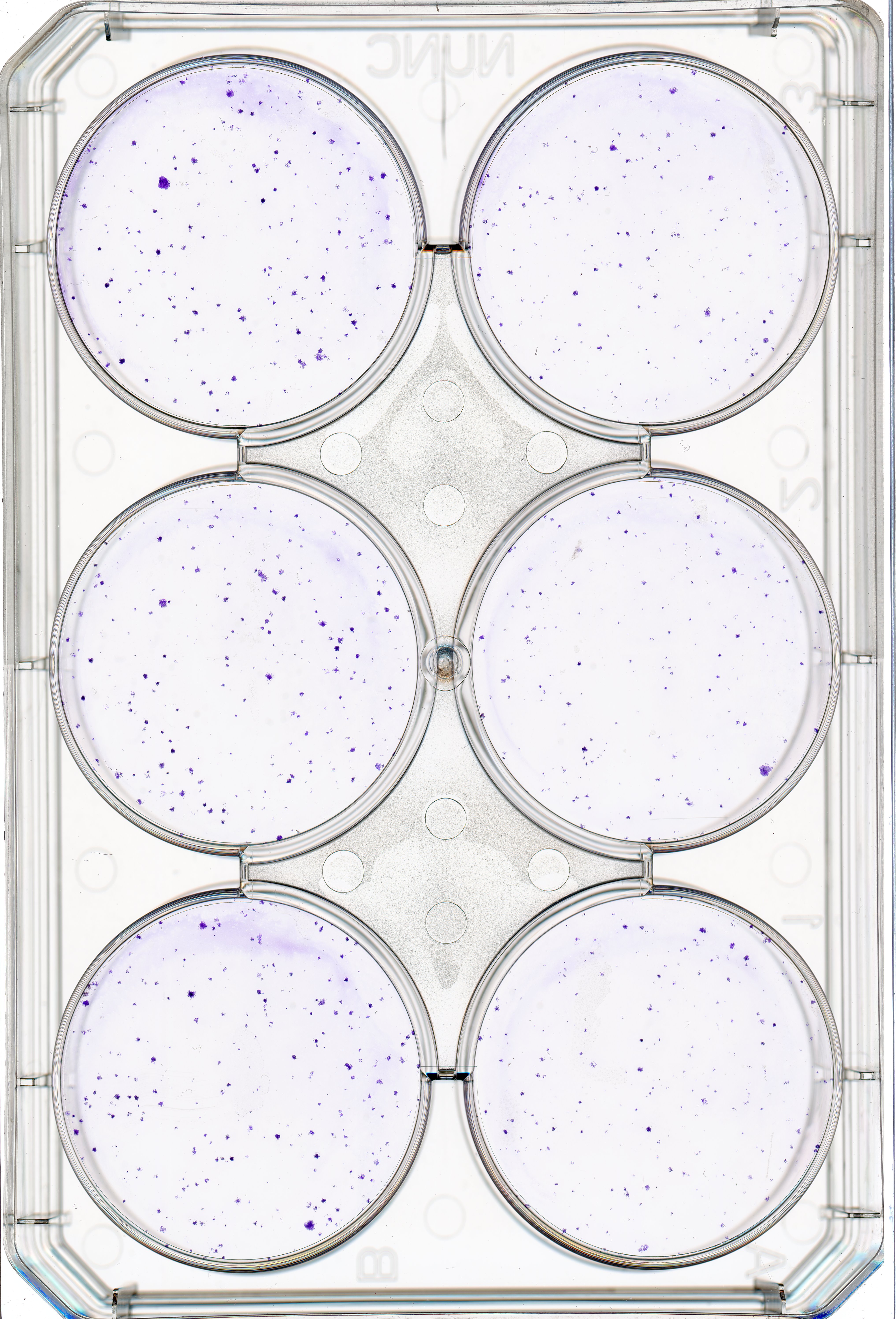

Supplement: Supplementary file 4 — Source data Fig. 1 [file 44318_2024_108_MOESM4_ESM.zip › EMBOJ-2023-115654_Fig1_sourcedata/Figure1E/E231005 DCTD 5dC600-800.jpg]

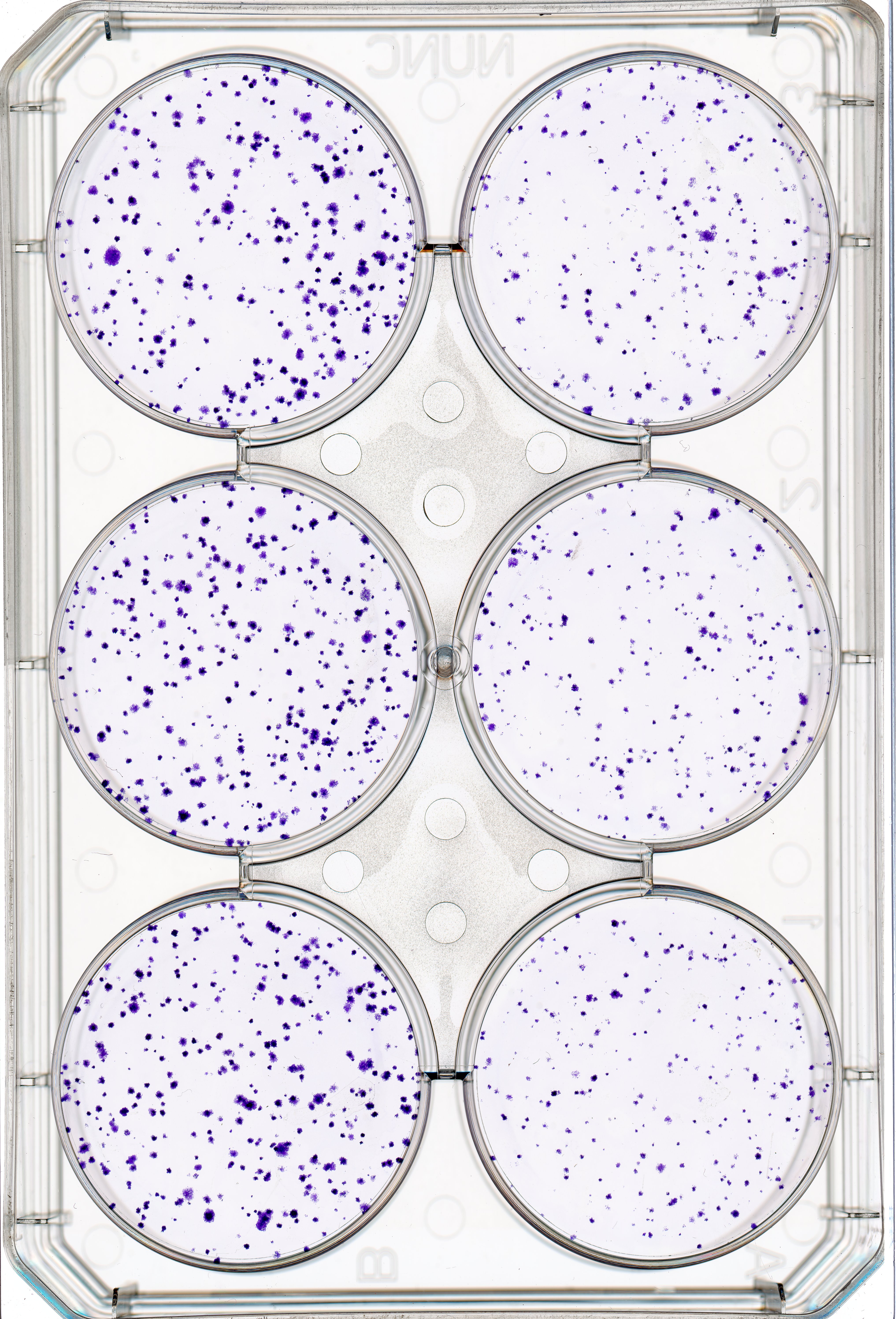

Supplement: Supplementary file 4 — Source data Fig. 1 [file 44318_2024_108_MOESM4_ESM.zip › EMBOJ-2023-115654_Fig1_sourcedata/Figure1E/E231005 DCTD 5dC200-400.jpg]

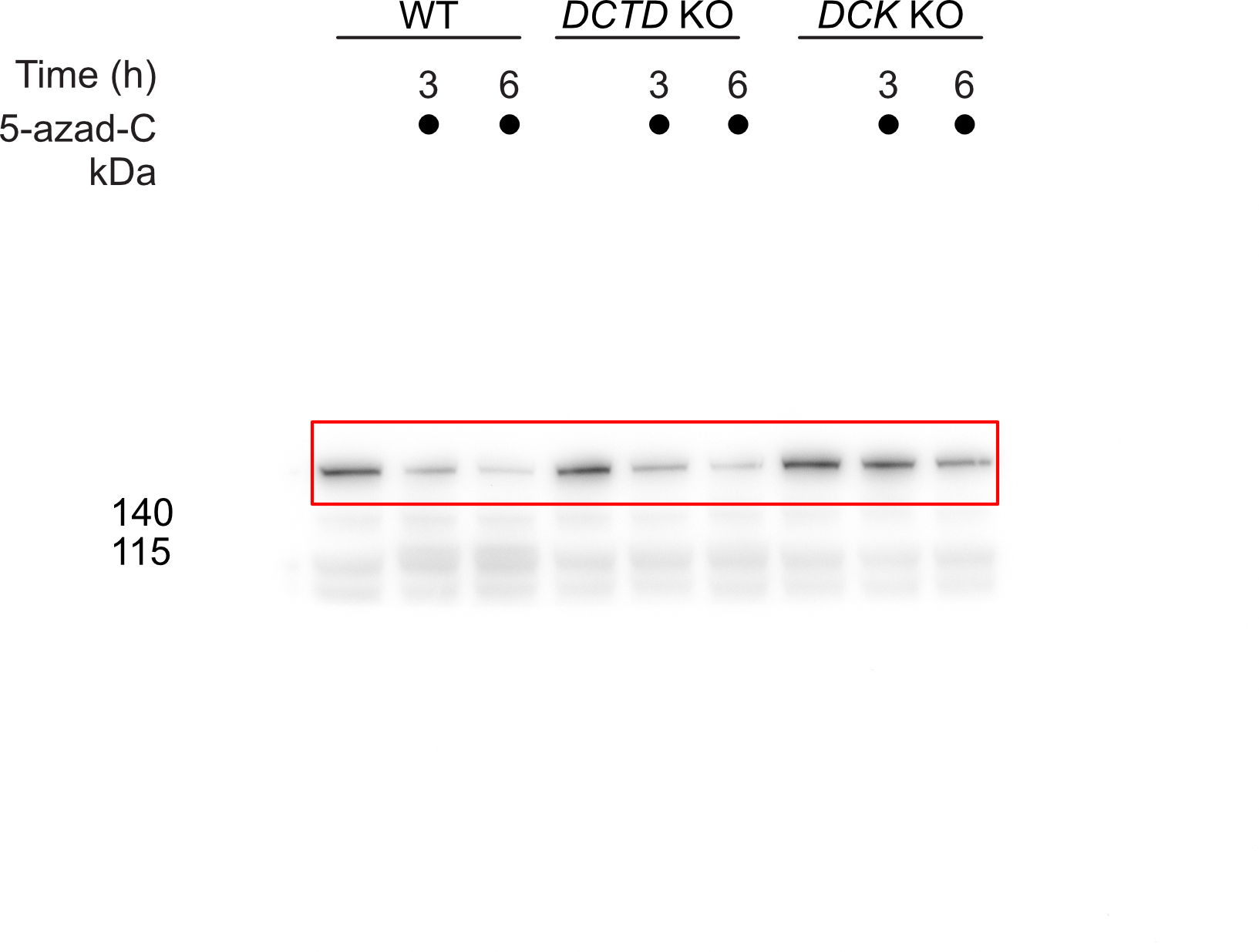

Supplement: Supplementary file 5 — Source data Fig. 2 [file 44318_2024_108_MOESM5_ESM.zip › EMBOJ-2023-115654_Fig2_sourcedata/Fig2B/western blot DNMT1 input.tiff]

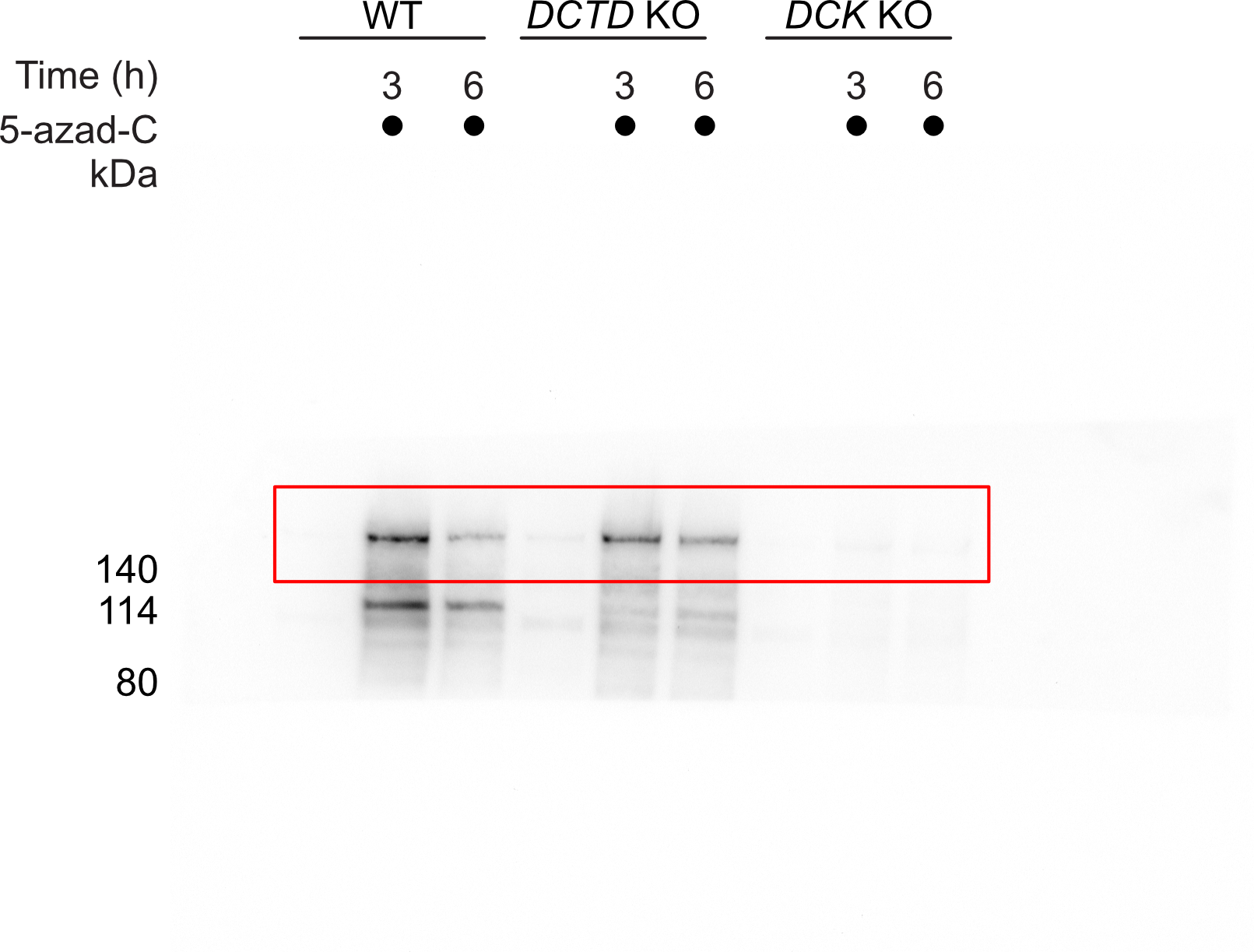

Supplement: Supplementary file 5 — Source data Fig. 2 [file 44318_2024_108_MOESM5_ESM.zip › EMBOJ-2023-115654_Fig2_sourcedata/Fig2B/western blot DNMT1 PxP.tiff]

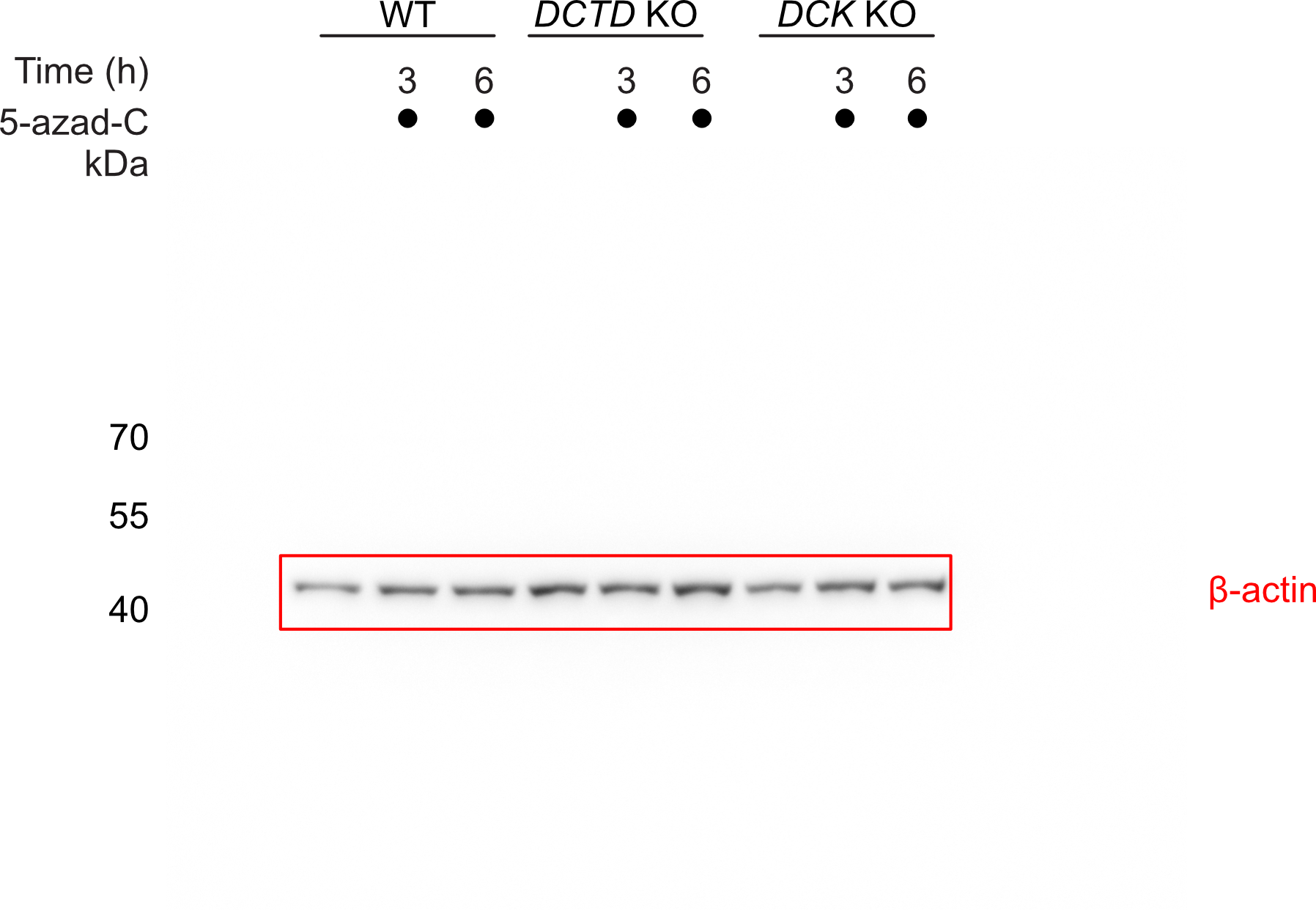

Supplement: Supplementary file 5 — Source data Fig. 2 [file 44318_2024_108_MOESM5_ESM.zip › EMBOJ-2023-115654_Fig2_sourcedata/Fig2B/western blot actin.tiff]

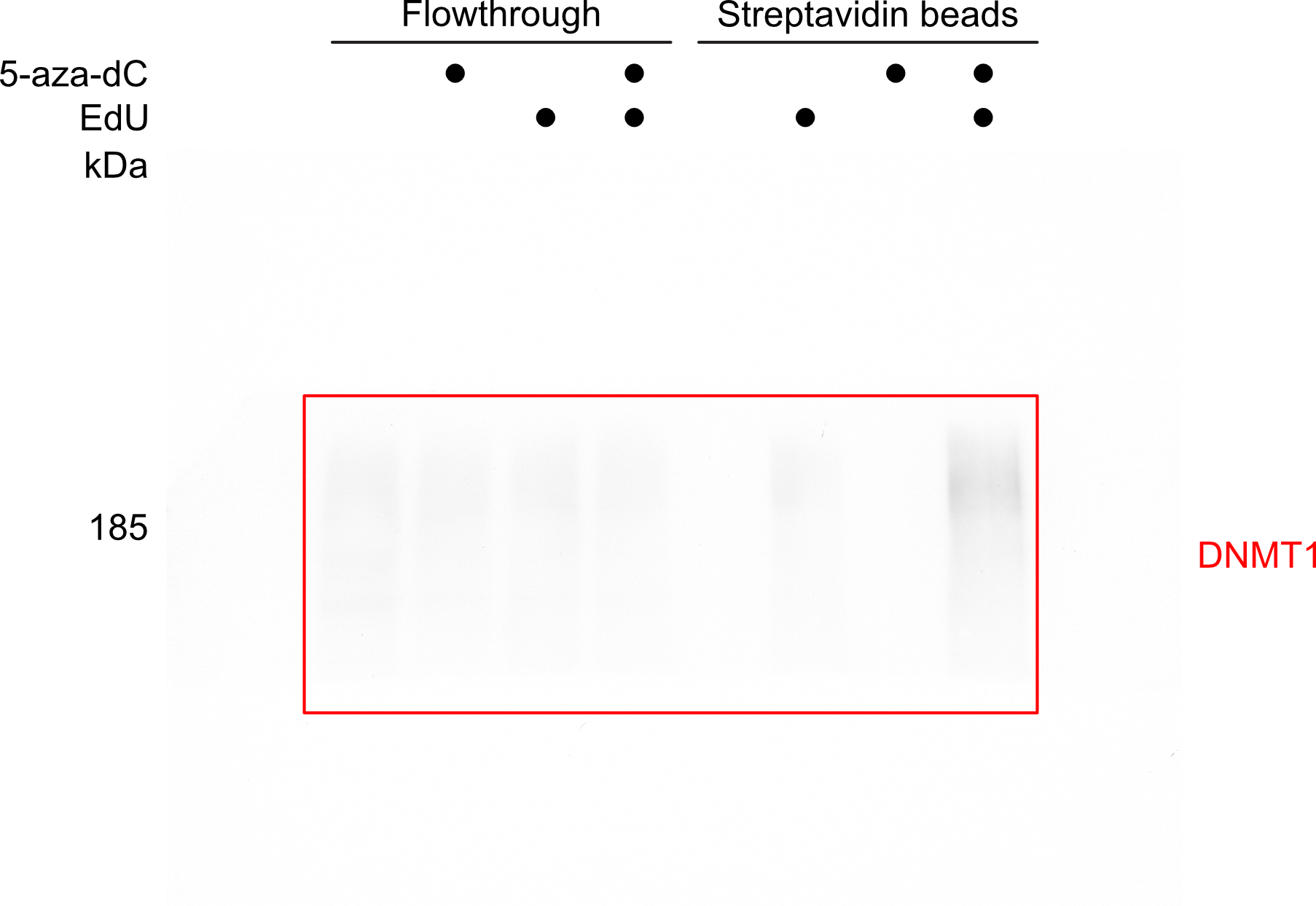

Supplement: Supplementary file 6 — Source data Fig. 3 [file 44318_2024_108_MOESM6_ESM.zip › EMBOJ-2023-115654_Fig3_sourcedata/3B/western blot DNMT1.tiff]

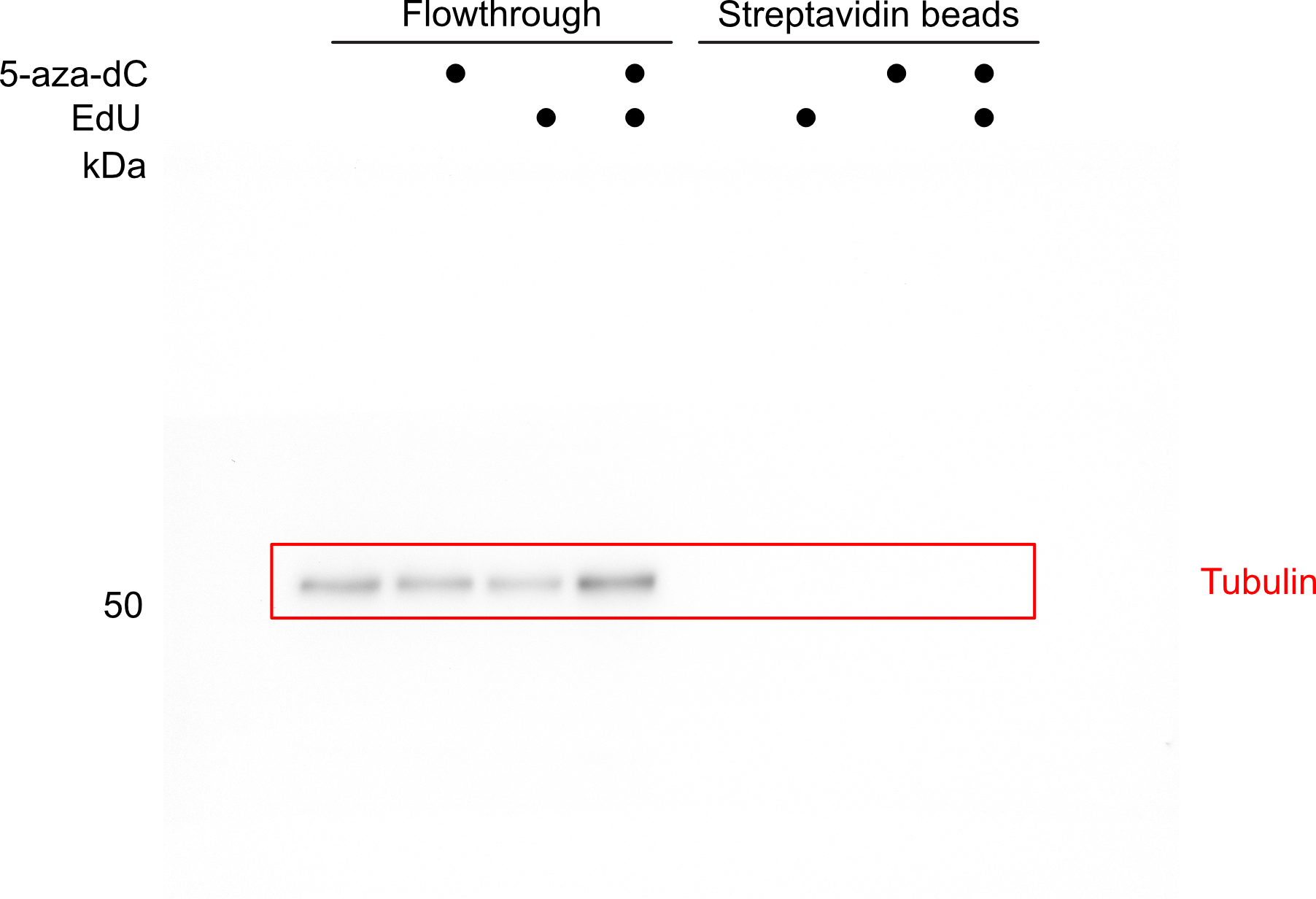

Supplement: Supplementary file 6 — Source data Fig. 3 [file 44318_2024_108_MOESM6_ESM.zip › EMBOJ-2023-115654_Fig3_sourcedata/3B/western blot tubulin.tiff]

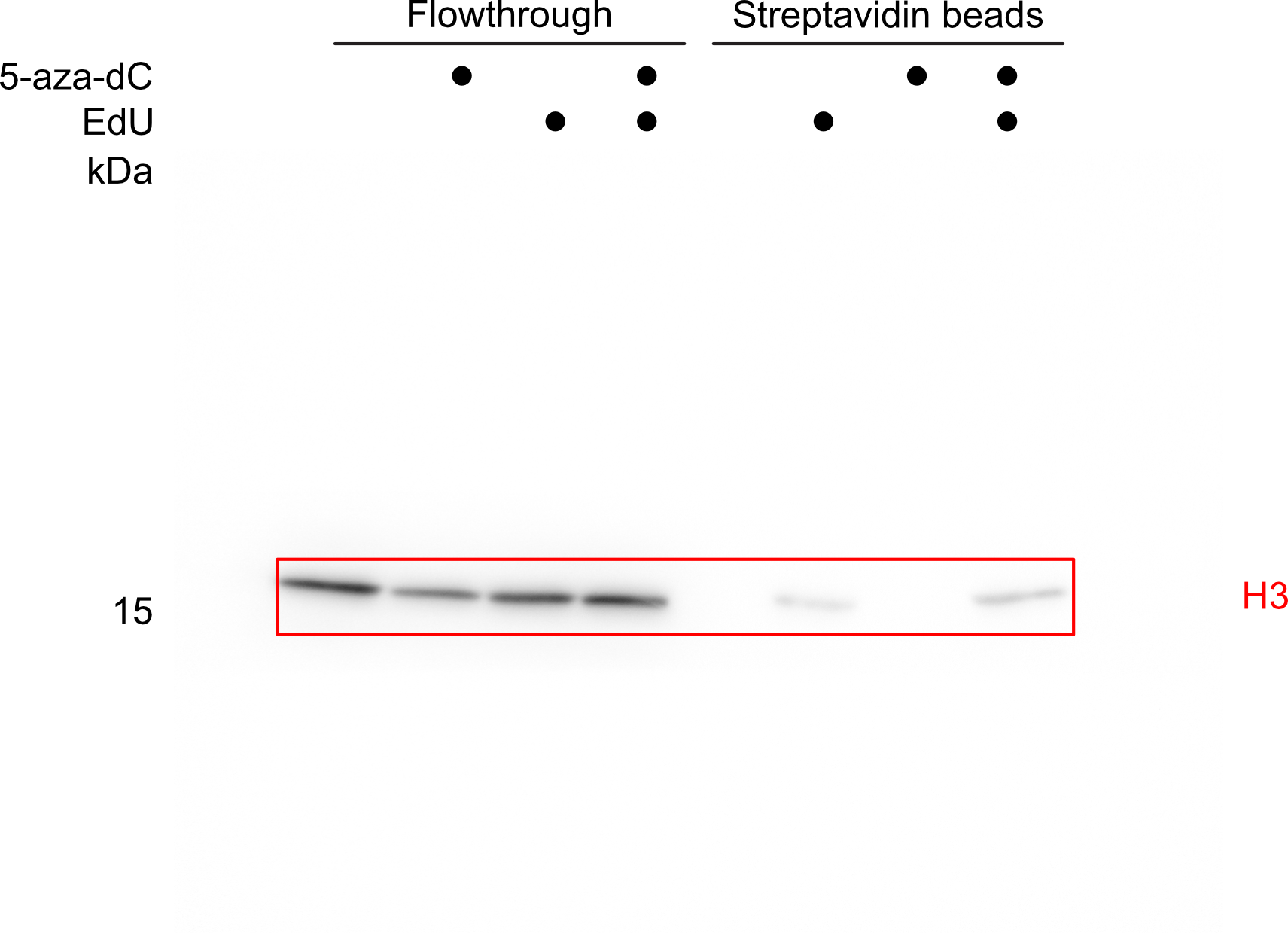

Supplement: Supplementary file 6 — Source data Fig. 3 [file 44318_2024_108_MOESM6_ESM.zip › EMBOJ-2023-115654_Fig3_sourcedata/3B/western blot H3.tif]

Source data: Figure 4F.

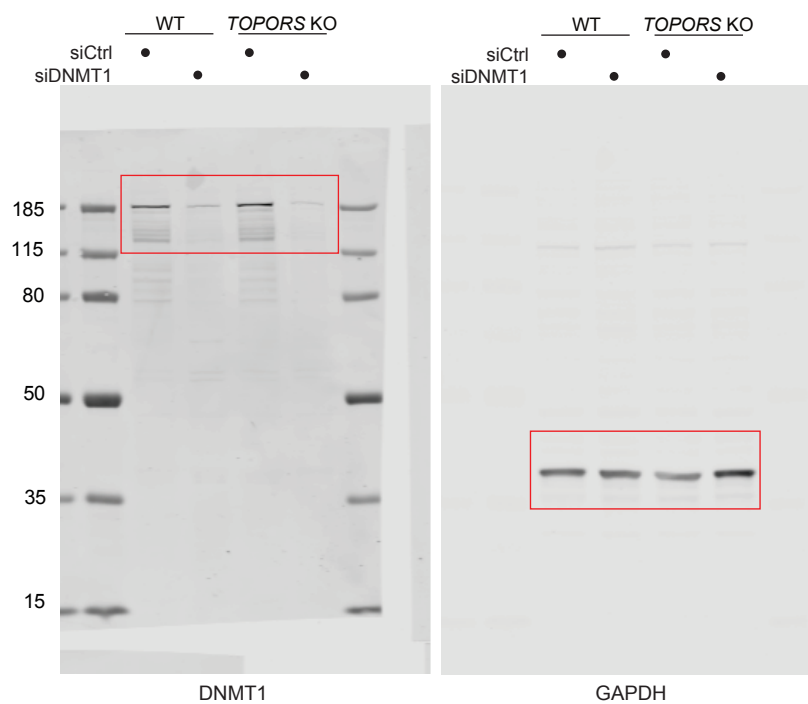

Supplement: Supplementary file 7 — Source data Fig. 4 [file 44318_2024_108_MOESM7_ESM.zip › EMBOJ-2023-115654_Fig4_sourcedata/Figure4F.pdf]

Source data: Figure 4I.

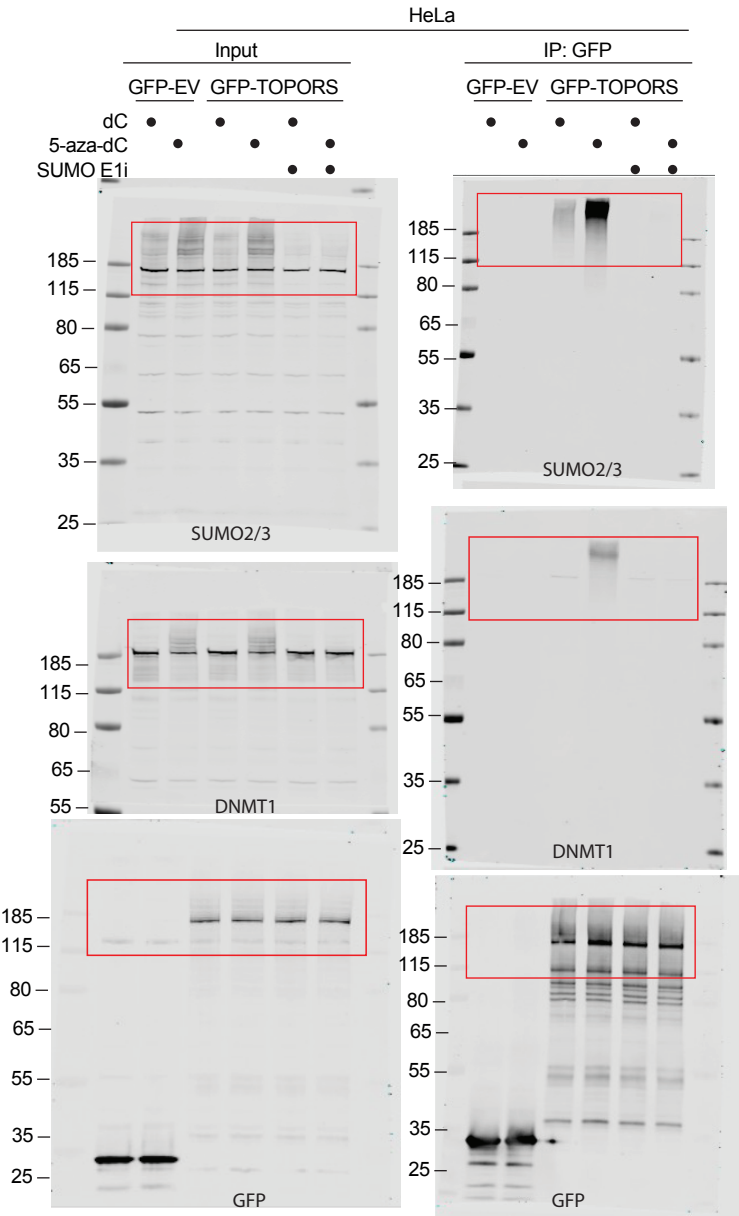

Supplement: Supplementary file 7 — Source data Fig. 4 [file 44318_2024_108_MOESM7_ESM.zip › EMBOJ-2023-115654_Fig4_sourcedata/Figure4I.pdf]

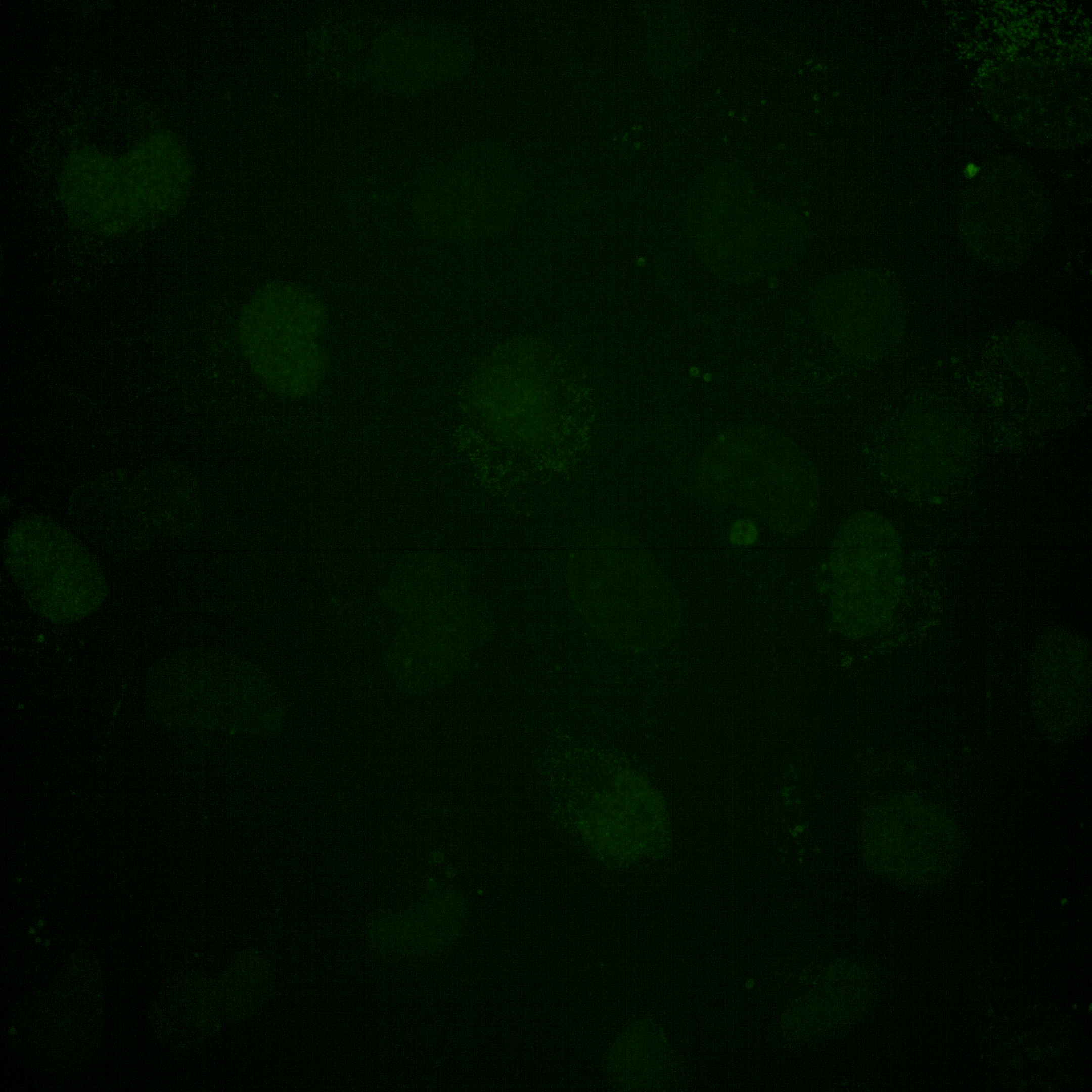

Supplement: Supplementary file 7 — Source data Fig. 4 [file 44318_2024_108_MOESM7_ESM.zip › EMBOJ-2023-115654_Fig4_sourcedata/Figure4G/E231109 HA-TRS PLA dC - GFP.png]

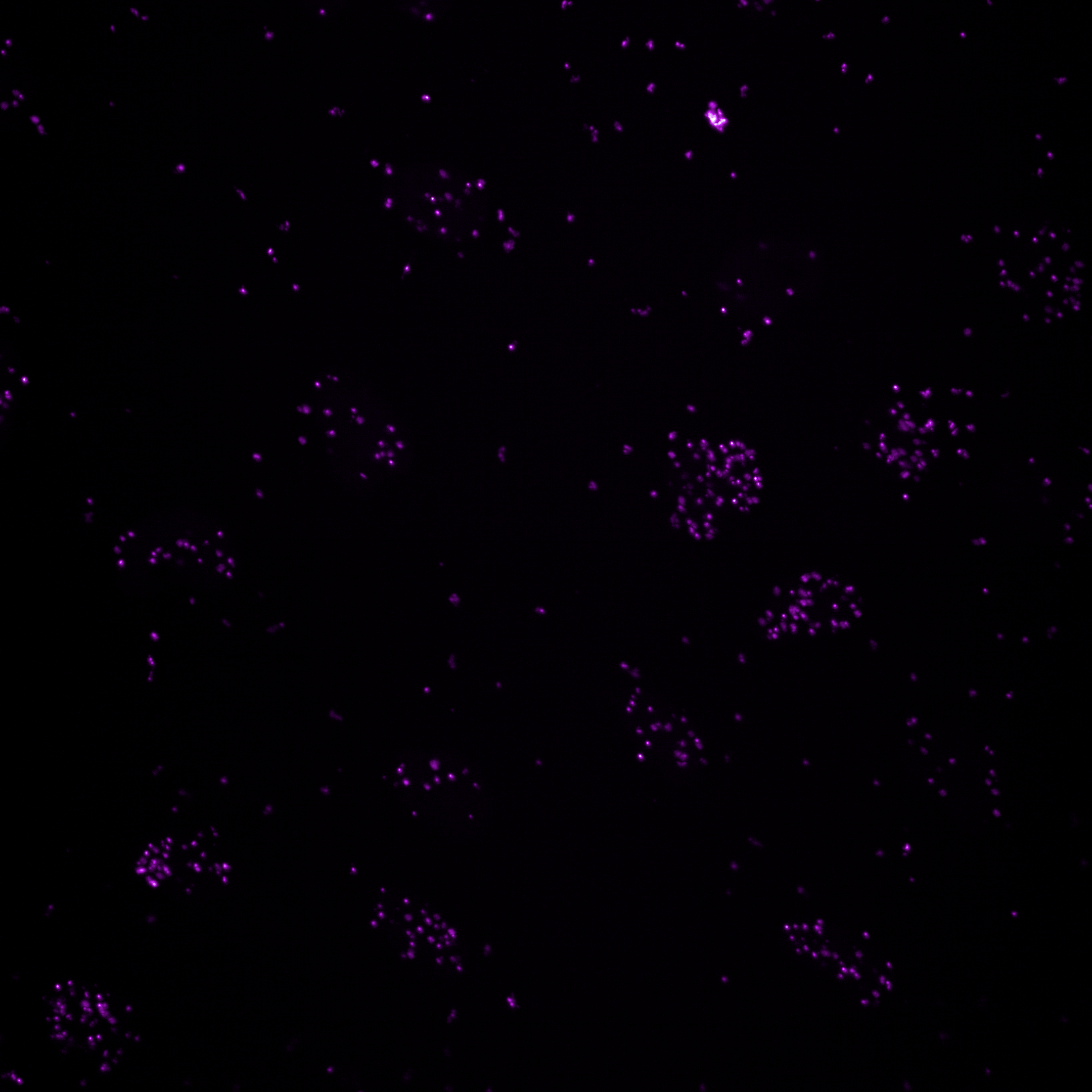

Supplement: Supplementary file 7 — Source data Fig. 4 [file 44318_2024_108_MOESM7_ESM.zip › EMBOJ-2023-115654_Fig4_sourcedata/Figure4G/E231109 HA-TRS PLA dC-Ubi - PLA.png]

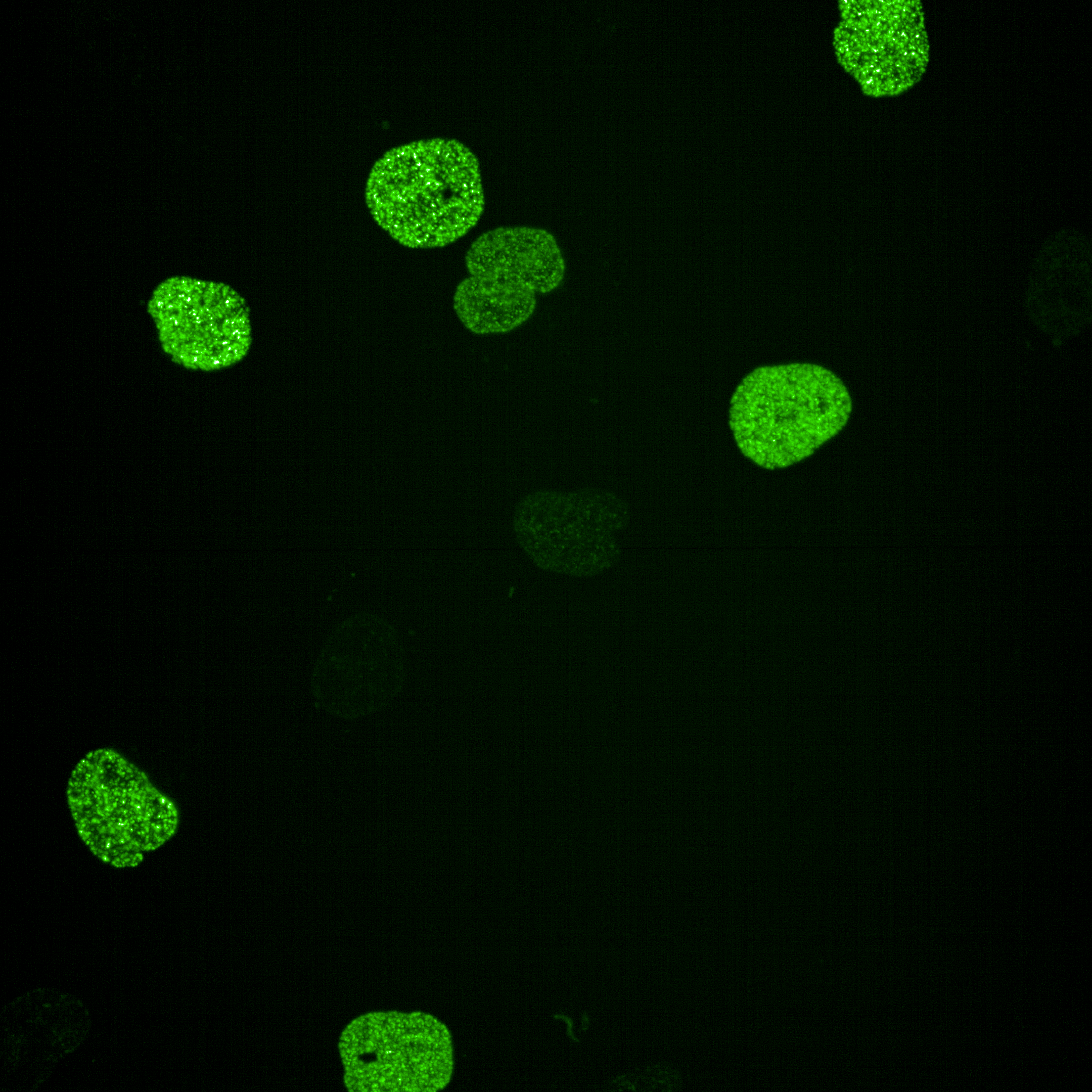

Supplement: Supplementary file 7 — Source data Fig. 4 [file 44318_2024_108_MOESM7_ESM.zip › EMBOJ-2023-115654_Fig4_sourcedata/Figure4G/E231109 HA-TRS HAonly 5dC - GFP.png]

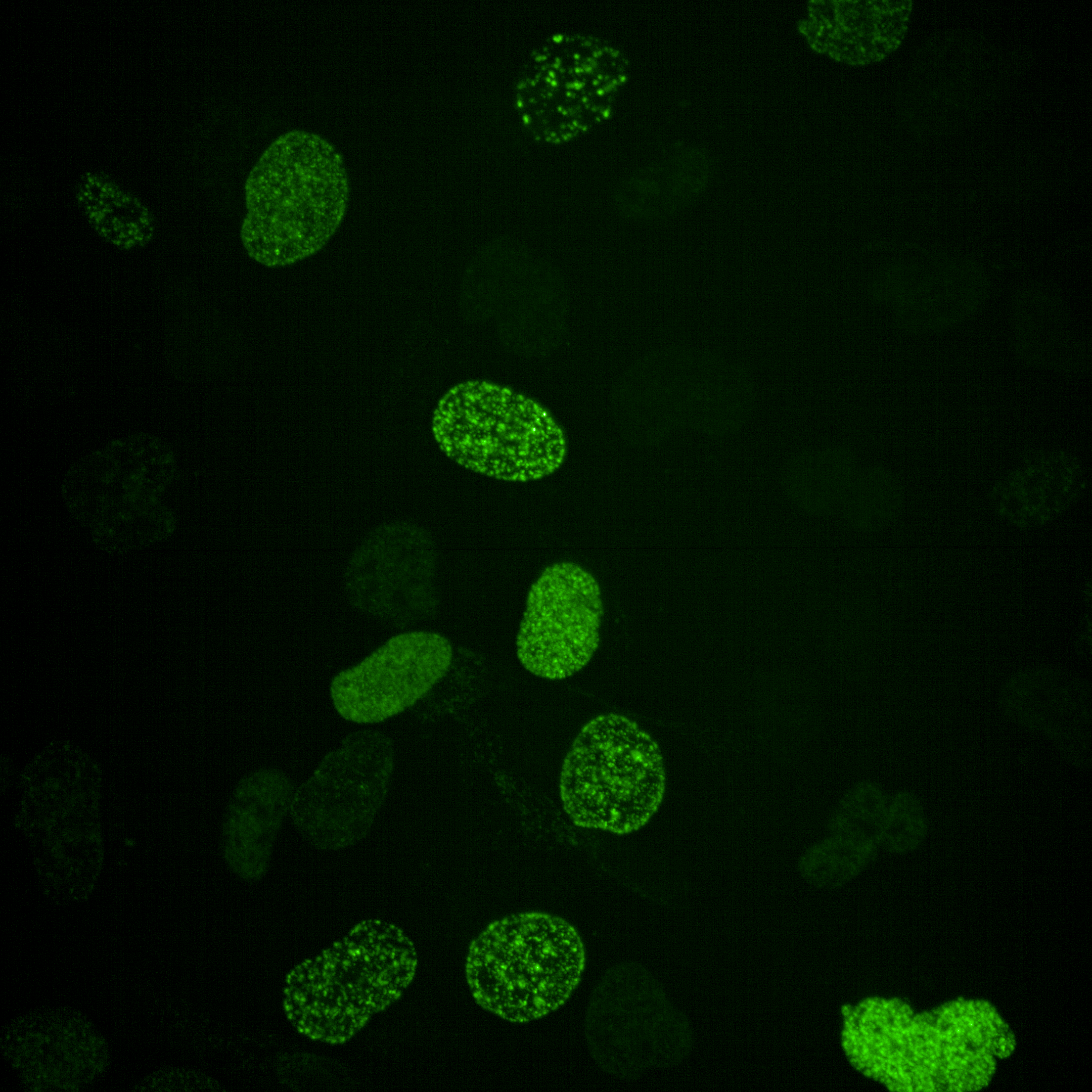

Supplement: Supplementary file 7 — Source data Fig. 4 [file 44318_2024_108_MOESM7_ESM.zip › EMBOJ-2023-115654_Fig4_sourcedata/Figure4G/E231109 HA-TRS PLA 5dC-Ubi - GFP.png]

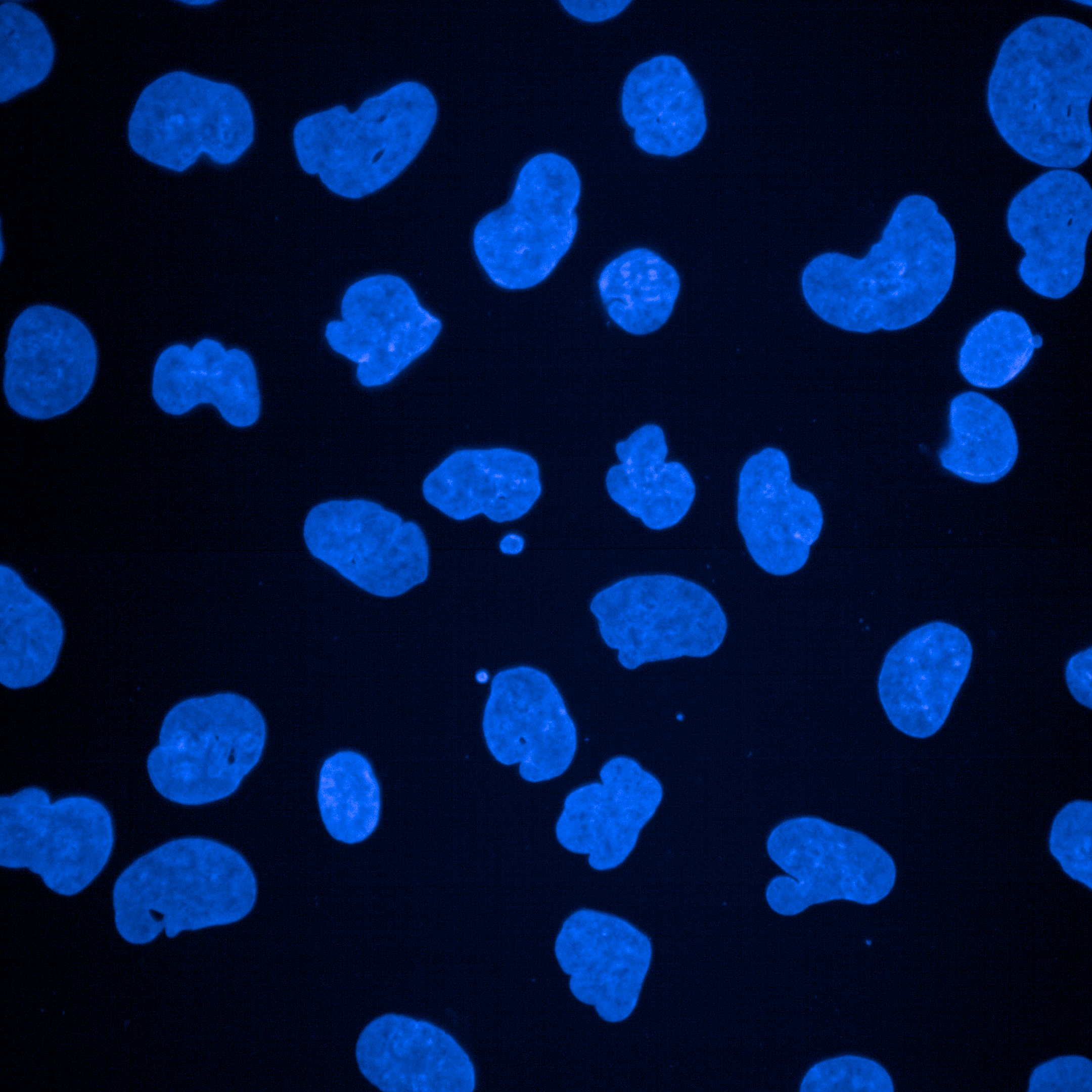

Supplement: Supplementary file 7 — Source data Fig. 4 [file 44318_2024_108_MOESM7_ESM.zip › EMBOJ-2023-115654_Fig4_sourcedata/Figure4G/E231109 HA-TRS PLA 5dC - DAPI.png]

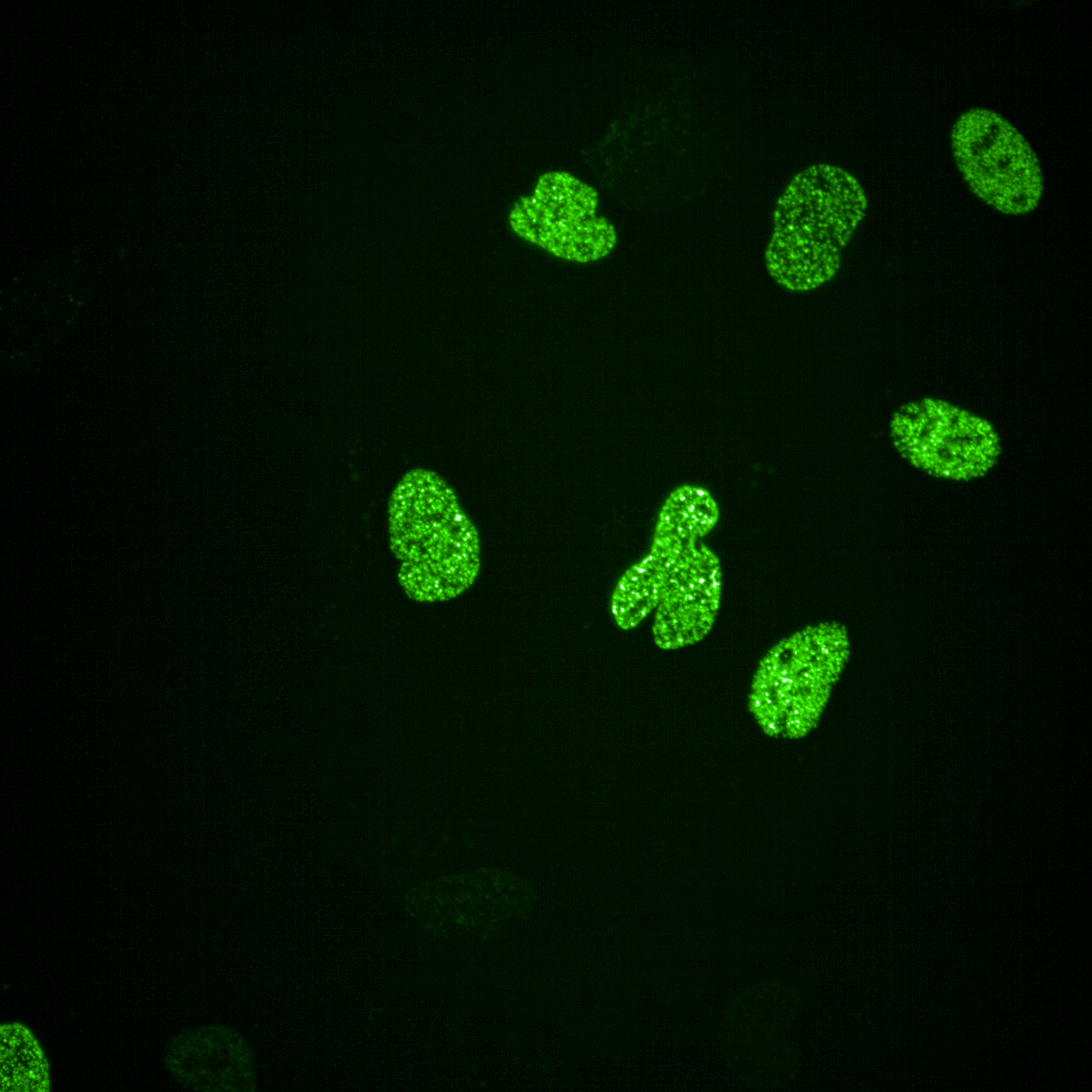

Supplement: Supplementary file 7 — Source data Fig. 4 [file 44318_2024_108_MOESM7_ESM.zip › EMBOJ-2023-115654_Fig4_sourcedata/Figure4G/E231109 HA-TRS gfponly 5dC - GFP.png]

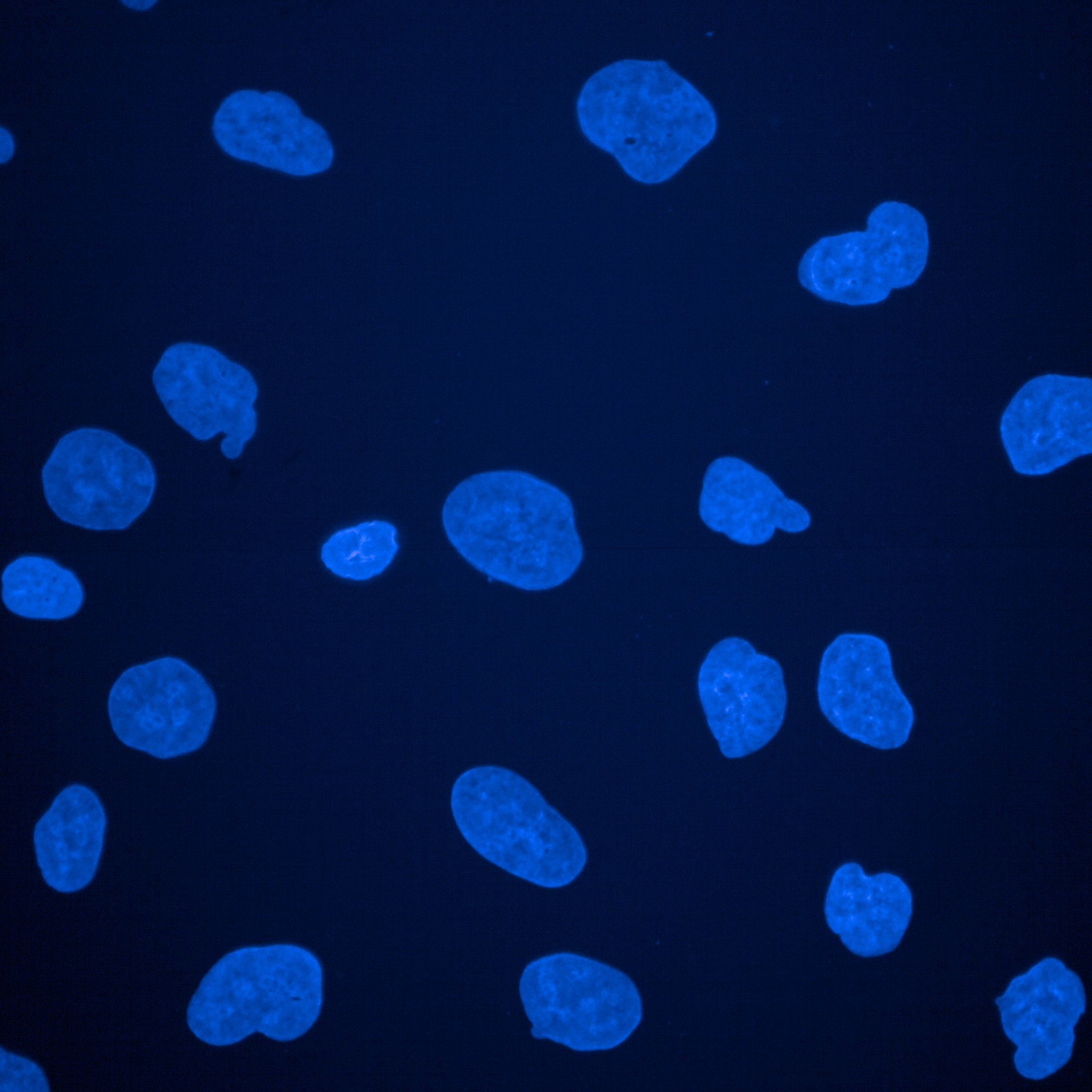

Supplement: Supplementary file 7 — Source data Fig. 4 [file 44318_2024_108_MOESM7_ESM.zip › EMBOJ-2023-115654_Fig4_sourcedata/Figure4G/E231109 HA-TRS PLA dC-SUMOi - DAPI.png]

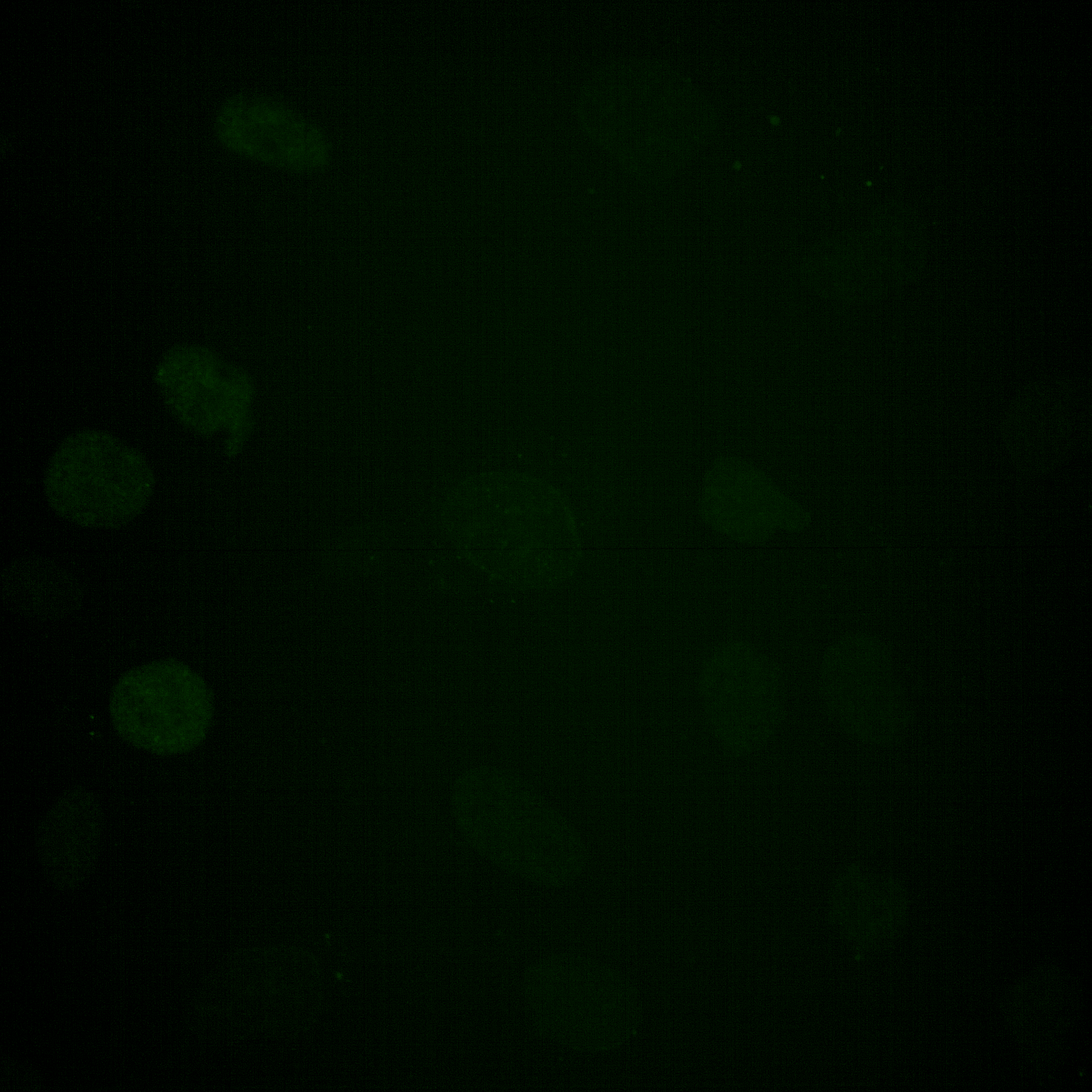

Supplement: Supplementary file 7 — Source data Fig. 4 [file 44318_2024_108_MOESM7_ESM.zip › EMBOJ-2023-115654_Fig4_sourcedata/Figure4G/E231109 HA-TRS PLA dC-SUMOi - GFP.png]

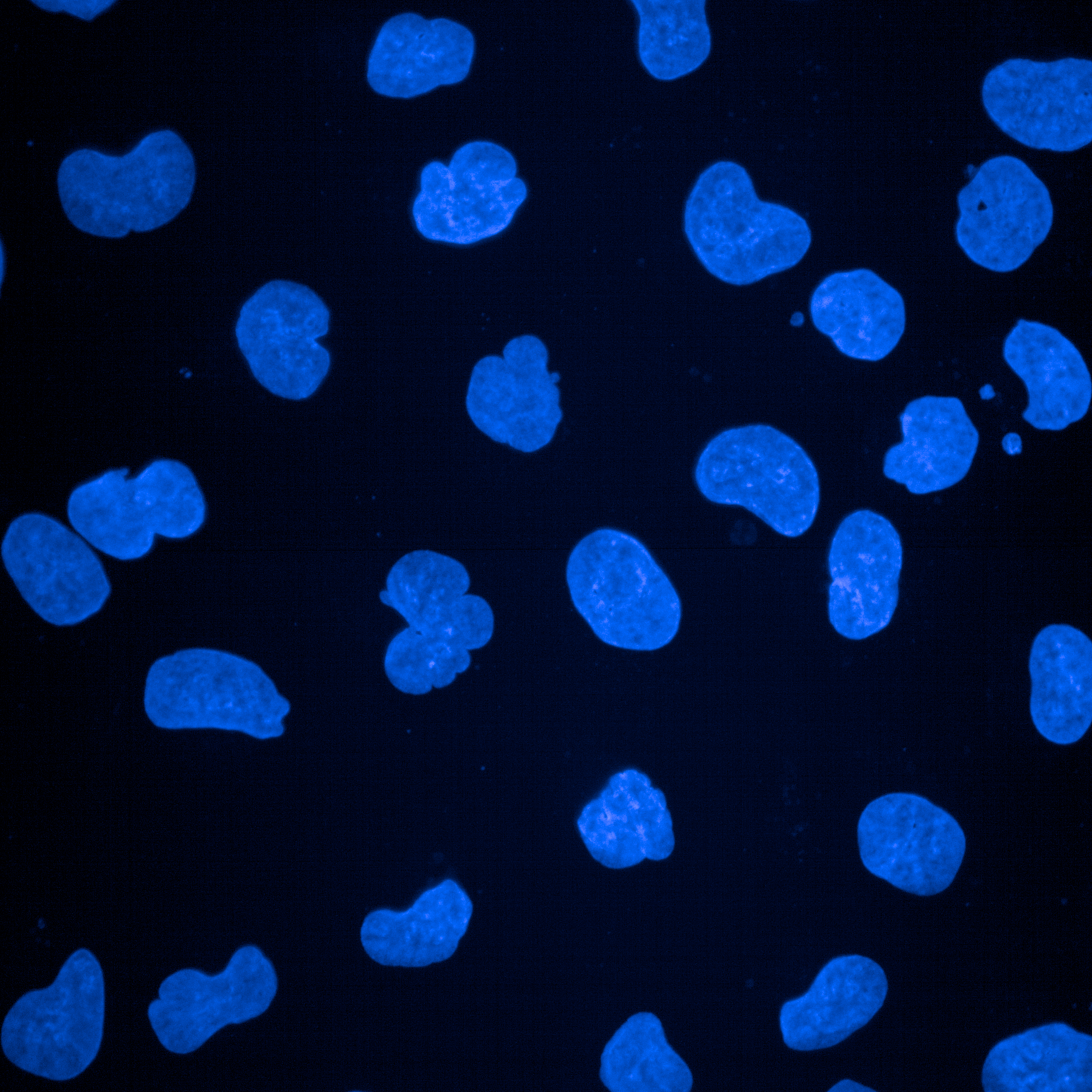

Supplement: Supplementary file 7 — Source data Fig. 4 [file 44318_2024_108_MOESM7_ESM.zip › EMBOJ-2023-115654_Fig4_sourcedata/Figure4G/E231109 HA-TRS PLA dC - DAPI.png]

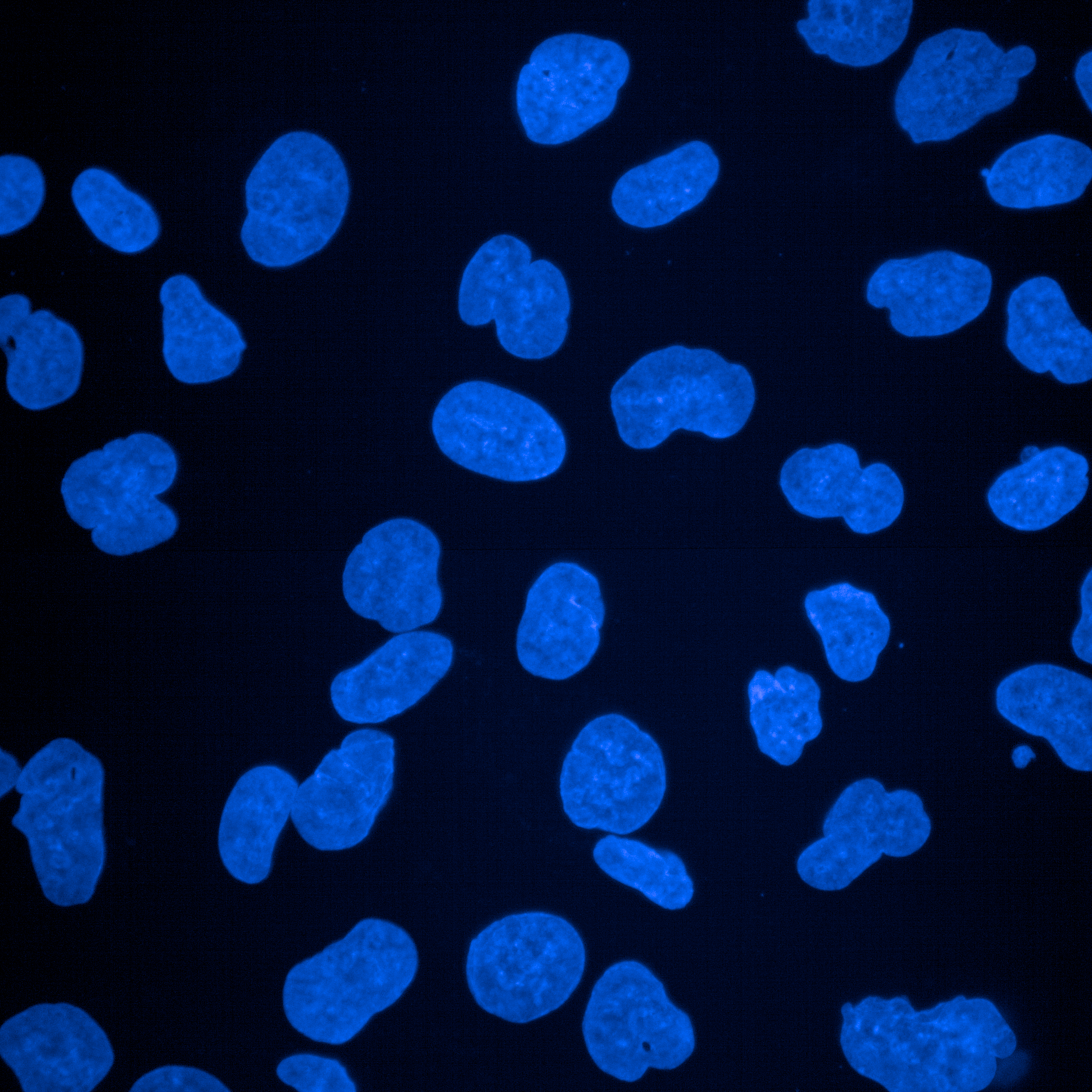

Supplement: Supplementary file 7 — Source data Fig. 4 [file 44318_2024_108_MOESM7_ESM.zip › EMBOJ-2023-115654_Fig4_sourcedata/Figure4G/E231109 HA-TRS PLA 5dC-Ubi - DAPI.png]

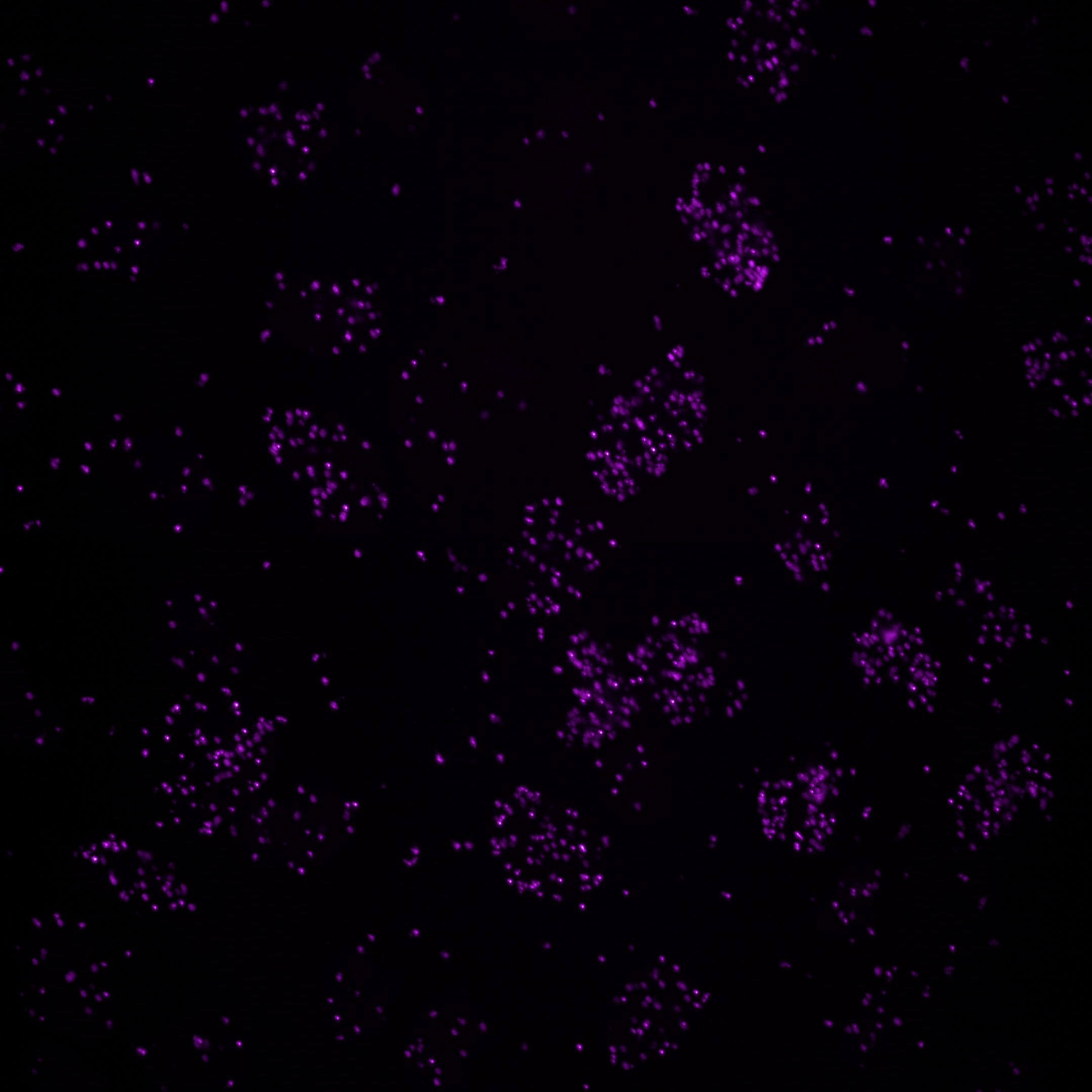

Supplement: Supplementary file 7 — Source data Fig. 4 [file 44318_2024_108_MOESM7_ESM.zip › EMBOJ-2023-115654_Fig4_sourcedata/Figure4G/E231109 HA-TRS PLA 5dC-SUMOi - PLA.png]

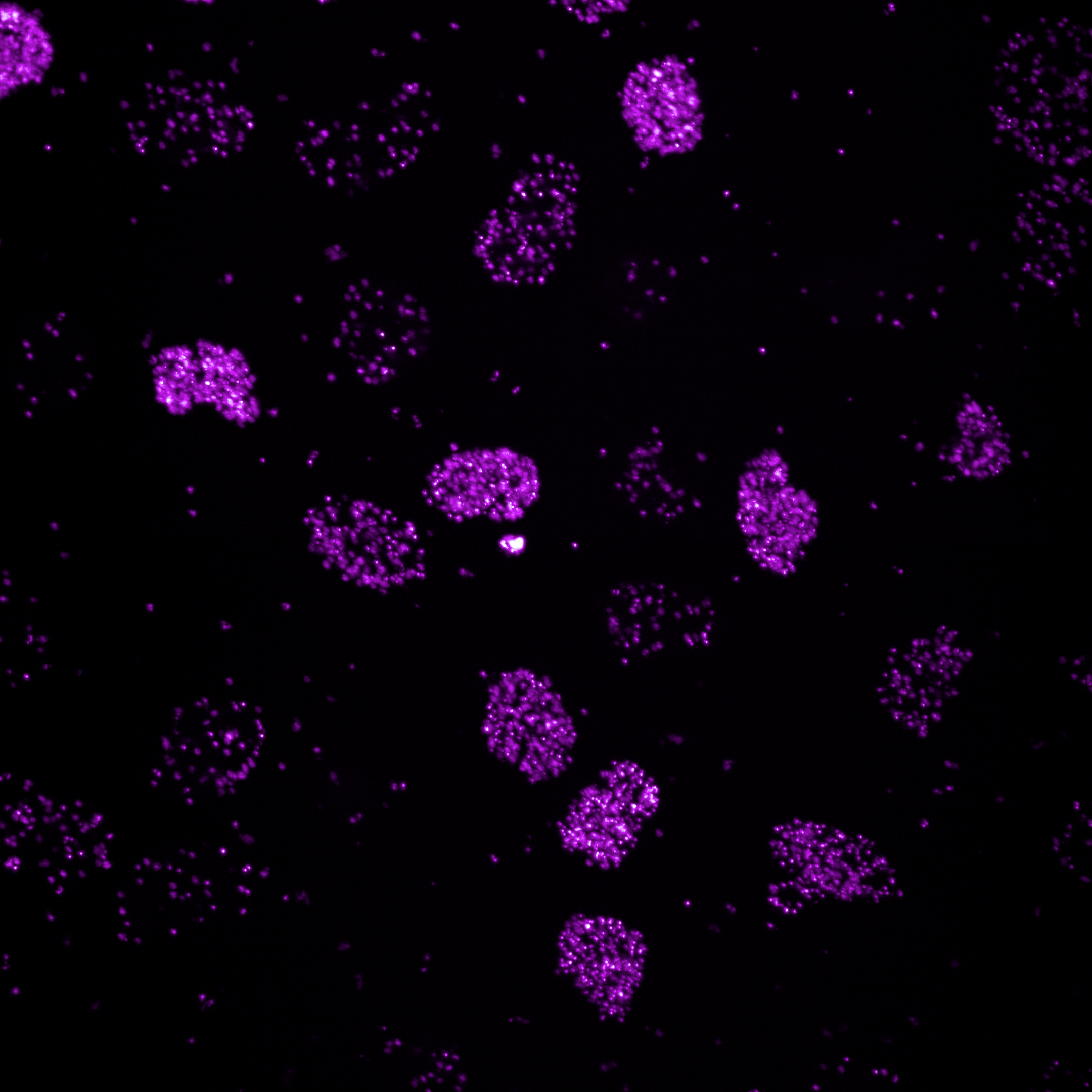

Supplement: Supplementary file 7 — Source data Fig. 4 [file 44318_2024_108_MOESM7_ESM.zip › EMBOJ-2023-115654_Fig4_sourcedata/Figure4G/E231109 HA-TRS PLA 5dC - PLA.png]

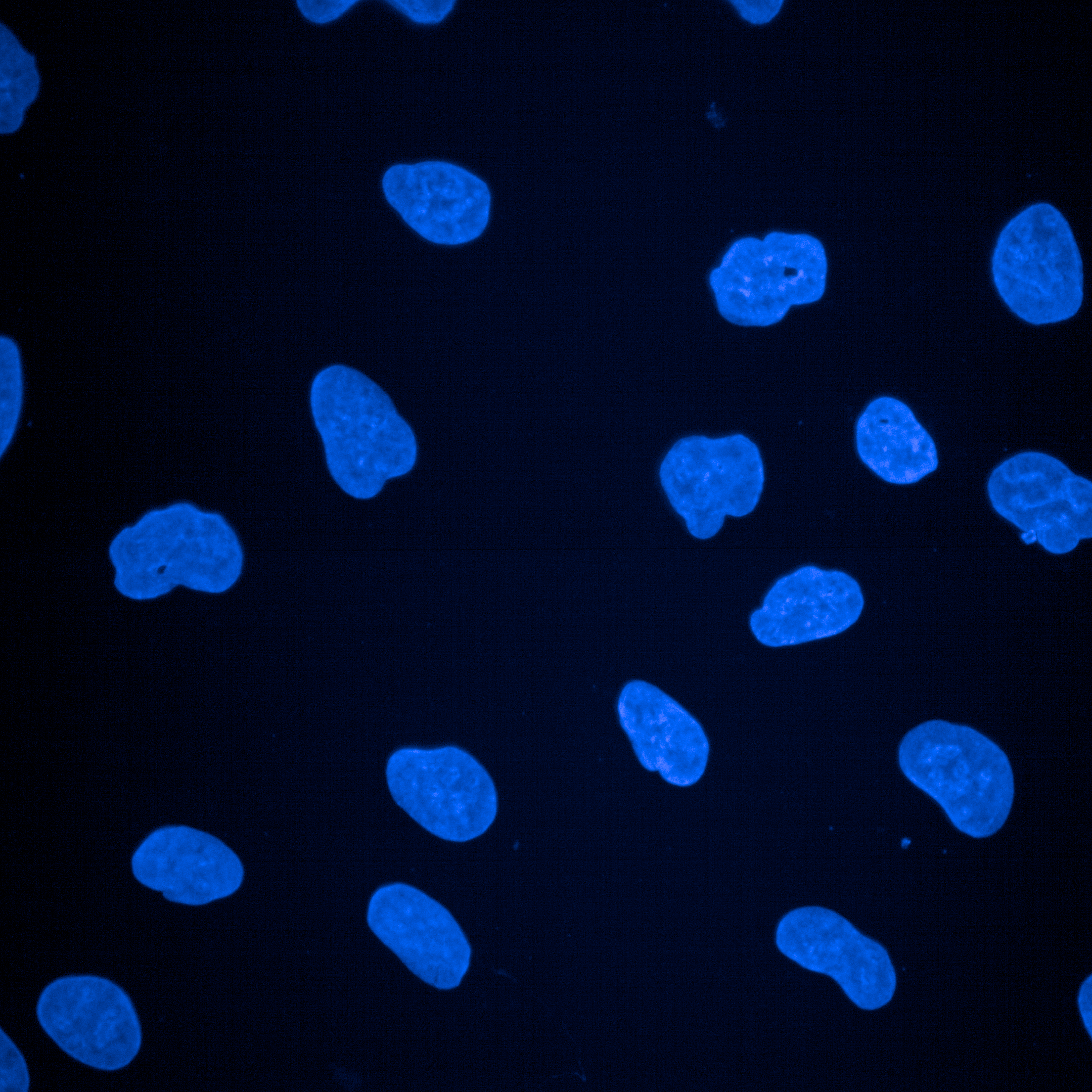

Supplement: Supplementary file 7 — Source data Fig. 4 [file 44318_2024_108_MOESM7_ESM.zip › EMBOJ-2023-115654_Fig4_sourcedata/Figure4G/E231109 HA-TRS PLA dC-Ubi - DAPI.png]

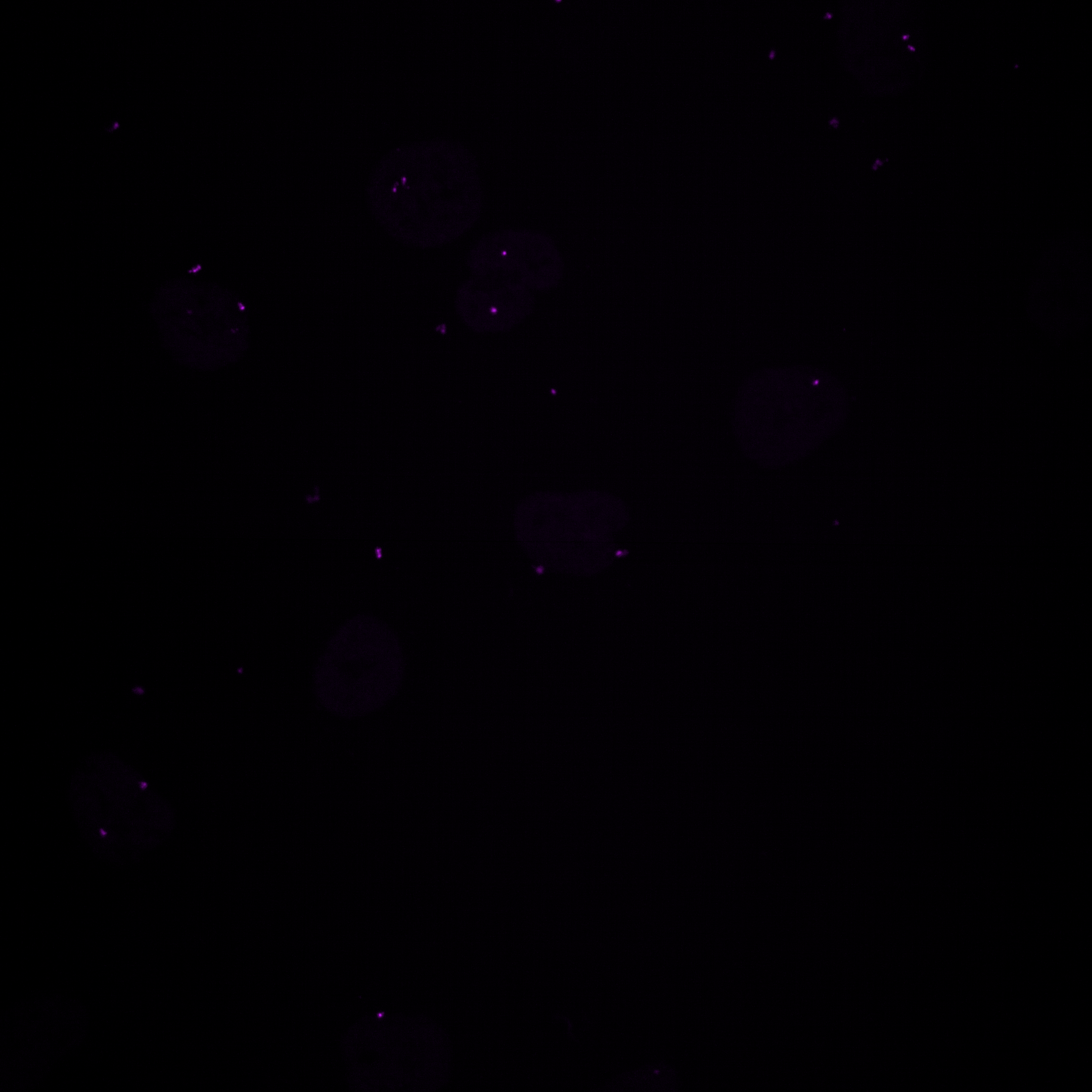

Supplement: Supplementary file 7 — Source data Fig. 4 [file 44318_2024_108_MOESM7_ESM.zip › EMBOJ-2023-115654_Fig4_sourcedata/Figure4G/E231109 HA-TRS HAonly 5dC - PLA.png]

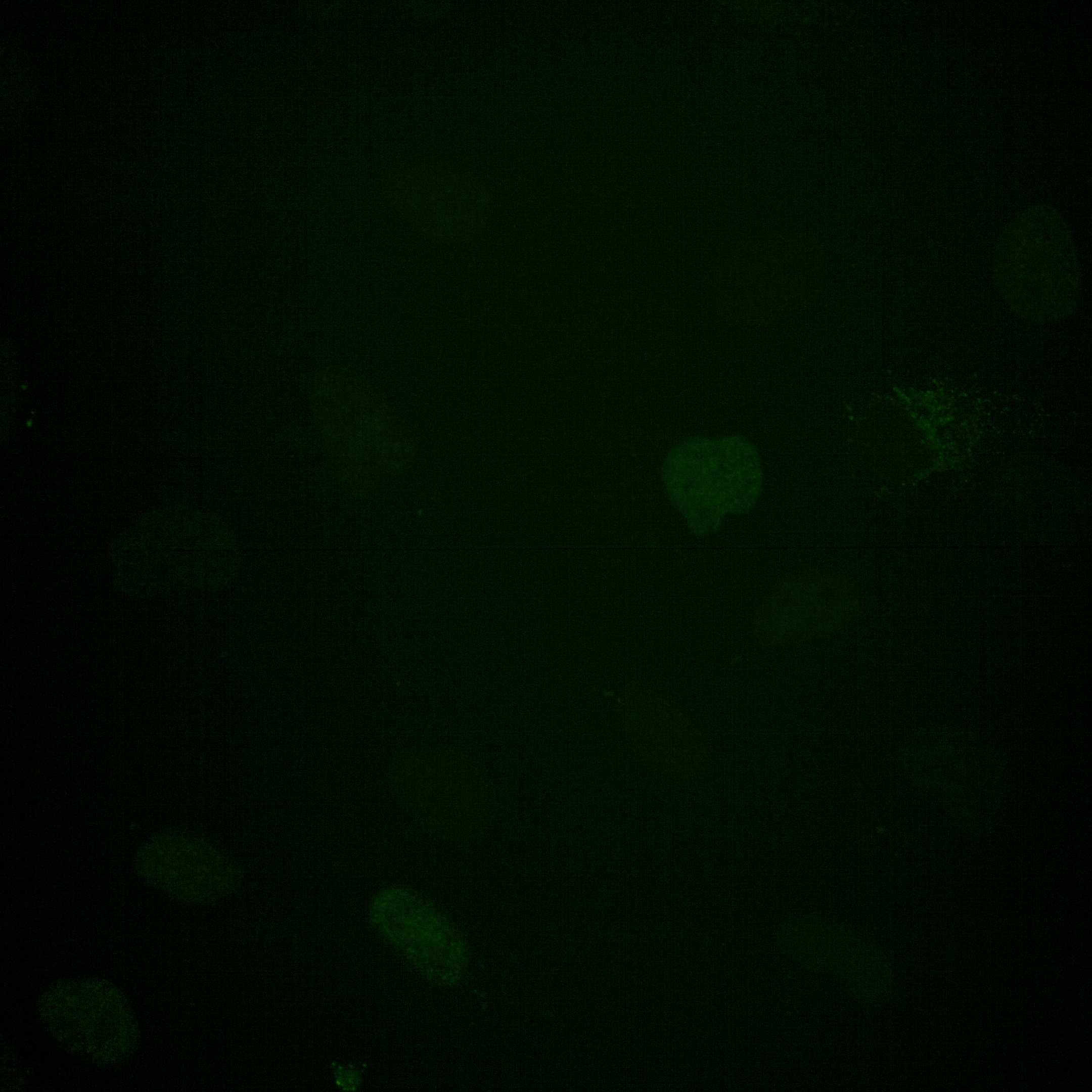

Supplement: Supplementary file 7 — Source data Fig. 4 [file 44318_2024_108_MOESM7_ESM.zip › EMBOJ-2023-115654_Fig4_sourcedata/Figure4G/E231109 HA-TRS PLA dC-Ubi - GFP.png]

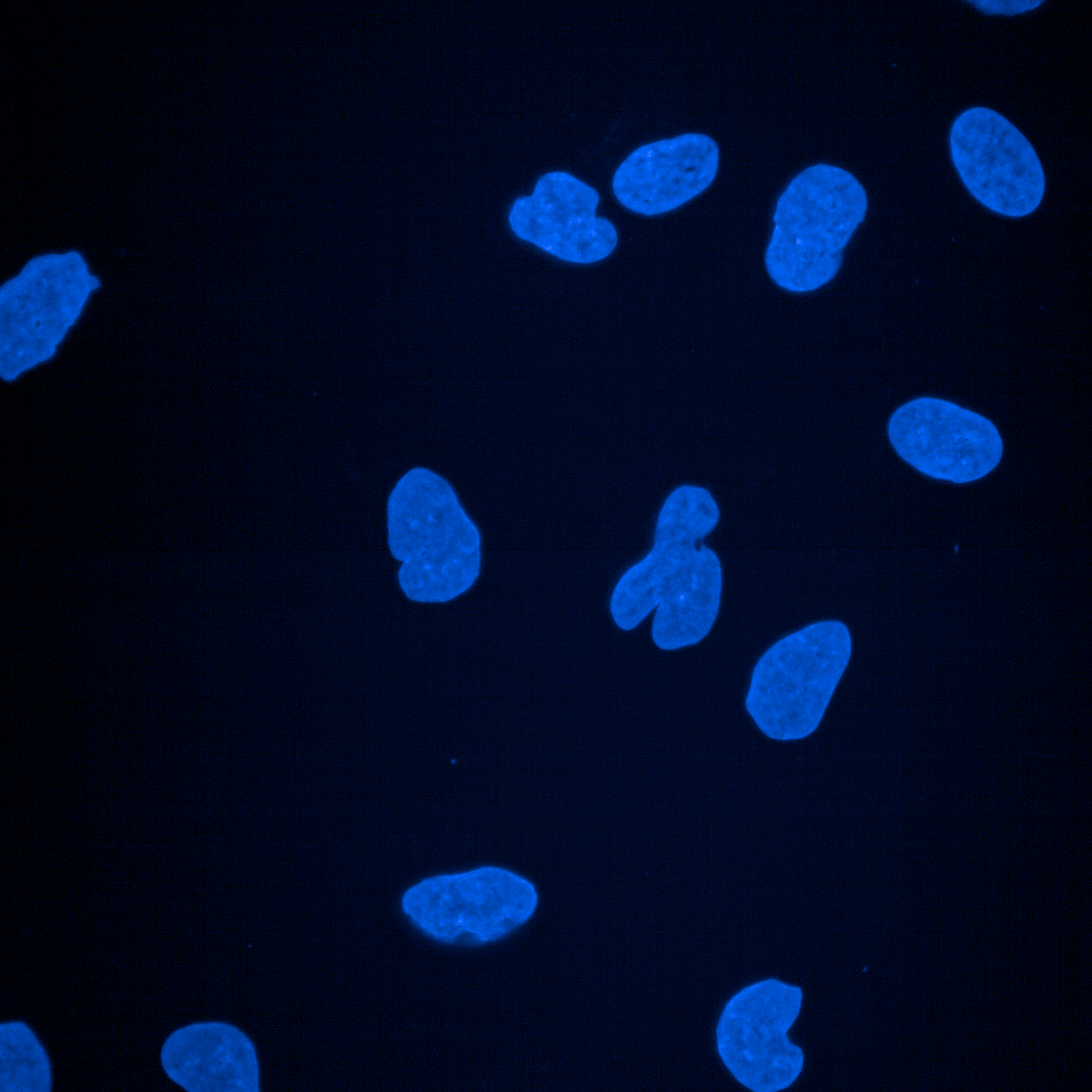

Supplement: Supplementary file 7 — Source data Fig. 4 [file 44318_2024_108_MOESM7_ESM.zip › EMBOJ-2023-115654_Fig4_sourcedata/Figure4G/E231109 HA-TRS gfponly 5dC - DAPI.png]

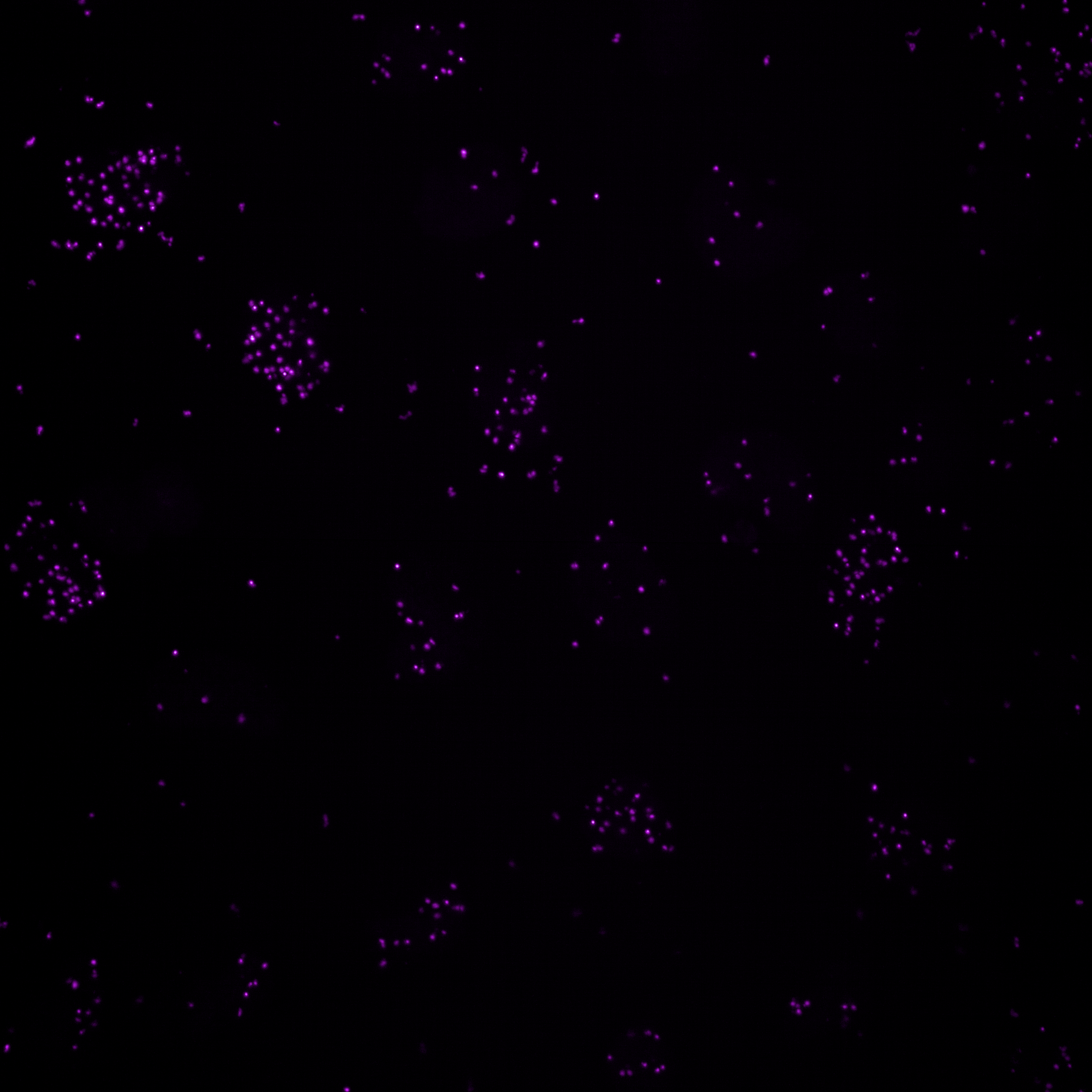

Supplement: Supplementary file 7 — Source data Fig. 4 [file 44318_2024_108_MOESM7_ESM.zip › EMBOJ-2023-115654_Fig4_sourcedata/Figure4G/E231109 HA-TRS PLA dC - PLA.png]

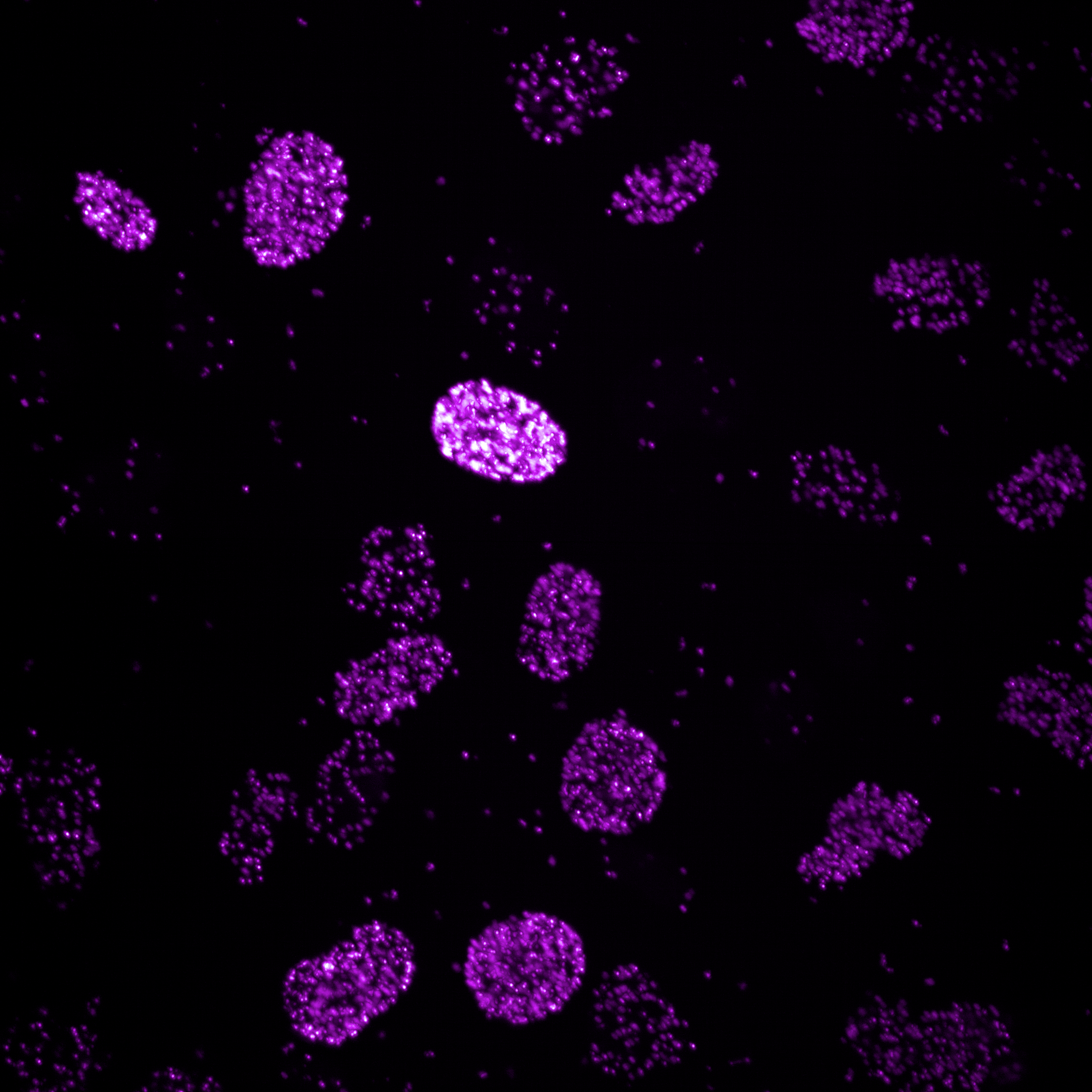

Supplement: Supplementary file 7 — Source data Fig. 4 [file 44318_2024_108_MOESM7_ESM.zip › EMBOJ-2023-115654_Fig4_sourcedata/Figure4G/E231109 HA-TRS PLA 5dC-Ubi - PLA.png]

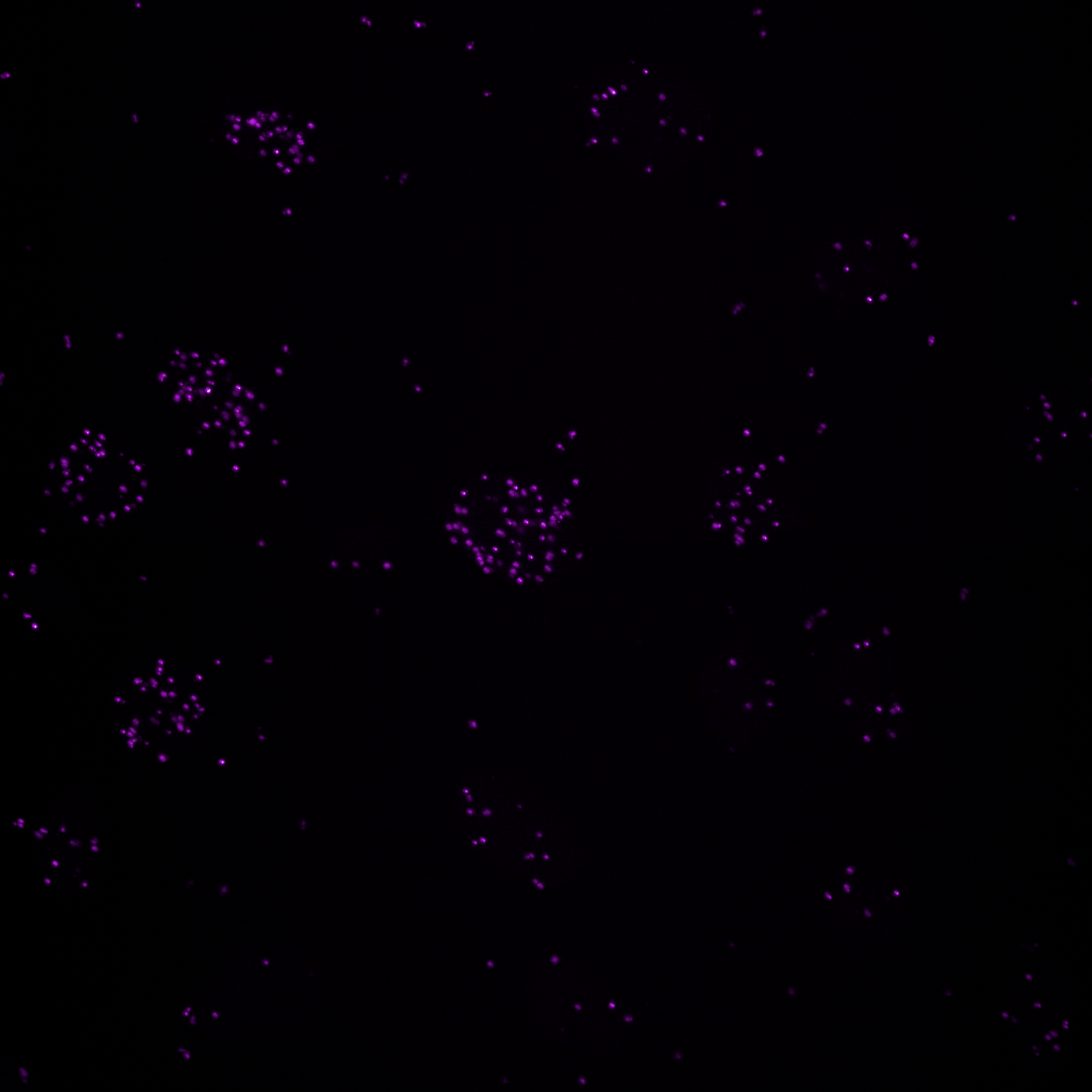

Supplement: Supplementary file 7 — Source data Fig. 4 [file 44318_2024_108_MOESM7_ESM.zip › EMBOJ-2023-115654_Fig4_sourcedata/Figure4G/E231109 HA-TRS PLA dC-SUMOi - PLA.png]

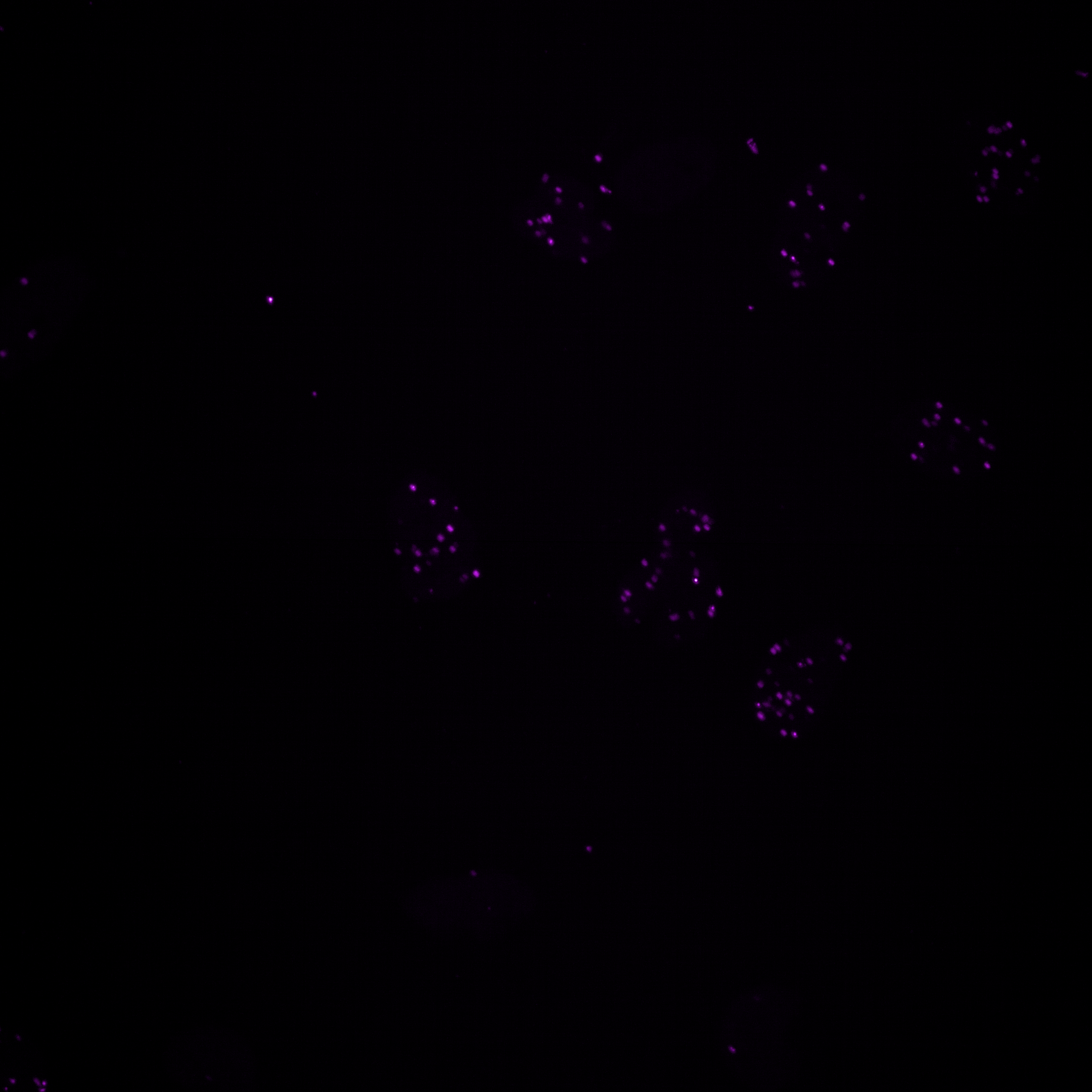

Supplement: Supplementary file 7 — Source data Fig. 4 [file 44318_2024_108_MOESM7_ESM.zip › EMBOJ-2023-115654_Fig4_sourcedata/Figure4G/E231109 HA-TRS gfponly 5dC - PLA.png]

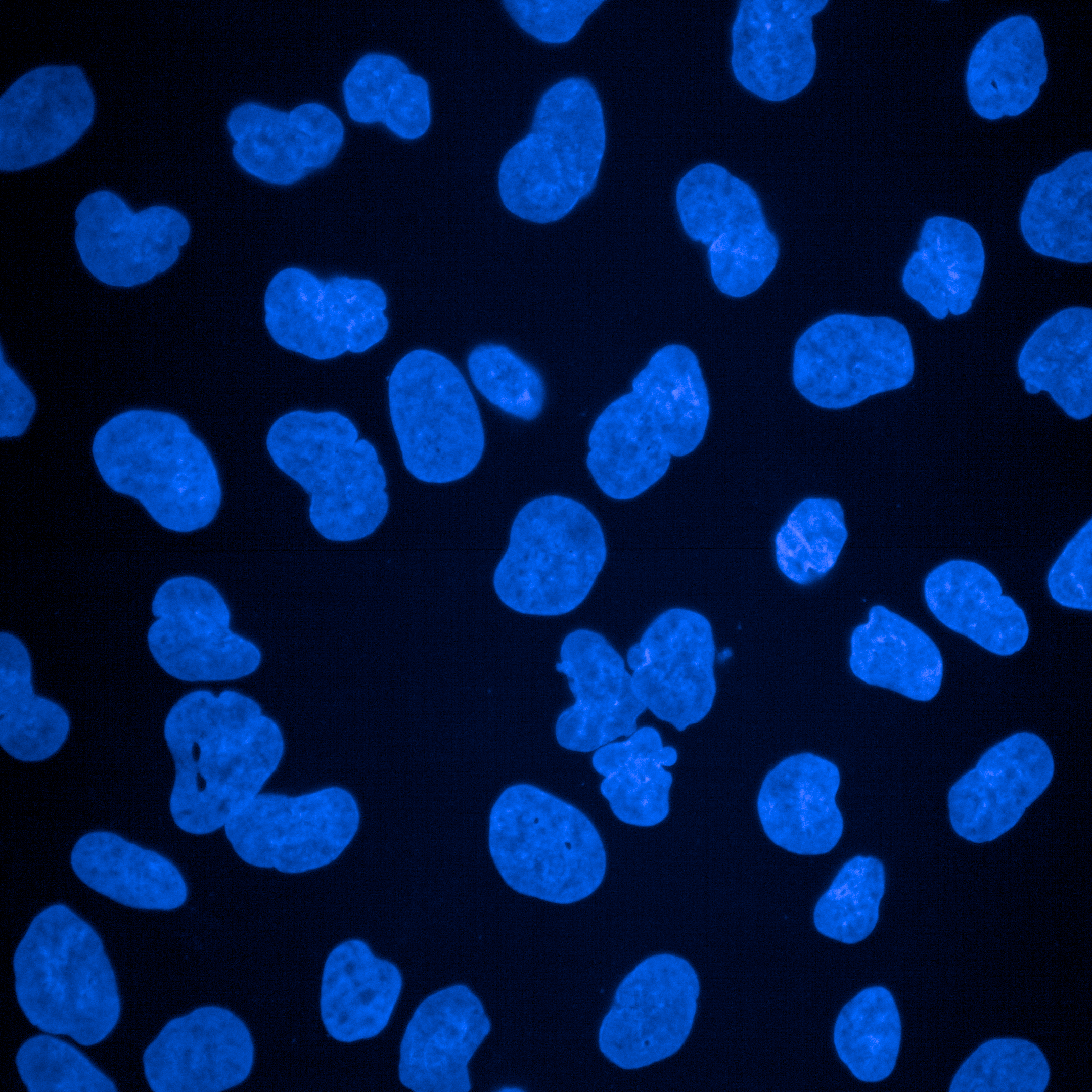

Supplement: Supplementary file 7 — Source data Fig. 4 [file 44318_2024_108_MOESM7_ESM.zip › EMBOJ-2023-115654_Fig4_sourcedata/Figure4G/E231109 HA-TRS PLA 5dC-SUMOi - DAPI.png]

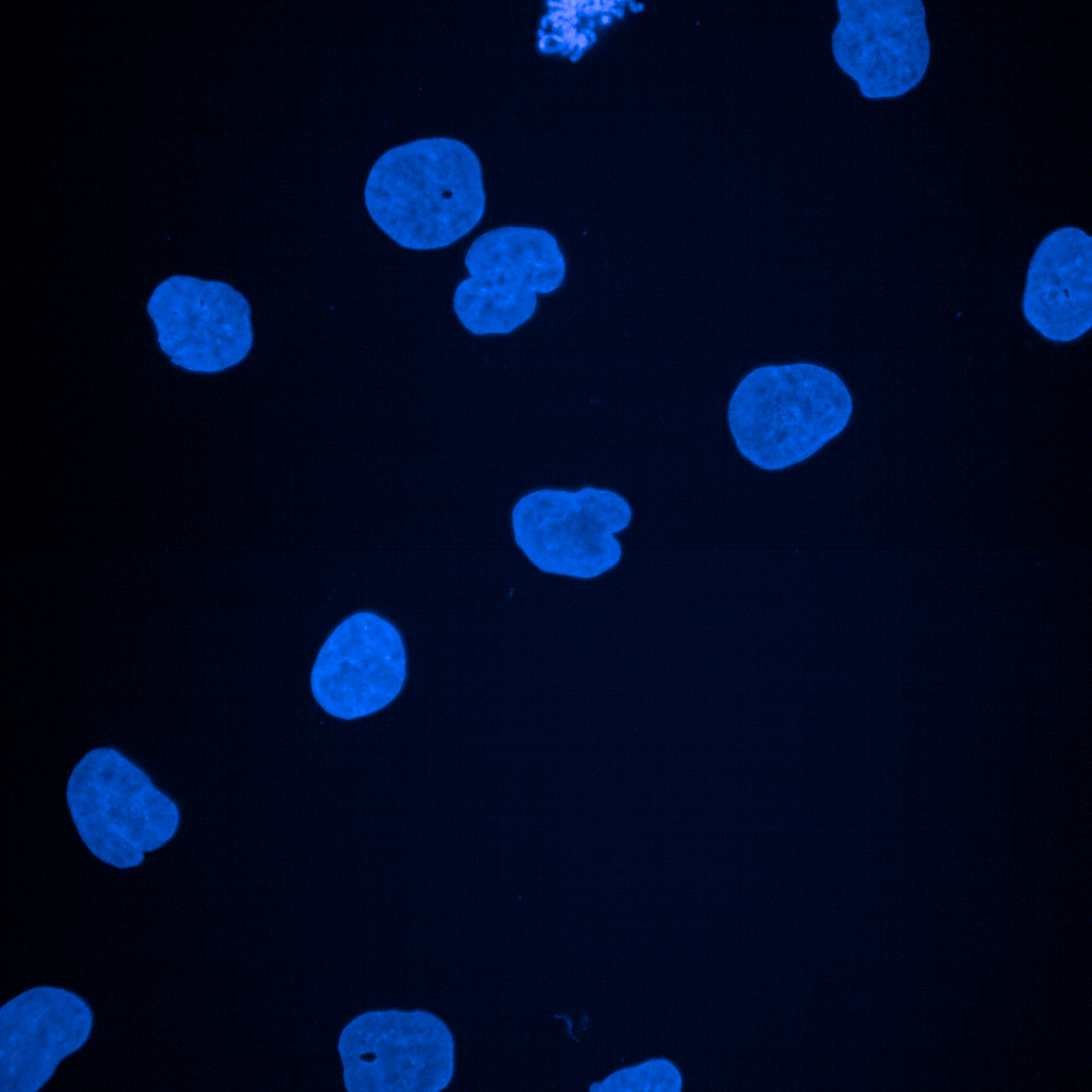

Supplement: Supplementary file 7 — Source data Fig. 4 [file 44318_2024_108_MOESM7_ESM.zip › EMBOJ-2023-115654_Fig4_sourcedata/Figure4G/E231109 HA-TRS HAonly 5dC - DAPI.png]

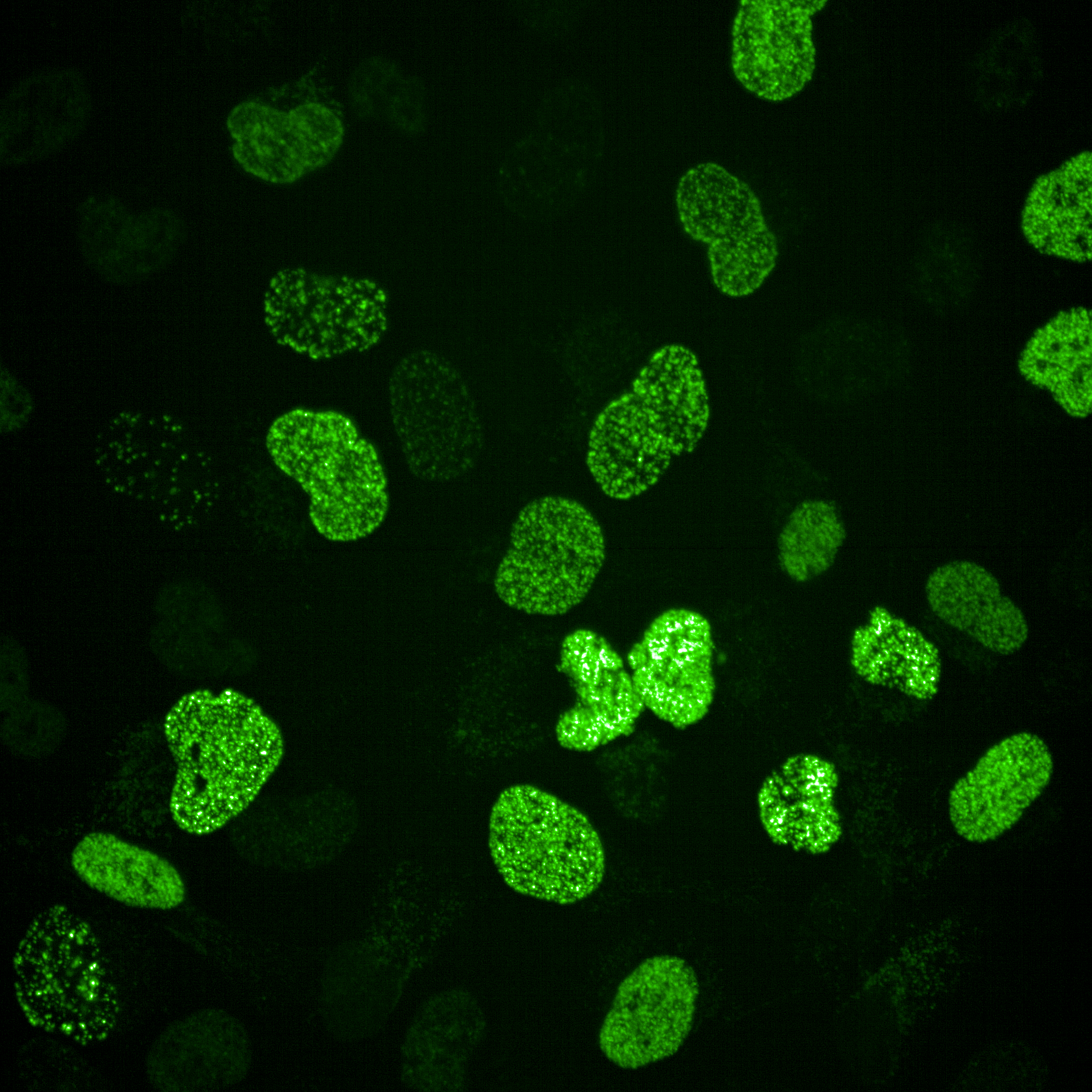

Supplement: Supplementary file 7 — Source data Fig. 4 [file 44318_2024_108_MOESM7_ESM.zip › EMBOJ-2023-115654_Fig4_sourcedata/Figure4G/E231109 HA-TRS PLA 5dC-SUMOi - GFP.png]

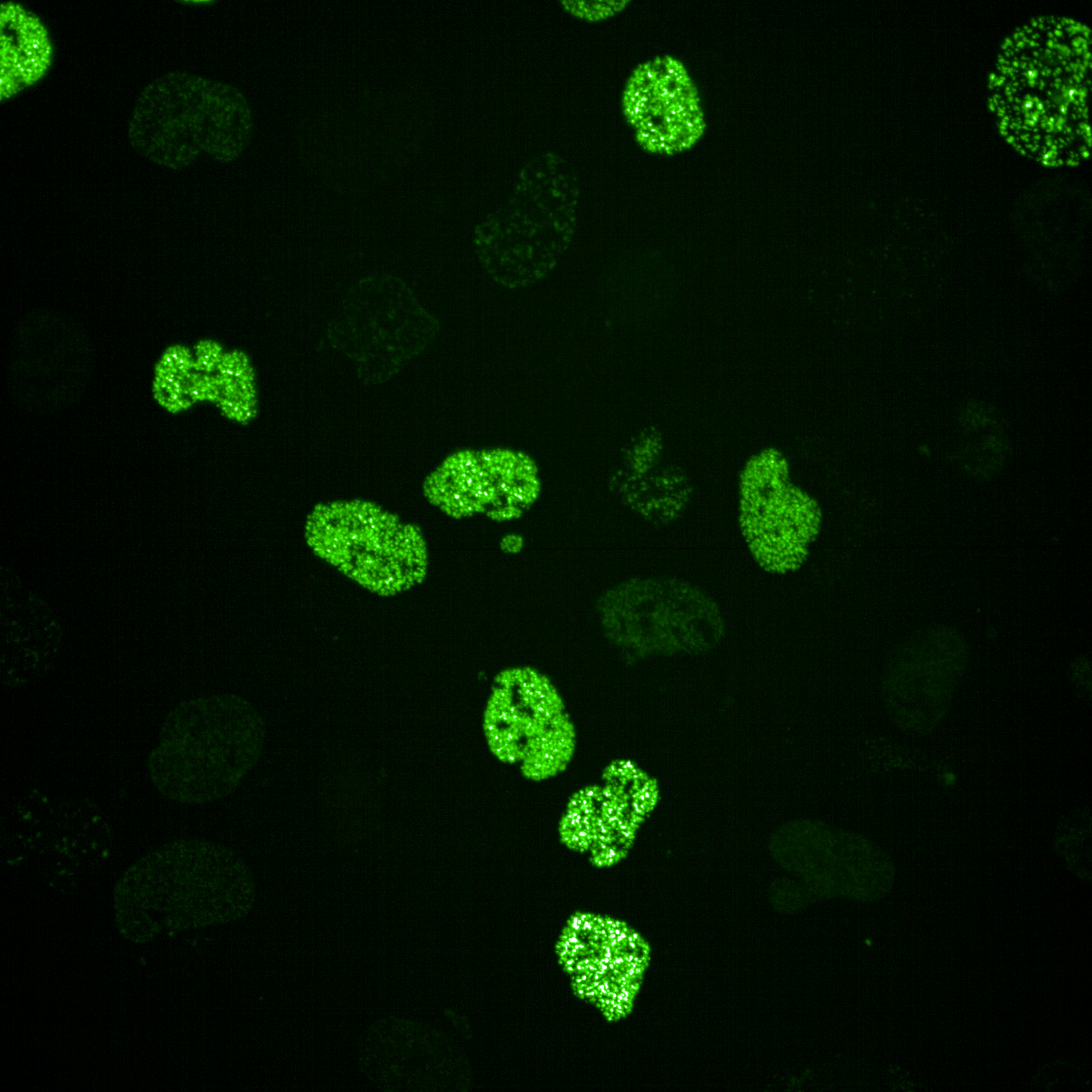

Supplement: Supplementary file 7 — Source data Fig. 4 [file 44318_2024_108_MOESM7_ESM.zip › EMBOJ-2023-115654_Fig4_sourcedata/Figure4G/E231109 HA-TRS PLA 5dC - GFP.png]

Source data: Figure 5D.

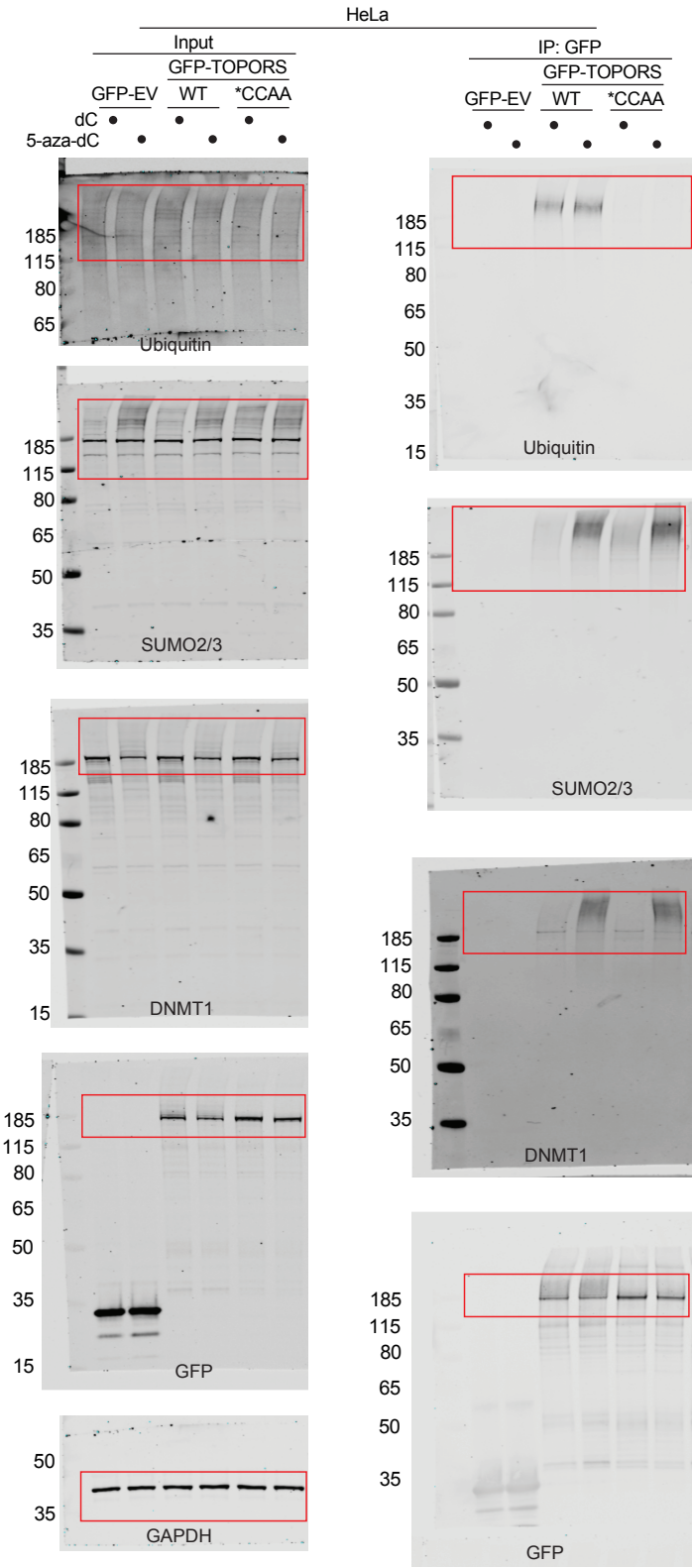

Supplement: Supplementary file 8 — Source data Fig. 5 [file 44318_2024_108_MOESM8_ESM.zip › EMBOJ-2023-115654_Fig5_sourcedata/Figure5D.pdf]

Source data: Figure 5E.

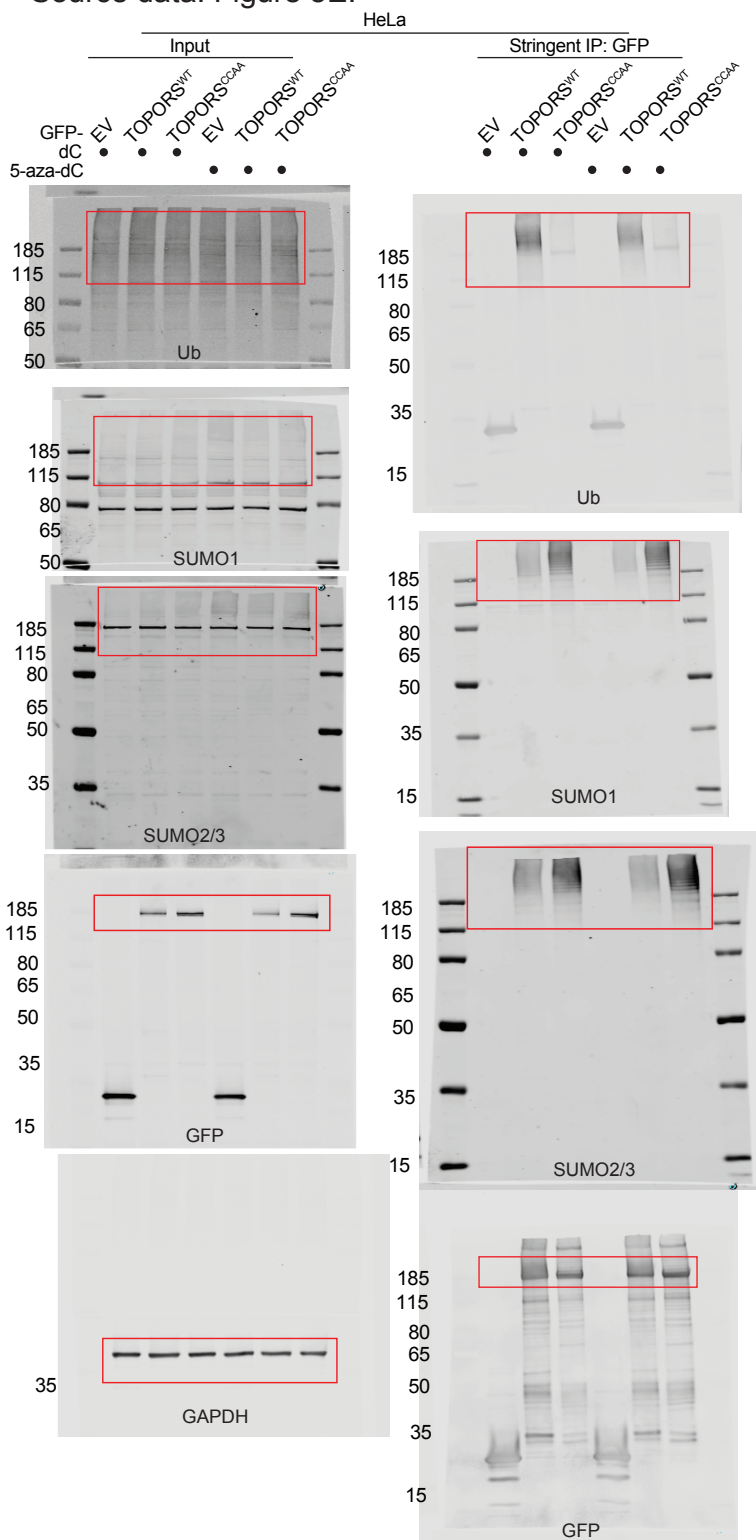

Supplement: Supplementary file 8 — Source data Fig. 5 [file 44318_2024_108_MOESM8_ESM.zip › EMBOJ-2023-115654_Fig5_sourcedata/Figure5E.pdf]

Source data: Figure 5F.

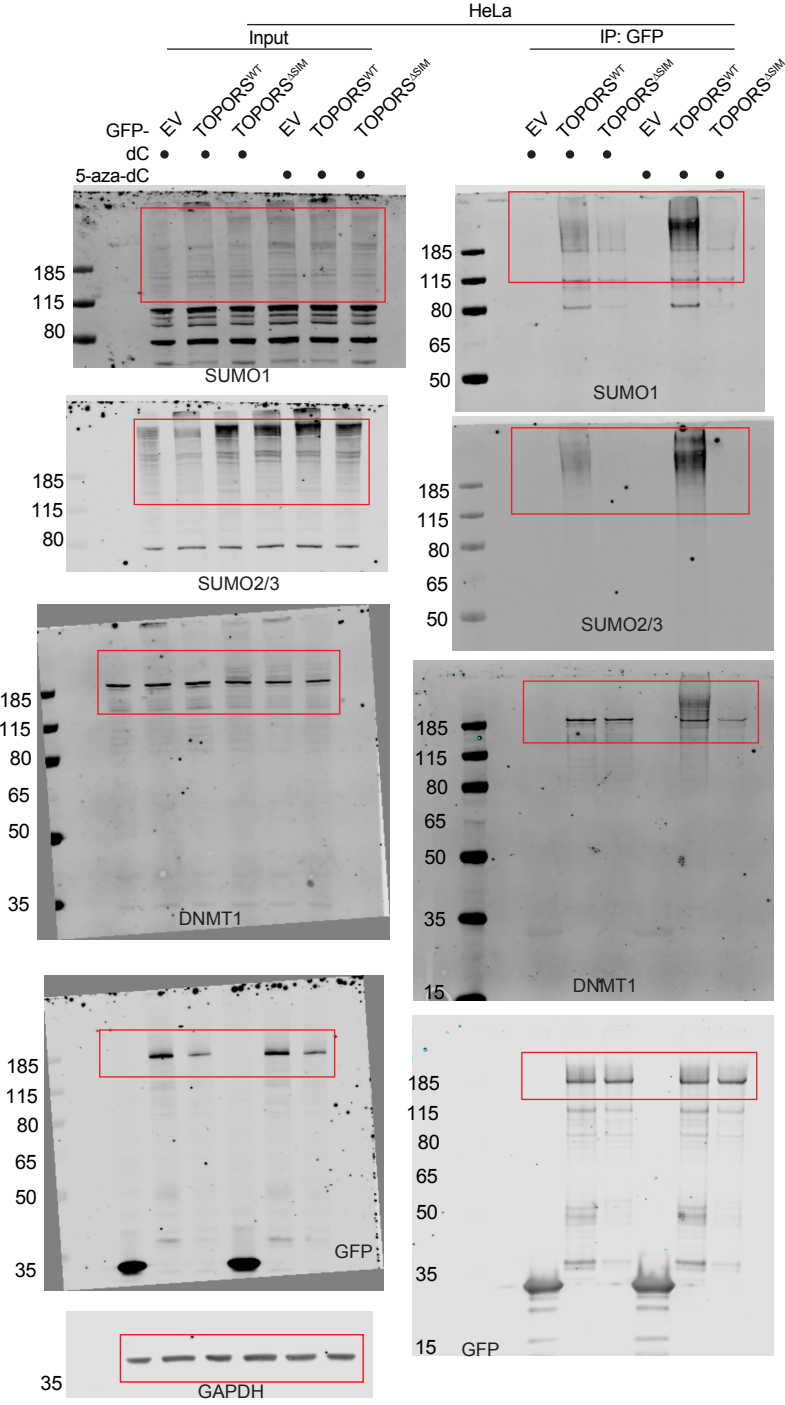

Supplement: Supplementary file 8 — Source data Fig. 5 [file 44318_2024_108_MOESM8_ESM.zip › EMBOJ-2023-115654_Fig5_sourcedata/Figure5F.pdf]

Source data: Figure 6H.

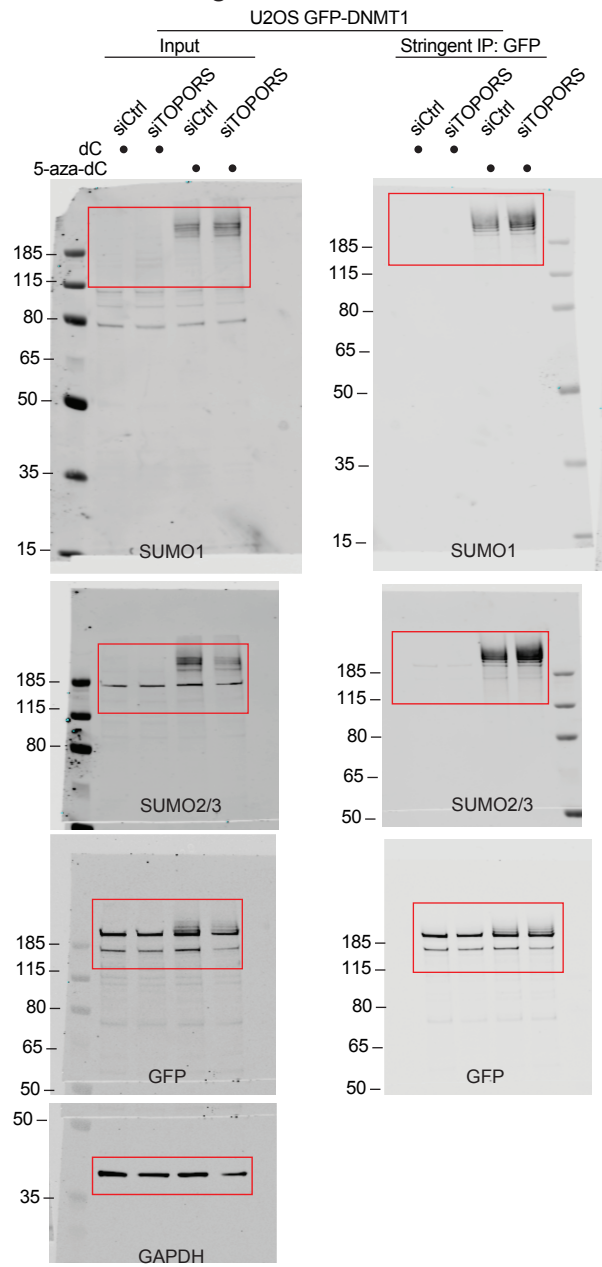

Supplement: Supplementary file 9 — Source data Fig. 6 [file 44318_2024_108_MOESM9_ESM.zip › EMBOJ-2023-115654_Fig6_sourcedata/Figure6H.pdf]

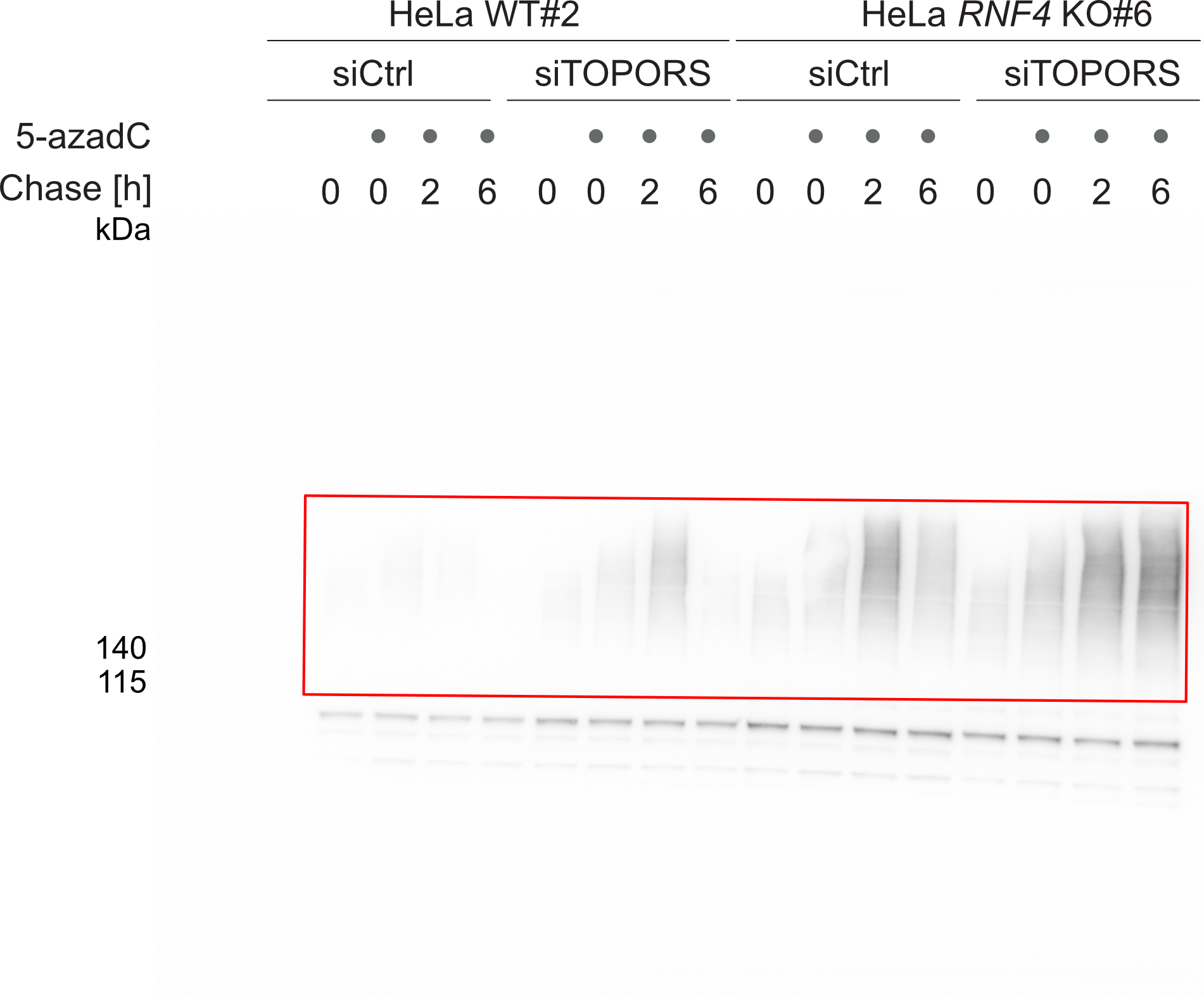

Supplement: Supplementary file 9 — Source data Fig. 6 [file 44318_2024_108_MOESM9_ESM.zip › EMBOJ-2023-115654_Fig6_sourcedata/Figure6G/western SUMO1 Input.tiff]

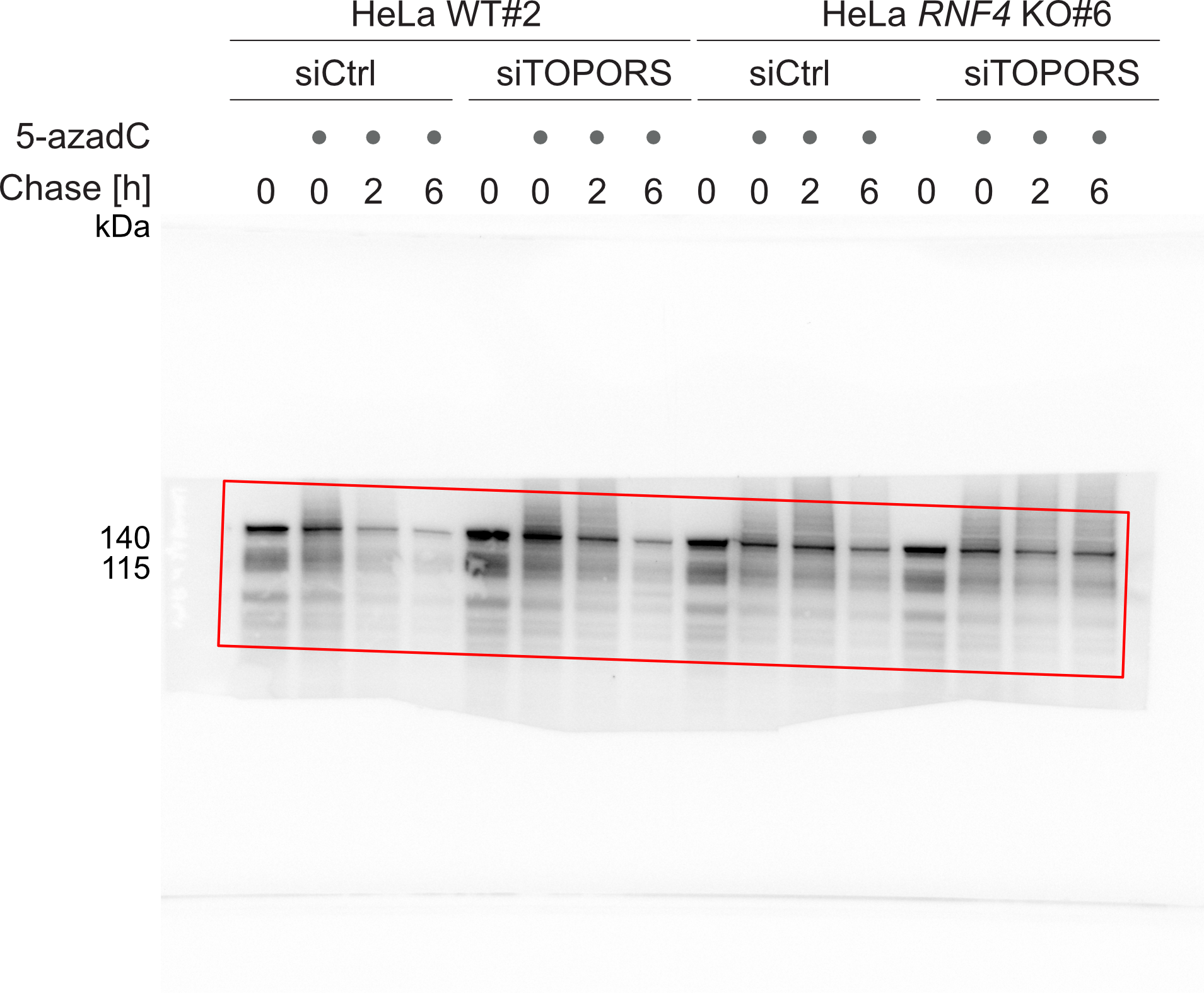

Supplement: Supplementary file 9 — Source data Fig. 6 [file 44318_2024_108_MOESM9_ESM.zip › EMBOJ-2023-115654_Fig6_sourcedata/Figure6G/western blot DNMT1 input.tiff]

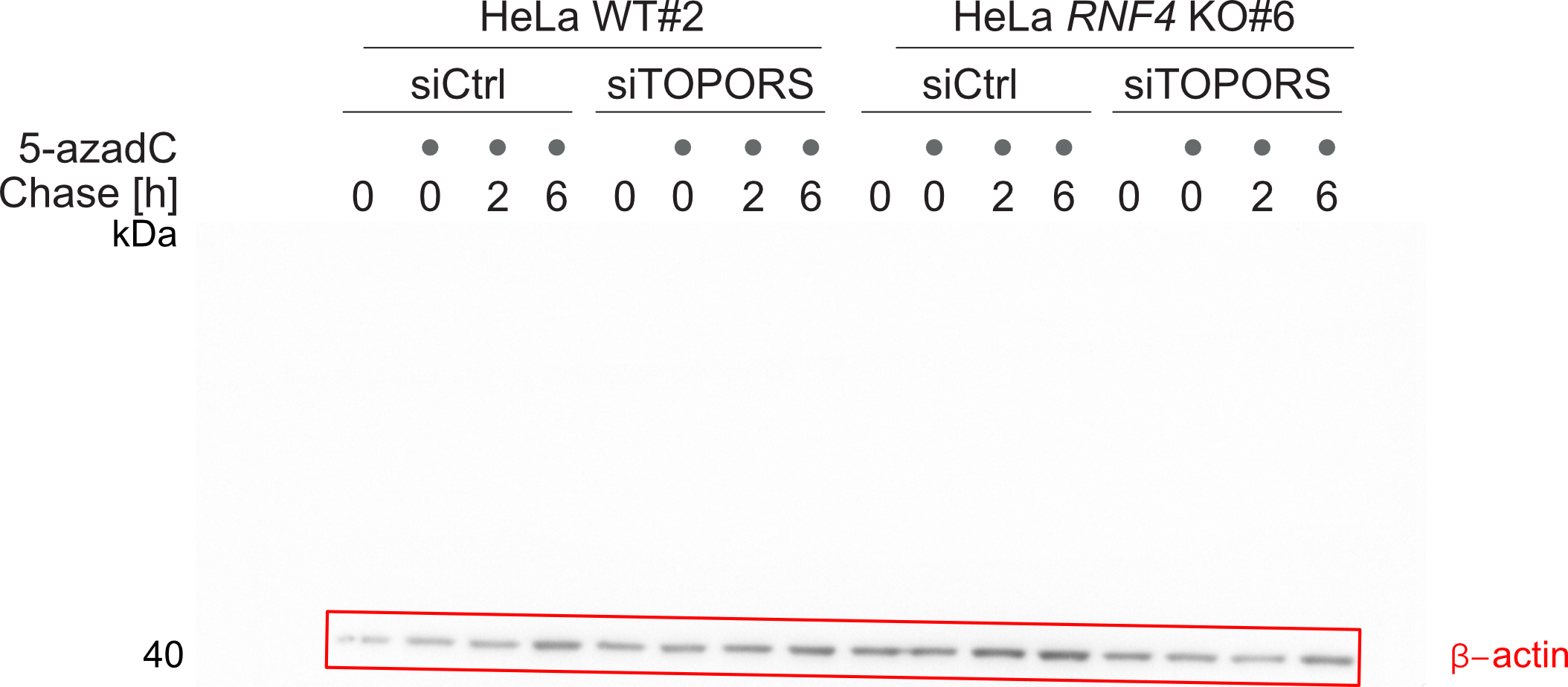

Supplement: Supplementary file 9 — Source data Fig. 6 [file 44318_2024_108_MOESM9_ESM.zip › EMBOJ-2023-115654_Fig6_sourcedata/Figure6G/western blot beta actin.tiff]

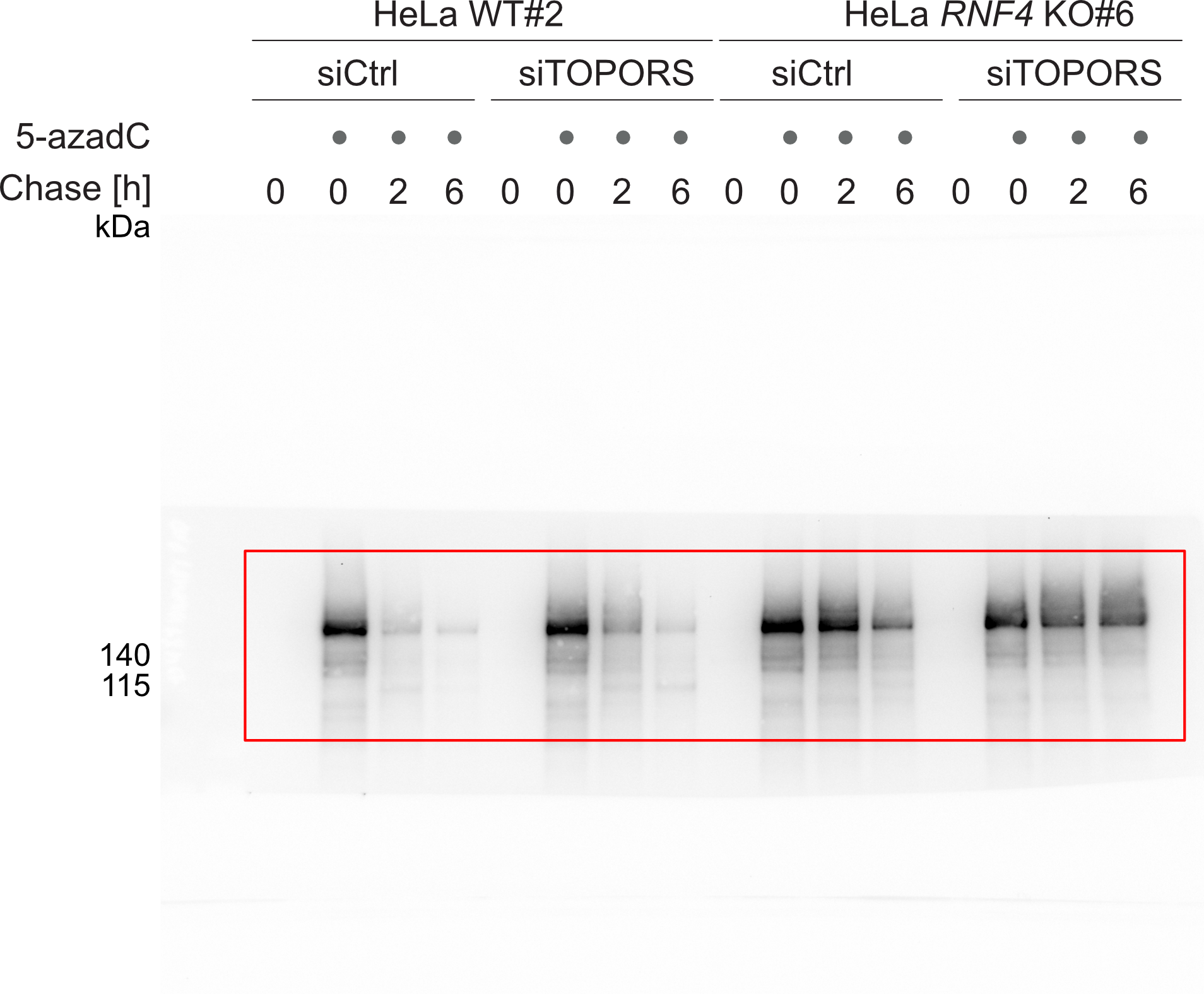

Supplement: Supplementary file 9 — Source data Fig. 6 [file 44318_2024_108_MOESM9_ESM.zip › EMBOJ-2023-115654_Fig6_sourcedata/Figure6G/western blot DNMT1 PxP.tiff]

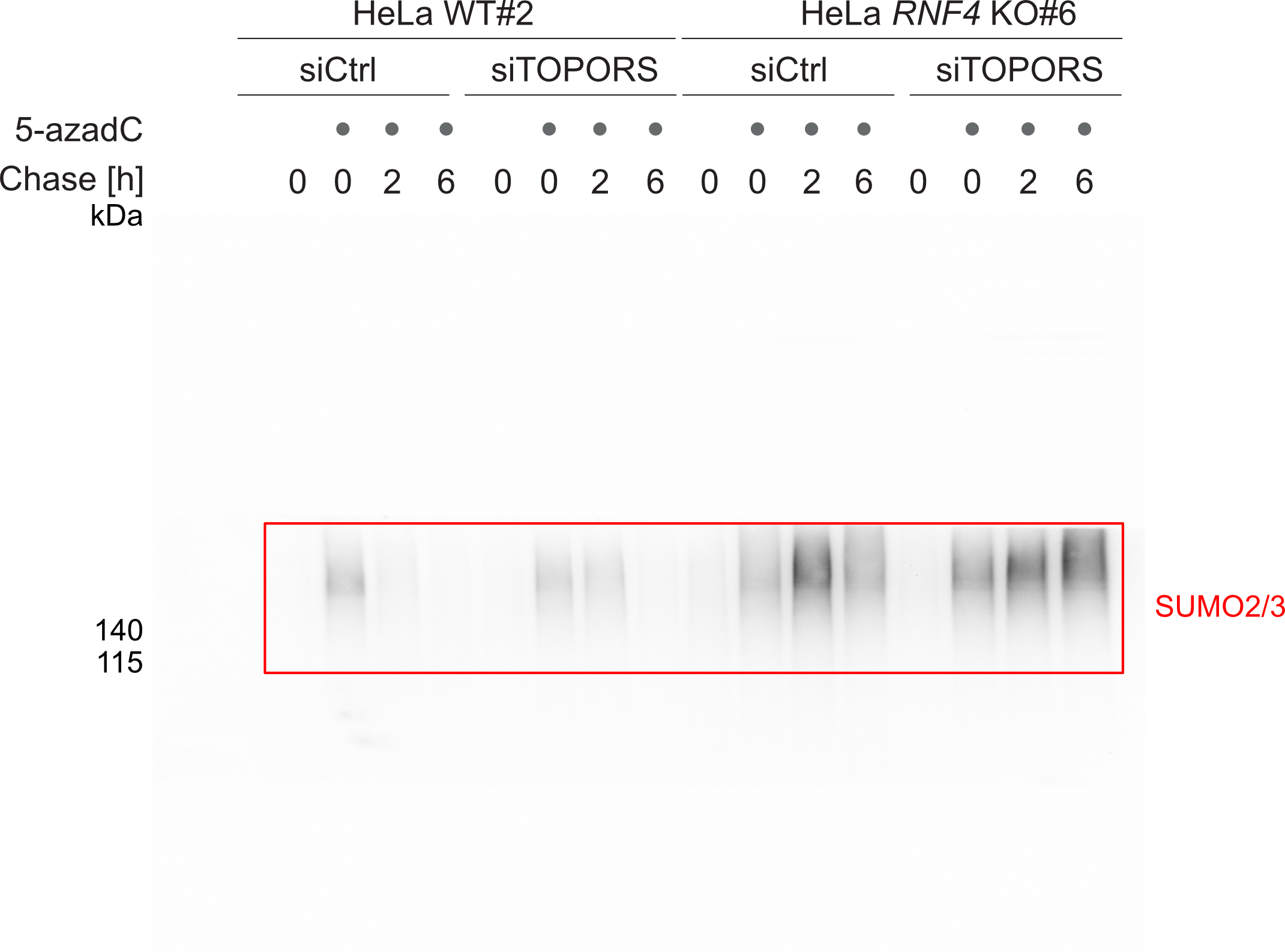

Supplement: Supplementary file 9 — Source data Fig. 6 [file 44318_2024_108_MOESM9_ESM.zip › EMBOJ-2023-115654_Fig6_sourcedata/Figure6G/western blot SUMO23 PxP.tiff]

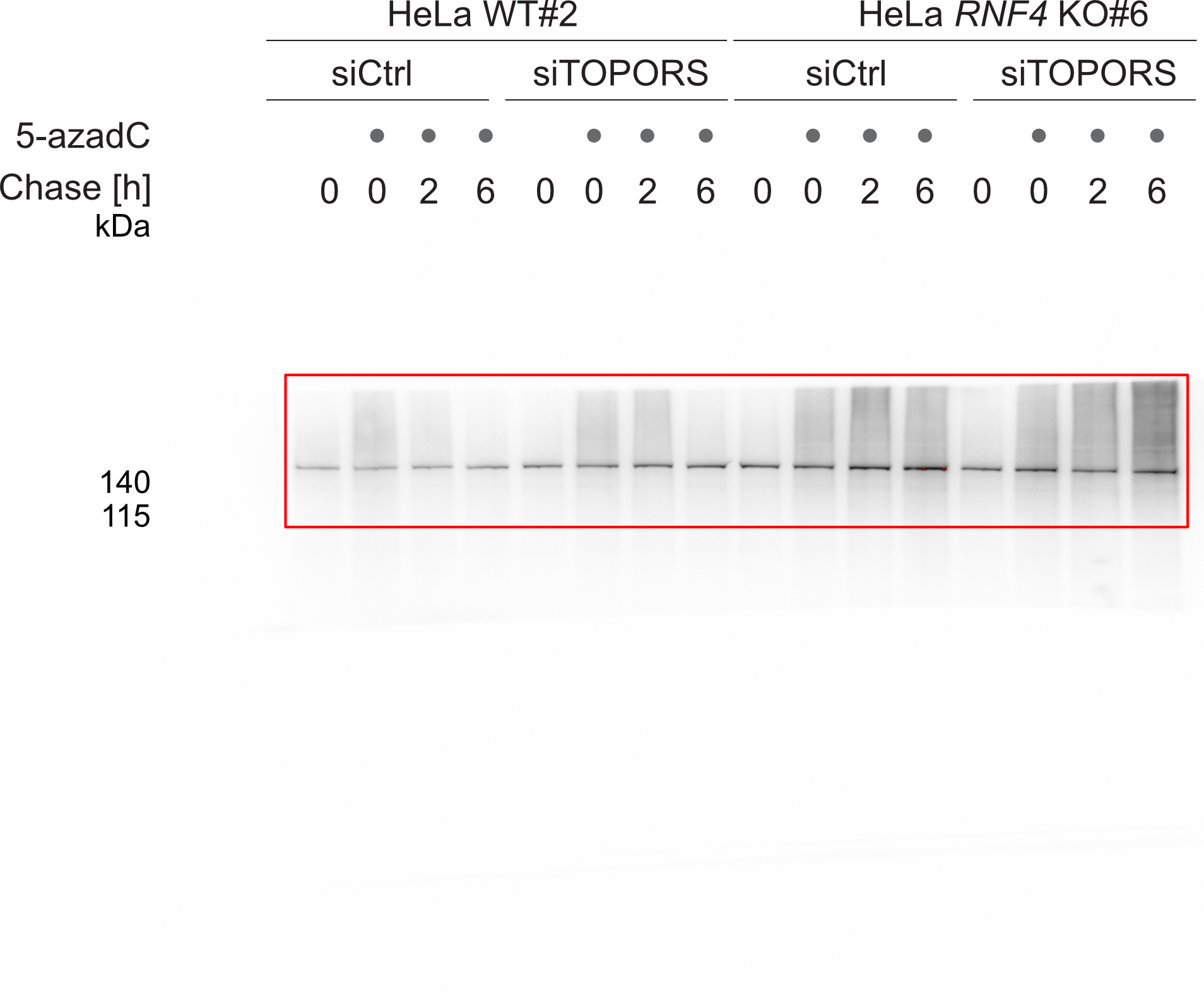

Supplement: Supplementary file 9 — Source data Fig. 6 [file 44318_2024_108_MOESM9_ESM.zip › EMBOJ-2023-115654_Fig6_sourcedata/Figure6G/western blot SUMO23 Input.tiff]

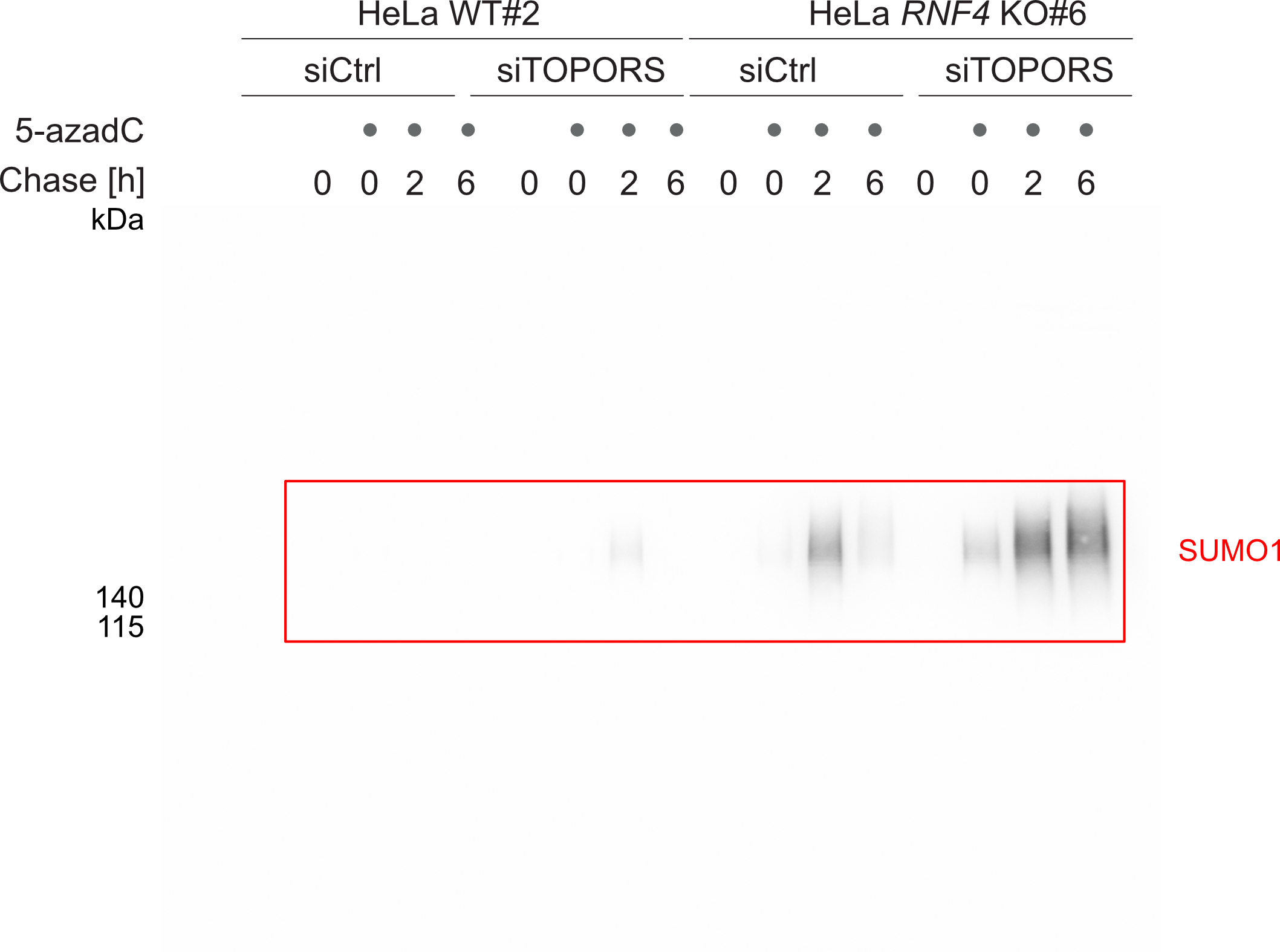

Supplement: Supplementary file 9 — Source data Fig. 6 [file 44318_2024_108_MOESM9_ESM.zip › EMBOJ-2023-115654_Fig6_sourcedata/Figure6G/western SUMO1 PxP.tiff]

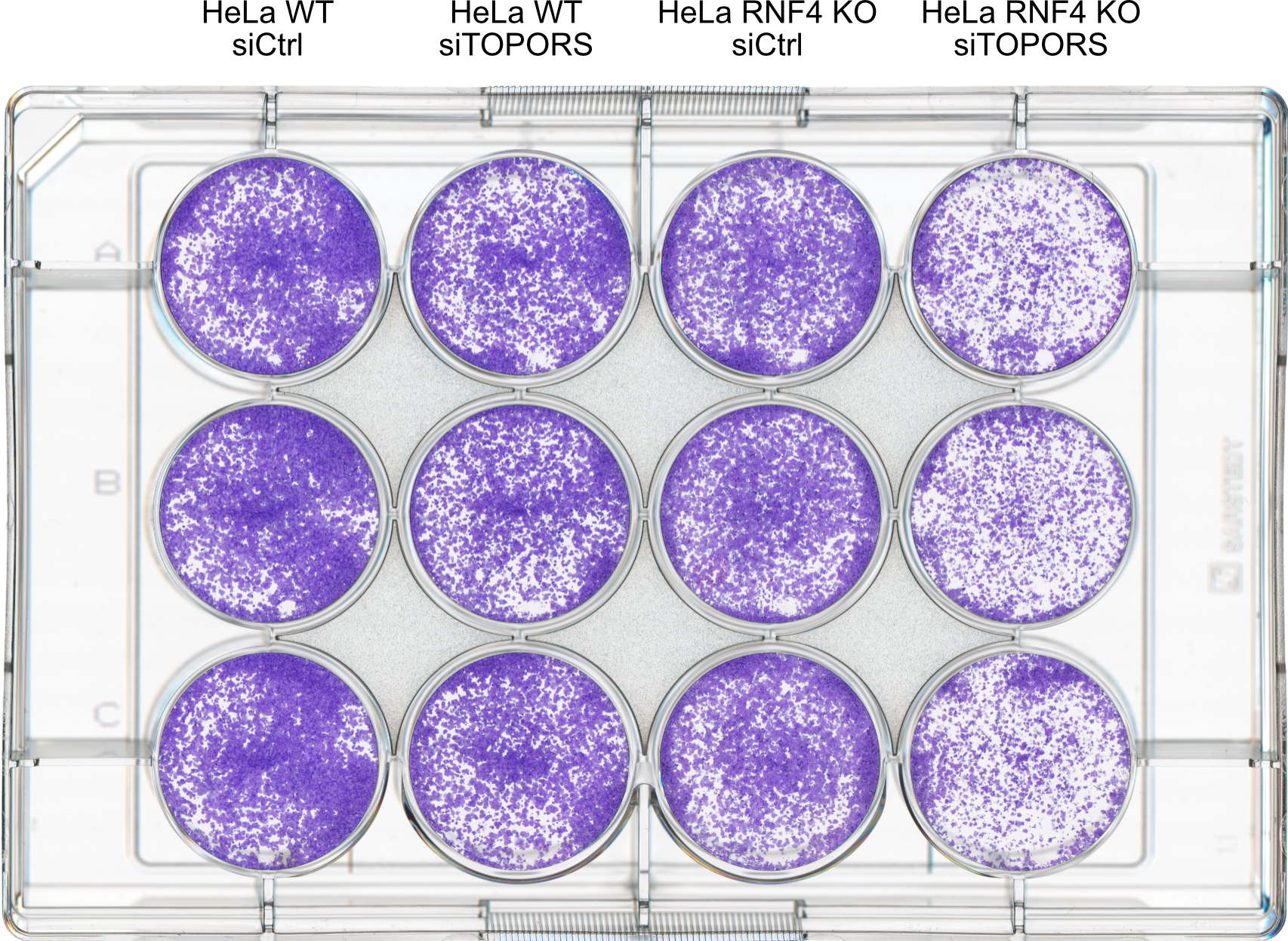

Supplement: Supplementary file 9 — Source data Fig. 6 [file 44318_2024_108_MOESM9_ESM.zip › EMBOJ-2023-115654_Fig6_sourcedata/Figure6D/image colony formation HeLa WT and RNF4 KO.tiff]

Source data: Fig EV1H.

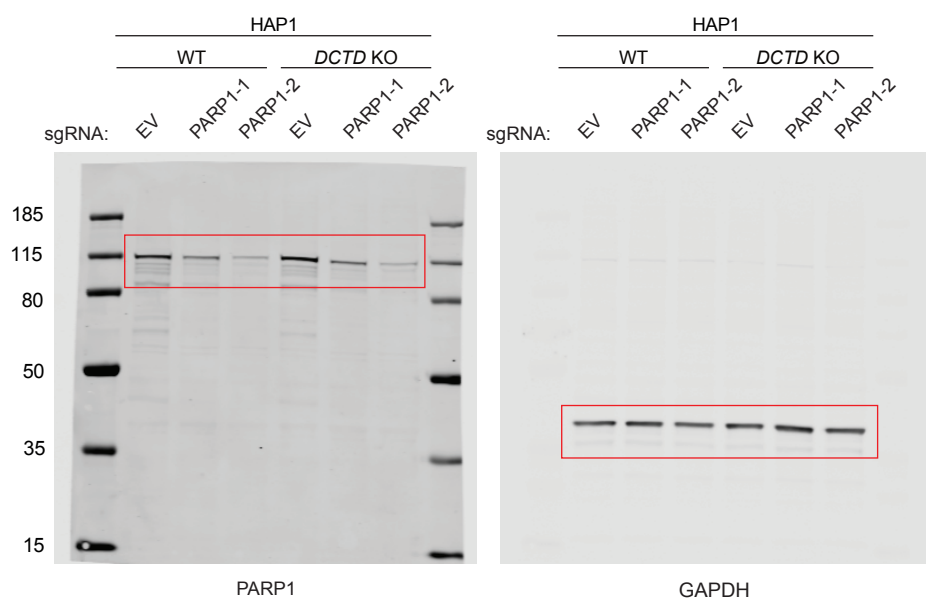

Supplement: Supplementary file 10 — Figure EV1 Source Data [file 44318_2024_108_MOESM10_ESM.zip › EMBOJ-2023-115654_FigEV1_sourcedata/EV1H.pdf]

Source data: Figure EV1D.

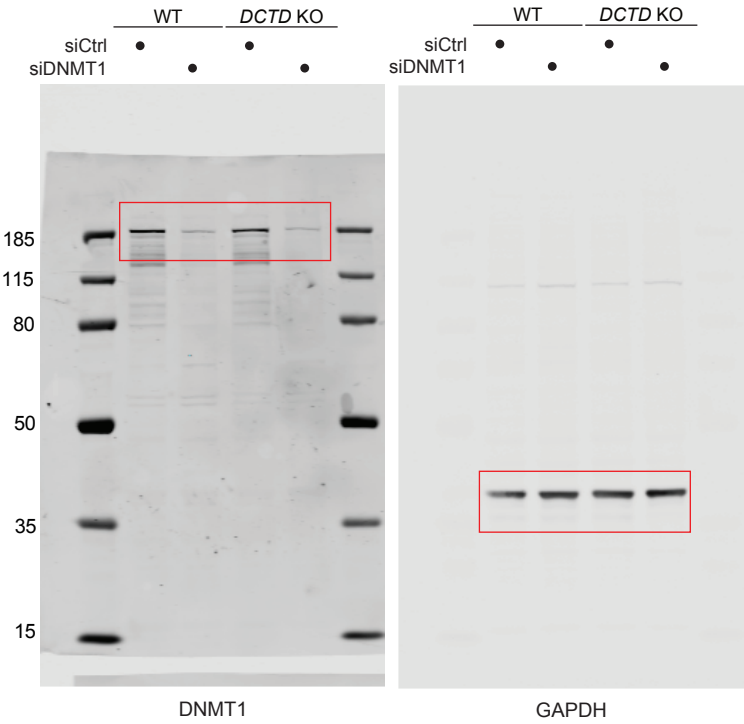

Supplement: Supplementary file 10 — Figure EV1 Source Data [file 44318_2024_108_MOESM10_ESM.zip › EMBOJ-2023-115654_FigEV1_sourcedata/EV1D.pdf]

Source data: Fig EV1A.

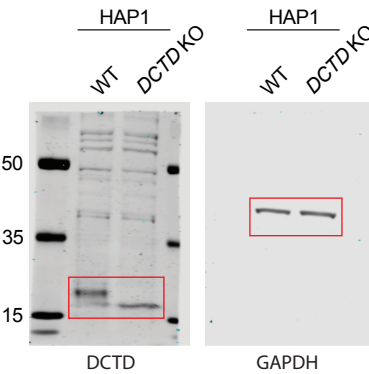

Supplement: Supplementary file 10 — Figure EV1 Source Data [file 44318_2024_108_MOESM10_ESM.zip › EMBOJ-2023-115654_FigEV1_sourcedata/EV1A.pdf]

Source data: Fig EV1B.

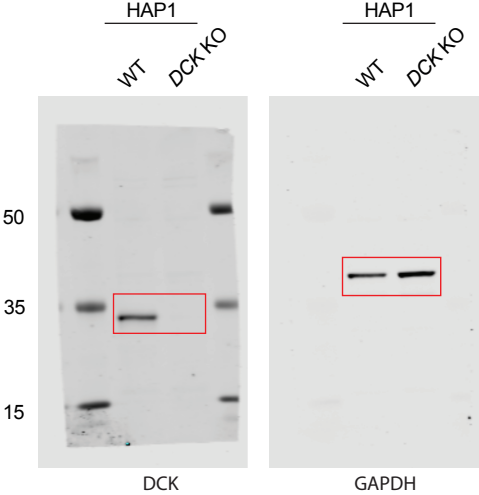

Supplement: Supplementary file 10 — Figure EV1 Source Data [file 44318_2024_108_MOESM10_ESM.zip › EMBOJ-2023-115654_FigEV1_sourcedata/EV1B.pdf]

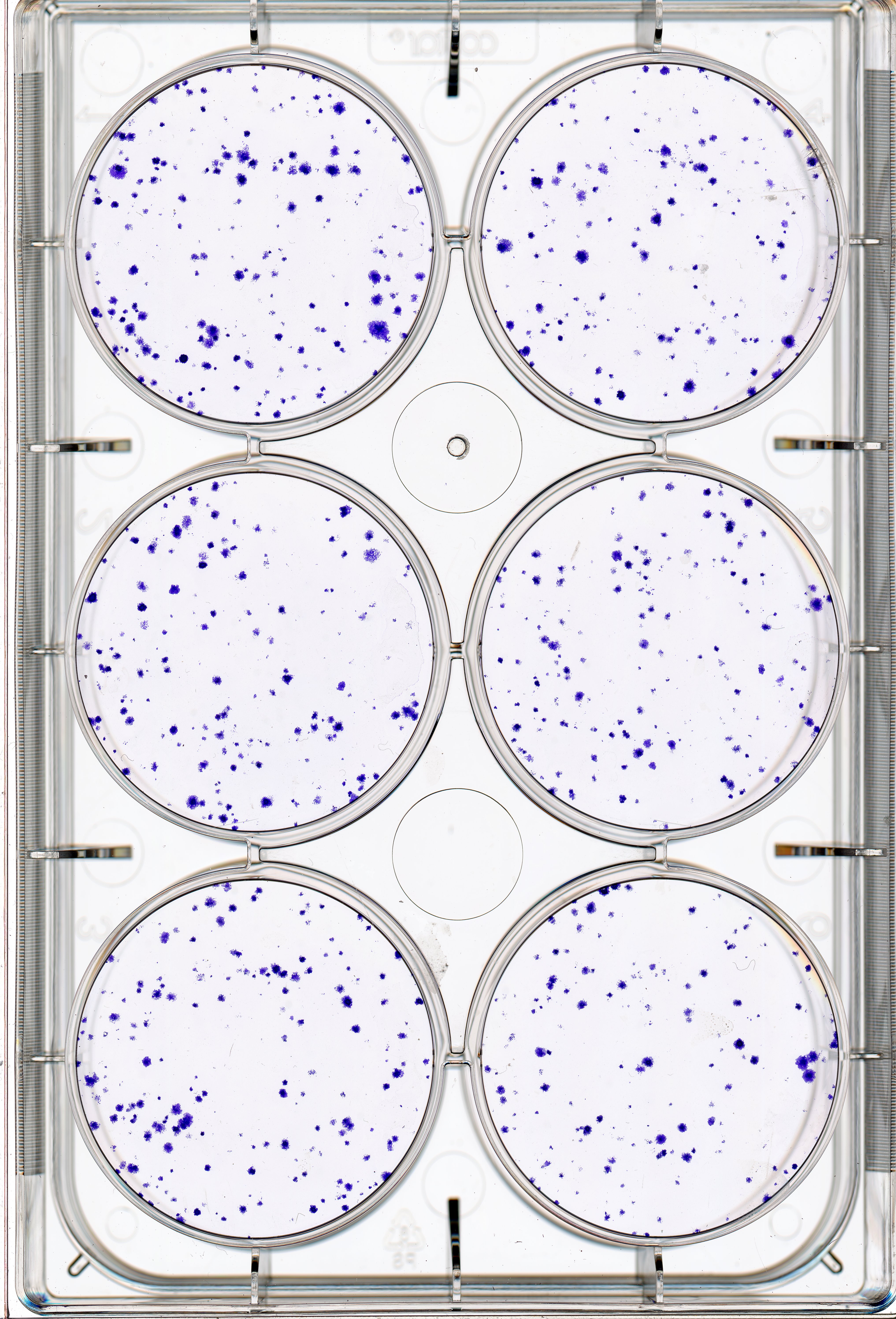

Supplement: Supplementary file 10 — Figure EV1 Source Data [file 44318_2024_108_MOESM10_ESM.zip › EMBOJ-2023-115654_FigEV1_sourcedata/EV1J/E231129 DCTDsgPARP1-2 5dC200-300.jpg]

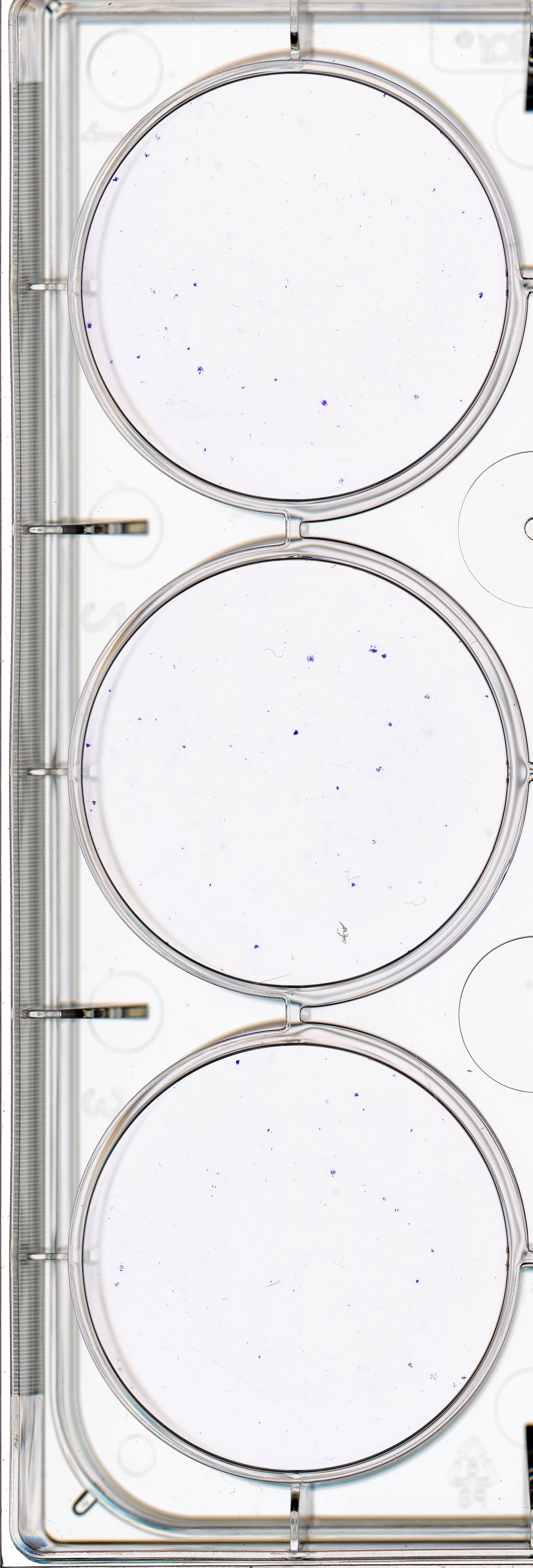

Supplement: Supplementary file 10 — Figure EV1 Source Data [file 44318_2024_108_MOESM10_ESM.zip › EMBOJ-2023-115654_FigEV1_sourcedata/EV1J/E231129 DCTDsgPARP1-1 5dC1200.jpg]

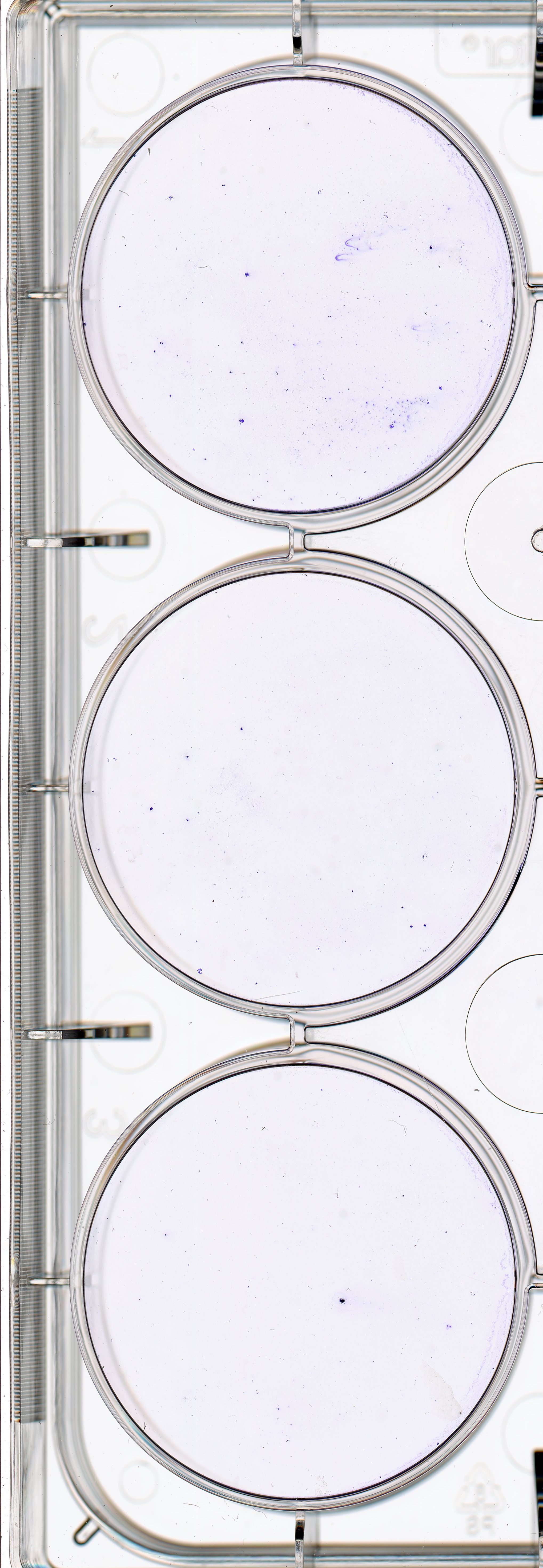

Supplement: Supplementary file 10 — Figure EV1 Source Data [file 44318_2024_108_MOESM10_ESM.zip › EMBOJ-2023-115654_FigEV1_sourcedata/EV1J/E231129 WTsgPARP1-1 5dC600.jpg]

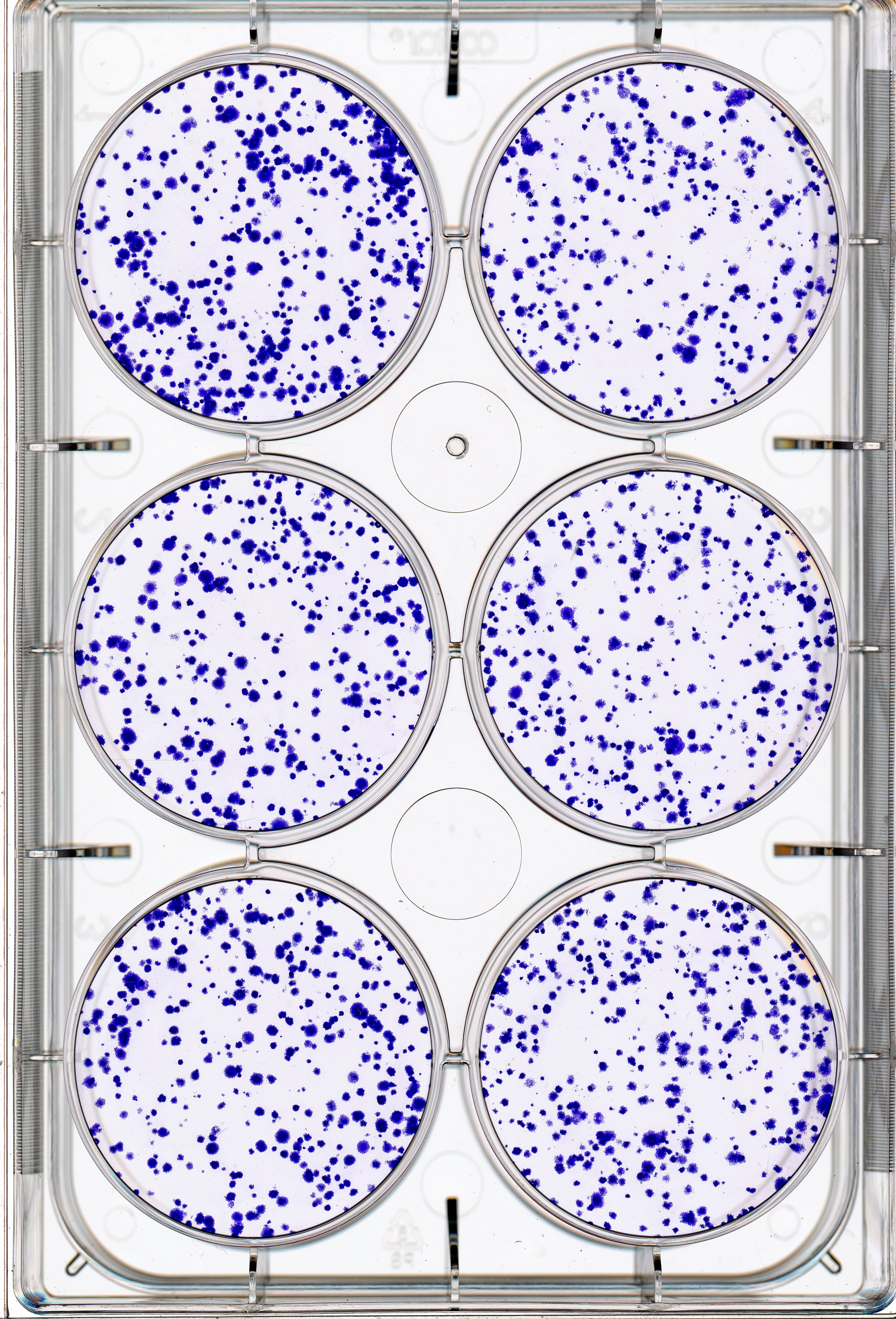

Supplement: Supplementary file 10 — Figure EV1 Source Data [file 44318_2024_108_MOESM10_ESM.zip › EMBOJ-2023-115654_FigEV1_sourcedata/EV1J/E231129 DCTDsgEV 5dC0-100.jpg]

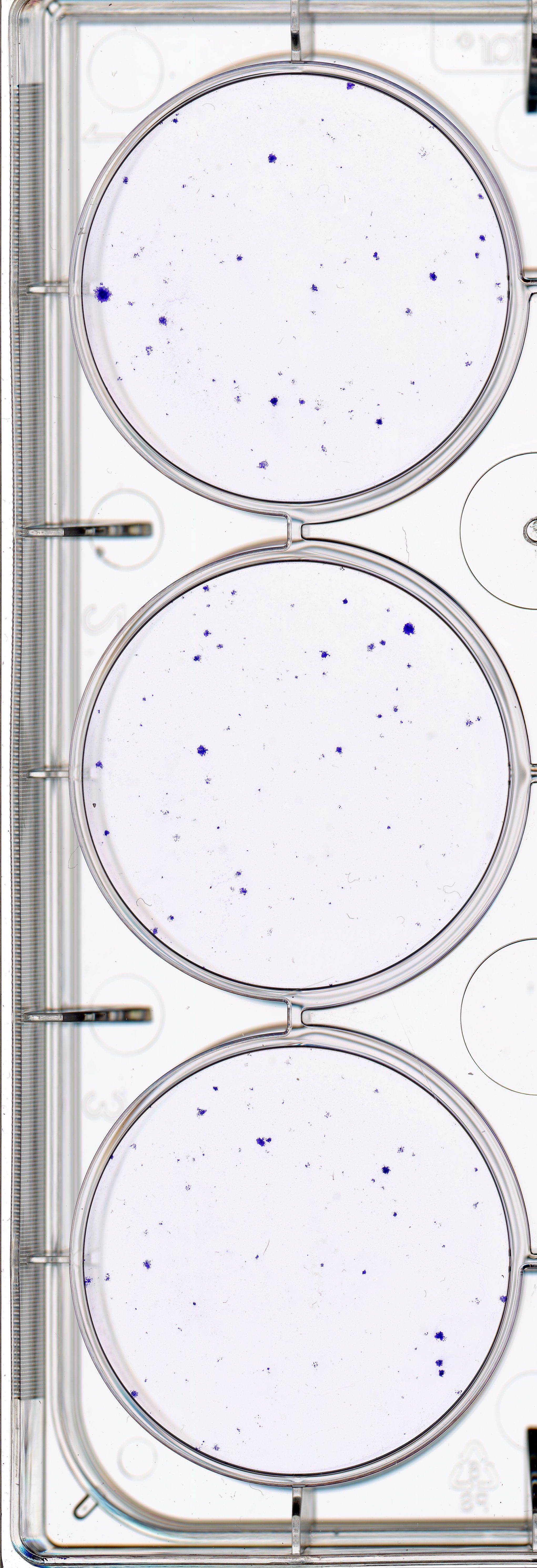

Supplement: Supplementary file 10 — Figure EV1 Source Data [file 44318_2024_108_MOESM10_ESM.zip › EMBOJ-2023-115654_FigEV1_sourcedata/EV1J/E231129 DCTDsgPARP1-2 5dC600.jpg]

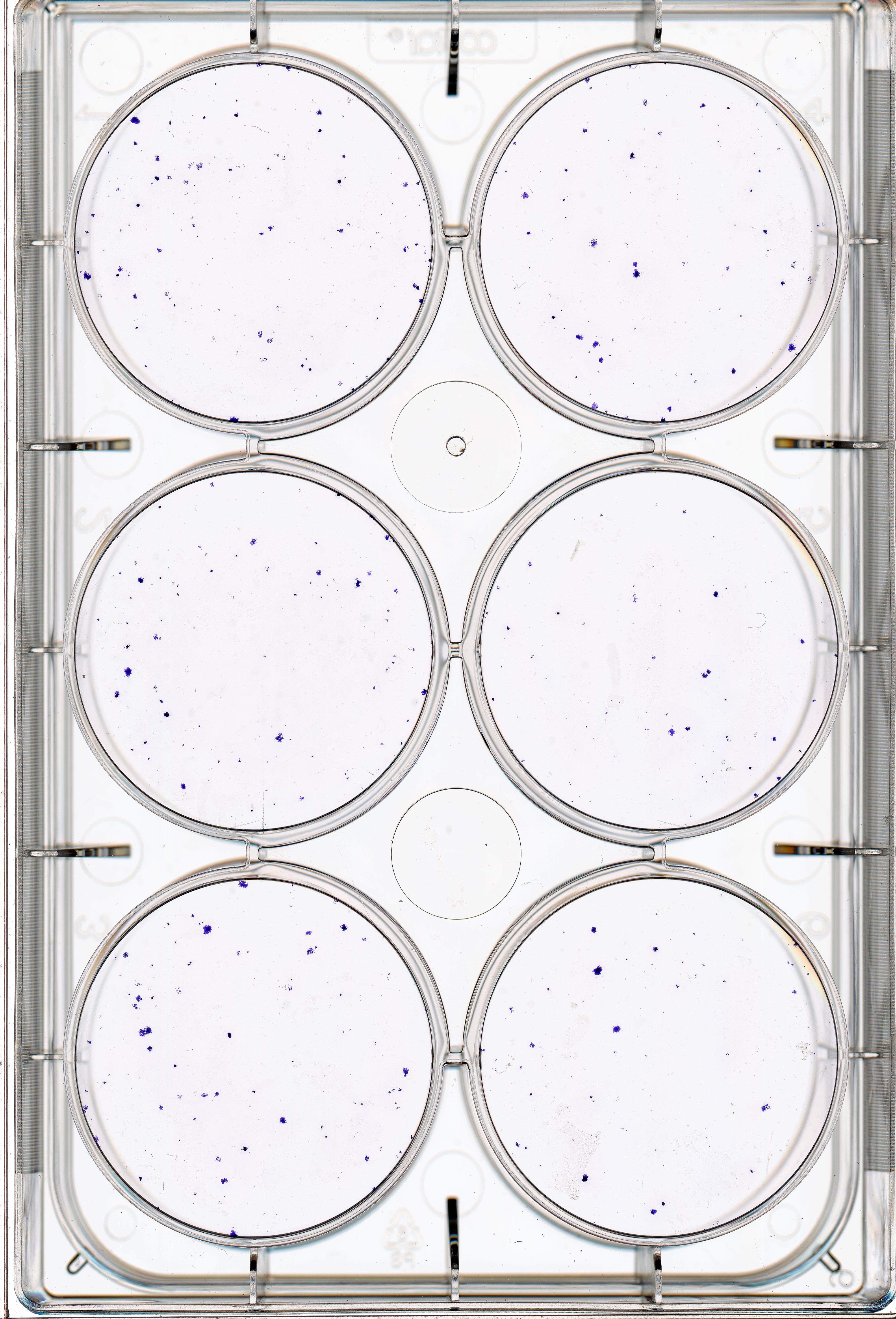

Supplement: Supplementary file 10 — Figure EV1 Source Data [file 44318_2024_108_MOESM10_ESM.zip › EMBOJ-2023-115654_FigEV1_sourcedata/EV1J/E231129 WTsgPARP1-2 5dC200-300.jpg]

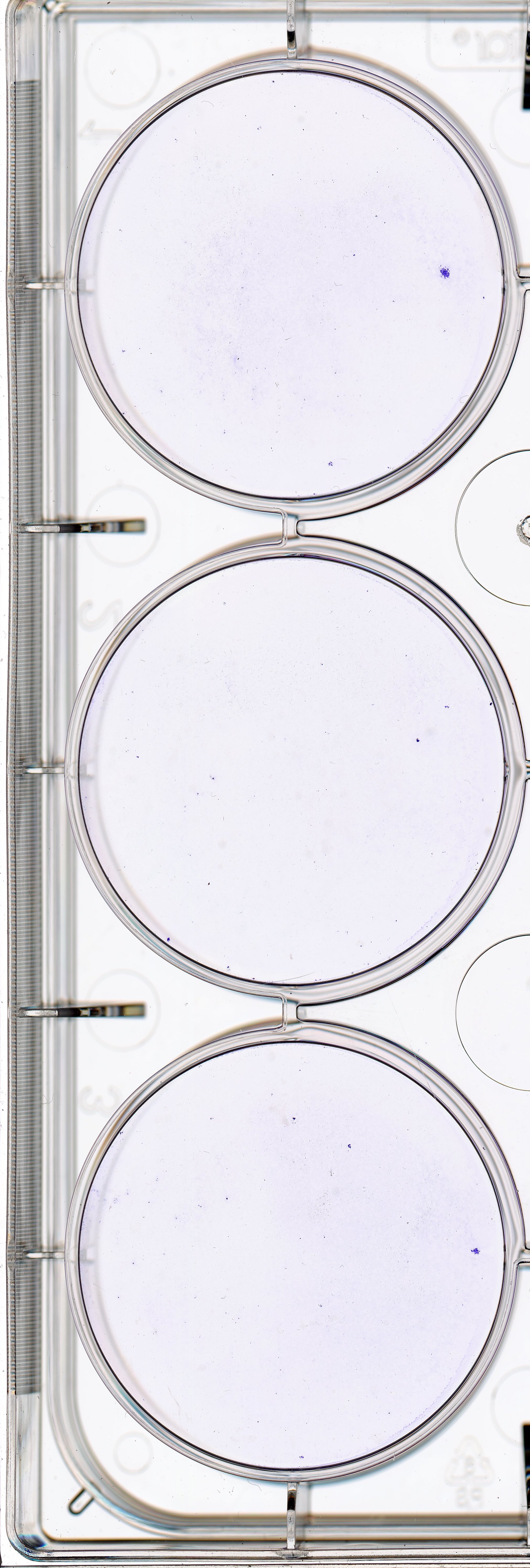

Supplement: Supplementary file 10 — Figure EV1 Source Data [file 44318_2024_108_MOESM10_ESM.zip › EMBOJ-2023-115654_FigEV1_sourcedata/EV1J/E231129 WTsgEV 5dC1200.jpg]

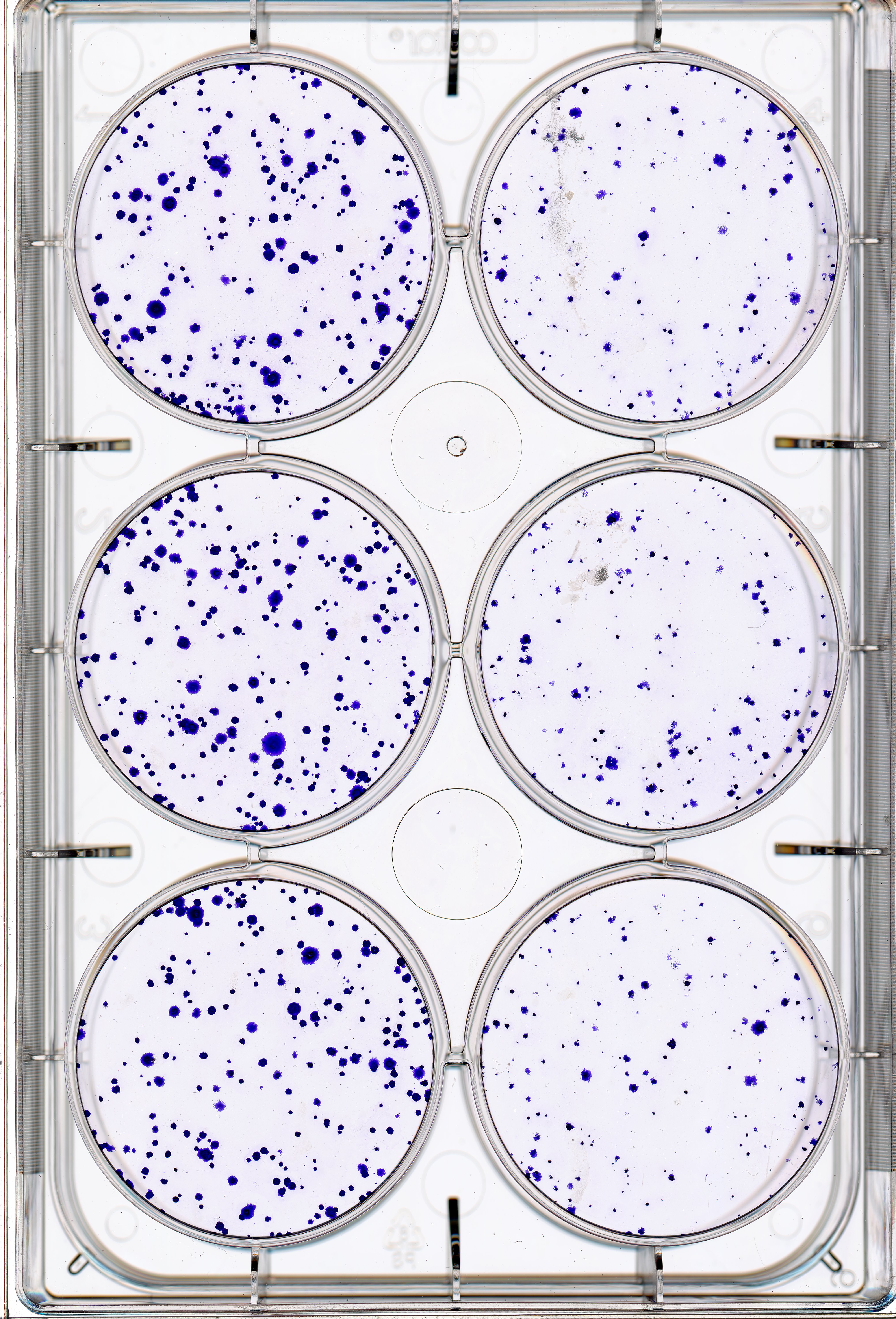

Supplement: Supplementary file 10 — Figure EV1 Source Data [file 44318_2024_108_MOESM10_ESM.zip › EMBOJ-2023-115654_FigEV1_sourcedata/EV1J/E231129 WTsgPARP1-1 5dC0-100.jpg]

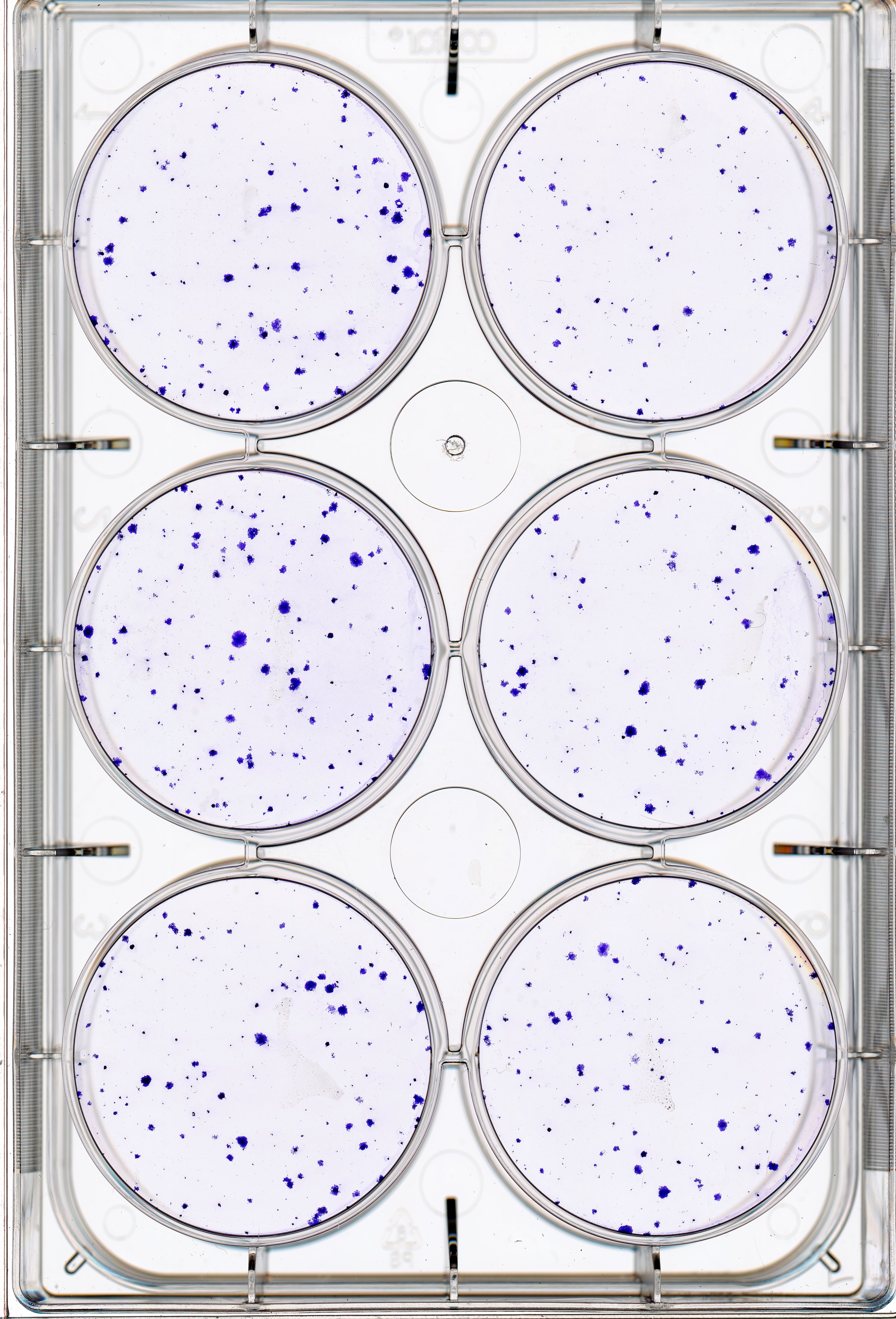

Supplement: Supplementary file 10 — Figure EV1 Source Data [file 44318_2024_108_MOESM10_ESM.zip › EMBOJ-2023-115654_FigEV1_sourcedata/EV1J/E231129 WTsgEV 5dC200-300.jpg]

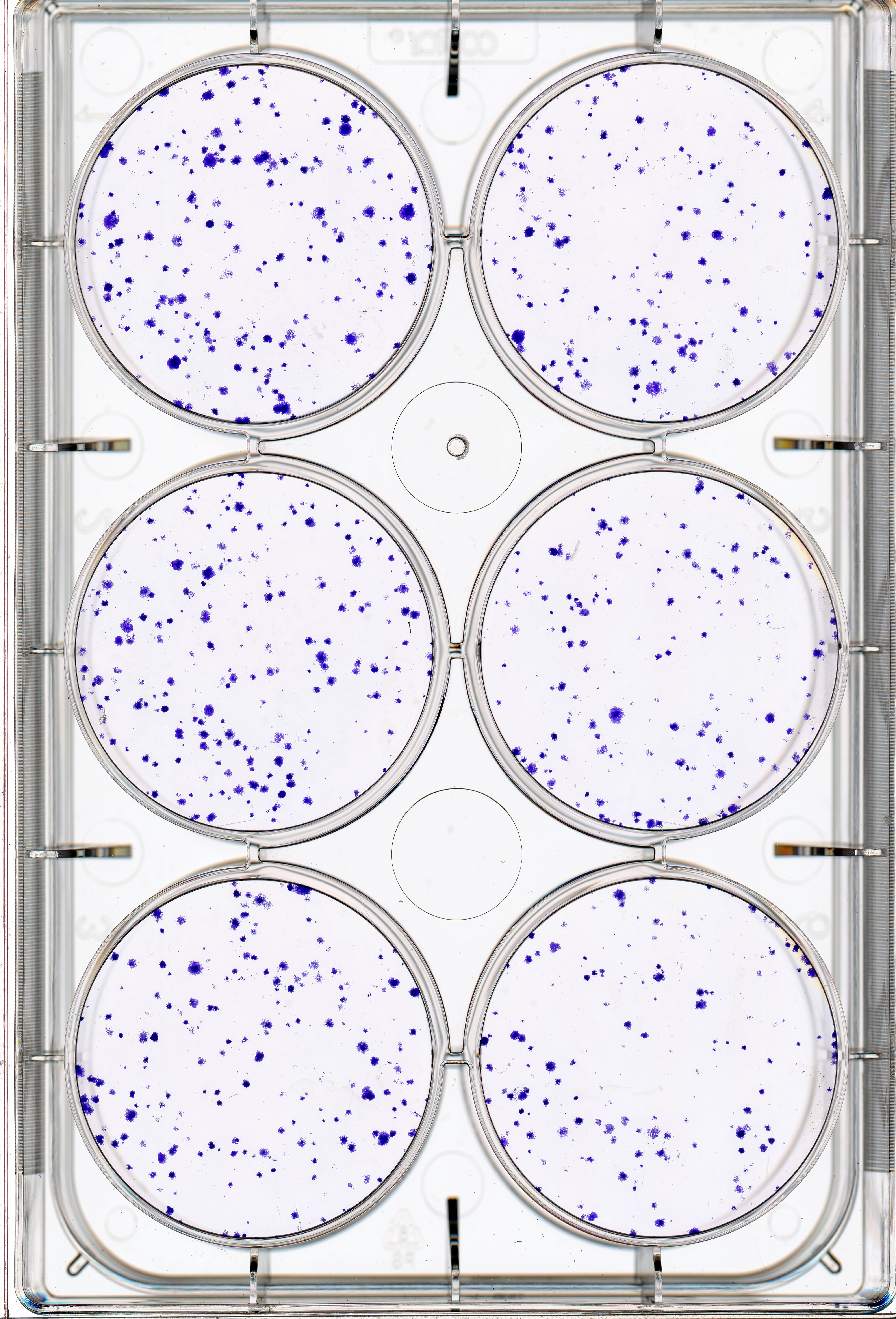

Supplement: Supplementary file 10 — Figure EV1 Source Data [file 44318_2024_108_MOESM10_ESM.zip › EMBOJ-2023-115654_FigEV1_sourcedata/EV1J/E231129 DCTDsgPARP1-1 5dC200-300.jpg]

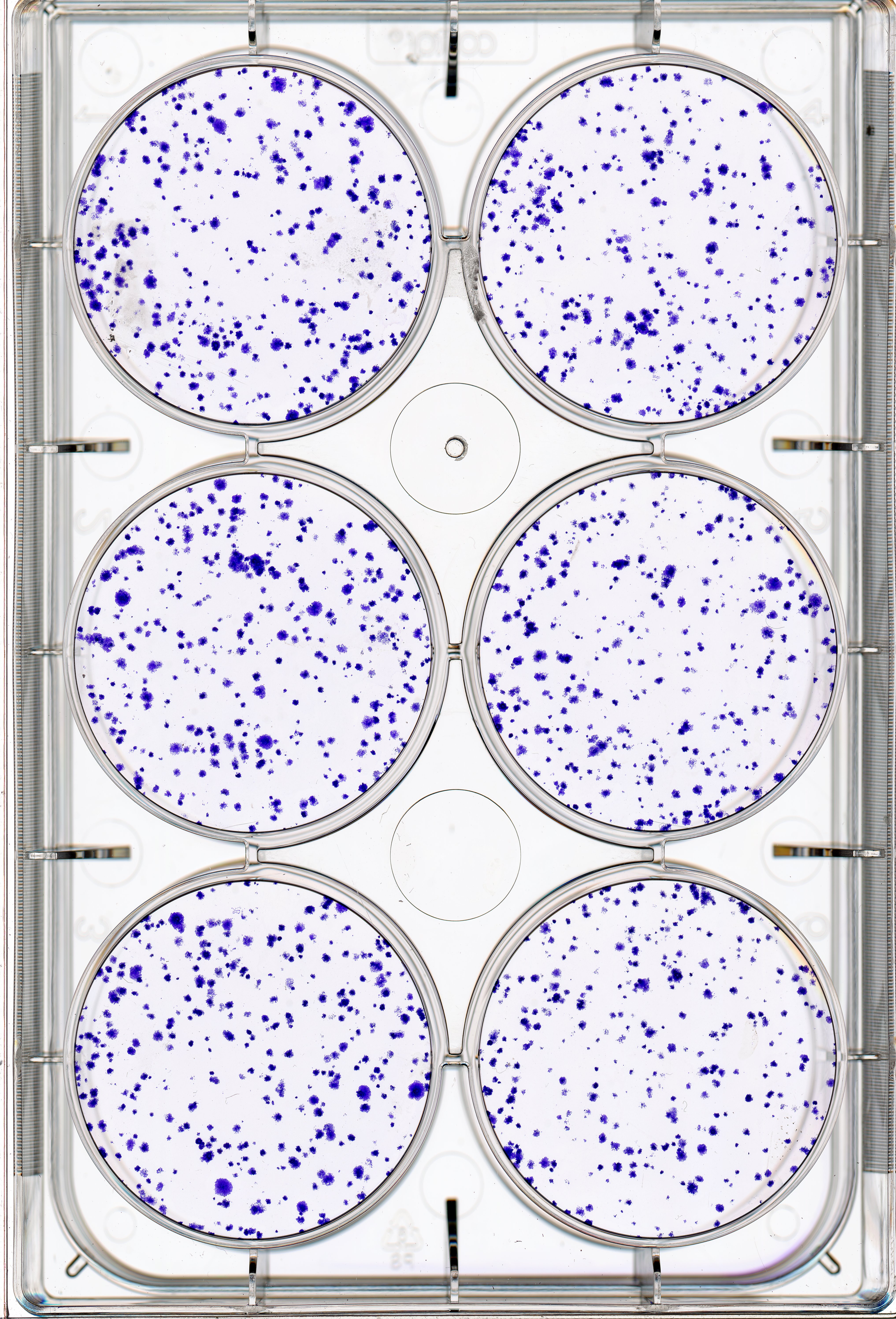

Supplement: Supplementary file 10 — Figure EV1 Source Data [file 44318_2024_108_MOESM10_ESM.zip › EMBOJ-2023-115654_FigEV1_sourcedata/EV1J/E231129 DCTDsgEV 5dC200-300.jpg]

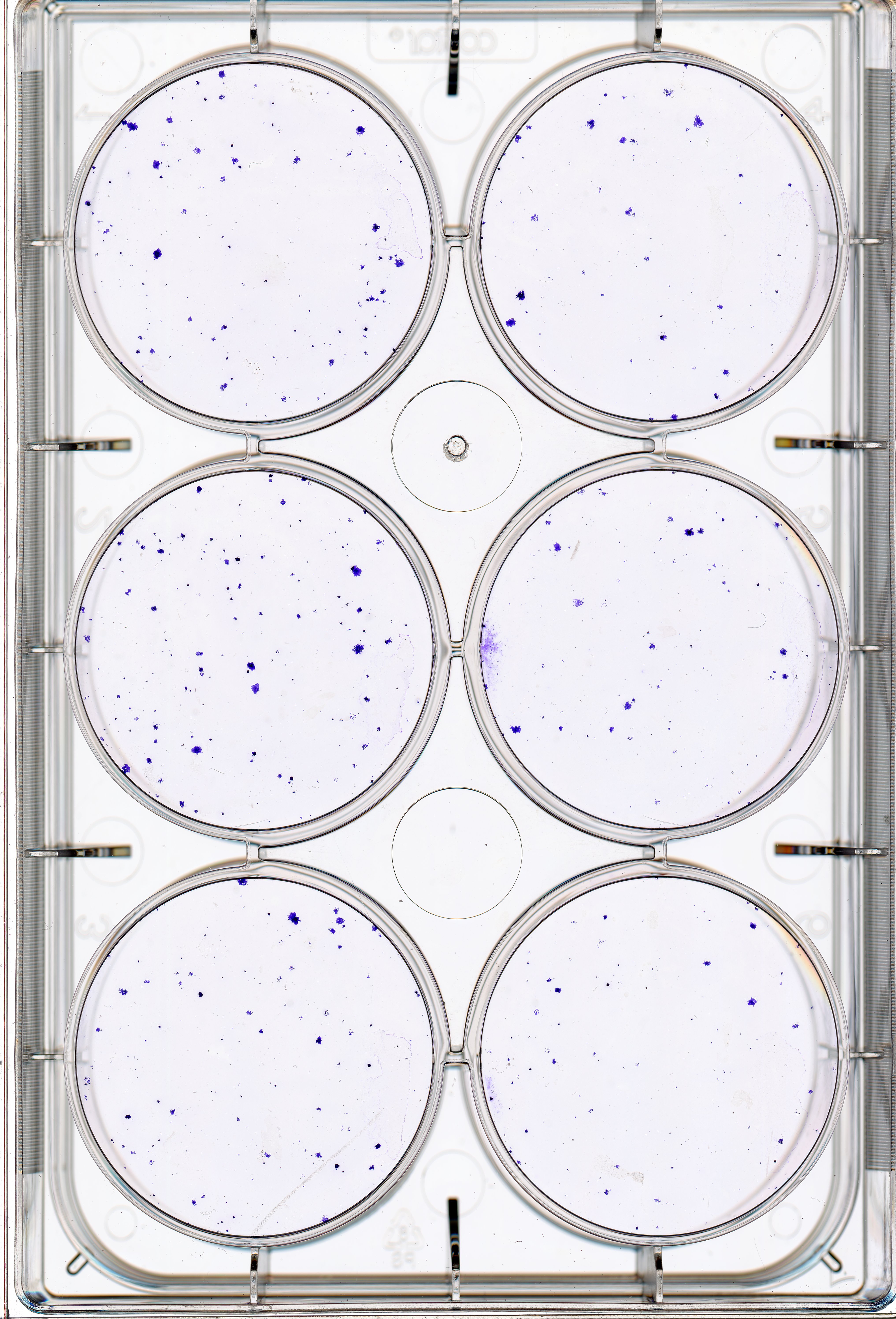

Supplement: Supplementary file 10 — Figure EV1 Source Data [file 44318_2024_108_MOESM10_ESM.zip › EMBOJ-2023-115654_FigEV1_sourcedata/EV1J/E231129 WTsgPARP1-1 5dC200-300.jpg]

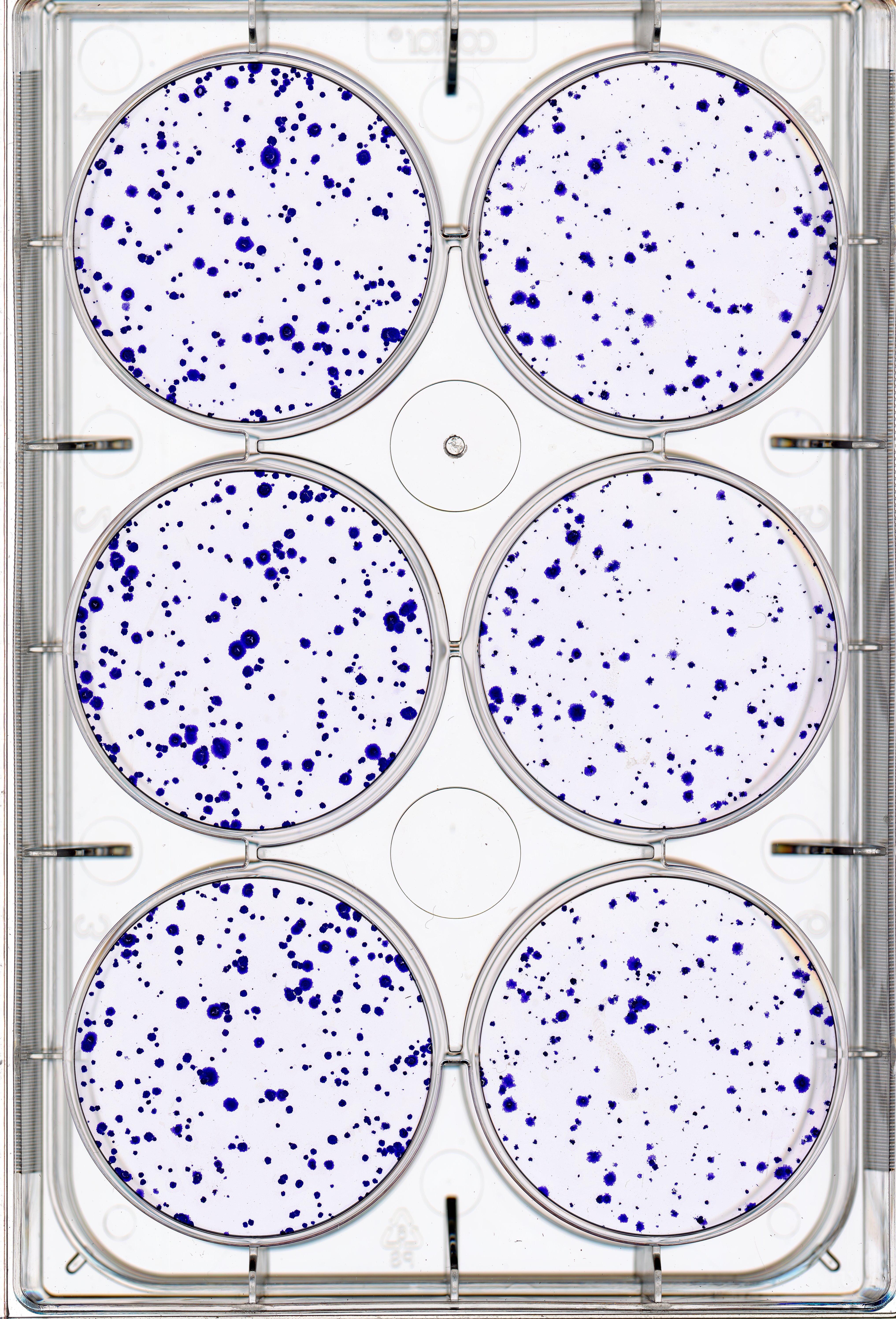

Supplement: Supplementary file 10 — Figure EV1 Source Data [file 44318_2024_108_MOESM10_ESM.zip › EMBOJ-2023-115654_FigEV1_sourcedata/EV1J/E231129 WTsgEV 5dC0-100.jpg]

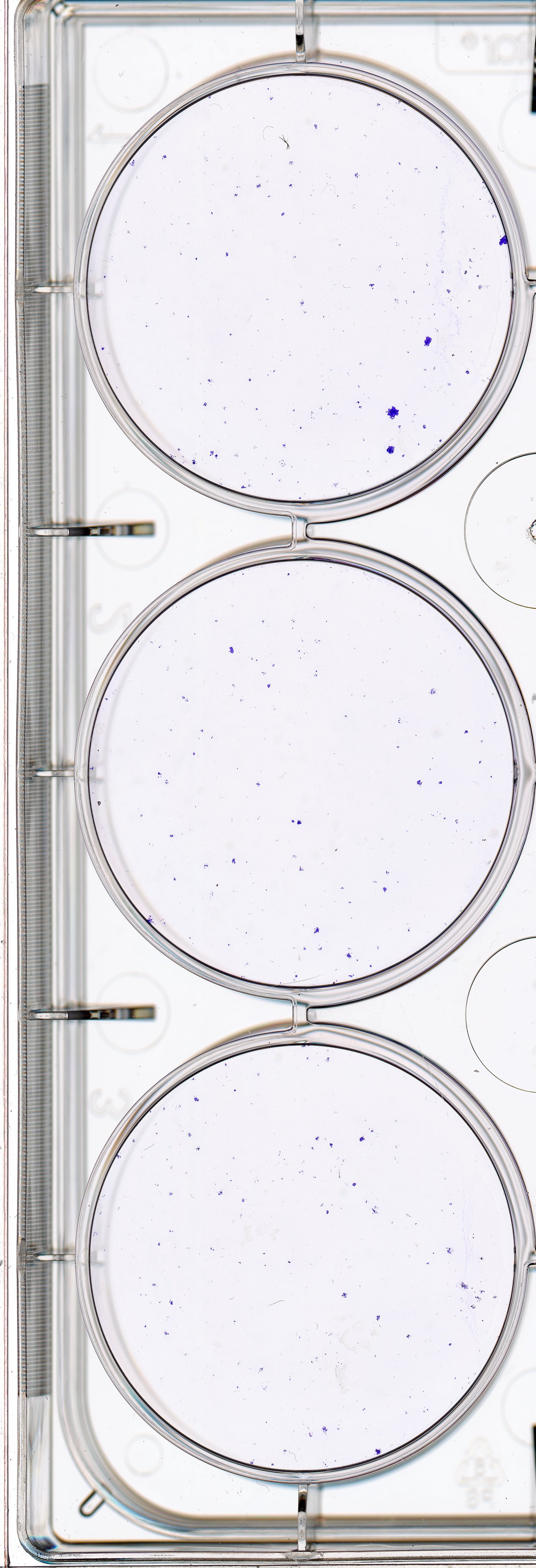

Supplement: Supplementary file 10 — Figure EV1 Source Data [file 44318_2024_108_MOESM10_ESM.zip › EMBOJ-2023-115654_FigEV1_sourcedata/EV1J/E231129 DCTDsgEV 5dC1200.jpg]

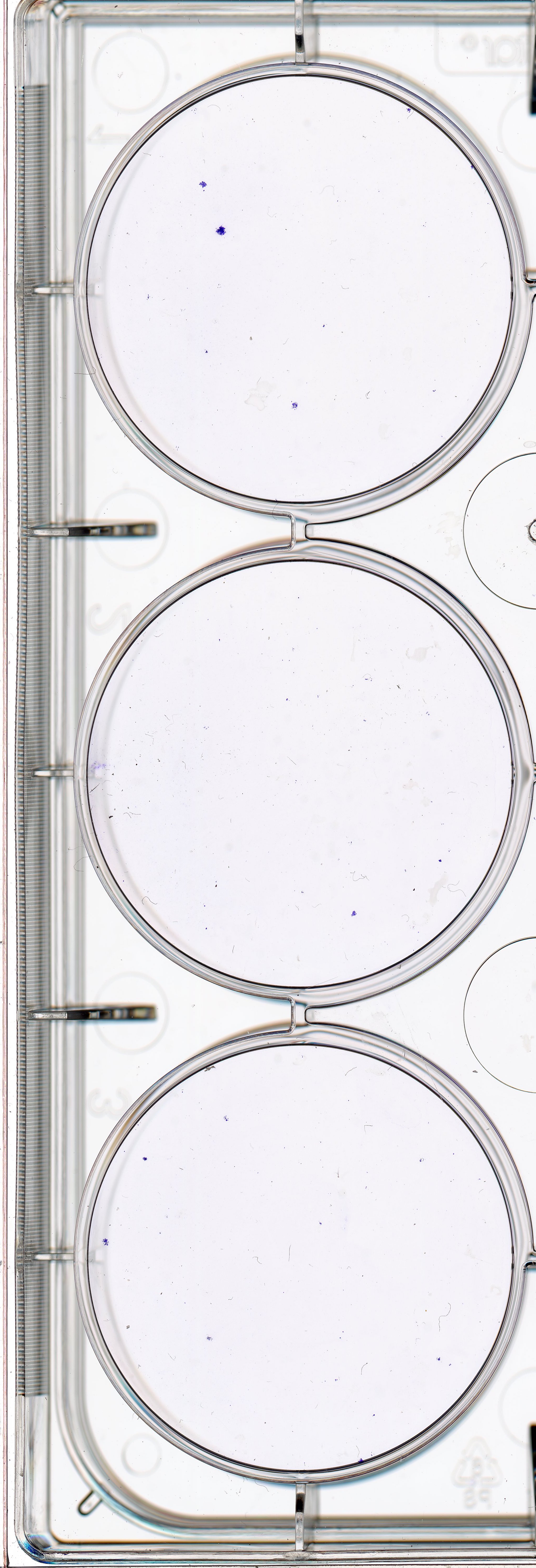

Supplement: Supplementary file 10 — Figure EV1 Source Data [file 44318_2024_108_MOESM10_ESM.zip › EMBOJ-2023-115654_FigEV1_sourcedata/EV1J/E231129 DCTDsgPARP1-2 5dC1200.jpg]

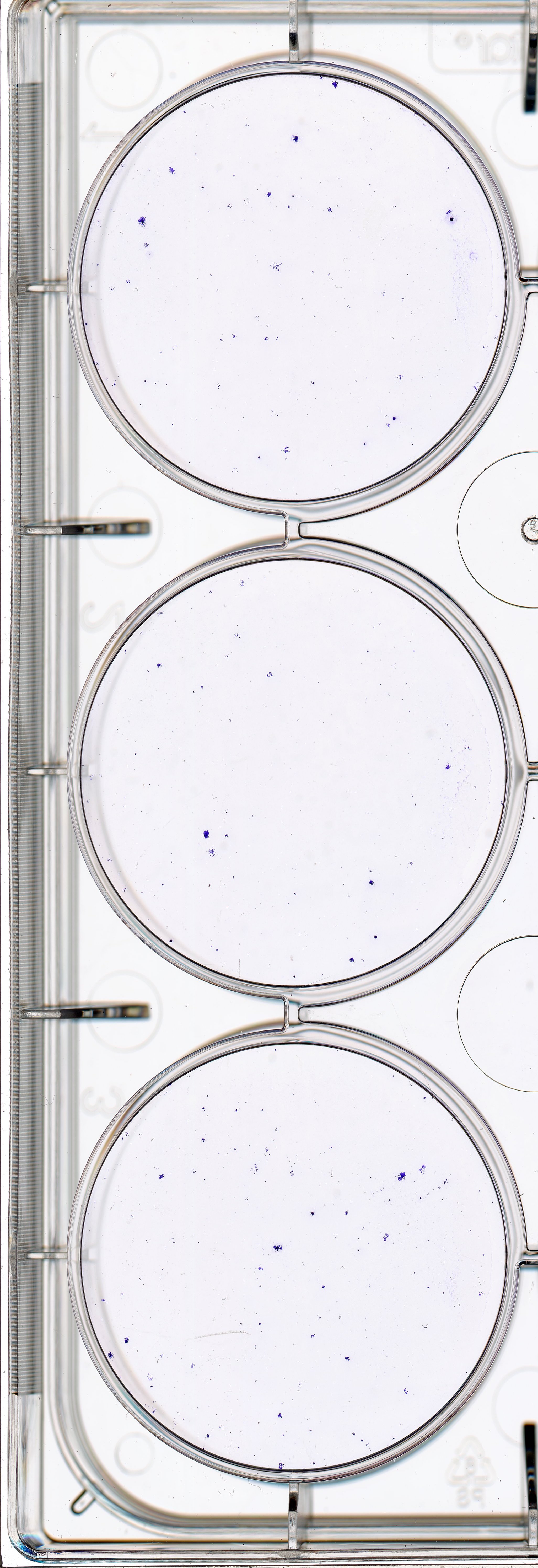

Supplement: Supplementary file 10 — Figure EV1 Source Data [file 44318_2024_108_MOESM10_ESM.zip › EMBOJ-2023-115654_FigEV1_sourcedata/EV1J/E231129 WTsgEV 5dC600.jpg]

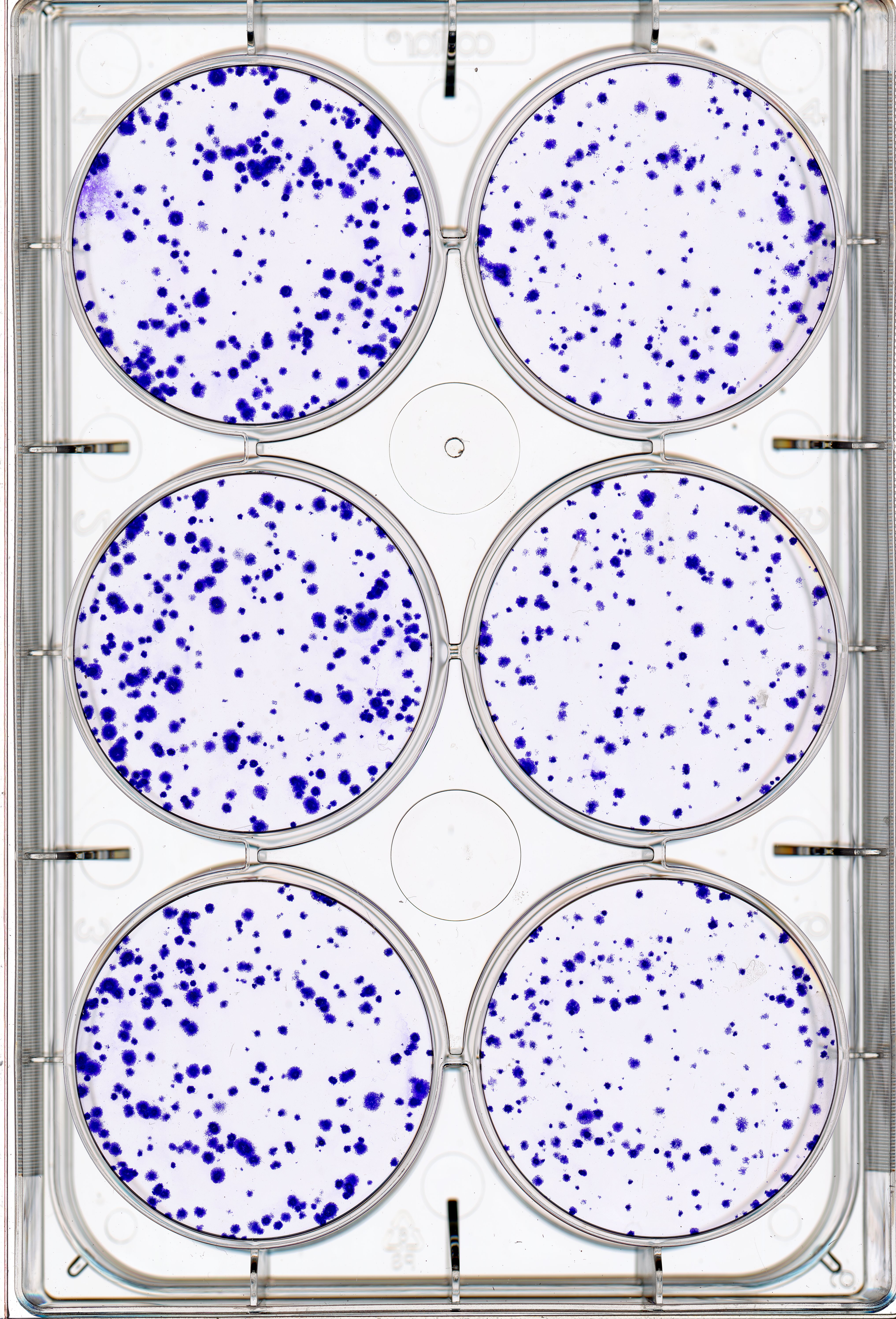

Supplement: Supplementary file 10 — Figure EV1 Source Data [file 44318_2024_108_MOESM10_ESM.zip › EMBOJ-2023-115654_FigEV1_sourcedata/EV1J/E231129 DCTDsgPARP1-2 5dC0-100.jpg]

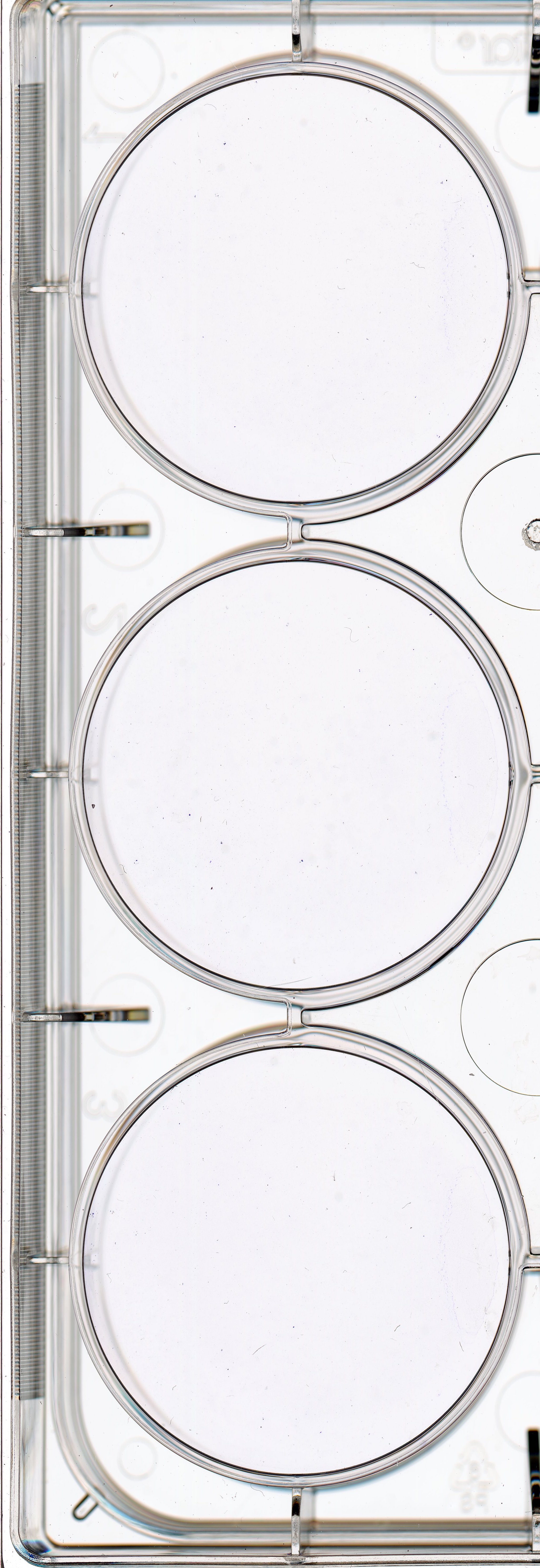

Supplement: Supplementary file 10 — Figure EV1 Source Data [file 44318_2024_108_MOESM10_ESM.zip › EMBOJ-2023-115654_FigEV1_sourcedata/EV1J/E231129 WTsgPARP1-2 5dC1200.jpg]

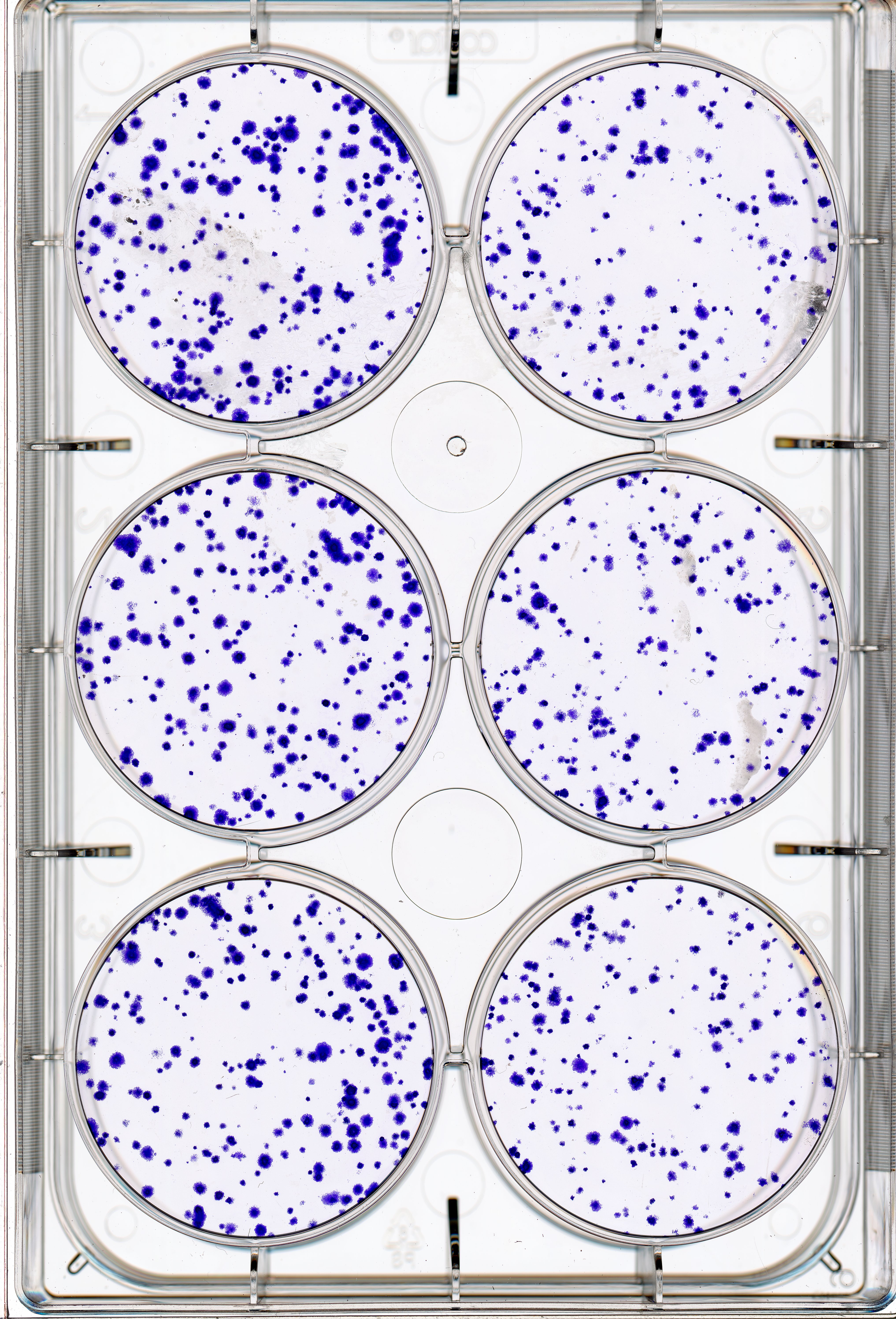

Supplement: Supplementary file 10 — Figure EV1 Source Data [file 44318_2024_108_MOESM10_ESM.zip › EMBOJ-2023-115654_FigEV1_sourcedata/EV1J/E231129 DCTDsgPARP1-1 5dC0-100.jpg]

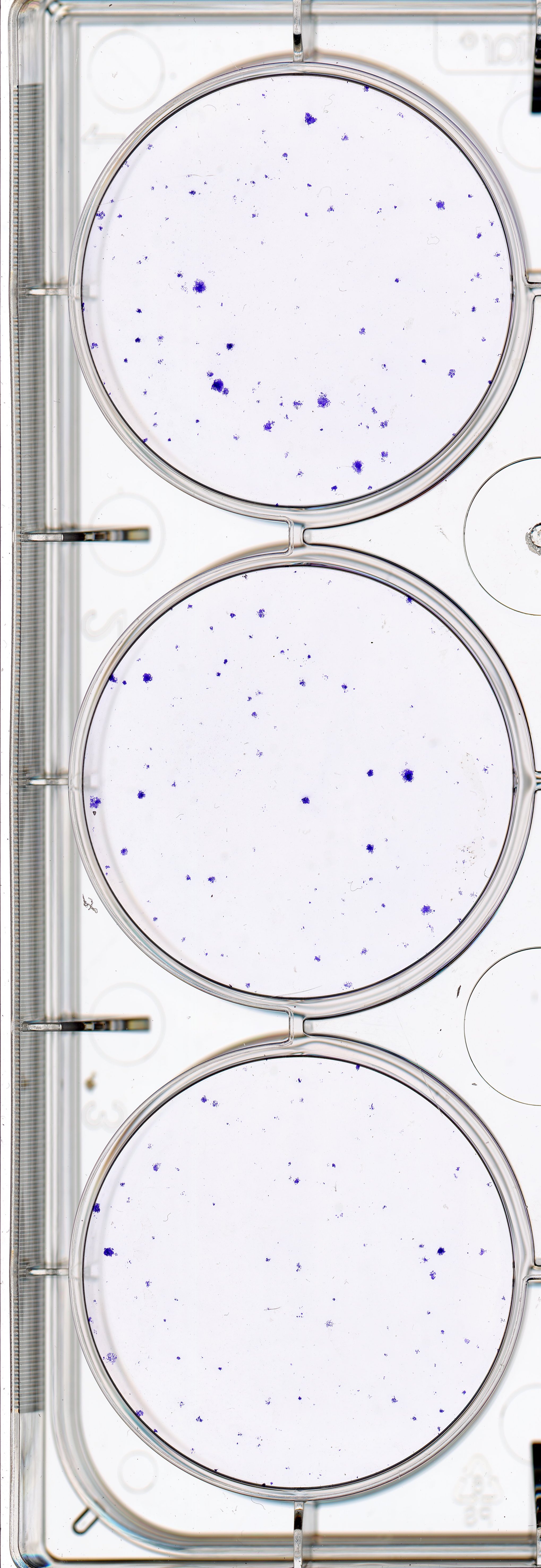

Supplement: Supplementary file 10 — Figure EV1 Source Data [file 44318_2024_108_MOESM10_ESM.zip › EMBOJ-2023-115654_FigEV1_sourcedata/EV1J/E231129 DCTDsgPARP1-1 5dC600.jpg]

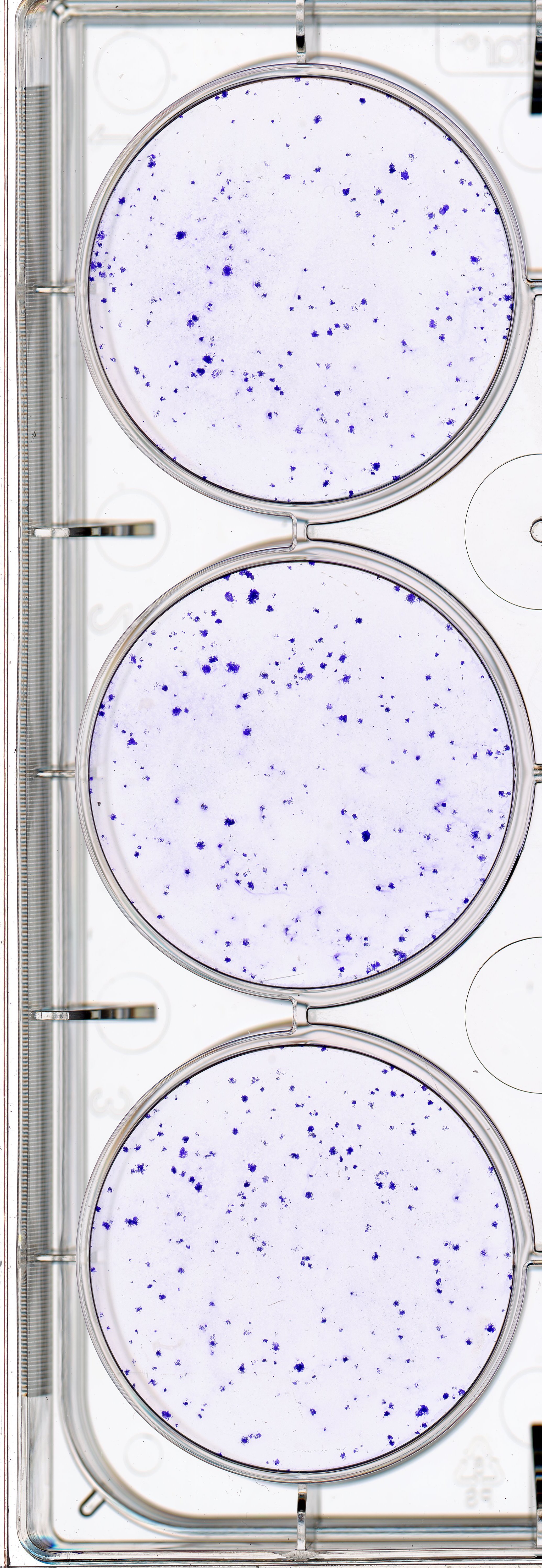

Supplement: Supplementary file 10 — Figure EV1 Source Data [file 44318_2024_108_MOESM10_ESM.zip › EMBOJ-2023-115654_FigEV1_sourcedata/EV1J/E231129 DCTDsgEV 5dC600.jpg]

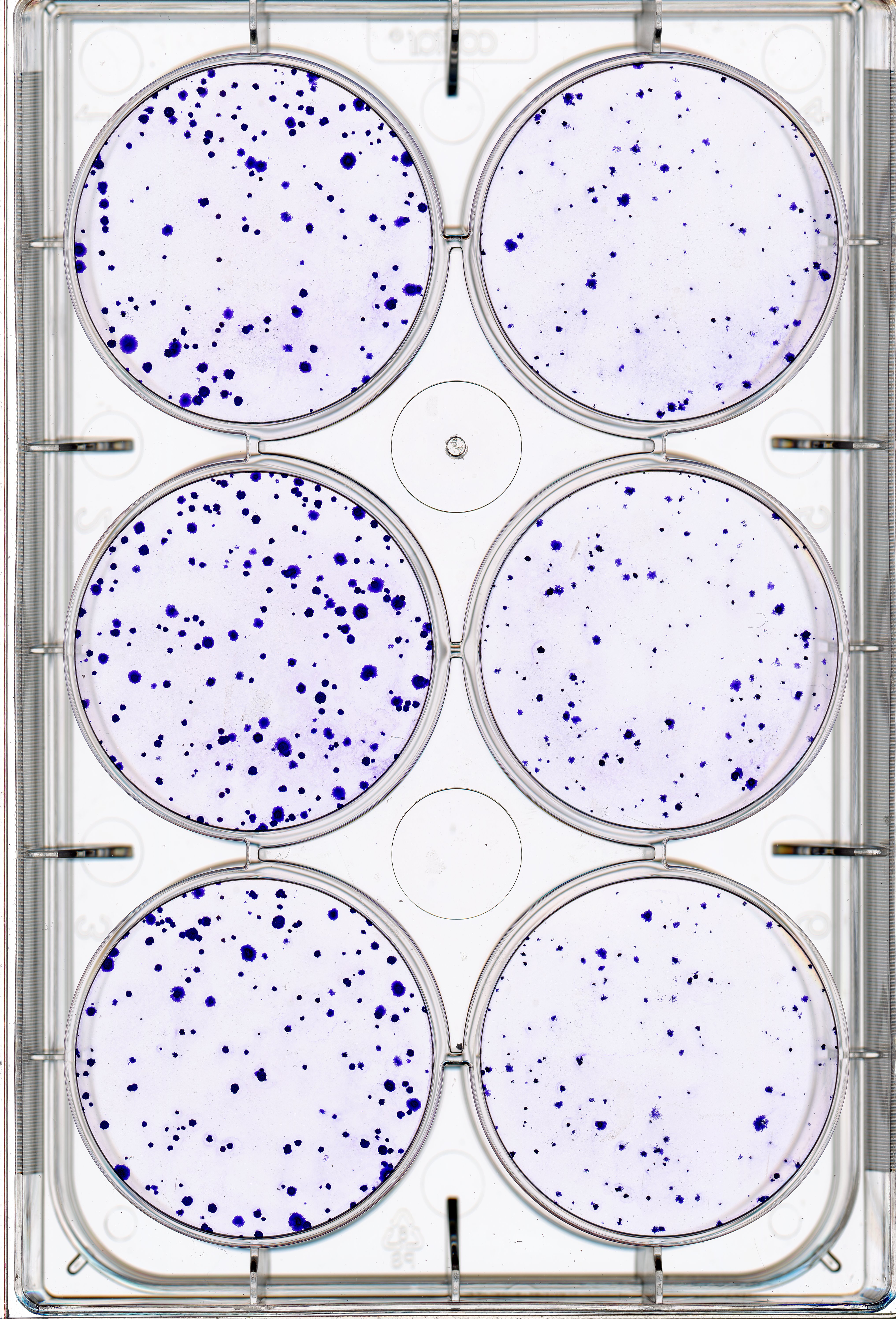

Supplement: Supplementary file 10 — Figure EV1 Source Data [file 44318_2024_108_MOESM10_ESM.zip › EMBOJ-2023-115654_FigEV1_sourcedata/EV1J/E231129 WTsgPARP1-2 5dC0-100.jpg]

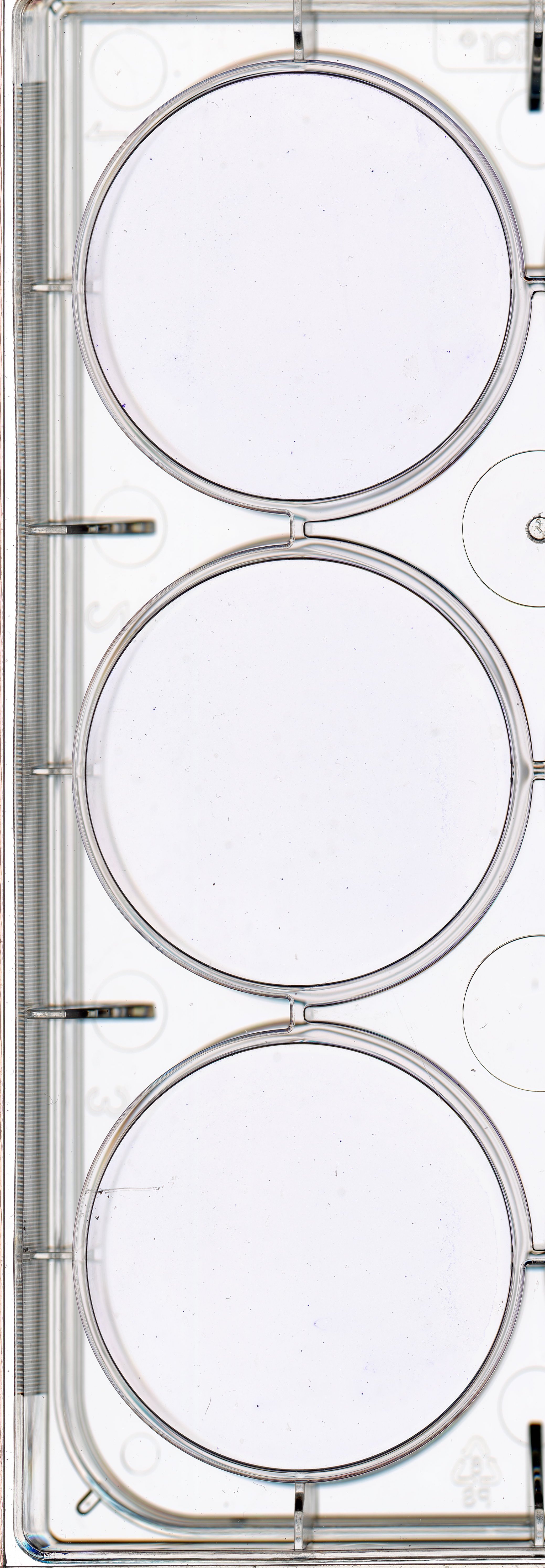

Supplement: Supplementary file 10 — Figure EV1 Source Data [file 44318_2024_108_MOESM10_ESM.zip › EMBOJ-2023-115654_FigEV1_sourcedata/EV1J/E231129 WTsgPARP1-1 5dC1200.jpg]

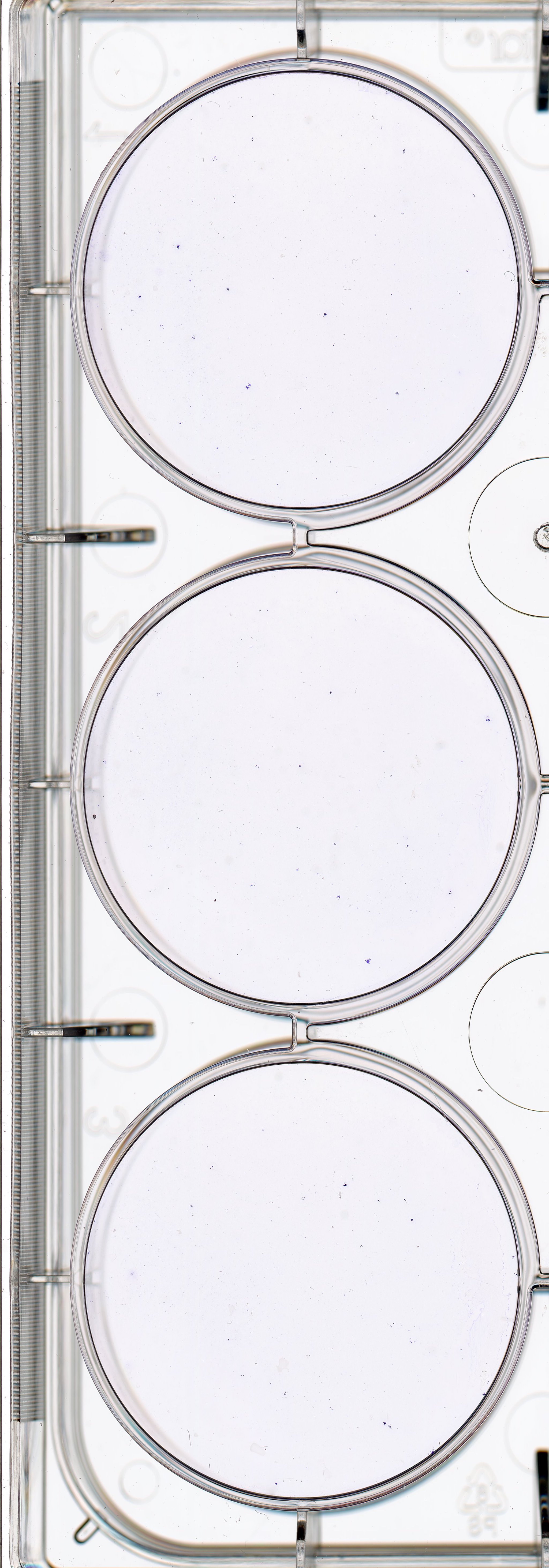

Supplement: Supplementary file 10 — Figure EV1 Source Data [file 44318_2024_108_MOESM10_ESM.zip › EMBOJ-2023-115654_FigEV1_sourcedata/EV1J/E231129 WTsgPARP1-2 5dC600.jpg]

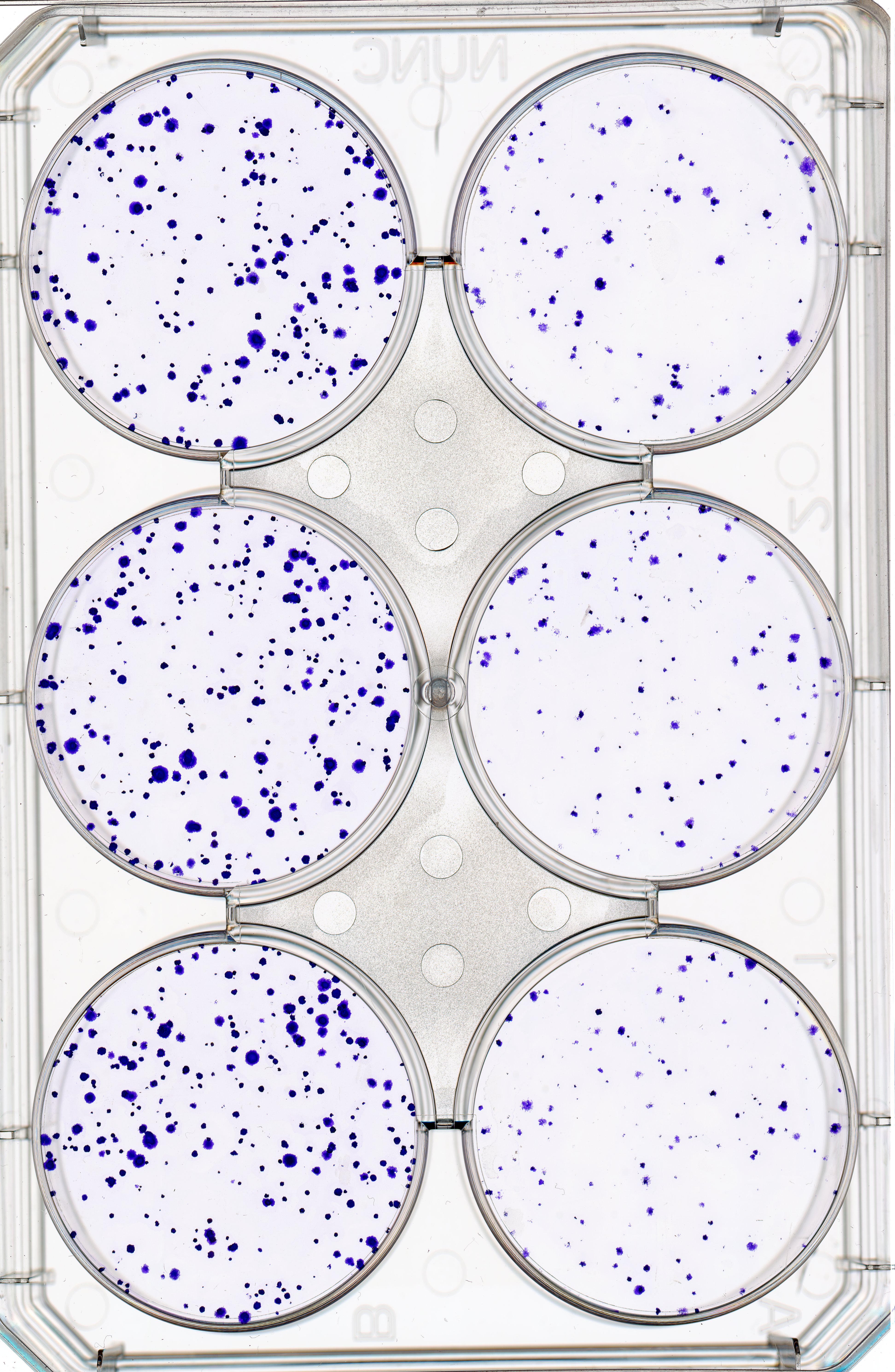

Supplement: Supplementary file 10 — Figure EV1 Source Data [file 44318_2024_108_MOESM10_ESM.zip › EMBOJ-2023-115654_FigEV1_sourcedata/EV1F/E231201 WTsiDNMT1 5dC0-200.jpg]

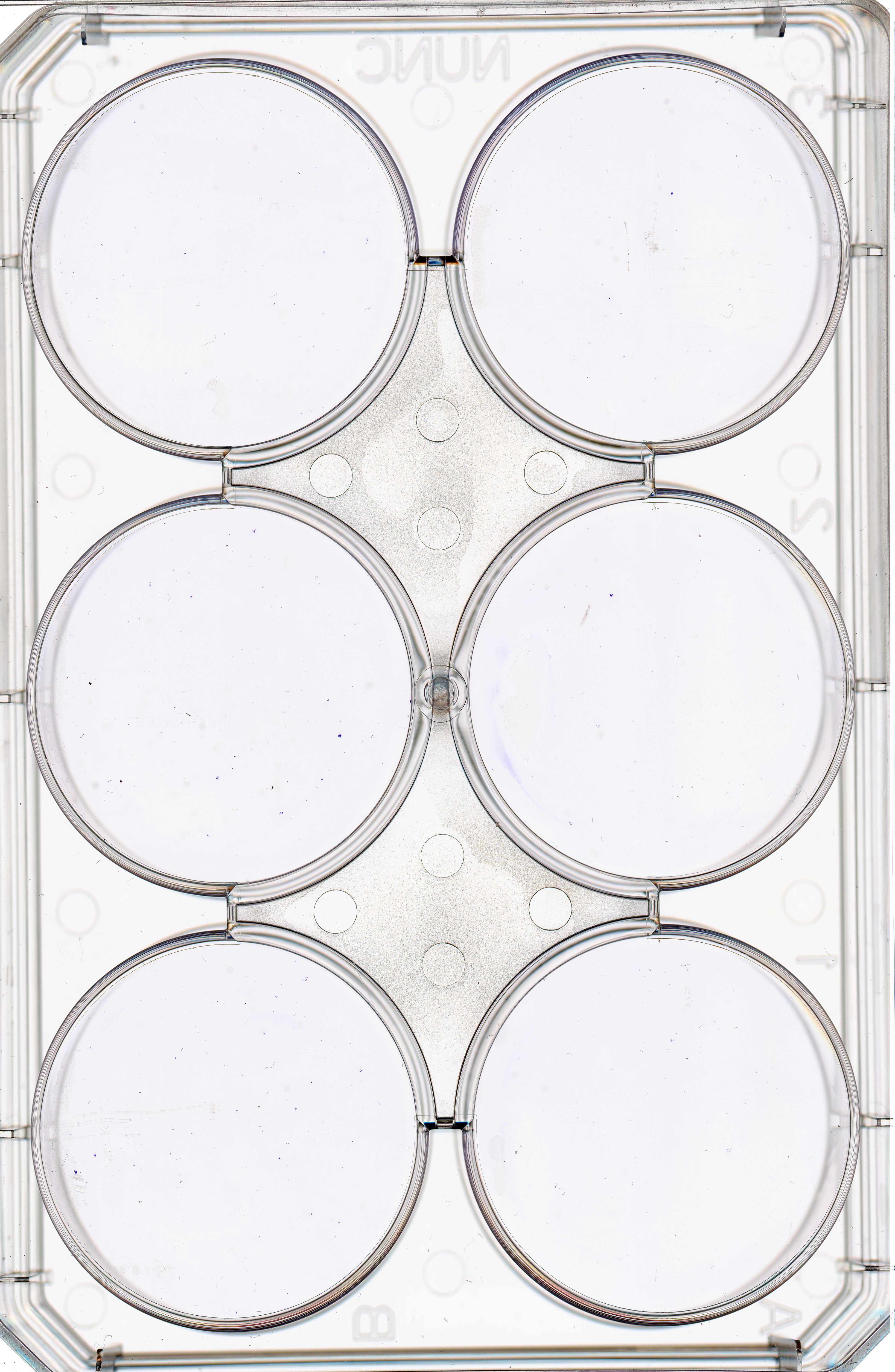

Supplement: Supplementary file 10 — Figure EV1 Source Data [file 44318_2024_108_MOESM10_ESM.zip › EMBOJ-2023-115654_FigEV1_sourcedata/EV1F/E231201 WTsiLuc 5dC1200-1600.jpg]

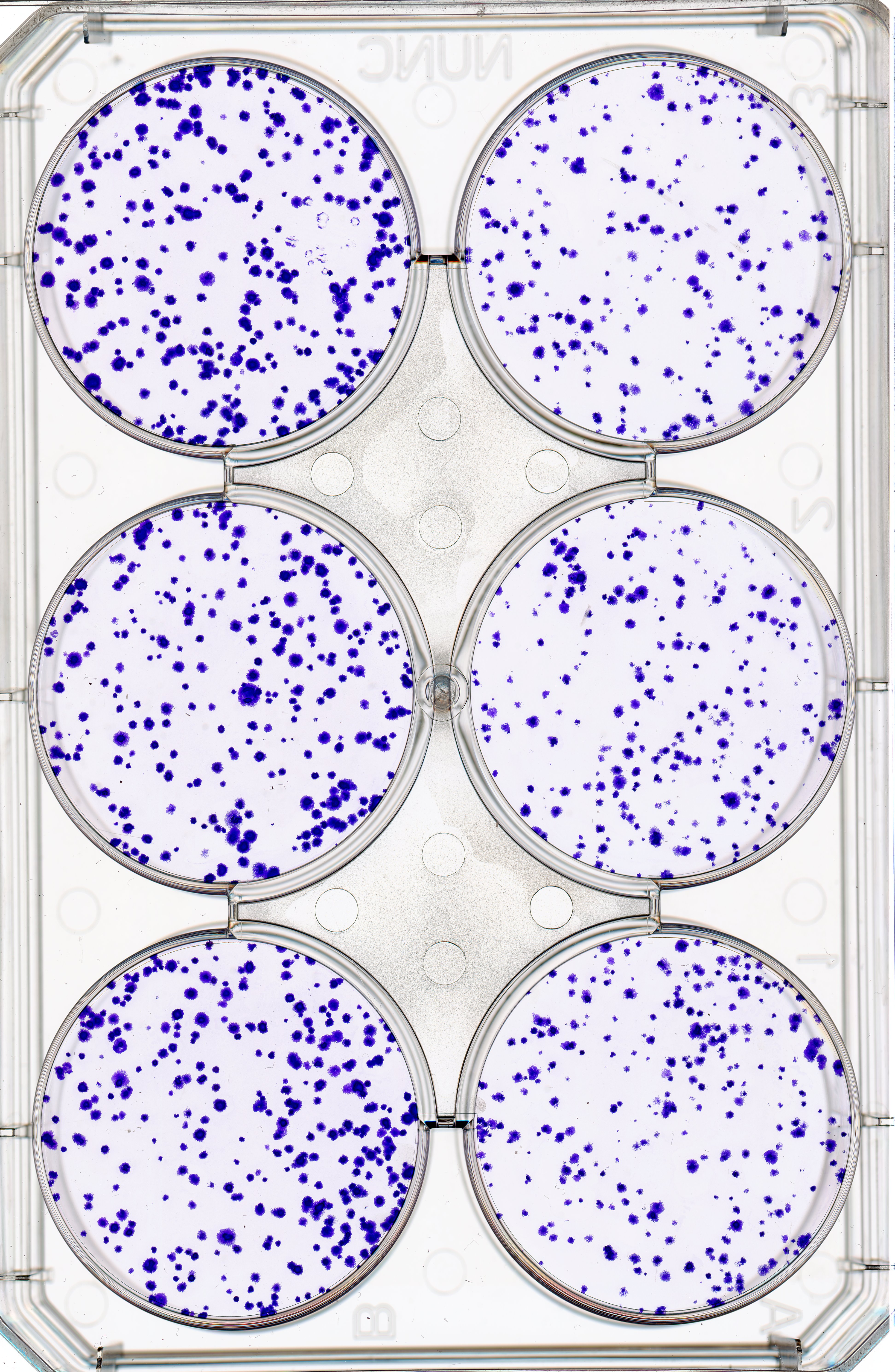

Supplement: Supplementary file 10 — Figure EV1 Source Data [file 44318_2024_108_MOESM10_ESM.zip › EMBOJ-2023-115654_FigEV1_sourcedata/EV1F/E231201 DCTDsiDNMT1 5dC0-200.jpg]

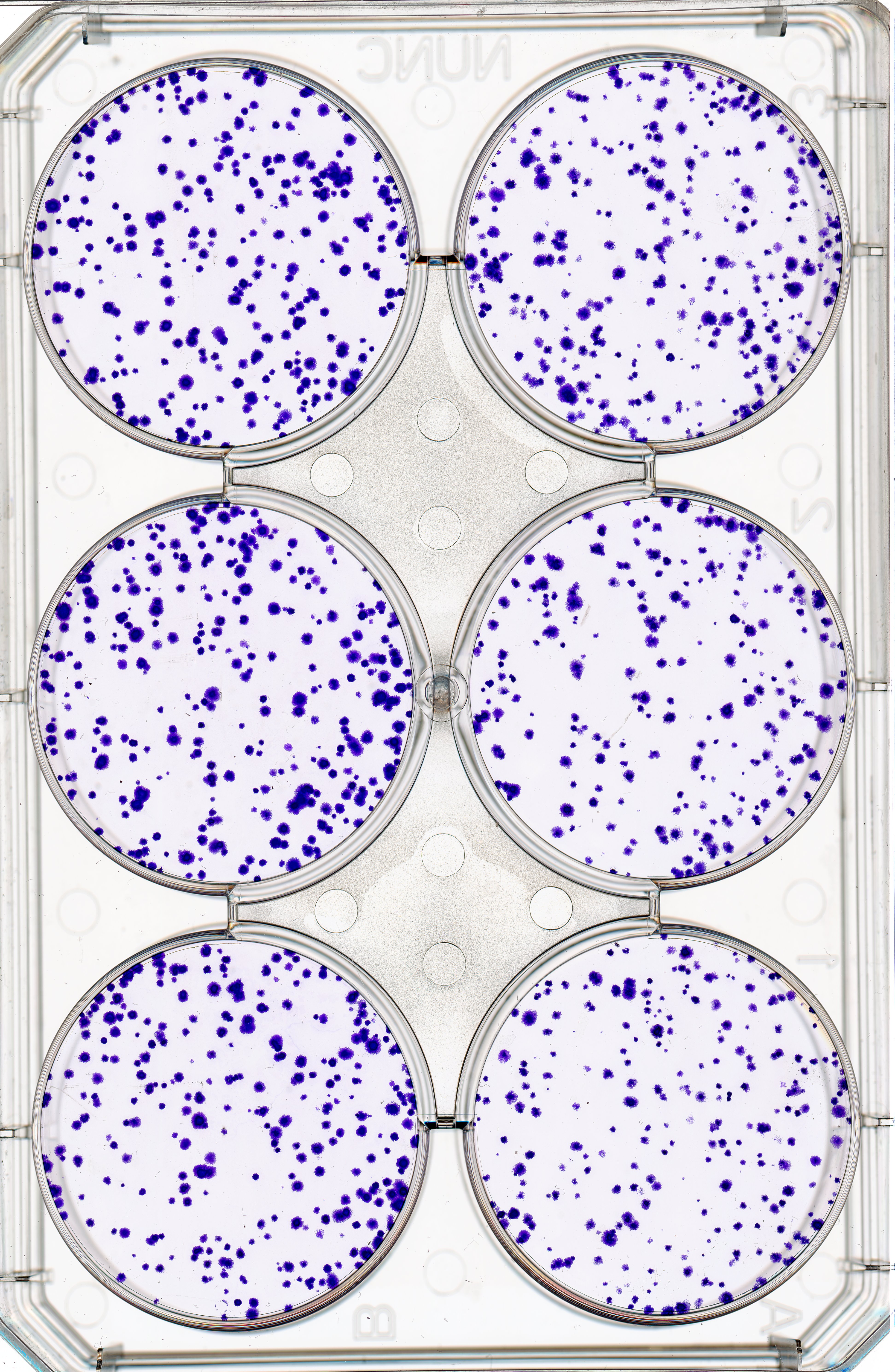

Supplement: Supplementary file 10 — Figure EV1 Source Data [file 44318_2024_108_MOESM10_ESM.zip › EMBOJ-2023-115654_FigEV1_sourcedata/EV1F/E231201 DCTDsiLuc 5dC0-200.jpg]

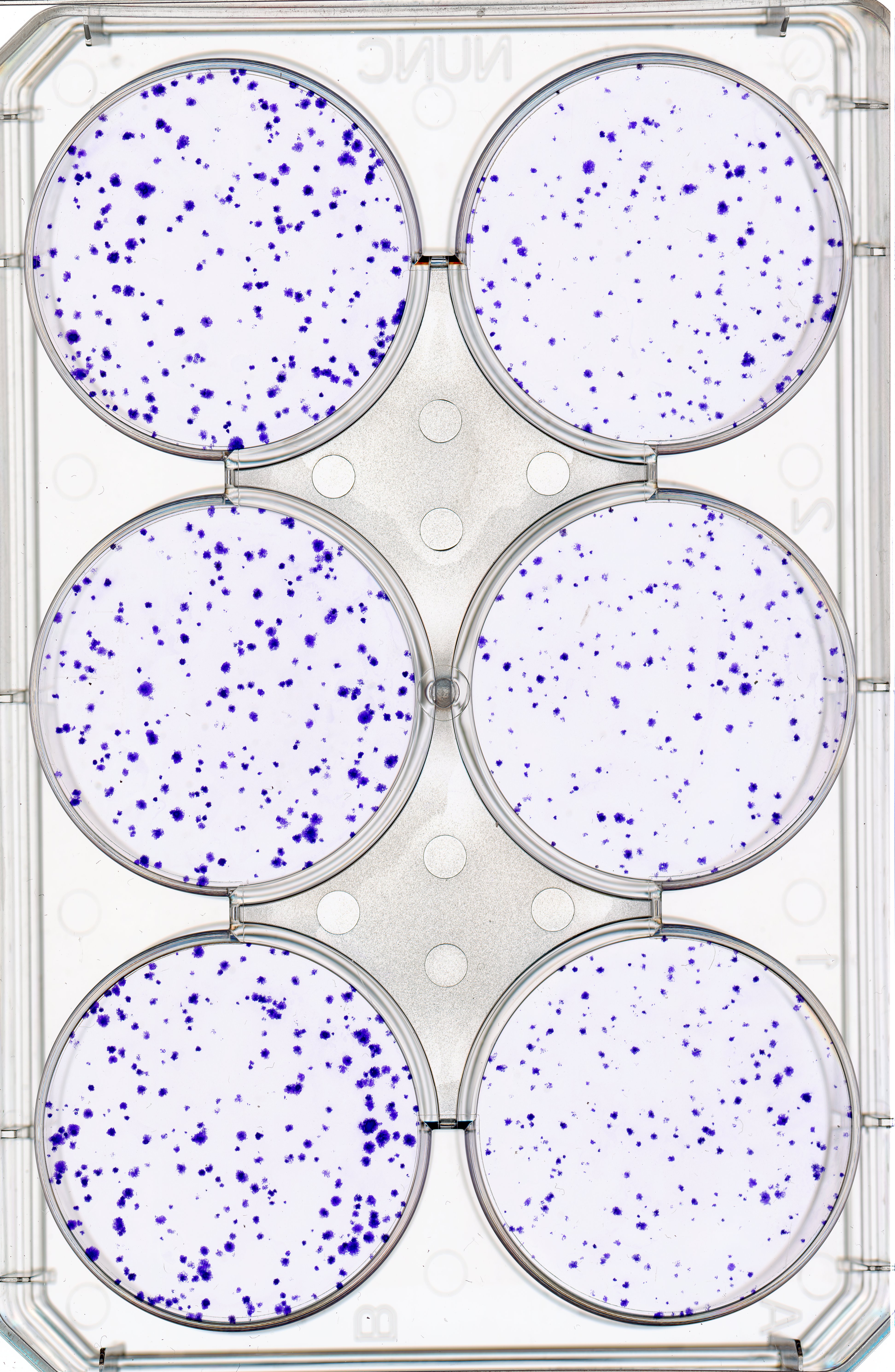

Supplement: Supplementary file 10 — Figure EV1 Source Data [file 44318_2024_108_MOESM10_ESM.zip › EMBOJ-2023-115654_FigEV1_sourcedata/EV1F/E231201 DCTDsiLuc 5dC400-800.jpg]

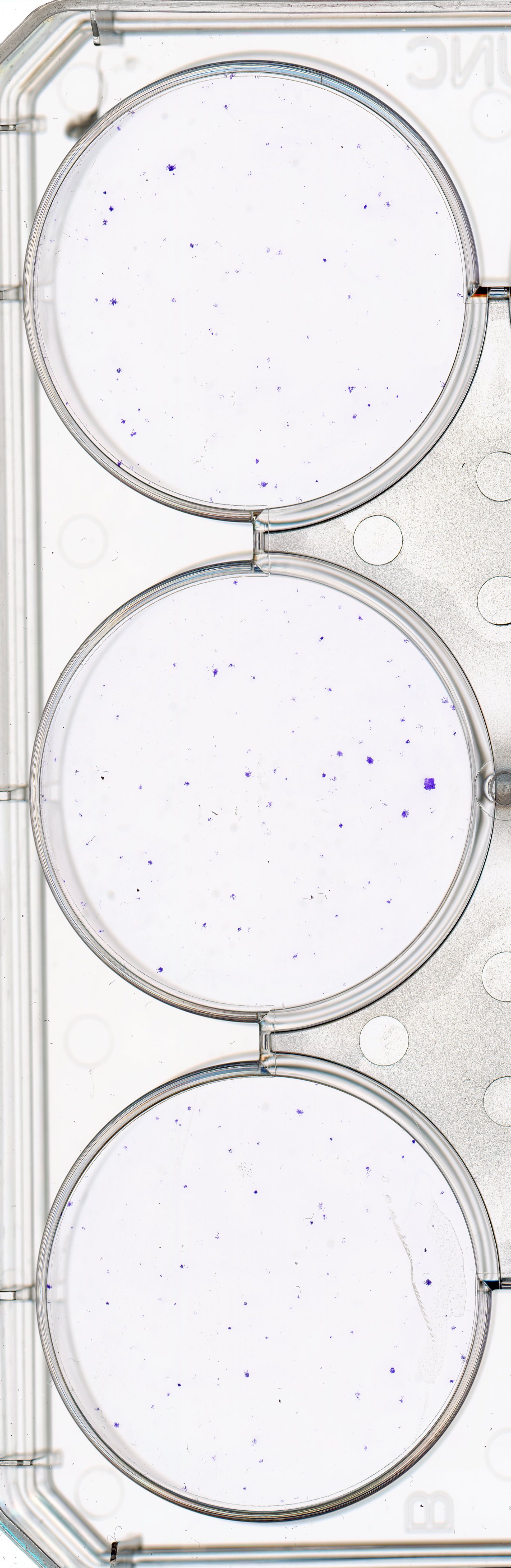

Supplement: Supplementary file 10 — Figure EV1 Source Data [file 44318_2024_108_MOESM10_ESM.zip › EMBOJ-2023-115654_FigEV1_sourcedata/EV1F/E231201 DCTDsiLuc 5dC2000.jpg]

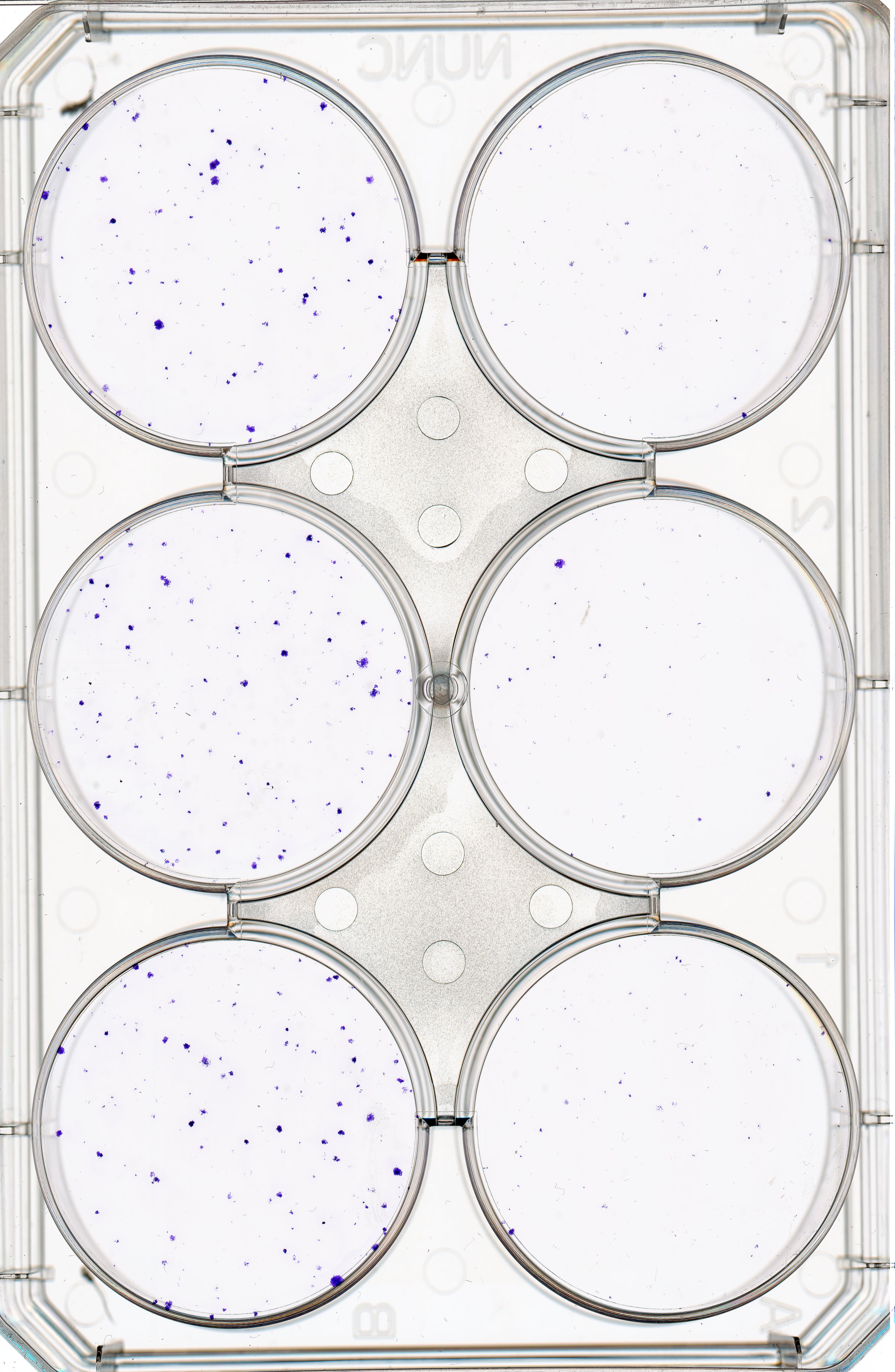

Supplement: Supplementary file 10 — Figure EV1 Source Data [file 44318_2024_108_MOESM10_ESM.zip › EMBOJ-2023-115654_FigEV1_sourcedata/EV1F/E231201 WTsiLuc 5dC400-800.jpg]

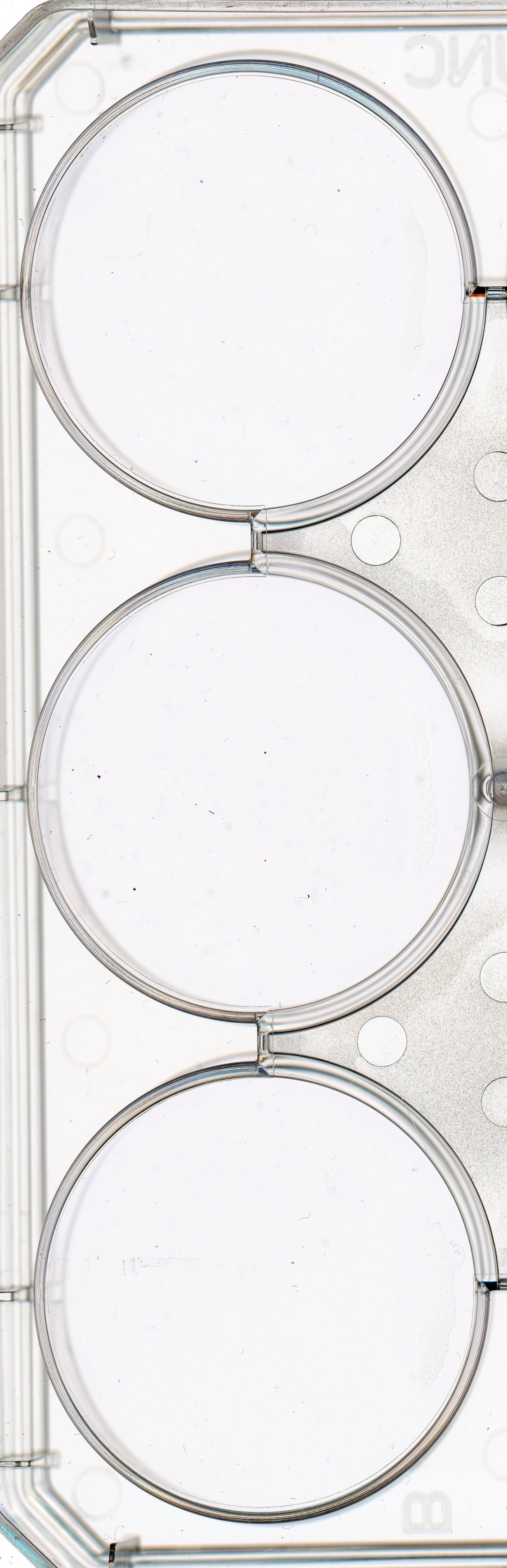

Supplement: Supplementary file 10 — Figure EV1 Source Data [file 44318_2024_108_MOESM10_ESM.zip › EMBOJ-2023-115654_FigEV1_sourcedata/EV1F/E231201 WTsiLuc 5dC2000.jpg]

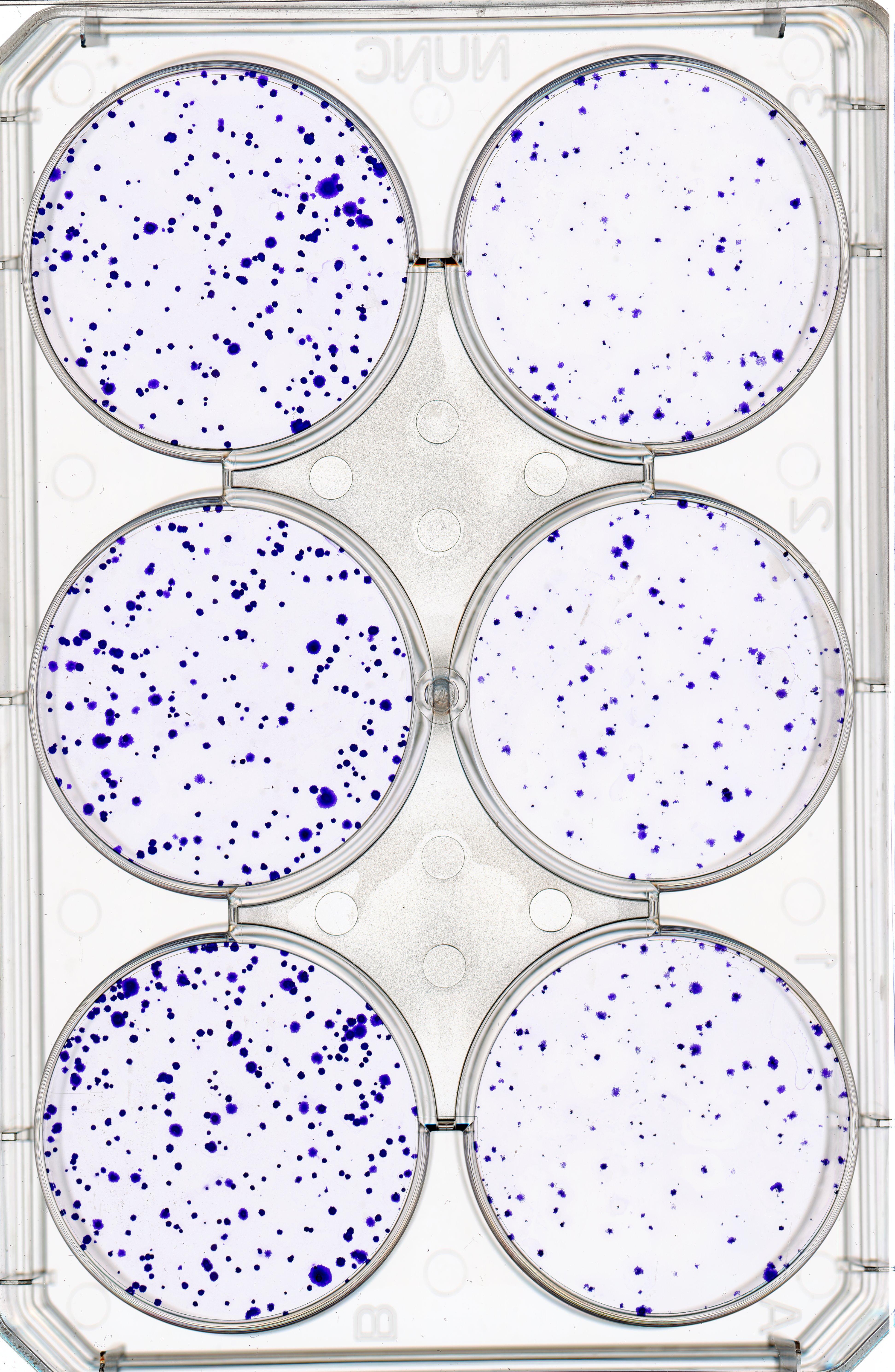

Supplement: Supplementary file 10 — Figure EV1 Source Data [file 44318_2024_108_MOESM10_ESM.zip › EMBOJ-2023-115654_FigEV1_sourcedata/EV1F/E231201 WTsiLuc 5dC0-200.jpg]

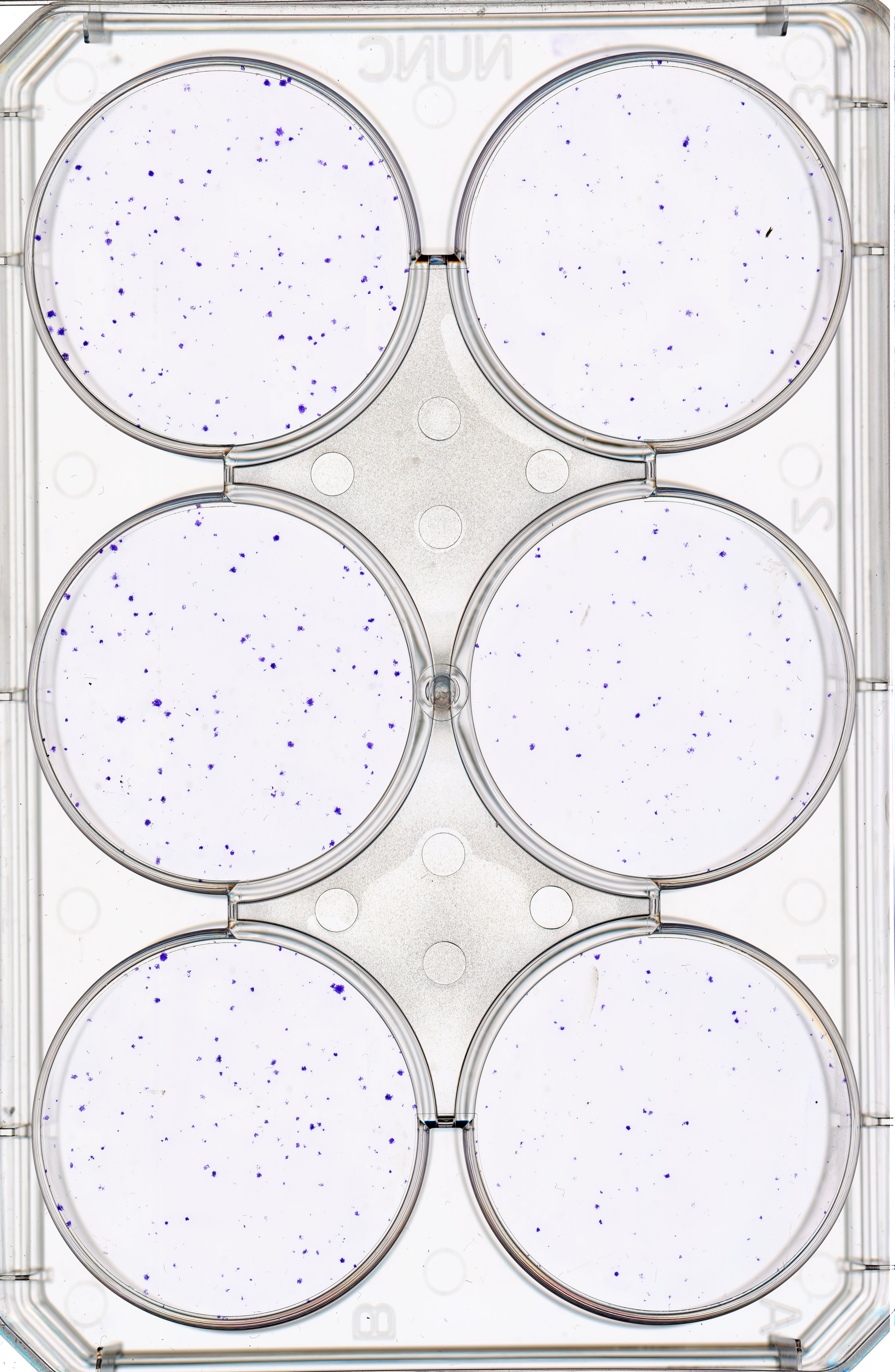

Supplement: Supplementary file 10 — Figure EV1 Source Data [file 44318_2024_108_MOESM10_ESM.zip › EMBOJ-2023-115654_FigEV1_sourcedata/EV1F/E231201 DCTDsiLuc 5dC1200-1600.jpg]

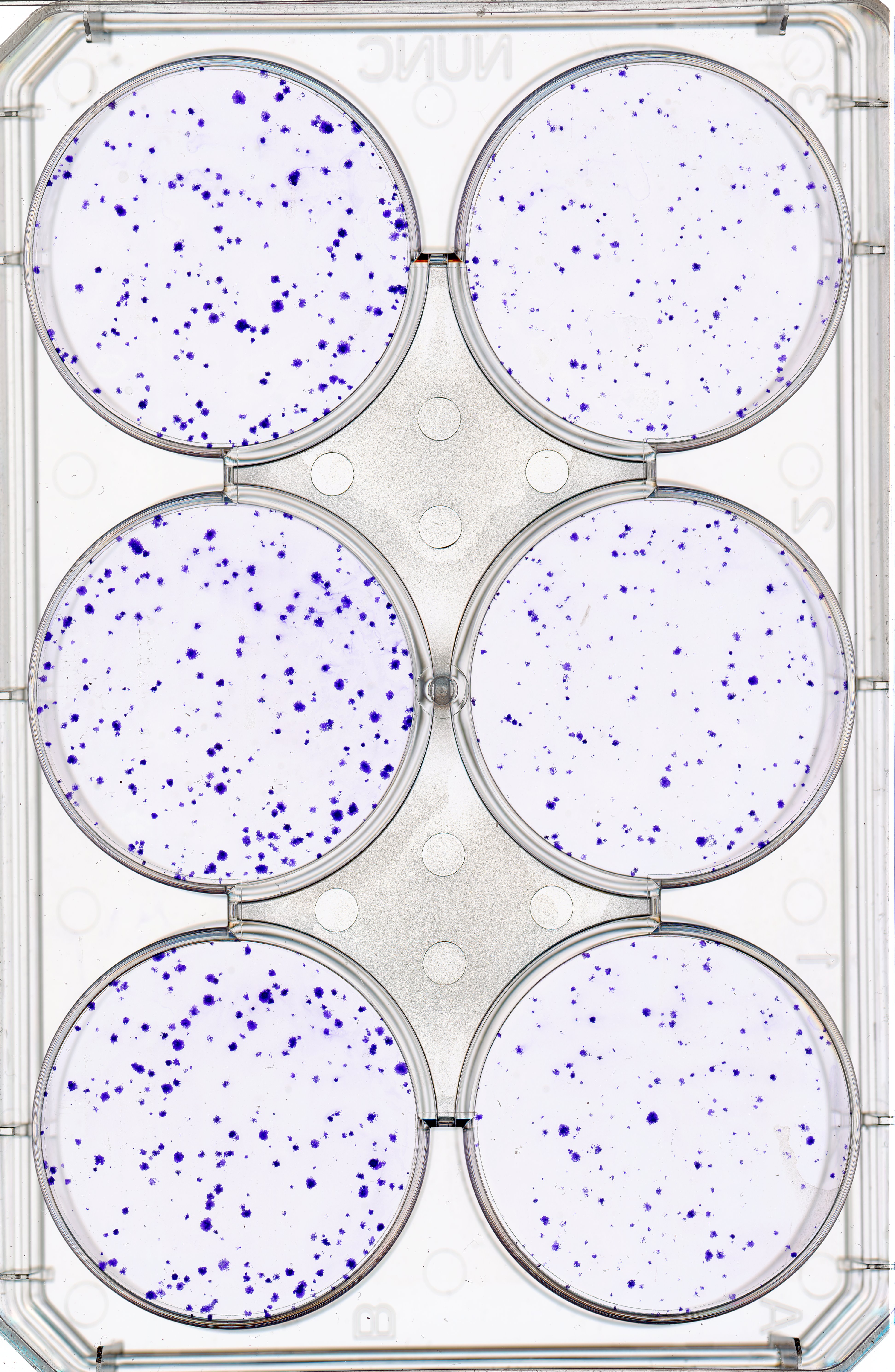

Supplement: Supplementary file 10 — Figure EV1 Source Data [file 44318_2024_108_MOESM10_ESM.zip › EMBOJ-2023-115654_FigEV1_sourcedata/EV1F/E231201 DCTDsiDNMT1 5dC400-800.jpg]

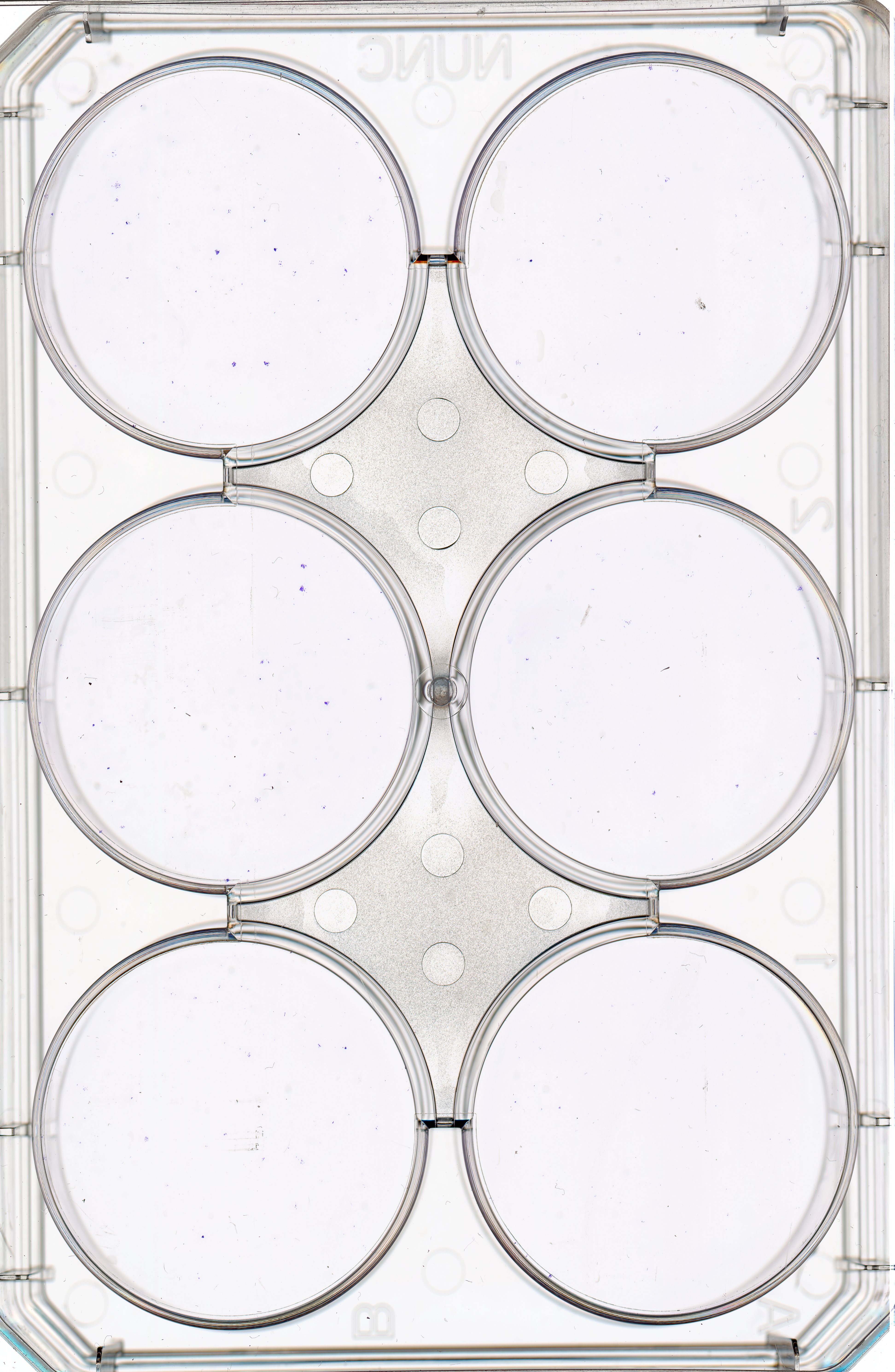

Supplement: Supplementary file 10 — Figure EV1 Source Data [file 44318_2024_108_MOESM10_ESM.zip › EMBOJ-2023-115654_FigEV1_sourcedata/EV1F/E231201 WTsiDNMT1 5dC1200-1600.jpg]

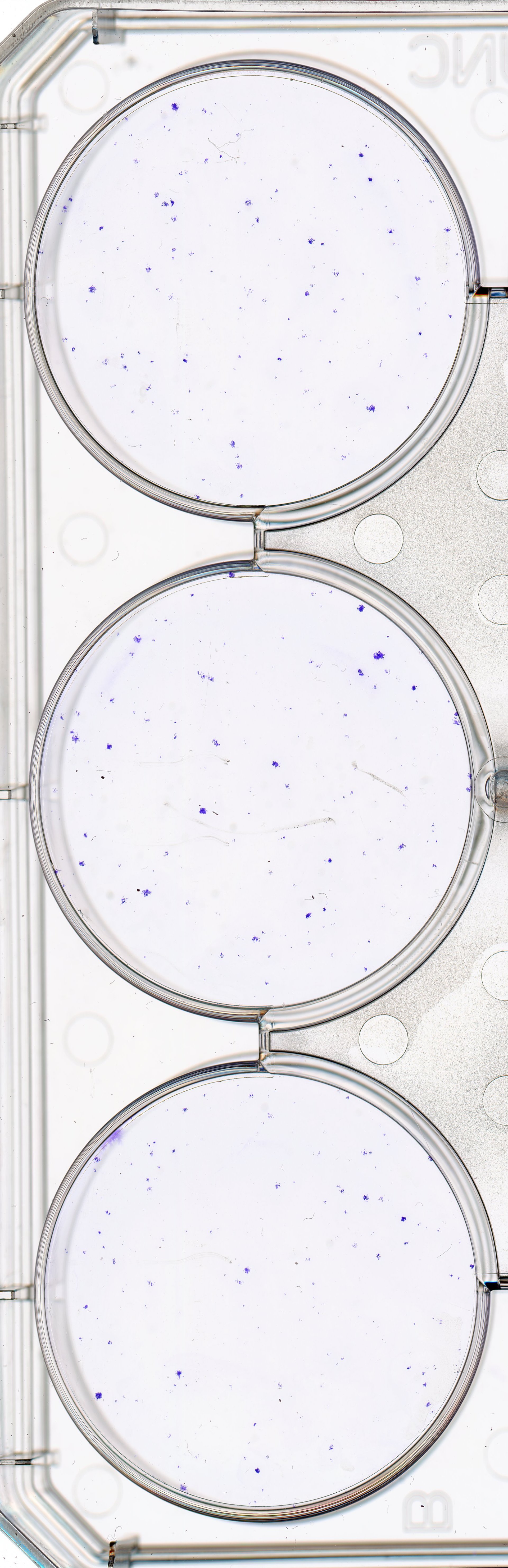

Supplement: Supplementary file 10 — Figure EV1 Source Data [file 44318_2024_108_MOESM10_ESM.zip › EMBOJ-2023-115654_FigEV1_sourcedata/EV1F/E231201 DCTDsiDNMT1 5dC2000.jpg]

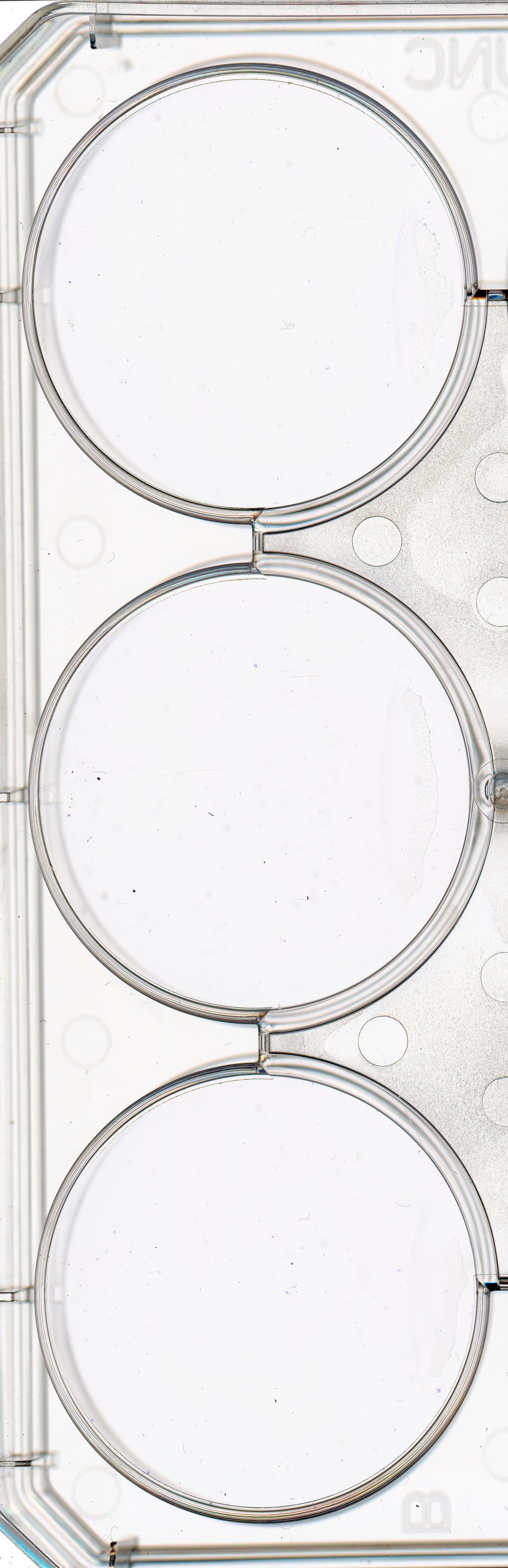

Supplement: Supplementary file 10 — Figure EV1 Source Data [file 44318_2024_108_MOESM10_ESM.zip › EMBOJ-2023-115654_FigEV1_sourcedata/EV1F/E231201 WTsiDNMT1 5dC2000.jpg]

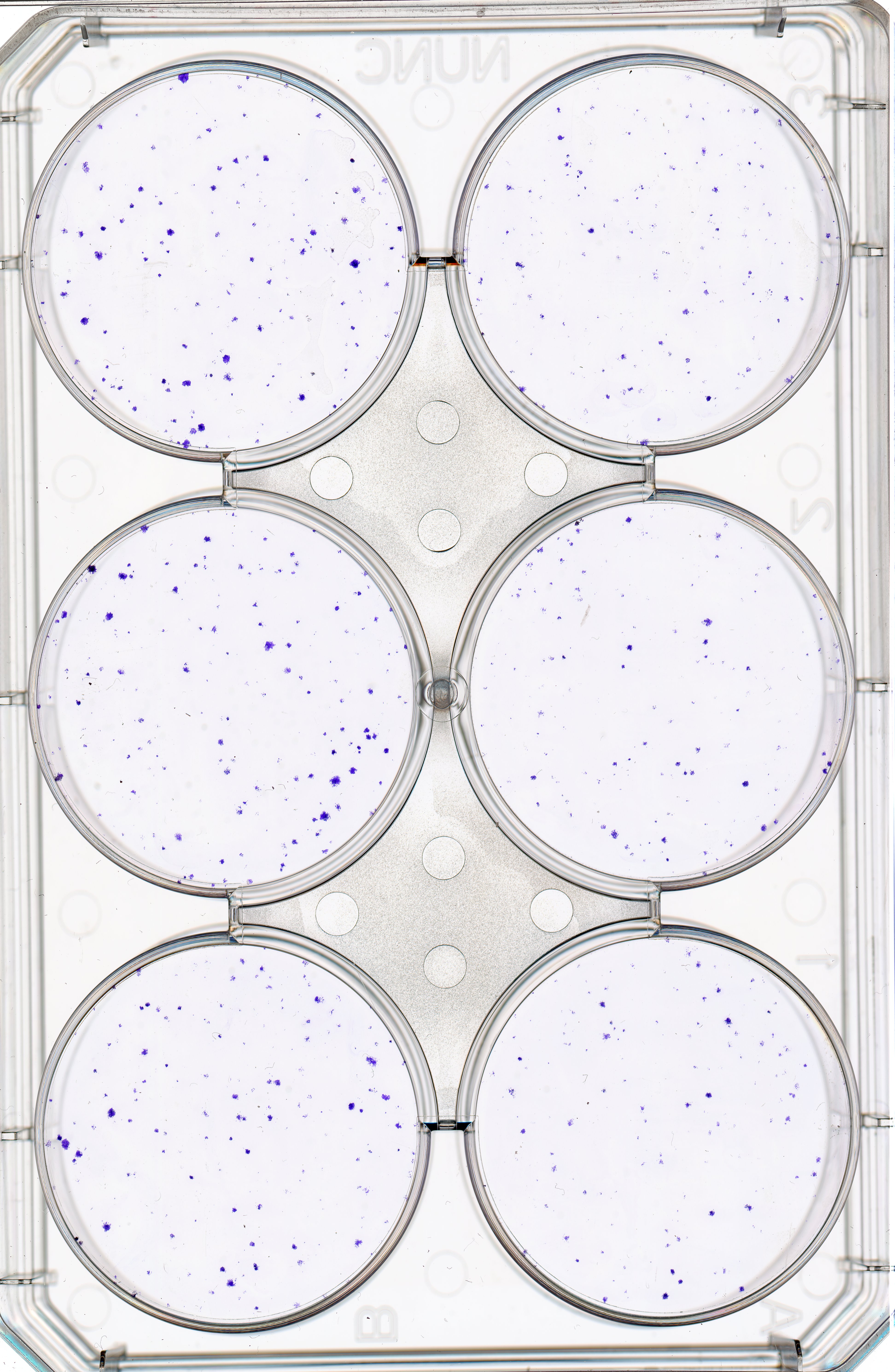

Supplement: Supplementary file 10 — Figure EV1 Source Data [file 44318_2024_108_MOESM10_ESM.zip › EMBOJ-2023-115654_FigEV1_sourcedata/EV1F/E231201 DCTDsiDNMT1 5dC1200-1600.jpg]

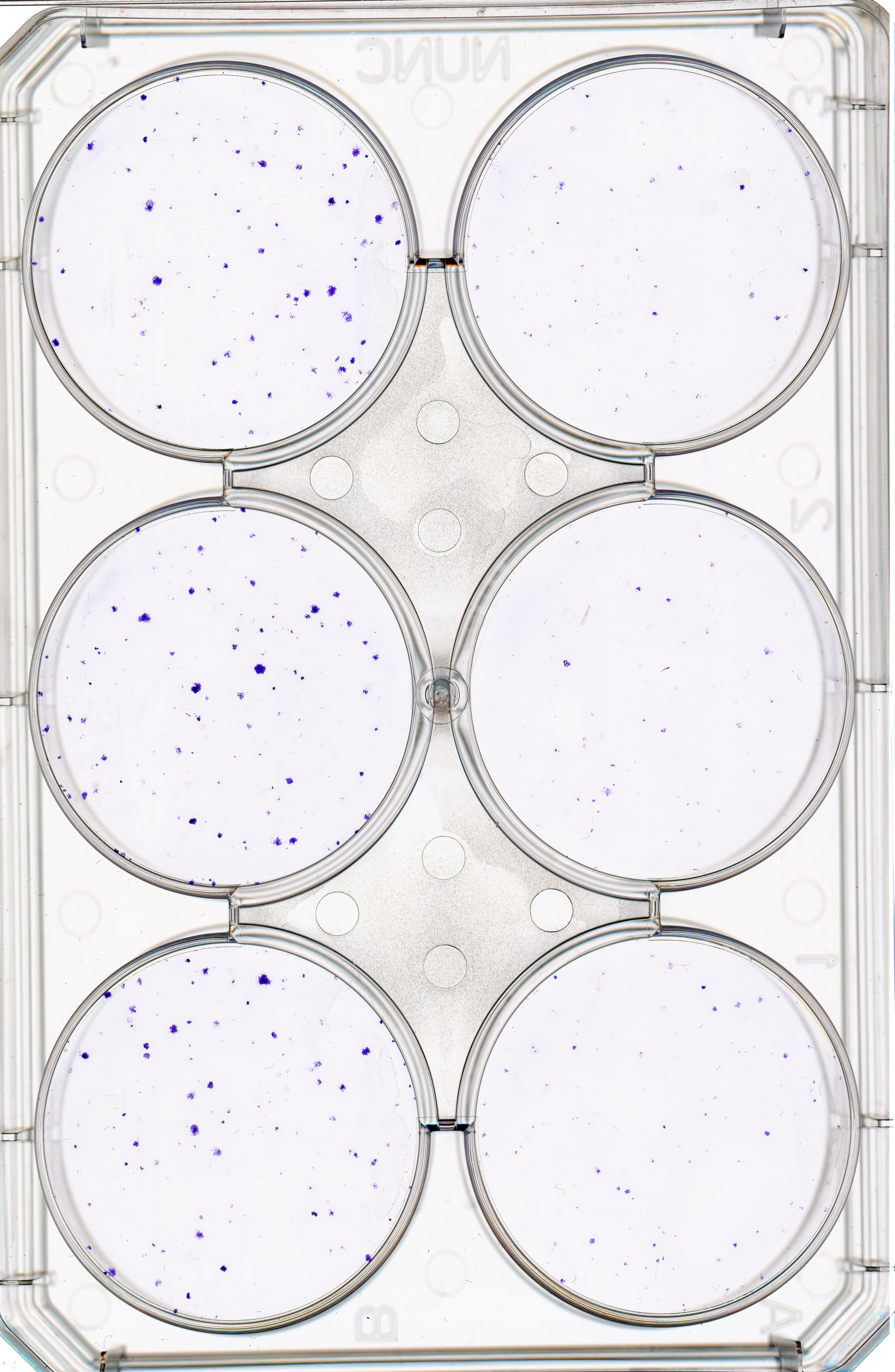

Supplement: Supplementary file 10 — Figure EV1 Source Data [file 44318_2024_108_MOESM10_ESM.zip › EMBOJ-2023-115654_FigEV1_sourcedata/EV1F/E231201 WTsiDNMT1 5dC400-800.jpg]
