# Supplementary figures and images for: Decitabine cytotoxicity is promoted by dCMP deaminase DCTD and mitigated by SUMO-dependent E3 ligase TOPORS (part 2 of 3)
Source: EMBO J. 2024 May 17;43(12):6. doi: 10.1038/s44318-024-00108-2 (PMC11183266; doi:10.1038/s44318-024-00108-2)

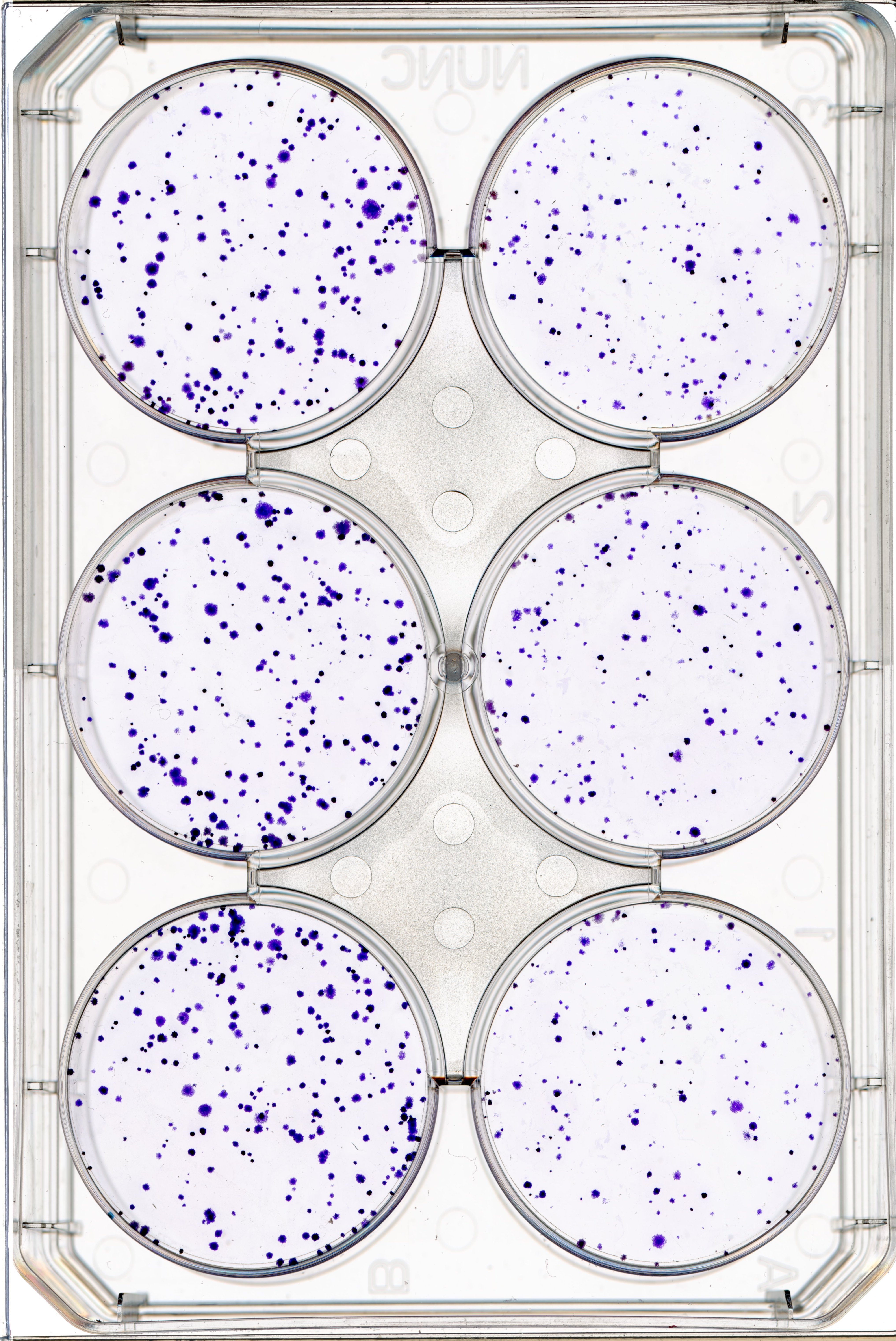

Supplement: Supplementary file 11 — Figure EV3 Source Data [file 44318_2024_108_MOESM11_ESM.zip › EMBOJ-2023-115654_FigEV3_sourcedata/EV3I/HAP1_TOPORS KO_CPT_0.5_1.jpg]

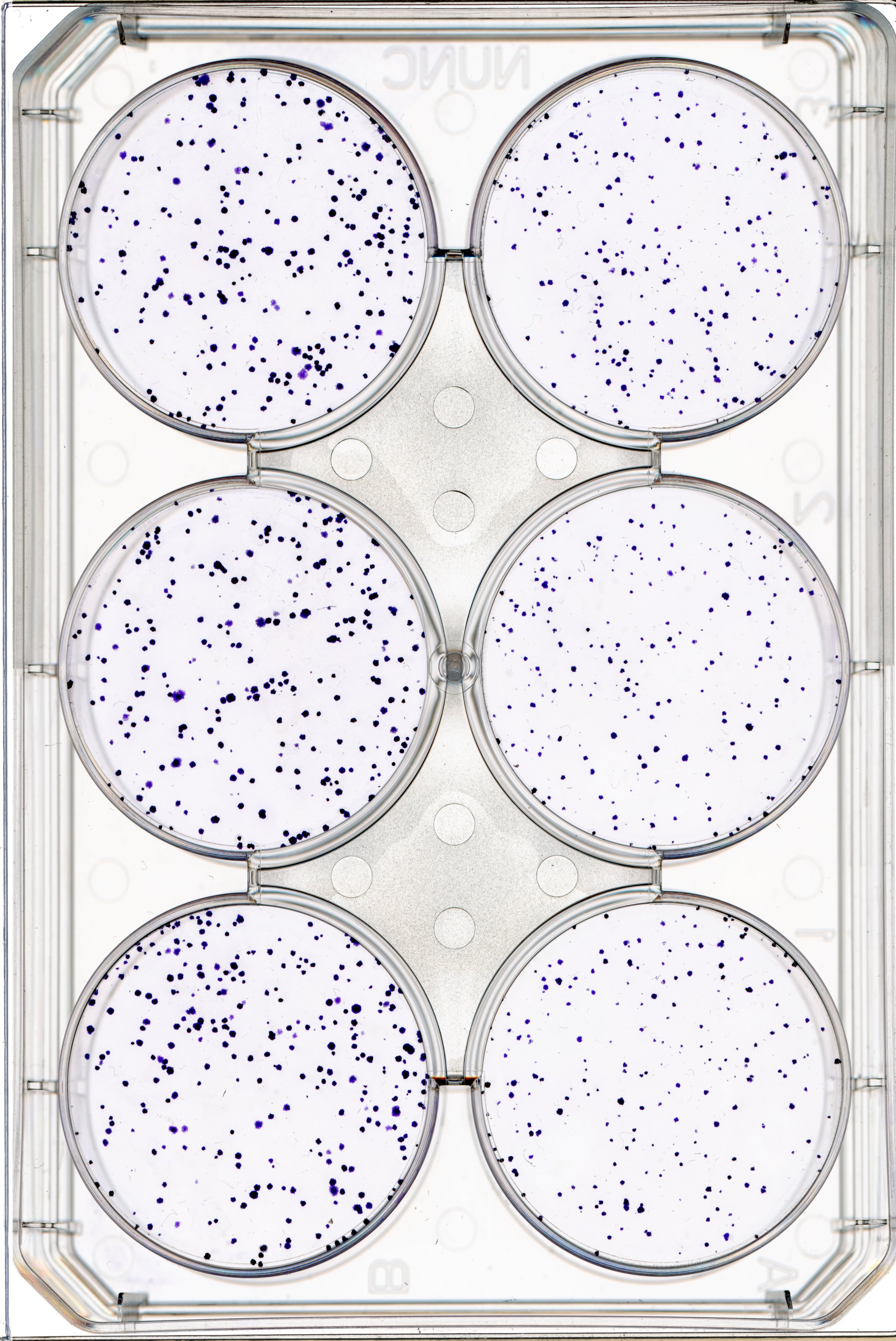

Supplement: Supplementary file 11 — Figure EV3 Source Data [file 44318_2024_108_MOESM11_ESM.zip › EMBOJ-2023-115654_FigEV3_sourcedata/EV3I/HAP1_WT_CPT_2_4.jpg]

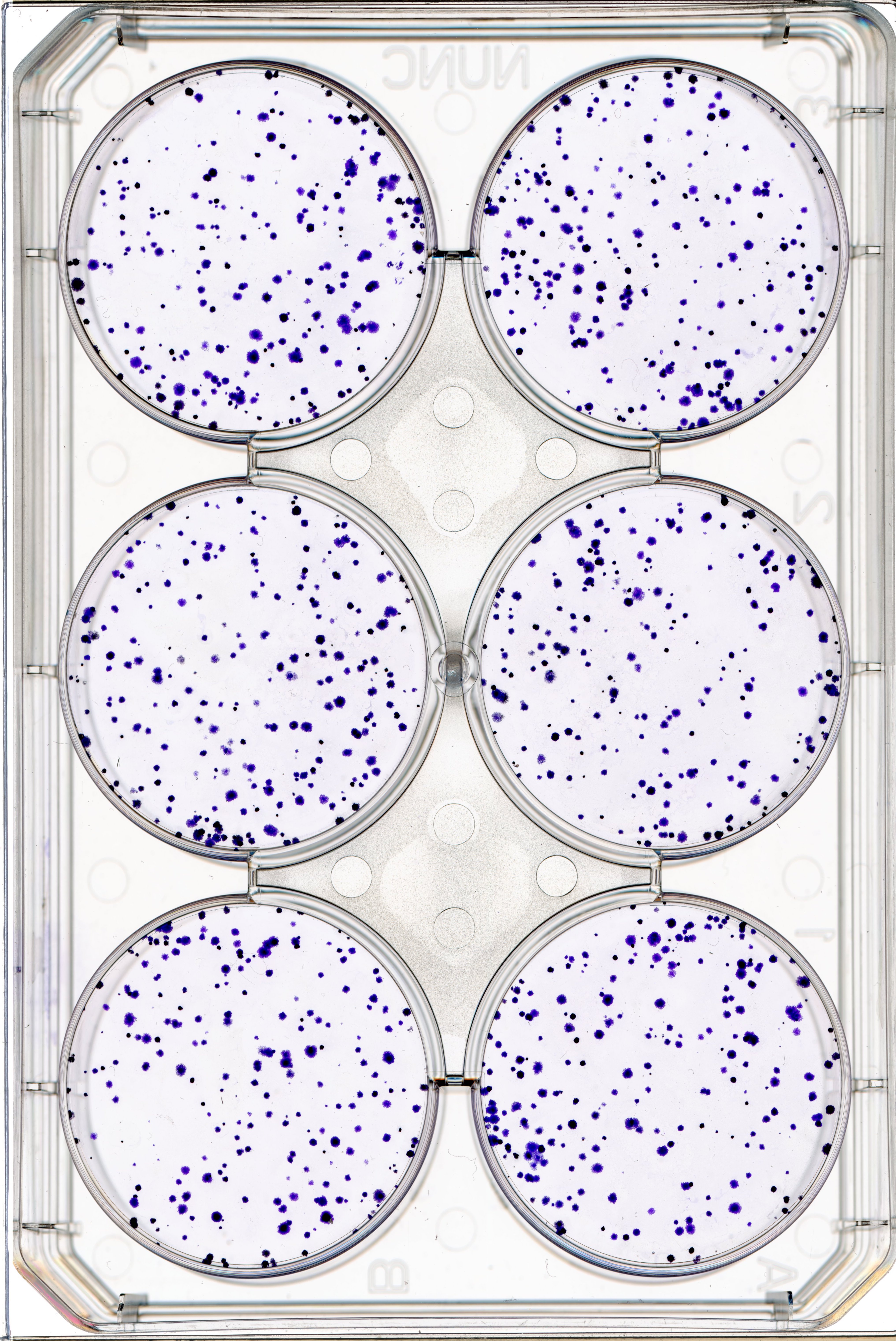

Supplement: Supplementary file 11 — Figure EV3 Source Data [file 44318_2024_108_MOESM11_ESM.zip › EMBOJ-2023-115654_FigEV3_sourcedata/EV3I/HAP1_TOPORS KO_CPT_0_0.2.jpg]

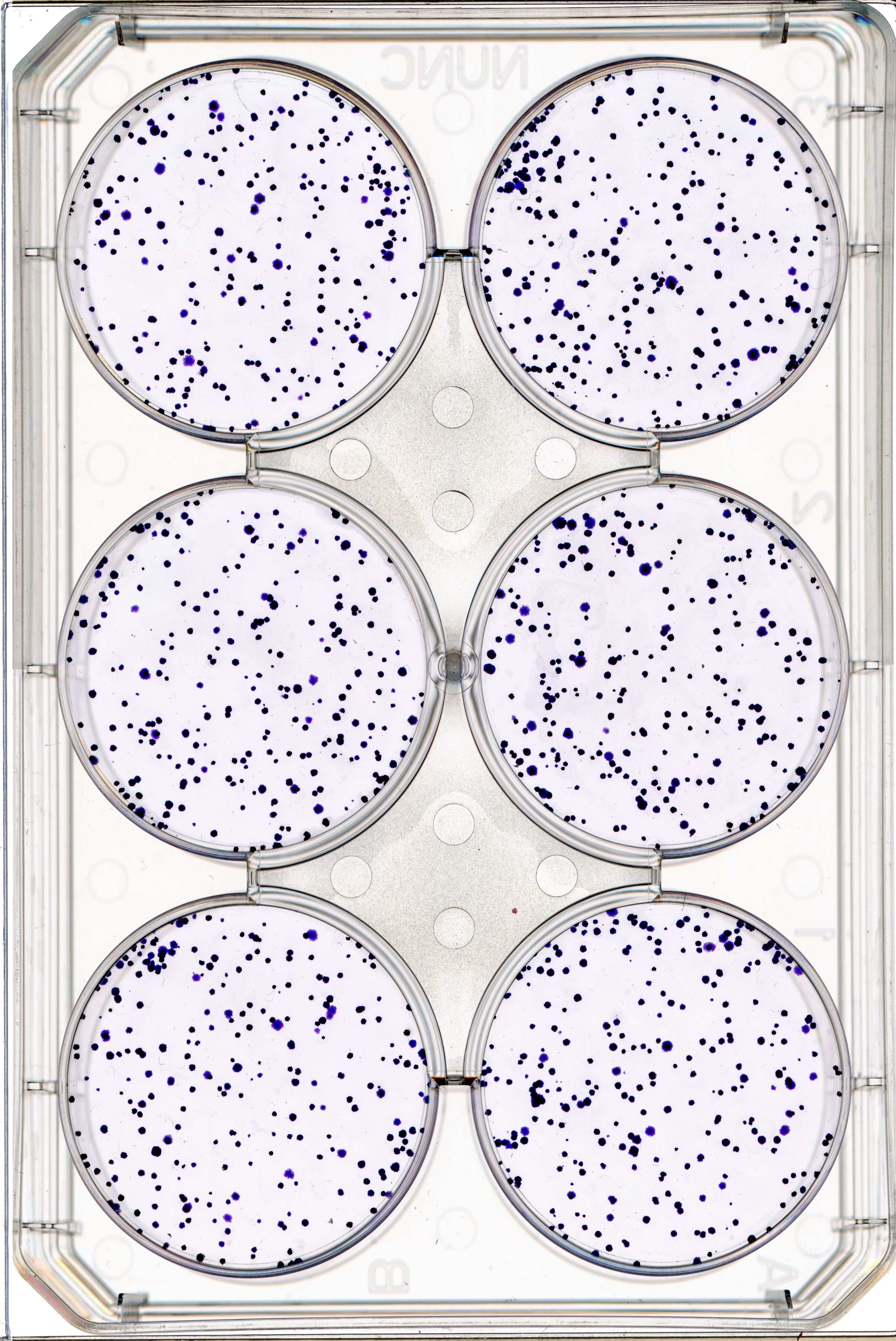

Supplement: Supplementary file 11 — Figure EV3 Source Data [file 44318_2024_108_MOESM11_ESM.zip › EMBOJ-2023-115654_FigEV3_sourcedata/EV3I/HAP1_WT_CPT_0_0.2.jpg]

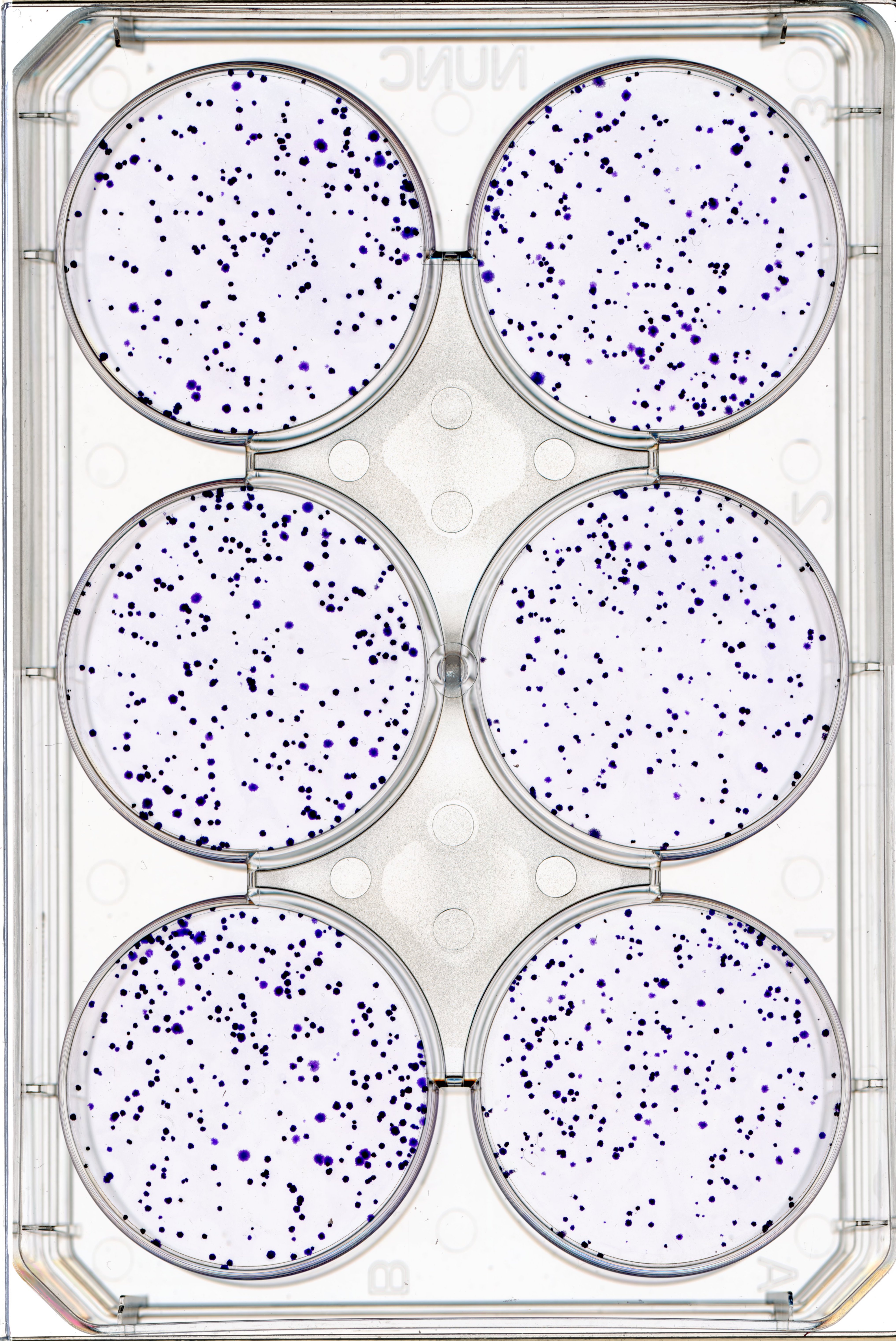

Supplement: Supplementary file 11 — Figure EV3 Source Data [file 44318_2024_108_MOESM11_ESM.zip › EMBOJ-2023-115654_FigEV3_sourcedata/EV3I/HAP1_WT_CPT_0.5_1.jpg]

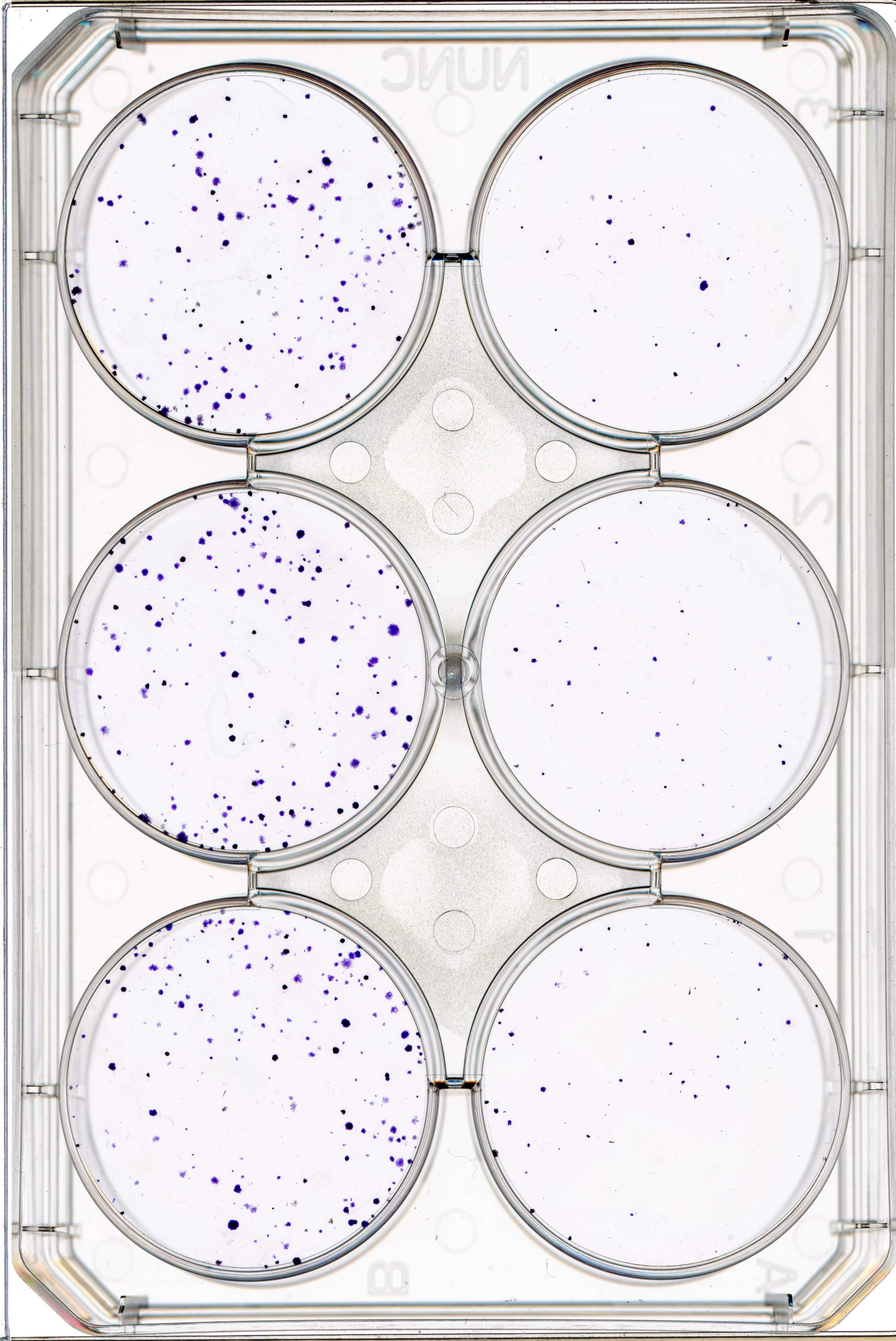

Supplement: Supplementary file 11 — Figure EV3 Source Data [file 44318_2024_108_MOESM11_ESM.zip › EMBOJ-2023-115654_FigEV3_sourcedata/EV3I/HAP1_TOPORS KO_CPT_2_4.jpg]

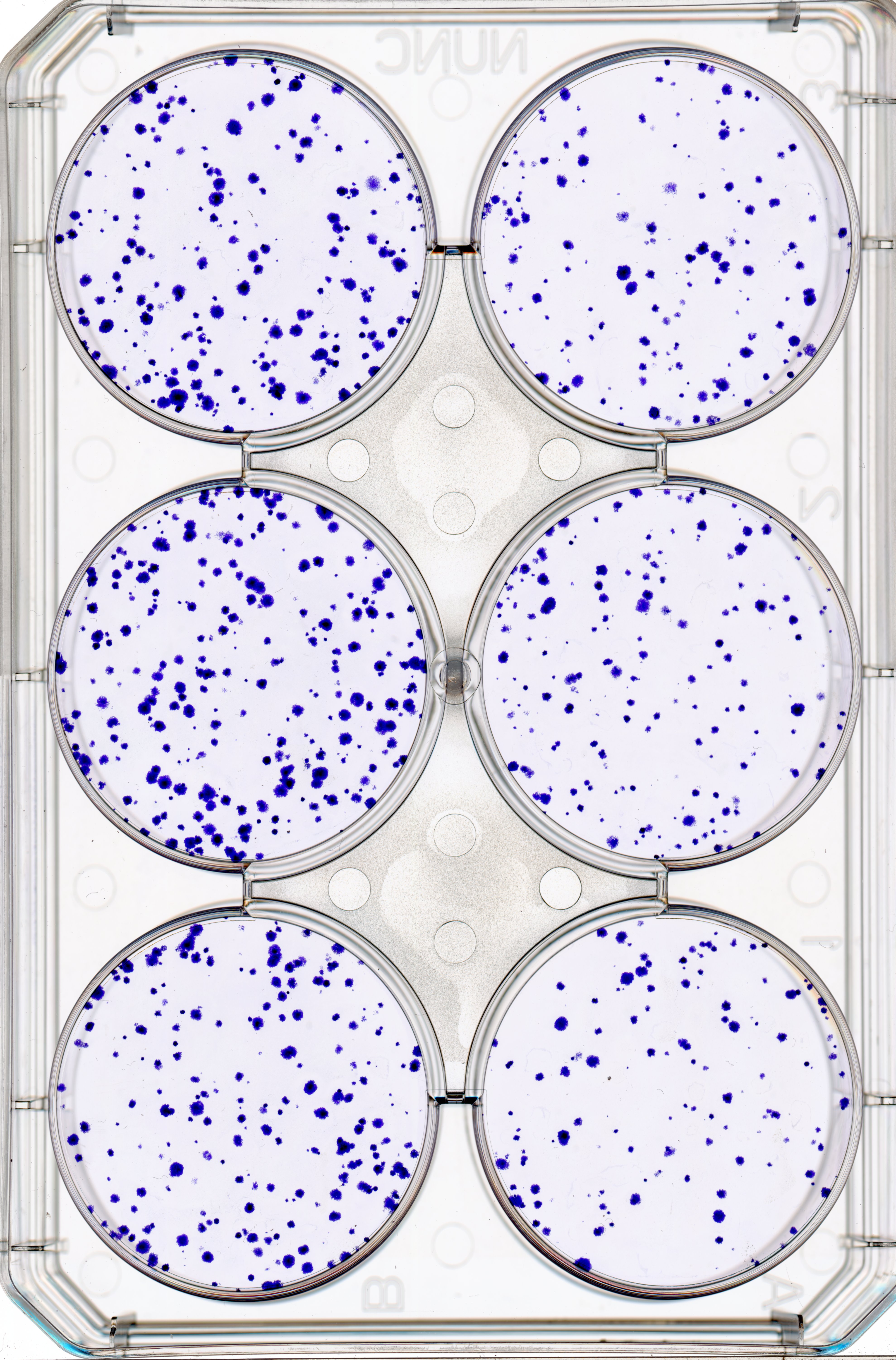

Supplement: Supplementary file 11 — Figure EV3 Source Data [file 44318_2024_108_MOESM11_ESM.zip › EMBOJ-2023-115654_FigEV3_sourcedata/EV3F/E230215 TOPORS FA30-40.jpg]

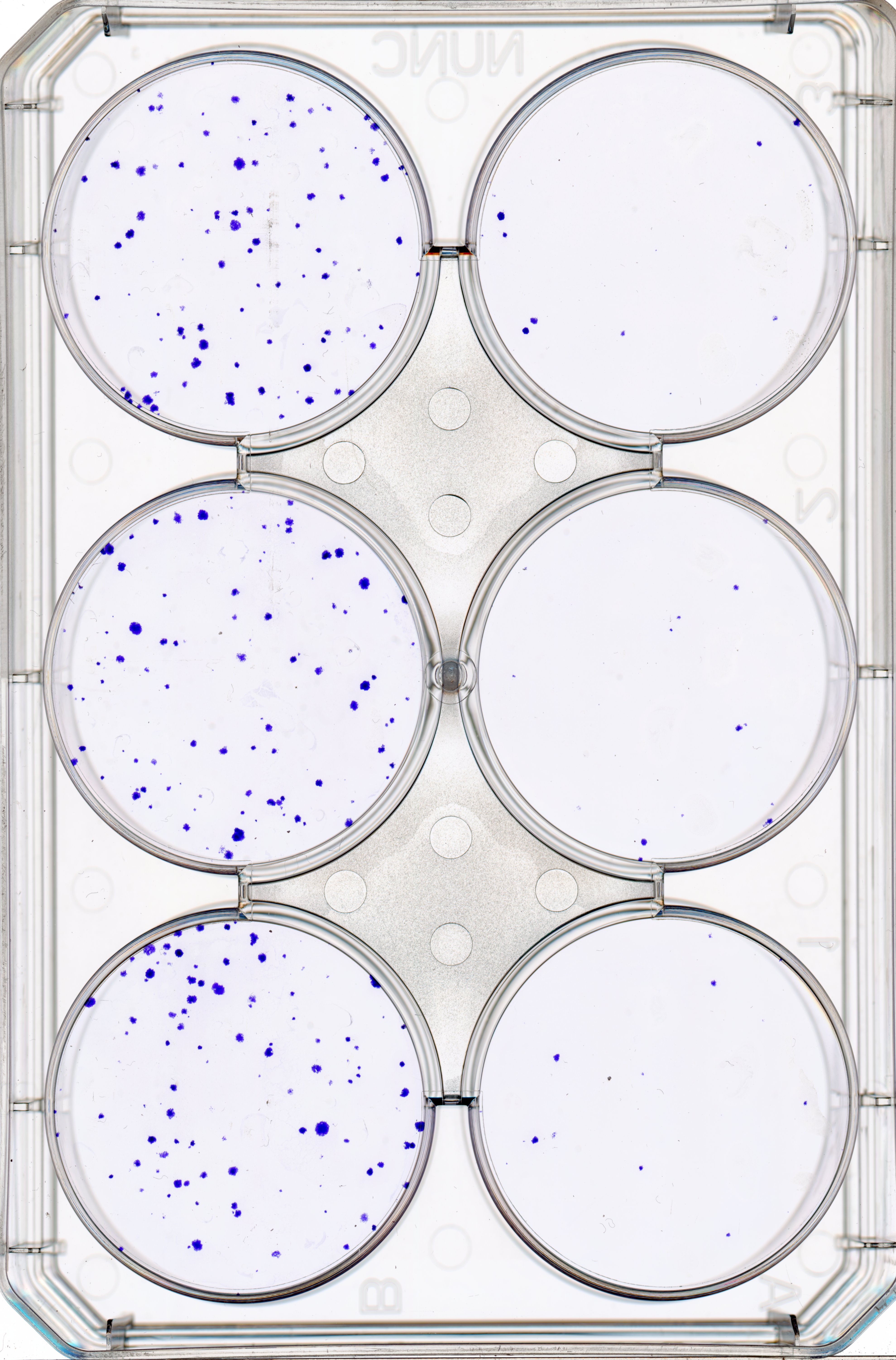

Supplement: Supplementary file 11 — Figure EV3 Source Data [file 44318_2024_108_MOESM11_ESM.zip › EMBOJ-2023-115654_FigEV3_sourcedata/EV3F/E230215 TOPORS FA50-75.jpg]

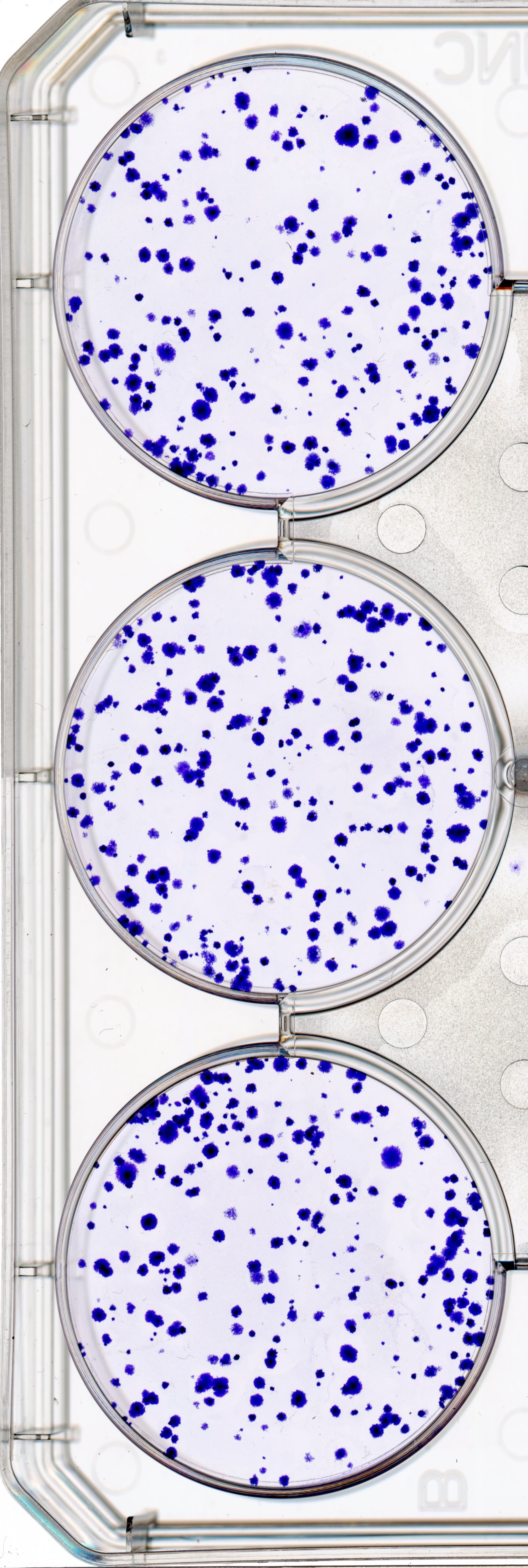

Supplement: Supplementary file 11 — Figure EV3 Source Data [file 44318_2024_108_MOESM11_ESM.zip › EMBOJ-2023-115654_FigEV3_sourcedata/EV3F/E230215 TOPORS FA0.jpg]

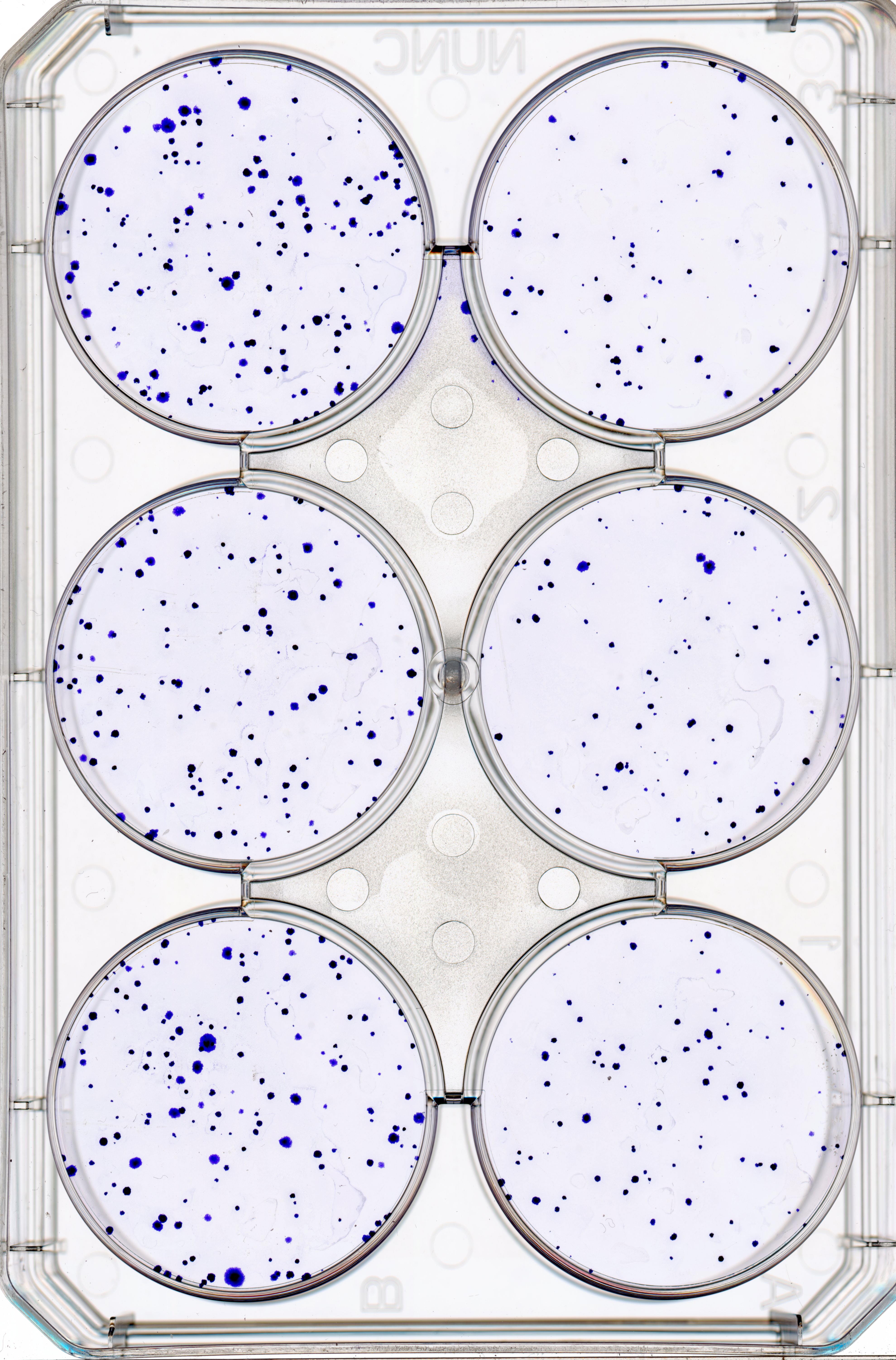

Supplement: Supplementary file 11 — Figure EV3 Source Data [file 44318_2024_108_MOESM11_ESM.zip › EMBOJ-2023-115654_FigEV3_sourcedata/EV3F/E230215 WT FA50-75.jpg]

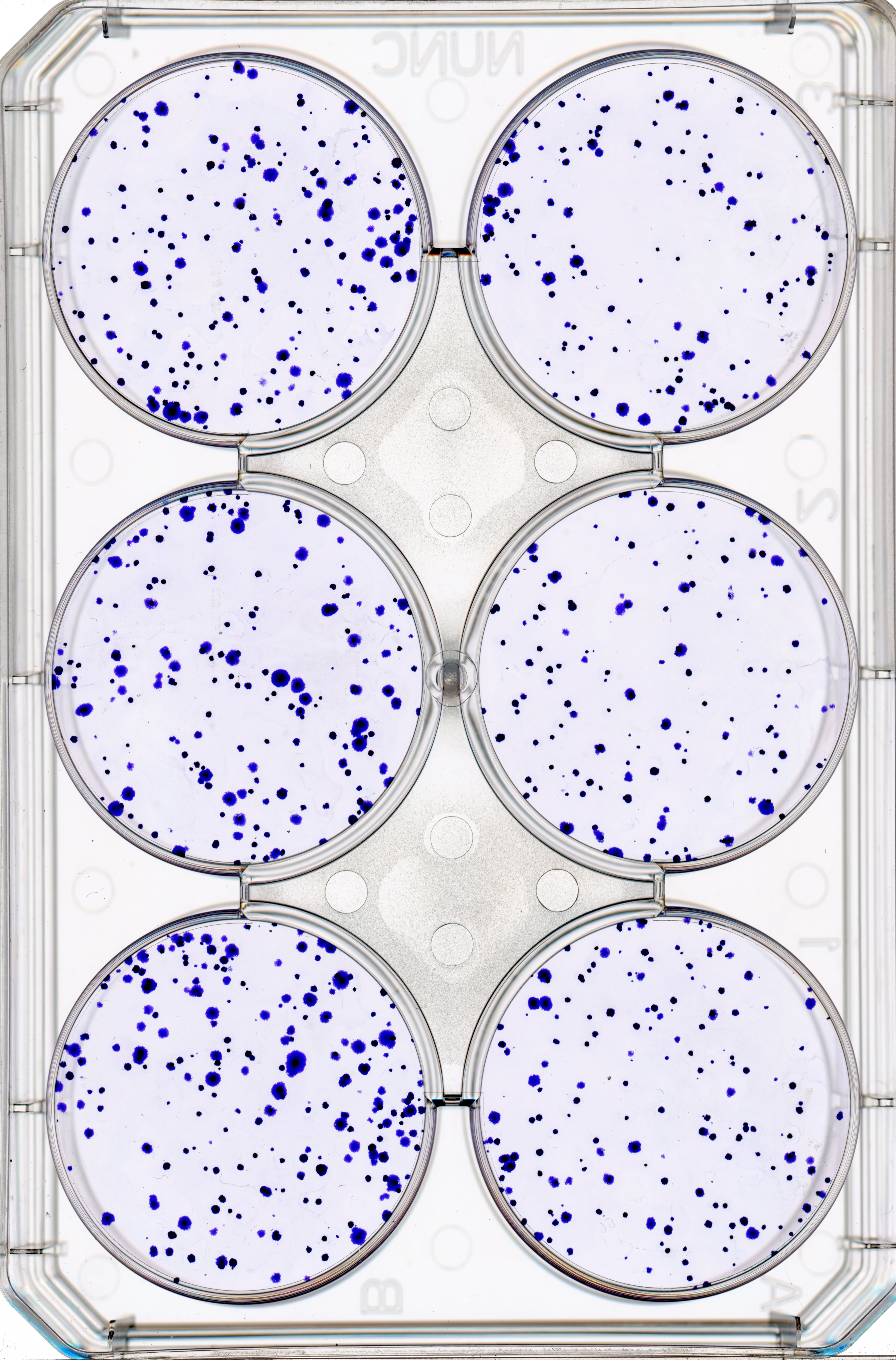

Supplement: Supplementary file 11 — Figure EV3 Source Data [file 44318_2024_108_MOESM11_ESM.zip › EMBOJ-2023-115654_FigEV3_sourcedata/EV3F/E230215 WT FA30-40.jpg]

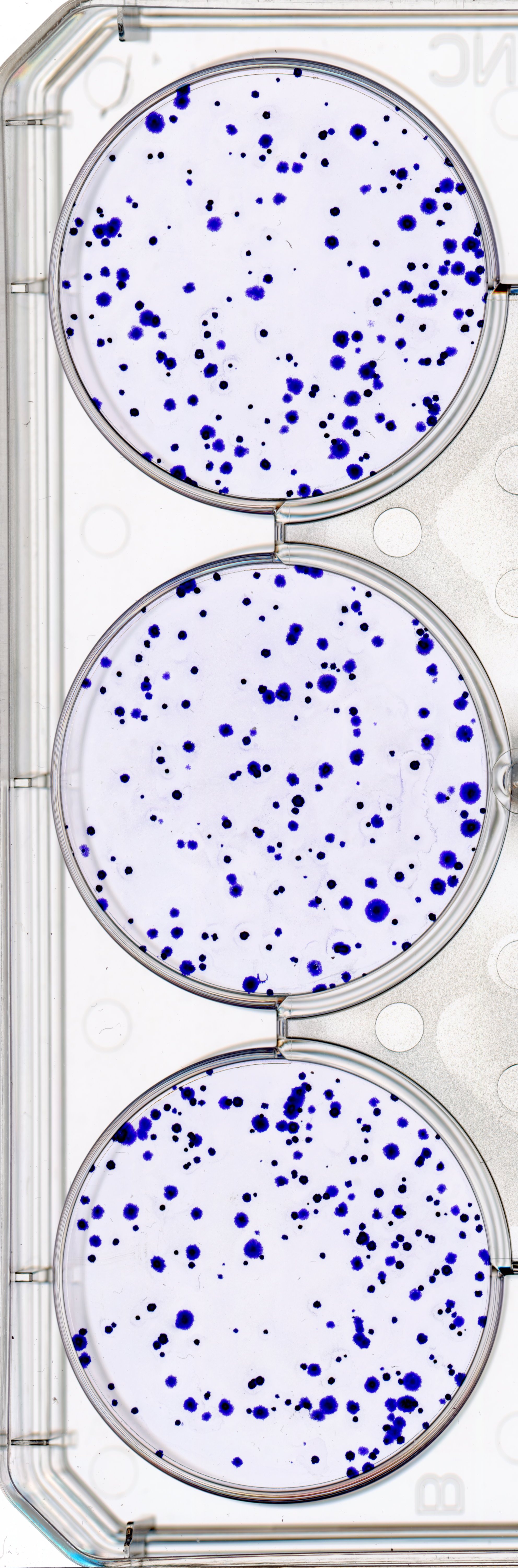

Supplement: Supplementary file 11 — Figure EV3 Source Data [file 44318_2024_108_MOESM11_ESM.zip › EMBOJ-2023-115654_FigEV3_sourcedata/EV3F/E230215 WT FA0.jpg]

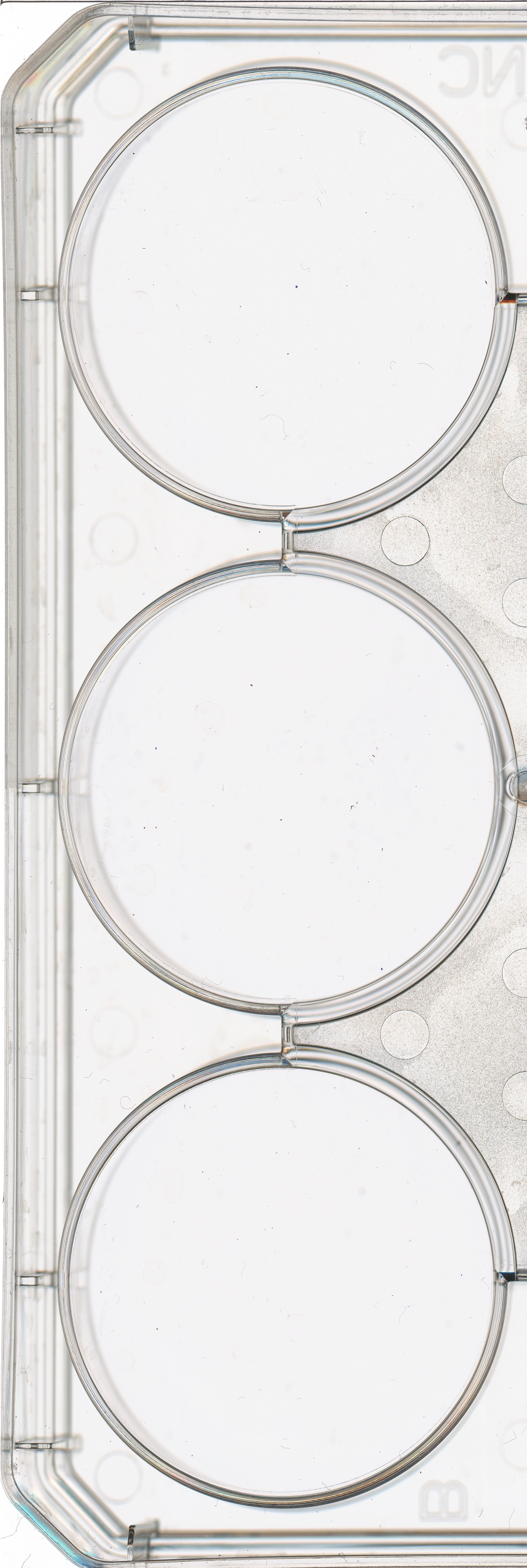

Supplement: Supplementary file 11 — Figure EV3 Source Data [file 44318_2024_108_MOESM11_ESM.zip › EMBOJ-2023-115654_FigEV3_sourcedata/EV3J/HAP1 WT IR4.jpg]

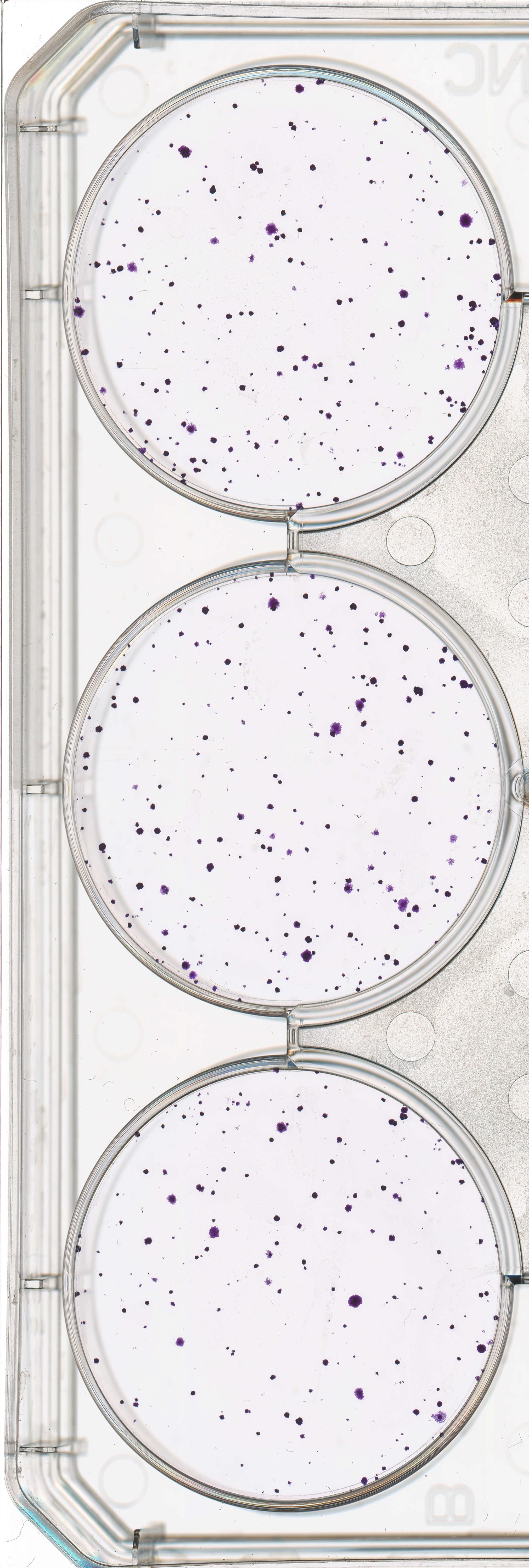

Supplement: Supplementary file 11 — Figure EV3 Source Data [file 44318_2024_108_MOESM11_ESM.zip › EMBOJ-2023-115654_FigEV3_sourcedata/EV3J/HAP1 WT IR1.jpg]

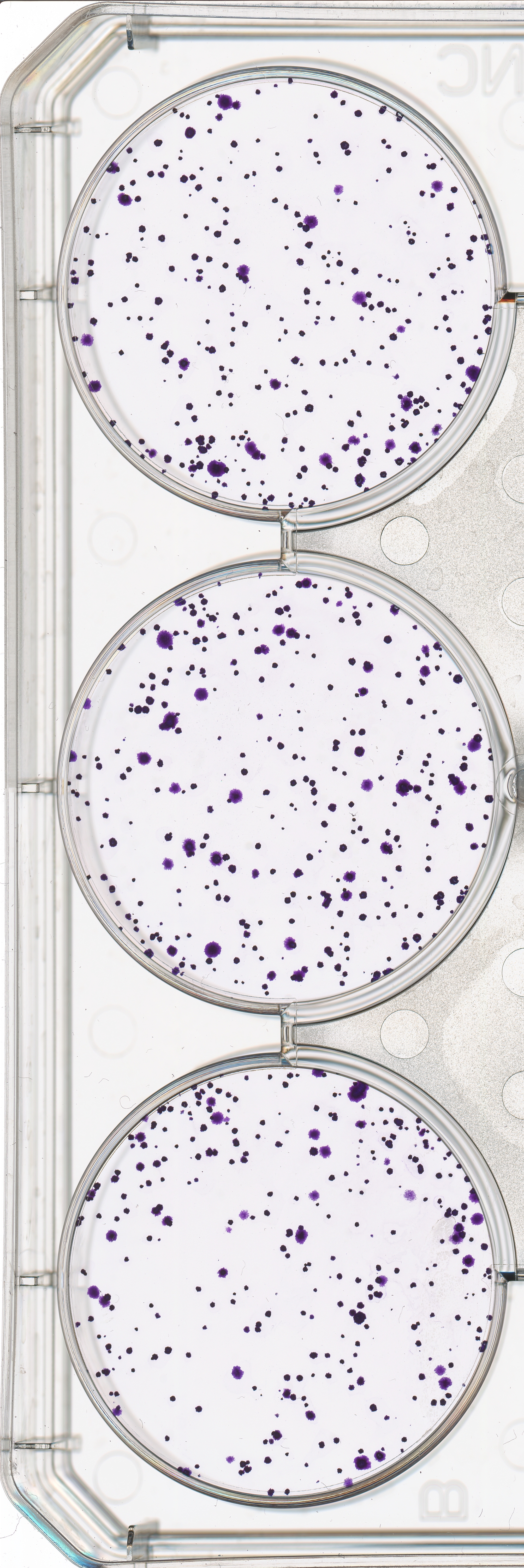

Supplement: Supplementary file 11 — Figure EV3 Source Data [file 44318_2024_108_MOESM11_ESM.zip › EMBOJ-2023-115654_FigEV3_sourcedata/EV3J/HAP1 WT IR0.jpg]

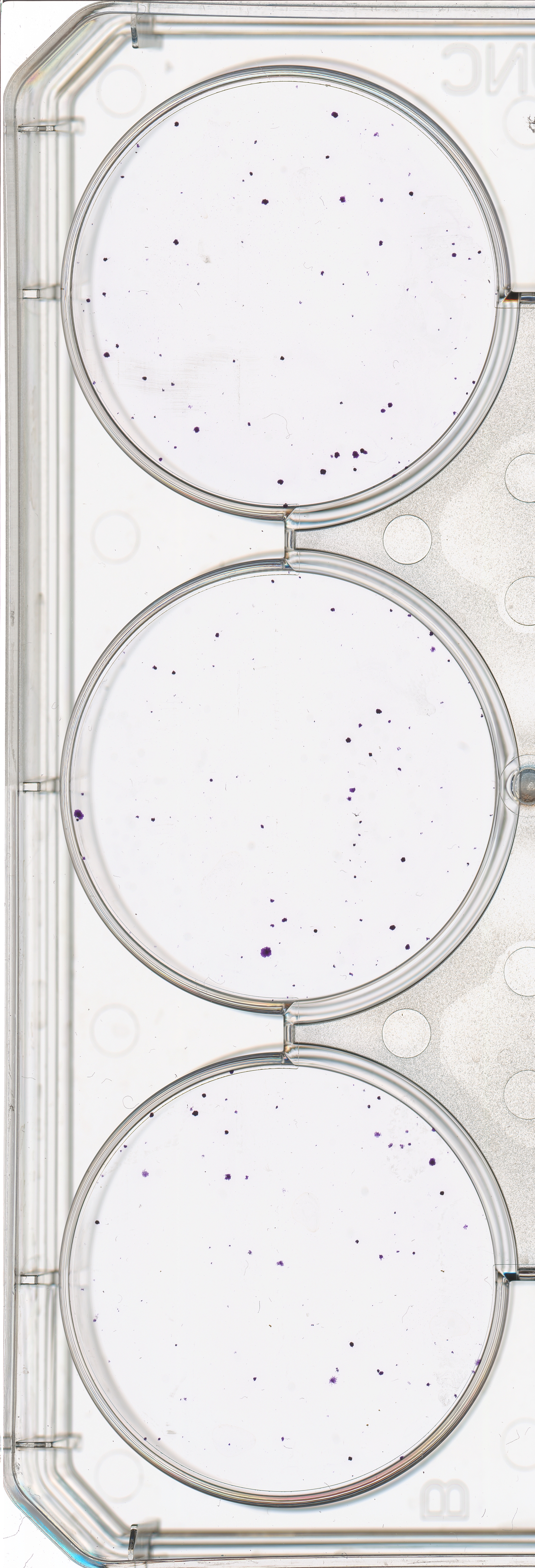

Supplement: Supplementary file 11 — Figure EV3 Source Data [file 44318_2024_108_MOESM11_ESM.zip › EMBOJ-2023-115654_FigEV3_sourcedata/EV3J/HAP1 WT IR2.jpg]

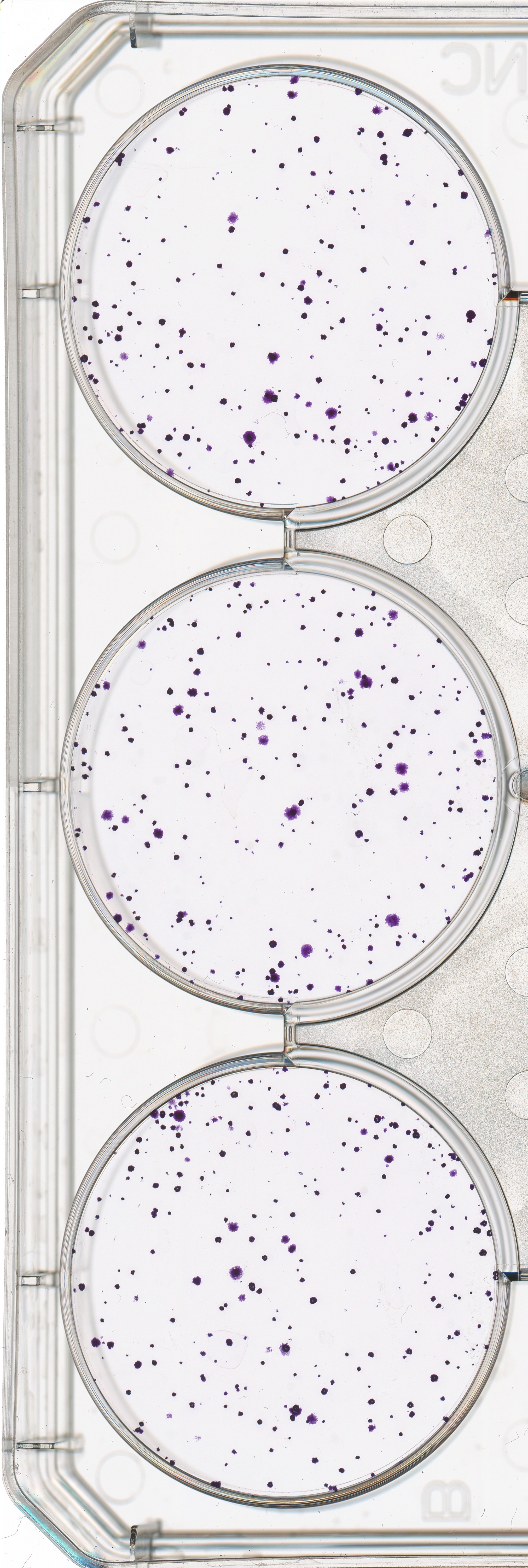

Supplement: Supplementary file 11 — Figure EV3 Source Data [file 44318_2024_108_MOESM11_ESM.zip › EMBOJ-2023-115654_FigEV3_sourcedata/EV3J/HAP1 WT IR0.5.jpg]

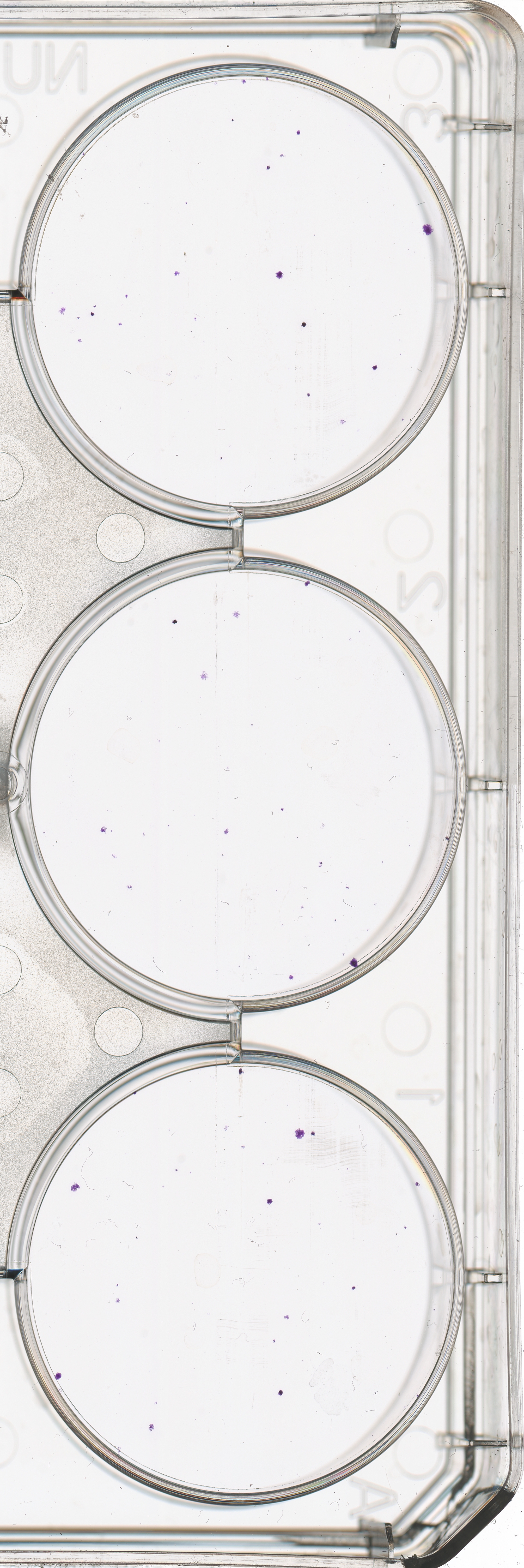

Supplement: Supplementary file 11 — Figure EV3 Source Data [file 44318_2024_108_MOESM11_ESM.zip › EMBOJ-2023-115654_FigEV3_sourcedata/EV3J/HAP1 TOPORSko IR2.jpg]

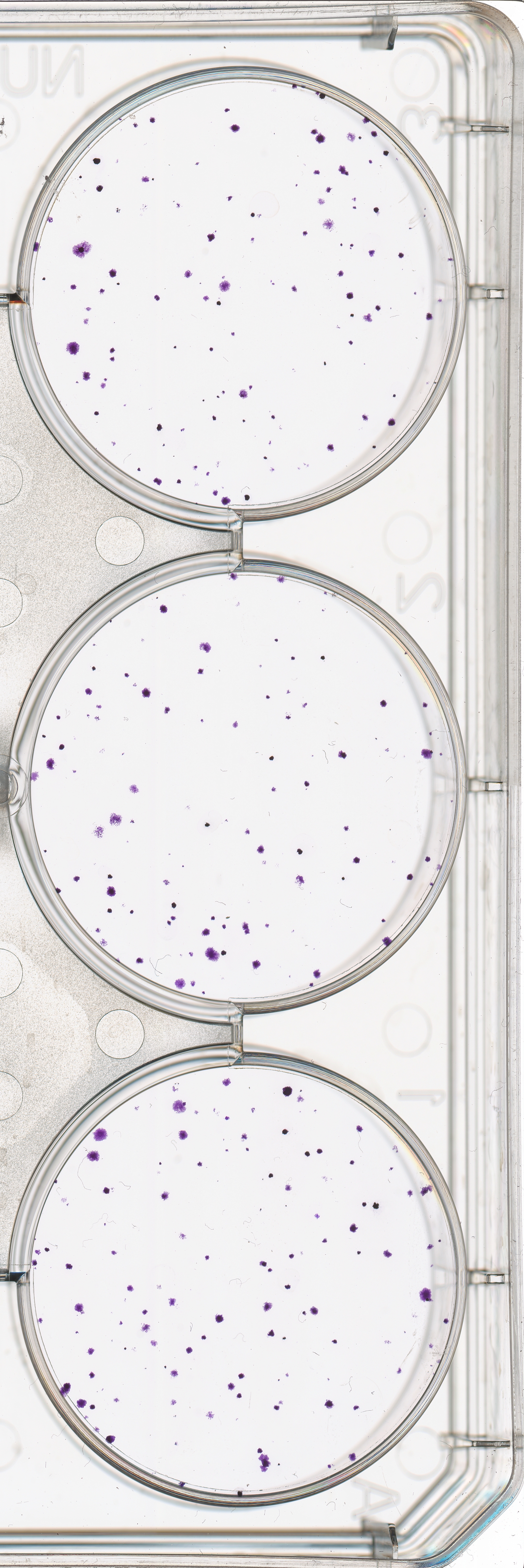

Supplement: Supplementary file 11 — Figure EV3 Source Data [file 44318_2024_108_MOESM11_ESM.zip › EMBOJ-2023-115654_FigEV3_sourcedata/EV3J/HAP1 TOPORSko IR1.jpg]

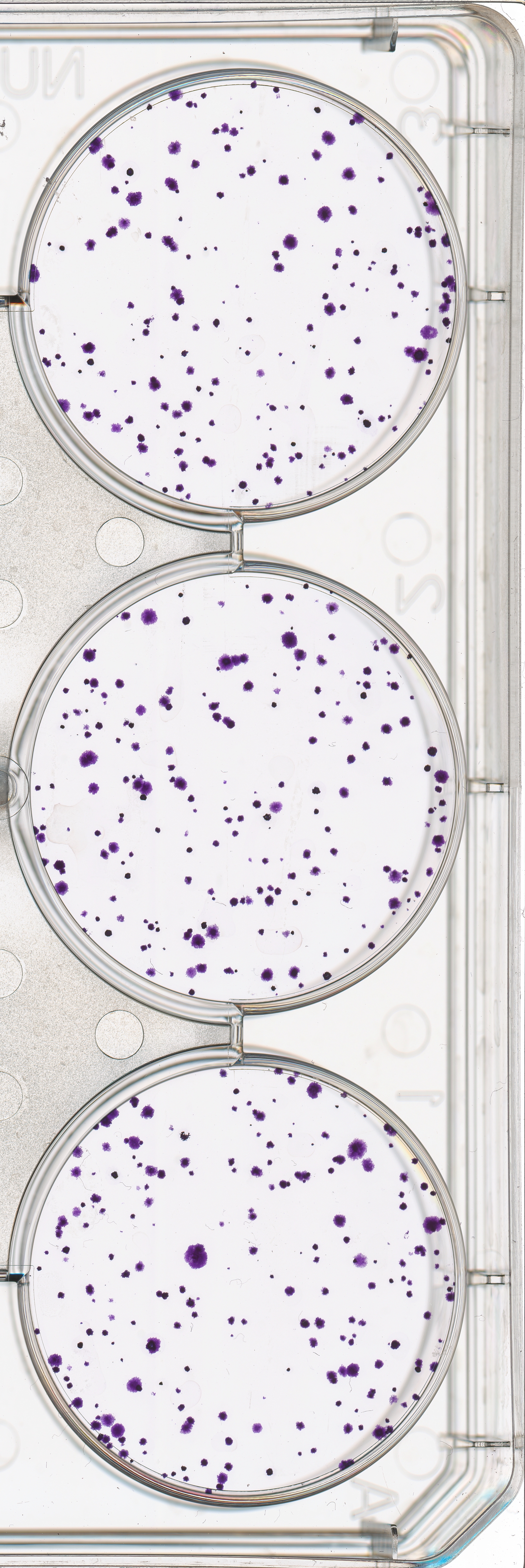

Supplement: Supplementary file 11 — Figure EV3 Source Data [file 44318_2024_108_MOESM11_ESM.zip › EMBOJ-2023-115654_FigEV3_sourcedata/EV3J/HAP1 TOPORSko IR0.jpg]

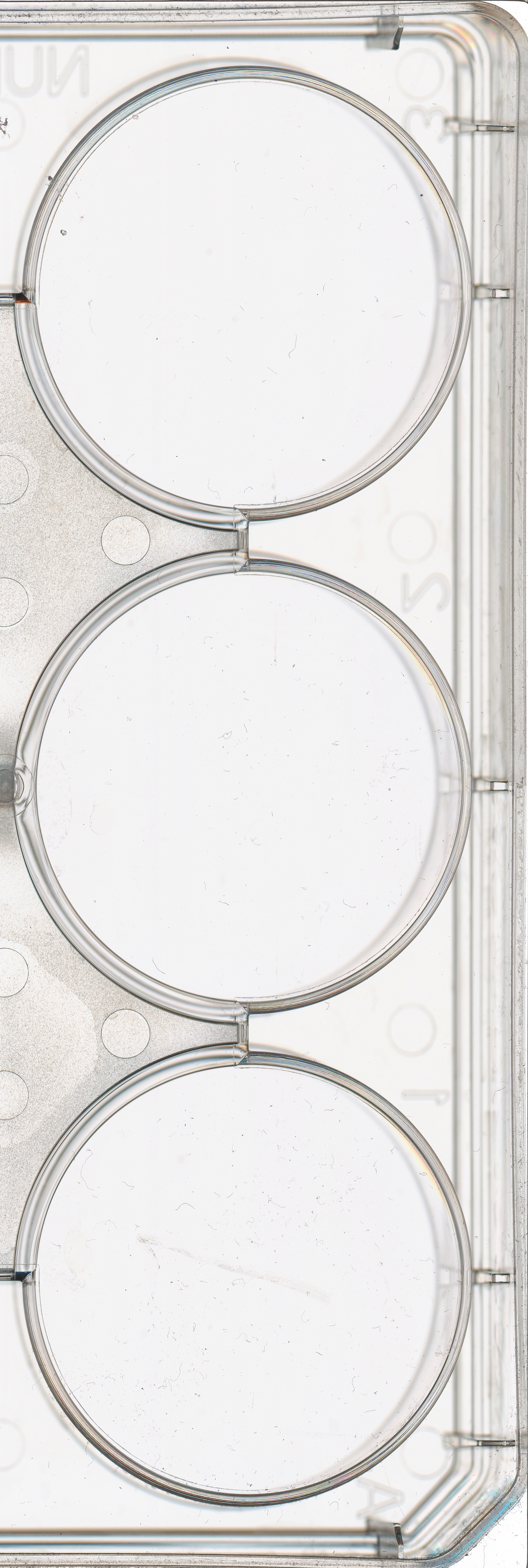

Supplement: Supplementary file 11 — Figure EV3 Source Data [file 44318_2024_108_MOESM11_ESM.zip › EMBOJ-2023-115654_FigEV3_sourcedata/EV3J/HAP1 TOPORSko IR4.jpg]

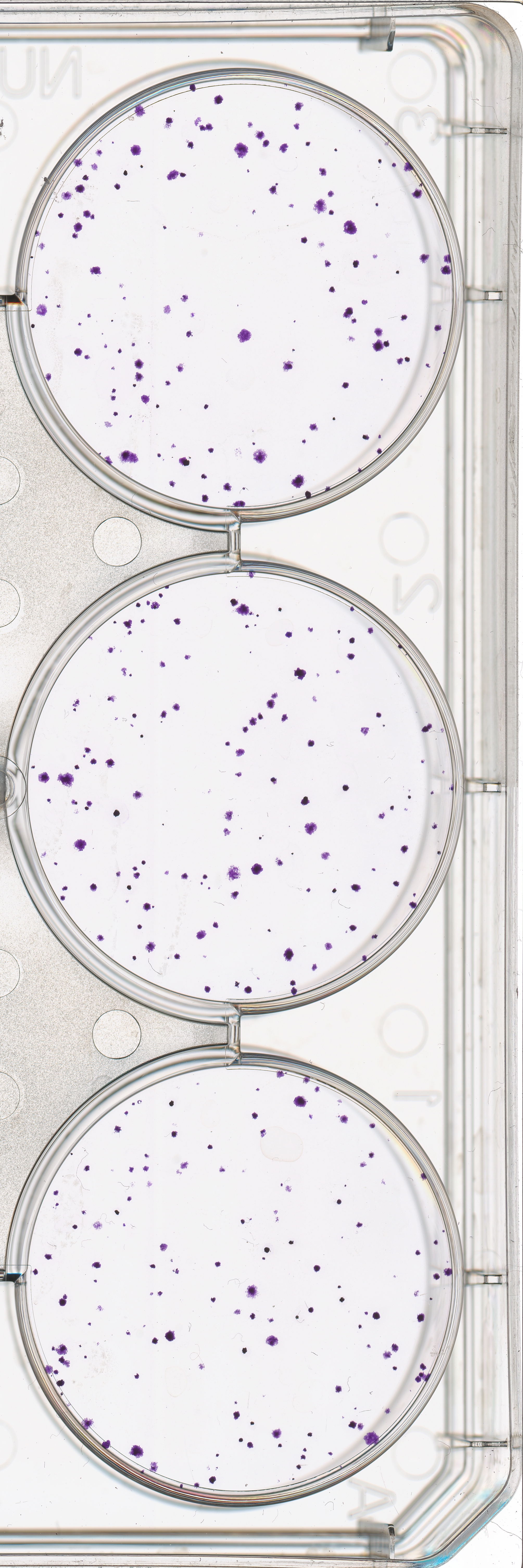

Supplement: Supplementary file 11 — Figure EV3 Source Data [file 44318_2024_108_MOESM11_ESM.zip › EMBOJ-2023-115654_FigEV3_sourcedata/EV3J/HAP1 TOPORSko IR0.5.jpg]

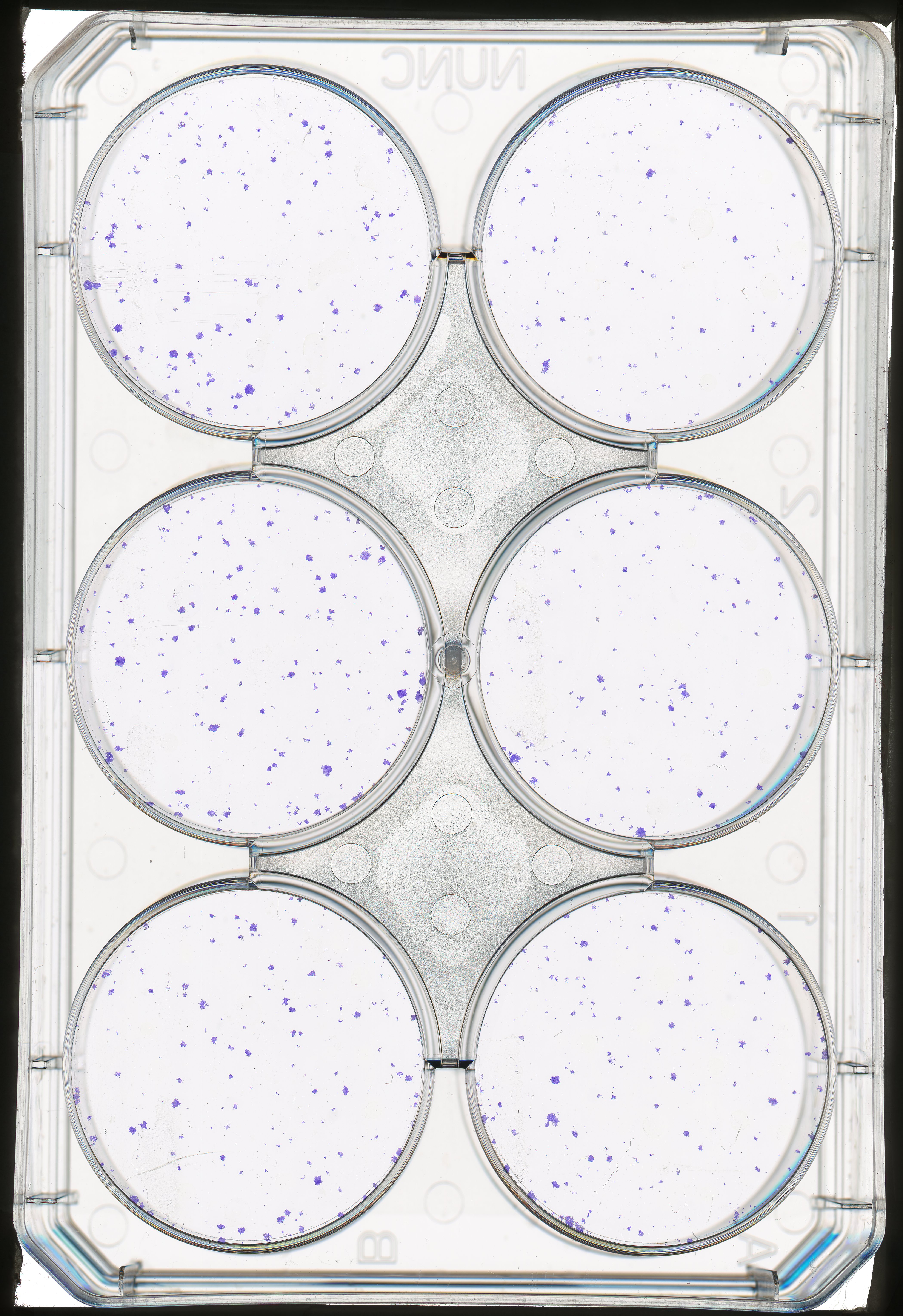

Supplement: Supplementary file 11 — Figure EV3 Source Data [file 44318_2024_108_MOESM11_ESM.zip › EMBOJ-2023-115654_FigEV3_sourcedata/EV3D/U2OS siCtrl 5dC30-60.jpg]

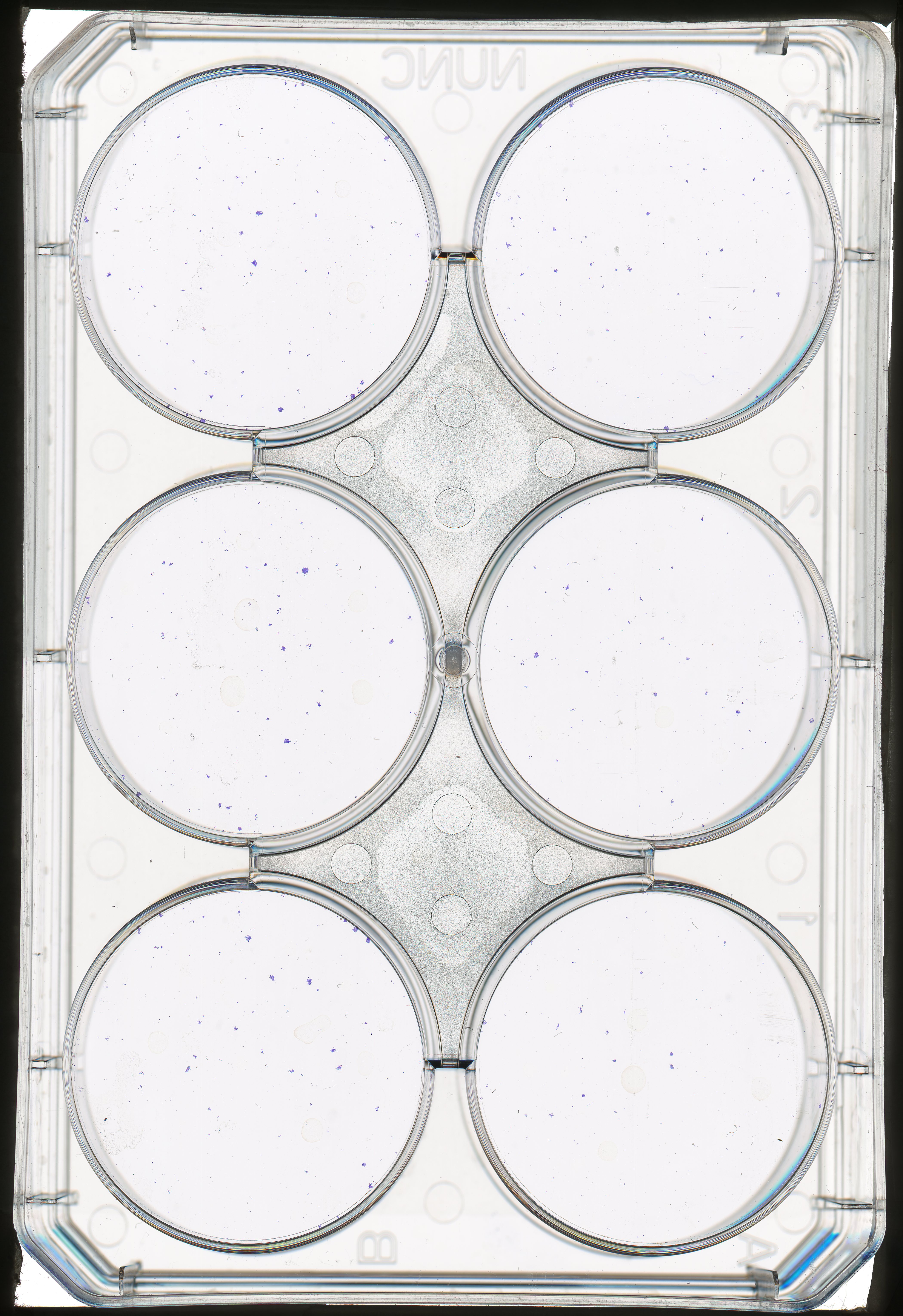

Supplement: Supplementary file 11 — Figure EV3 Source Data [file 44318_2024_108_MOESM11_ESM.zip › EMBOJ-2023-115654_FigEV3_sourcedata/EV3D/U2OS siTOPORS 5dC90-120.jpg]

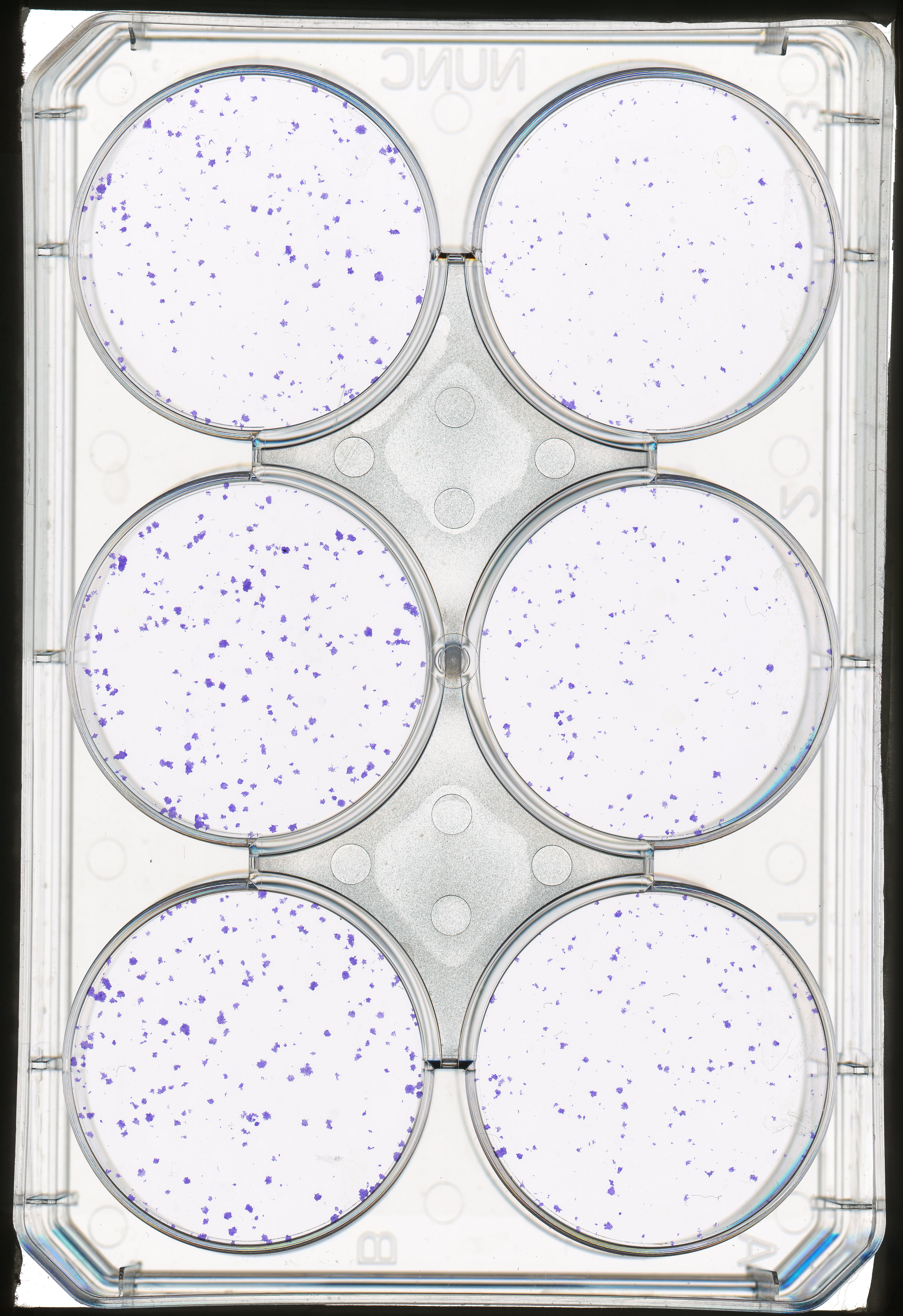

Supplement: Supplementary file 11 — Figure EV3 Source Data [file 44318_2024_108_MOESM11_ESM.zip › EMBOJ-2023-115654_FigEV3_sourcedata/EV3D/U2OS siTOPORS 5dC0-10.jpg]

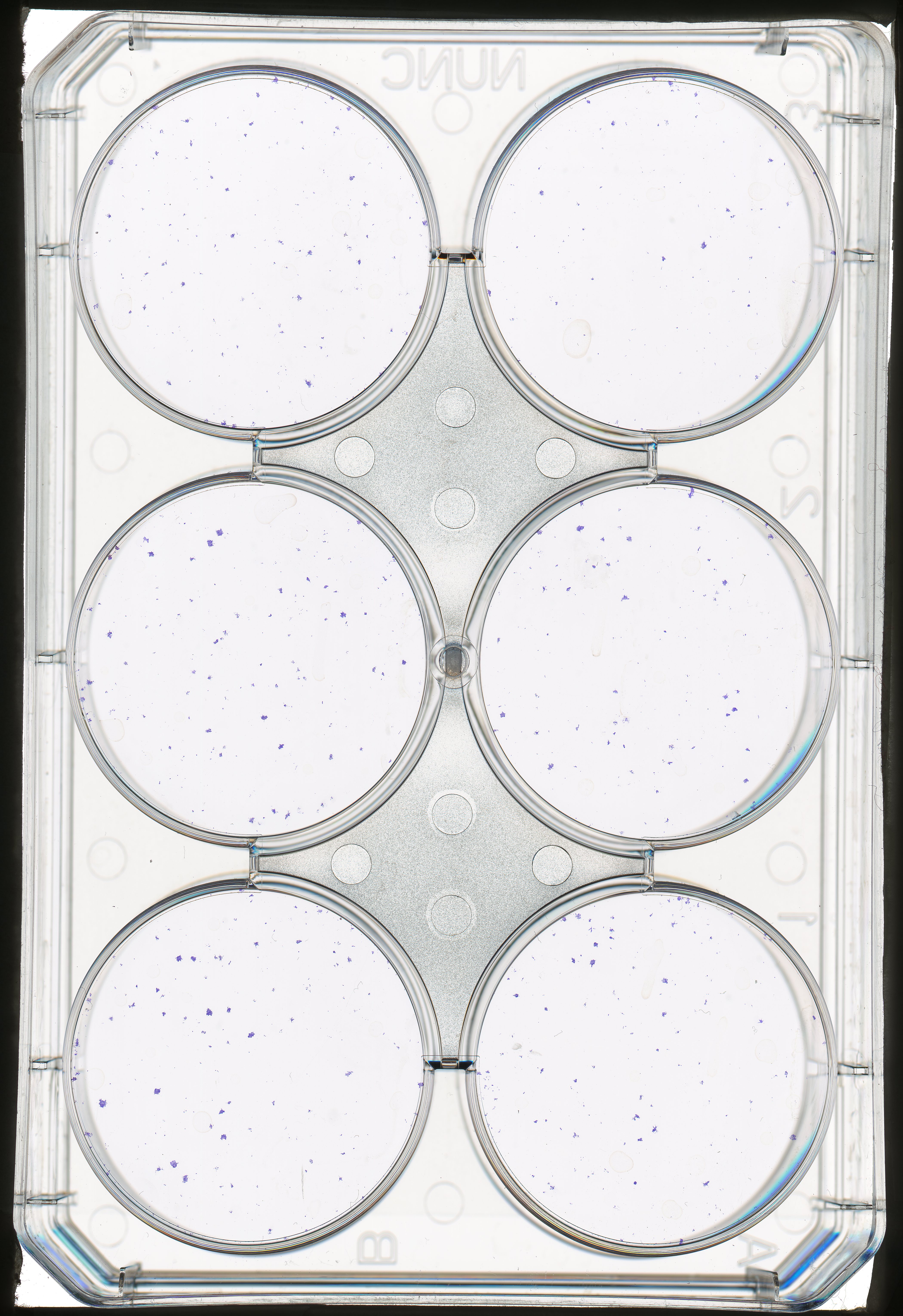

Supplement: Supplementary file 11 — Figure EV3 Source Data [file 44318_2024_108_MOESM11_ESM.zip › EMBOJ-2023-115654_FigEV3_sourcedata/EV3D/U2OS siTOPORS 5dC30-60.jpg]

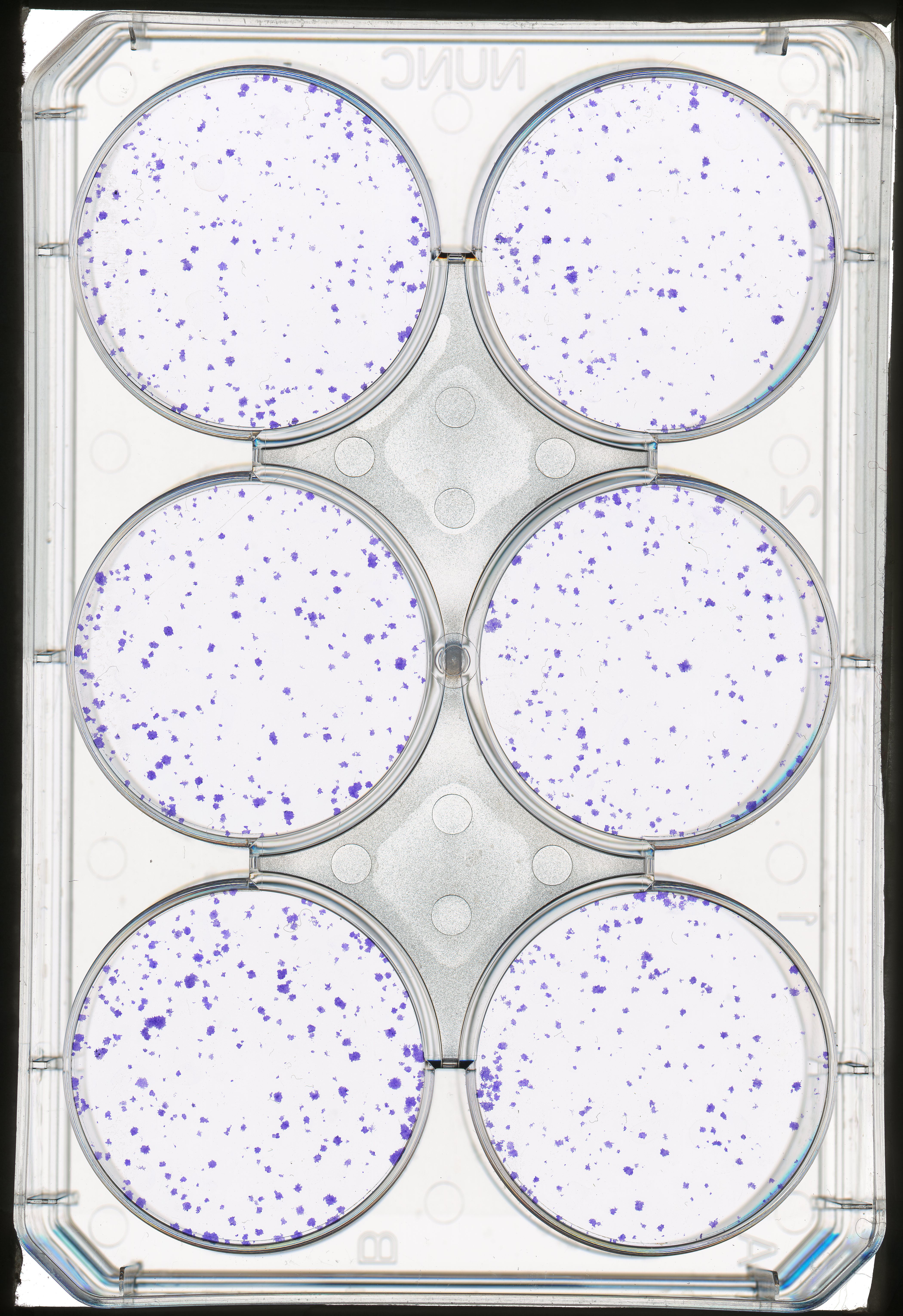

Supplement: Supplementary file 11 — Figure EV3 Source Data [file 44318_2024_108_MOESM11_ESM.zip › EMBOJ-2023-115654_FigEV3_sourcedata/EV3D/U2OS siCtrl 5dC0-10.jpg]

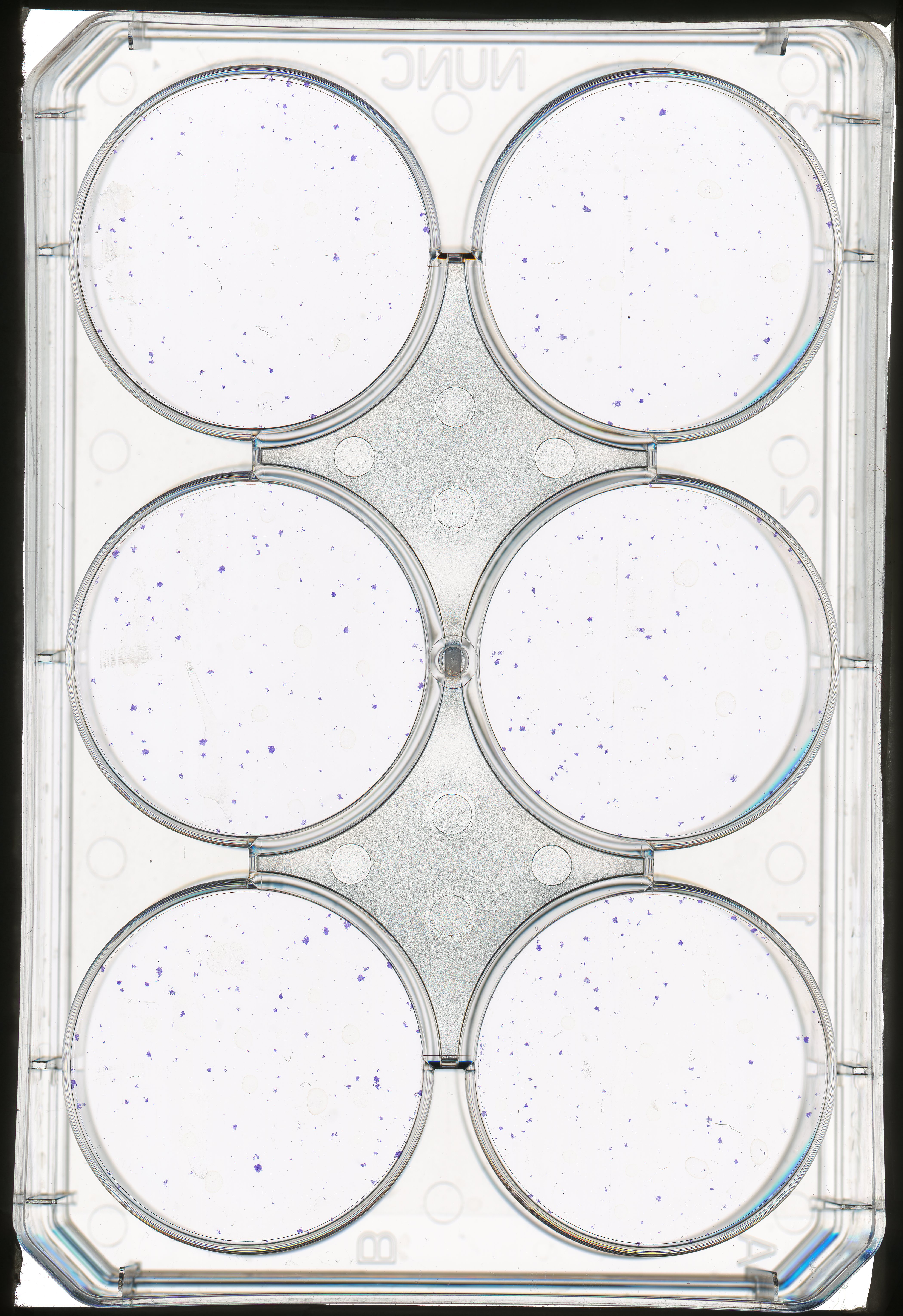

Supplement: Supplementary file 11 — Figure EV3 Source Data [file 44318_2024_108_MOESM11_ESM.zip › EMBOJ-2023-115654_FigEV3_sourcedata/EV3D/U2OS siCtrl 5dC90-120.jpg]

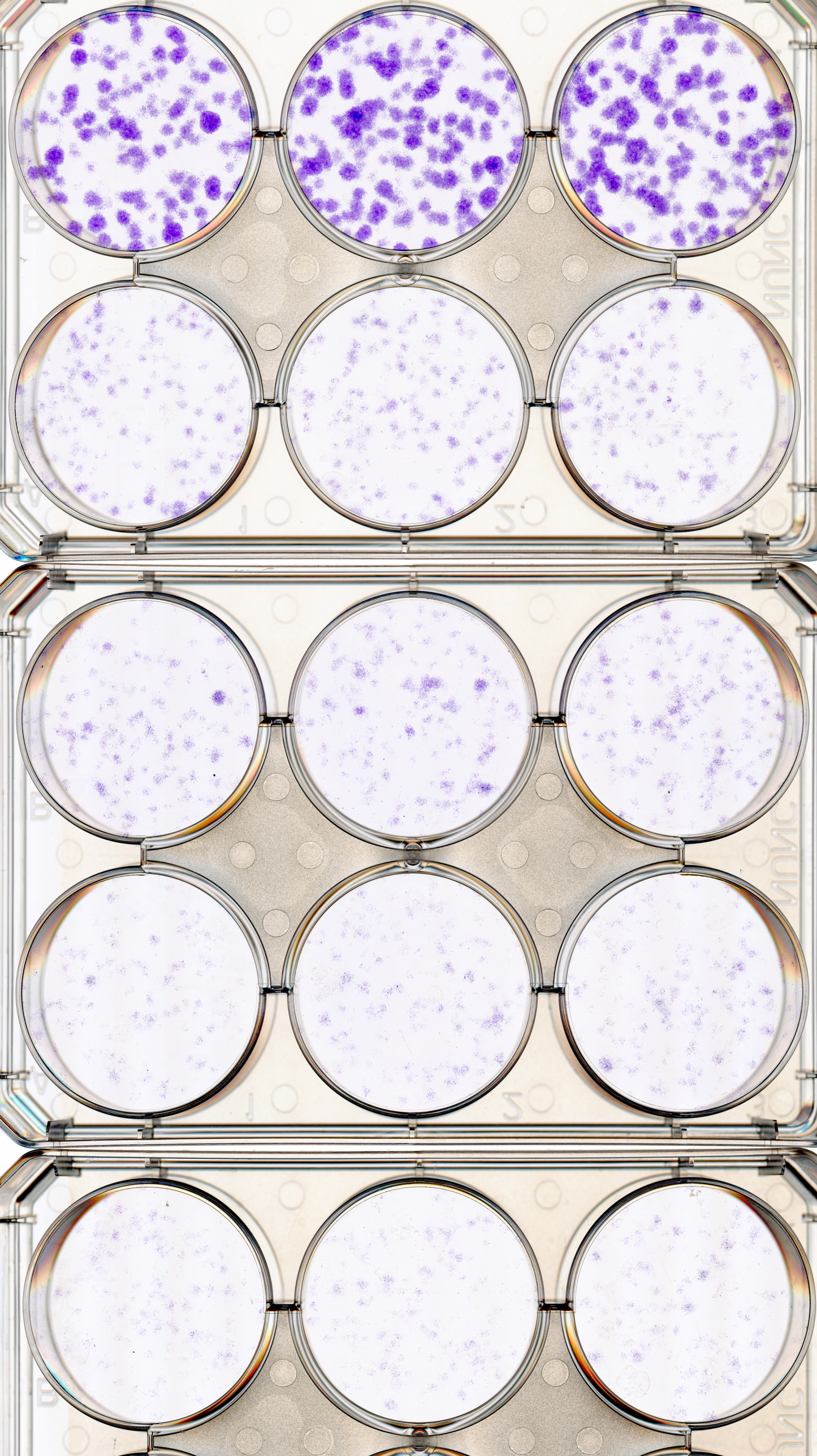

Supplement: Supplementary file 11 — Figure EV3 Source Data [file 44318_2024_108_MOESM11_ESM.zip › EMBOJ-2023-115654_FigEV3_sourcedata/EV3B/TP53-TOPORSdko1 5dC0-300nM.jpg]

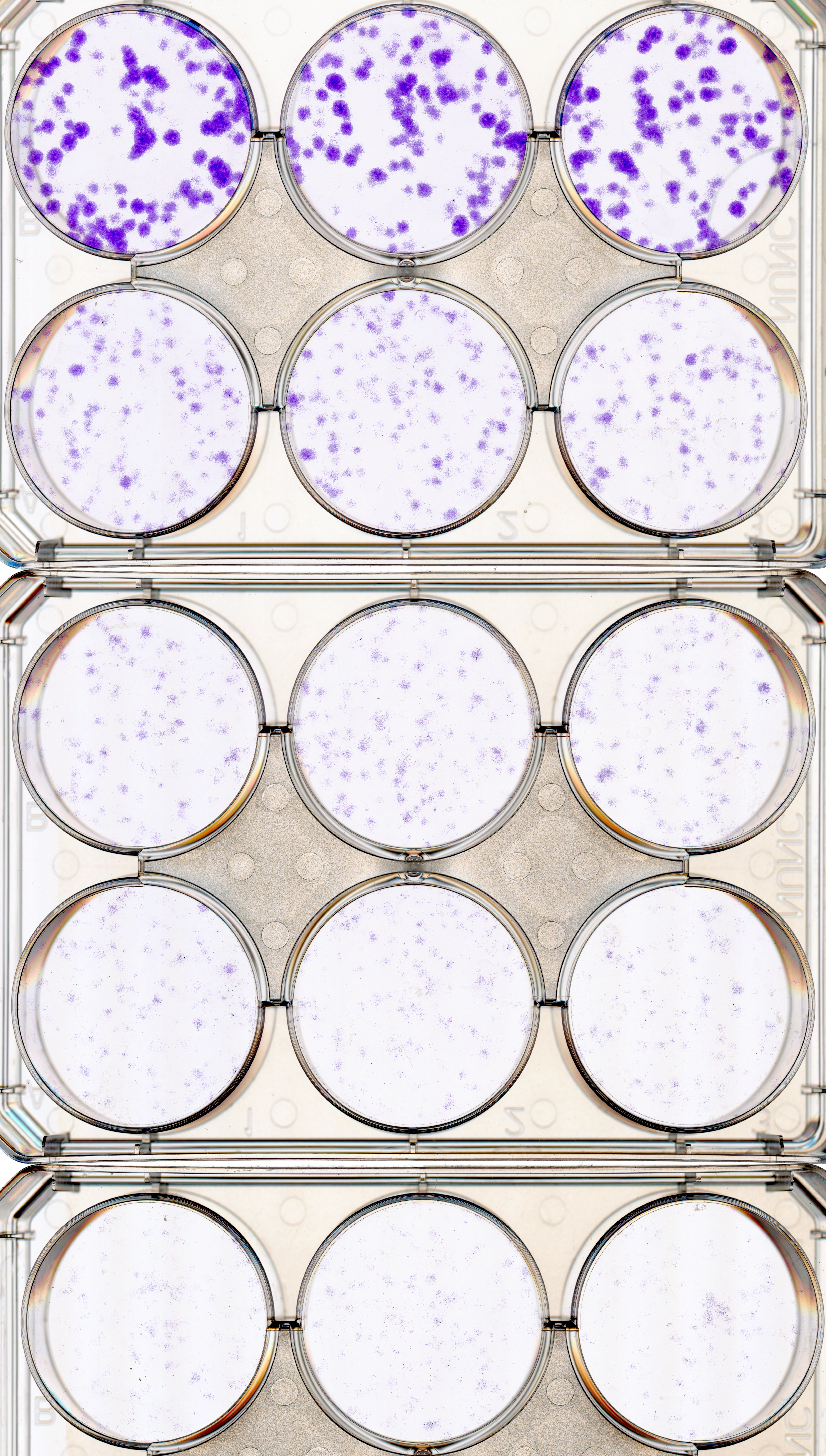

Supplement: Supplementary file 11 — Figure EV3 Source Data [file 44318_2024_108_MOESM11_ESM.zip › EMBOJ-2023-115654_FigEV3_sourcedata/EV3B/TP53-TOPORSdko6 5dC0-300nM.jpg]

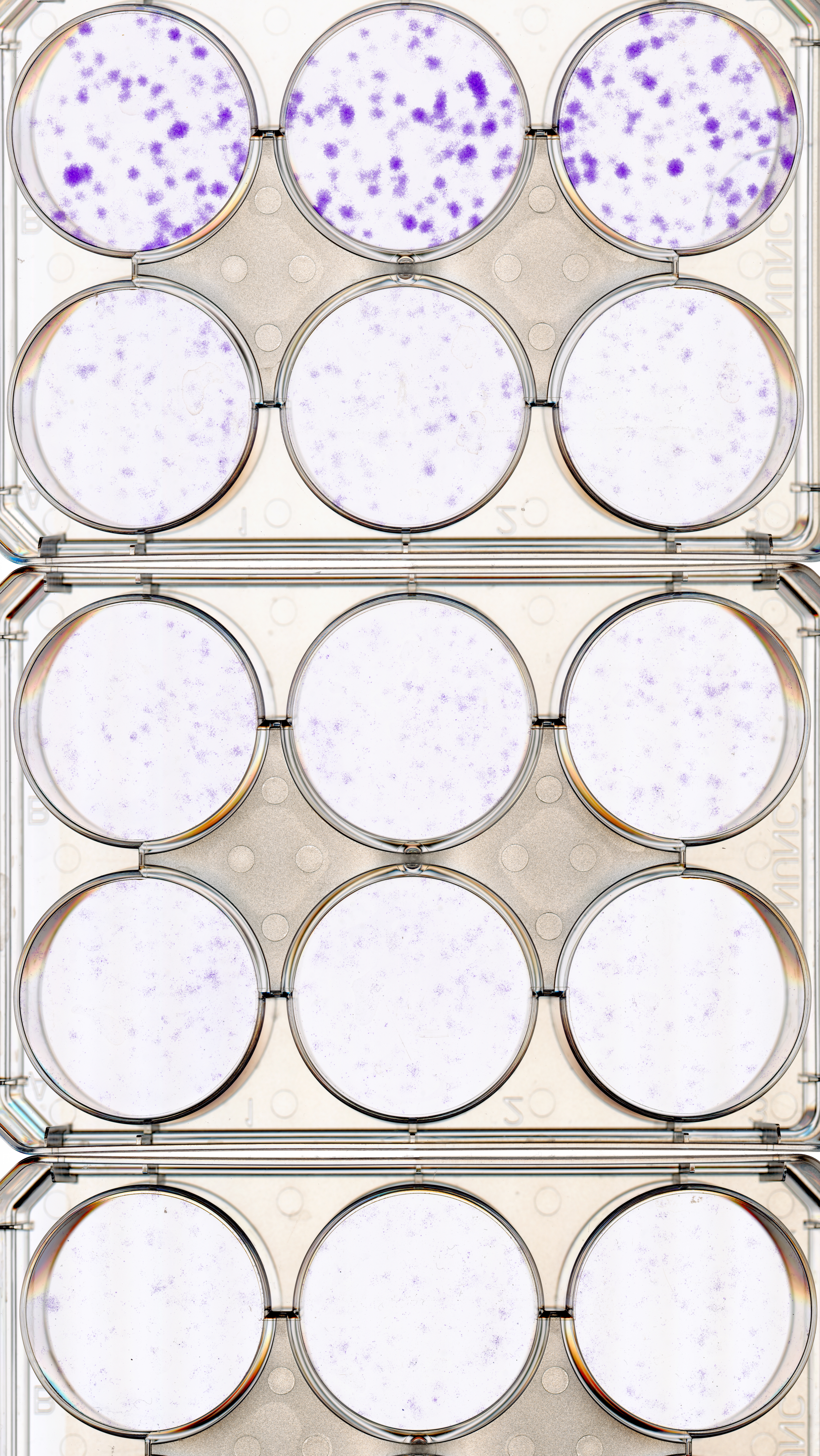

Supplement: Supplementary file 11 — Figure EV3 Source Data [file 44318_2024_108_MOESM11_ESM.zip › EMBOJ-2023-115654_FigEV3_sourcedata/EV3B/TP53-TOPORSdko3 5dC0-300nM.jpg]

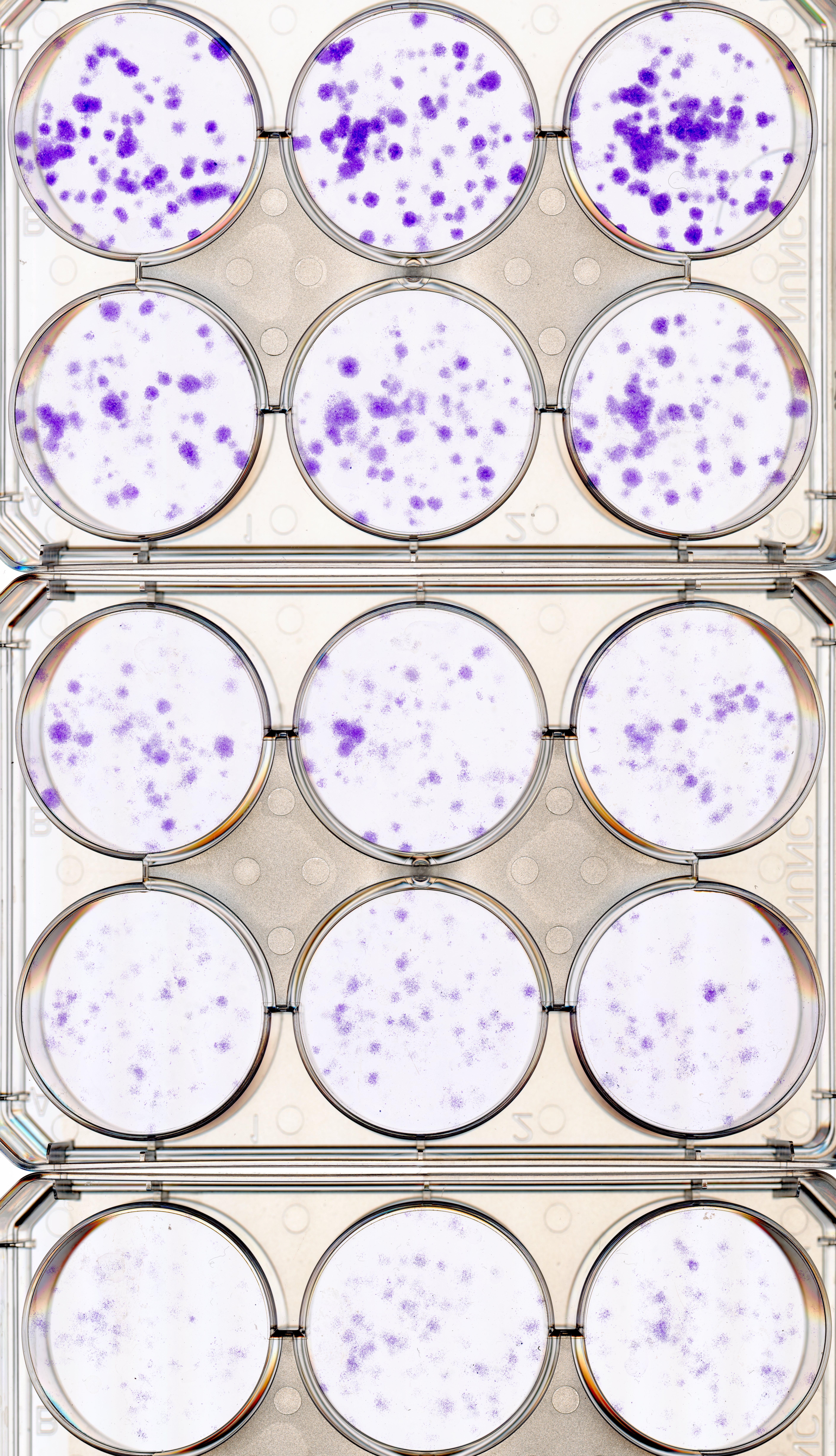

Supplement: Supplementary file 11 — Figure EV3 Source Data [file 44318_2024_108_MOESM11_ESM.zip › EMBOJ-2023-115654_FigEV3_sourcedata/EV3B/TP53ko 5dC0-300nM.jpg]

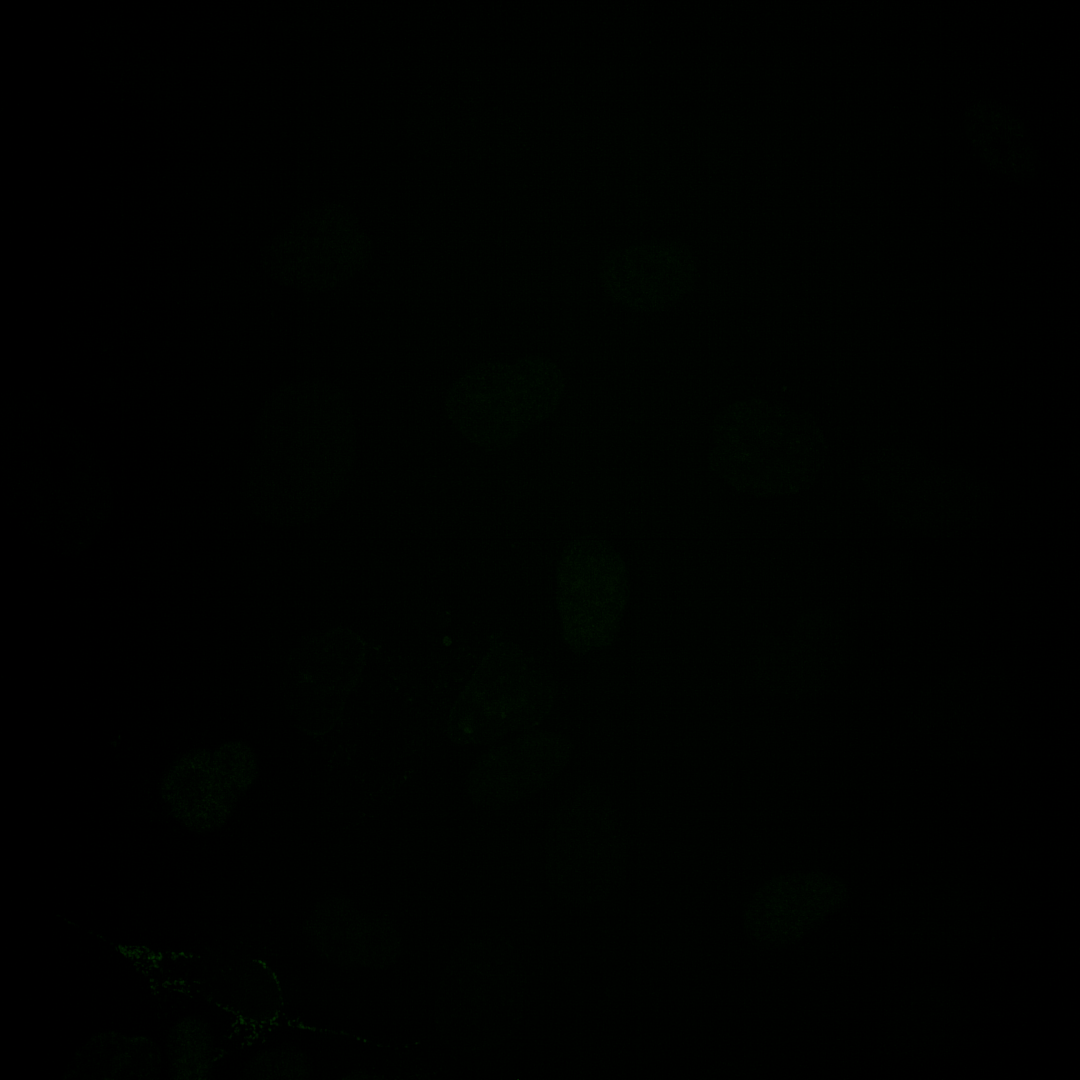

Supplement: Supplementary file 12 — Figure EV4 Source Data [file 44318_2024_108_MOESM12_ESM.zip › EMBOJ-2023-115654_FigEV4_sourcedata/EV4F/E240103 EV dC PLA GFP.png]

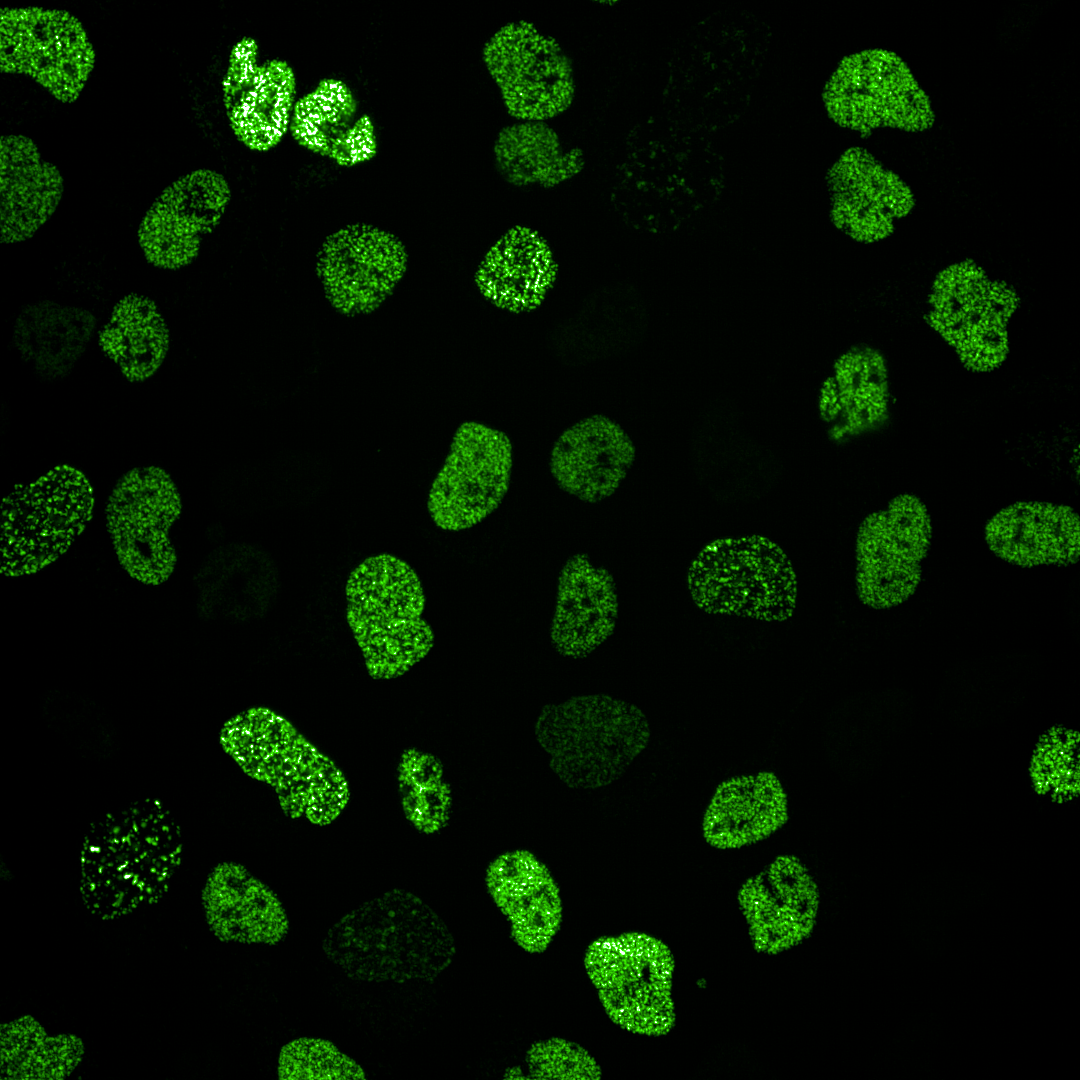

Supplement: Supplementary file 12 — Figure EV4 Source Data [file 44318_2024_108_MOESM12_ESM.zip › EMBOJ-2023-115654_FigEV4_sourcedata/EV4F/E240103 EV 5dC PLA GFP.png]

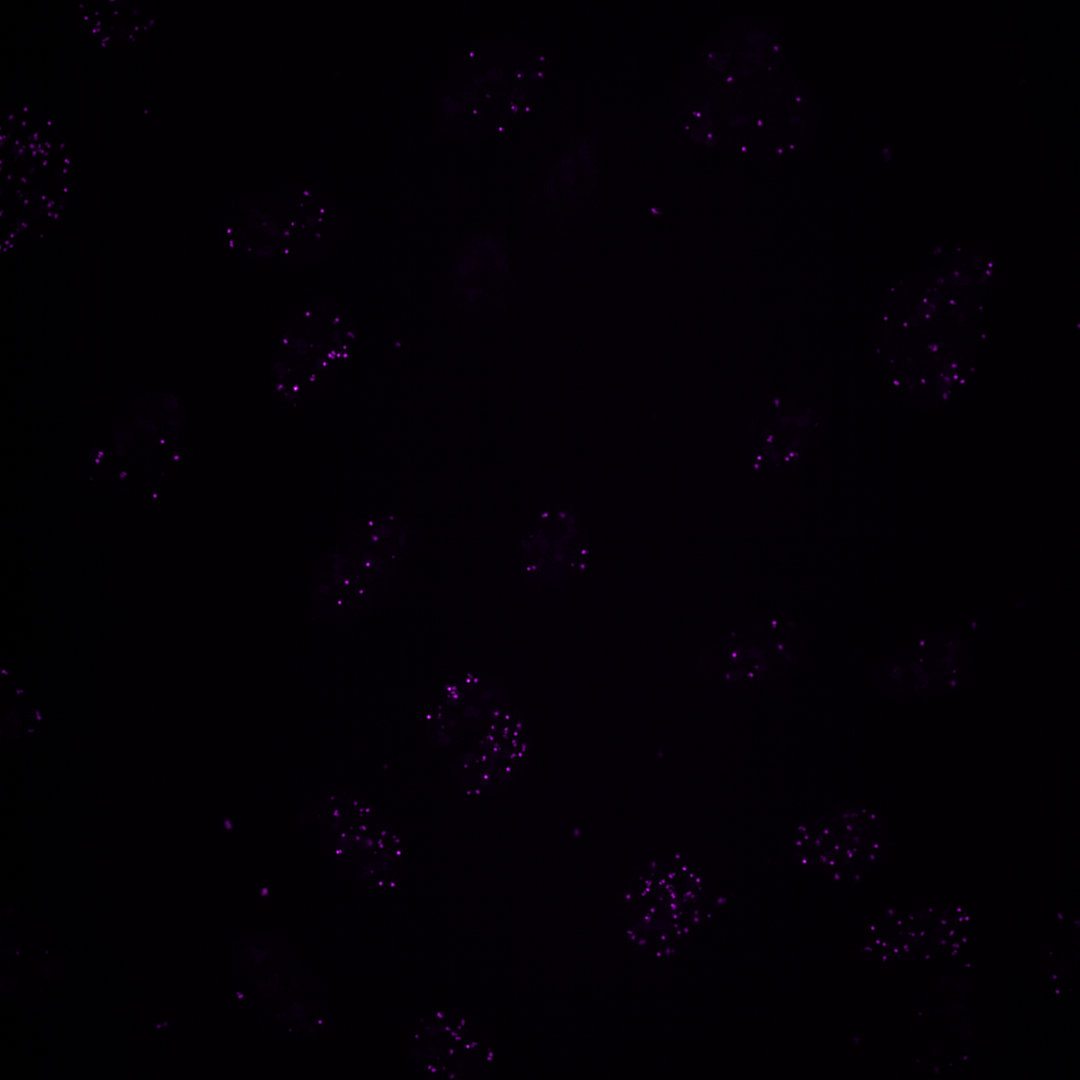

Supplement: Supplementary file 12 — Figure EV4 Source Data [file 44318_2024_108_MOESM12_ESM.zip › EMBOJ-2023-115654_FigEV4_sourcedata/EV4F/E240103 WT 5dC HAonly PLA.png]

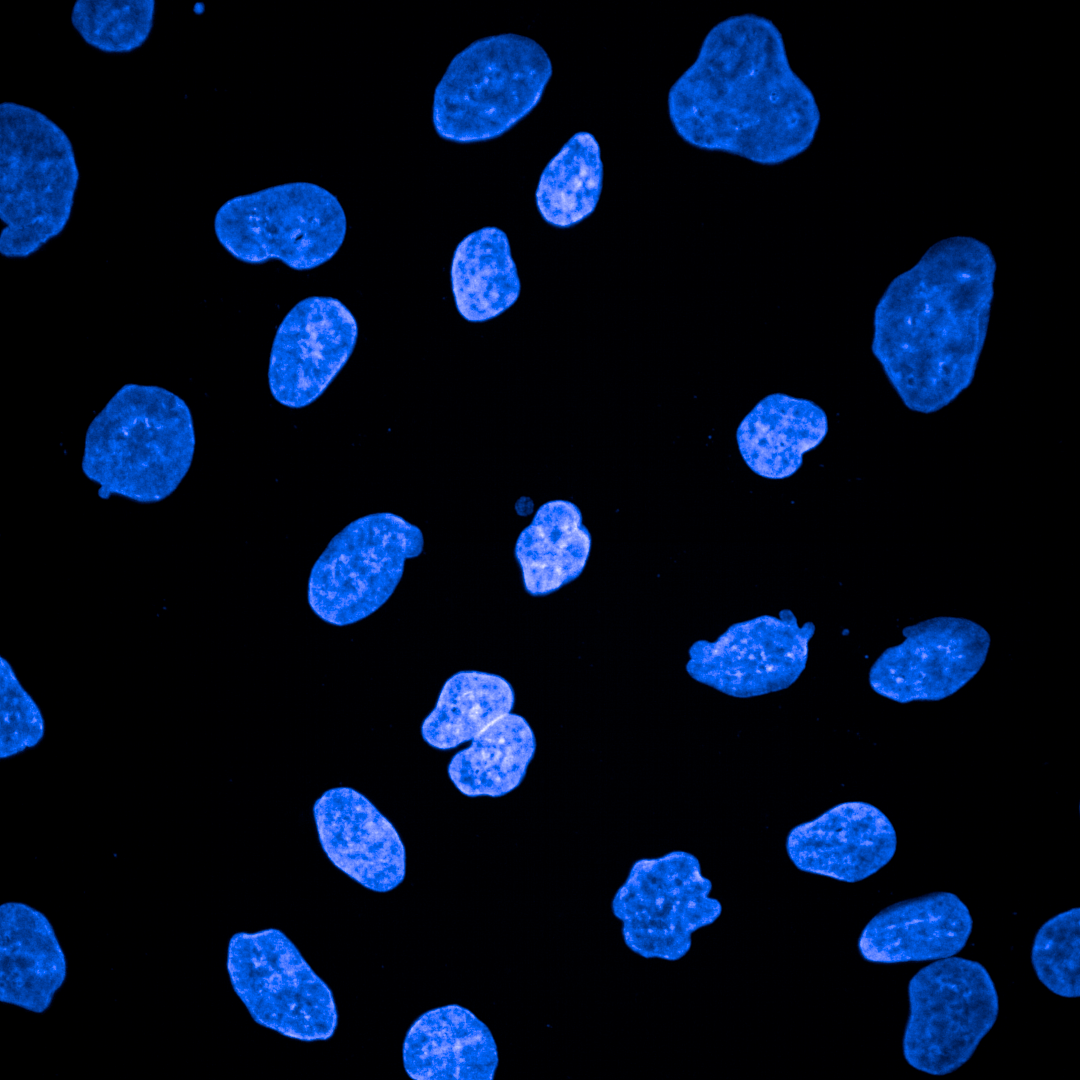

Supplement: Supplementary file 12 — Figure EV4 Source Data [file 44318_2024_108_MOESM12_ESM.zip › EMBOJ-2023-115654_FigEV4_sourcedata/EV4F/E240103 WT 5dC HAonly DAPI.png]

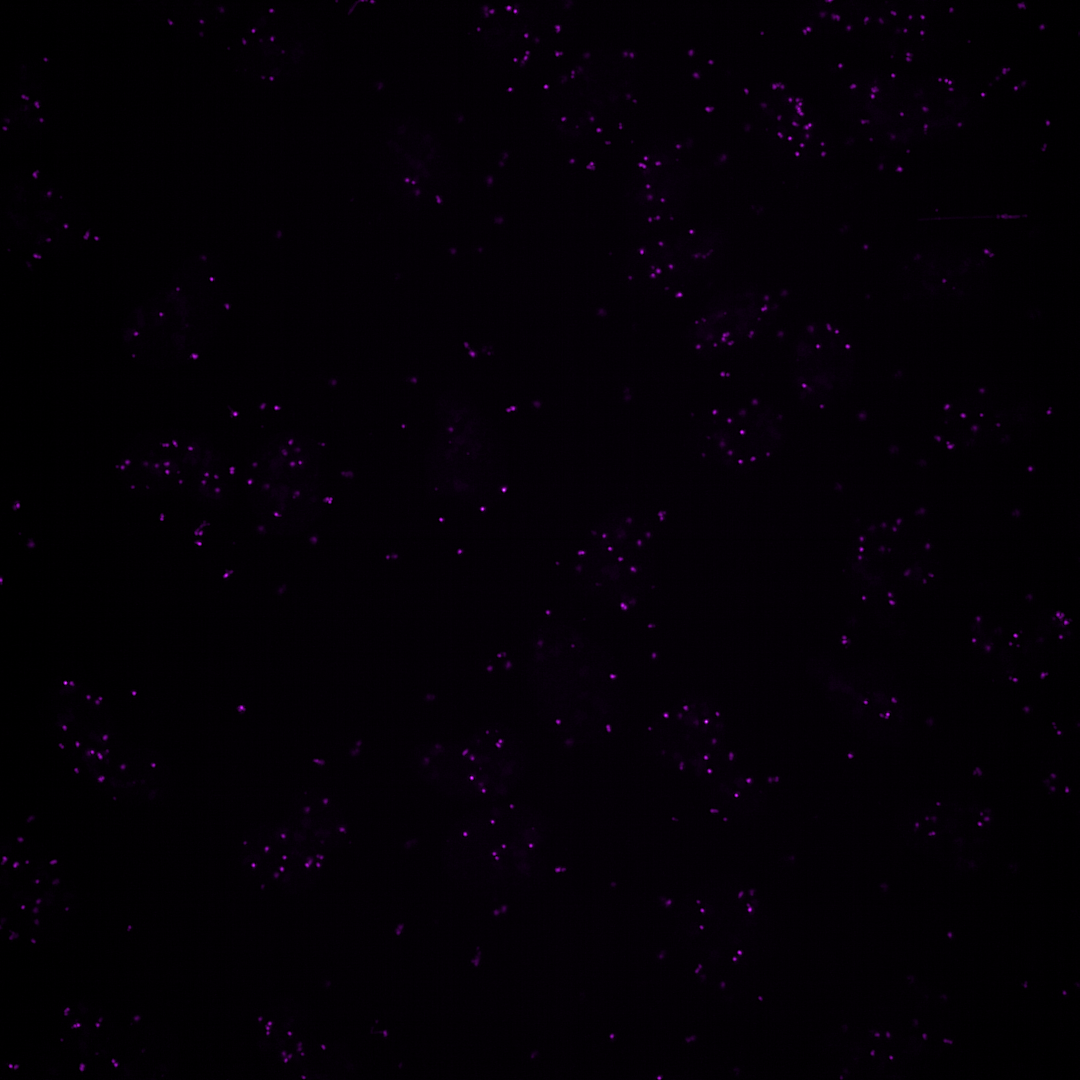

Supplement: Supplementary file 12 — Figure EV4 Source Data [file 44318_2024_108_MOESM12_ESM.zip › EMBOJ-2023-115654_FigEV4_sourcedata/EV4F/E240103 WT 5dC GFPonly PLA.png]

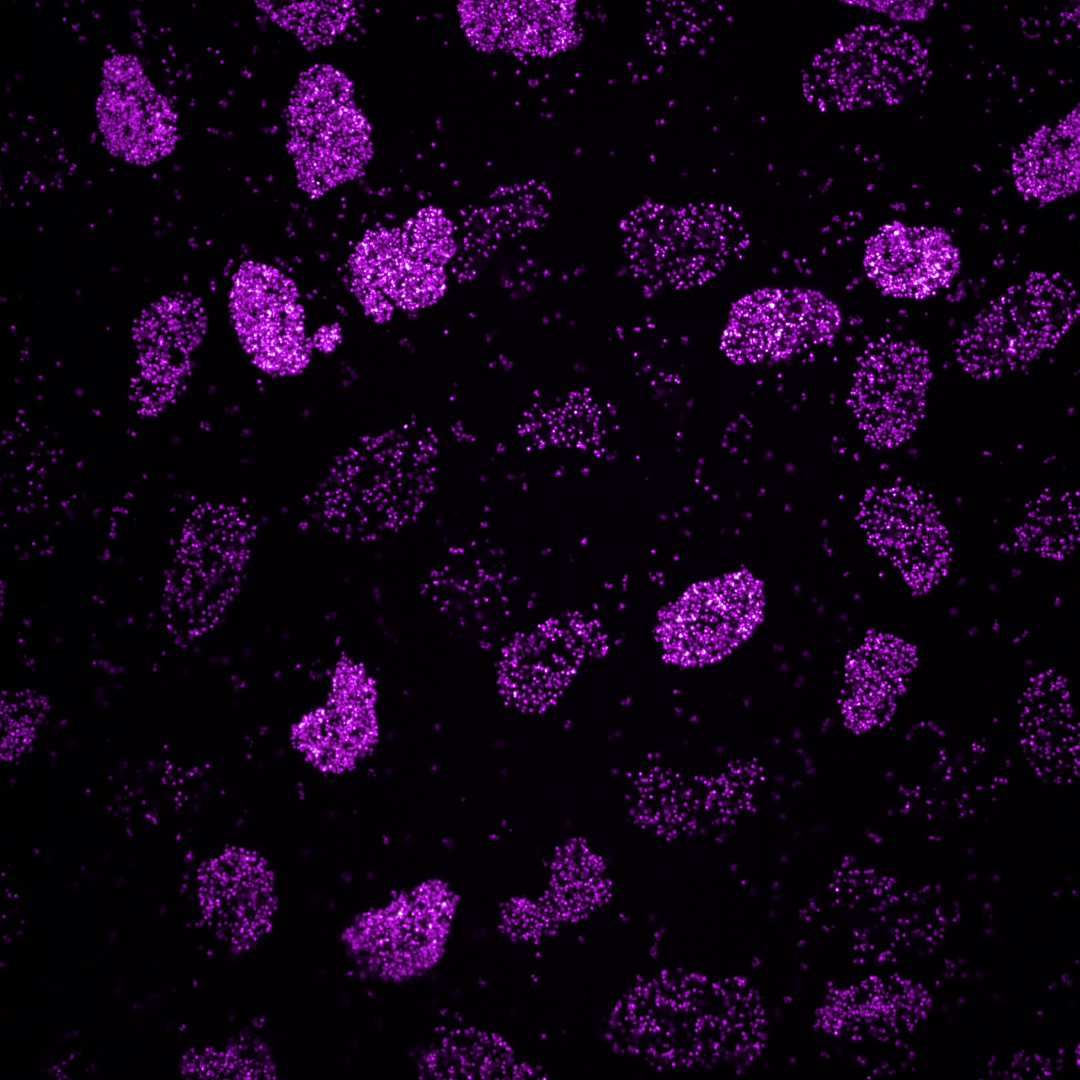

Supplement: Supplementary file 12 — Figure EV4 Source Data [file 44318_2024_108_MOESM12_ESM.zip › EMBOJ-2023-115654_FigEV4_sourcedata/EV4F/E240103 WT 5dC PLA PLA 2.png]

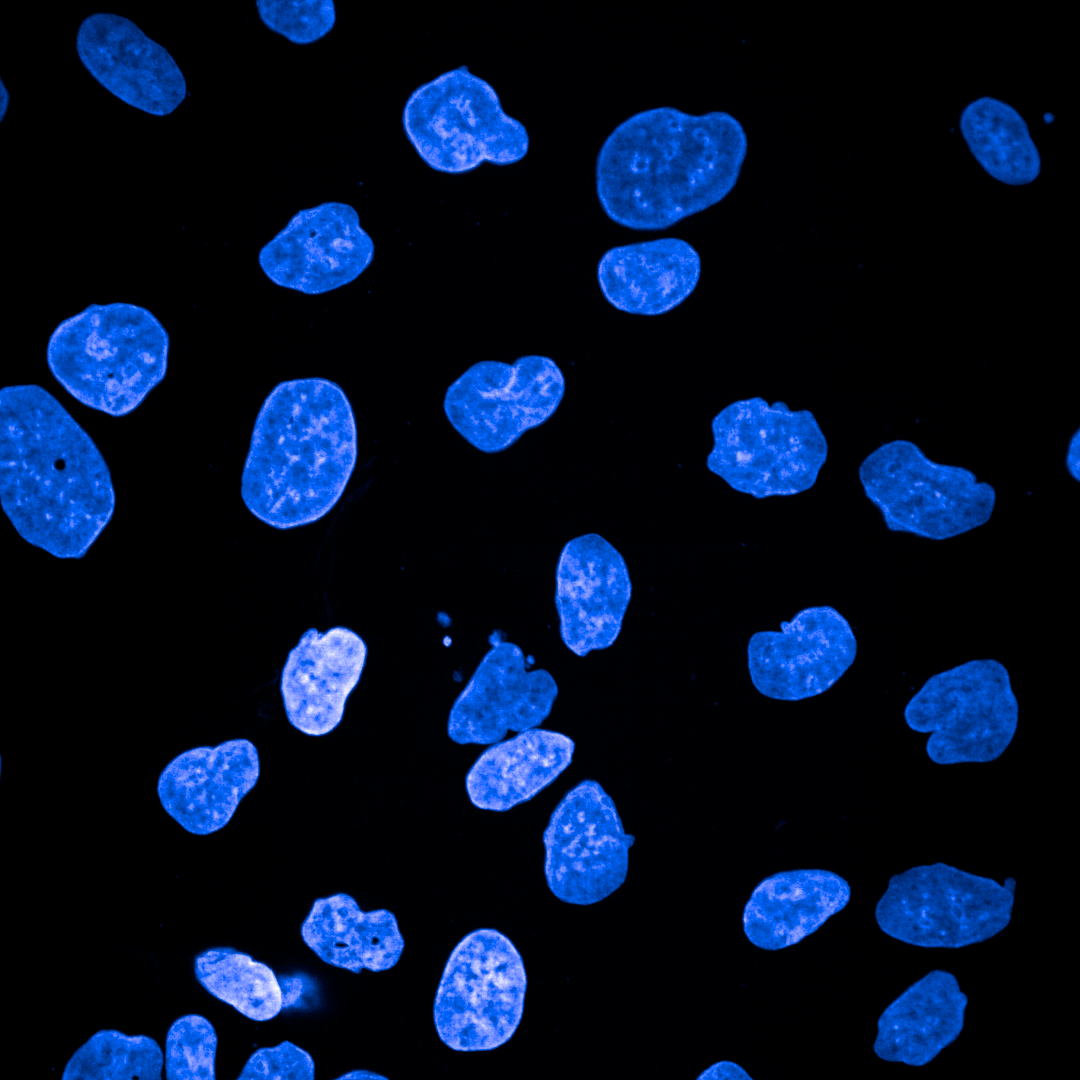

Supplement: Supplementary file 12 — Figure EV4 Source Data [file 44318_2024_108_MOESM12_ESM.zip › EMBOJ-2023-115654_FigEV4_sourcedata/EV4F/E240103 EV dC PLA DAPI.png]

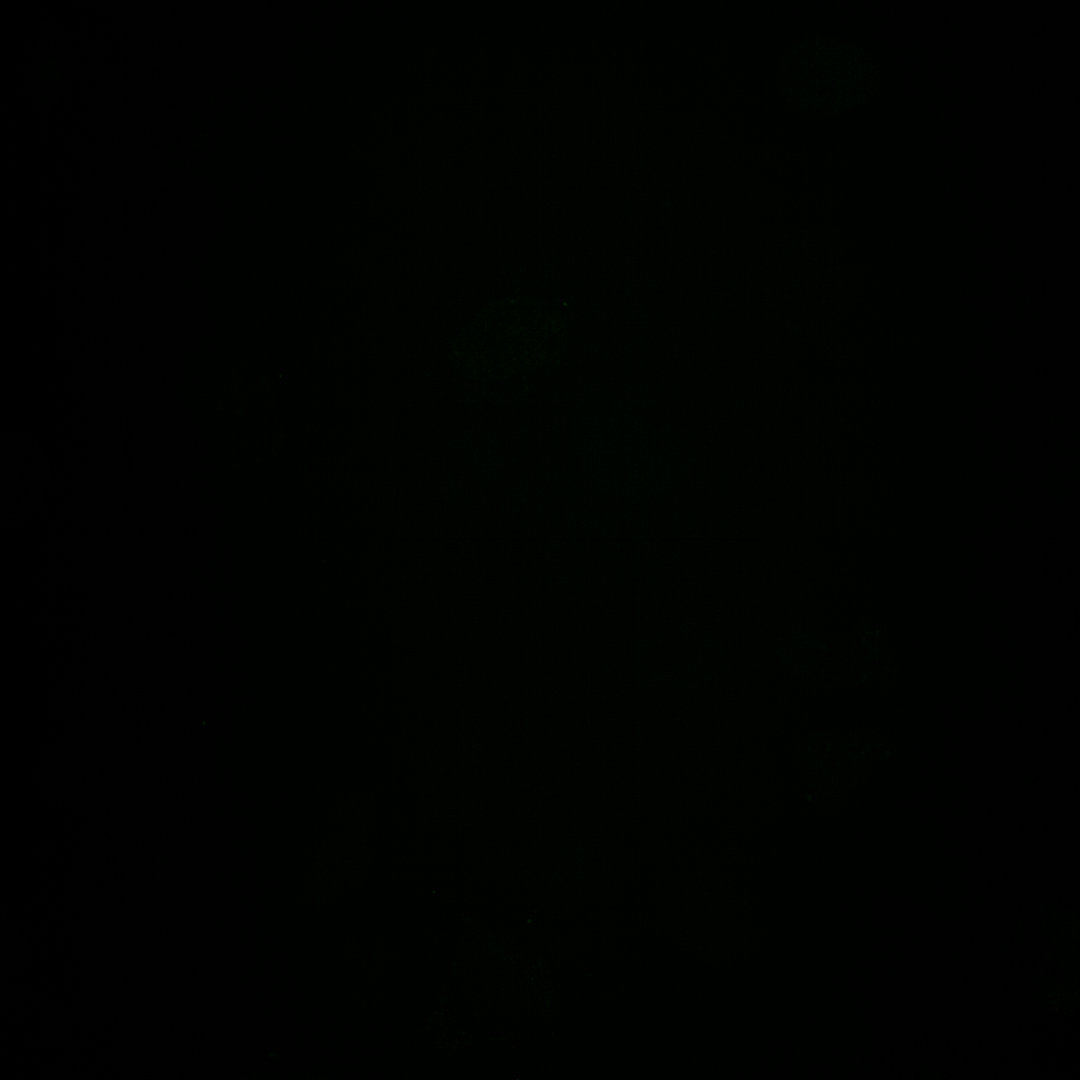

Supplement: Supplementary file 12 — Figure EV4 Source Data [file 44318_2024_108_MOESM12_ESM.zip › EMBOJ-2023-115654_FigEV4_sourcedata/EV4F/E240103 WT dC PLA GFP.png]

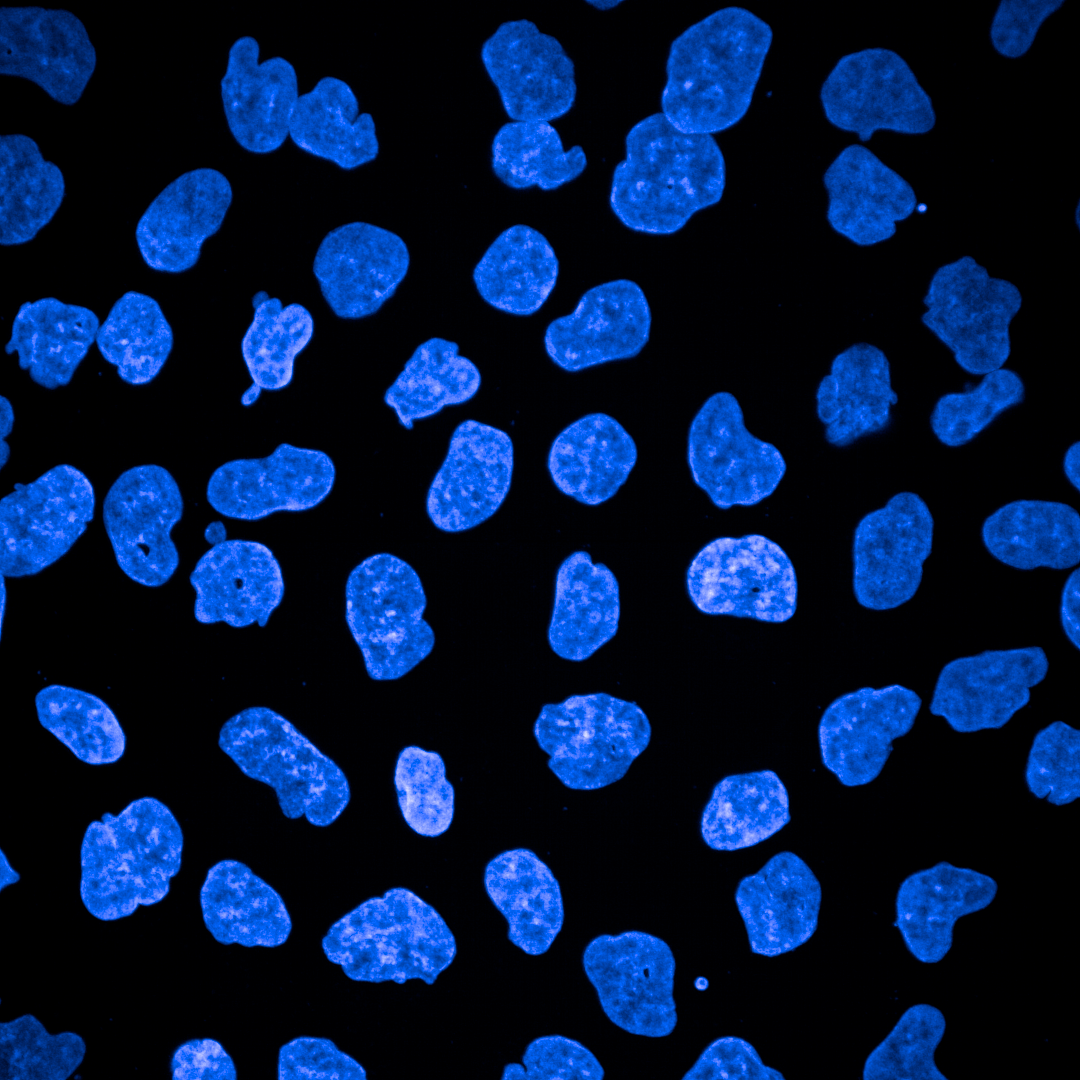

Supplement: Supplementary file 12 — Figure EV4 Source Data [file 44318_2024_108_MOESM12_ESM.zip › EMBOJ-2023-115654_FigEV4_sourcedata/EV4F/E240103 EV 5dC PLA DAPI.png]

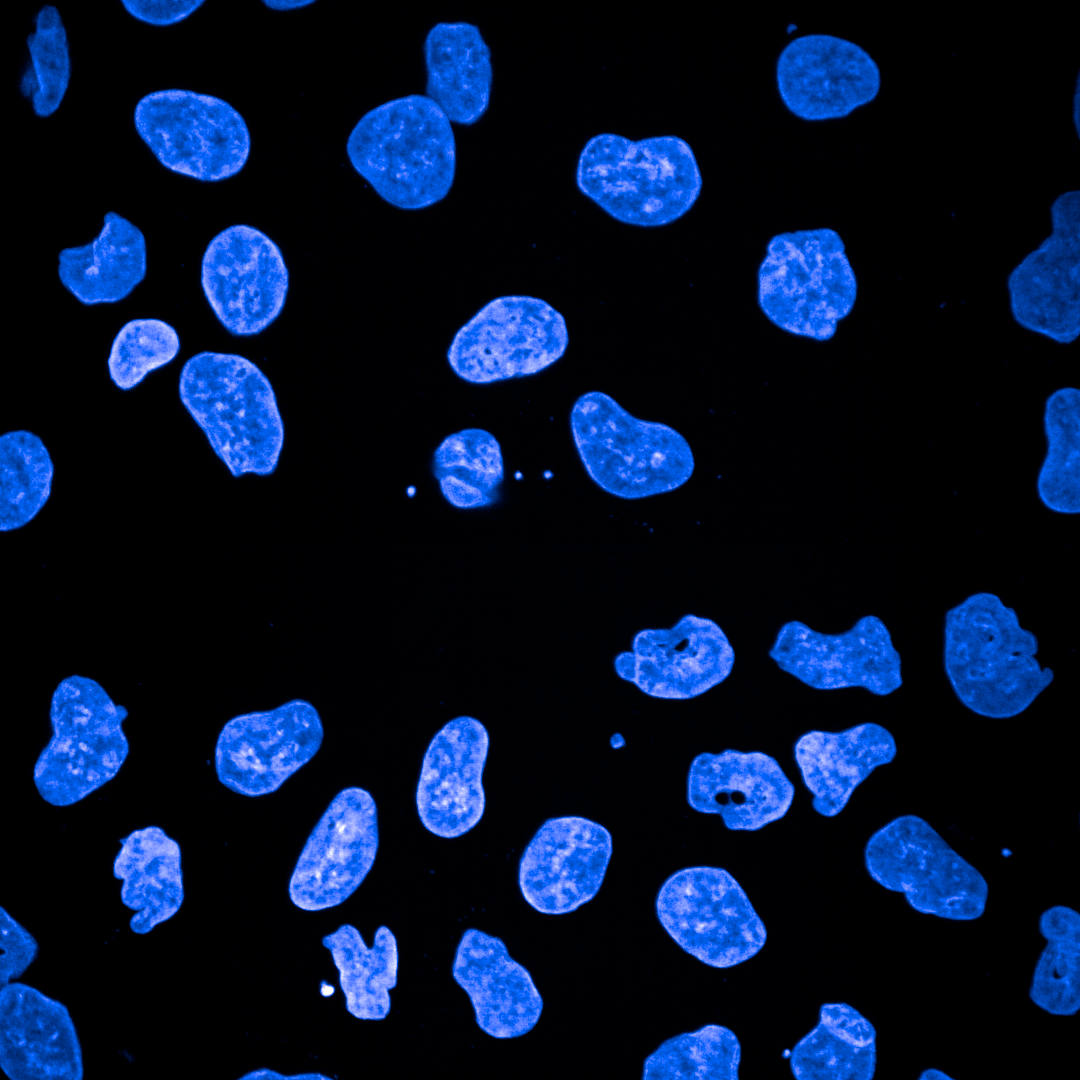

Supplement: Supplementary file 12 — Figure EV4 Source Data [file 44318_2024_108_MOESM12_ESM.zip › EMBOJ-2023-115654_FigEV4_sourcedata/EV4F/E240103 WT dC PLA DAPI.png]

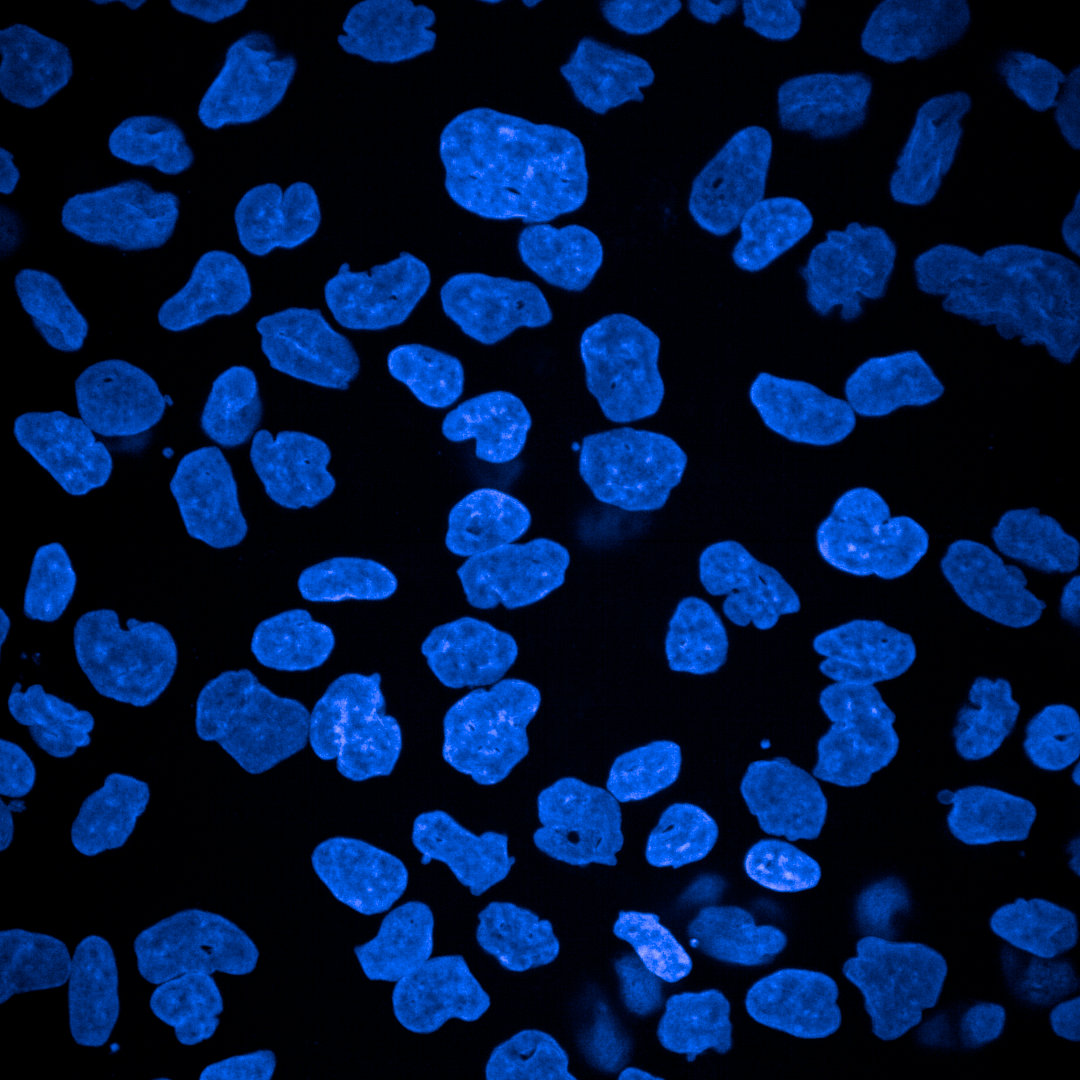

Supplement: Supplementary file 12 — Figure EV4 Source Data [file 44318_2024_108_MOESM12_ESM.zip › EMBOJ-2023-115654_FigEV4_sourcedata/EV4F/E240103 DSIM dC PLA DAPI.png]

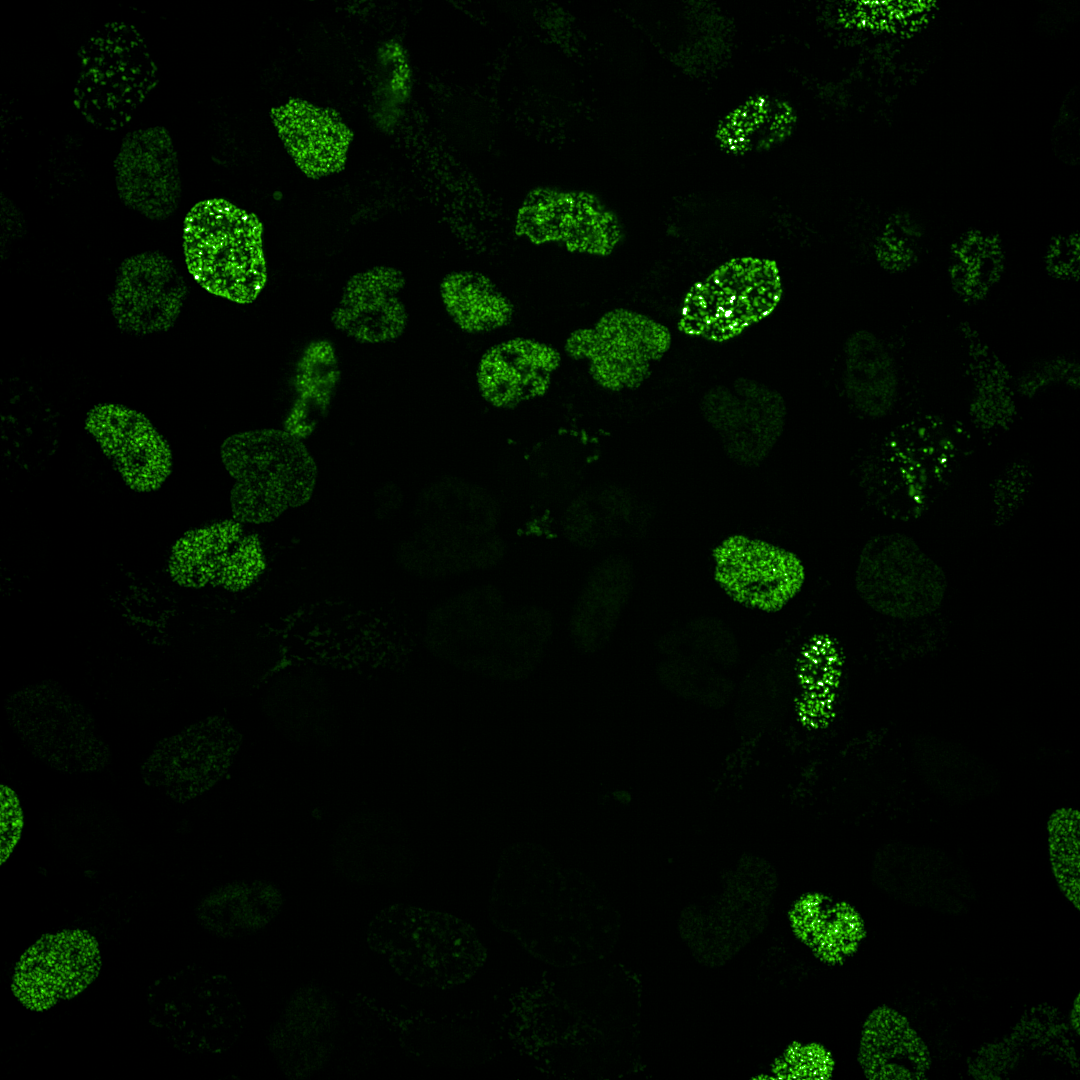

Supplement: Supplementary file 12 — Figure EV4 Source Data [file 44318_2024_108_MOESM12_ESM.zip › EMBOJ-2023-115654_FigEV4_sourcedata/EV4F/E240103 DSIM 5dC PLA GFP.png]

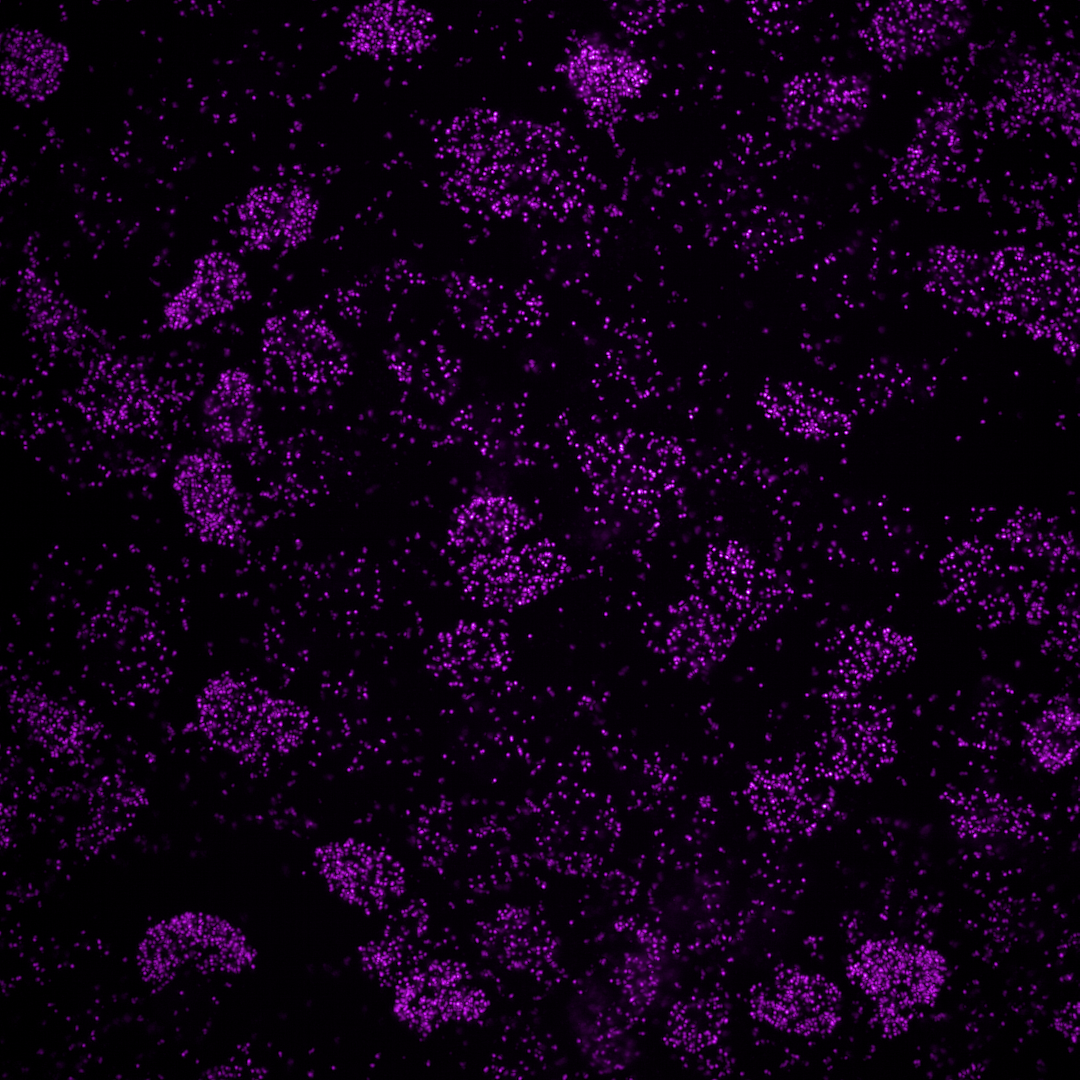

Supplement: Supplementary file 12 — Figure EV4 Source Data [file 44318_2024_108_MOESM12_ESM.zip › EMBOJ-2023-115654_FigEV4_sourcedata/EV4F/E240103 DSIM dC PLA PLA.png]

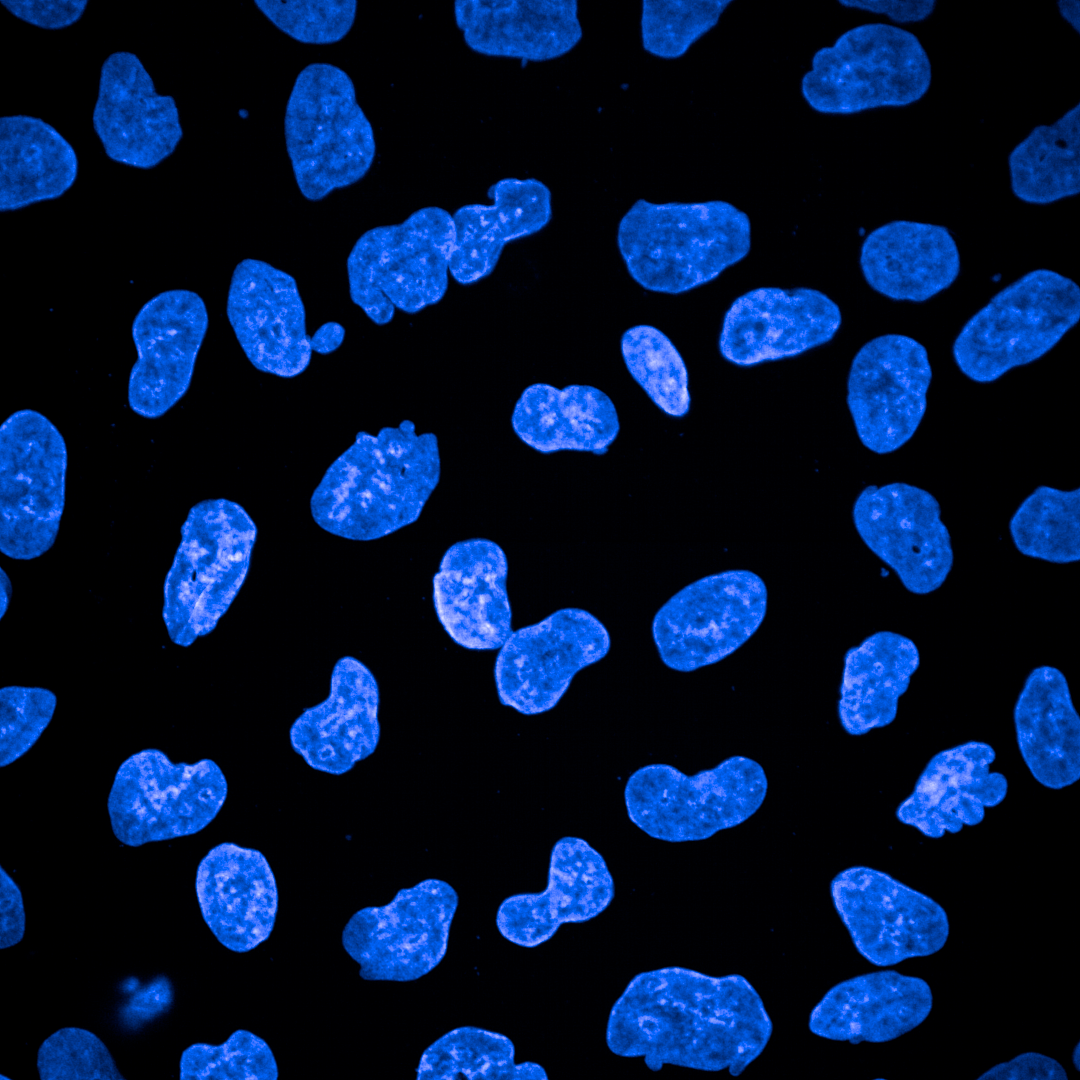

Supplement: Supplementary file 12 — Figure EV4 Source Data [file 44318_2024_108_MOESM12_ESM.zip › EMBOJ-2023-115654_FigEV4_sourcedata/EV4F/E240103 WT 5dC PLA DAPI 2.png]

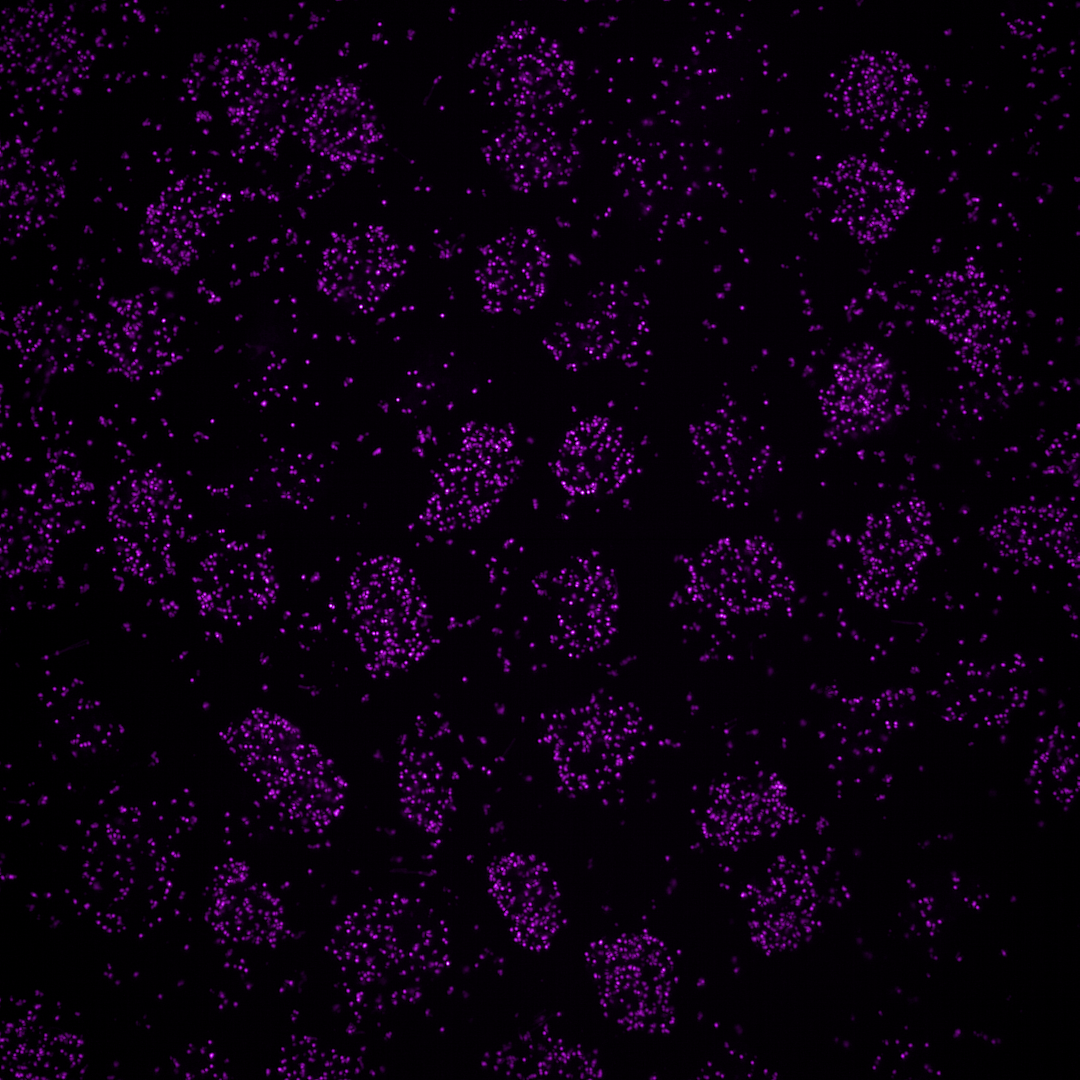

Supplement: Supplementary file 12 — Figure EV4 Source Data [file 44318_2024_108_MOESM12_ESM.zip › EMBOJ-2023-115654_FigEV4_sourcedata/EV4F/E240103 EV 5dC PLA PLA.png]

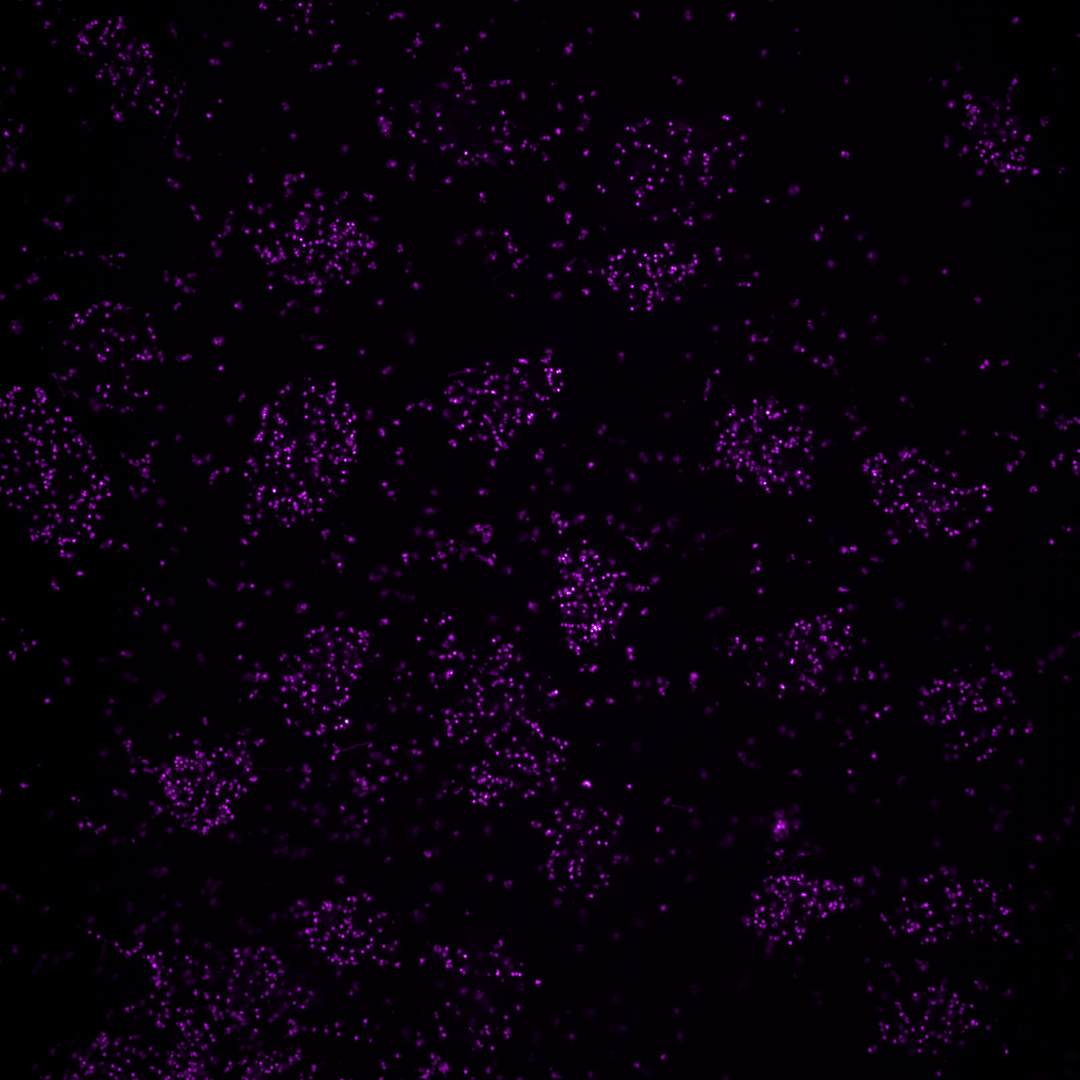

Supplement: Supplementary file 12 — Figure EV4 Source Data [file 44318_2024_108_MOESM12_ESM.zip › EMBOJ-2023-115654_FigEV4_sourcedata/EV4F/E240103 EV dC PLA PLA.png]

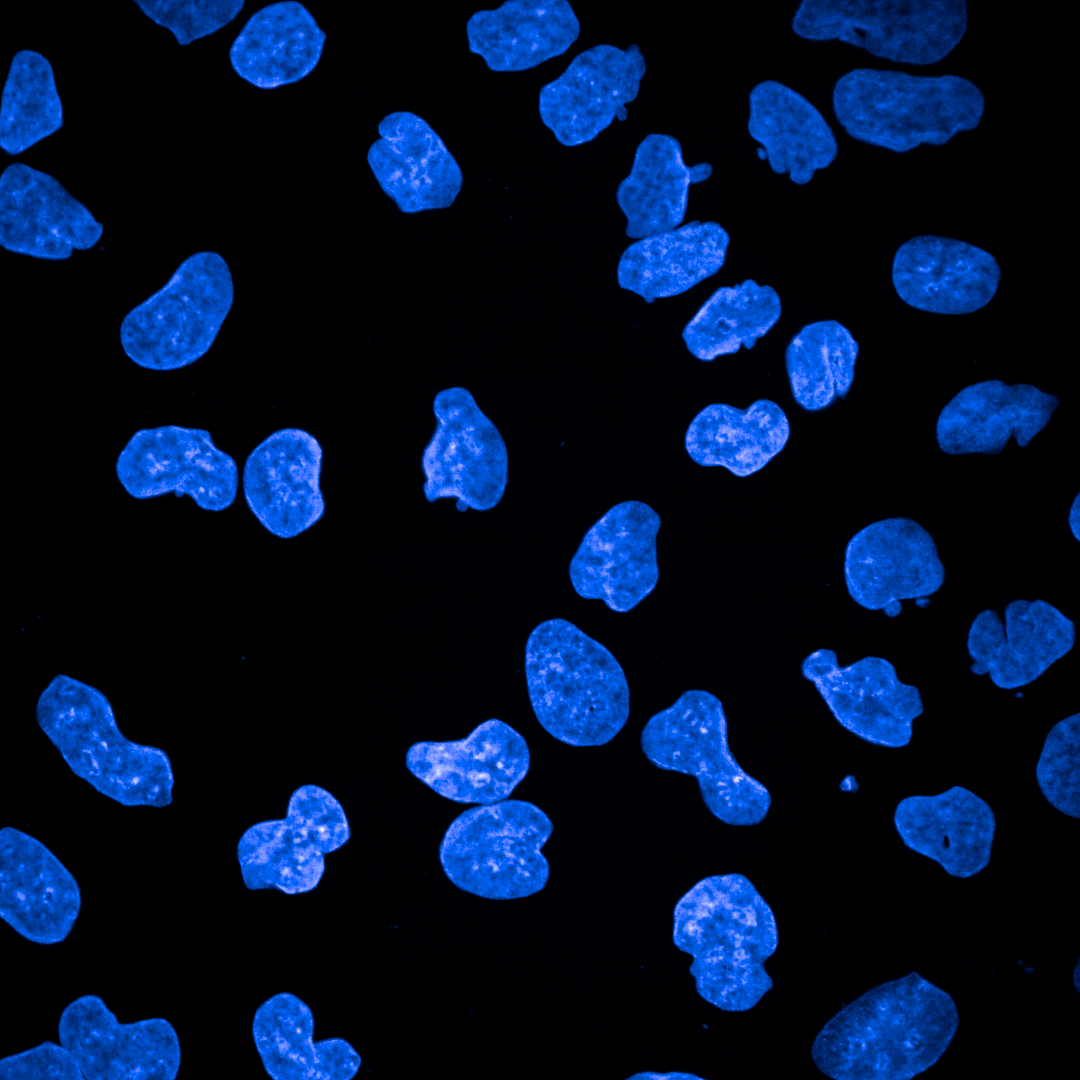

Supplement: Supplementary file 12 — Figure EV4 Source Data [file 44318_2024_108_MOESM12_ESM.zip › EMBOJ-2023-115654_FigEV4_sourcedata/EV4F/E240103 WT 5dC GFPonly DAPI.png]

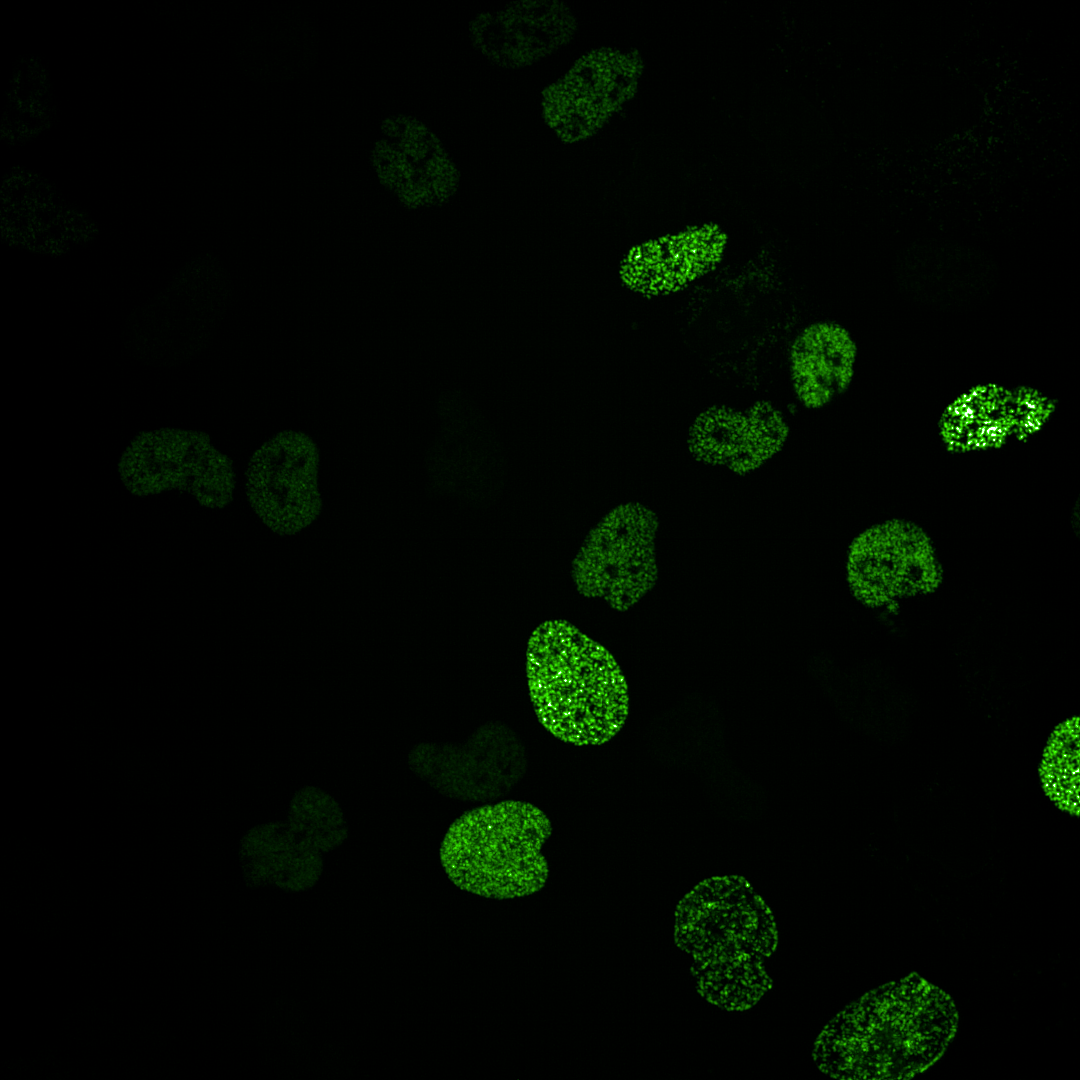

Supplement: Supplementary file 12 — Figure EV4 Source Data [file 44318_2024_108_MOESM12_ESM.zip › EMBOJ-2023-115654_FigEV4_sourcedata/EV4F/E240103 WT 5dC GFPonly GFP.png]

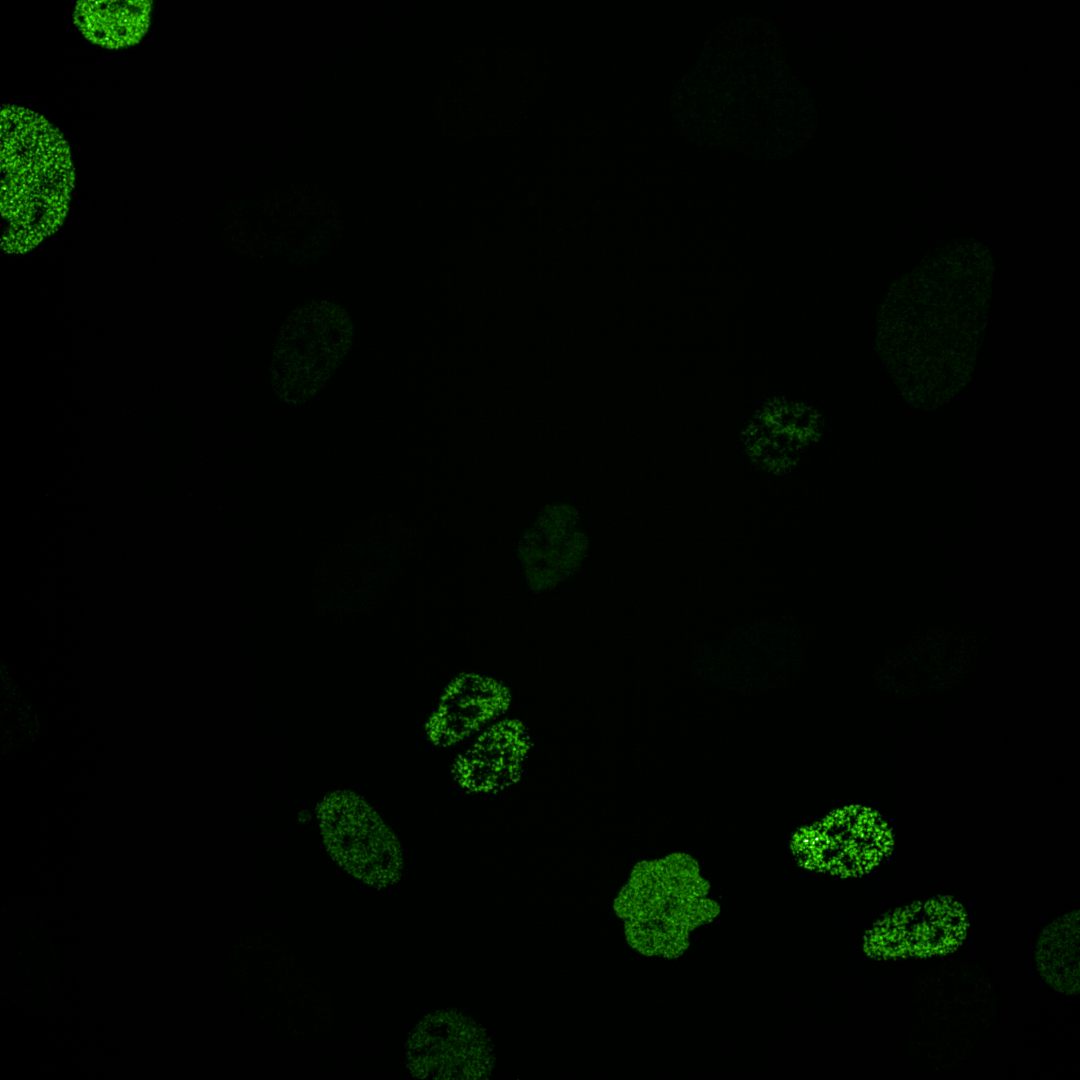

Supplement: Supplementary file 12 — Figure EV4 Source Data [file 44318_2024_108_MOESM12_ESM.zip › EMBOJ-2023-115654_FigEV4_sourcedata/EV4F/E240103 WT 5dC HAonly GFP.png]

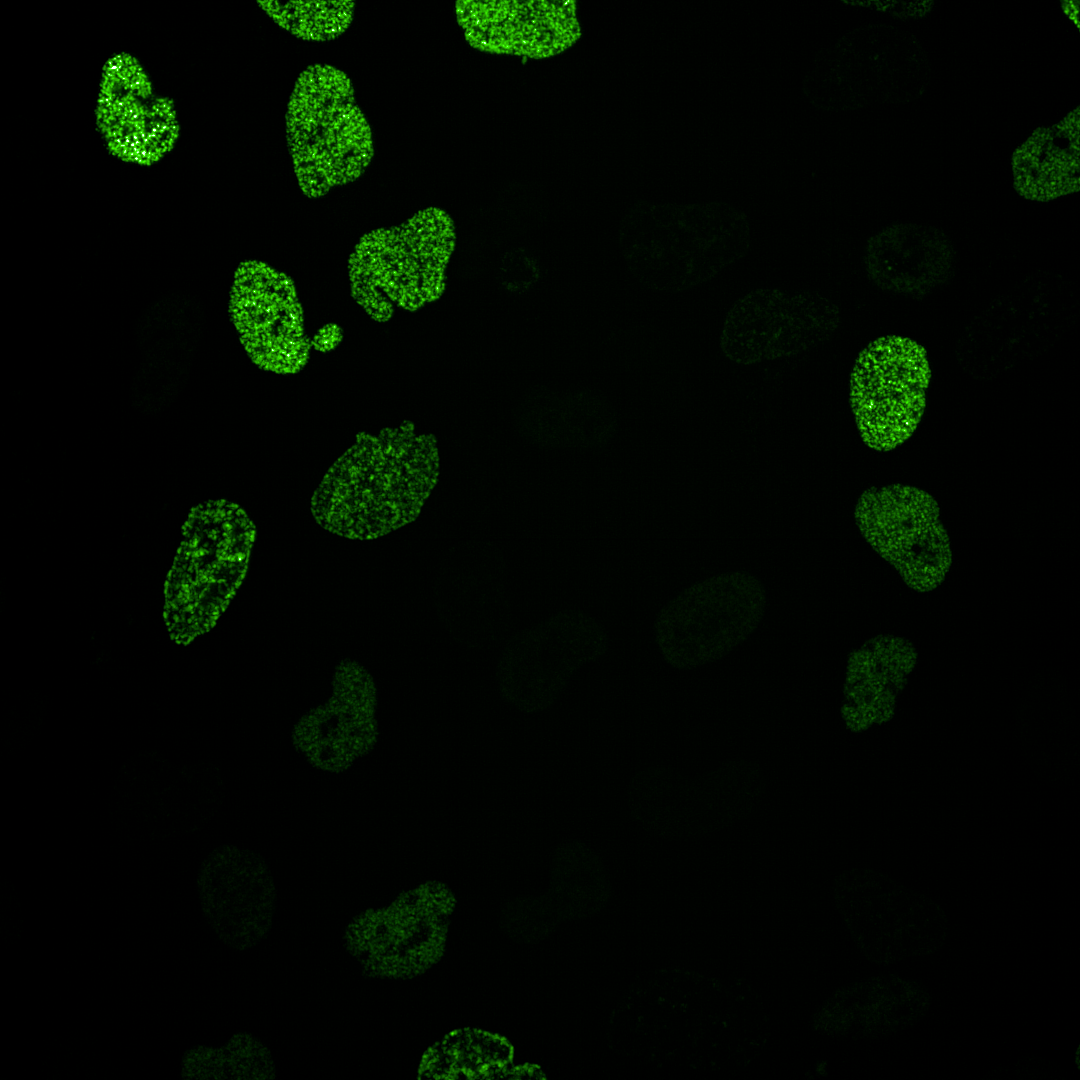

Supplement: Supplementary file 12 — Figure EV4 Source Data [file 44318_2024_108_MOESM12_ESM.zip › EMBOJ-2023-115654_FigEV4_sourcedata/EV4F/E240103 WT 5dC PLA GFP 2.png]

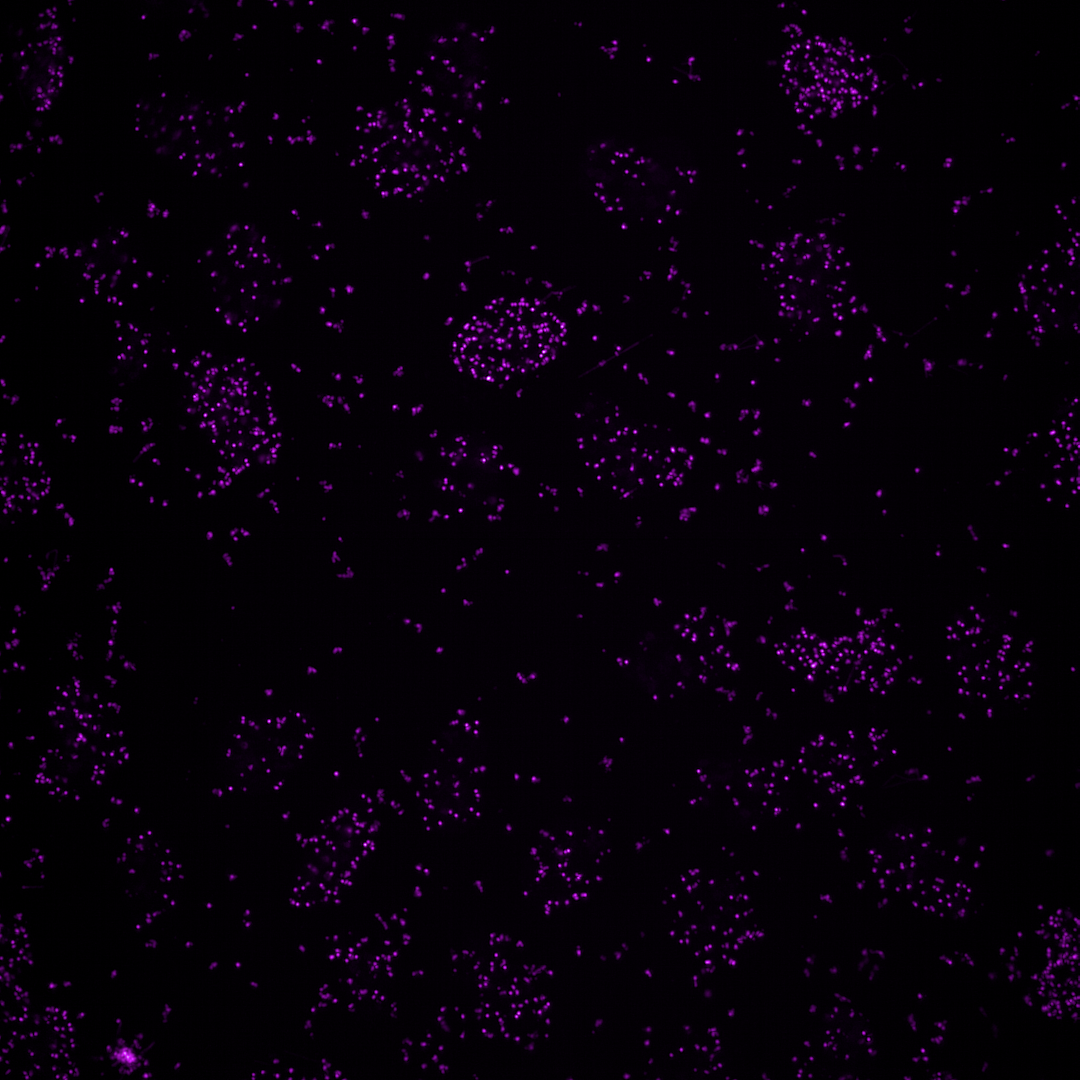

Supplement: Supplementary file 12 — Figure EV4 Source Data [file 44318_2024_108_MOESM12_ESM.zip › EMBOJ-2023-115654_FigEV4_sourcedata/EV4F/E240103 WT dC PLA PLA.png]

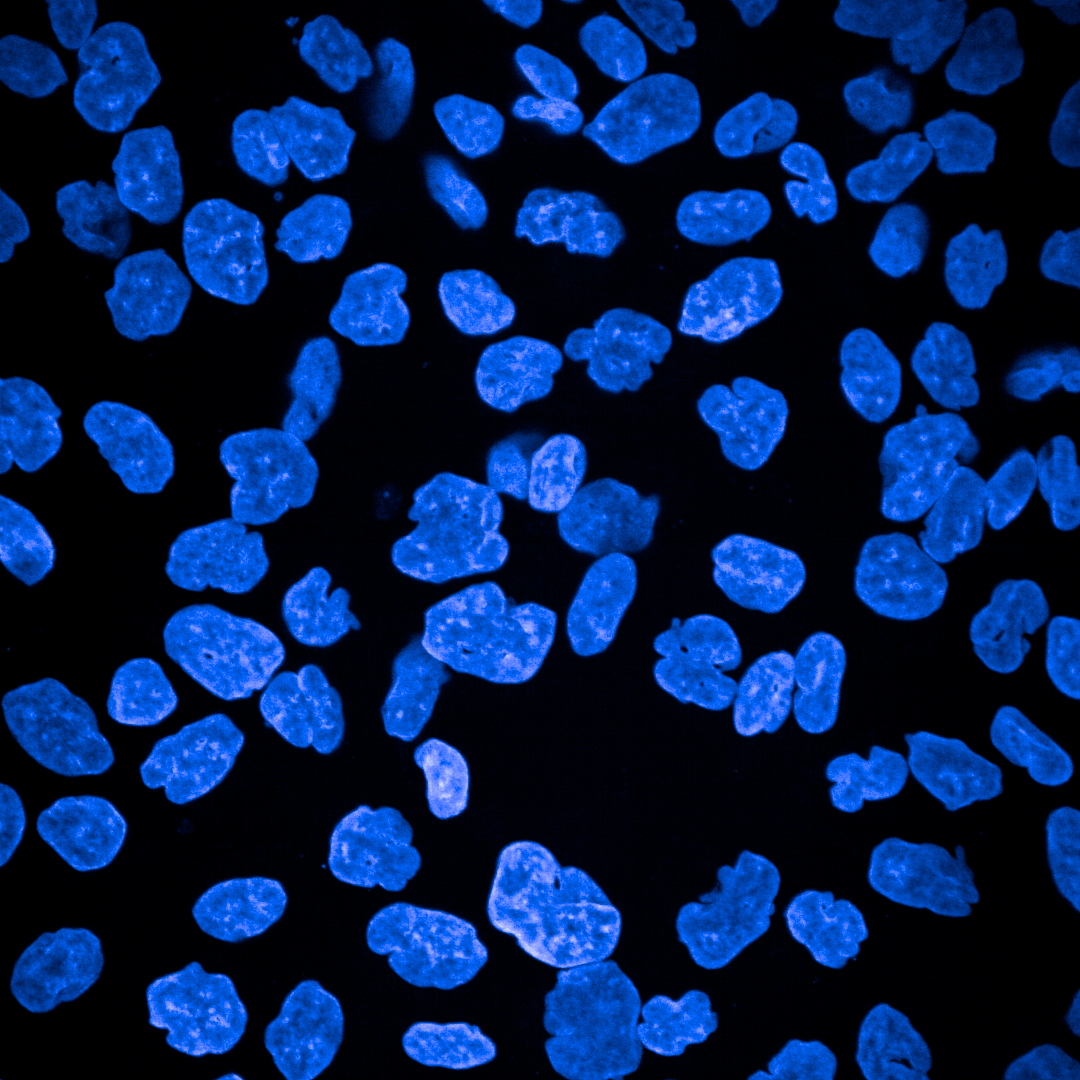

Supplement: Supplementary file 12 — Figure EV4 Source Data [file 44318_2024_108_MOESM12_ESM.zip › EMBOJ-2023-115654_FigEV4_sourcedata/EV4F/E240103 DSIM 5dC PLA DAPI.png]

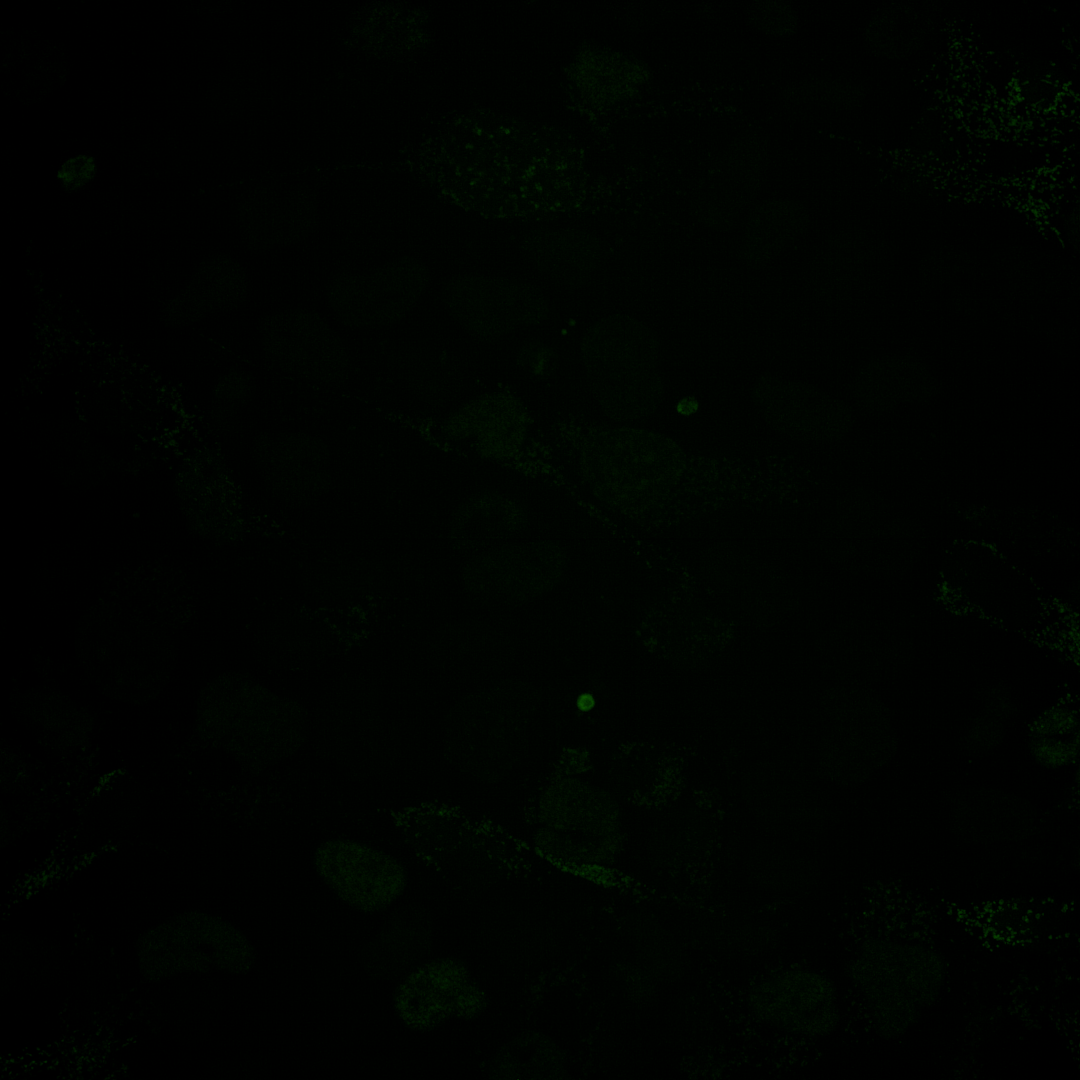

Supplement: Supplementary file 12 — Figure EV4 Source Data [file 44318_2024_108_MOESM12_ESM.zip › EMBOJ-2023-115654_FigEV4_sourcedata/EV4F/E240103 DSIM dC PLA GFP.png]

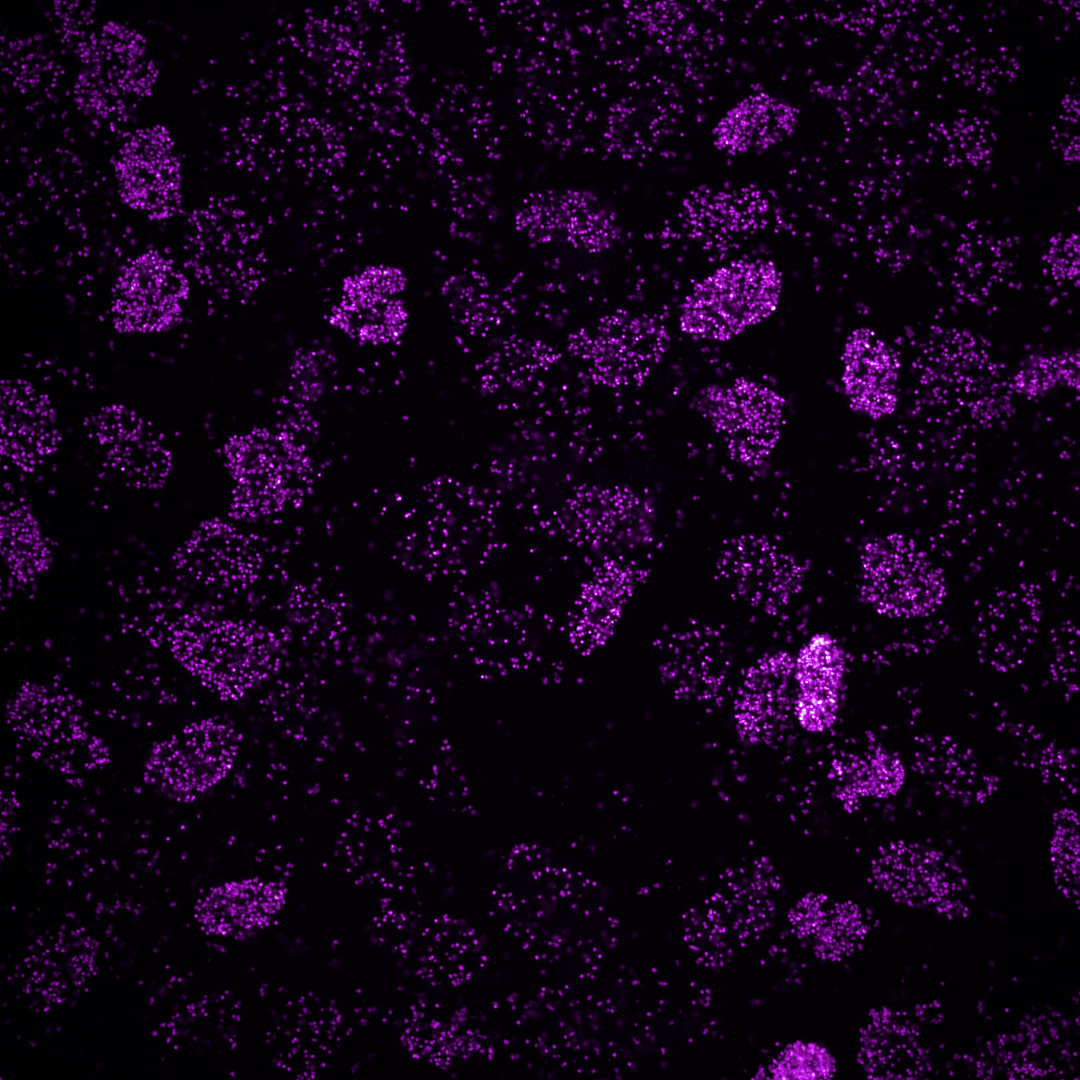

Supplement: Supplementary file 12 — Figure EV4 Source Data [file 44318_2024_108_MOESM12_ESM.zip › EMBOJ-2023-115654_FigEV4_sourcedata/EV4F/E240103 DSIM 5dC PLA PLA.png]

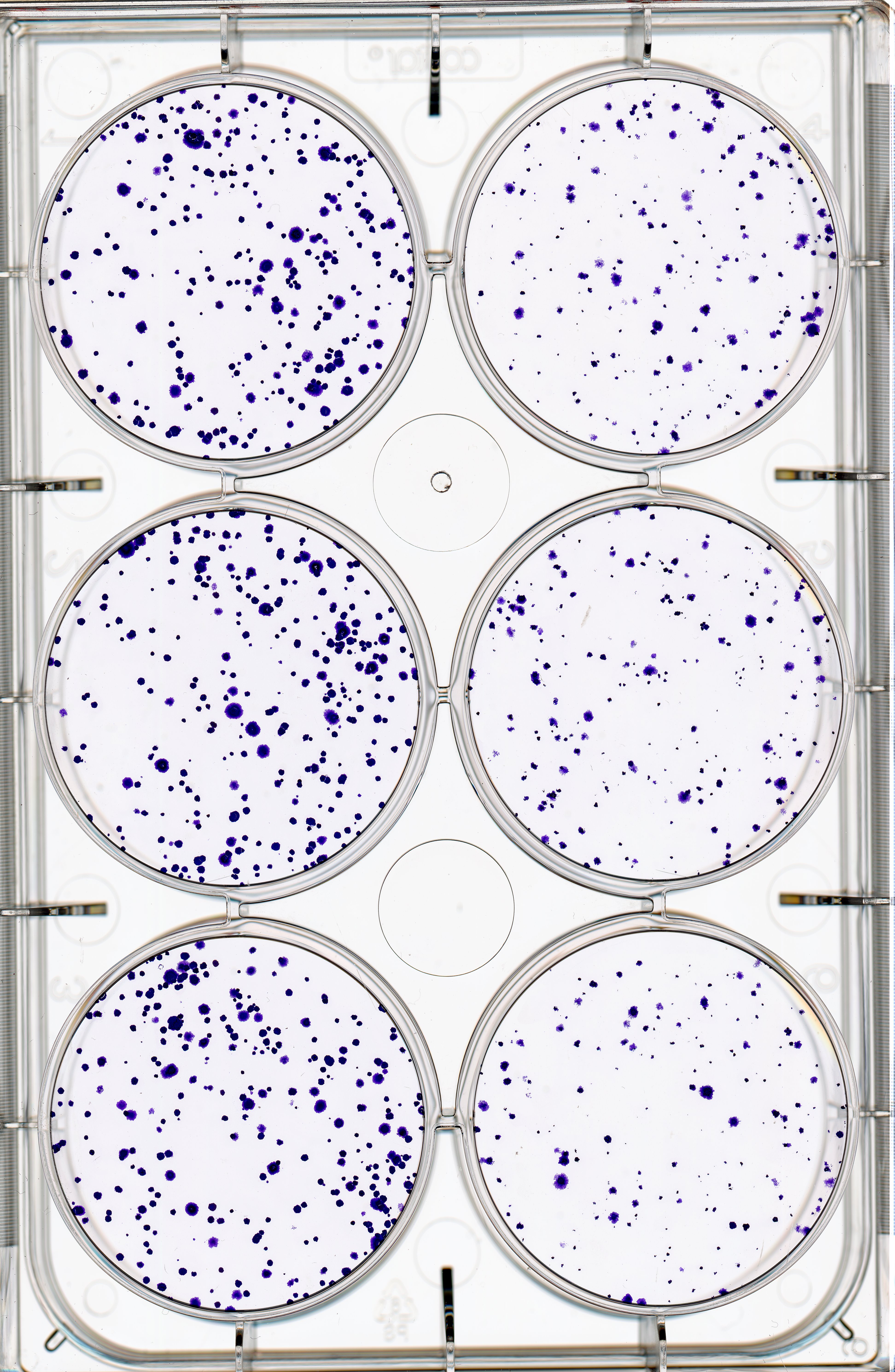

Supplement: Supplementary file 12 — Figure EV4 Source Data [file 44318_2024_108_MOESM12_ESM.zip › EMBOJ-2023-115654_FigEV4_sourcedata/EV4A/E231204 WTsiCtrl 5dC0-100.jpg]

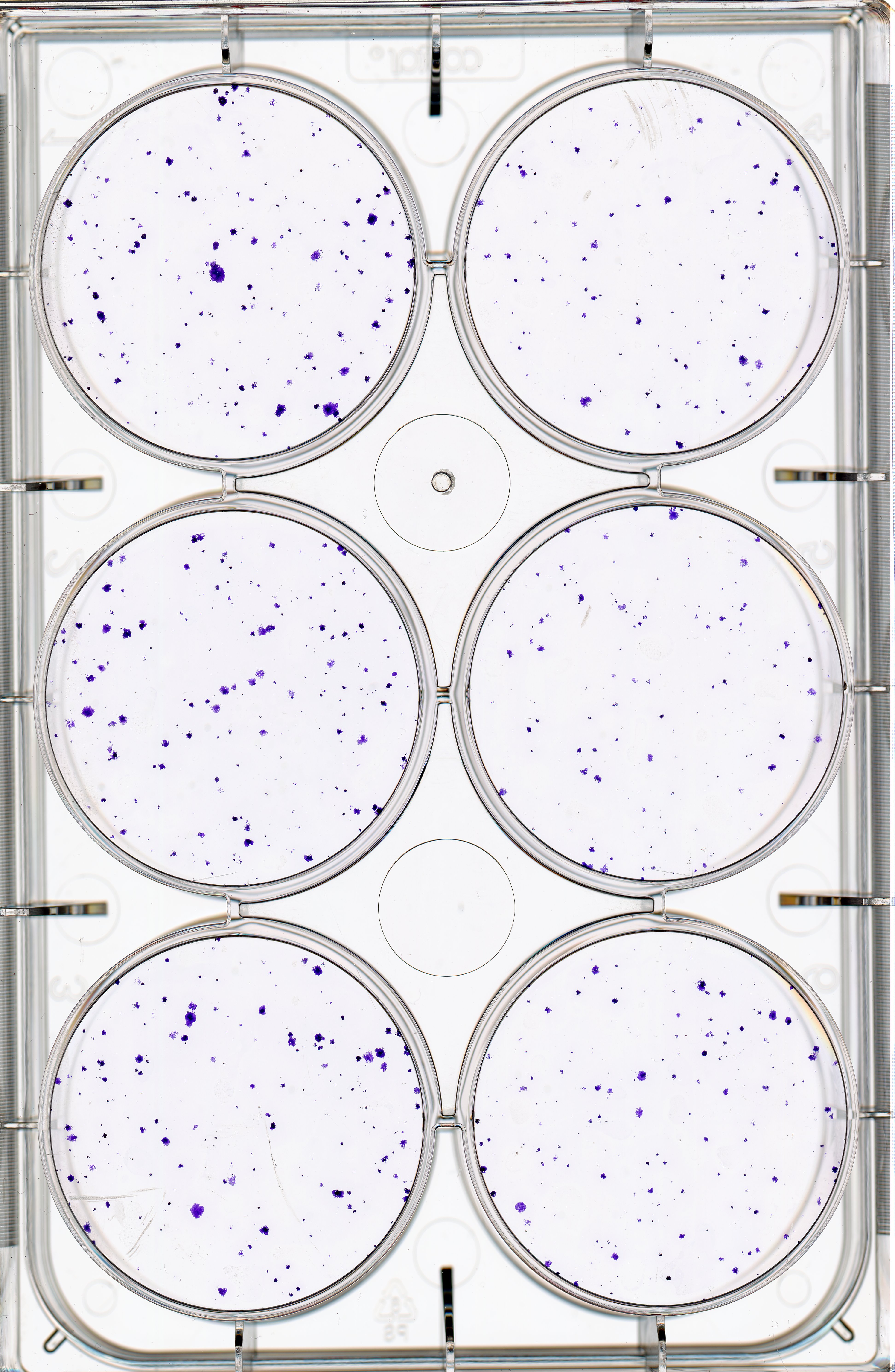

Supplement: Supplementary file 12 — Figure EV4 Source Data [file 44318_2024_108_MOESM12_ESM.zip › EMBOJ-2023-115654_FigEV4_sourcedata/EV4A/E231204 WTsiCtrl 5dC200-400.jpg]

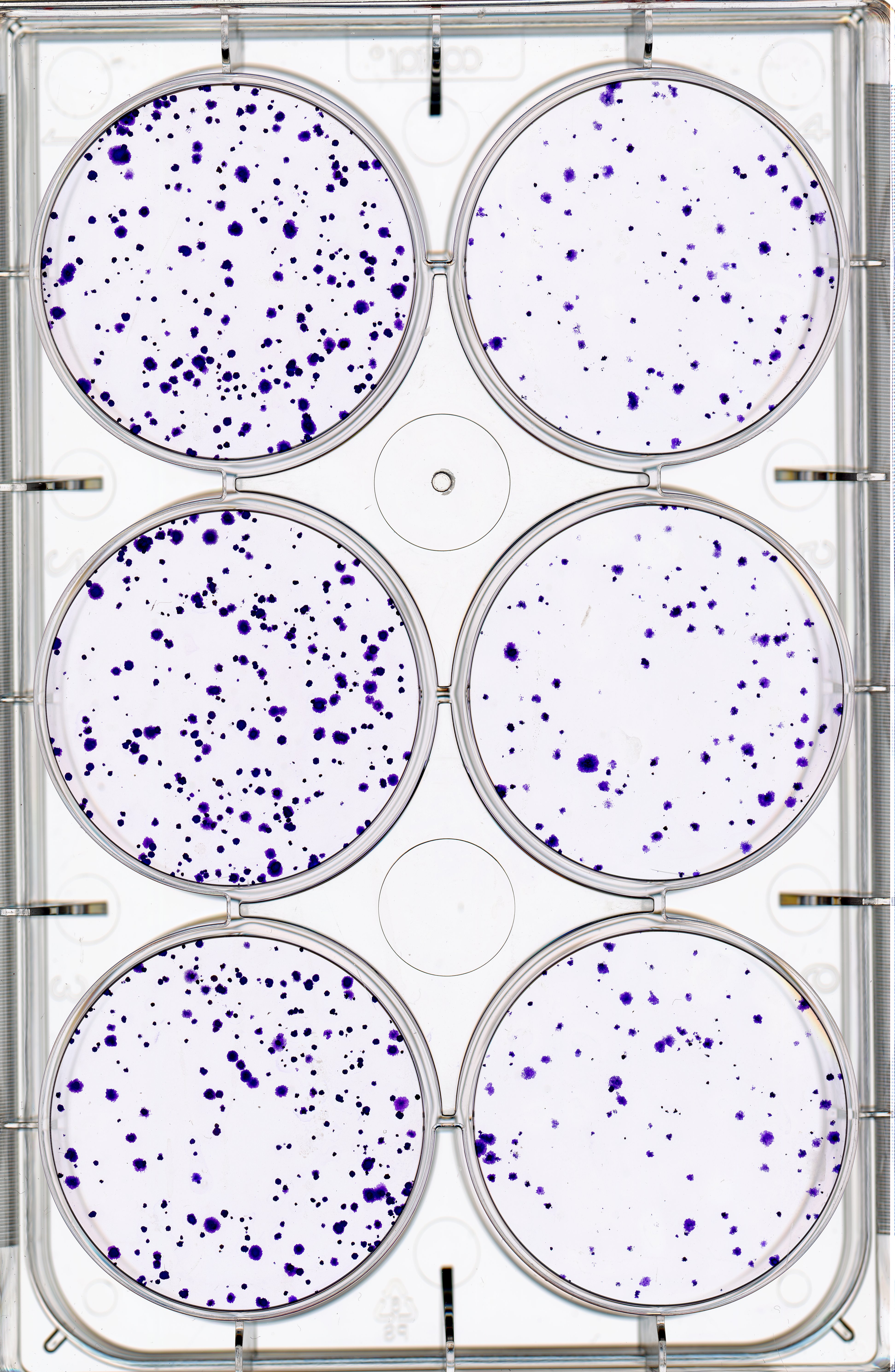

Supplement: Supplementary file 12 — Figure EV4 Source Data [file 44318_2024_108_MOESM12_ESM.zip › EMBOJ-2023-115654_FigEV4_sourcedata/EV4A/E231204 WTsiDNMT1 5dC0-100.jpg]

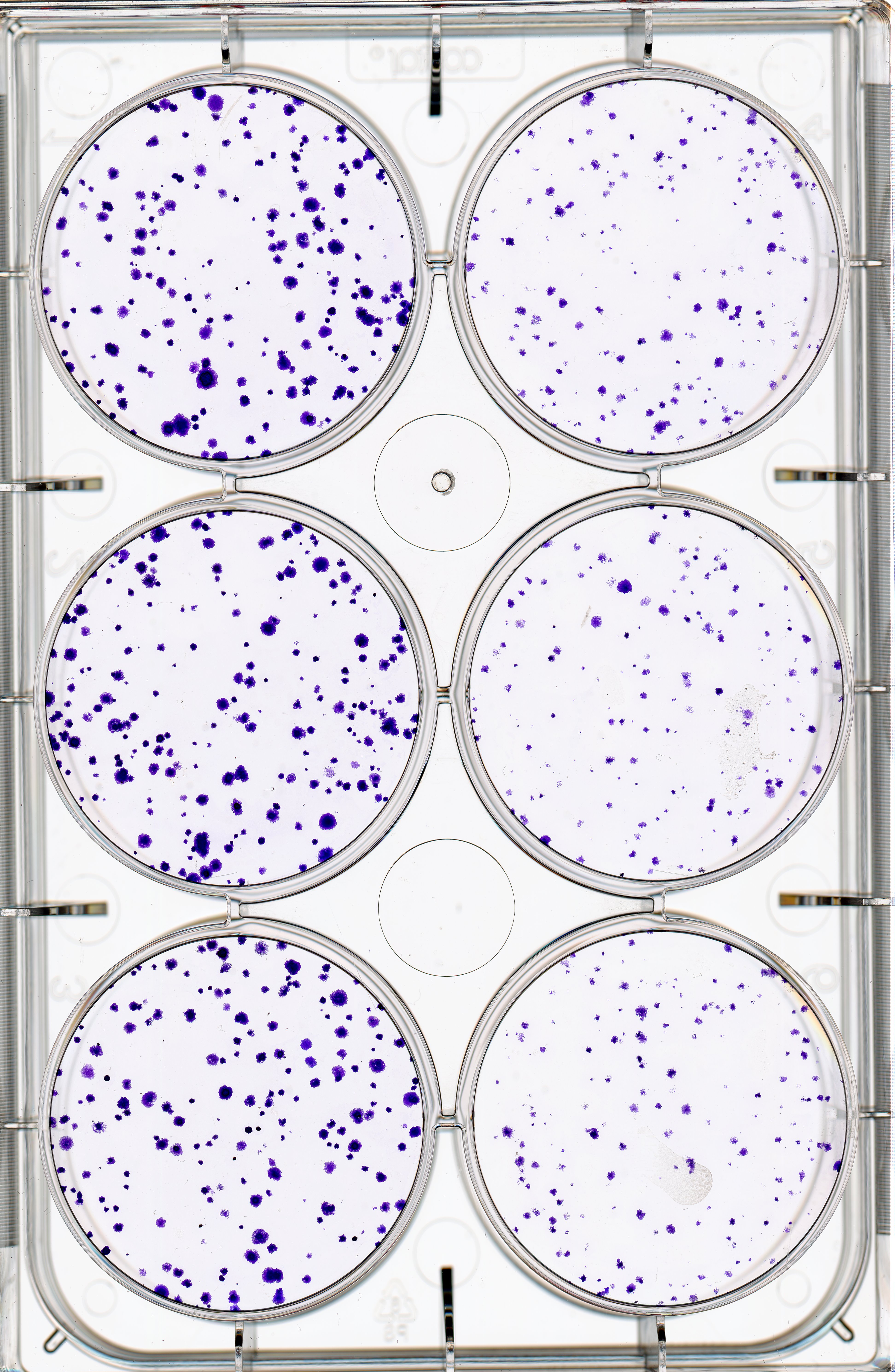

Supplement: Supplementary file 12 — Figure EV4 Source Data [file 44318_2024_108_MOESM12_ESM.zip › EMBOJ-2023-115654_FigEV4_sourcedata/EV4A/E231204 TOPORSsiDNMT1 5dC0-100.jpg]

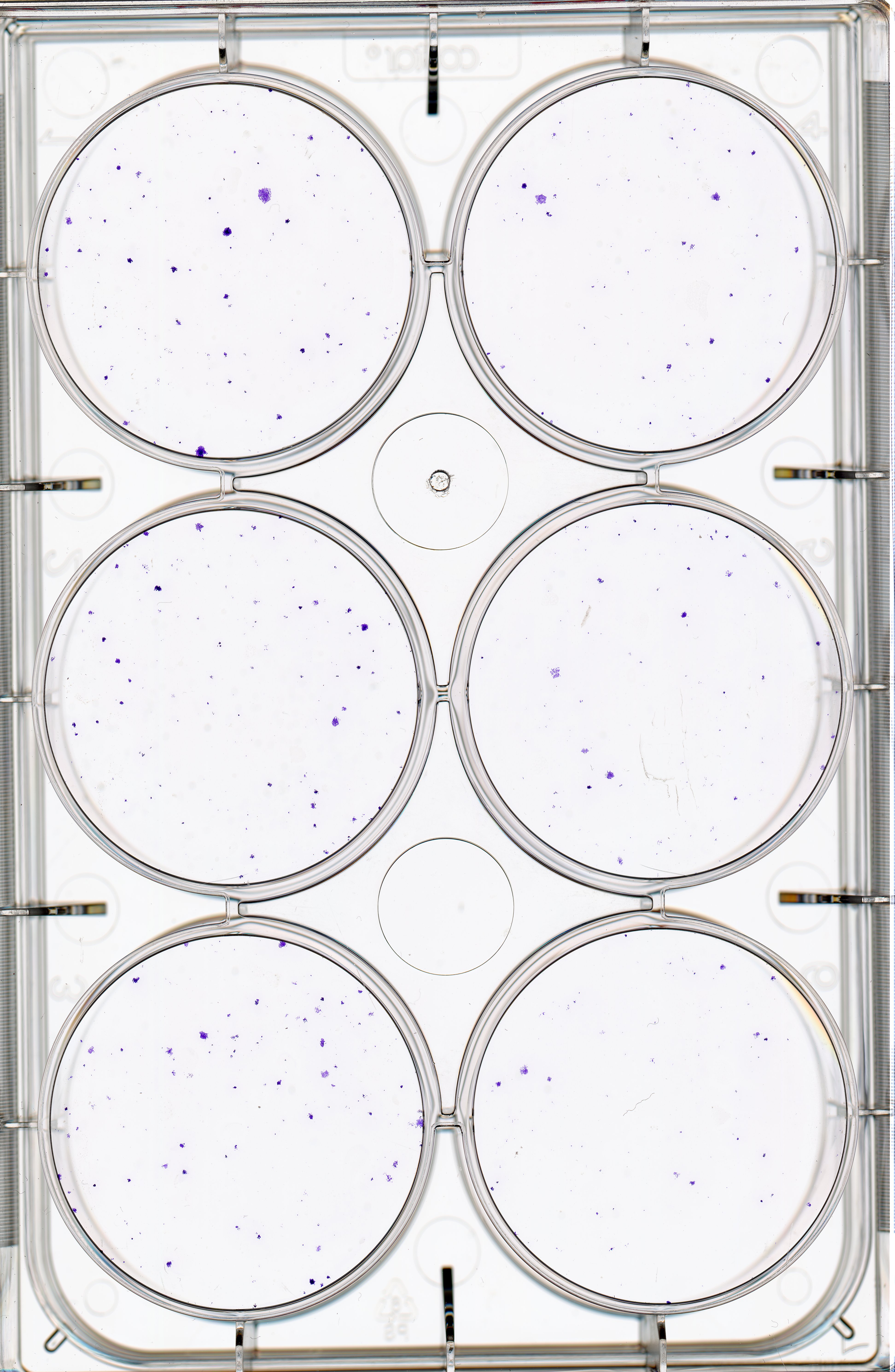

Supplement: Supplementary file 12 — Figure EV4 Source Data [file 44318_2024_108_MOESM12_ESM.zip › EMBOJ-2023-115654_FigEV4_sourcedata/EV4A/E231204 WTsiCtrl 5dC400-600.jpg]

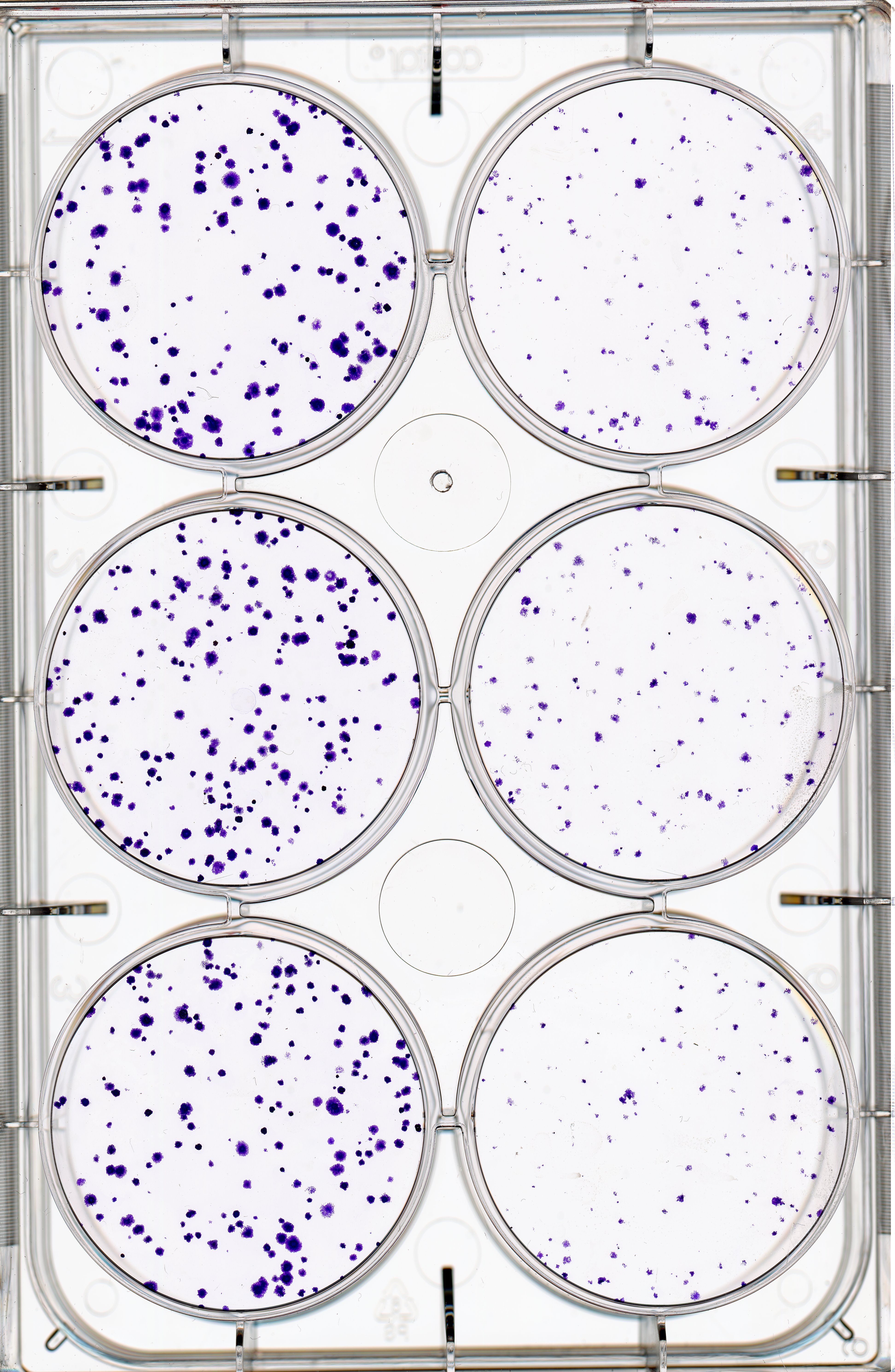

Supplement: Supplementary file 12 — Figure EV4 Source Data [file 44318_2024_108_MOESM12_ESM.zip › EMBOJ-2023-115654_FigEV4_sourcedata/EV4A/E231204 TOPORSsiCtrl 5dC0-100.jpg]

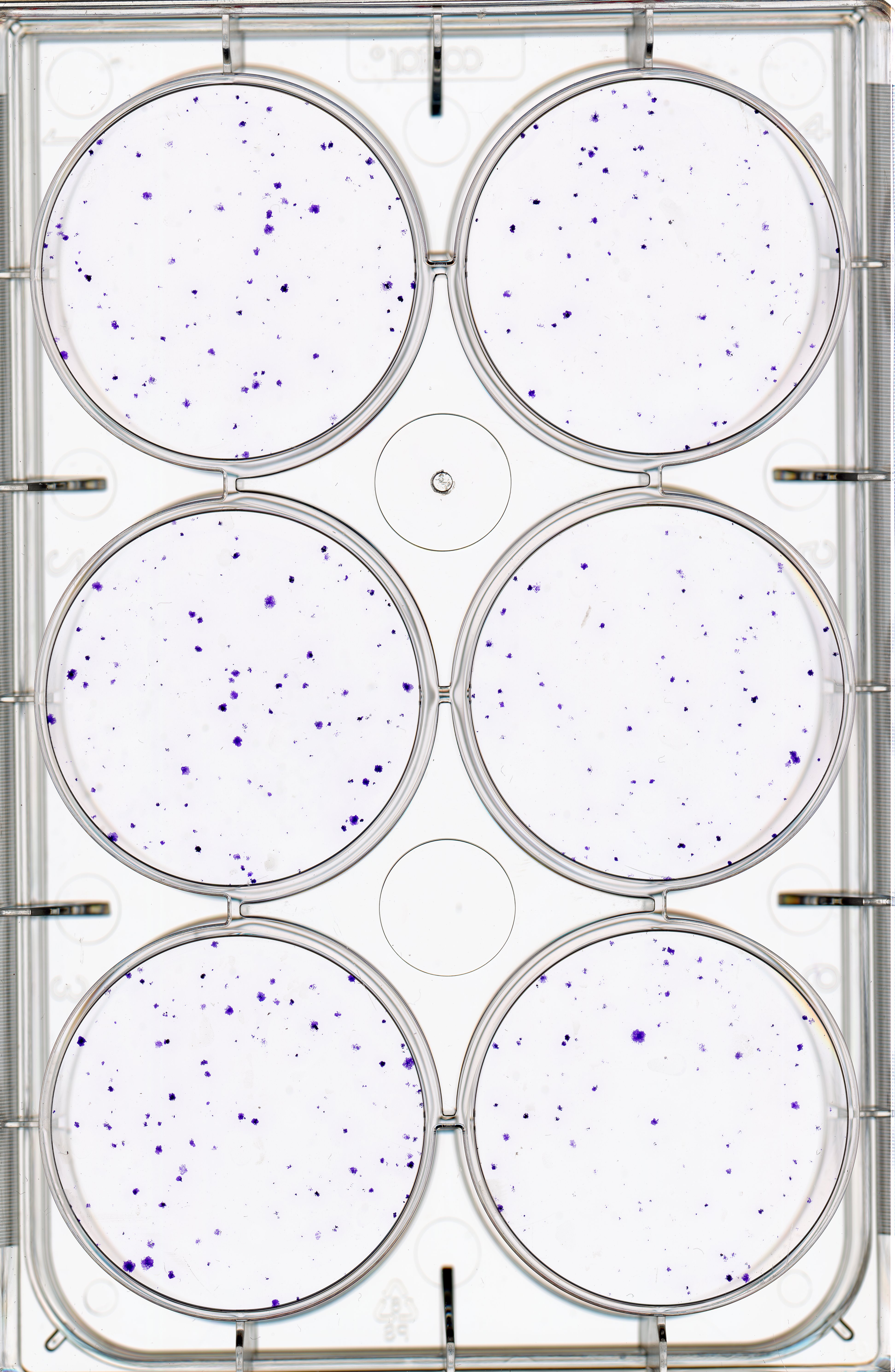

Supplement: Supplementary file 12 — Figure EV4 Source Data [file 44318_2024_108_MOESM12_ESM.zip › EMBOJ-2023-115654_FigEV4_sourcedata/EV4A/E231204 WTsiDNMT1 5dC200-400.jpg]

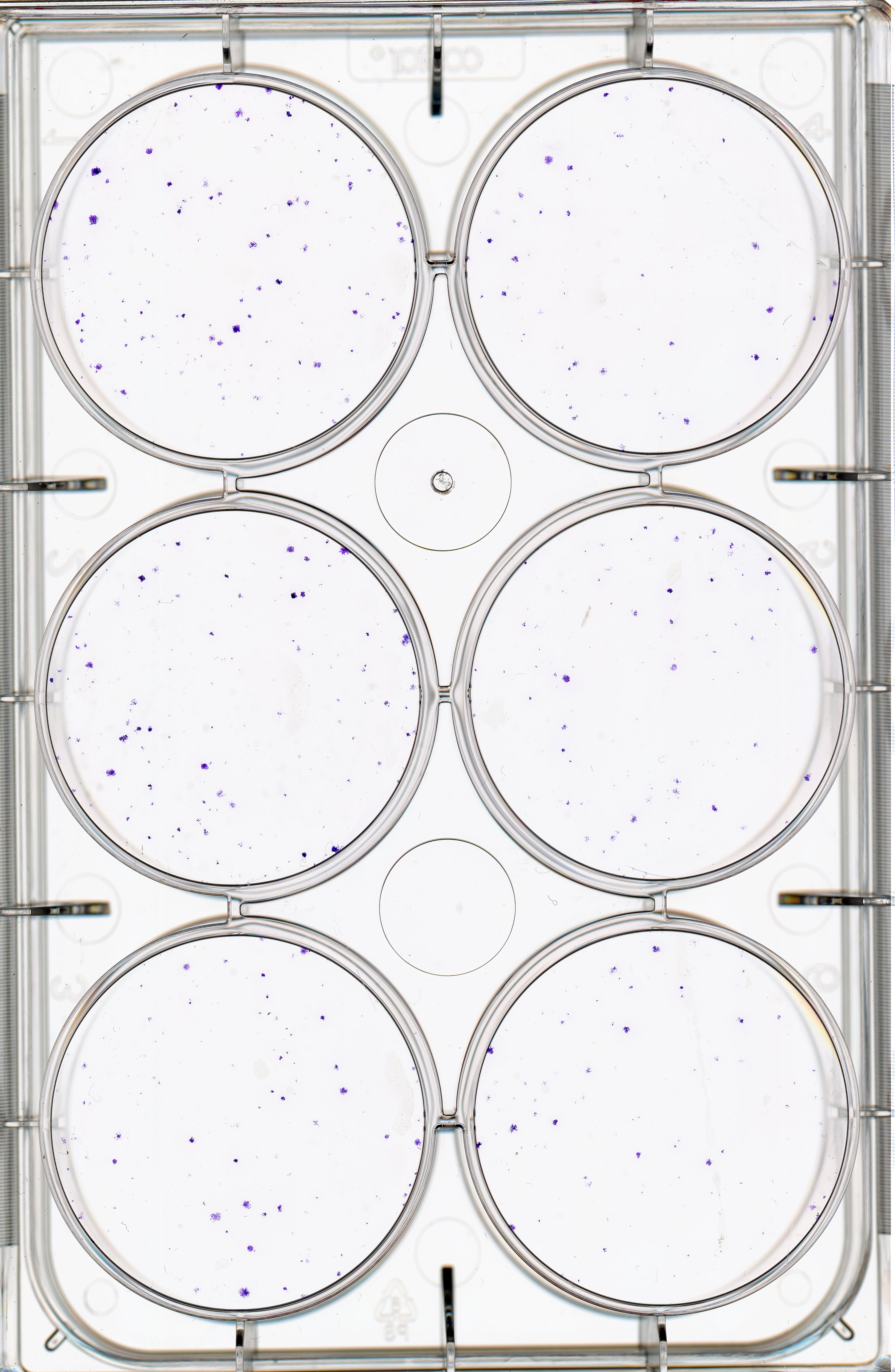

Supplement: Supplementary file 12 — Figure EV4 Source Data [file 44318_2024_108_MOESM12_ESM.zip › EMBOJ-2023-115654_FigEV4_sourcedata/EV4A/E231204 WTsiDNMT1 5dC400-600.jpg]

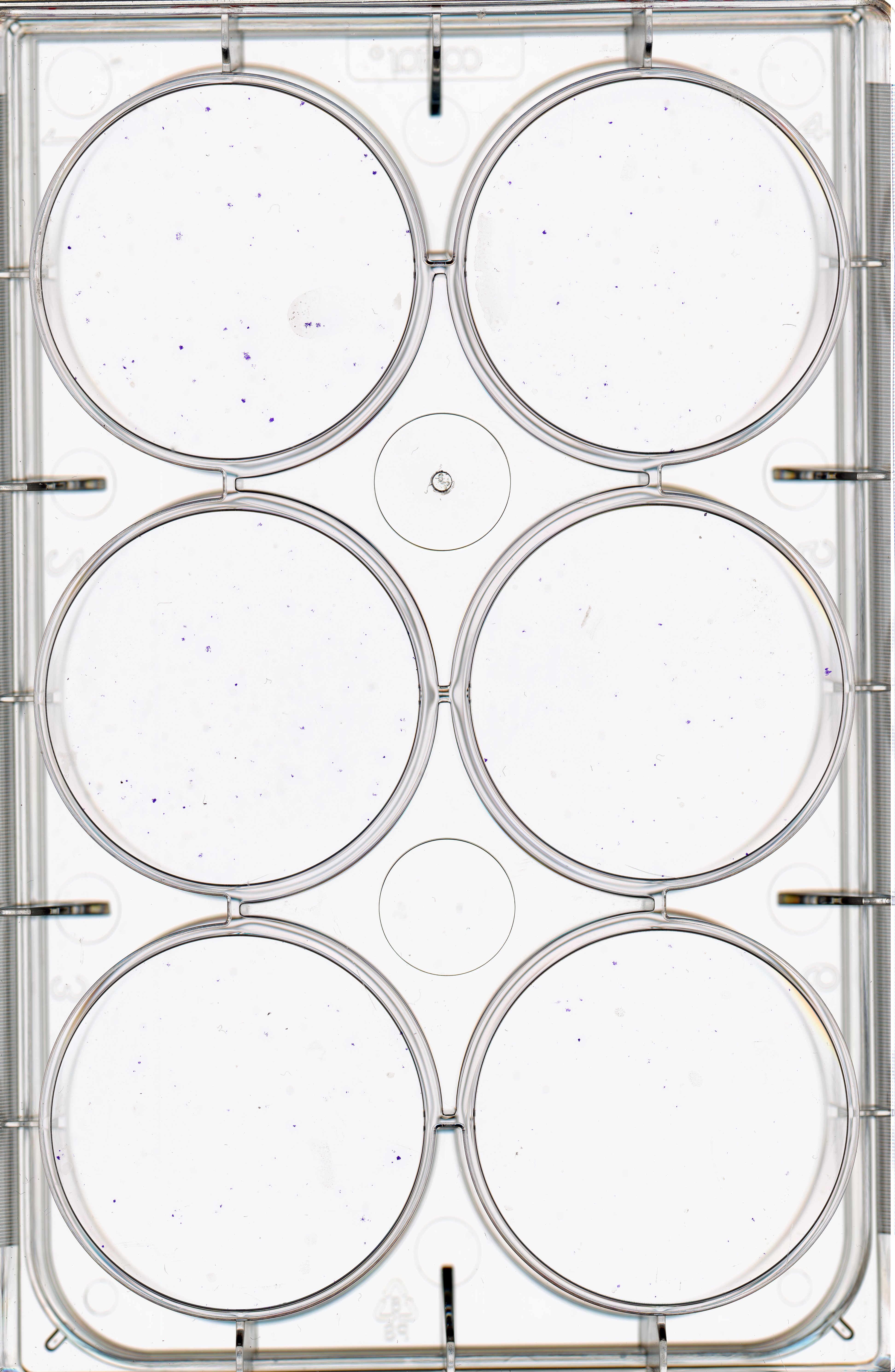

Supplement: Supplementary file 12 — Figure EV4 Source Data [file 44318_2024_108_MOESM12_ESM.zip › EMBOJ-2023-115654_FigEV4_sourcedata/EV4A/E231204 TOPORSsiCtrl 5dC400-600.jpg]

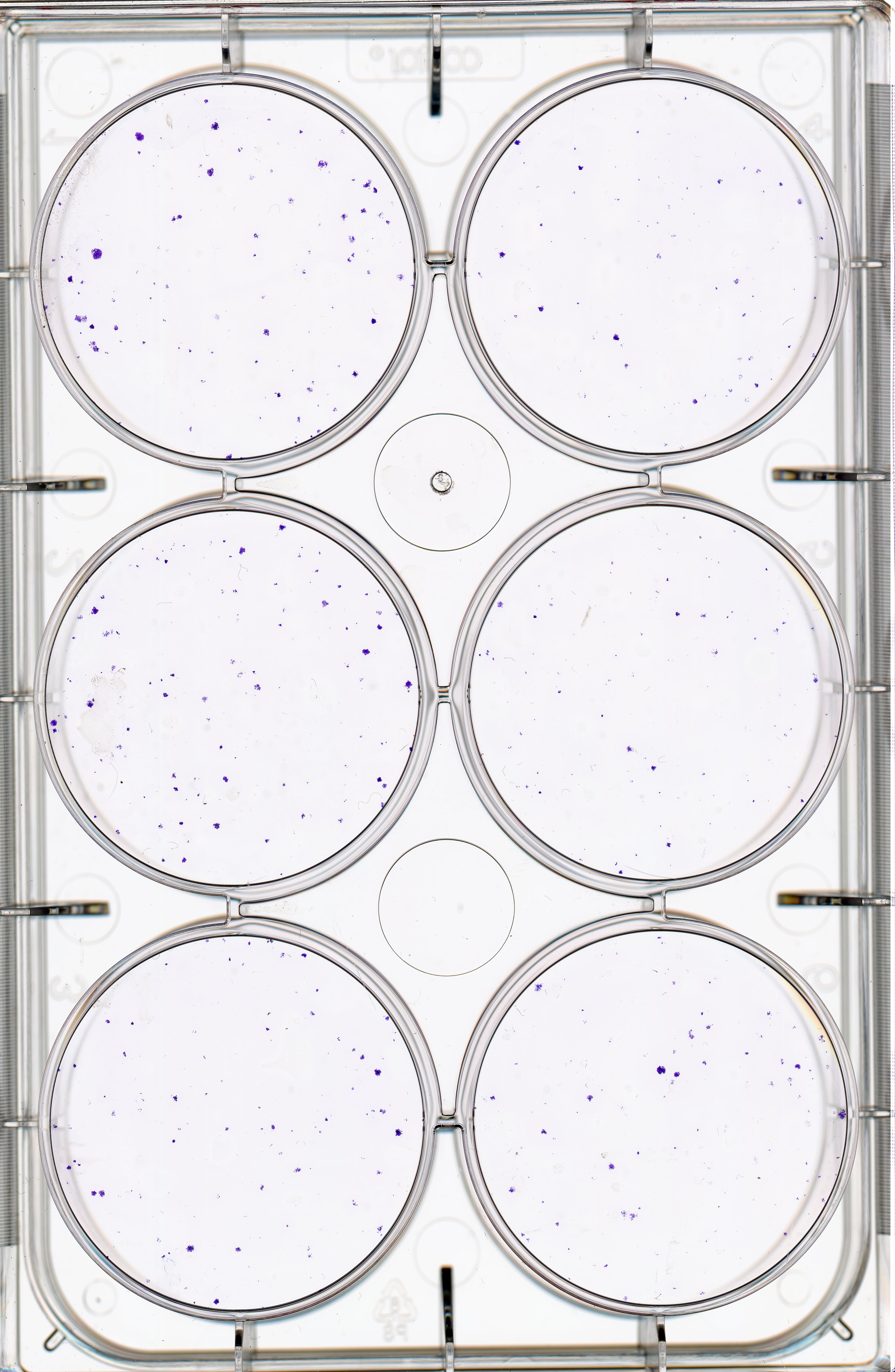

Supplement: Supplementary file 12 — Figure EV4 Source Data [file 44318_2024_108_MOESM12_ESM.zip › EMBOJ-2023-115654_FigEV4_sourcedata/EV4A/E231204 TOPORSsiCtrl 5dC200-400.jpg]

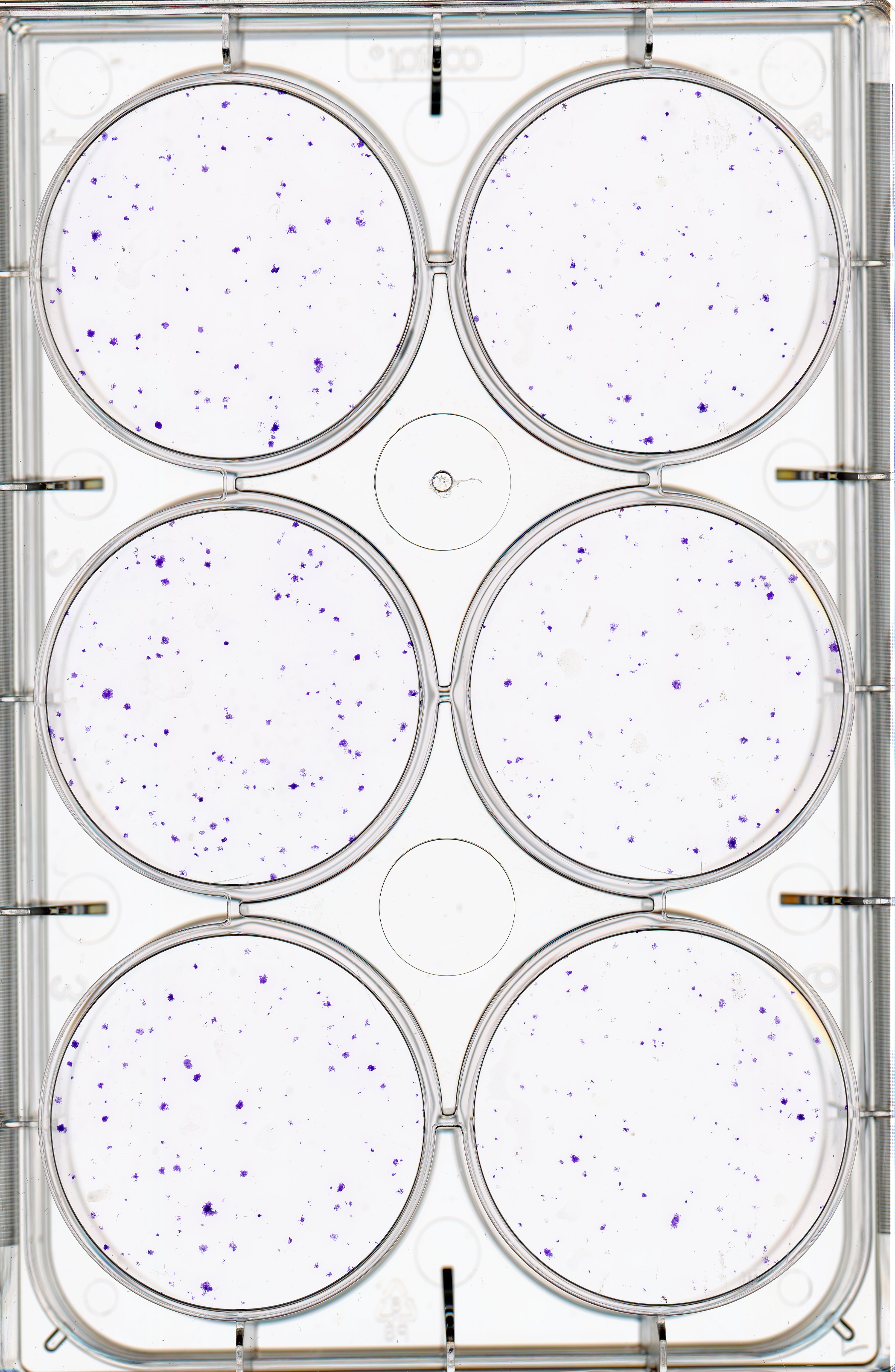

Supplement: Supplementary file 12 — Figure EV4 Source Data [file 44318_2024_108_MOESM12_ESM.zip › EMBOJ-2023-115654_FigEV4_sourcedata/EV4A/E231204 TOPORSsiDNMT1 5dC200-400.jpg]

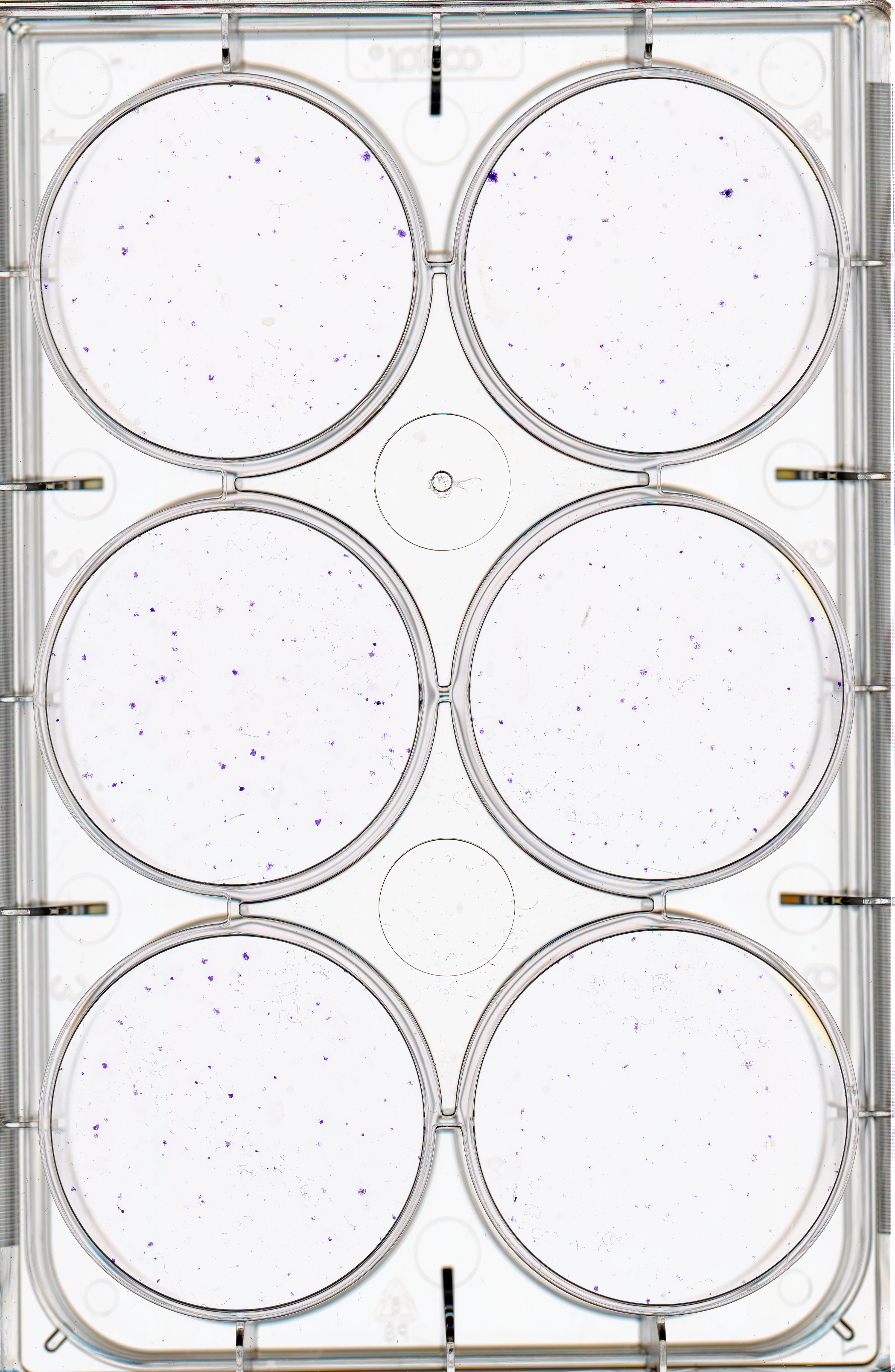

Supplement: Supplementary file 12 — Figure EV4 Source Data [file 44318_2024_108_MOESM12_ESM.zip › EMBOJ-2023-115654_FigEV4_sourcedata/EV4A/E231204 TOPORSsiDNMT1 5dC400-600.jpg]

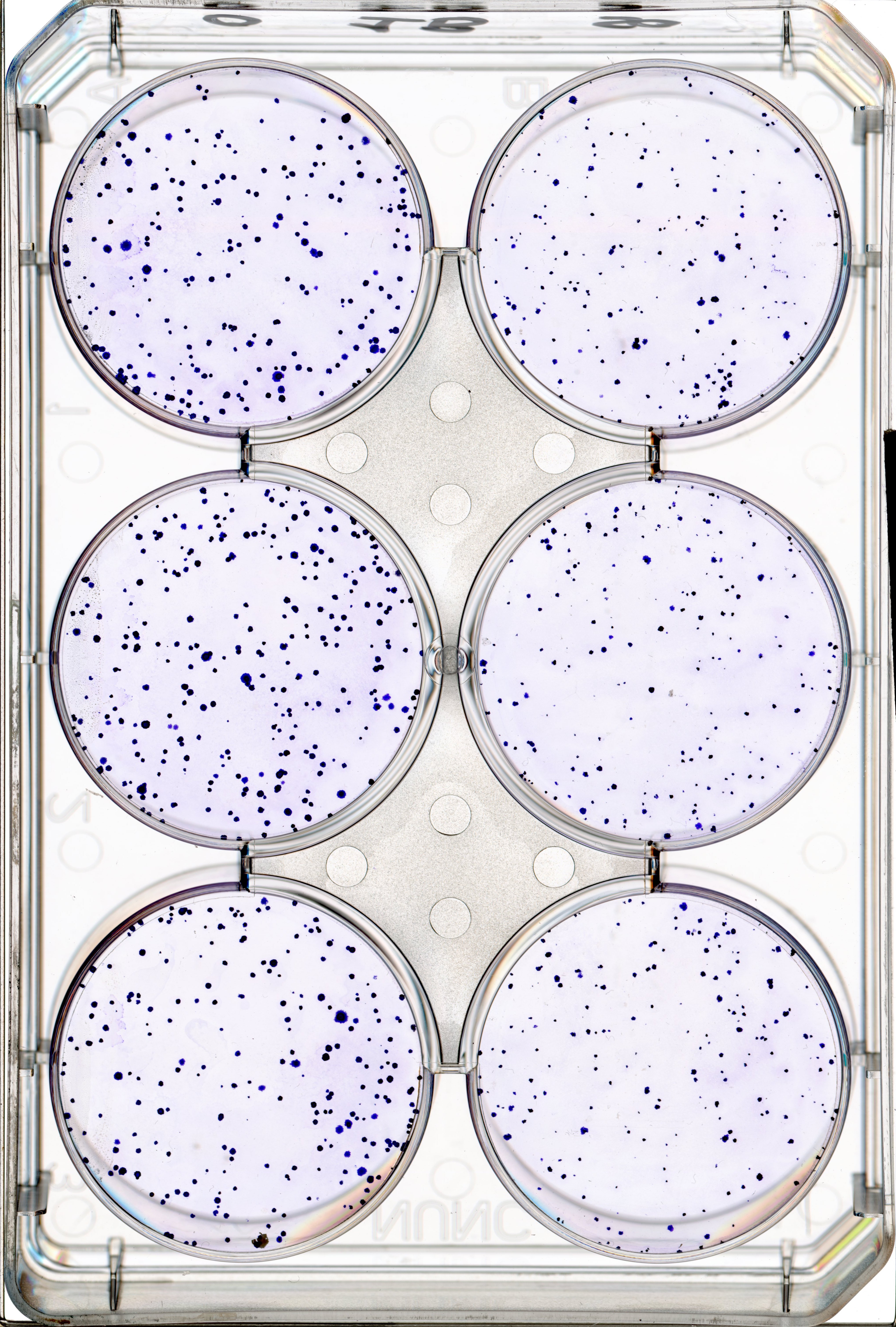

Supplement: Supplementary file 12 — Figure EV4 Source Data [file 44318_2024_108_MOESM12_ESM.zip › EMBOJ-2023-115654_FigEV4_sourcedata/EV4D/nodox_WT_0_50.jpg]

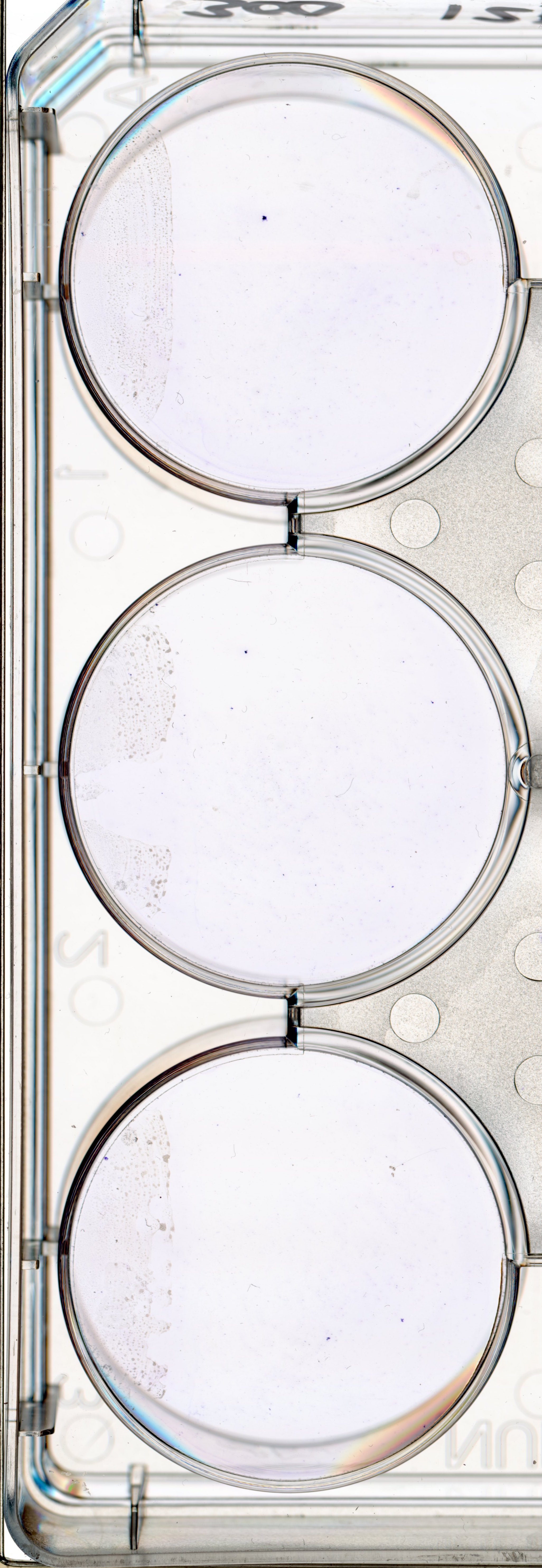

Supplement: Supplementary file 12 — Figure EV4 Source Data [file 44318_2024_108_MOESM12_ESM.zip › EMBOJ-2023-115654_FigEV4_sourcedata/EV4D/DOX-KO-dSIM_300.jpg]

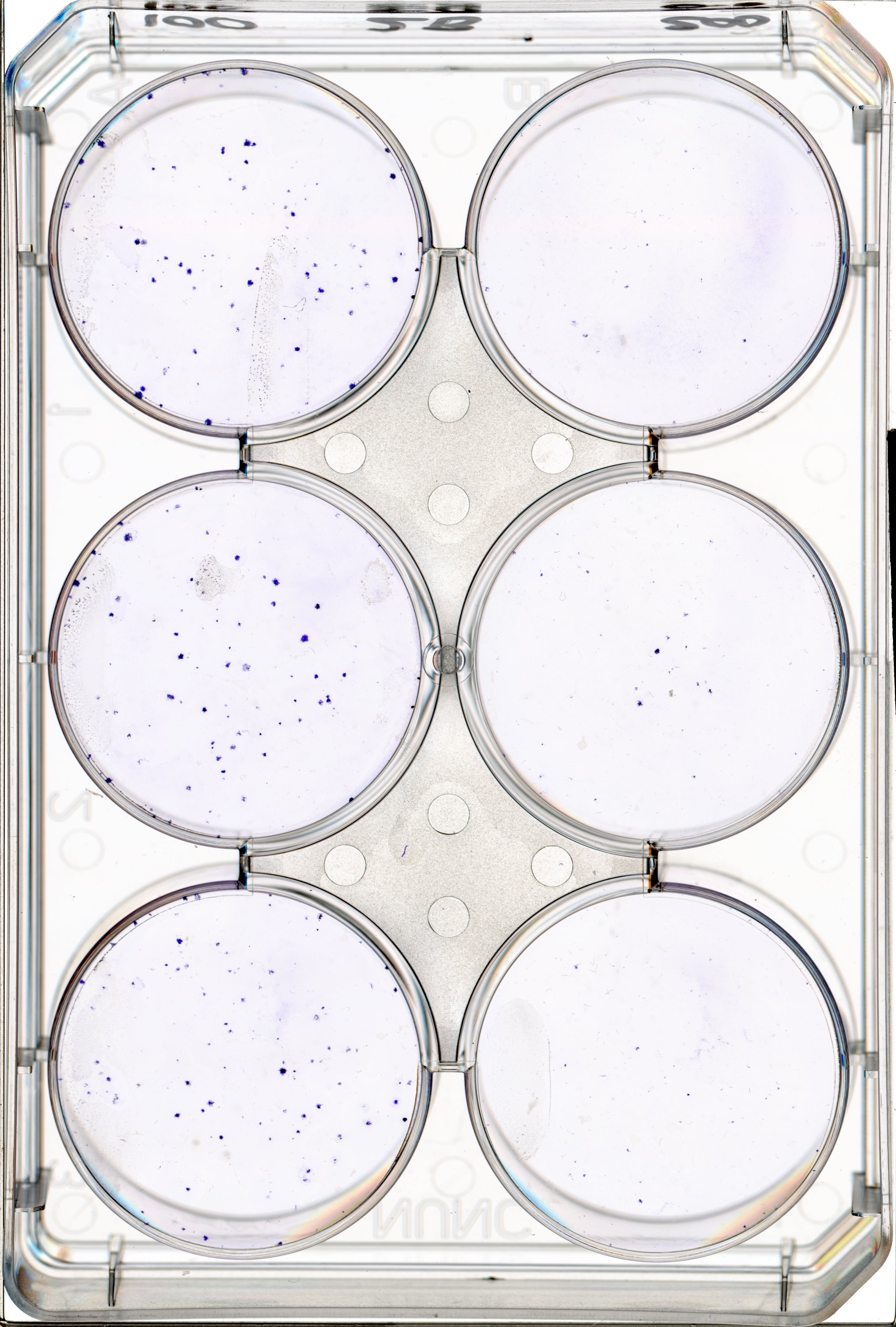

Supplement: Supplementary file 12 — Figure EV4 Source Data [file 44318_2024_108_MOESM12_ESM.zip › EMBOJ-2023-115654_FigEV4_sourcedata/EV4D/nodox_KOev_100_200.jpg]

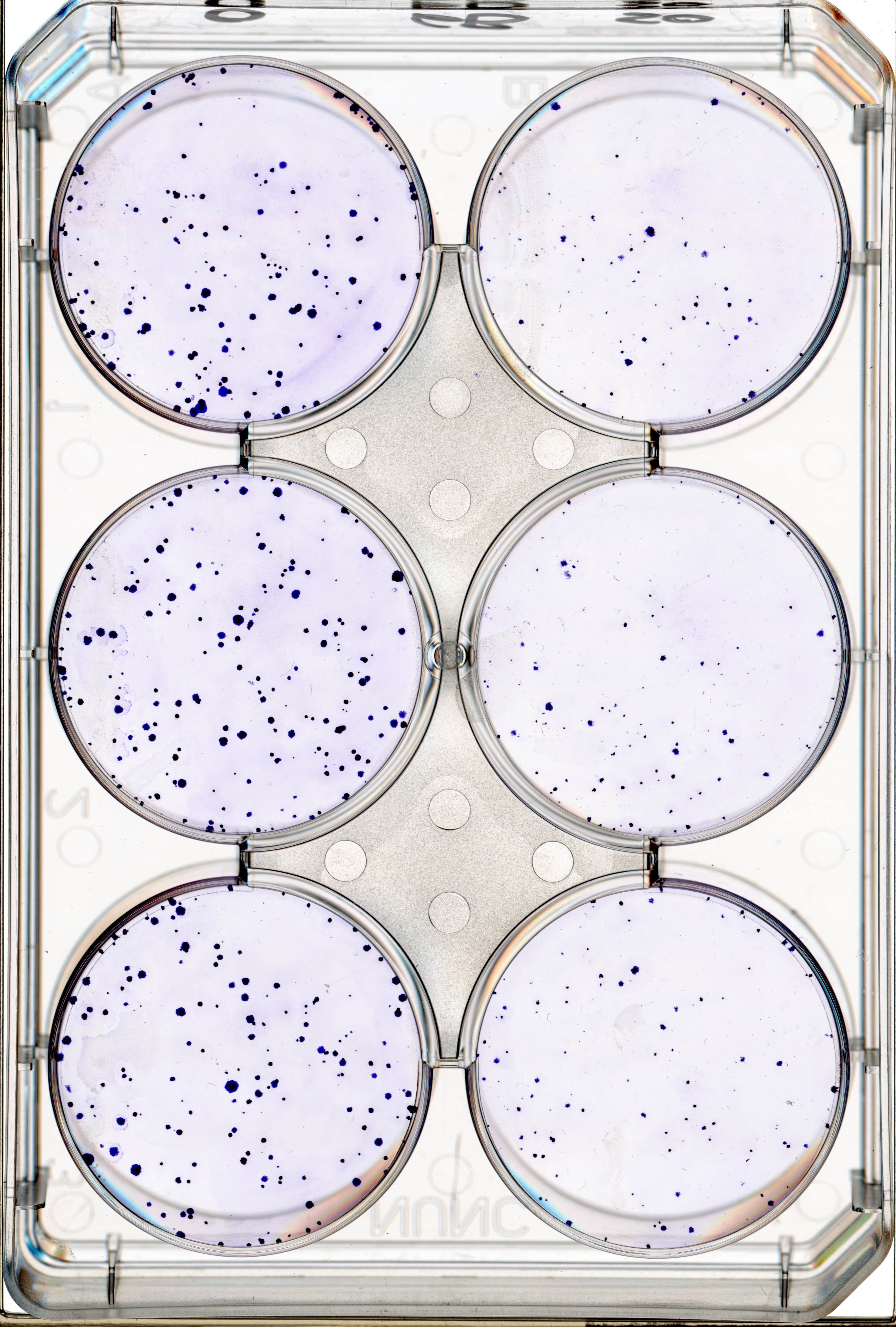

Supplement: Supplementary file 12 — Figure EV4 Source Data [file 44318_2024_108_MOESM12_ESM.zip › EMBOJ-2023-115654_FigEV4_sourcedata/EV4D/nodox_KO-dSIM_0_50.jpg]

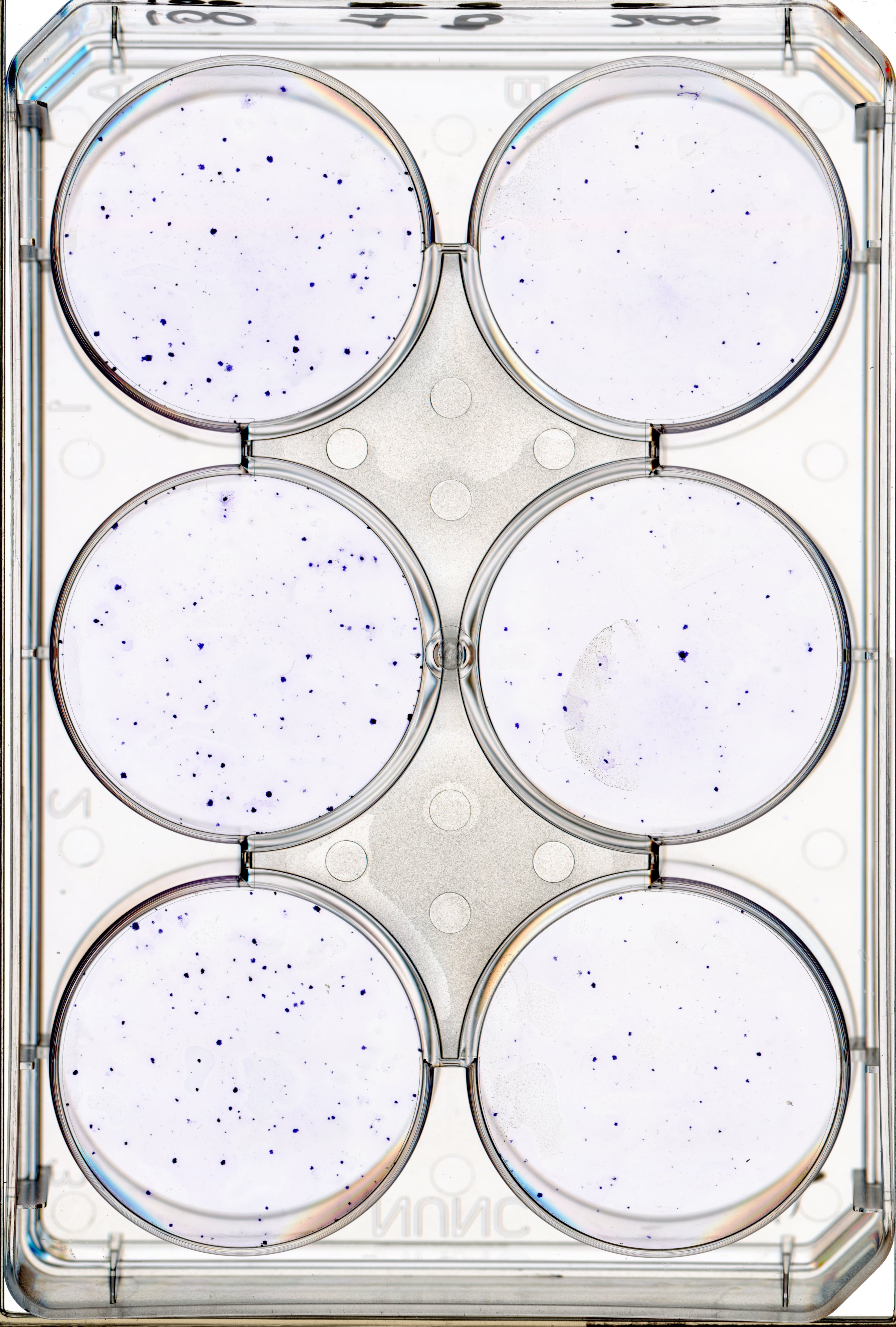

Supplement: Supplementary file 12 — Figure EV4 Source Data [file 44318_2024_108_MOESM12_ESM.zip › EMBOJ-2023-115654_FigEV4_sourcedata/EV4D/nodox_WT_100_200.jpg]

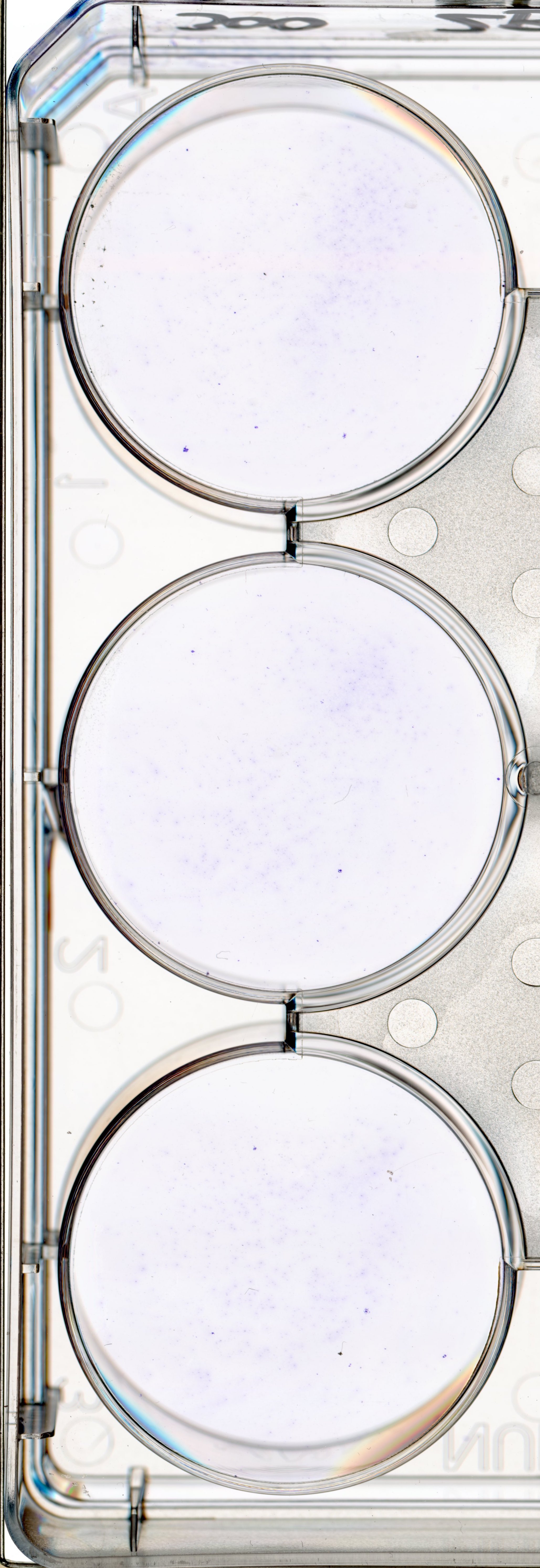

Supplement: Supplementary file 12 — Figure EV4 Source Data [file 44318_2024_108_MOESM12_ESM.zip › EMBOJ-2023-115654_FigEV4_sourcedata/EV4D/nodox_KOev_300.jpg]

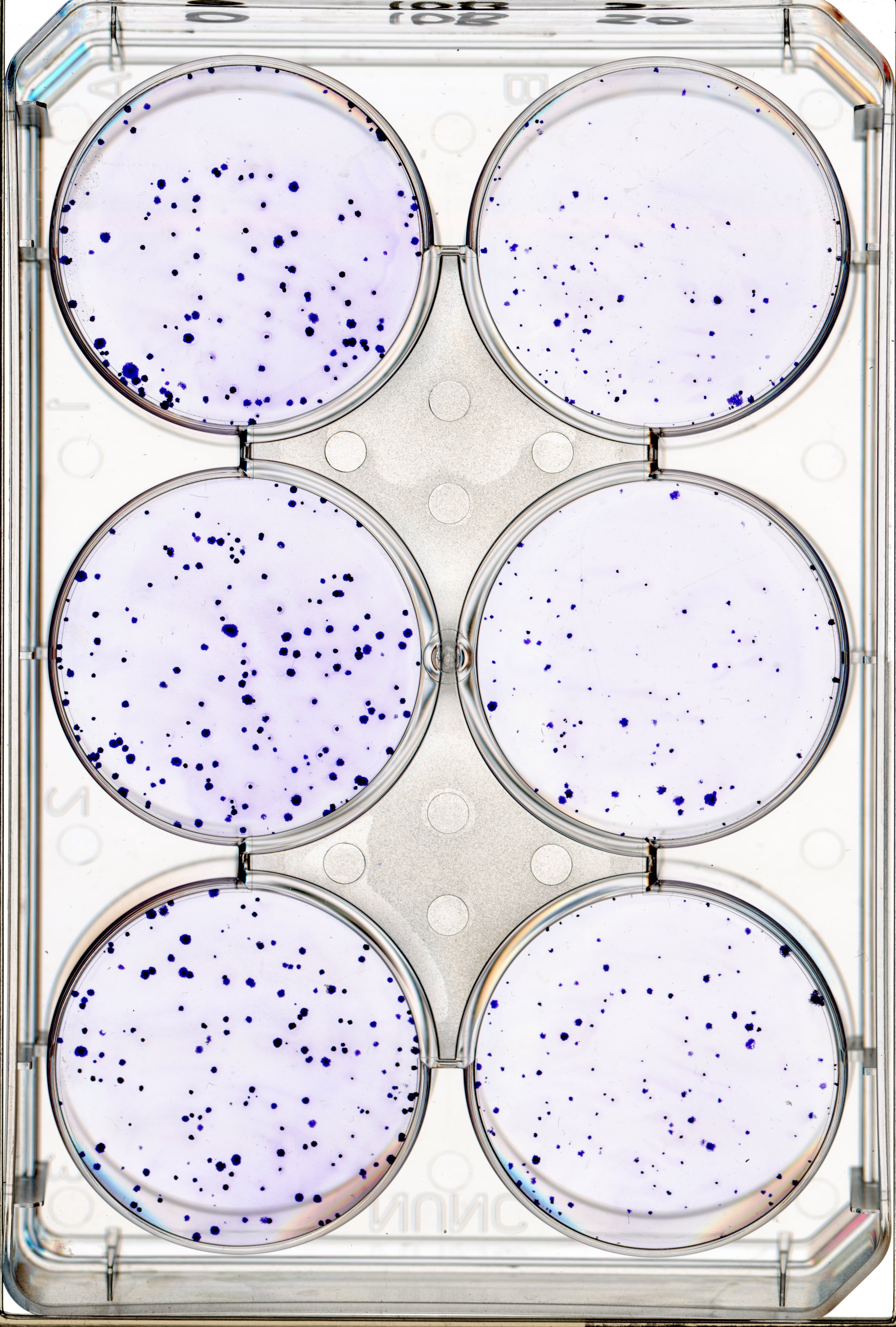

Supplement: Supplementary file 12 — Figure EV4 Source Data [file 44318_2024_108_MOESM12_ESM.zip › EMBOJ-2023-115654_FigEV4_sourcedata/EV4D/DOX-KO-CCAA_0_50.jpg]

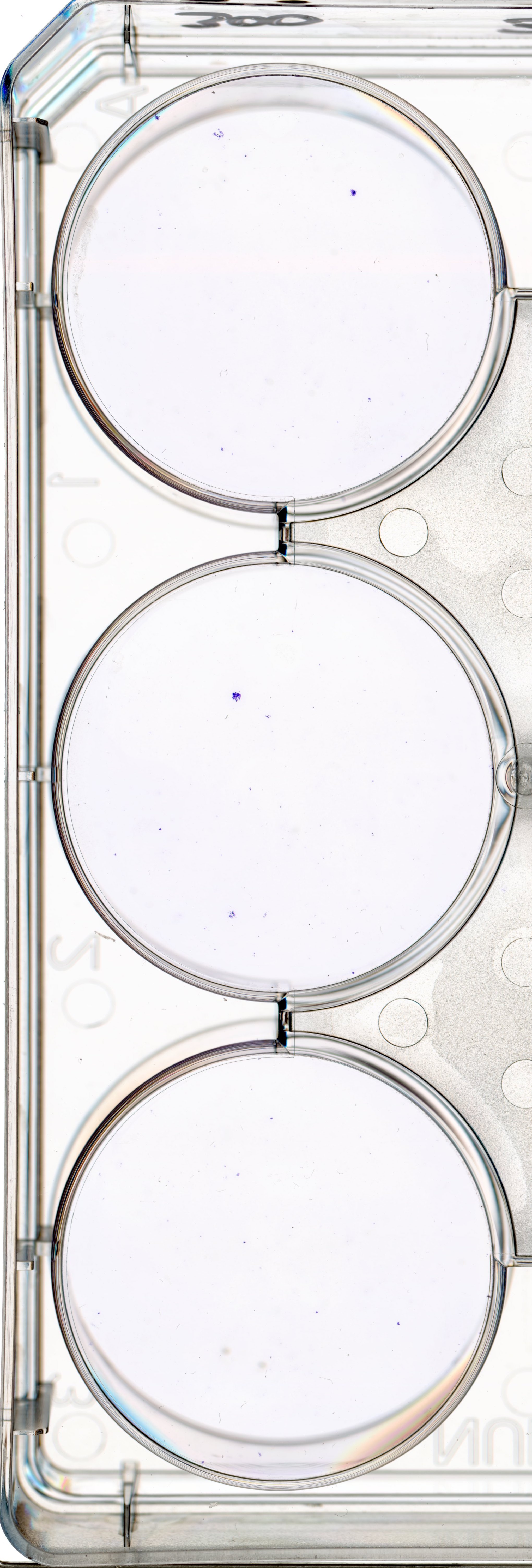

Supplement: Supplementary file 12 — Figure EV4 Source Data [file 44318_2024_108_MOESM12_ESM.zip › EMBOJ-2023-115654_FigEV4_sourcedata/EV4D/nodox_KO-WT_300.jpg]

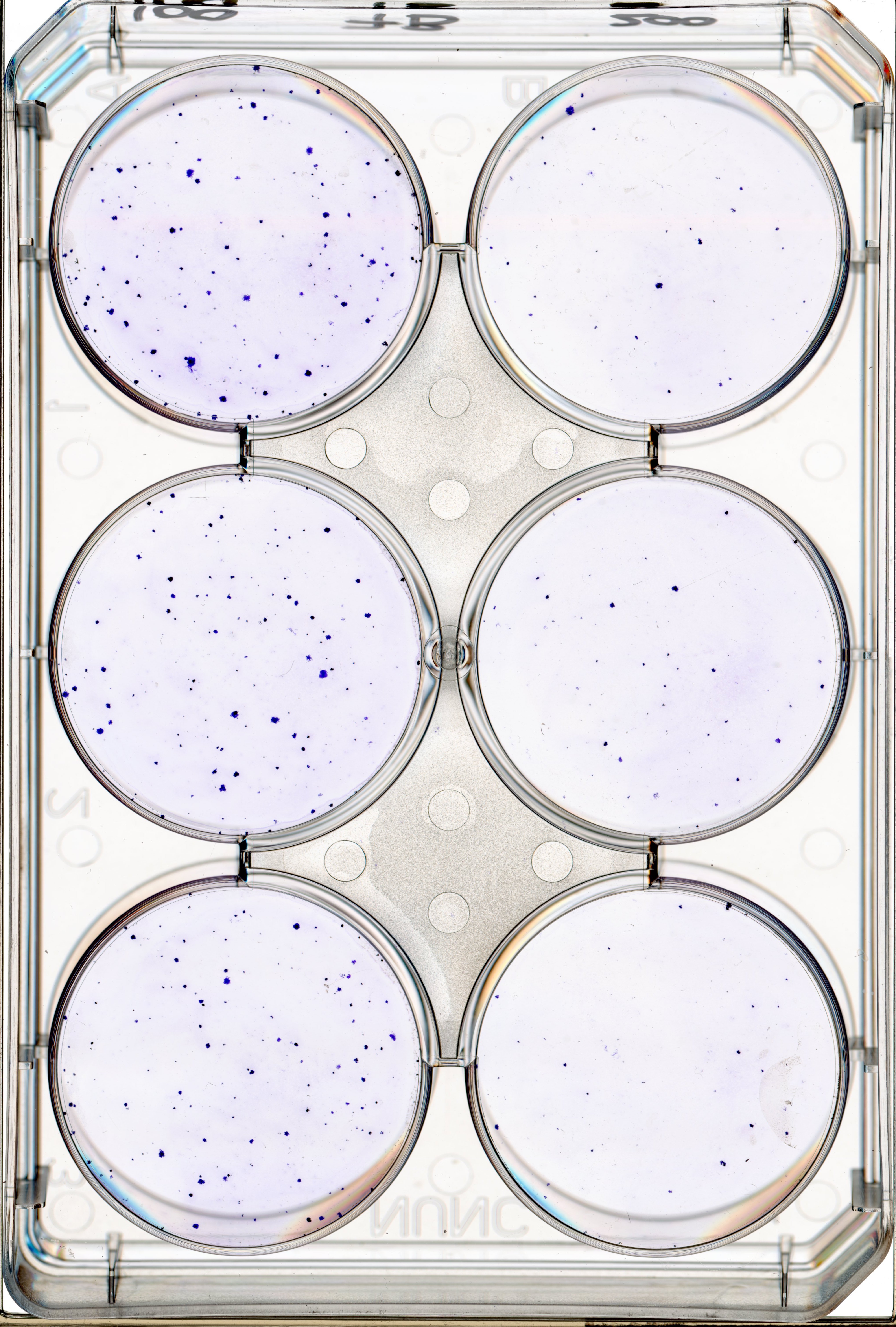

Supplement: Supplementary file 12 — Figure EV4 Source Data [file 44318_2024_108_MOESM12_ESM.zip › EMBOJ-2023-115654_FigEV4_sourcedata/EV4D/DOX-WT_100_200.jpg]

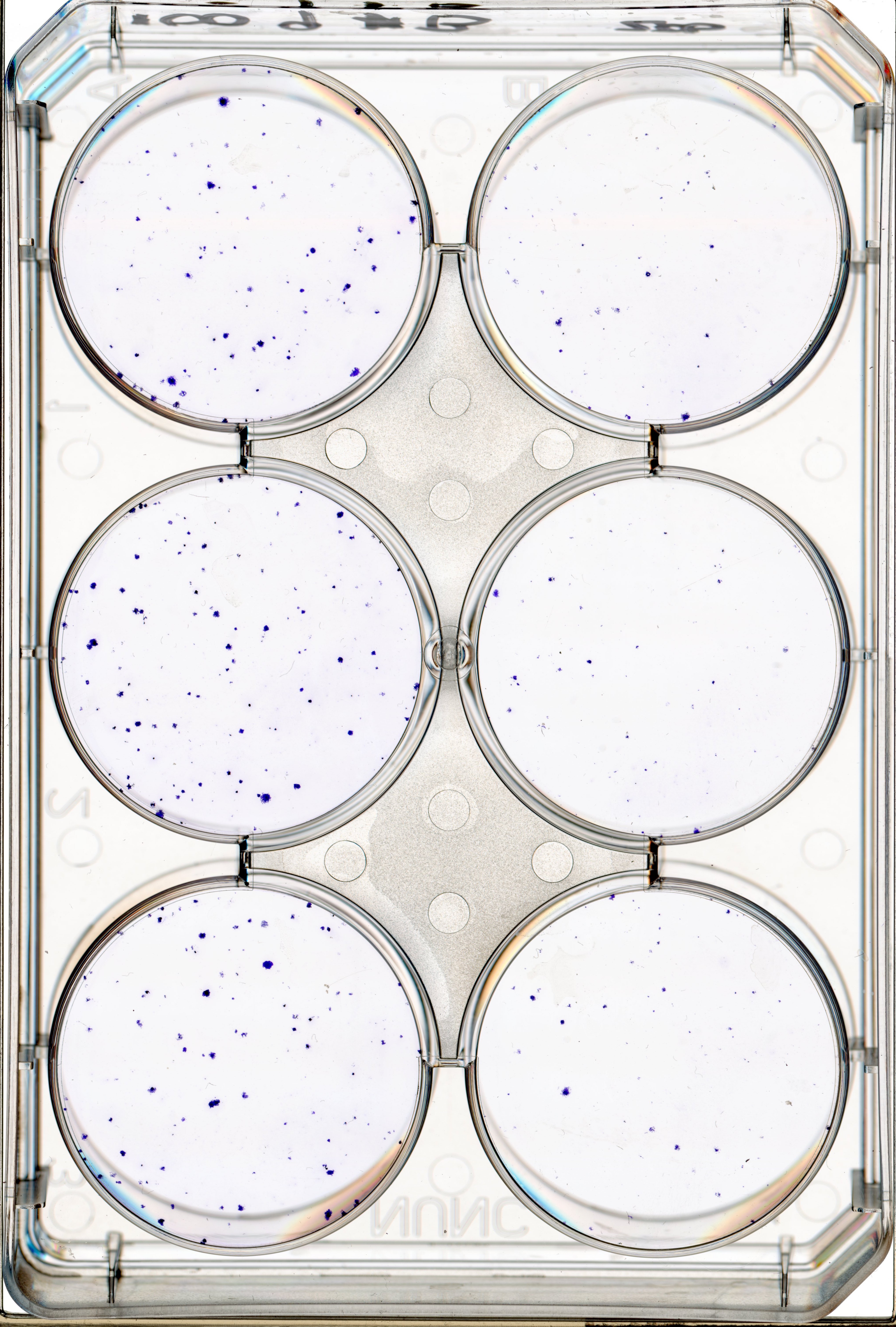

Supplement: Supplementary file 12 — Figure EV4 Source Data [file 44318_2024_108_MOESM12_ESM.zip › EMBOJ-2023-115654_FigEV4_sourcedata/EV4D/DOX-KO-WT_100_200.jpg]

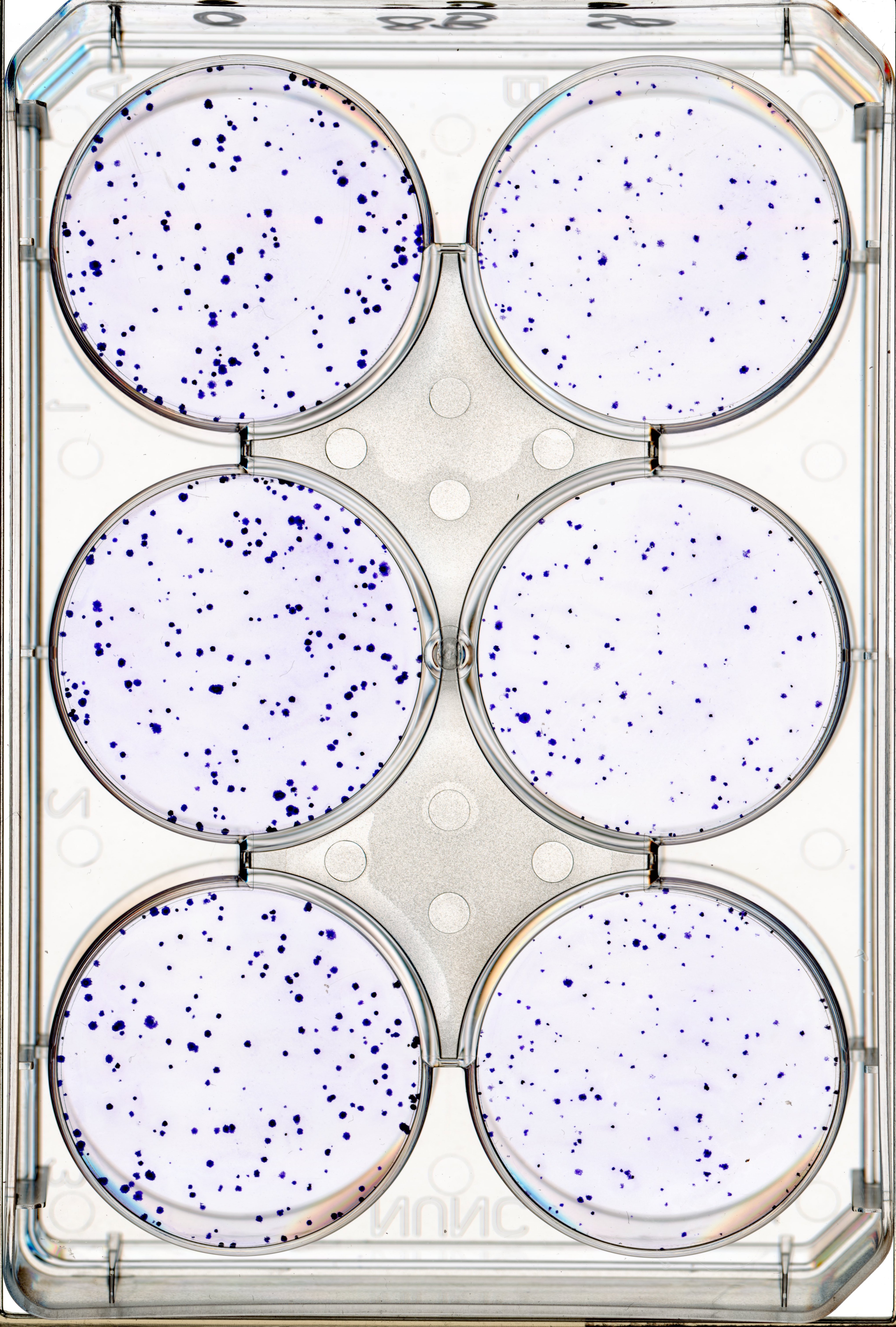

Supplement: Supplementary file 12 — Figure EV4 Source Data [file 44318_2024_108_MOESM12_ESM.zip › EMBOJ-2023-115654_FigEV4_sourcedata/EV4D/DOX-KO-ev_0_50.jpg]

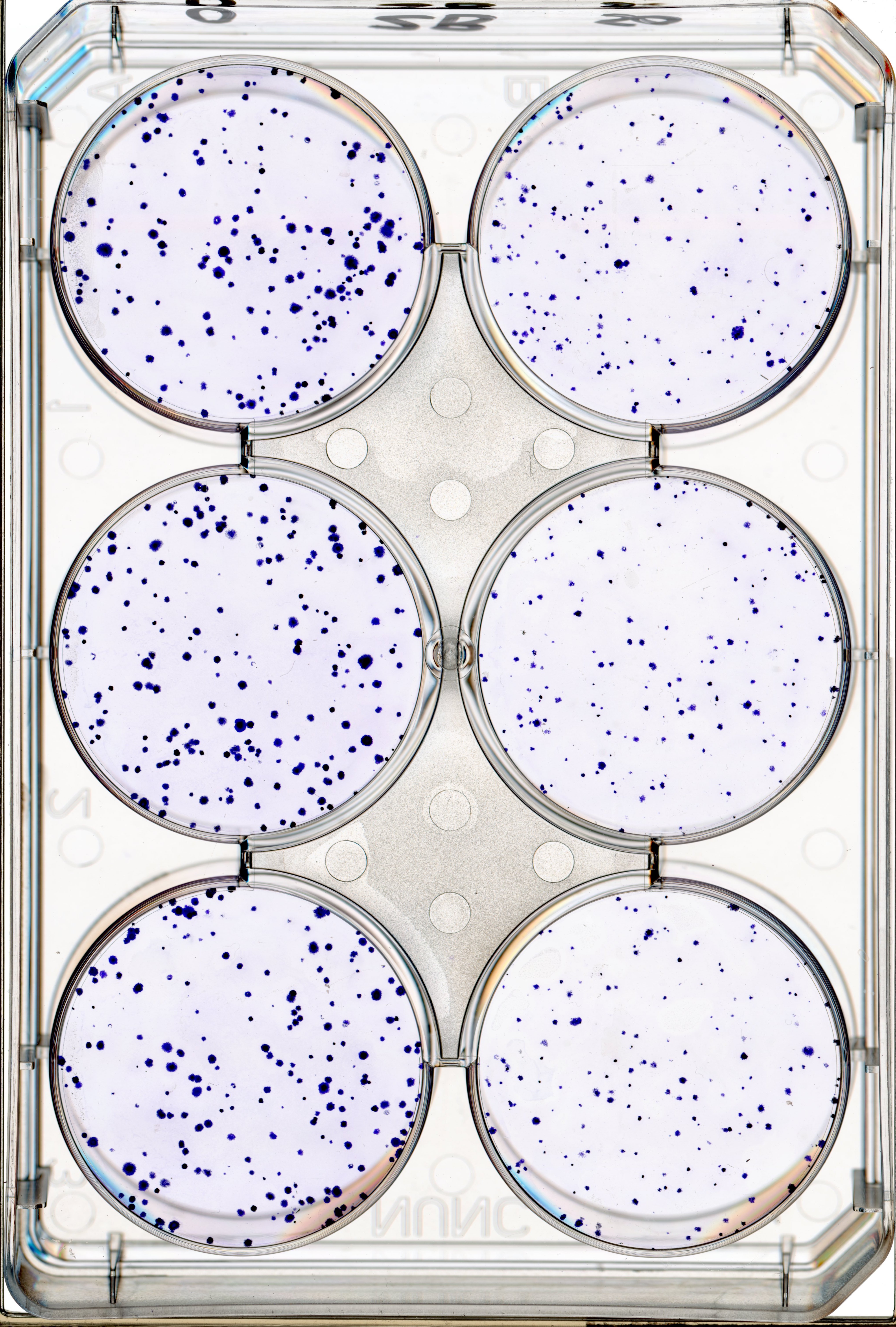

Supplement: Supplementary file 12 — Figure EV4 Source Data [file 44318_2024_108_MOESM12_ESM.zip › EMBOJ-2023-115654_FigEV4_sourcedata/EV4D/nodox_KOev_0_50.jpg]

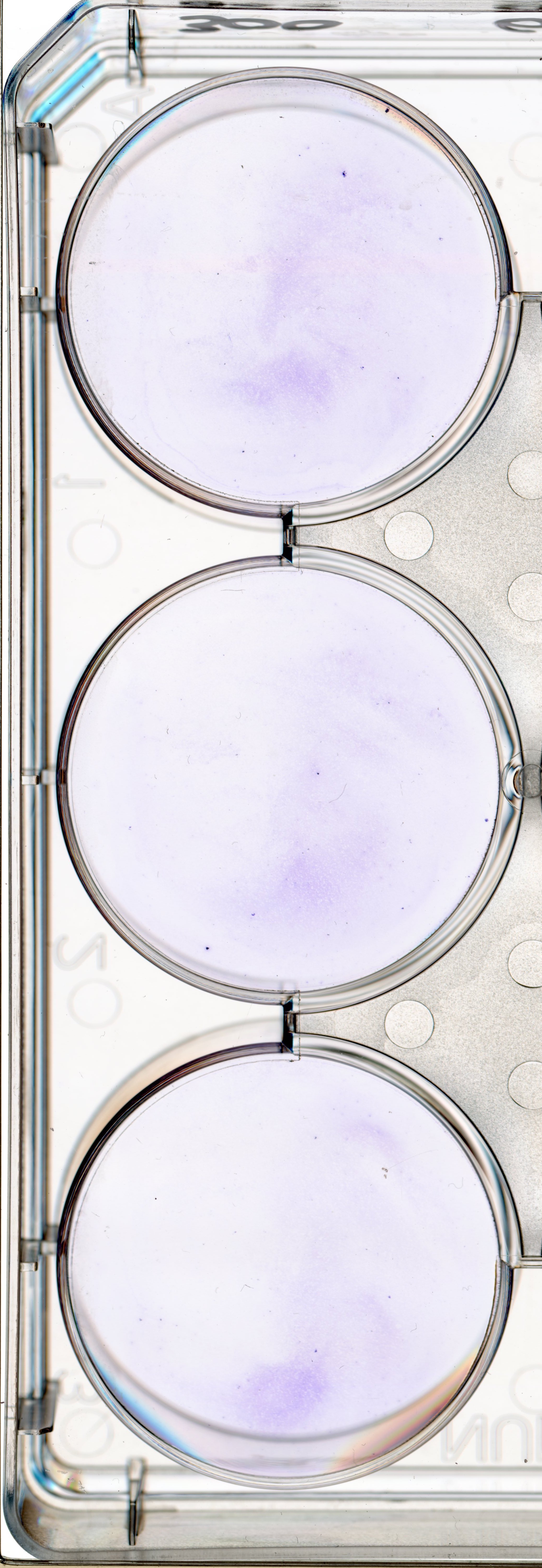

Supplement: Supplementary file 12 — Figure EV4 Source Data [file 44318_2024_108_MOESM12_ESM.zip › EMBOJ-2023-115654_FigEV4_sourcedata/EV4D/nodox_KO-dSIM_300.jpg]

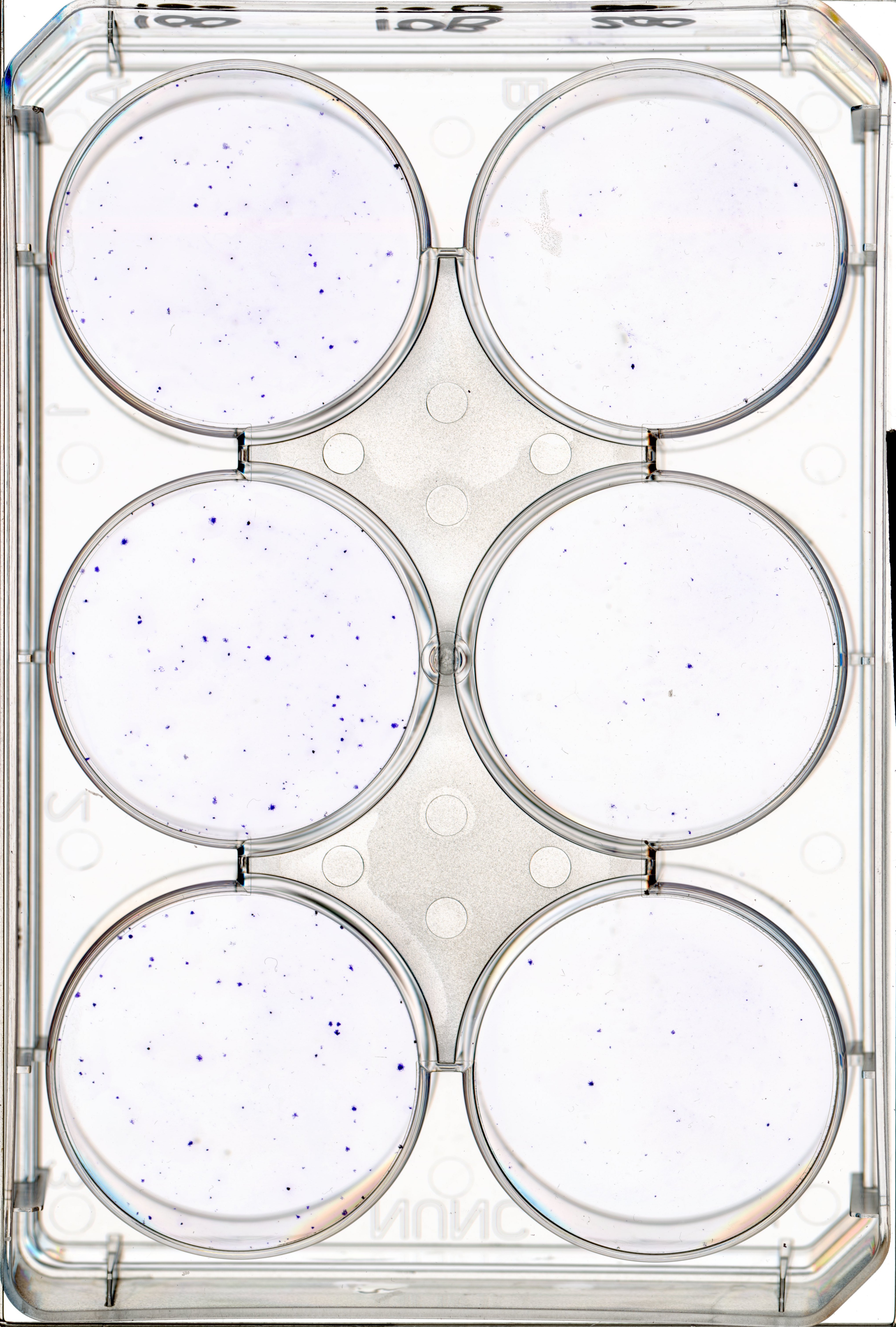

Supplement: Supplementary file 12 — Figure EV4 Source Data [file 44318_2024_108_MOESM12_ESM.zip › EMBOJ-2023-115654_FigEV4_sourcedata/EV4D/DOX-KO-CCAA_100_200.jpg]

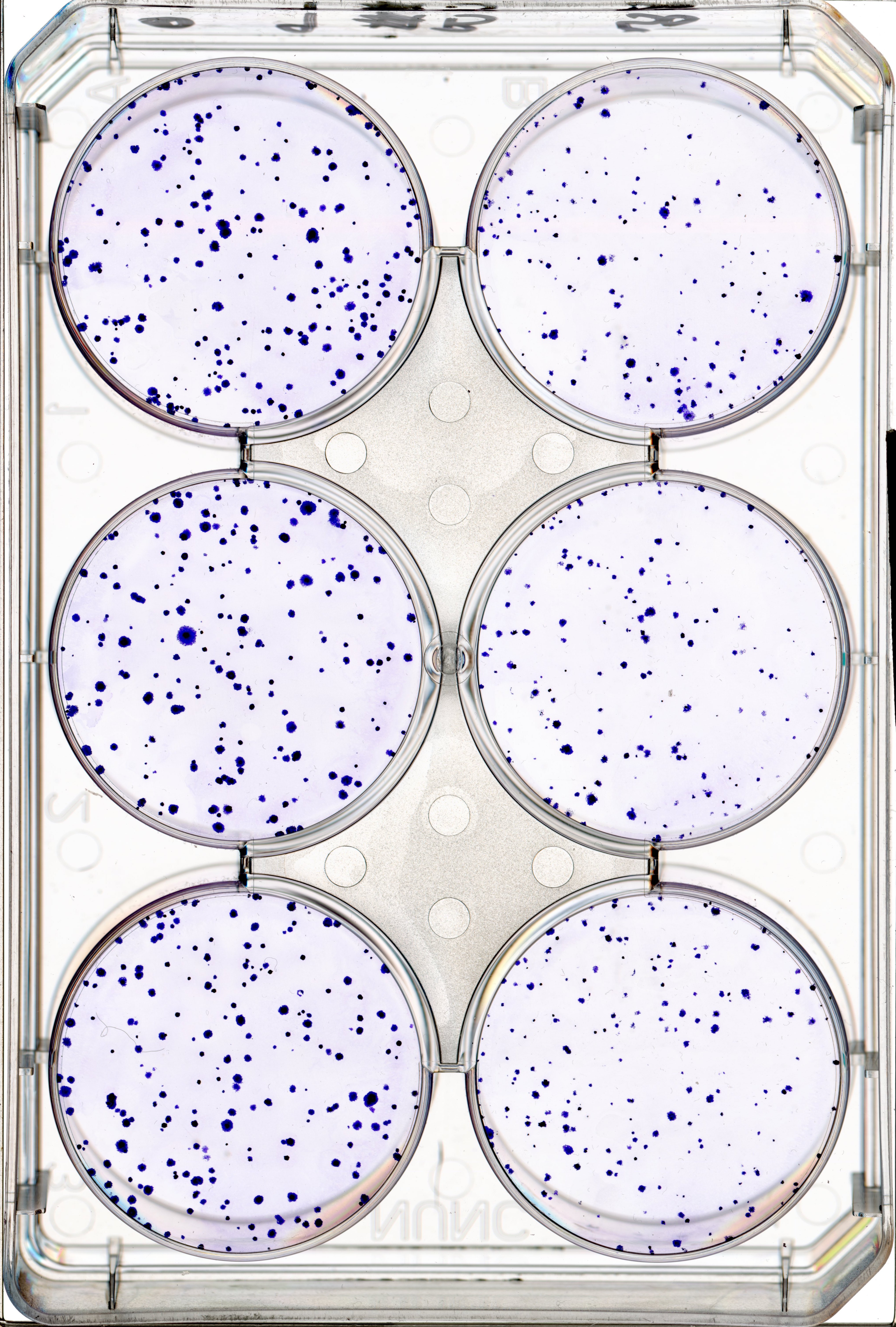

Supplement: Supplementary file 12 — Figure EV4 Source Data [file 44318_2024_108_MOESM12_ESM.zip › EMBOJ-2023-115654_FigEV4_sourcedata/EV4D/DOX-KO-WT_0_50.jpg]

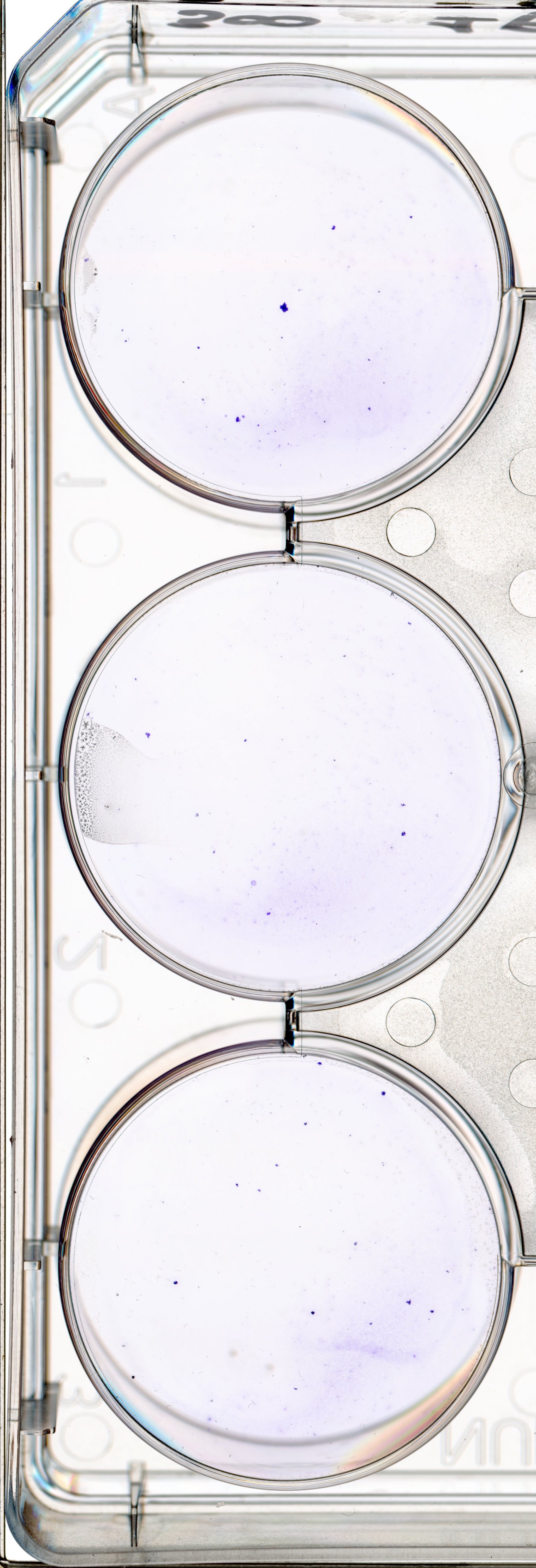

Supplement: Supplementary file 12 — Figure EV4 Source Data [file 44318_2024_108_MOESM12_ESM.zip › EMBOJ-2023-115654_FigEV4_sourcedata/EV4D/nodox_WT_300.jpg]

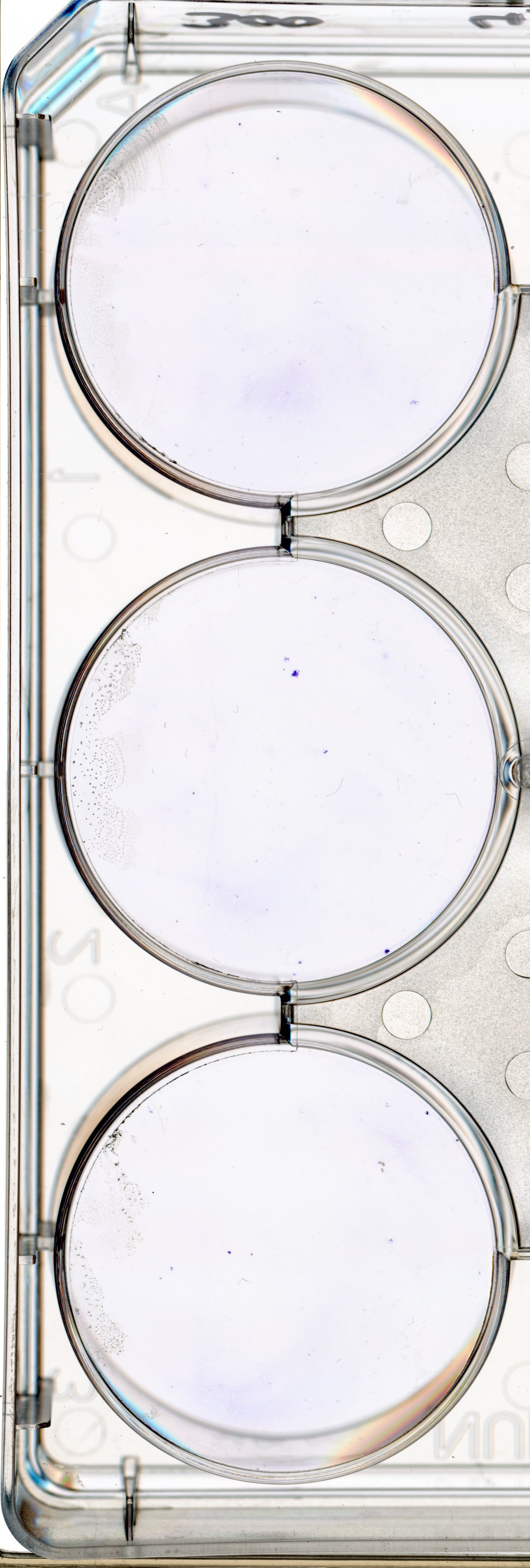

Supplement: Supplementary file 12 — Figure EV4 Source Data [file 44318_2024_108_MOESM12_ESM.zip › EMBOJ-2023-115654_FigEV4_sourcedata/EV4D/nodox_KO-CCAA_300.jpg]

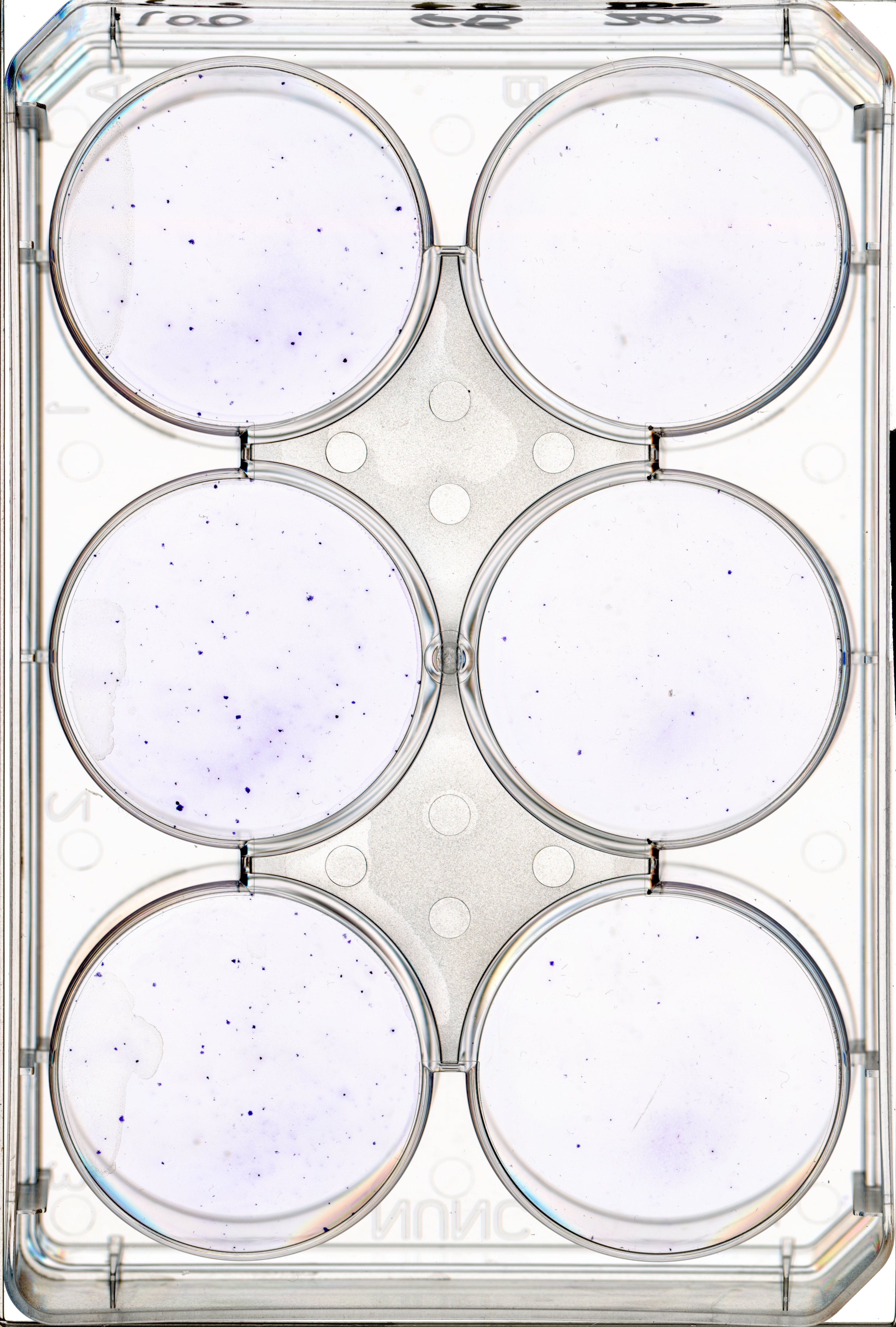

Supplement: Supplementary file 12 — Figure EV4 Source Data [file 44318_2024_108_MOESM12_ESM.zip › EMBOJ-2023-115654_FigEV4_sourcedata/EV4D/nodox_KO-dSIM_100_200.jpg]

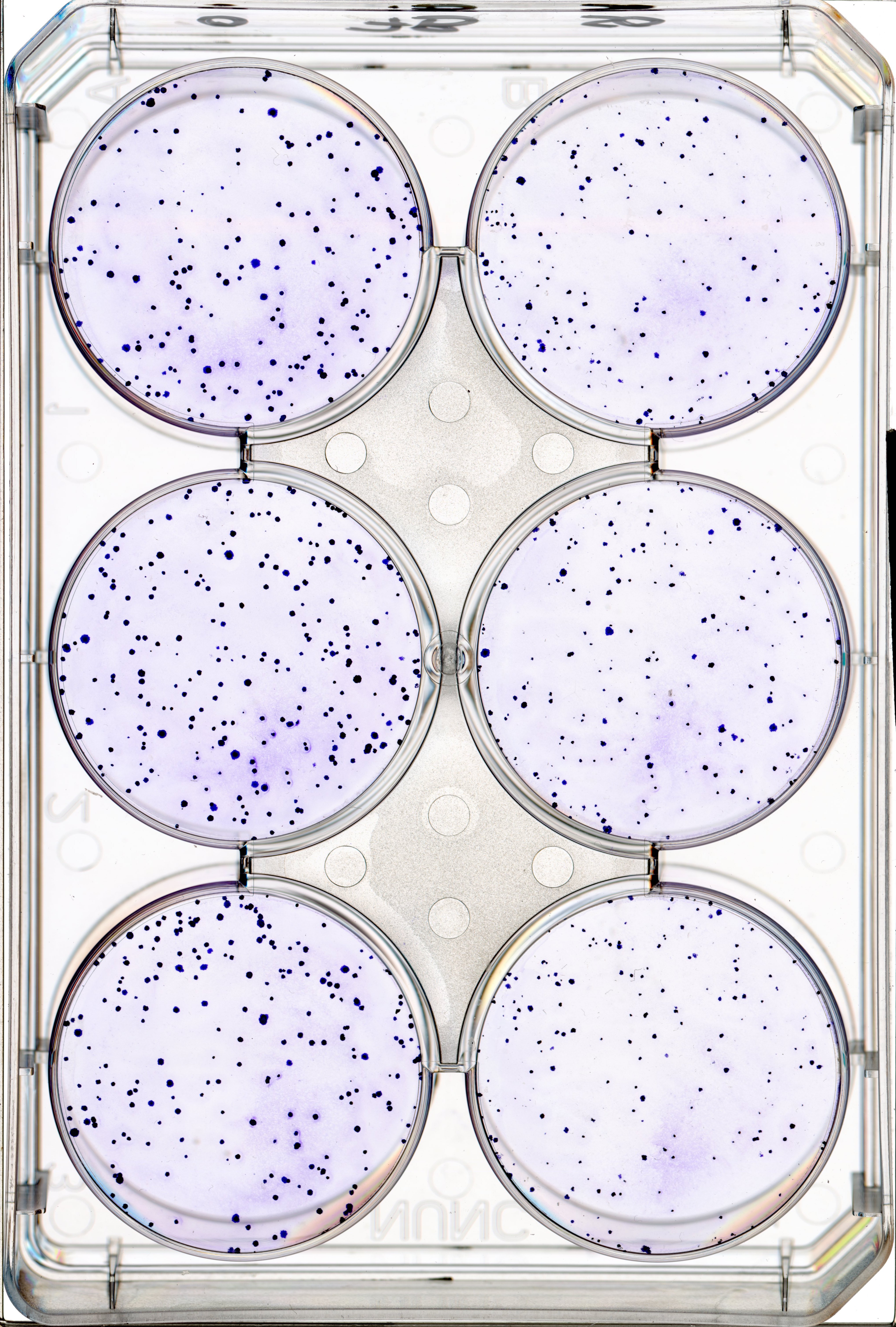

Supplement: Supplementary file 12 — Figure EV4 Source Data [file 44318_2024_108_MOESM12_ESM.zip › EMBOJ-2023-115654_FigEV4_sourcedata/EV4D/DOX-WT_0_50.jpg]

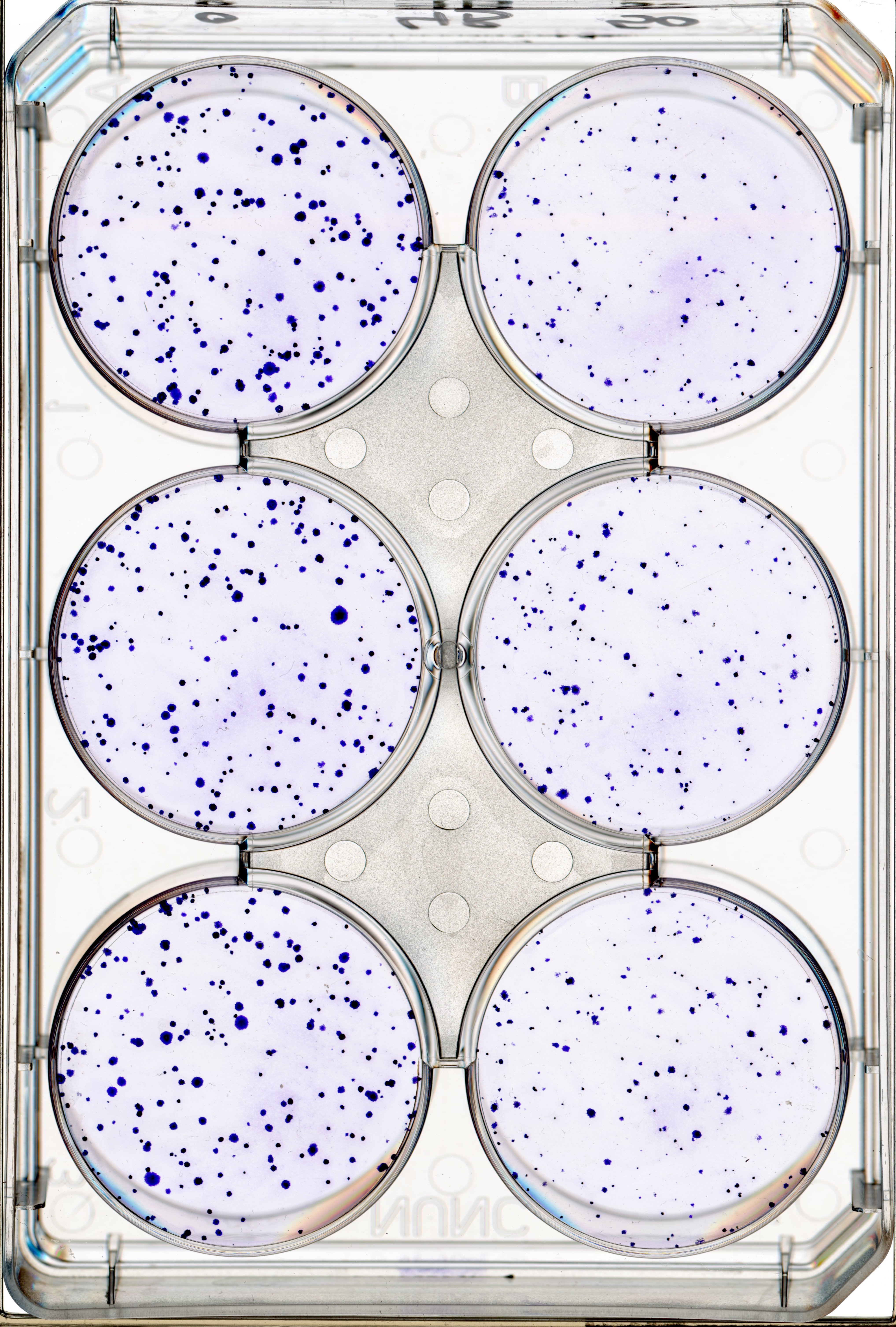

Supplement: Supplementary file 12 — Figure EV4 Source Data [file 44318_2024_108_MOESM12_ESM.zip › EMBOJ-2023-115654_FigEV4_sourcedata/EV4D/nodox_KO-CCAA_0_50.jpg]

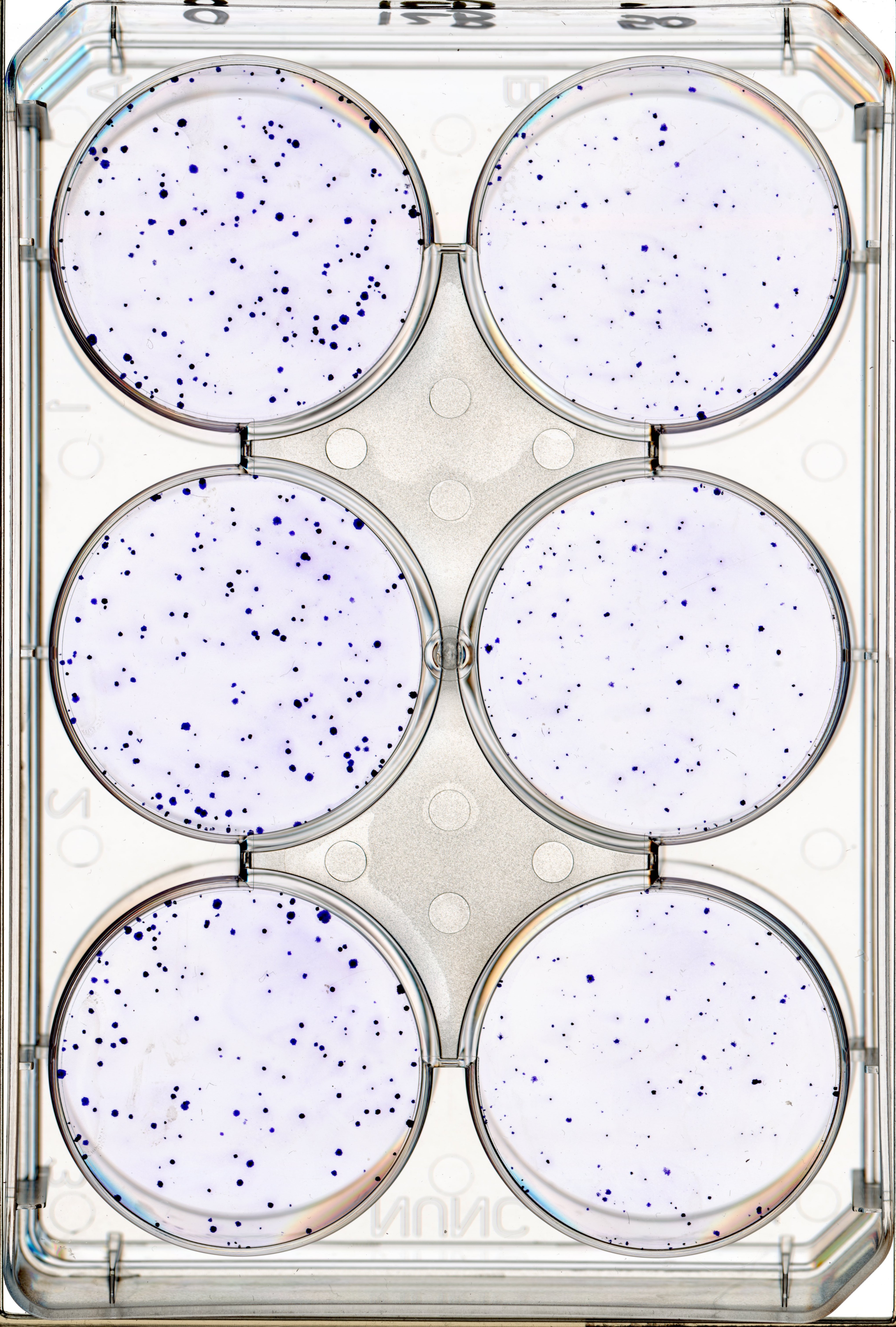

Supplement: Supplementary file 12 — Figure EV4 Source Data [file 44318_2024_108_MOESM12_ESM.zip › EMBOJ-2023-115654_FigEV4_sourcedata/EV4D/DOX-KO-dSIM_0_50.jpg]

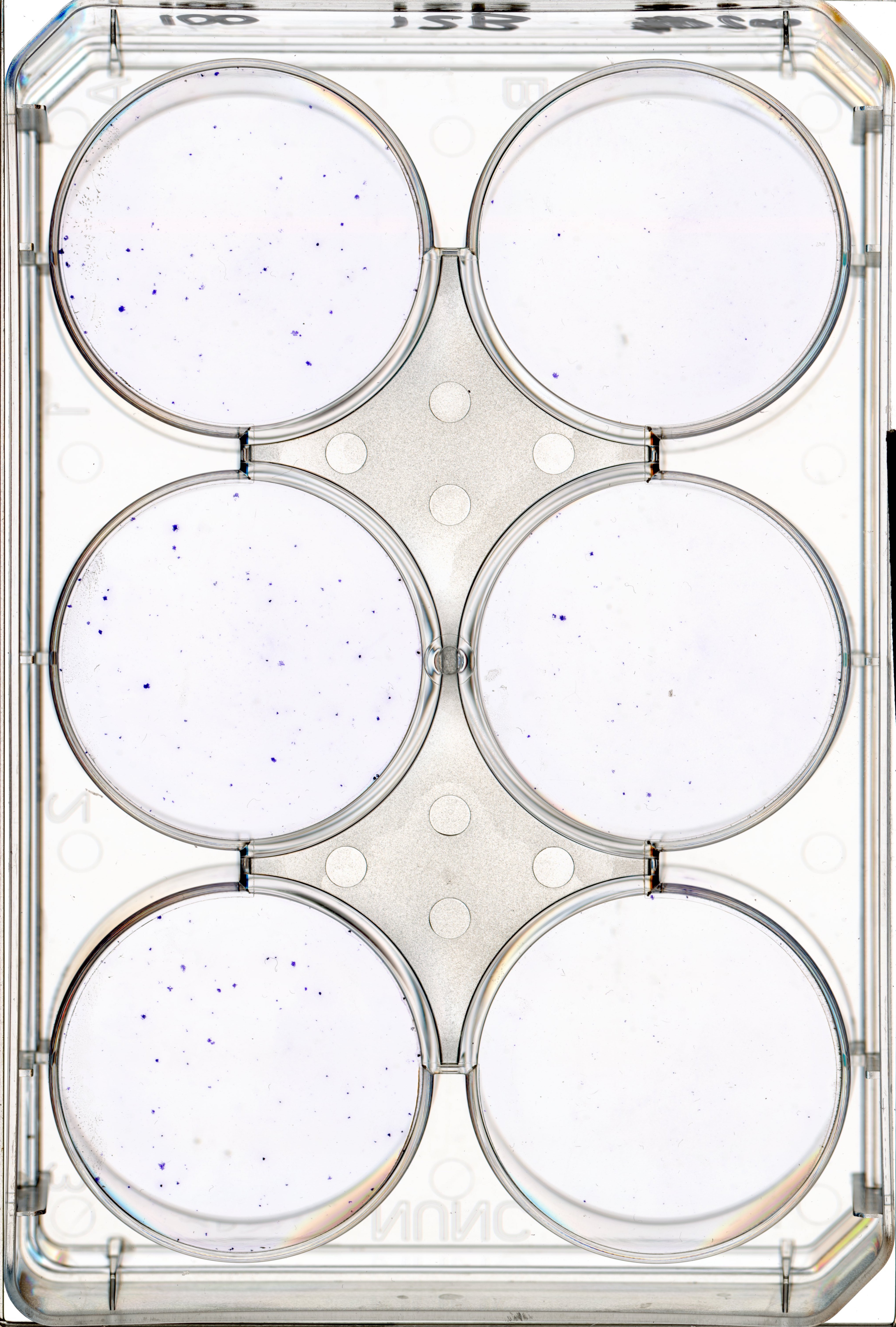

Supplement: Supplementary file 12 — Figure EV4 Source Data [file 44318_2024_108_MOESM12_ESM.zip › EMBOJ-2023-115654_FigEV4_sourcedata/EV4D/DOX-KO-dSIM_100_200.jpg]

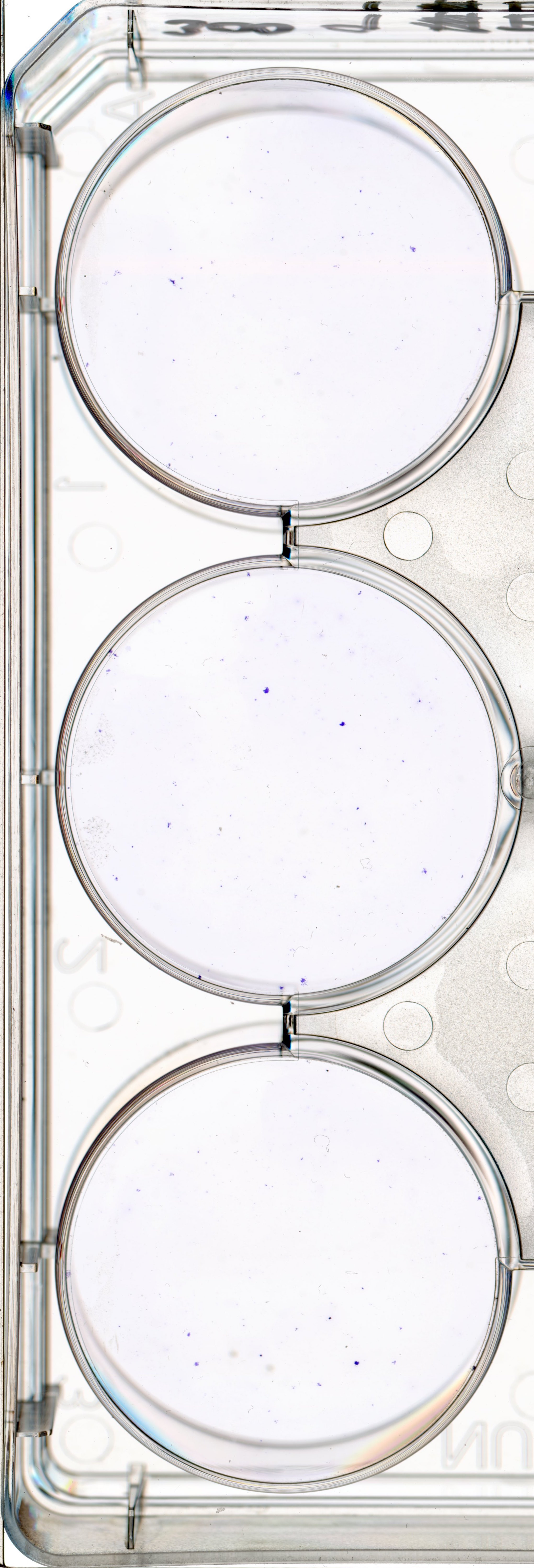

Supplement: Supplementary file 12 — Figure EV4 Source Data [file 44318_2024_108_MOESM12_ESM.zip › EMBOJ-2023-115654_FigEV4_sourcedata/EV4D/DOX-KO-WT_300.jpg]

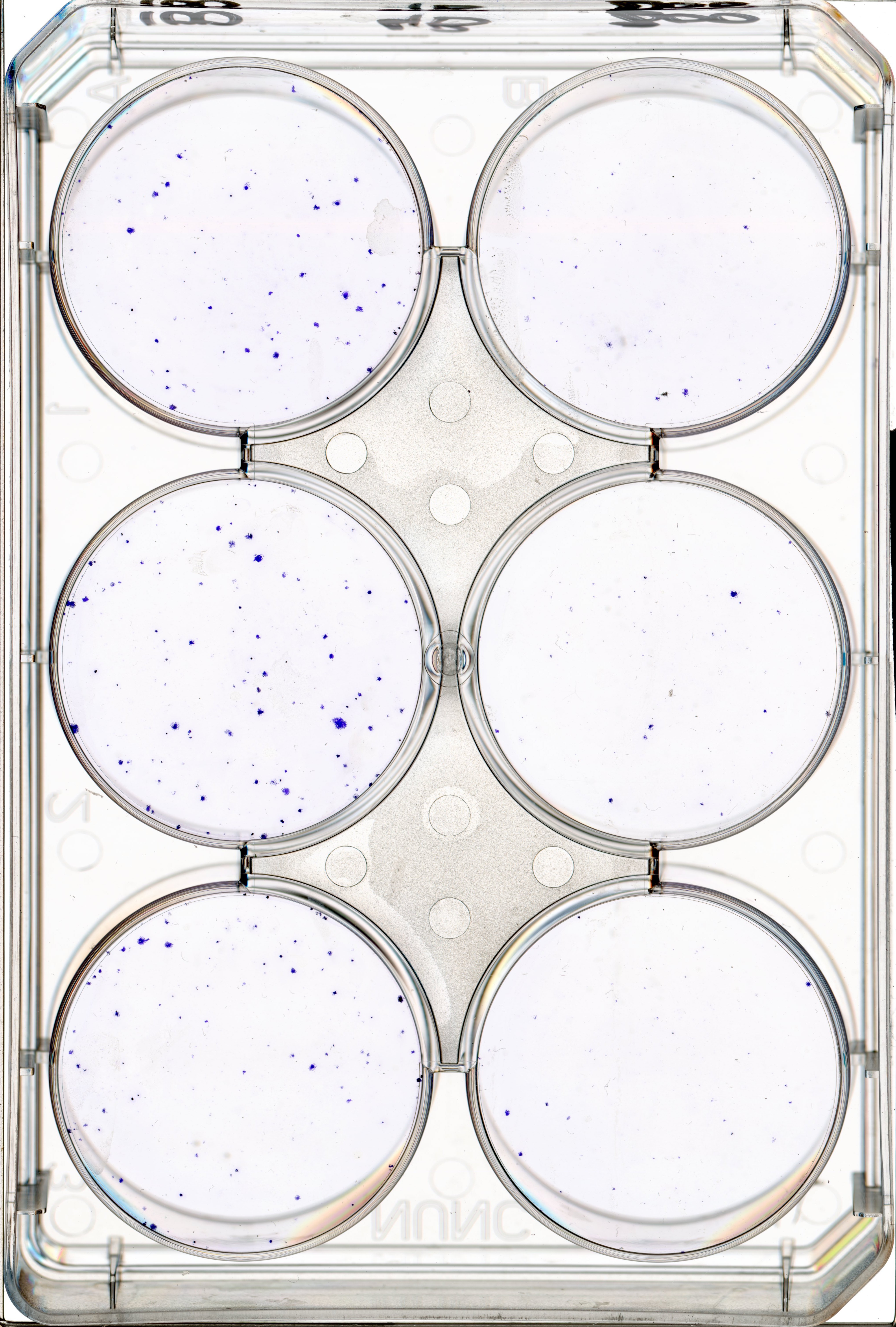

Supplement: Supplementary file 12 — Figure EV4 Source Data [file 44318_2024_108_MOESM12_ESM.zip › EMBOJ-2023-115654_FigEV4_sourcedata/EV4D/nodox_KO-CCAA_100_200.jpg]

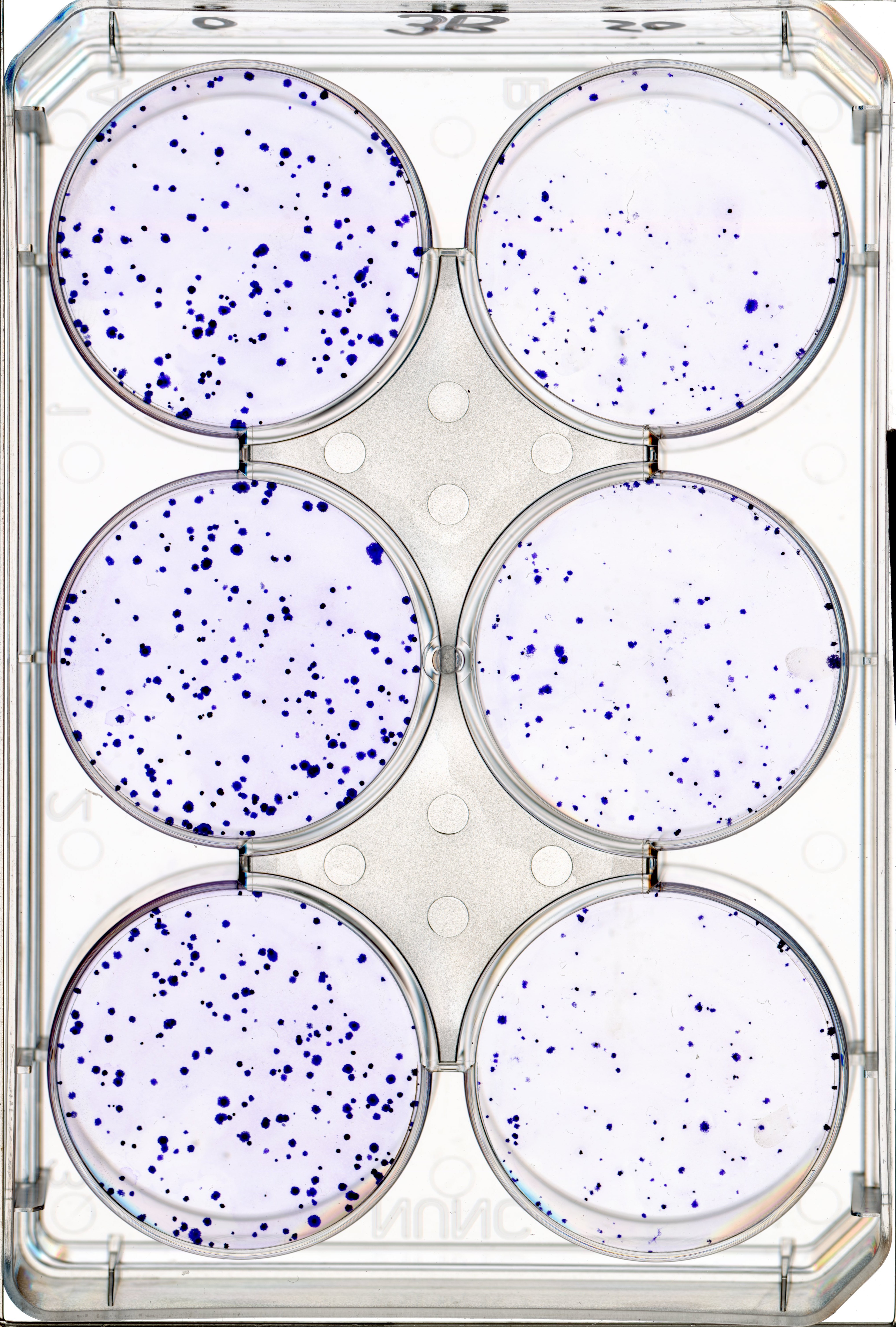

Supplement: Supplementary file 12 — Figure EV4 Source Data [file 44318_2024_108_MOESM12_ESM.zip › EMBOJ-2023-115654_FigEV4_sourcedata/EV4D/nodox_KO-WT_0_50.jpg]

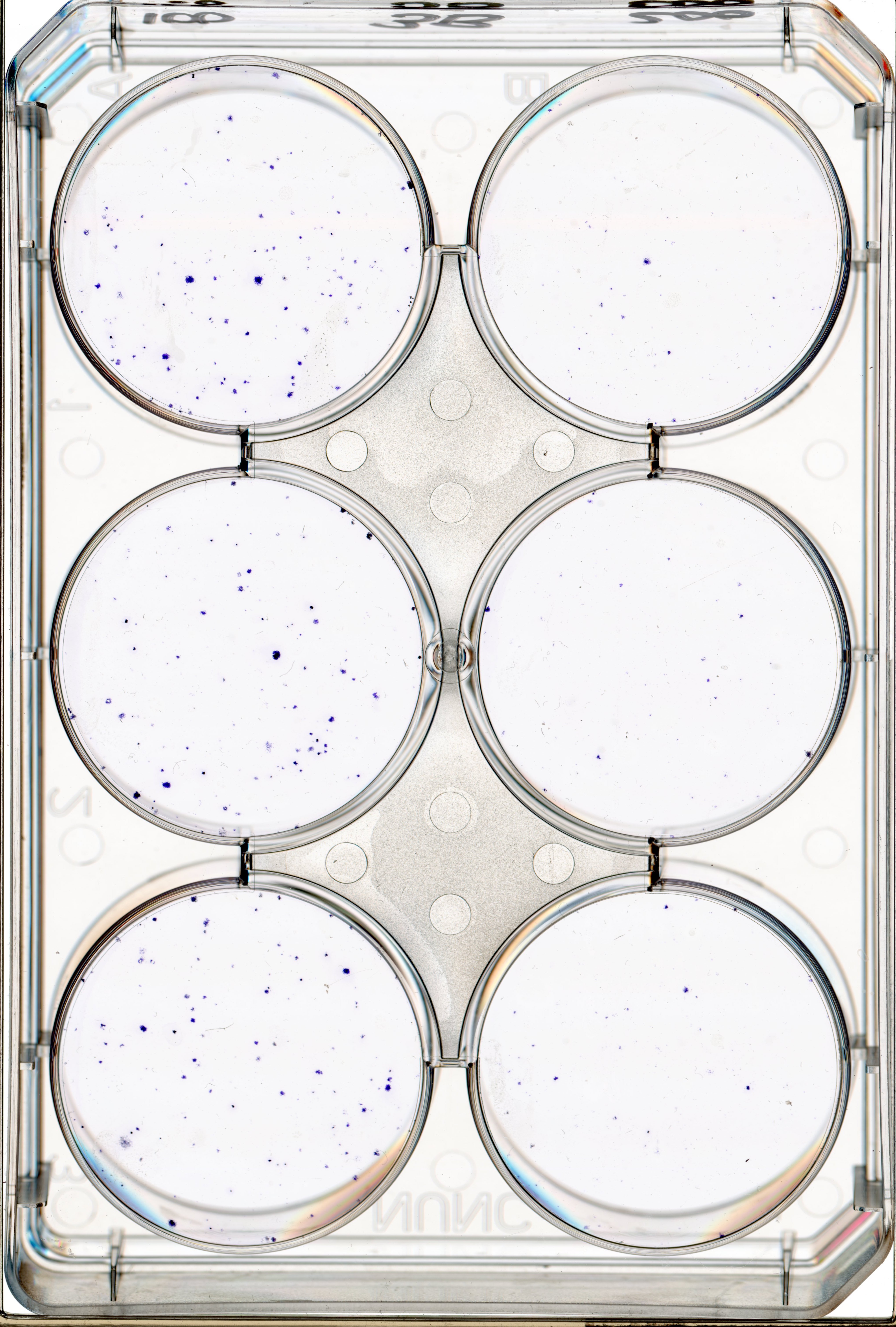

Supplement: Supplementary file 12 — Figure EV4 Source Data [file 44318_2024_108_MOESM12_ESM.zip › EMBOJ-2023-115654_FigEV4_sourcedata/EV4D/nodox_KO-WT_100_200.jpg]

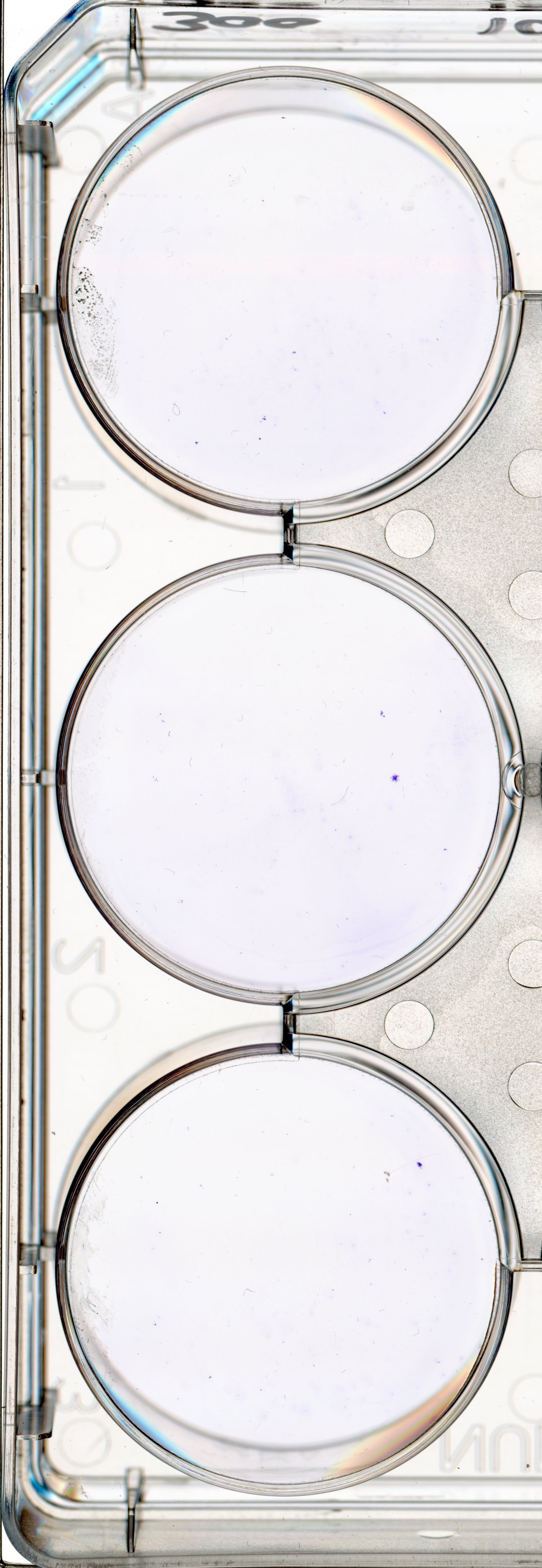

Supplement: Supplementary file 12 — Figure EV4 Source Data [file 44318_2024_108_MOESM12_ESM.zip › EMBOJ-2023-115654_FigEV4_sourcedata/EV4D/DOX-KO-CCAA_300.jpg]

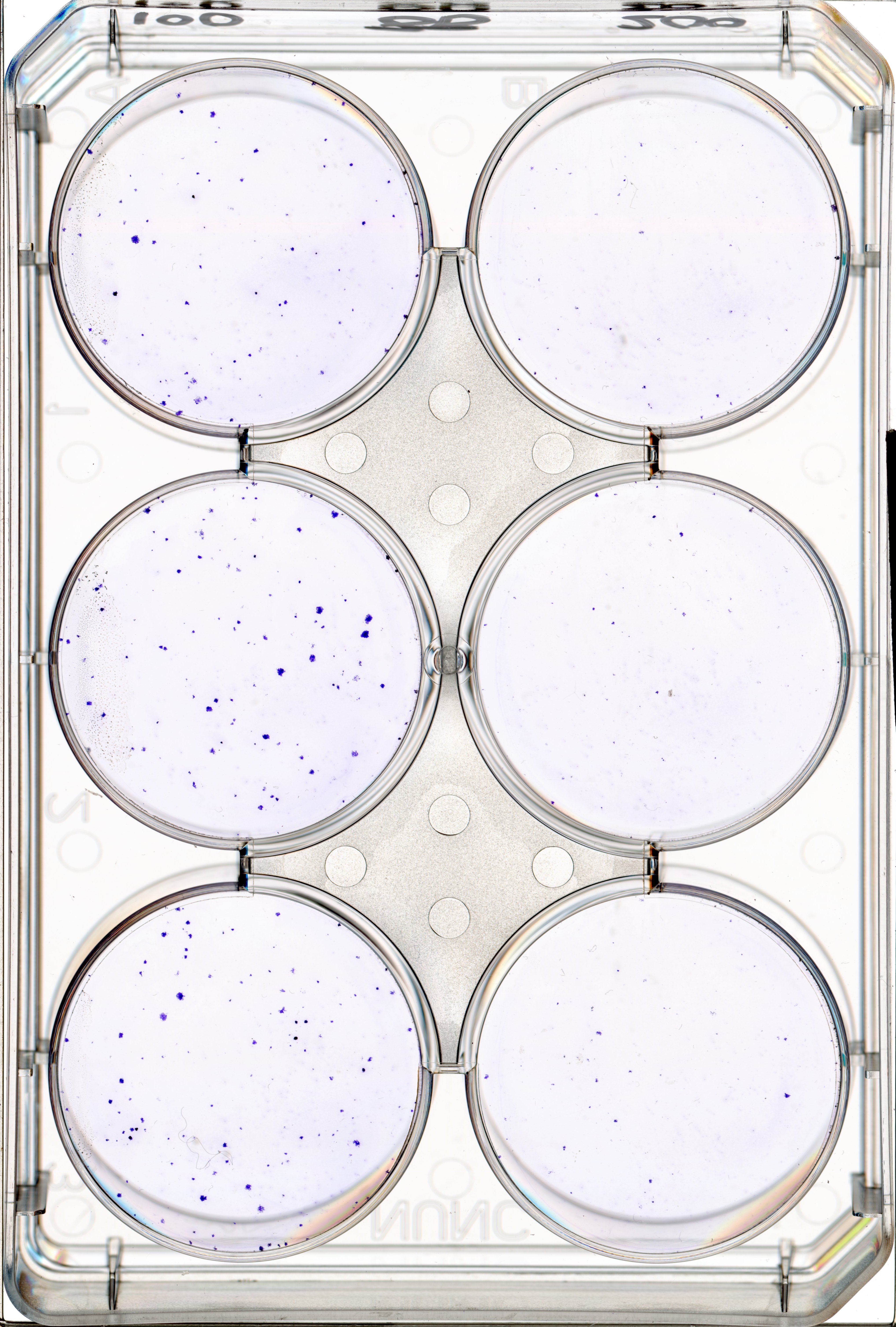

Supplement: Supplementary file 12 — Figure EV4 Source Data [file 44318_2024_108_MOESM12_ESM.zip › EMBOJ-2023-115654_FigEV4_sourcedata/EV4D/DOX-KO-ev_100_200.jpg]

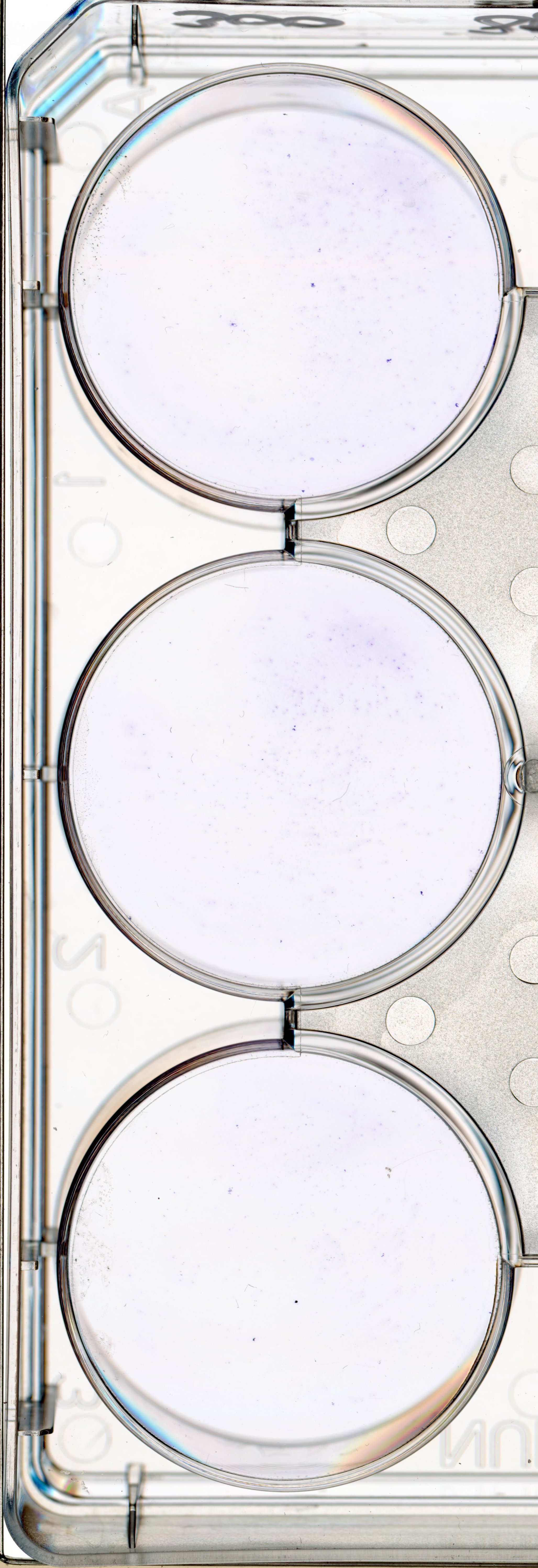

Supplement: Supplementary file 12 — Figure EV4 Source Data [file 44318_2024_108_MOESM12_ESM.zip › EMBOJ-2023-115654_FigEV4_sourcedata/EV4D/DOX-KO-ev_300.jpg]

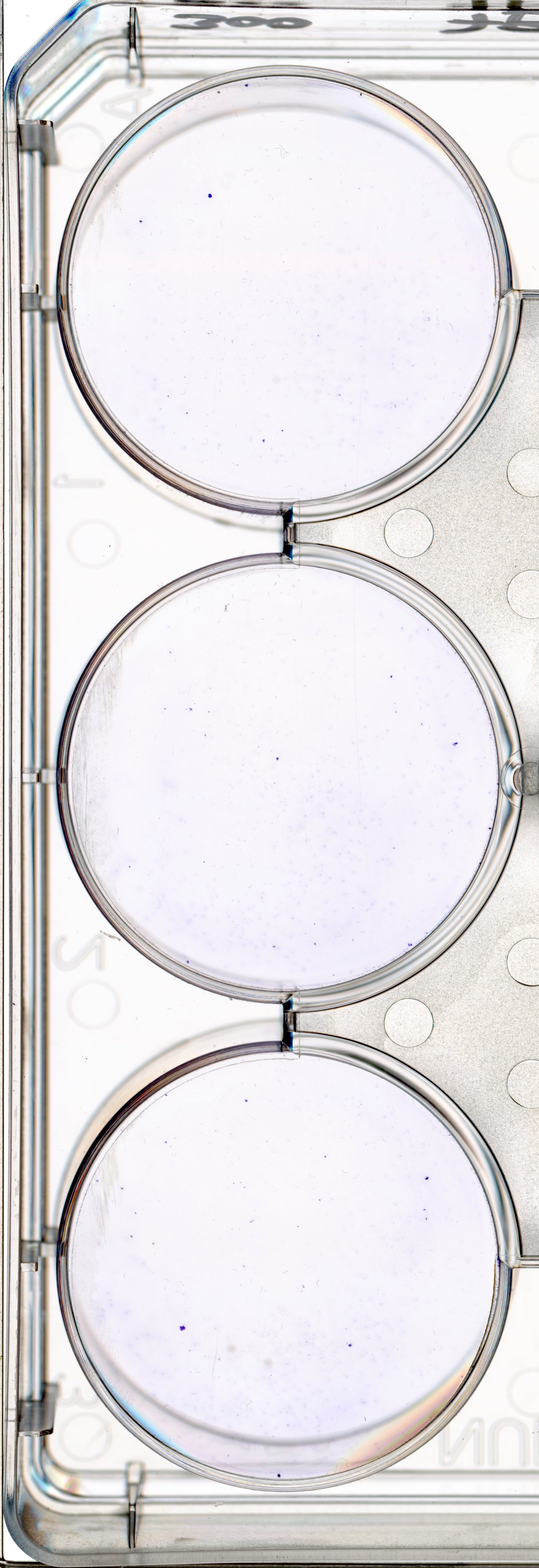

Supplement: Supplementary file 12 — Figure EV4 Source Data [file 44318_2024_108_MOESM12_ESM.zip › EMBOJ-2023-115654_FigEV4_sourcedata/EV4D/DOX-WT_300.jpg]

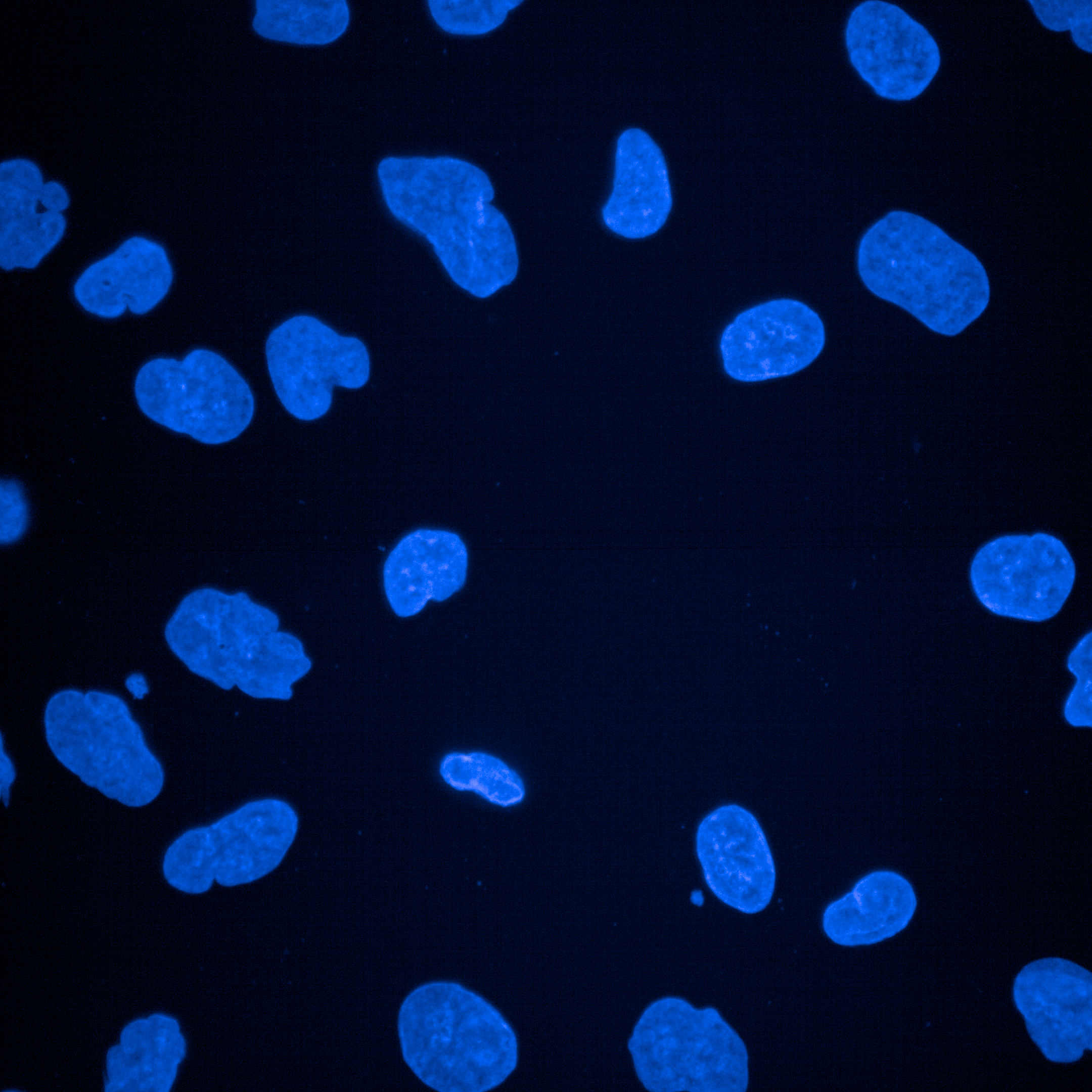

Supplement: Supplementary file 12 — Figure EV4 Source Data [file 44318_2024_108_MOESM12_ESM.zip › EMBOJ-2023-115654_FigEV4_sourcedata/EV4B/E231109 HA-EV PLA 5dC - DAPI.png]

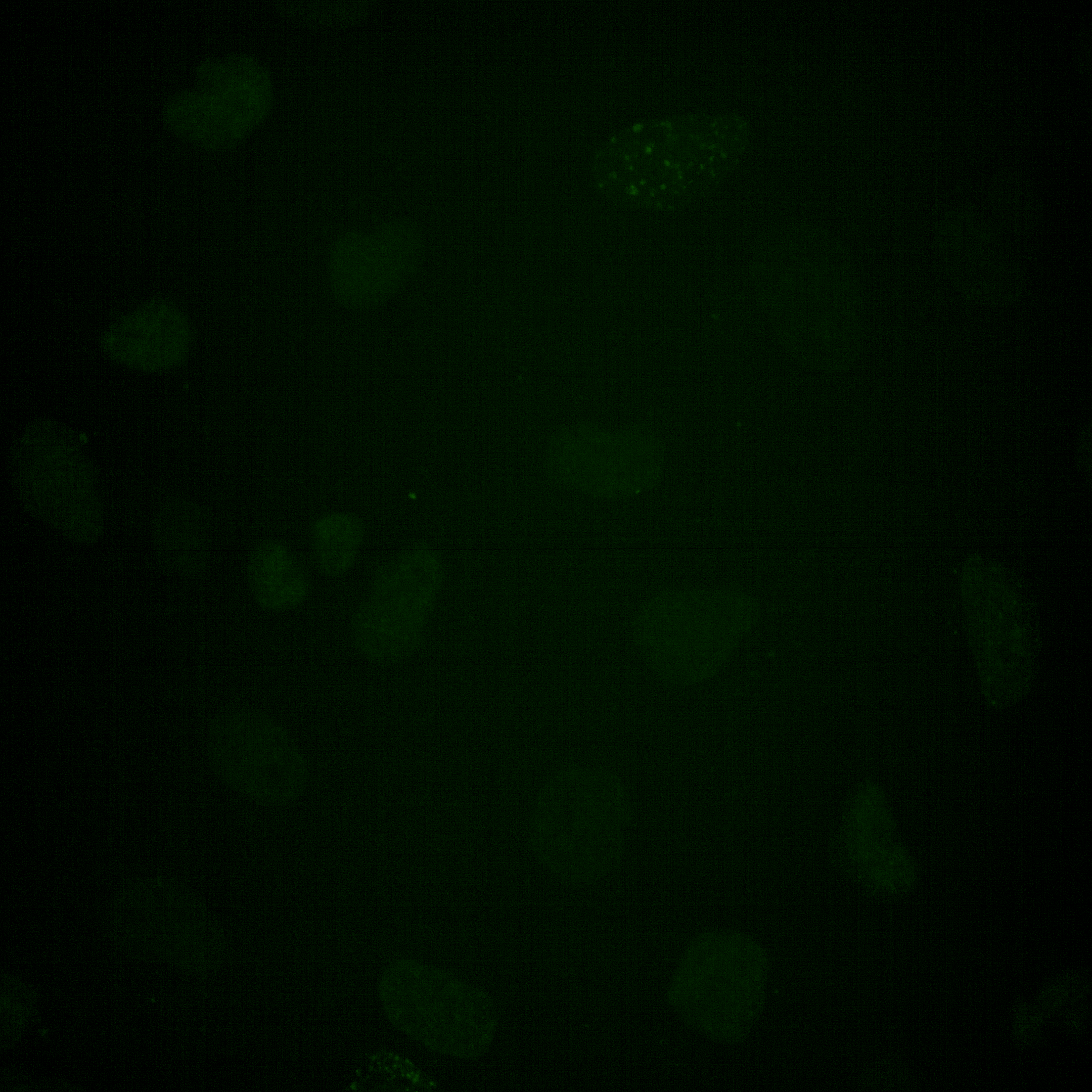

Supplement: Supplementary file 12 — Figure EV4 Source Data [file 44318_2024_108_MOESM12_ESM.zip › EMBOJ-2023-115654_FigEV4_sourcedata/EV4B/E231109 HA-EV PLA dC - GFP.png]
