# Supplementary figures and images for: Decitabine cytotoxicity is promoted by dCMP deaminase DCTD and mitigated by SUMO-dependent E3 ligase TOPORS (part 3 of 3)
Source: EMBO J. 2024 May 17;43(12):6. doi: 10.1038/s44318-024-00108-2 (PMC11183266; doi:10.1038/s44318-024-00108-2)

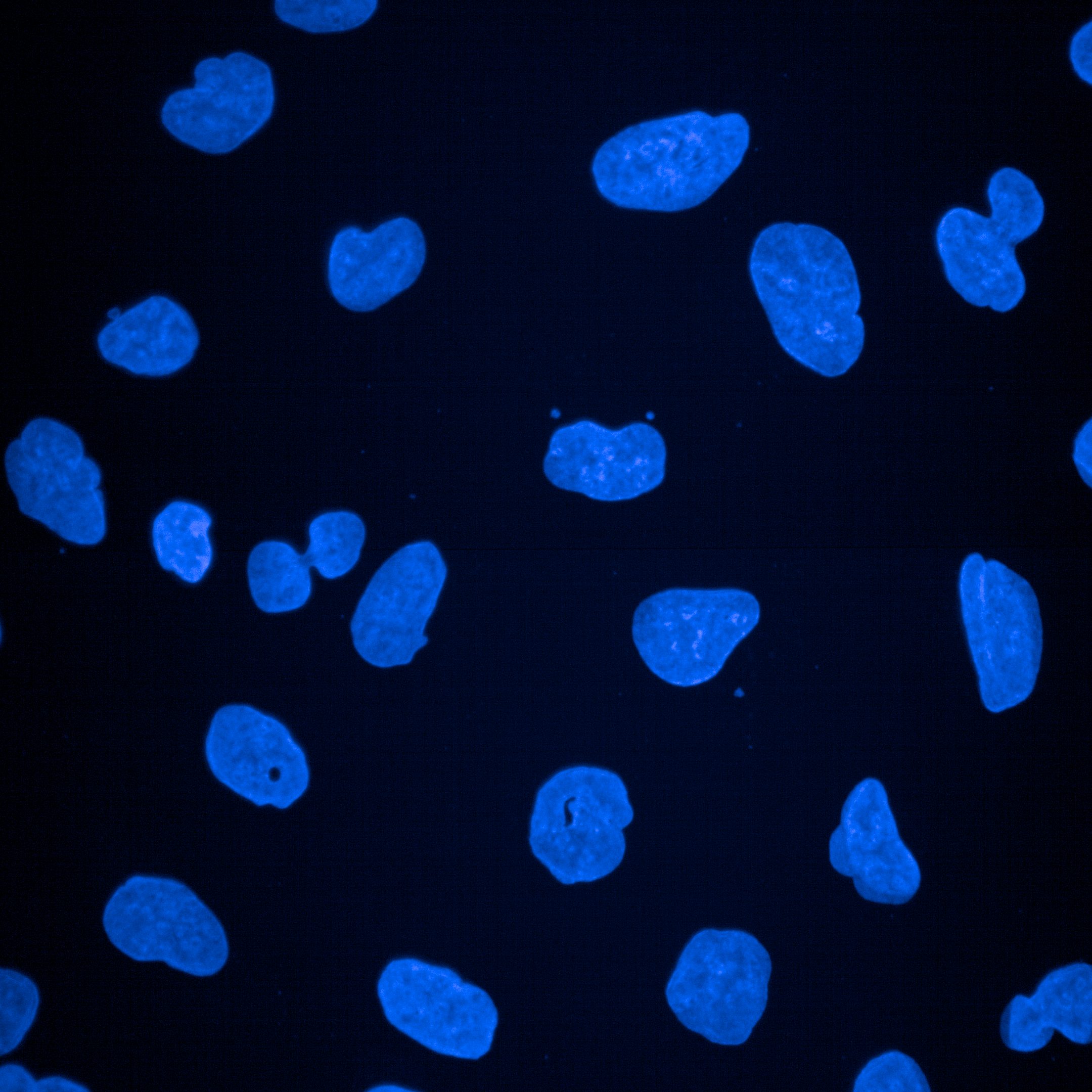

Supplement: Supplementary file 12 — Figure EV4 Source Data [file 44318_2024_108_MOESM12_ESM.zip › EMBOJ-2023-115654_FigEV4_sourcedata/EV4B/E231109 HA-EV PLA dC - DAPI.png]

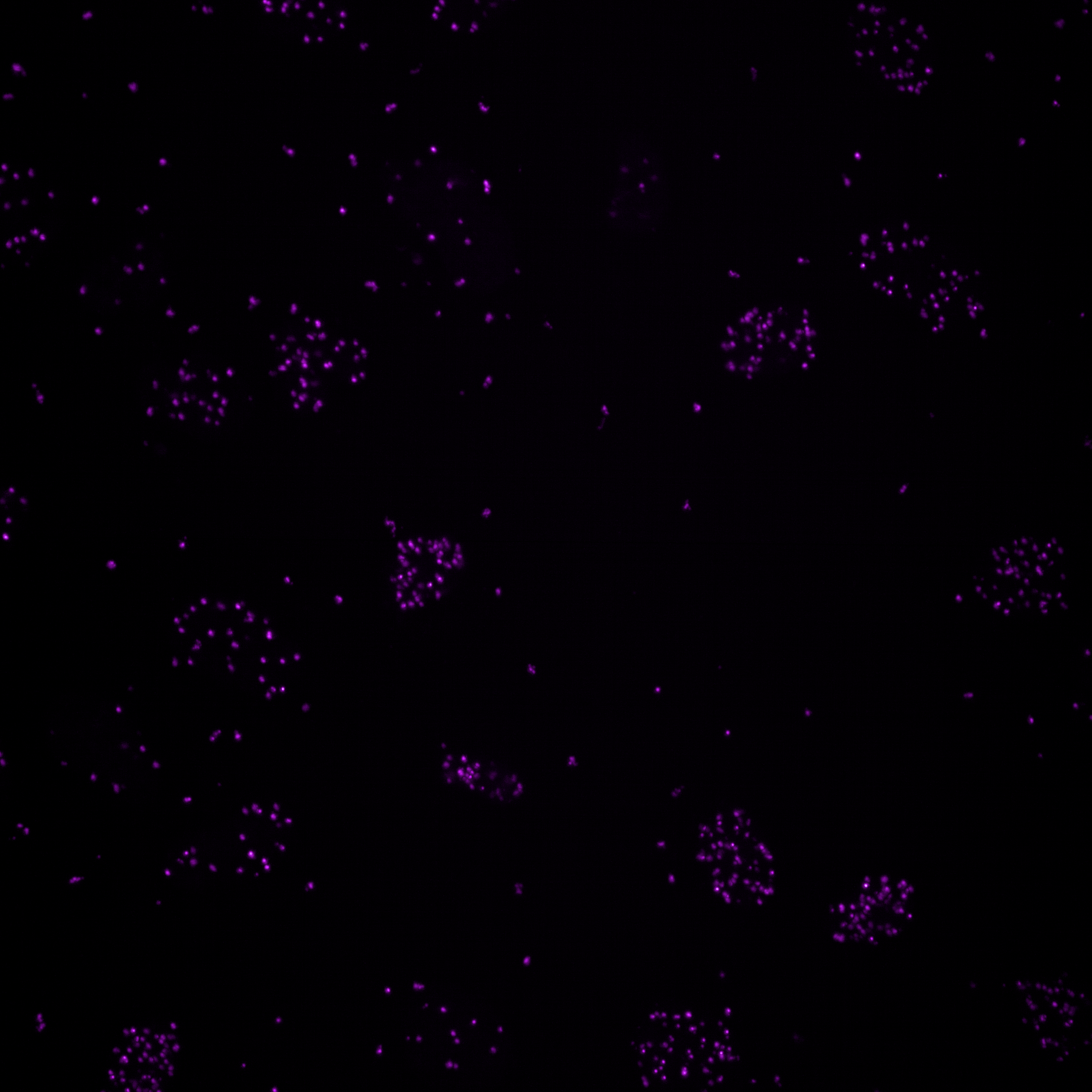

Supplement: Supplementary file 12 — Figure EV4 Source Data [file 44318_2024_108_MOESM12_ESM.zip › EMBOJ-2023-115654_FigEV4_sourcedata/EV4B/E231109 HA-EV PLA 5dC - PLA.png]

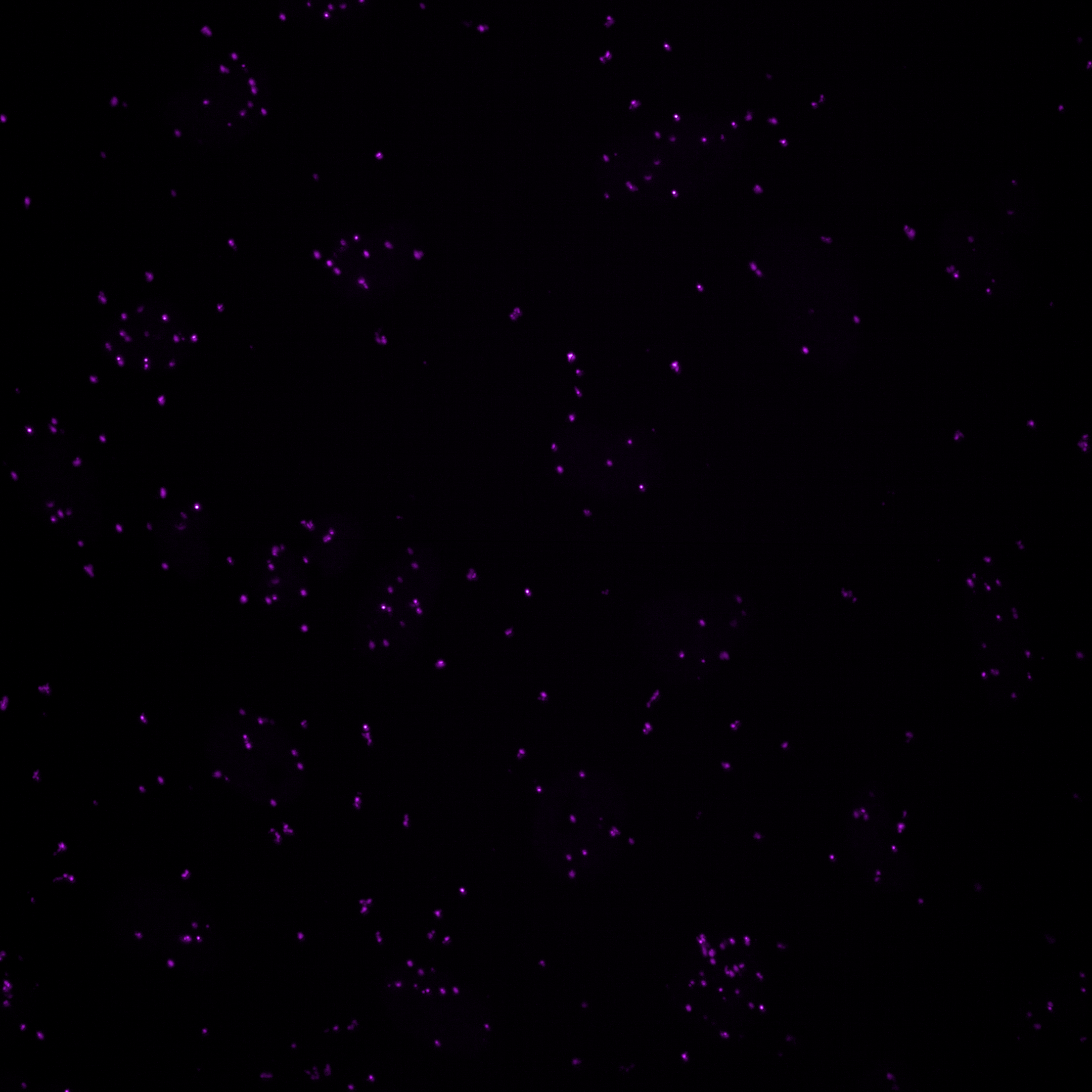

Supplement: Supplementary file 12 — Figure EV4 Source Data [file 44318_2024_108_MOESM12_ESM.zip › EMBOJ-2023-115654_FigEV4_sourcedata/EV4B/E231109 HA-EV PLA dC - PLA.png]

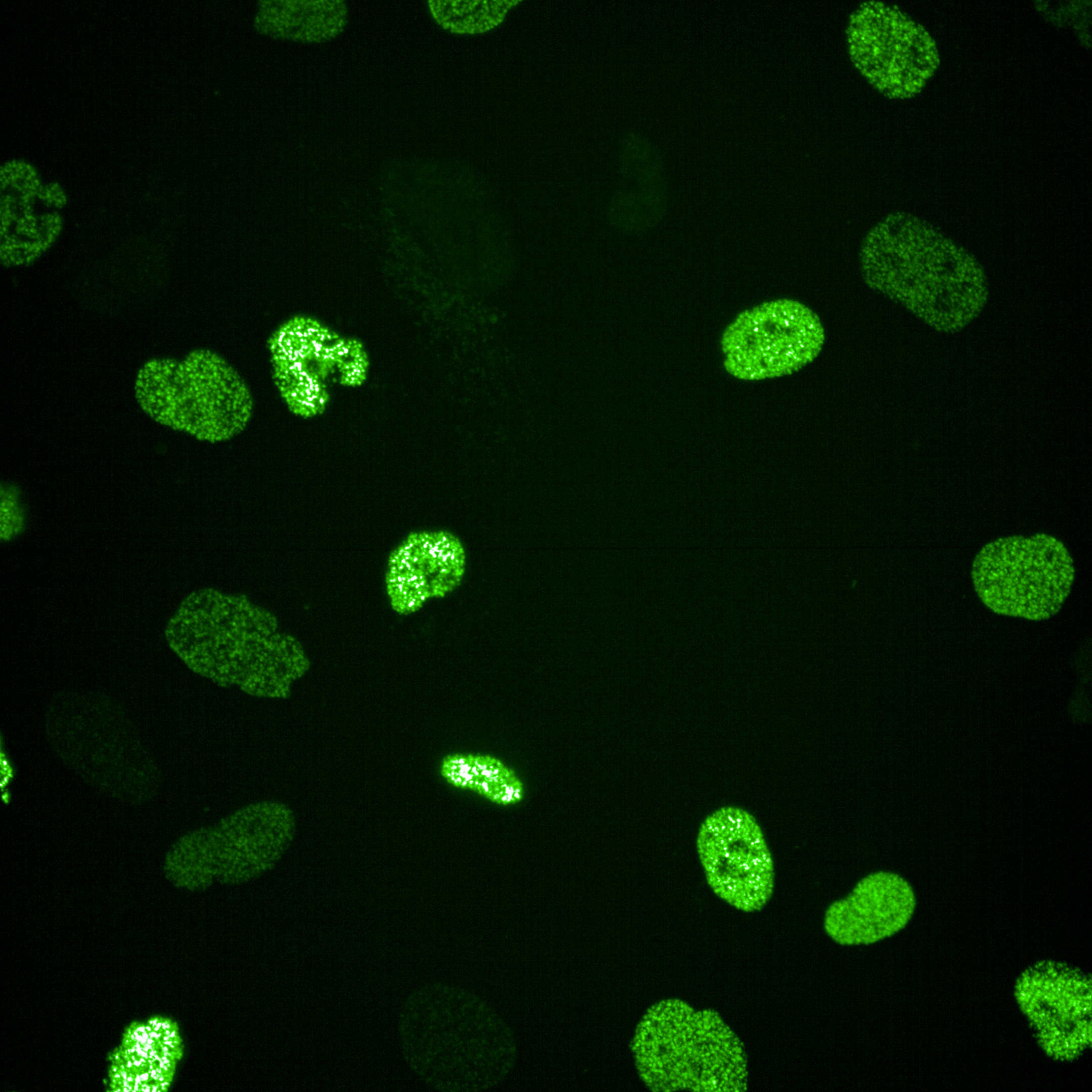

Supplement: Supplementary file 12 — Figure EV4 Source Data [file 44318_2024_108_MOESM12_ESM.zip › EMBOJ-2023-115654_FigEV4_sourcedata/EV4B/E231109 HA-EV PLA 5dC - GFP.png]

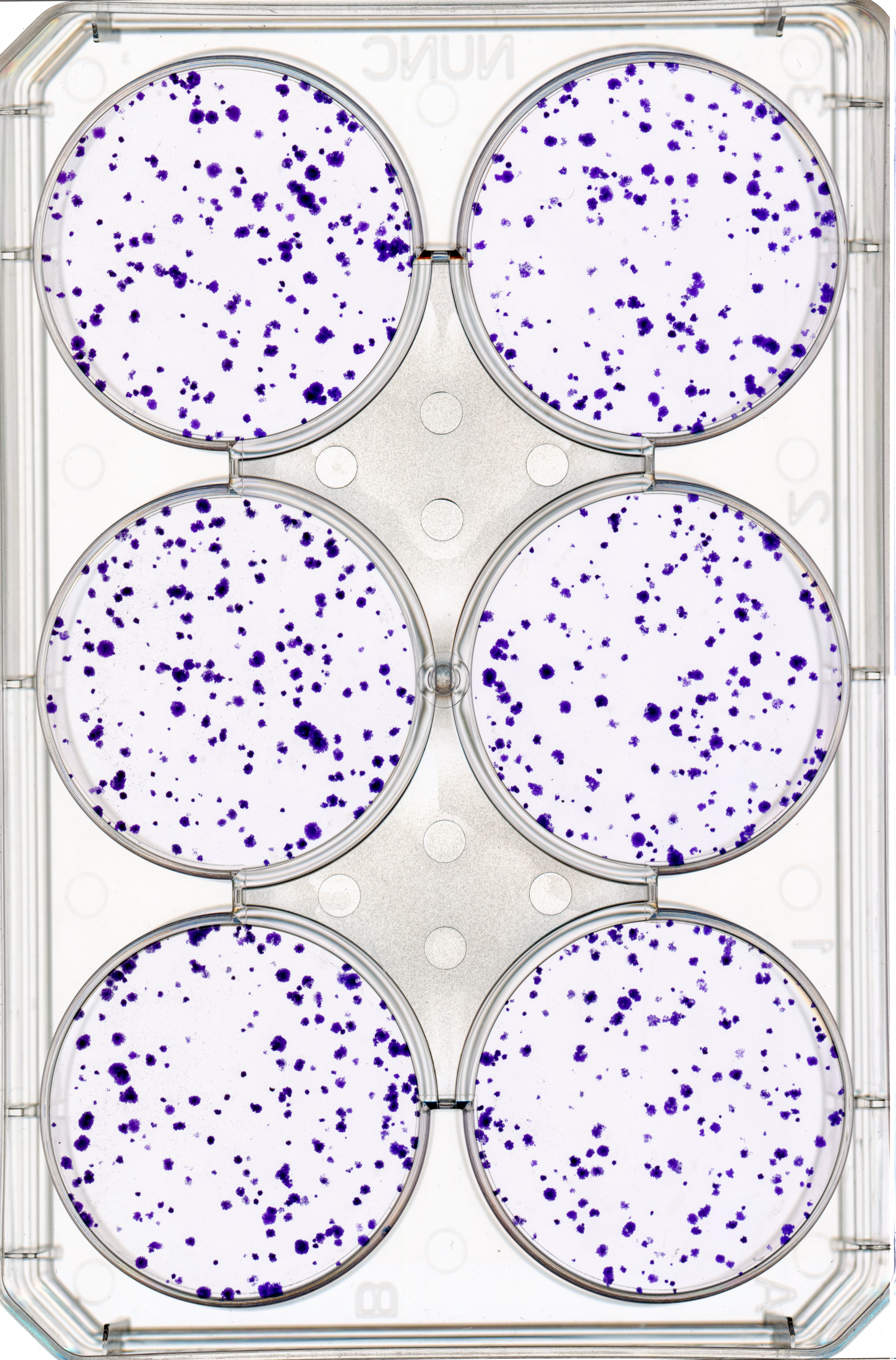

Supplement: Supplementary file 13 — Figure EV5 Source Data [file 44318_2024_108_MOESM13_ESM.zip › EMBOJ-2023-115654_FigEV5_sourcedata/EV5K/E230501 U2KTRSdko1siCtrl 5dC0-2.5.jpg]

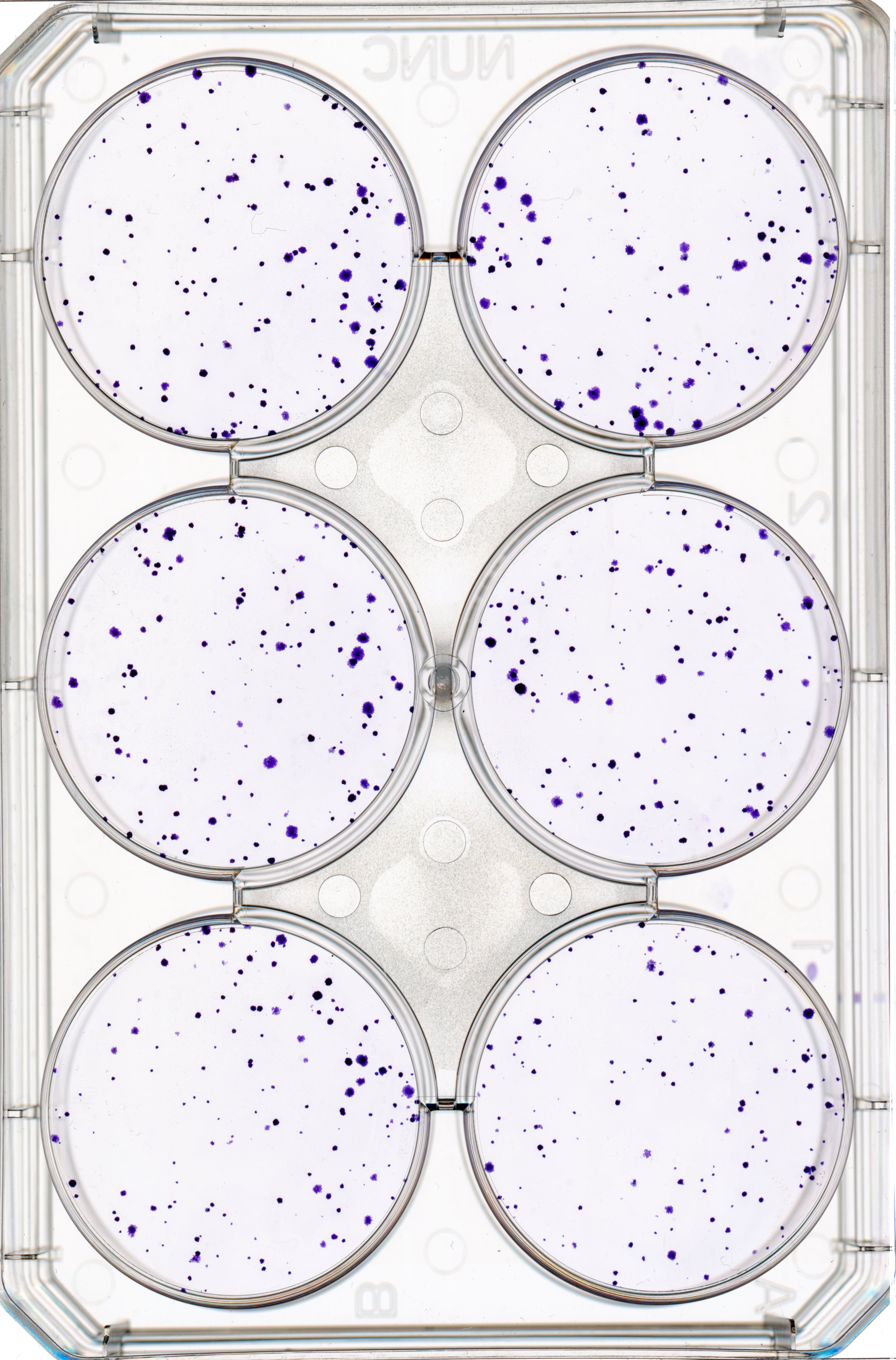

Supplement: Supplementary file 13 — Figure EV5 Source Data [file 44318_2024_108_MOESM13_ESM.zip › EMBOJ-2023-115654_FigEV5_sourcedata/EV5K/E230501 WTsiRNF4 5dC5-7.5.jpg]

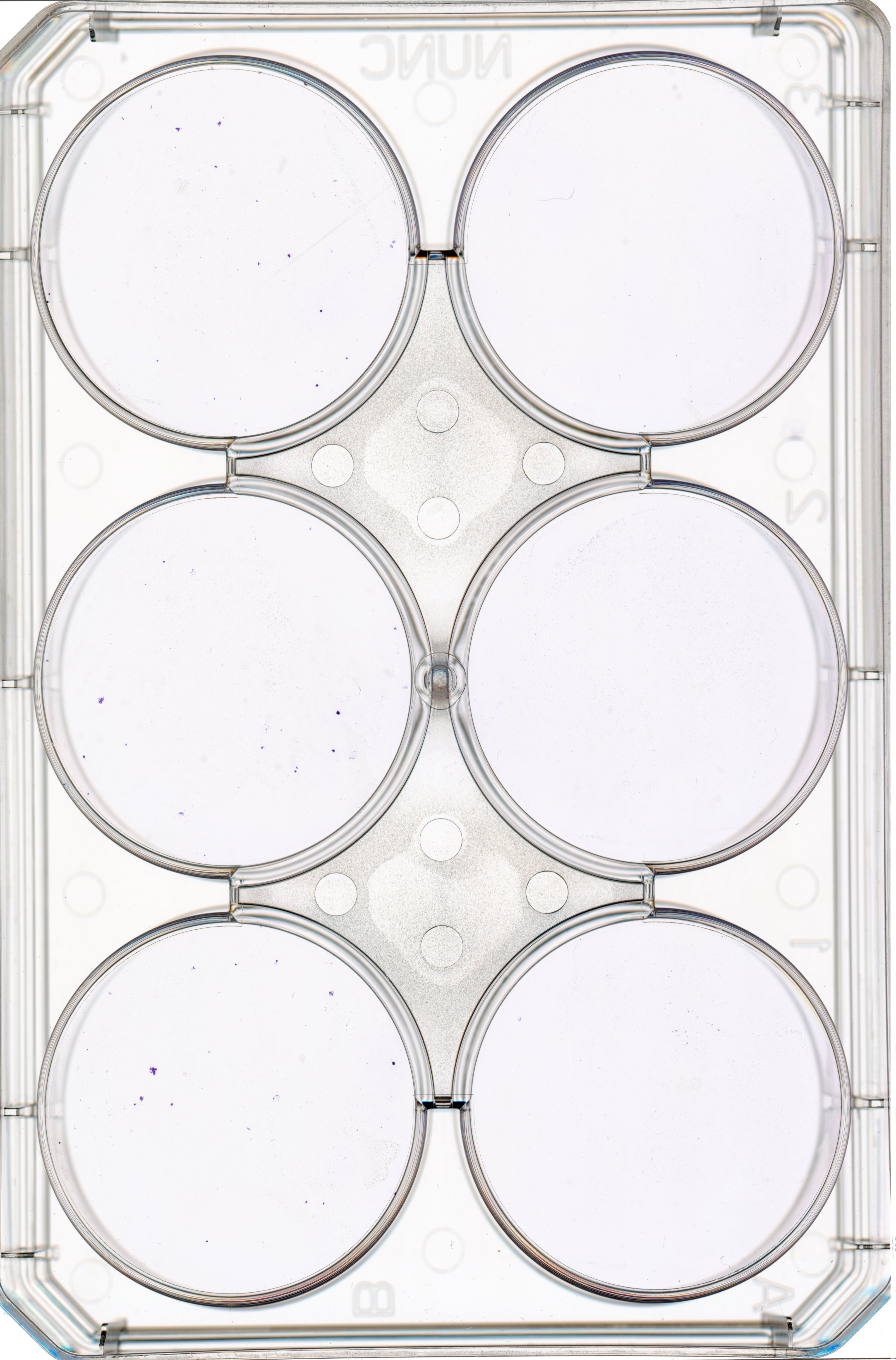

Supplement: Supplementary file 13 — Figure EV5 Source Data [file 44318_2024_108_MOESM13_ESM.zip › EMBOJ-2023-115654_FigEV5_sourcedata/EV5K/E230501 U2KTRSdko1siRNF4 5dC10-20.jpg]

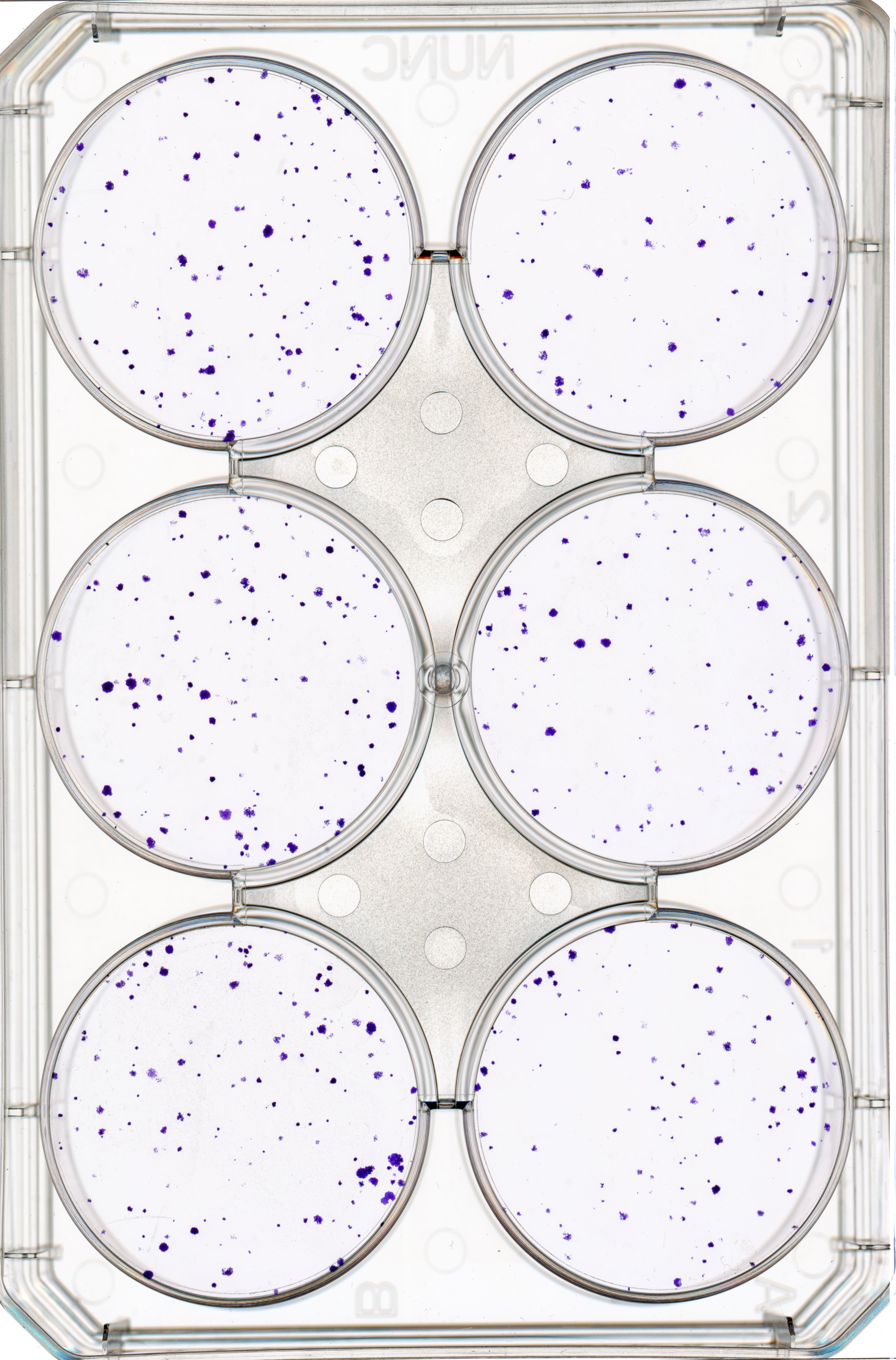

Supplement: Supplementary file 13 — Figure EV5 Source Data [file 44318_2024_108_MOESM13_ESM.zip › EMBOJ-2023-115654_FigEV5_sourcedata/EV5K/E230501 U2KTRSdko1siRNF4 5dC0-2.5.jpg]

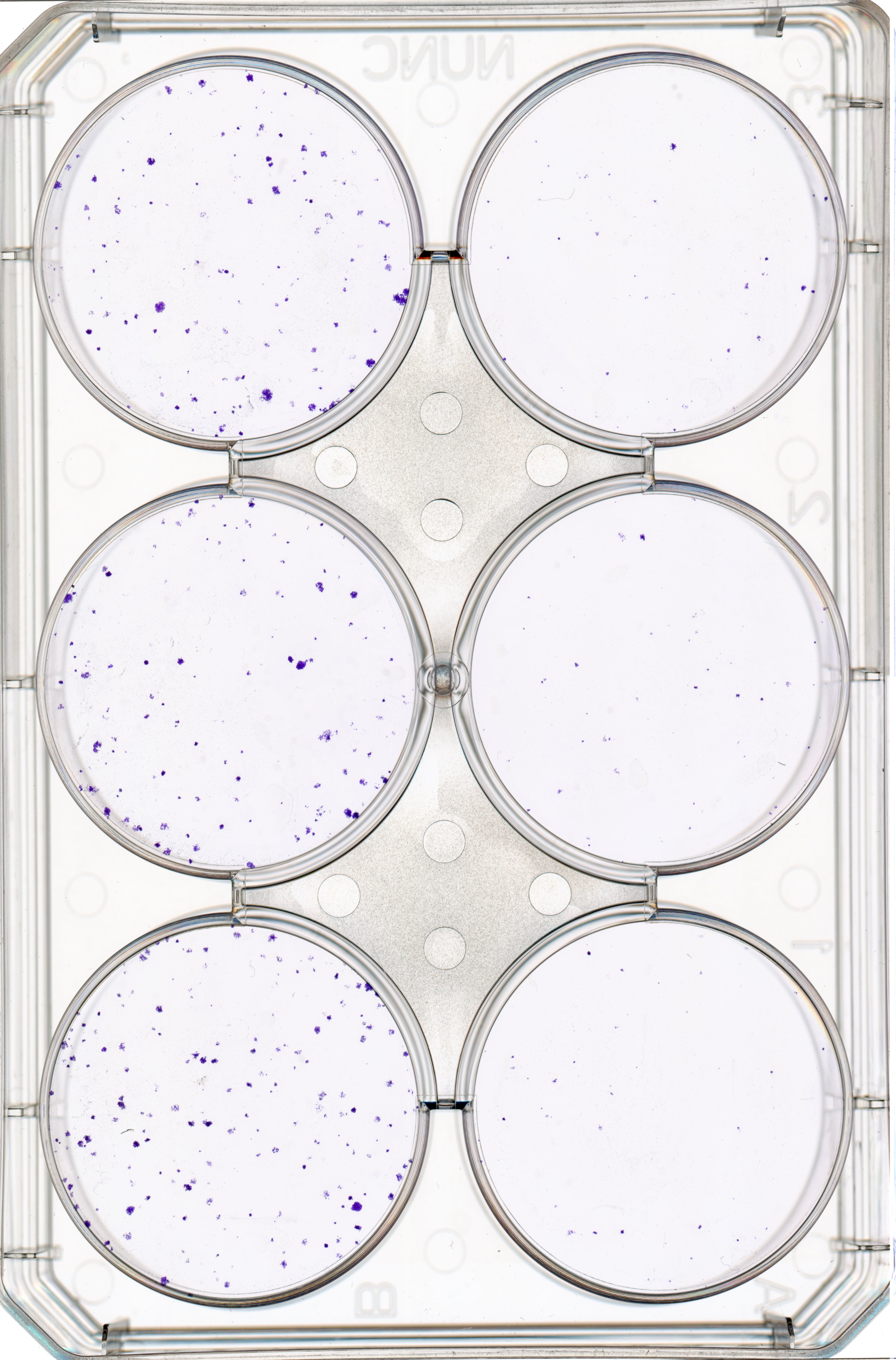

Supplement: Supplementary file 13 — Figure EV5 Source Data [file 44318_2024_108_MOESM13_ESM.zip › EMBOJ-2023-115654_FigEV5_sourcedata/EV5K/E230501 U2KTRSdko1siCtrl 5dC10-20.jpg]

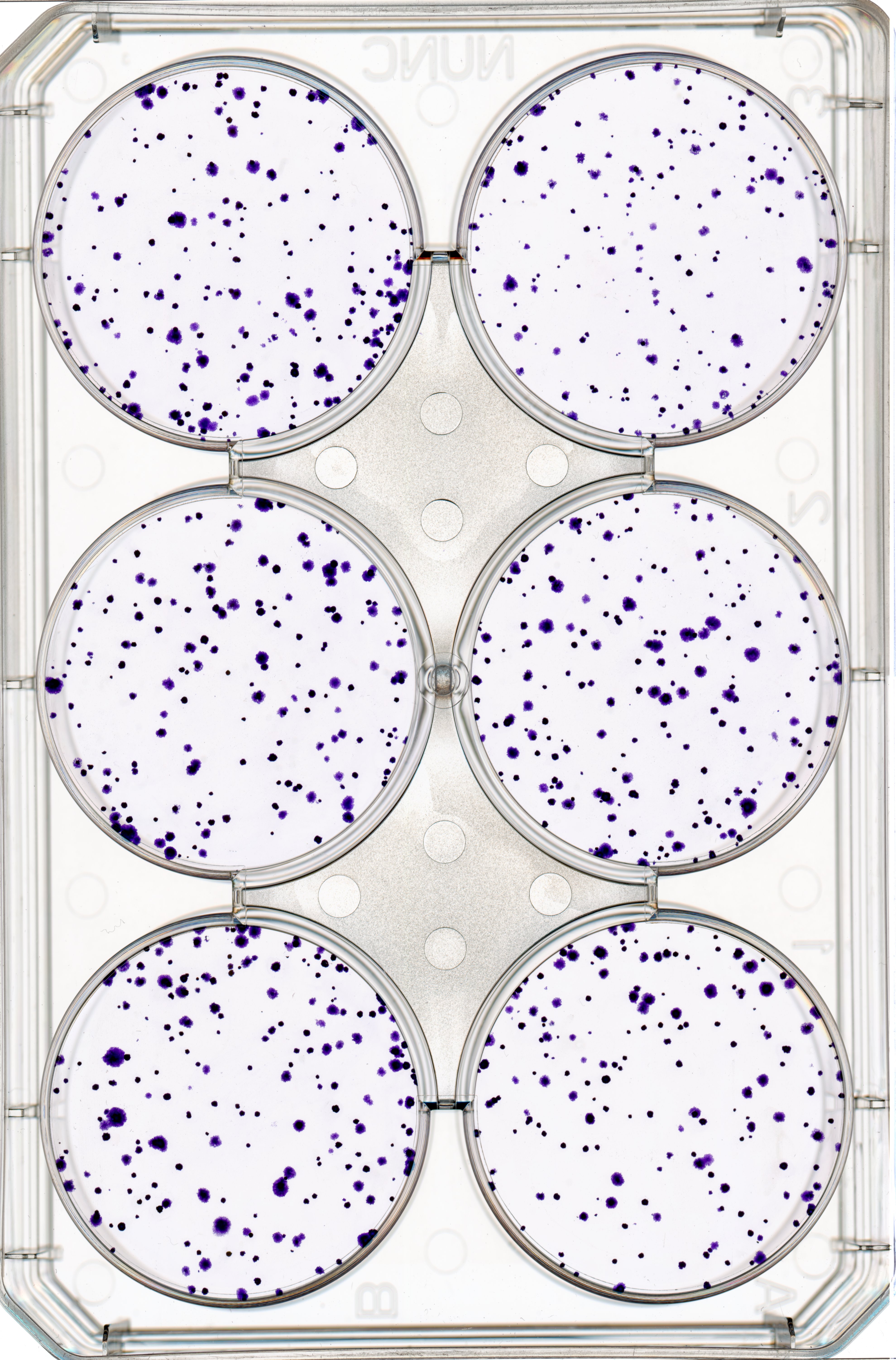

Supplement: Supplementary file 13 — Figure EV5 Source Data [file 44318_2024_108_MOESM13_ESM.zip › EMBOJ-2023-115654_FigEV5_sourcedata/EV5K/E230501 WTsiCtrl 5dC5-7.5.jpg]

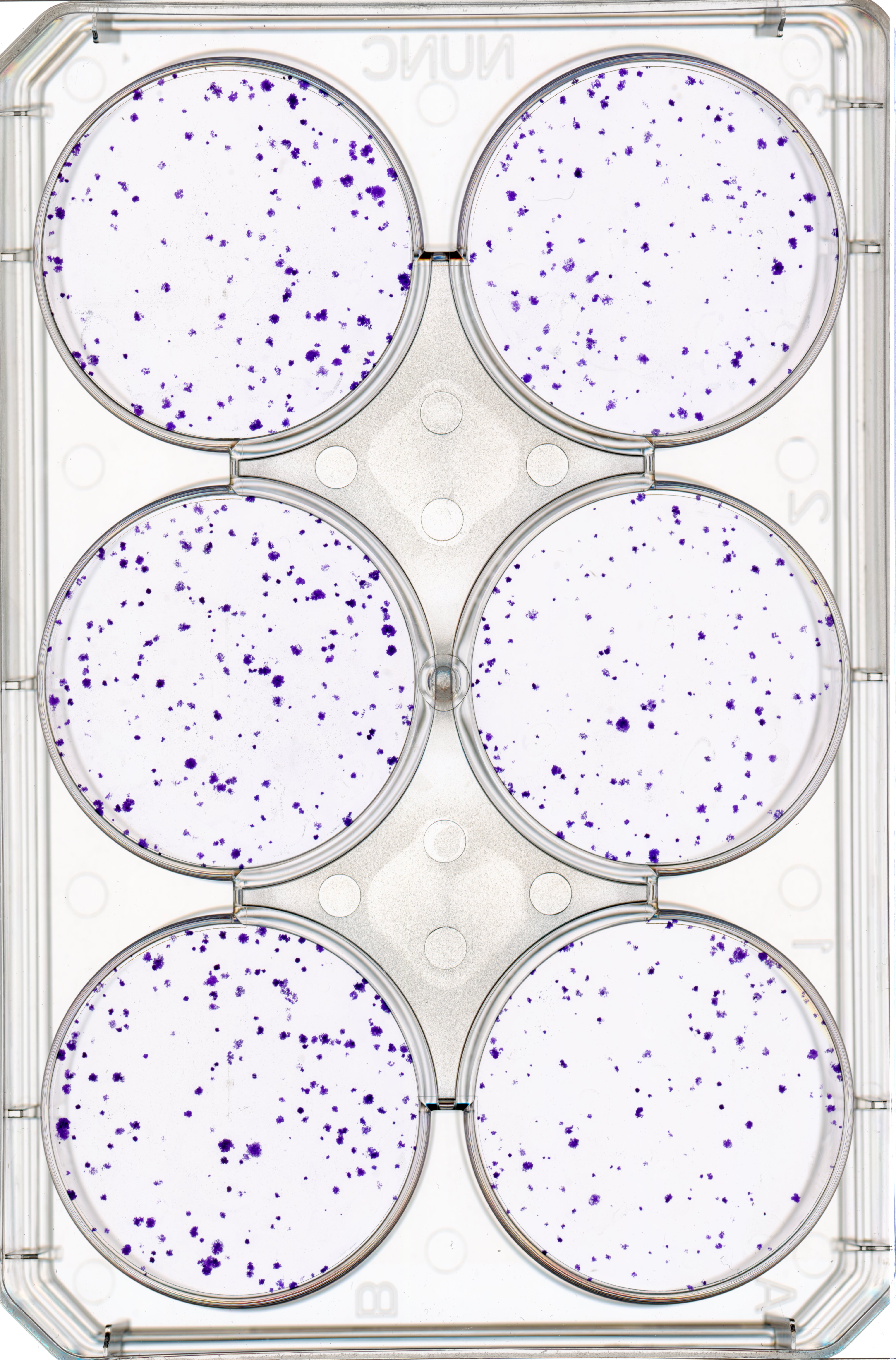

Supplement: Supplementary file 13 — Figure EV5 Source Data [file 44318_2024_108_MOESM13_ESM.zip › EMBOJ-2023-115654_FigEV5_sourcedata/EV5K/E230501 U2KTRSdko1siCtrl 5dC5-7.5.jpg]

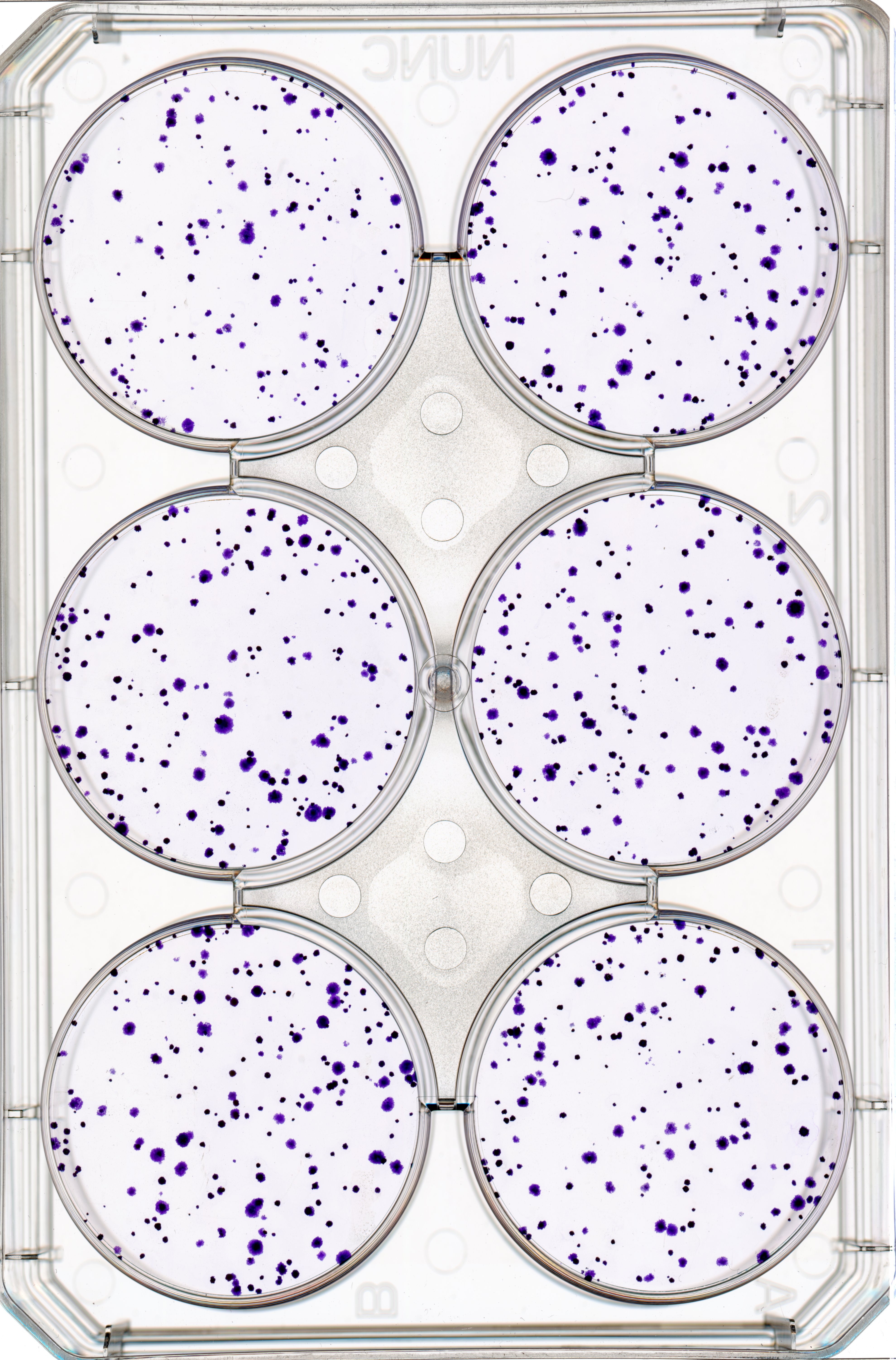

Supplement: Supplementary file 13 — Figure EV5 Source Data [file 44318_2024_108_MOESM13_ESM.zip › EMBOJ-2023-115654_FigEV5_sourcedata/EV5K/E230501 WTsiCtrl 5dC10-20.jpg]

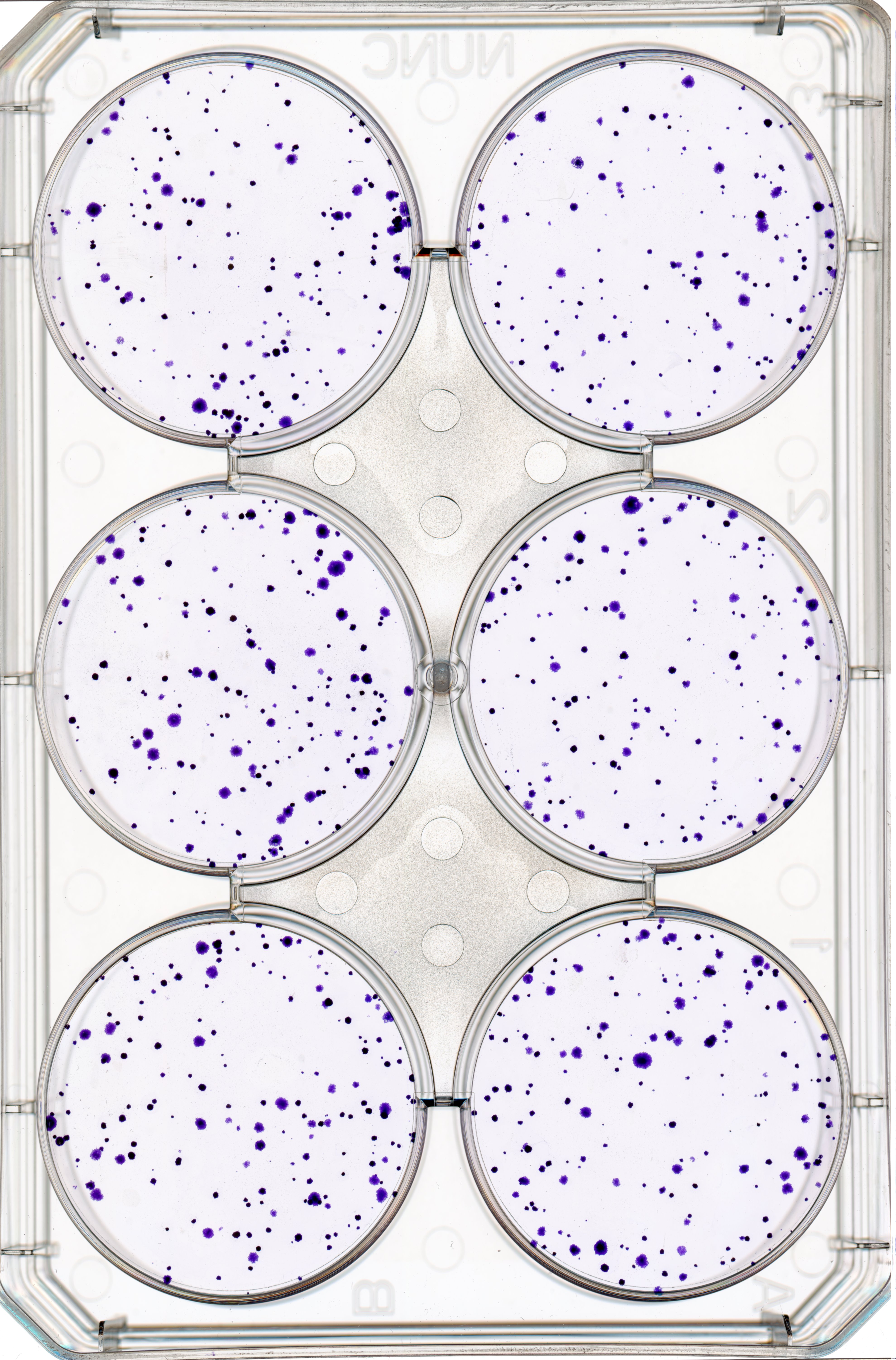

Supplement: Supplementary file 13 — Figure EV5 Source Data [file 44318_2024_108_MOESM13_ESM.zip › EMBOJ-2023-115654_FigEV5_sourcedata/EV5K/E230501 WTsiRNF4 5dC0-2.5.jpg]

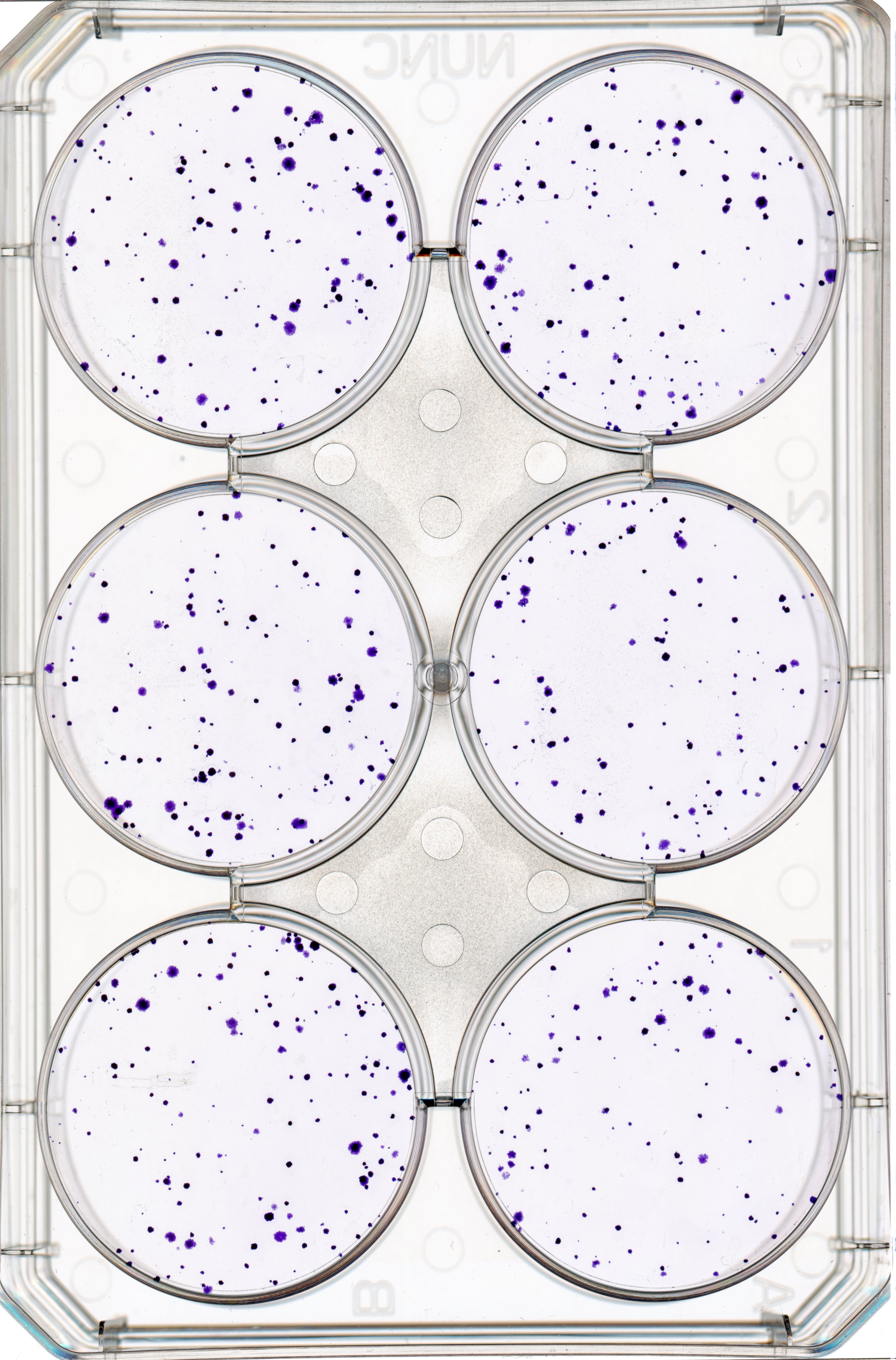

Supplement: Supplementary file 13 — Figure EV5 Source Data [file 44318_2024_108_MOESM13_ESM.zip › EMBOJ-2023-115654_FigEV5_sourcedata/EV5K/E230501 WTsiRNF4 5dC10-20.jpg]

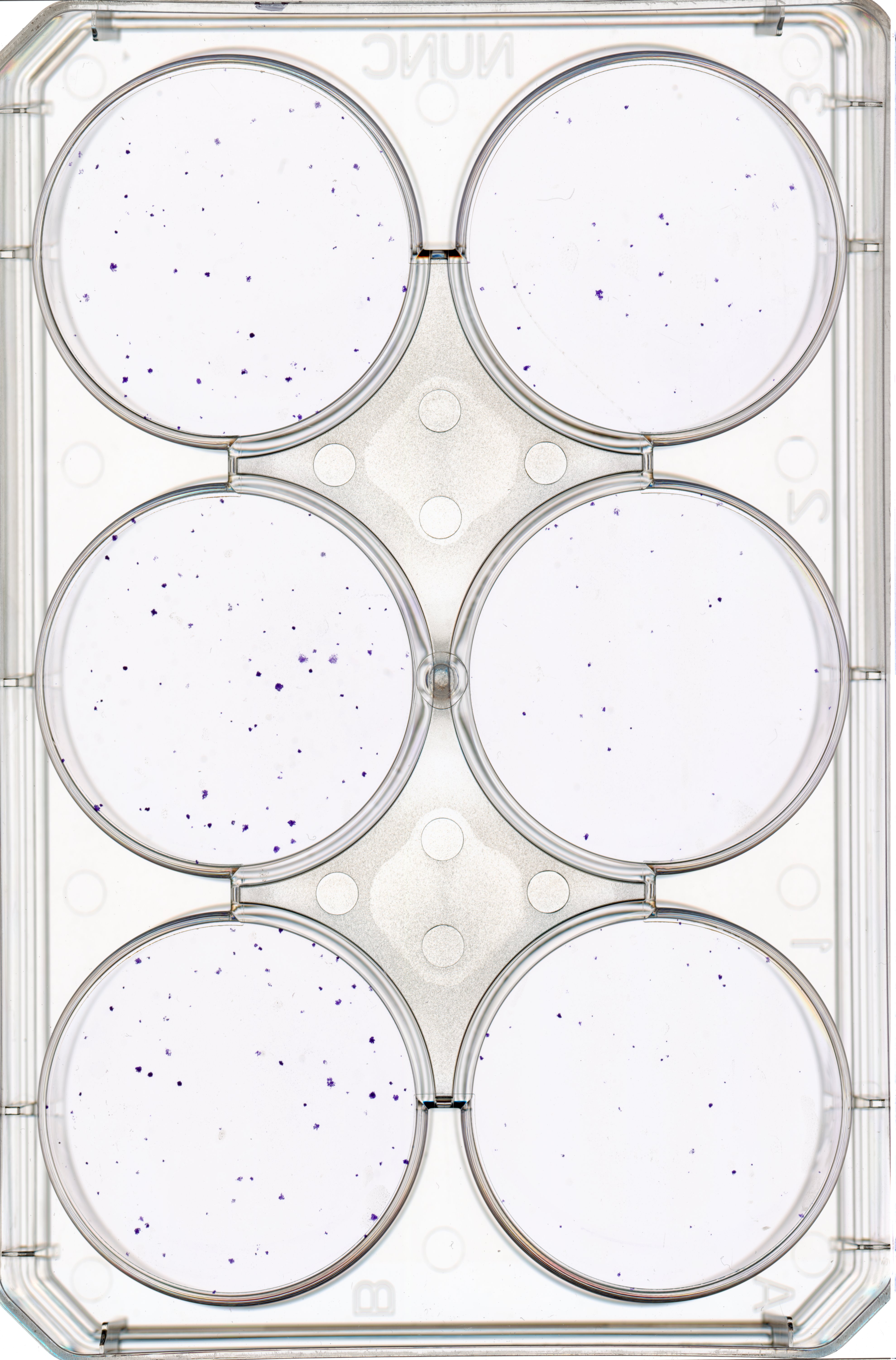

Supplement: Supplementary file 13 — Figure EV5 Source Data [file 44318_2024_108_MOESM13_ESM.zip › EMBOJ-2023-115654_FigEV5_sourcedata/EV5K/E230501 U2KTRSdko1siRNF4 5dC5-7.5.jpg]

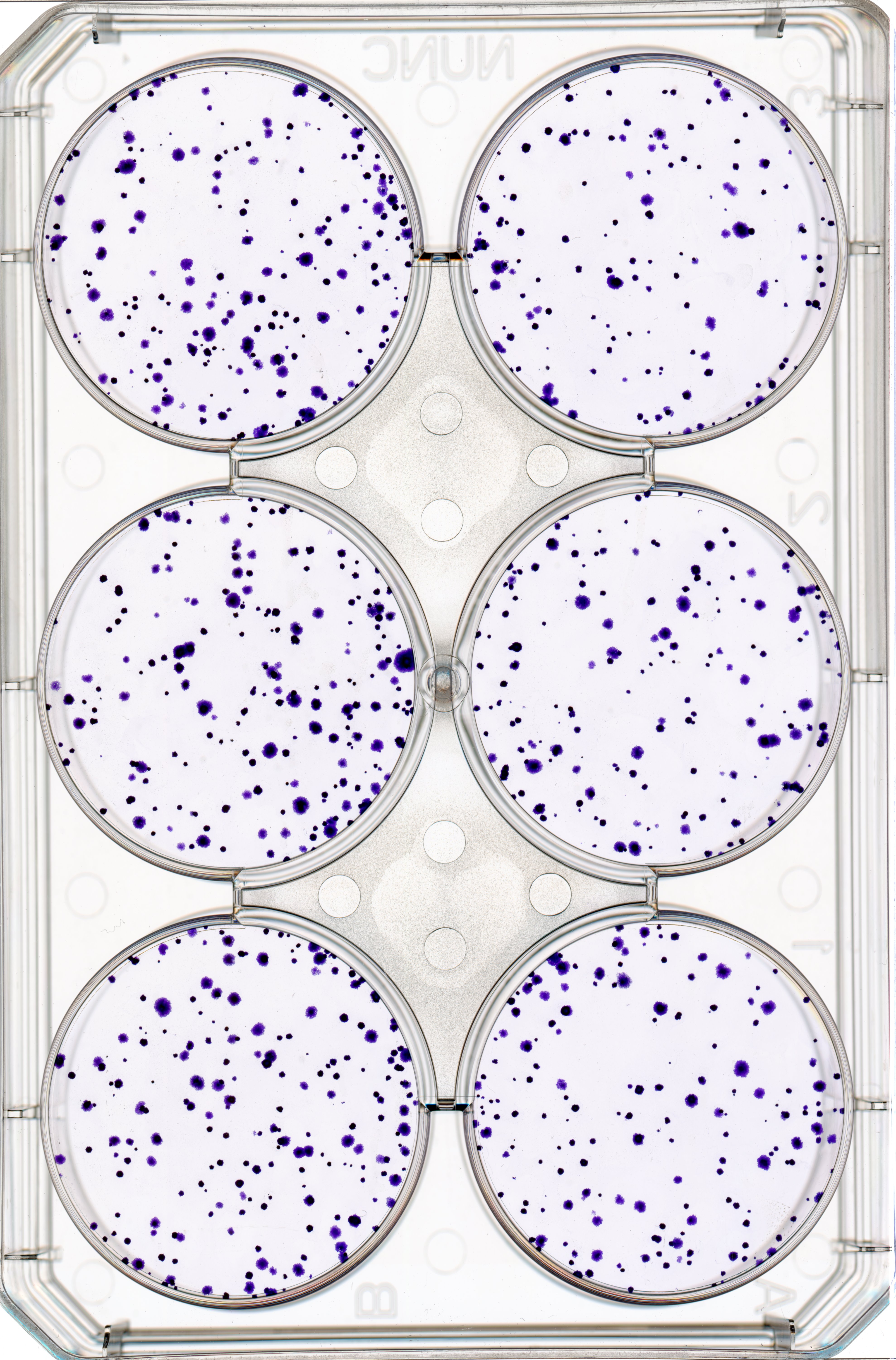

Supplement: Supplementary file 13 — Figure EV5 Source Data [file 44318_2024_108_MOESM13_ESM.zip › EMBOJ-2023-115654_FigEV5_sourcedata/EV5K/E230501 WTsiCtrl 5dC0-2.5.jpg]

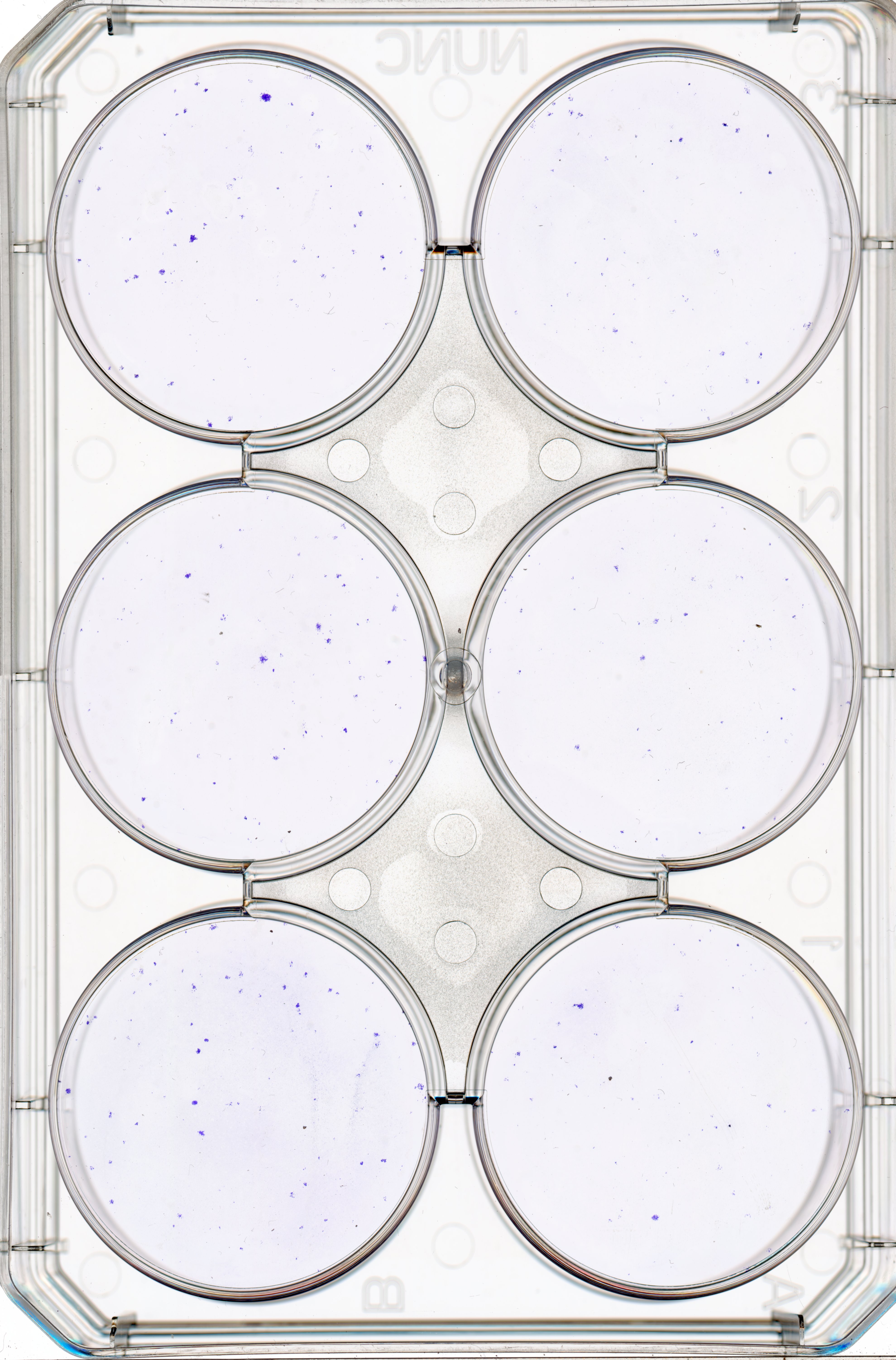

Supplement: Supplementary file 13 — Figure EV5 Source Data [file 44318_2024_108_MOESM13_ESM.zip › EMBOJ-2023-115654_FigEV5_sourcedata/EV5B/E230210 TOPORSsiRNF4 5dC200-300.jpg]

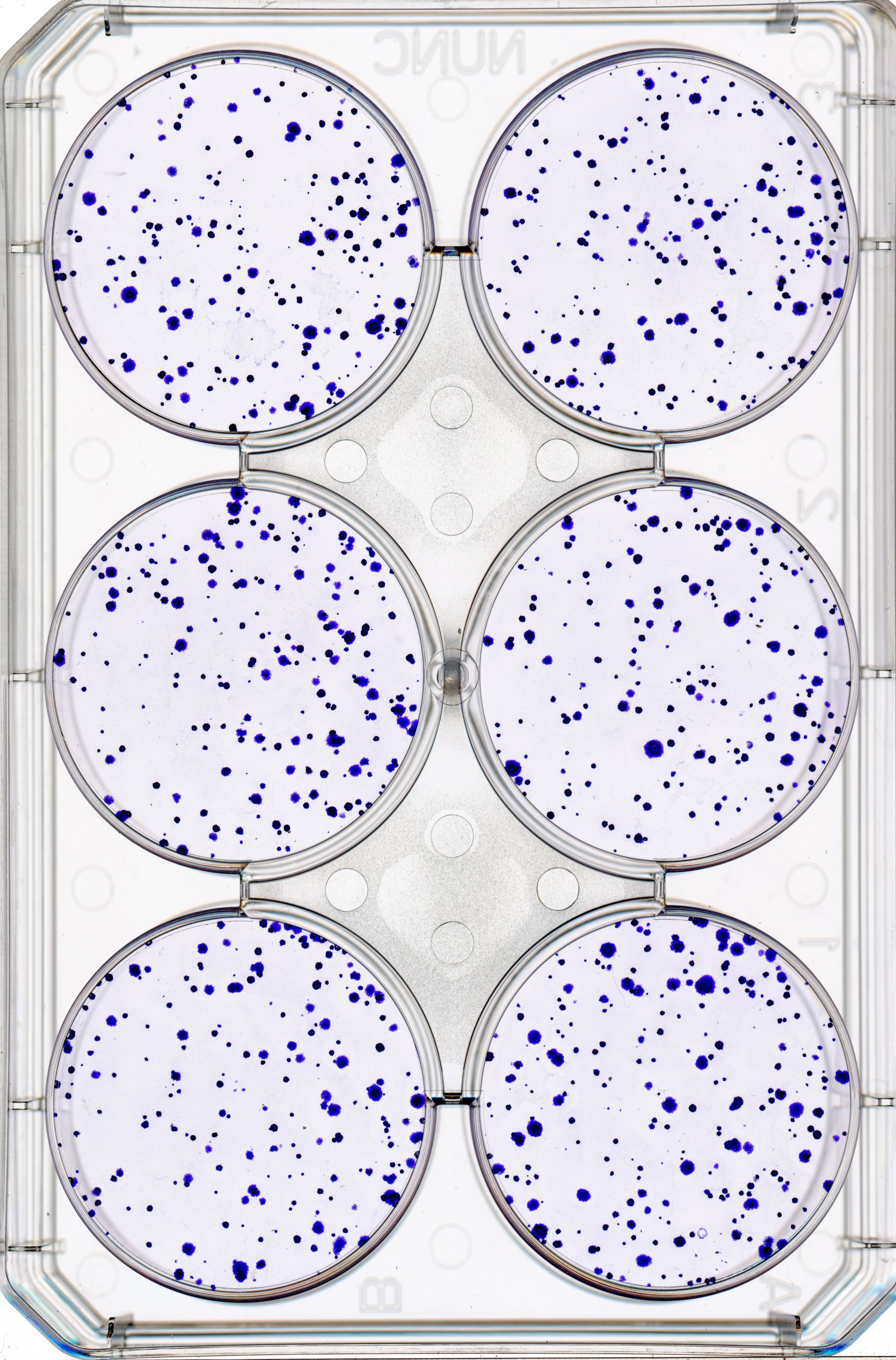

Supplement: Supplementary file 13 — Figure EV5 Source Data [file 44318_2024_108_MOESM13_ESM.zip › EMBOJ-2023-115654_FigEV5_sourcedata/EV5B/E230210 WTsiRNF4 5dC0-5.jpg]

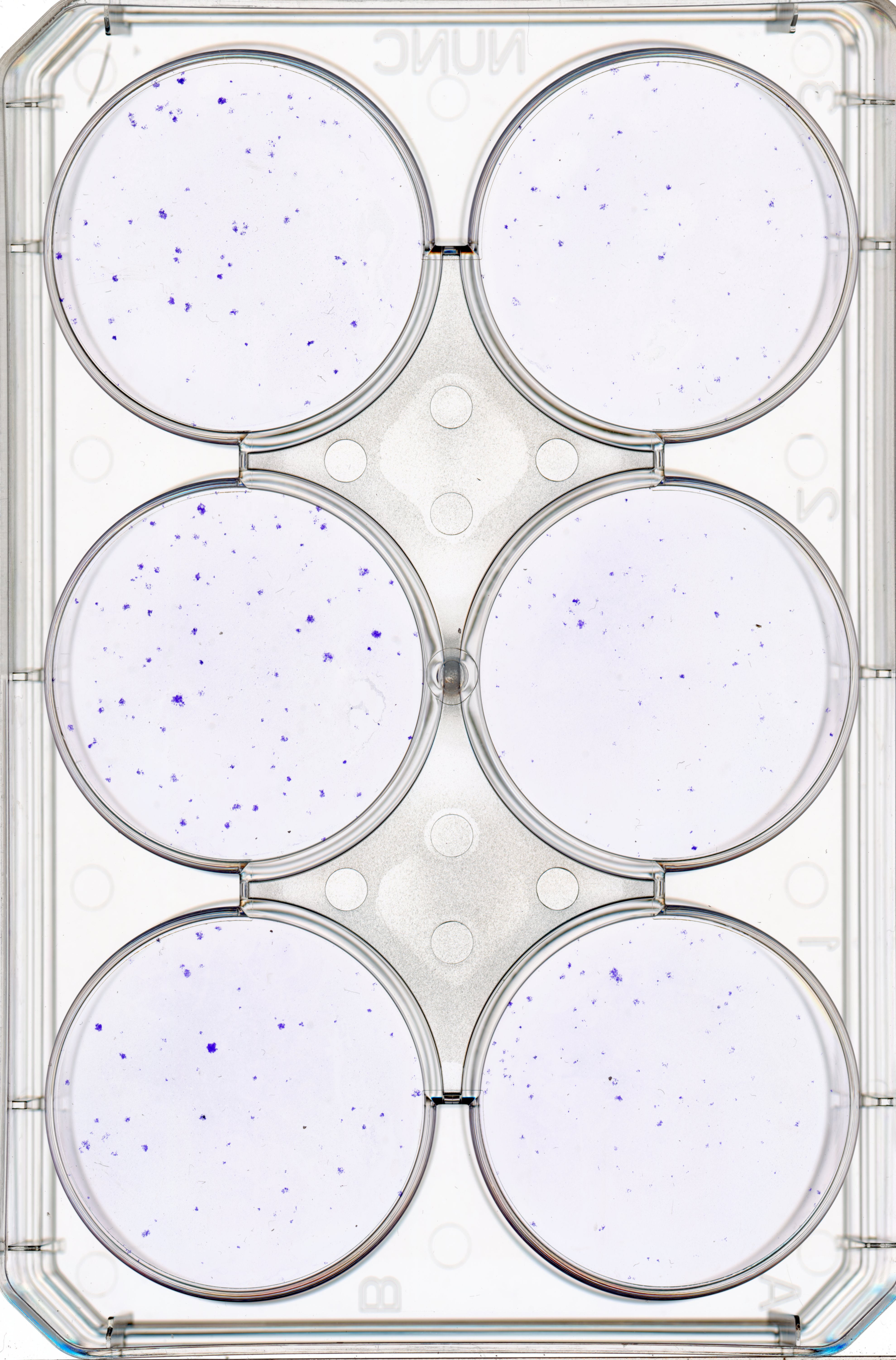

Supplement: Supplementary file 13 — Figure EV5 Source Data [file 44318_2024_108_MOESM13_ESM.zip › EMBOJ-2023-115654_FigEV5_sourcedata/EV5B/E230210 TOPORSsiCtrl 5dC200-300.jpg]

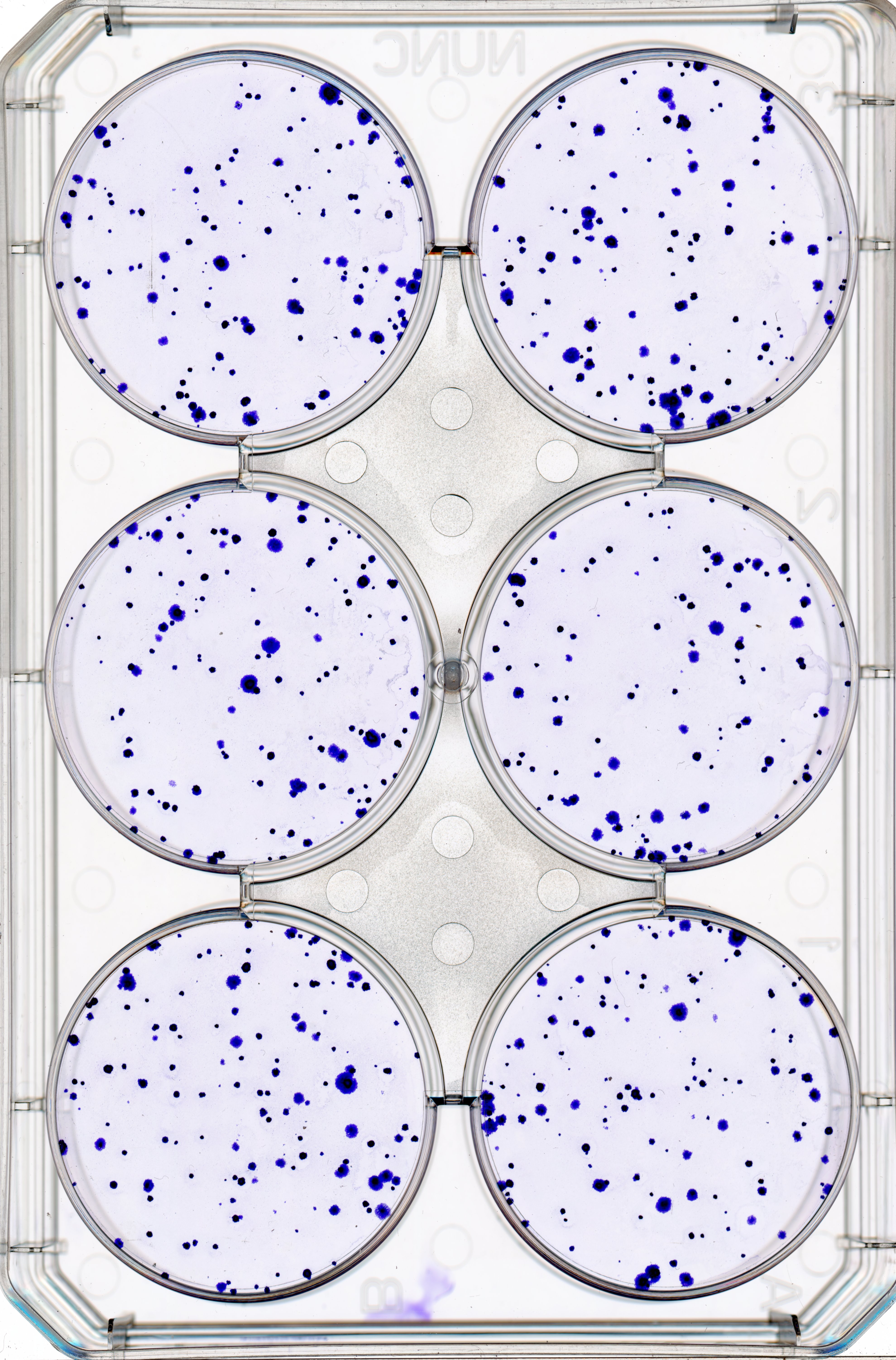

Supplement: Supplementary file 13 — Figure EV5 Source Data [file 44318_2024_108_MOESM13_ESM.zip › EMBOJ-2023-115654_FigEV5_sourcedata/EV5B/E230210 WTsiCtrl 5dC10-20.jpg]

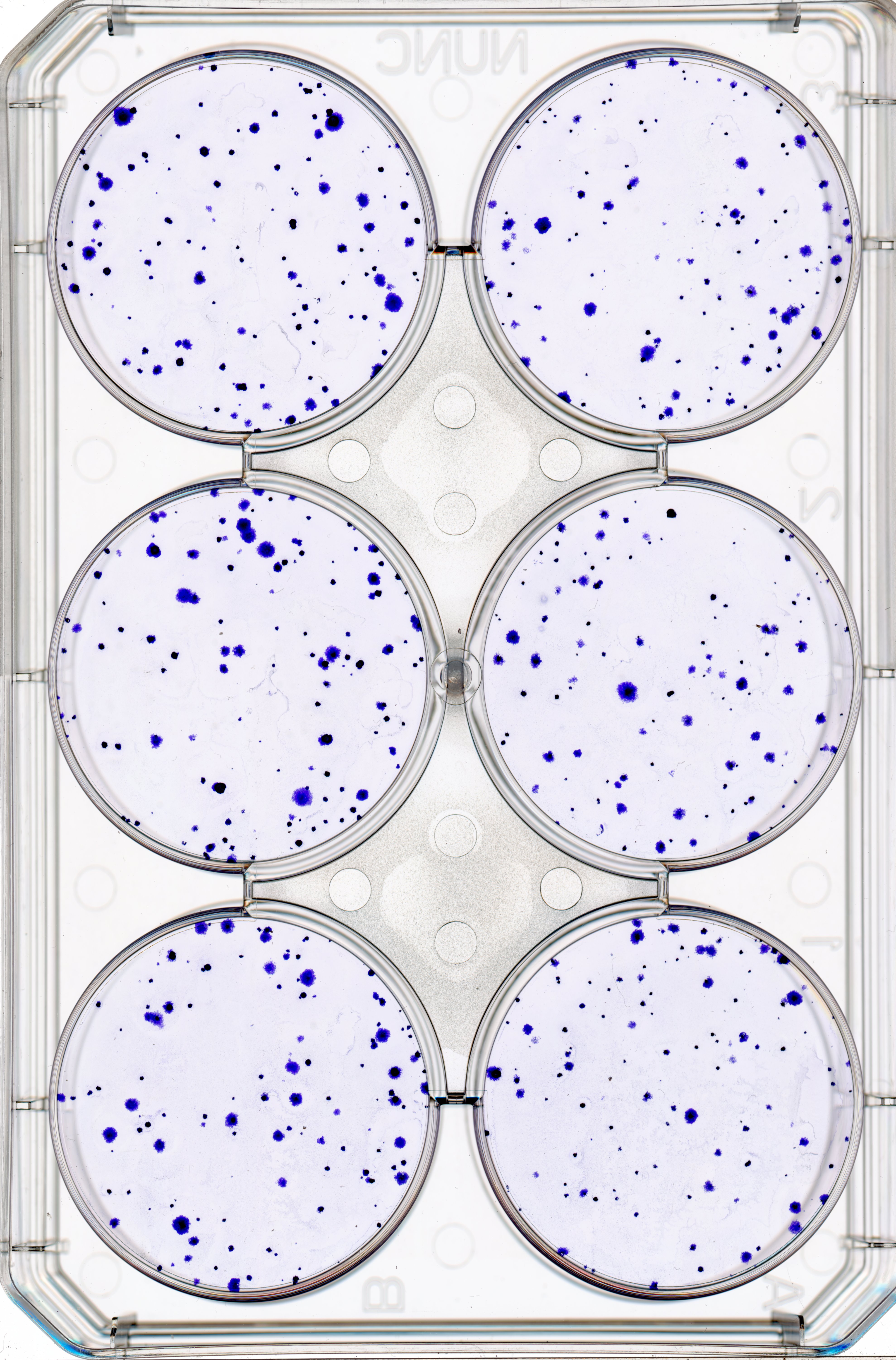

Supplement: Supplementary file 13 — Figure EV5 Source Data [file 44318_2024_108_MOESM13_ESM.zip › EMBOJ-2023-115654_FigEV5_sourcedata/EV5B/E230210 WTsiCtrl 5dC50-100.jpg]

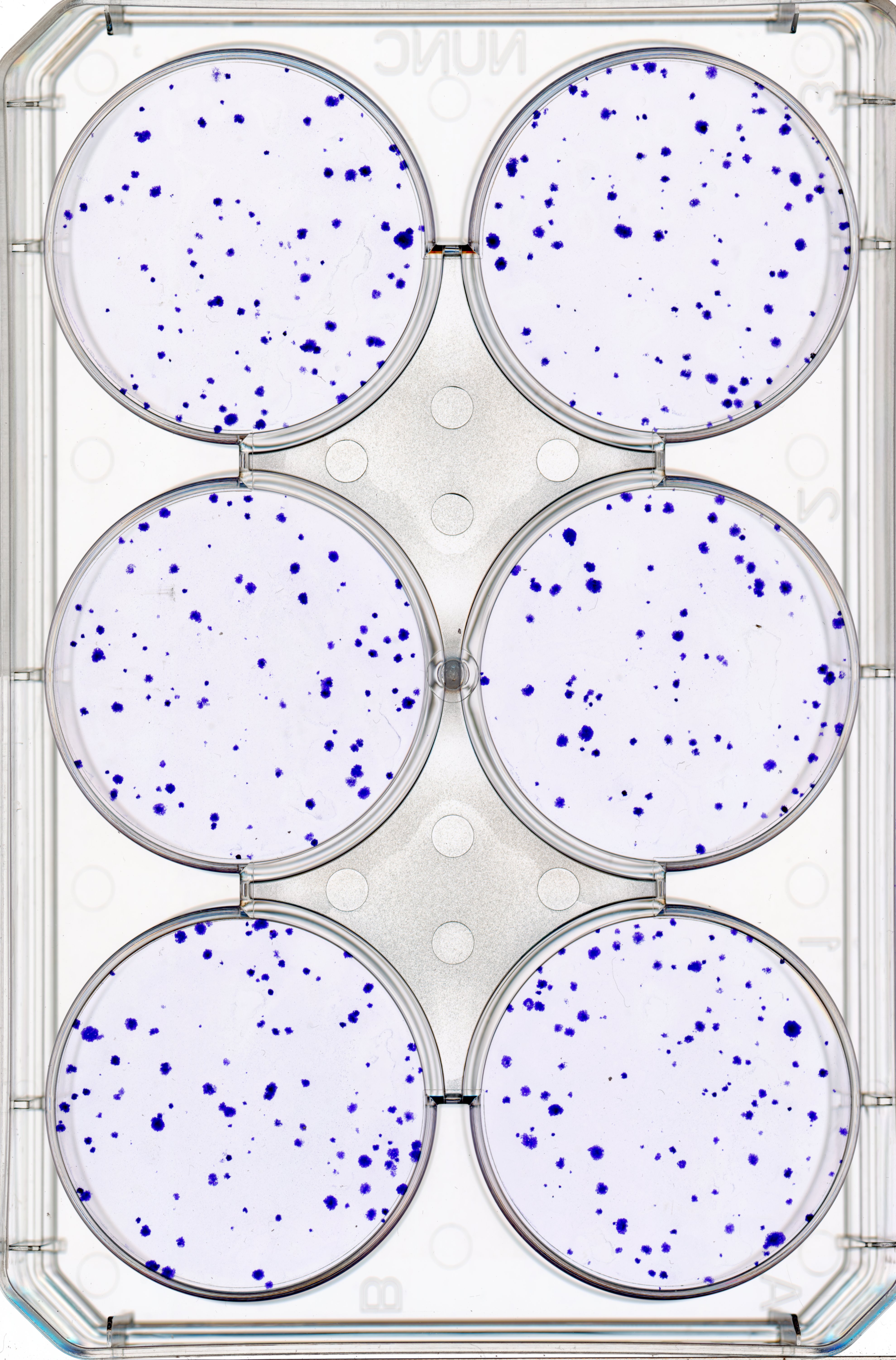

Supplement: Supplementary file 13 — Figure EV5 Source Data [file 44318_2024_108_MOESM13_ESM.zip › EMBOJ-2023-115654_FigEV5_sourcedata/EV5B/E230210 TOPORSsiCtrl 5dC10-20.jpg]

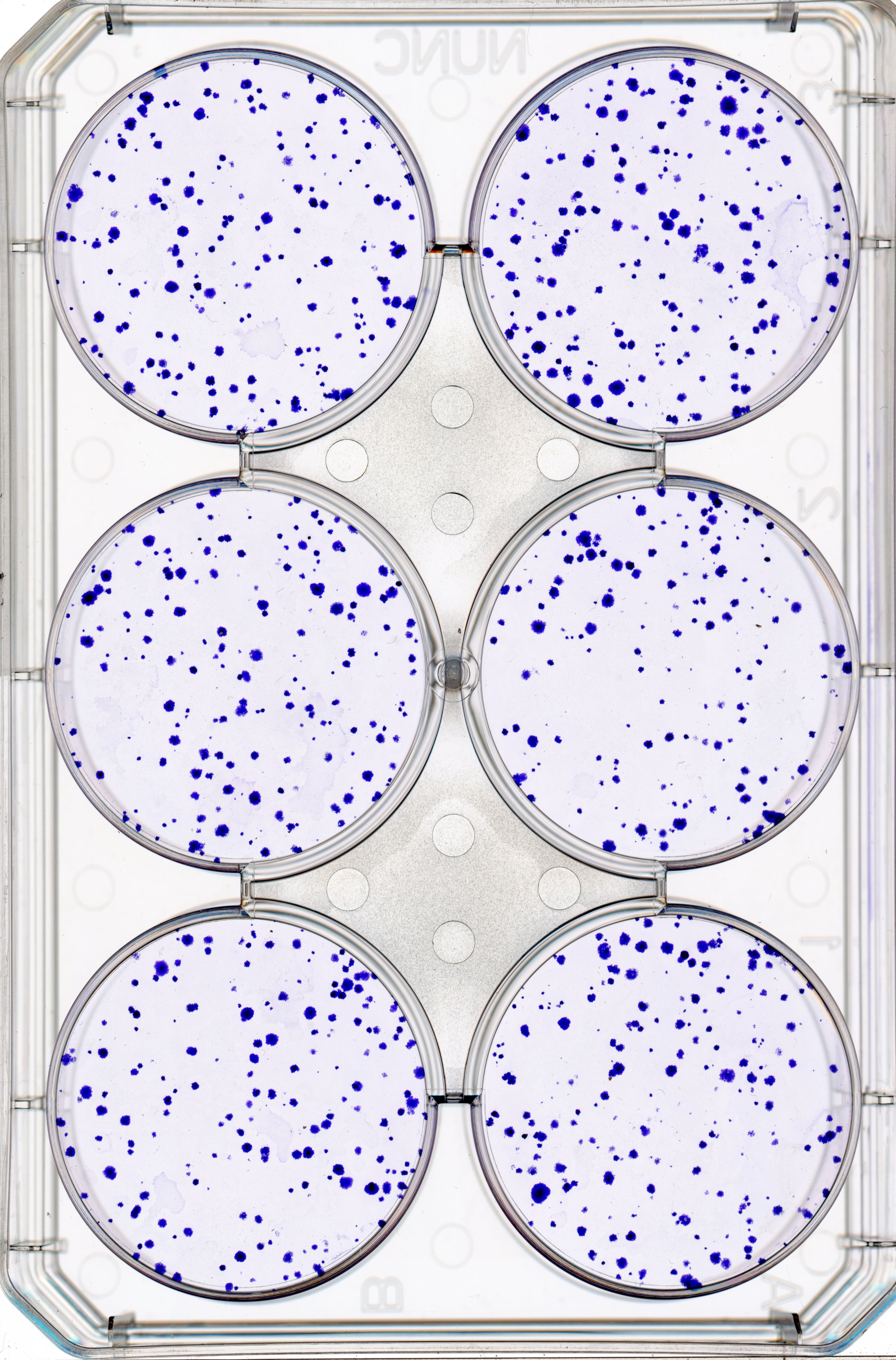

Supplement: Supplementary file 13 — Figure EV5 Source Data [file 44318_2024_108_MOESM13_ESM.zip › EMBOJ-2023-115654_FigEV5_sourcedata/EV5B/E230210 TOPORSsiRNF4 5dC0-5.jpg]

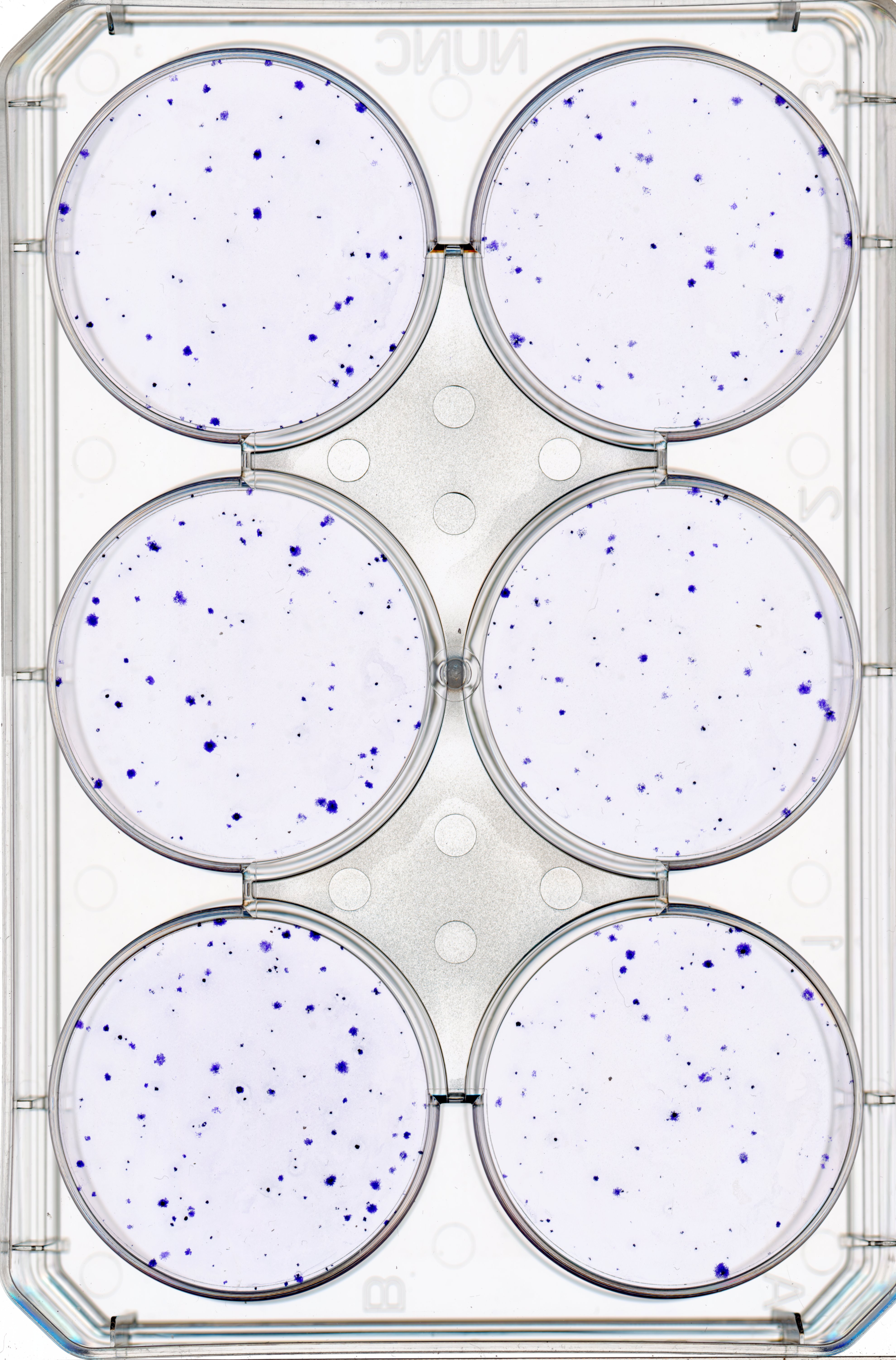

Supplement: Supplementary file 13 — Figure EV5 Source Data [file 44318_2024_108_MOESM13_ESM.zip › EMBOJ-2023-115654_FigEV5_sourcedata/EV5B/E230210 WTsiCtrl 5dC200-300.jpg]

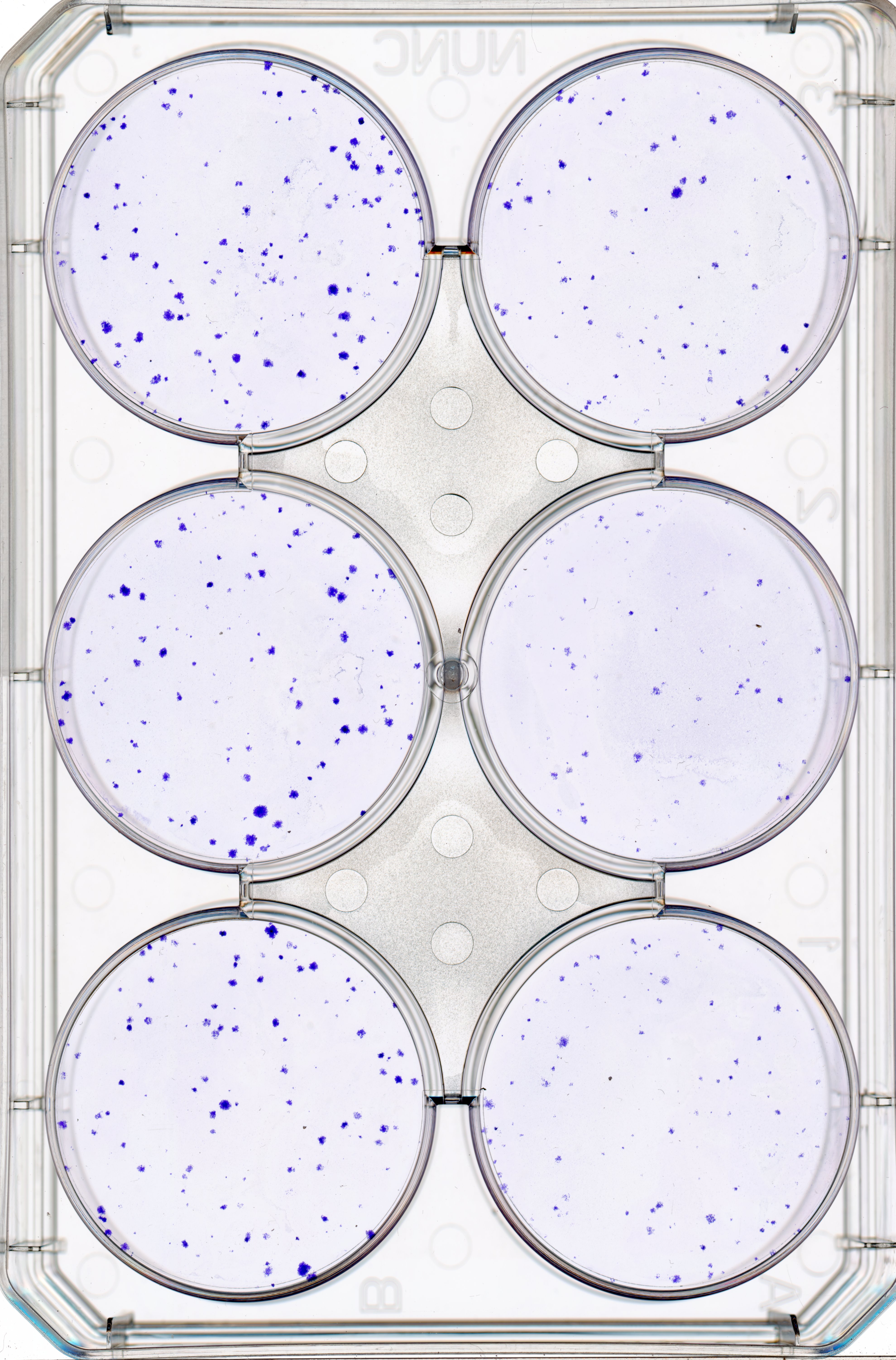

Supplement: Supplementary file 13 — Figure EV5 Source Data [file 44318_2024_108_MOESM13_ESM.zip › EMBOJ-2023-115654_FigEV5_sourcedata/EV5B/E230210 TOPORSsiRNF4 5dC50-100.jpg]

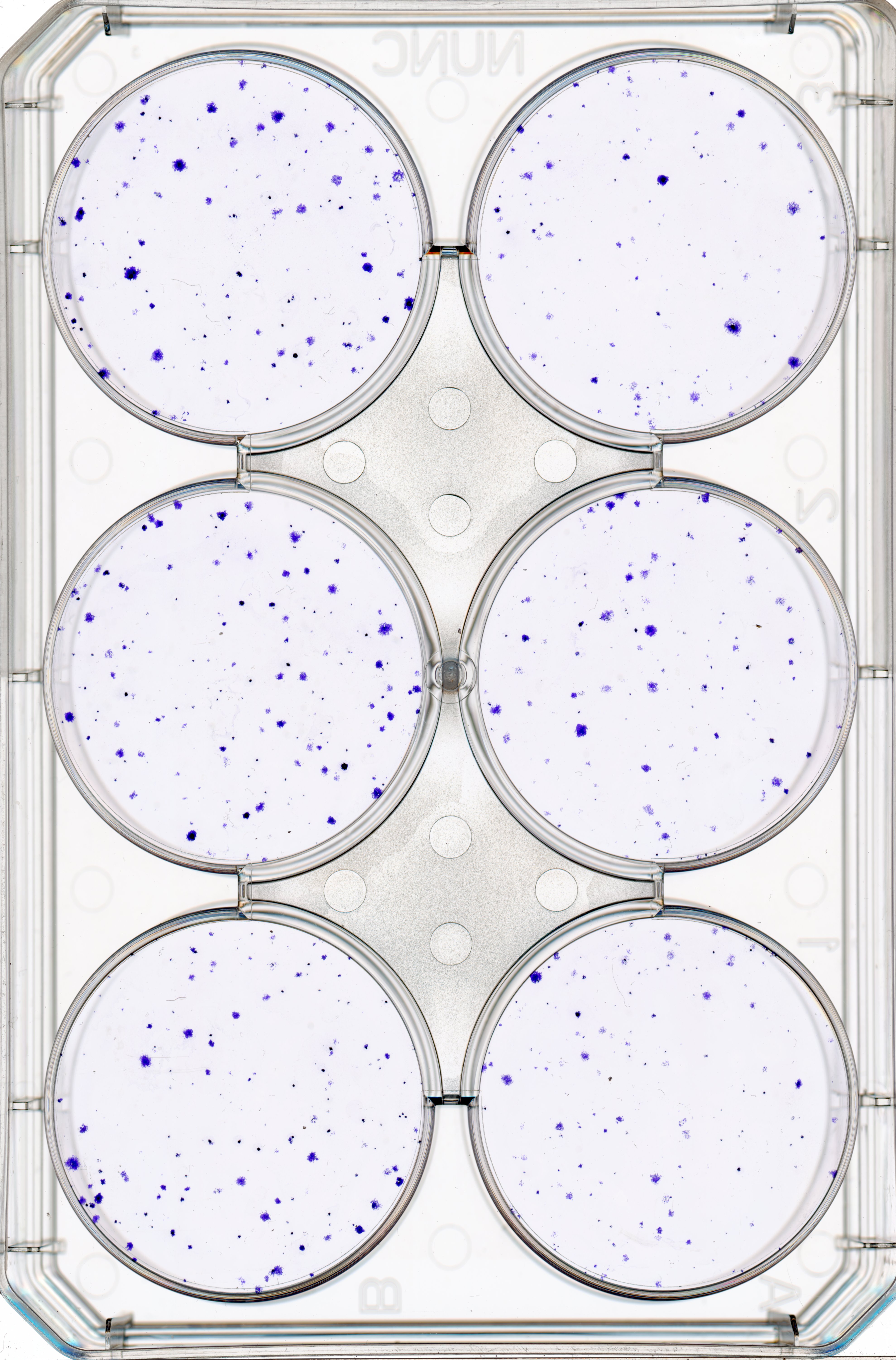

Supplement: Supplementary file 13 — Figure EV5 Source Data [file 44318_2024_108_MOESM13_ESM.zip › EMBOJ-2023-115654_FigEV5_sourcedata/EV5B/E230210 WTsiRNF4 5dC200-300.jpg]

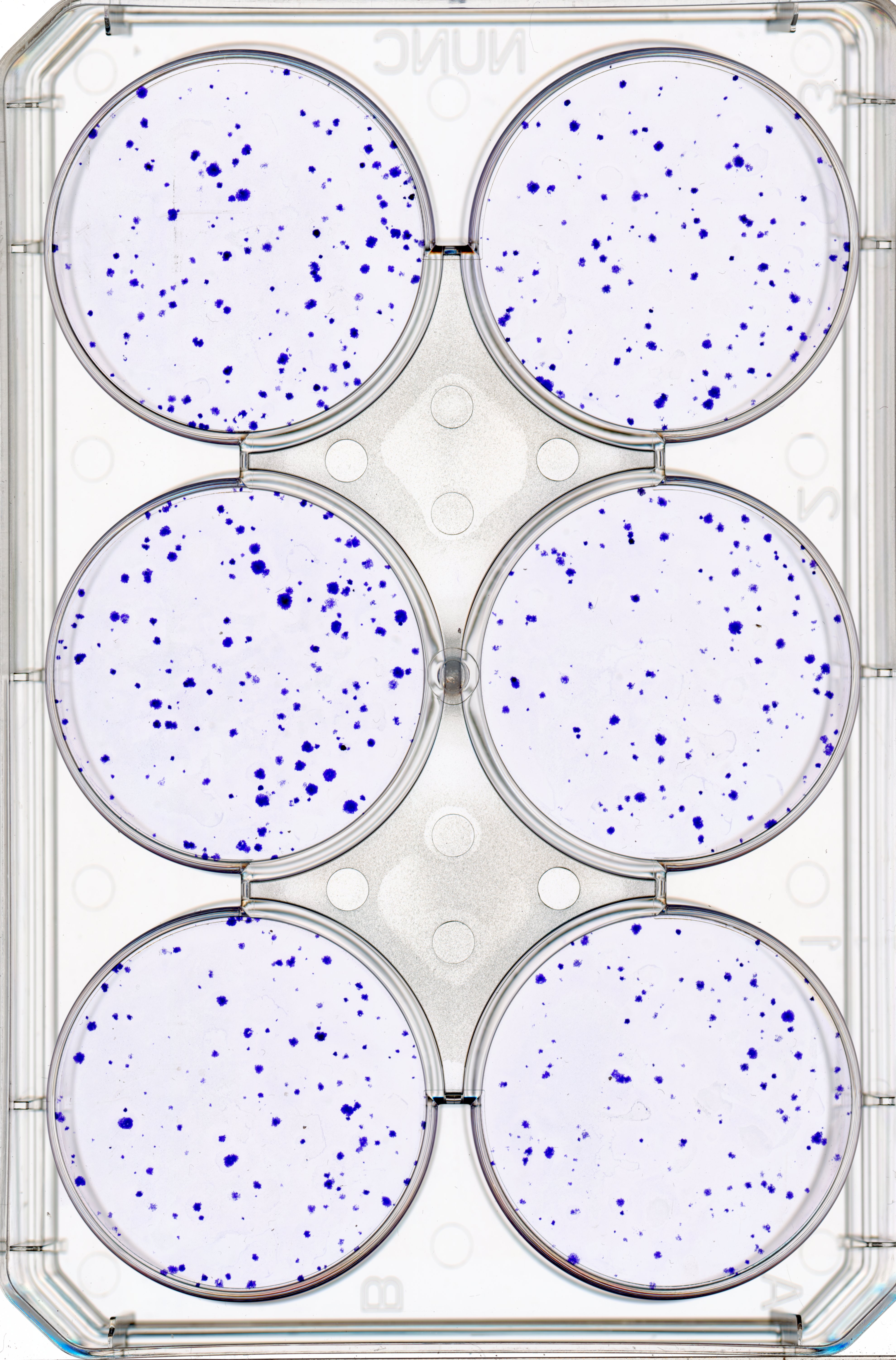

Supplement: Supplementary file 13 — Figure EV5 Source Data [file 44318_2024_108_MOESM13_ESM.zip › EMBOJ-2023-115654_FigEV5_sourcedata/EV5B/E230210 TOPORSsiRNF4 5dC10-20.jpg]

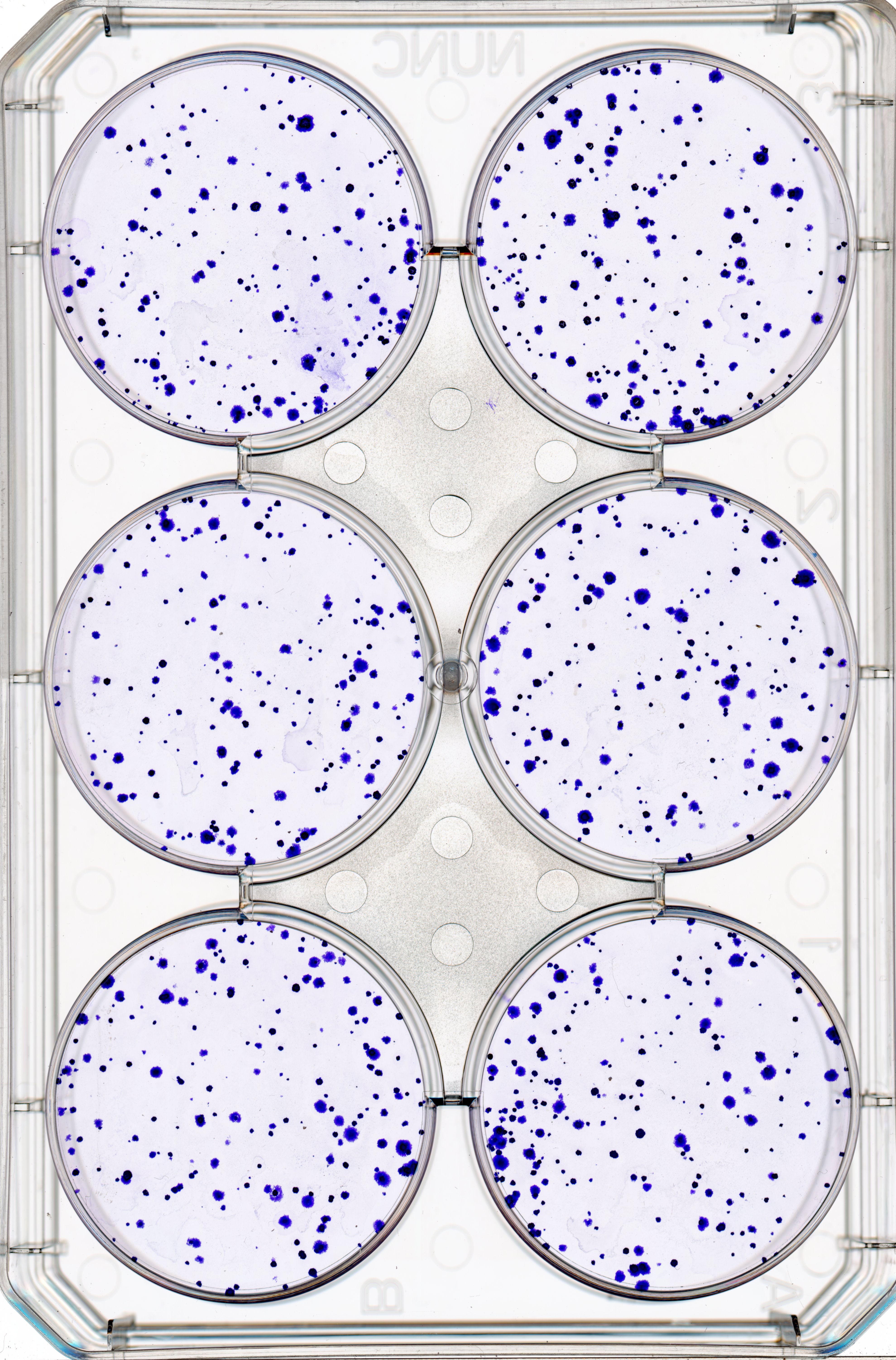

Supplement: Supplementary file 13 — Figure EV5 Source Data [file 44318_2024_108_MOESM13_ESM.zip › EMBOJ-2023-115654_FigEV5_sourcedata/EV5B/E230210 WTsiRNF4 5dC10-20.jpg]

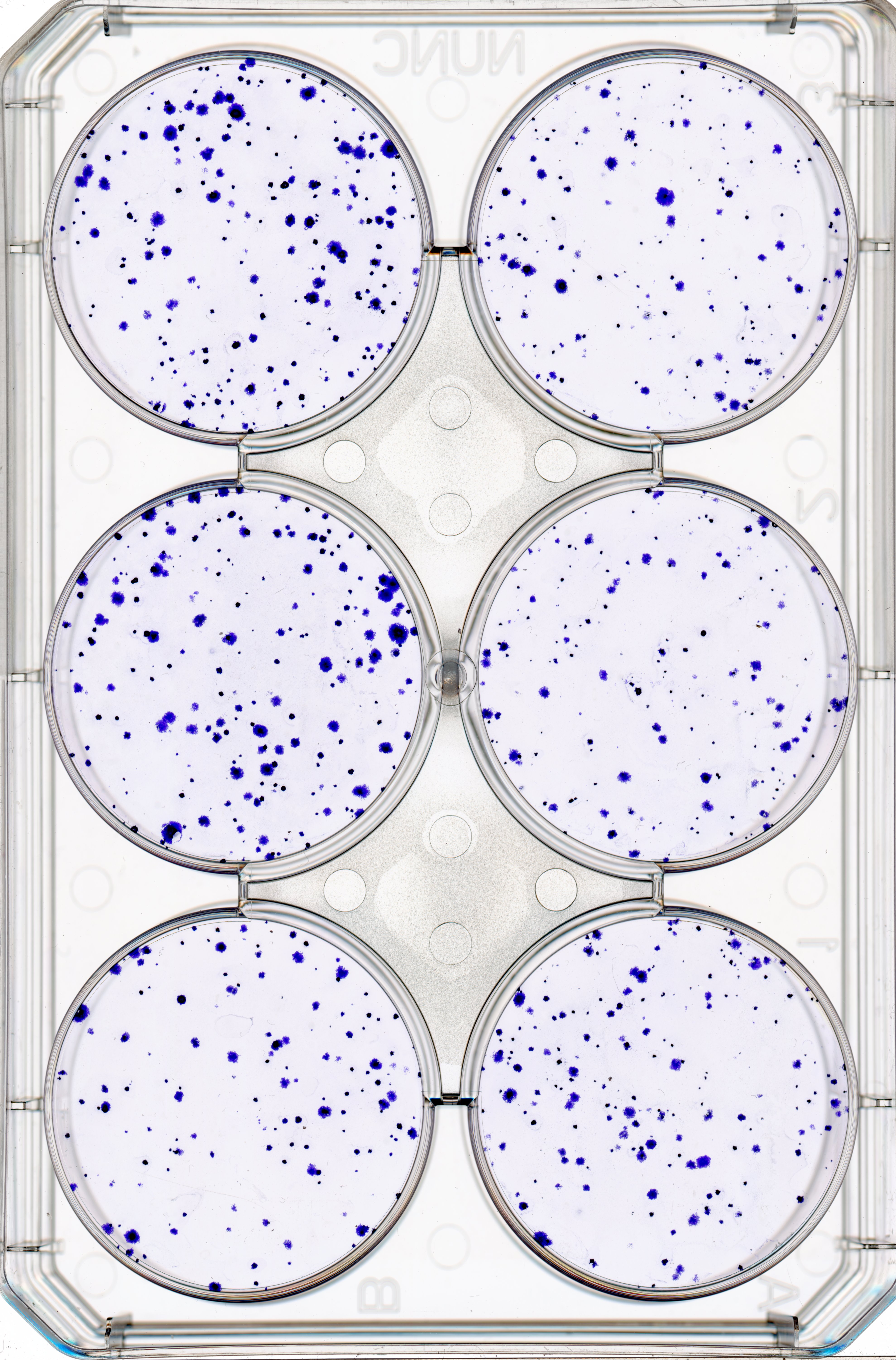

Supplement: Supplementary file 13 — Figure EV5 Source Data [file 44318_2024_108_MOESM13_ESM.zip › EMBOJ-2023-115654_FigEV5_sourcedata/EV5B/E230210 WTsiRNF4 5dC50-100.jpg]

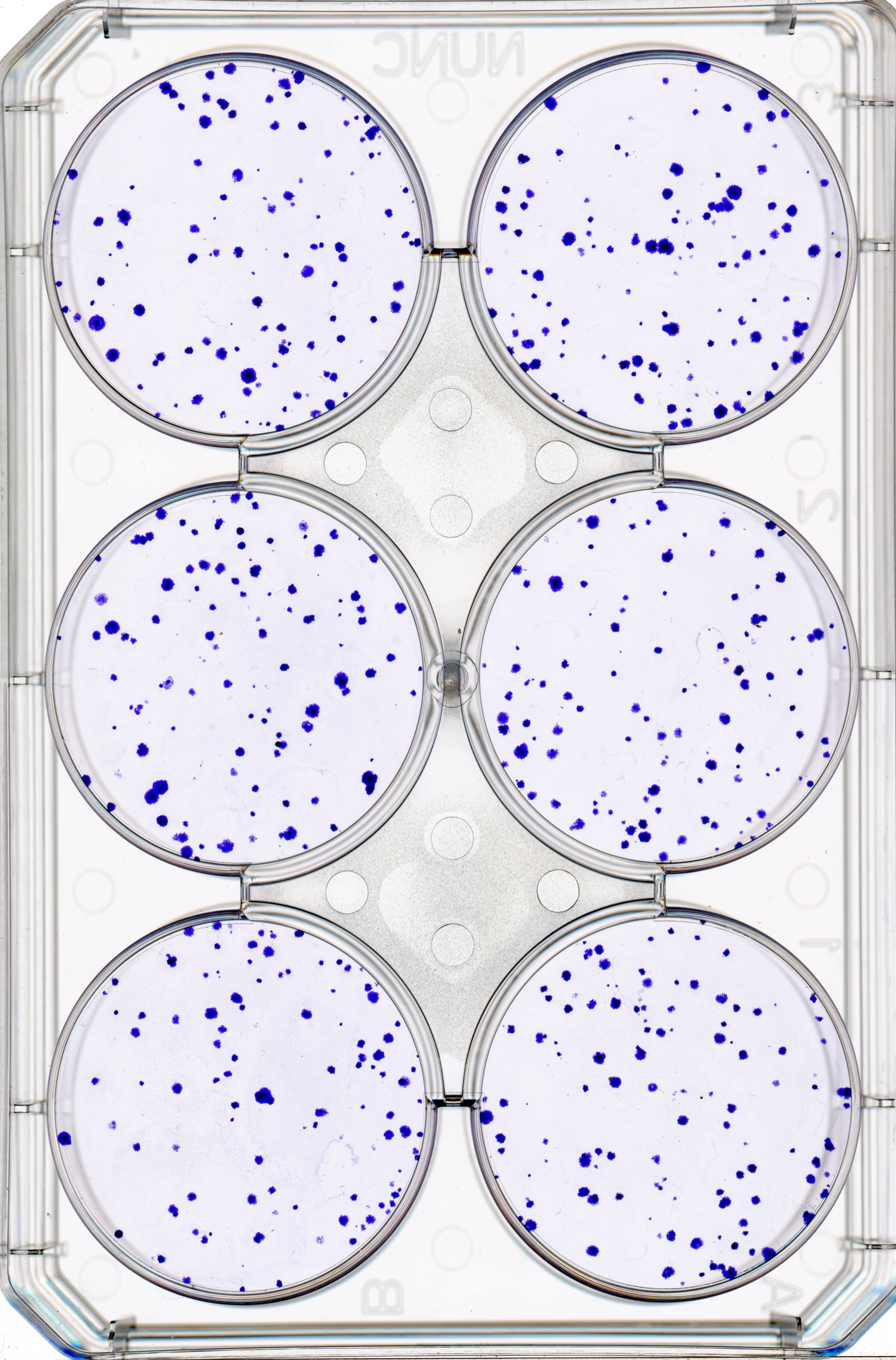

Supplement: Supplementary file 13 — Figure EV5 Source Data [file 44318_2024_108_MOESM13_ESM.zip › EMBOJ-2023-115654_FigEV5_sourcedata/EV5B/E230210 TOPORSsiCtrl 5dC0-5.jpg]

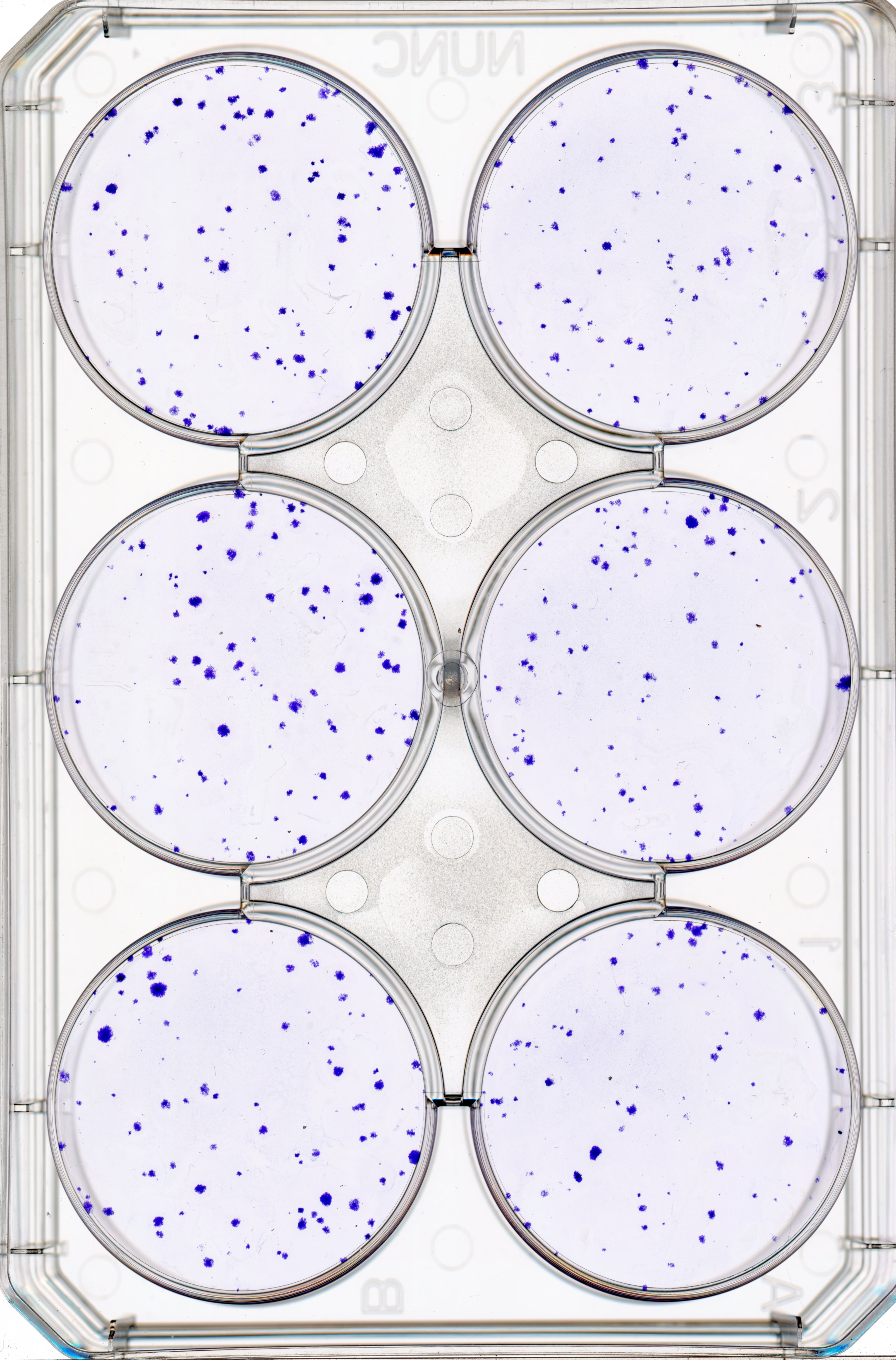

Supplement: Supplementary file 13 — Figure EV5 Source Data [file 44318_2024_108_MOESM13_ESM.zip › EMBOJ-2023-115654_FigEV5_sourcedata/EV5B/E230210 TOPORSsiCtrl 5dC50-100.jpg]

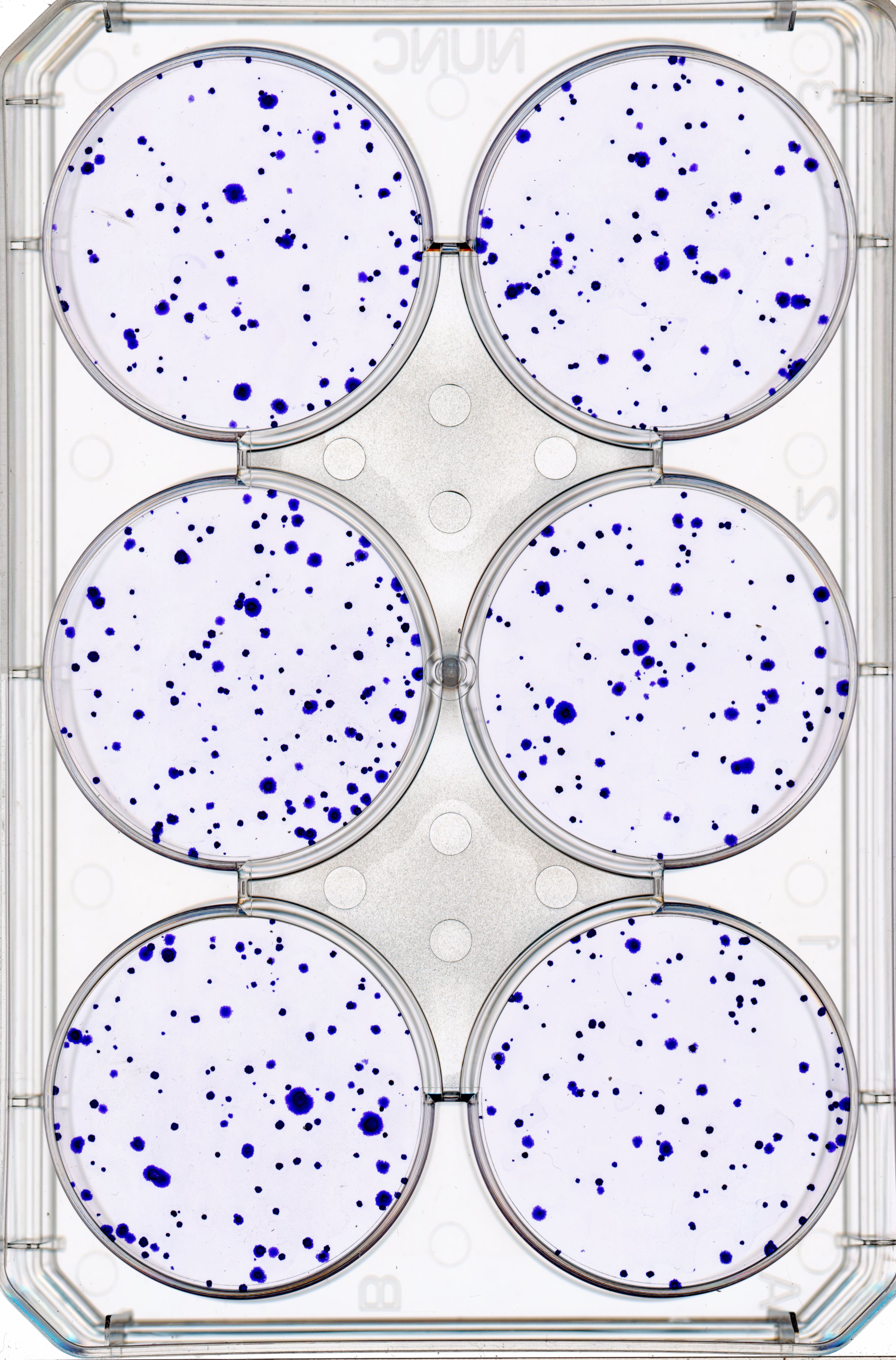

Supplement: Supplementary file 13 — Figure EV5 Source Data [file 44318_2024_108_MOESM13_ESM.zip › EMBOJ-2023-115654_FigEV5_sourcedata/EV5B/E230210 WTsiCtrl 5dC0-5.jpg]

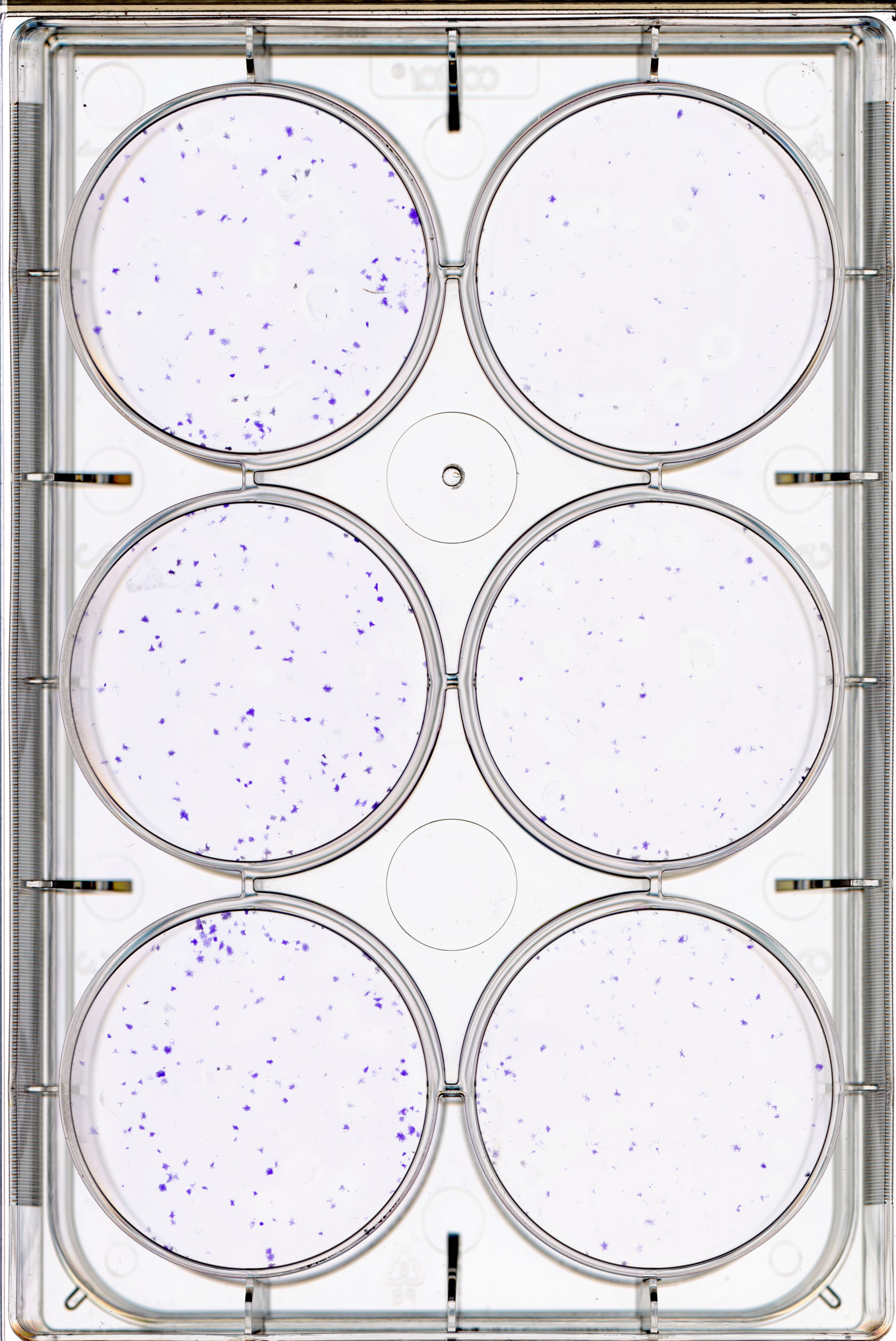

Supplement: Supplementary file 13 — Figure EV5 Source Data [file 44318_2024_108_MOESM13_ESM.zip › EMBOJ-2023-115654_FigEV5_sourcedata/EV5C/RNF4 KO_0_20.jpg]

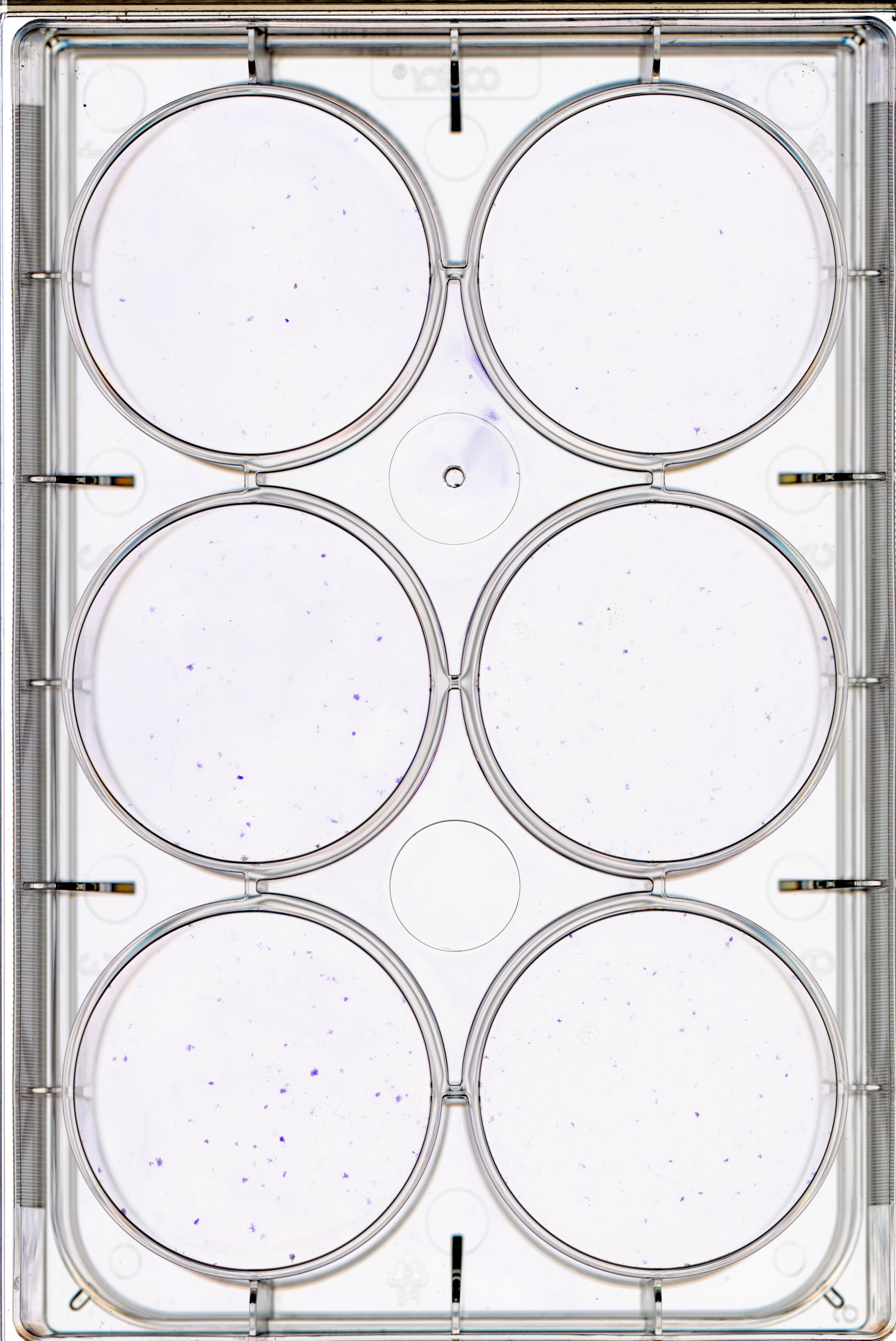

Supplement: Supplementary file 13 — Figure EV5 Source Data [file 44318_2024_108_MOESM13_ESM.zip › EMBOJ-2023-115654_FigEV5_sourcedata/EV5C/RNF4 KO_50_100.jpg]

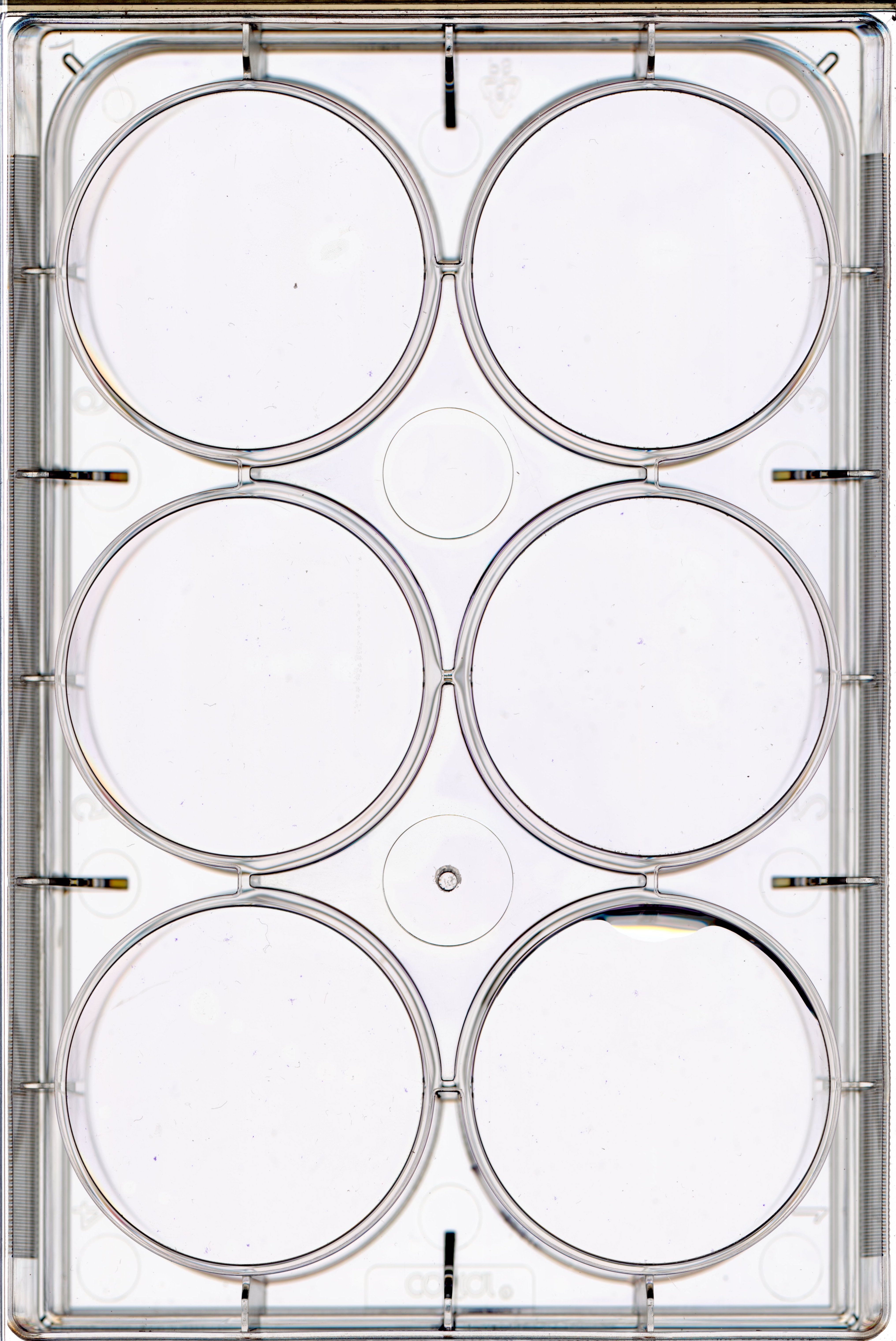

Supplement: Supplementary file 13 — Figure EV5 Source Data [file 44318_2024_108_MOESM13_ESM.zip › EMBOJ-2023-115654_FigEV5_sourcedata/EV5C/RNF4 KO siTOPORS_50_100.jpg]

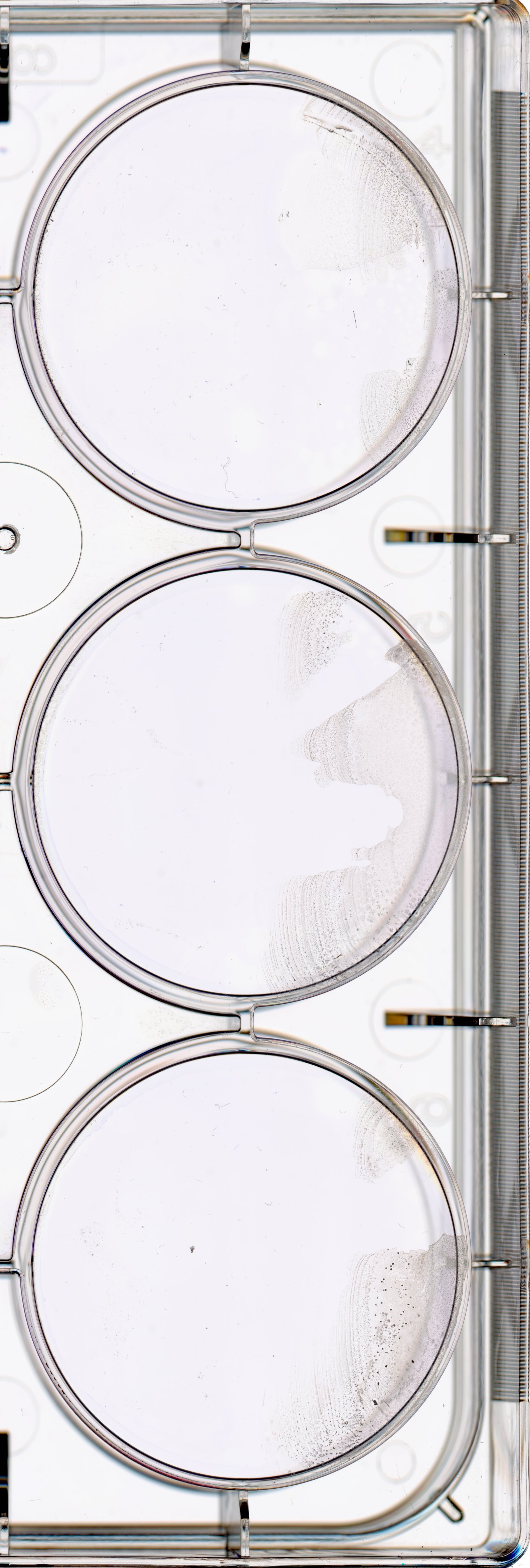

Supplement: Supplementary file 13 — Figure EV5 Source Data [file 44318_2024_108_MOESM13_ESM.zip › EMBOJ-2023-115654_FigEV5_sourcedata/EV5C/RNF4 KO siTOPORS_200.jpg]

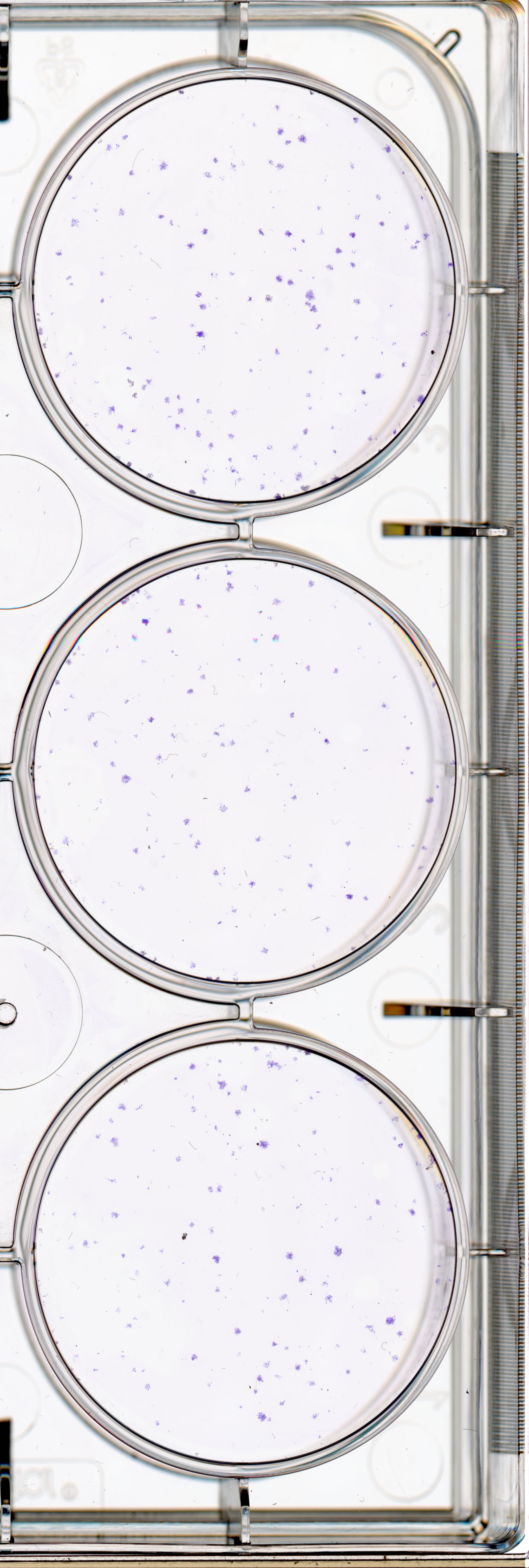

Supplement: Supplementary file 13 — Figure EV5 Source Data [file 44318_2024_108_MOESM13_ESM.zip › EMBOJ-2023-115654_FigEV5_sourcedata/EV5C/Hela WT_200.jpg]

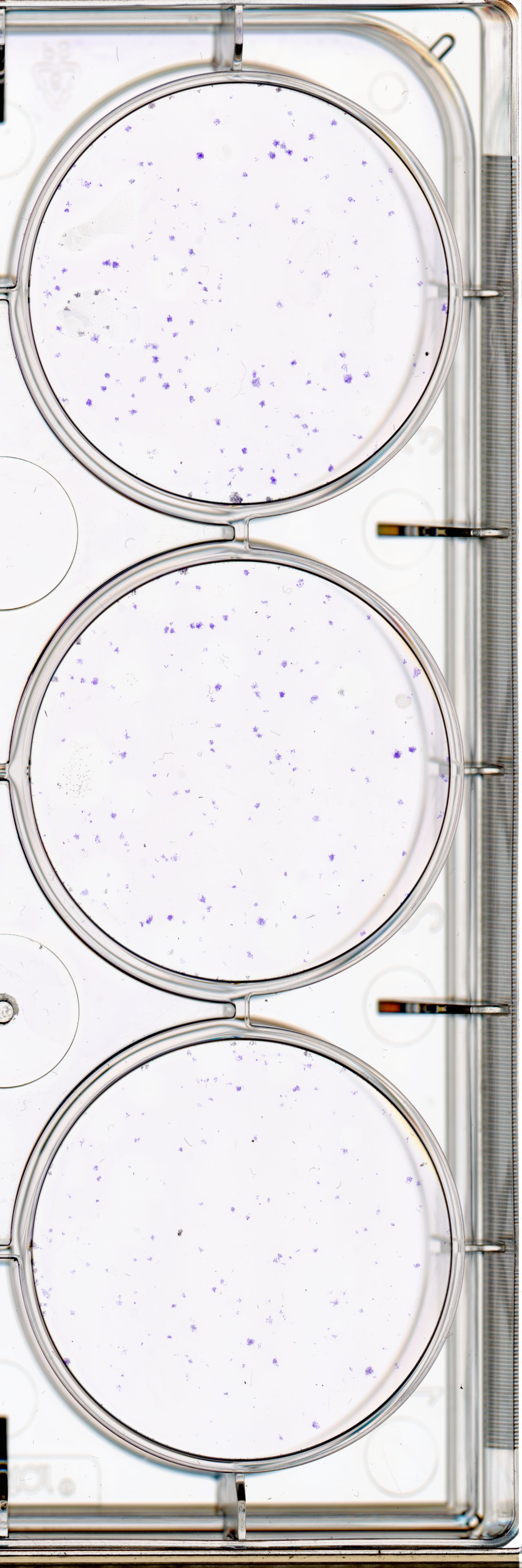

Supplement: Supplementary file 13 — Figure EV5 Source Data [file 44318_2024_108_MOESM13_ESM.zip › EMBOJ-2023-115654_FigEV5_sourcedata/EV5C/Hela siTOPORS_200.jpg]

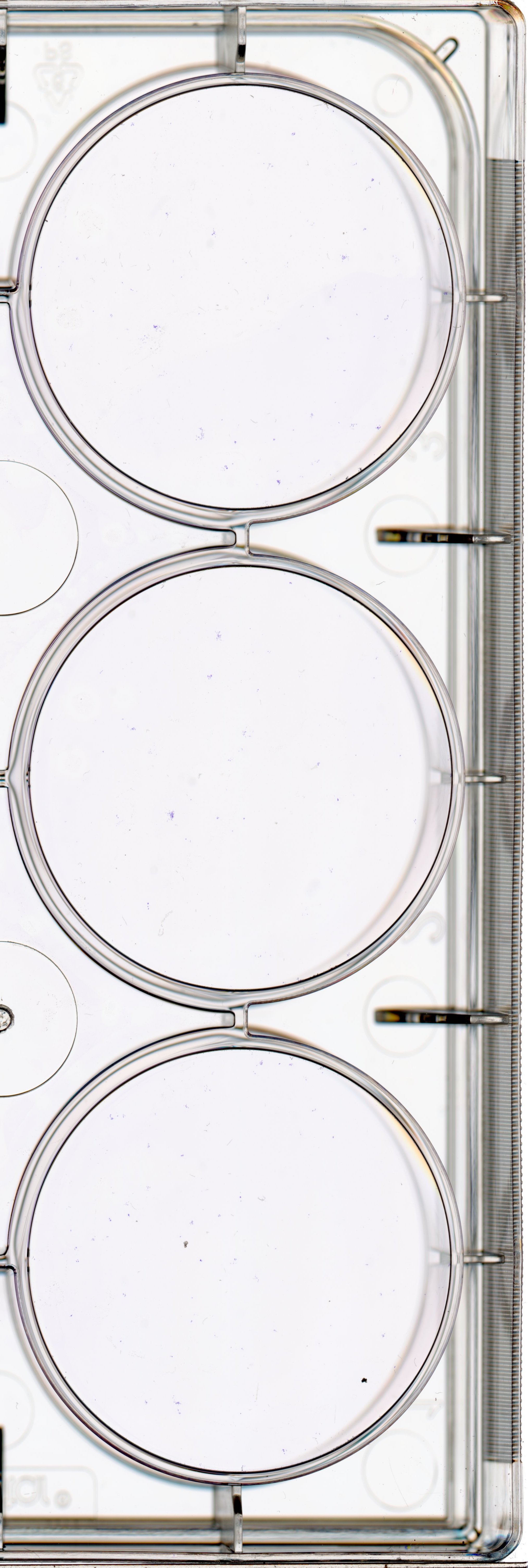

Supplement: Supplementary file 13 — Figure EV5 Source Data [file 44318_2024_108_MOESM13_ESM.zip › EMBOJ-2023-115654_FigEV5_sourcedata/EV5C/RNF4 KO_200.jpg]

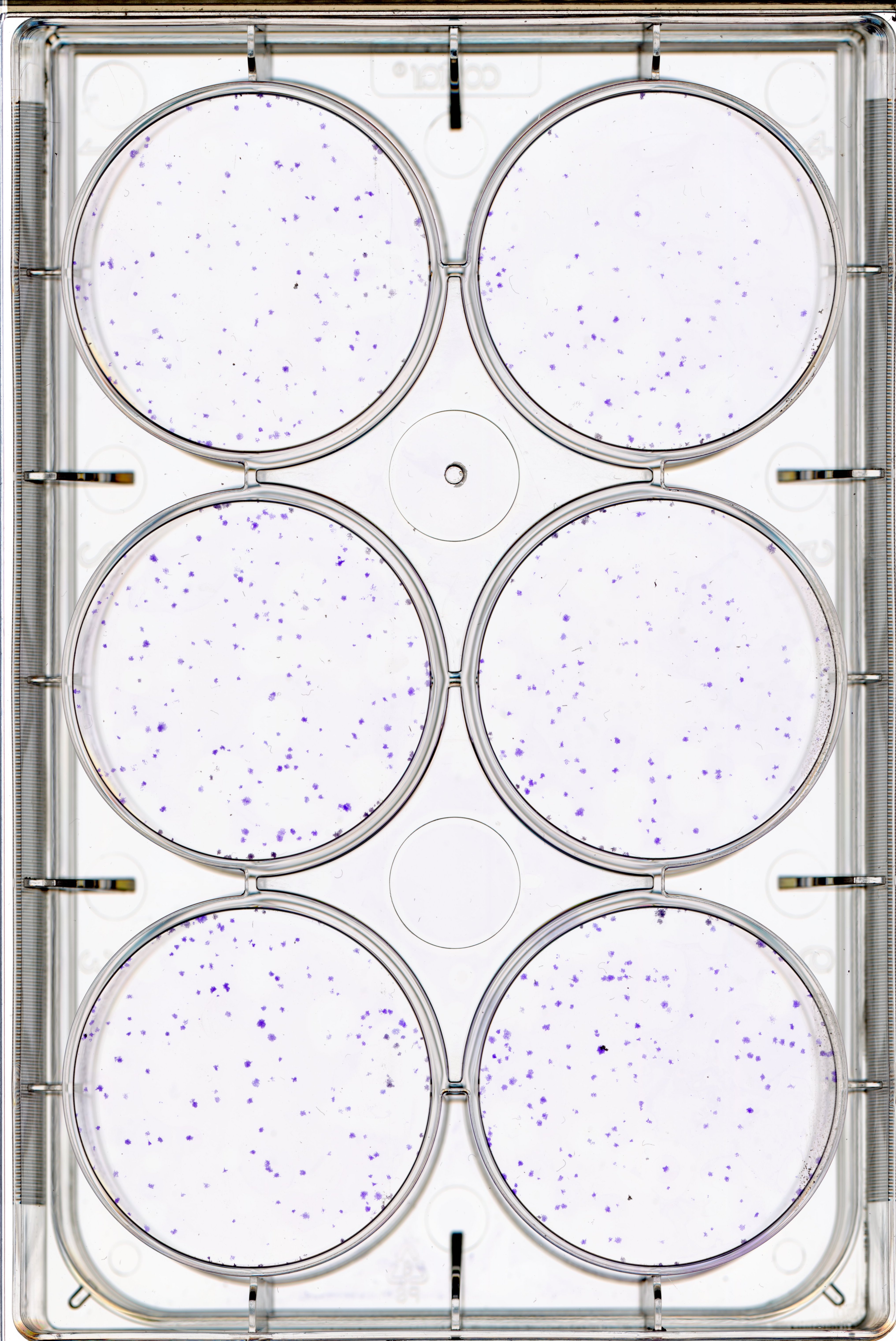

Supplement: Supplementary file 13 — Figure EV5 Source Data [file 44318_2024_108_MOESM13_ESM.zip › EMBOJ-2023-115654_FigEV5_sourcedata/EV5C/Hela WT_50_100.jpg]

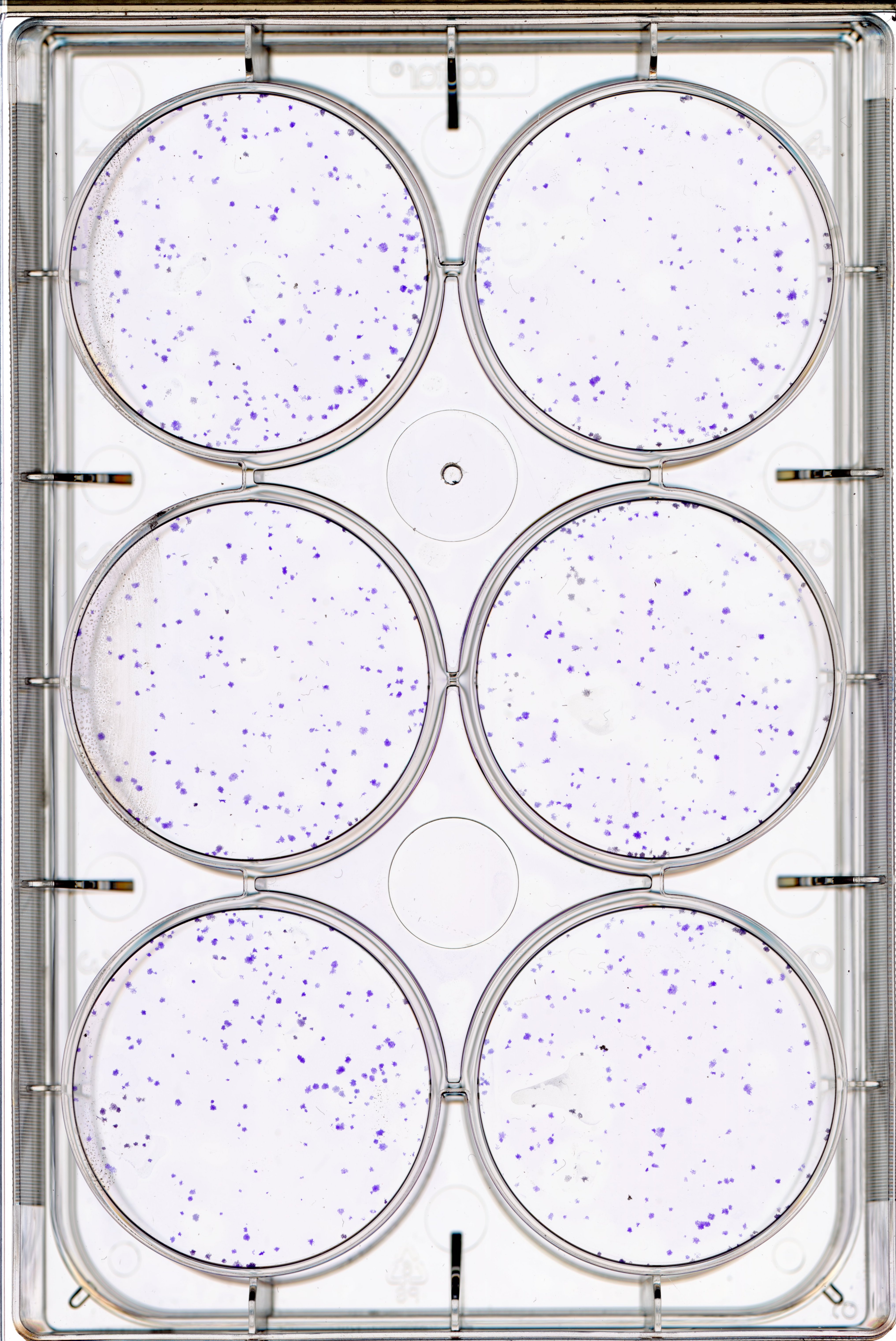

Supplement: Supplementary file 13 — Figure EV5 Source Data [file 44318_2024_108_MOESM13_ESM.zip › EMBOJ-2023-115654_FigEV5_sourcedata/EV5C/Hela siTOPORS_0_20.jpg]

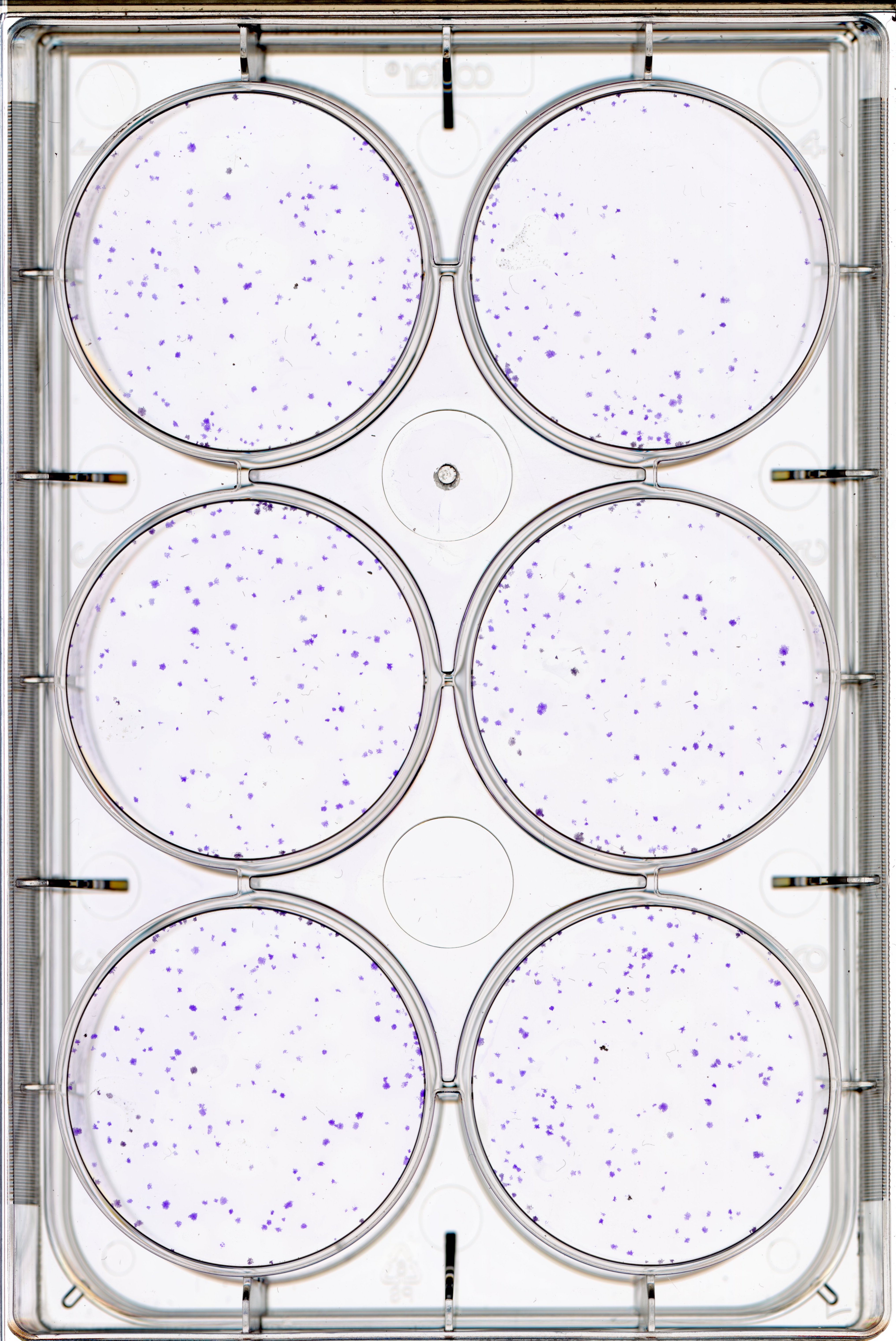

Supplement: Supplementary file 13 — Figure EV5 Source Data [file 44318_2024_108_MOESM13_ESM.zip › EMBOJ-2023-115654_FigEV5_sourcedata/EV5C/Hela WT_0_20.jpg]

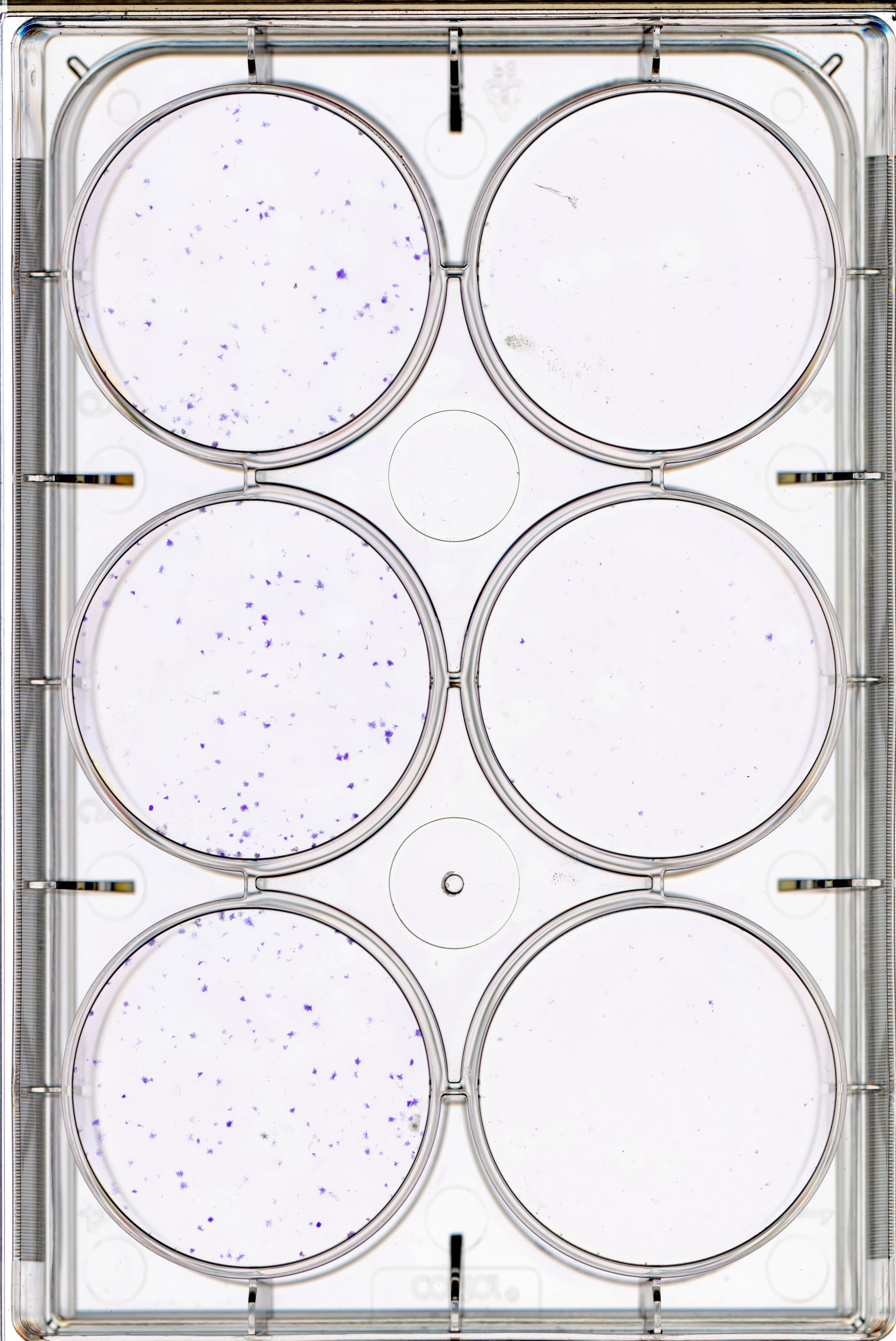

Supplement: Supplementary file 13 — Figure EV5 Source Data [file 44318_2024_108_MOESM13_ESM.zip › EMBOJ-2023-115654_FigEV5_sourcedata/EV5C/RNF4 KO siTOPORS_0_20.jpg]

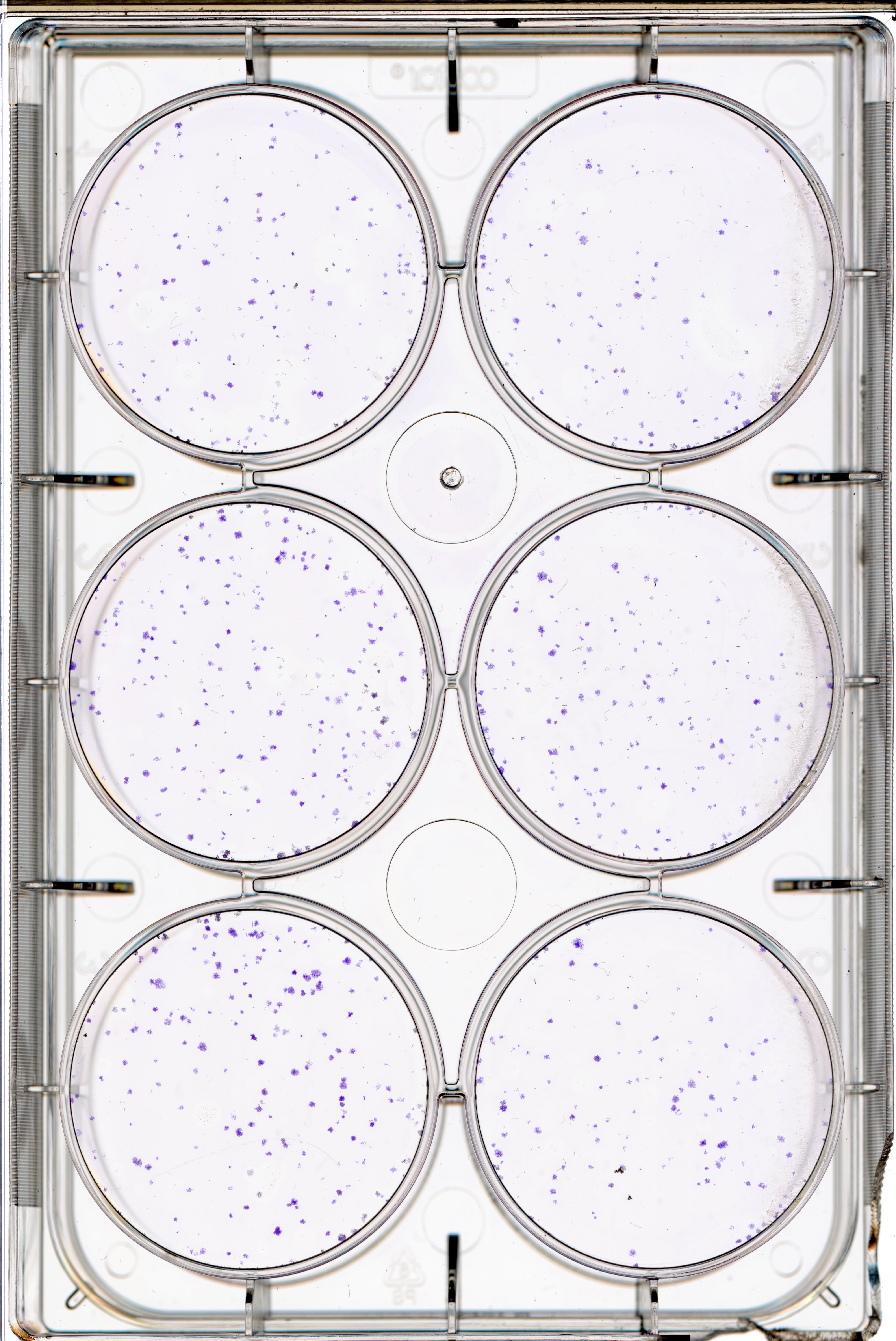

Supplement: Supplementary file 13 — Figure EV5 Source Data [file 44318_2024_108_MOESM13_ESM.zip › EMBOJ-2023-115654_FigEV5_sourcedata/EV5C/Hela siTOPORS_50_100.jpg]

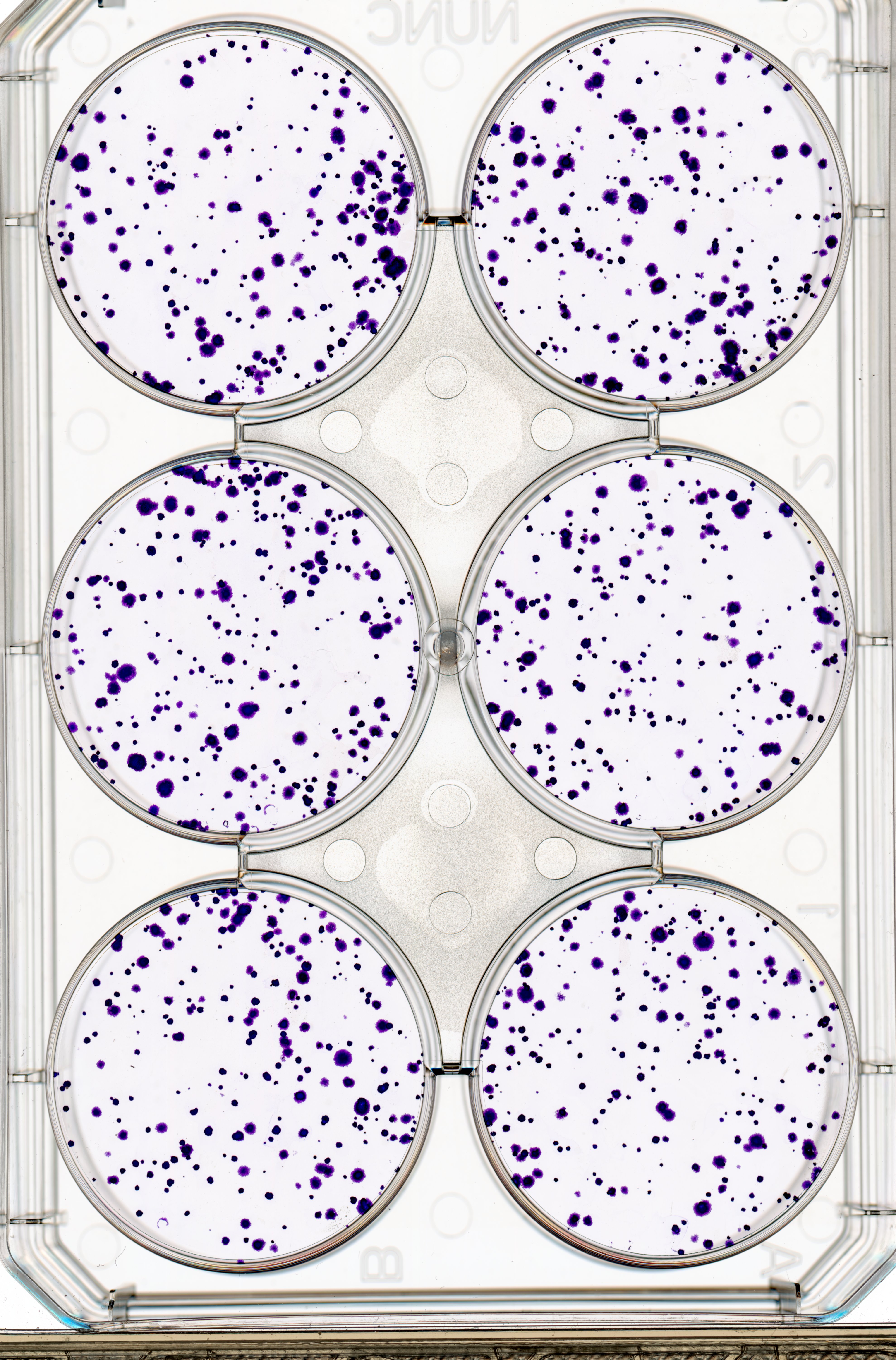

Supplement: Supplementary file 13 — Figure EV5 Source Data [file 44318_2024_108_MOESM13_ESM.zip › EMBOJ-2023-115654_FigEV5_sourcedata/EV5J/E230213 WTsiRNF4 5dC0-5.jpg]

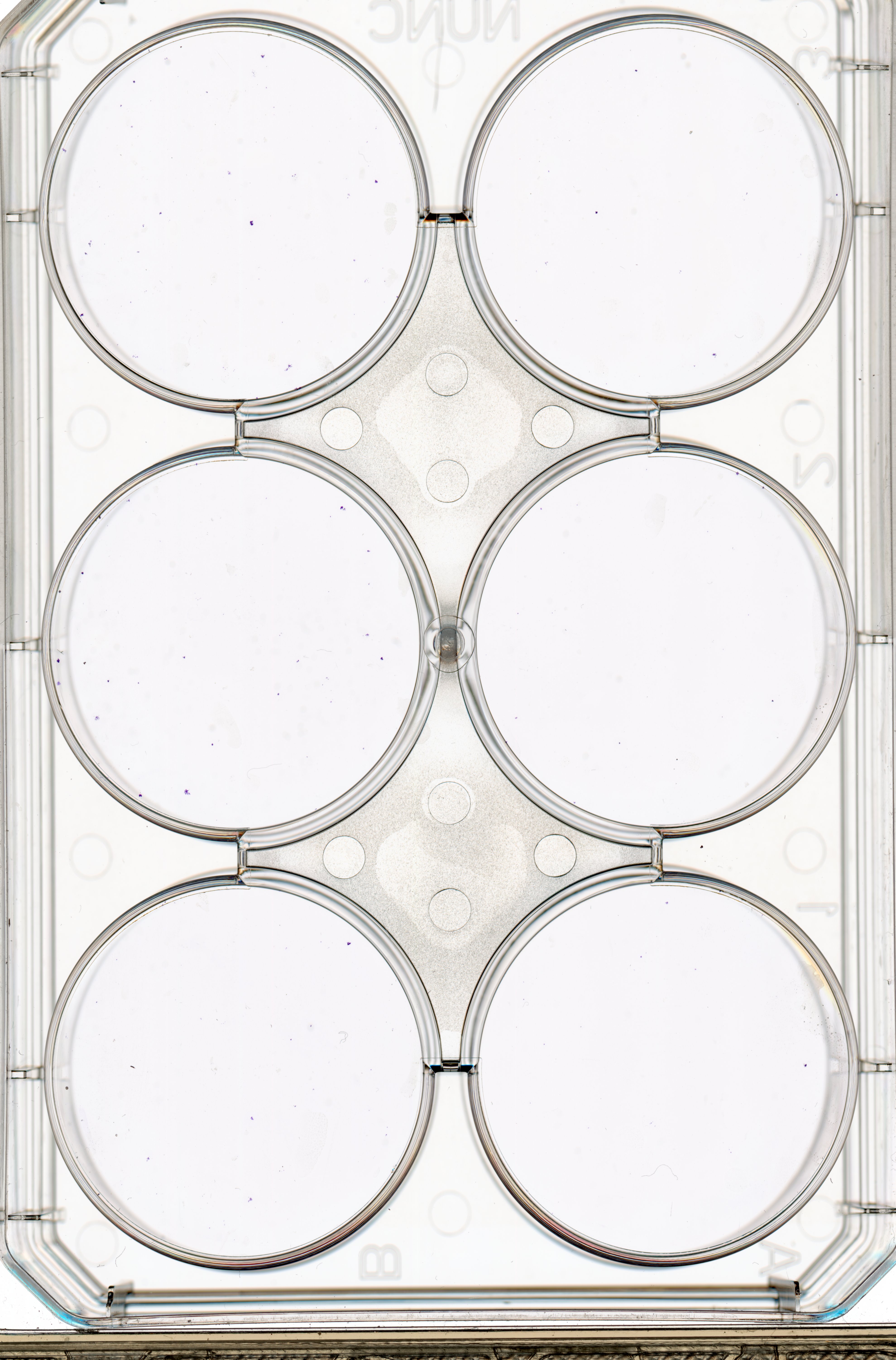

Supplement: Supplementary file 13 — Figure EV5 Source Data [file 44318_2024_108_MOESM13_ESM.zip › EMBOJ-2023-115654_FigEV5_sourcedata/EV5J/E230213 UBE2KsiCtrl 5dC200-300.jpg]

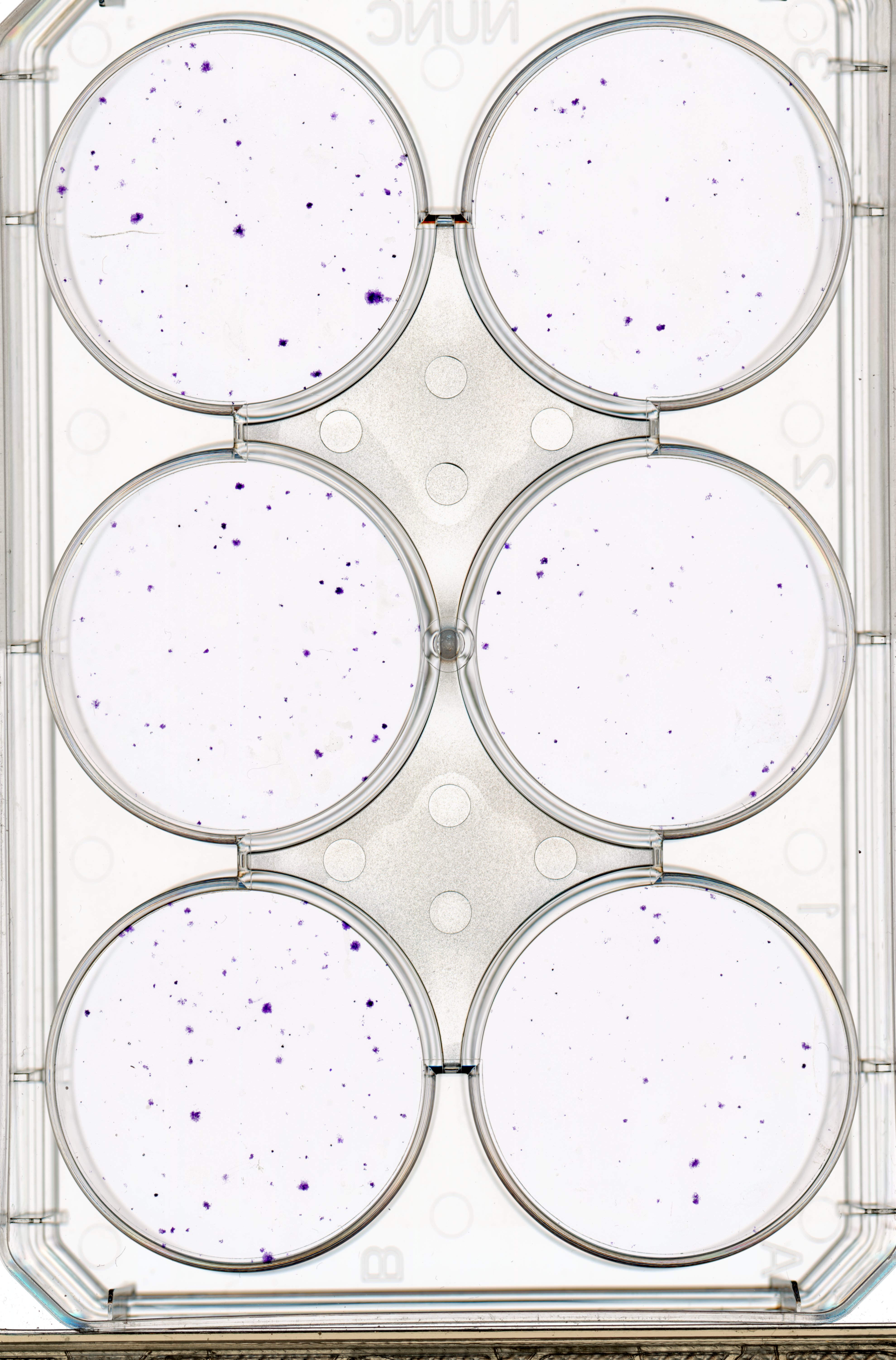

Supplement: Supplementary file 13 — Figure EV5 Source Data [file 44318_2024_108_MOESM13_ESM.zip › EMBOJ-2023-115654_FigEV5_sourcedata/EV5J/E230213 WTsiRNF4 5dC200-300.jpg]

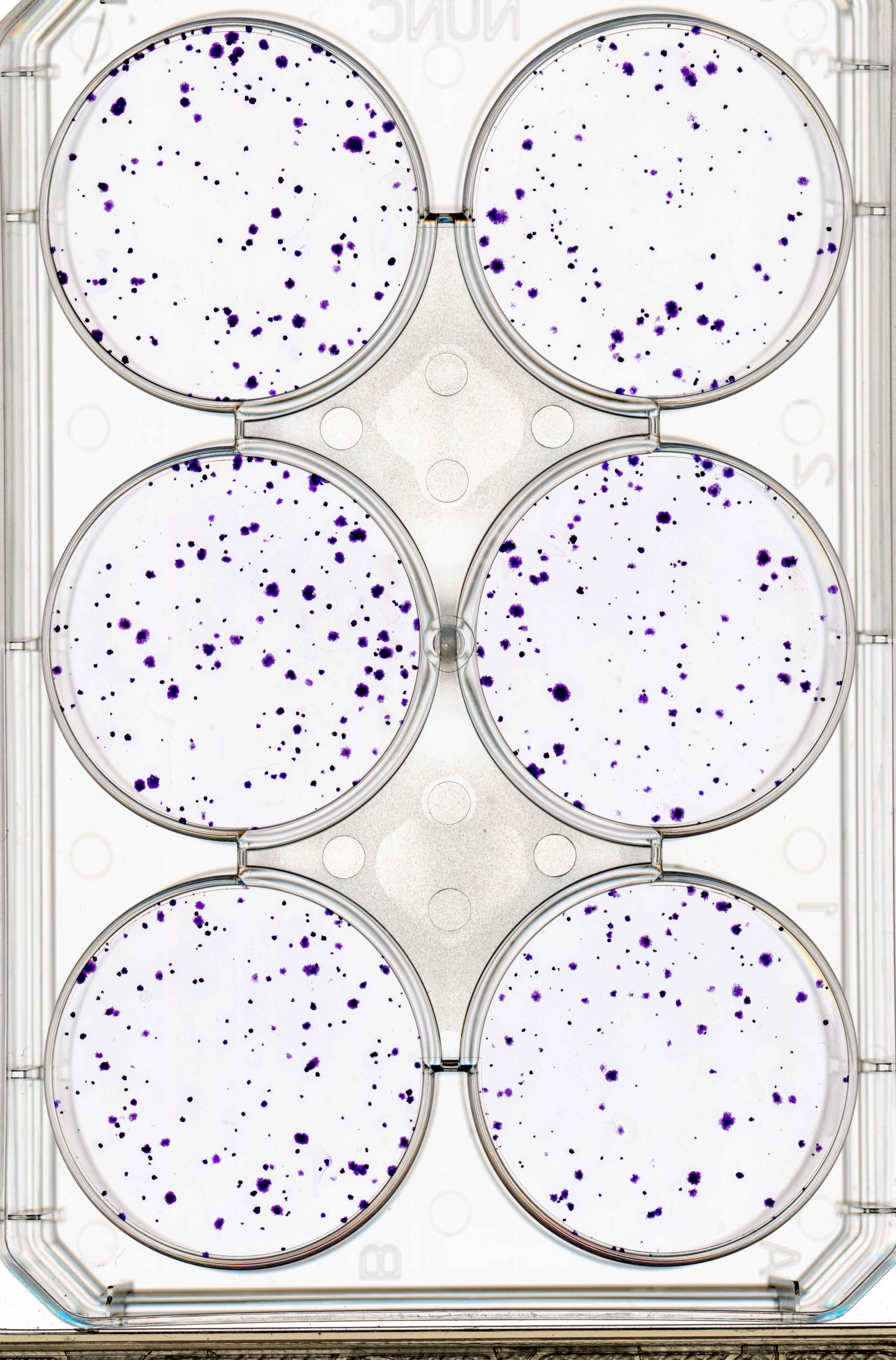

Supplement: Supplementary file 13 — Figure EV5 Source Data [file 44318_2024_108_MOESM13_ESM.zip › EMBOJ-2023-115654_FigEV5_sourcedata/EV5J/E230213 WTsiCtrl 5dC50-100.jpg]

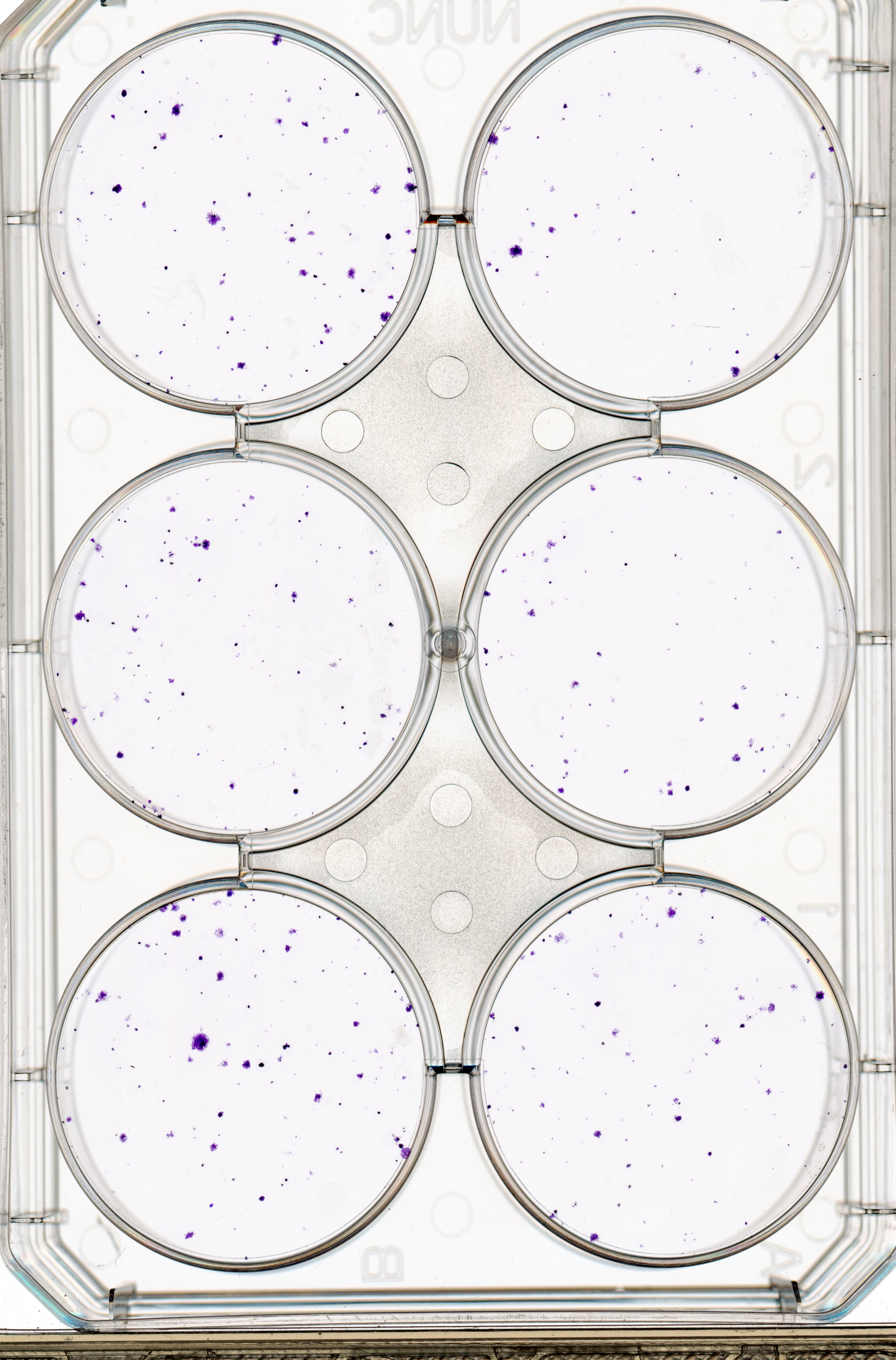

Supplement: Supplementary file 13 — Figure EV5 Source Data [file 44318_2024_108_MOESM13_ESM.zip › EMBOJ-2023-115654_FigEV5_sourcedata/EV5J/E230213 WTsiCtrl 5dC200-300.jpg]

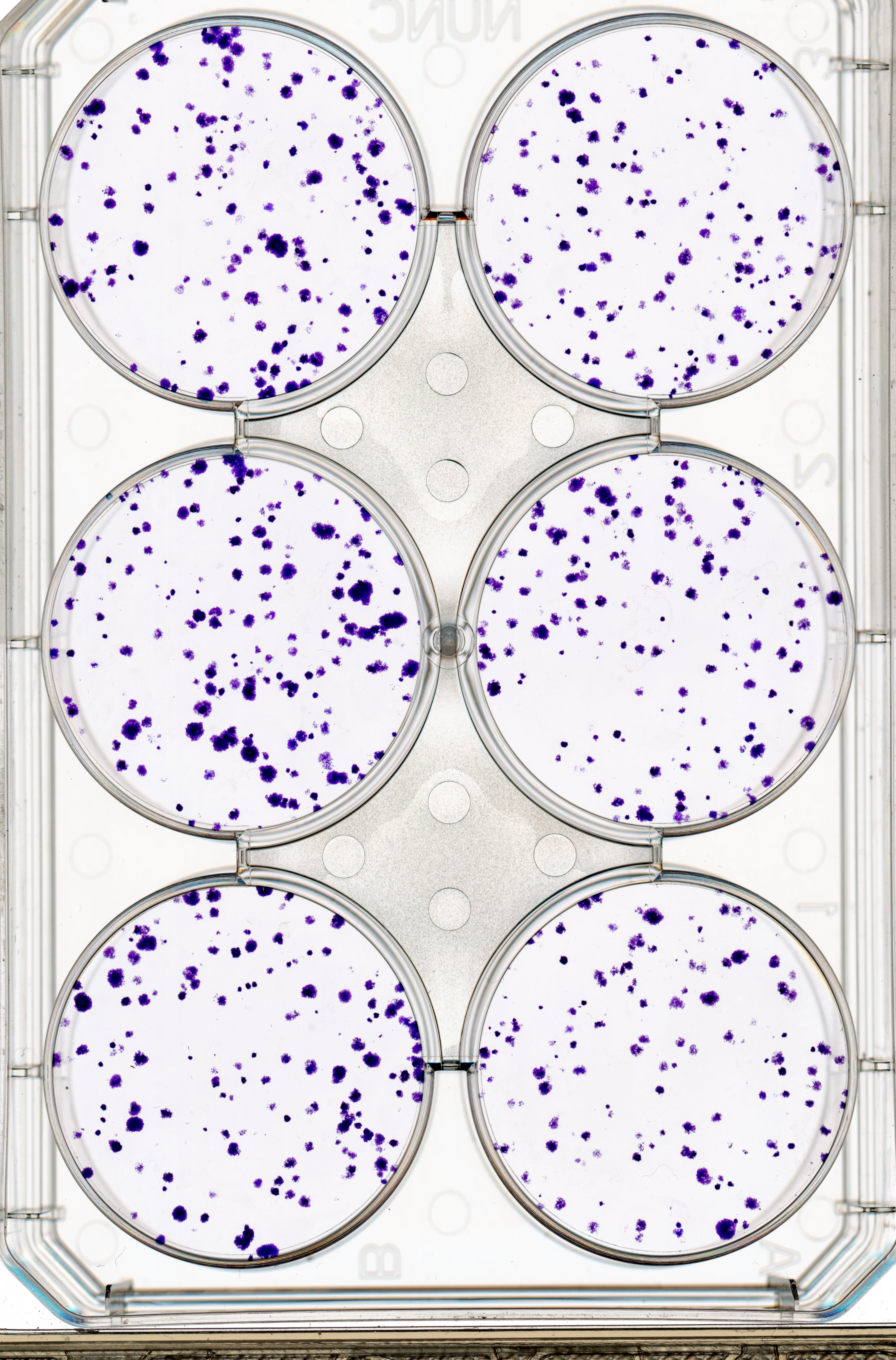

Supplement: Supplementary file 13 — Figure EV5 Source Data [file 44318_2024_108_MOESM13_ESM.zip › EMBOJ-2023-115654_FigEV5_sourcedata/EV5J/E230213 UBE2KsiCtrl 5dC10-20.jpg]

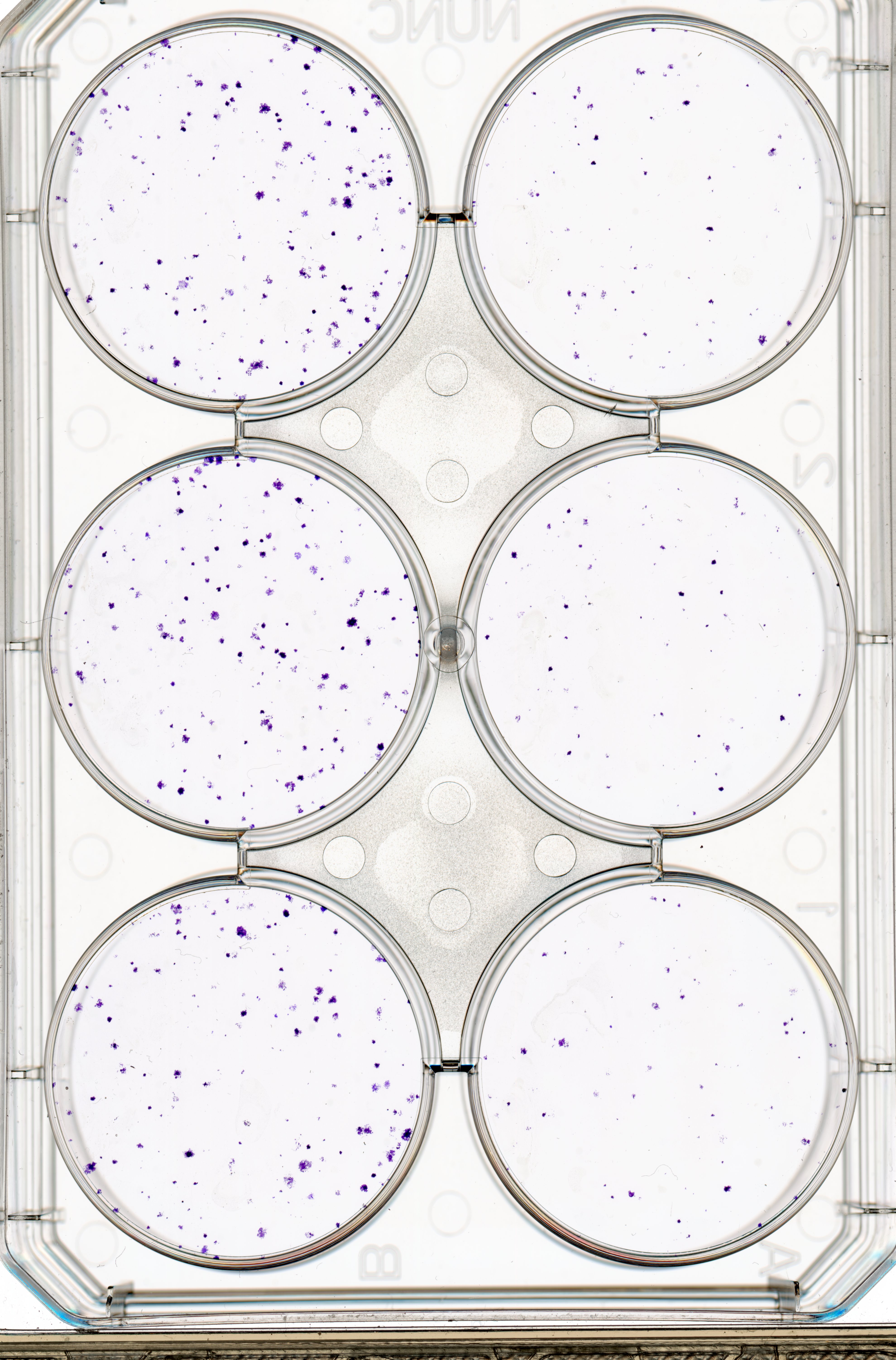

Supplement: Supplementary file 13 — Figure EV5 Source Data [file 44318_2024_108_MOESM13_ESM.zip › EMBOJ-2023-115654_FigEV5_sourcedata/EV5J/E230213 UBE2KsiRNF4 5dC50-100.jpg]

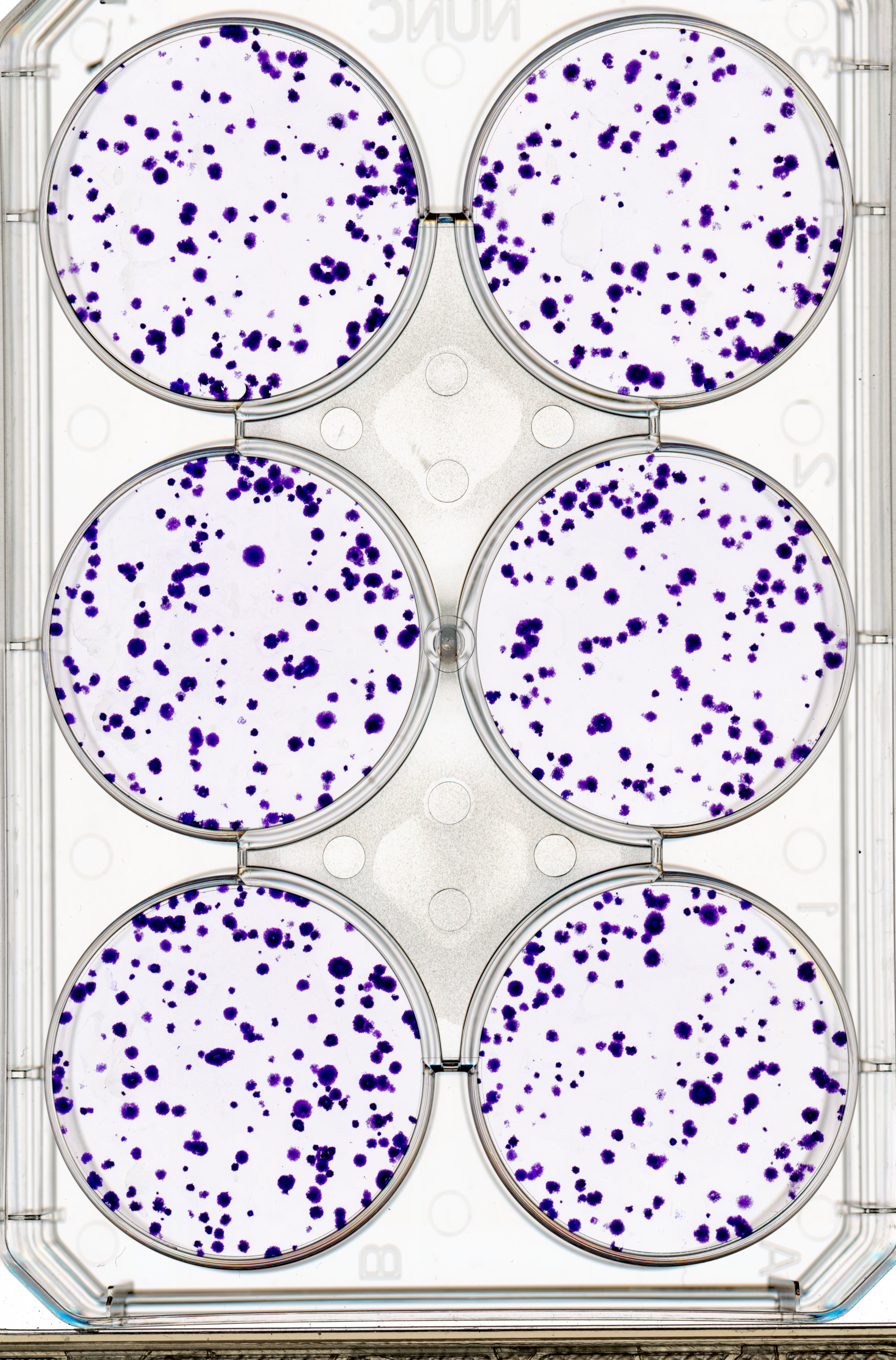

Supplement: Supplementary file 13 — Figure EV5 Source Data [file 44318_2024_108_MOESM13_ESM.zip › EMBOJ-2023-115654_FigEV5_sourcedata/EV5J/E230213 UBE2KsiCtrl 5dC0-5.jpg]

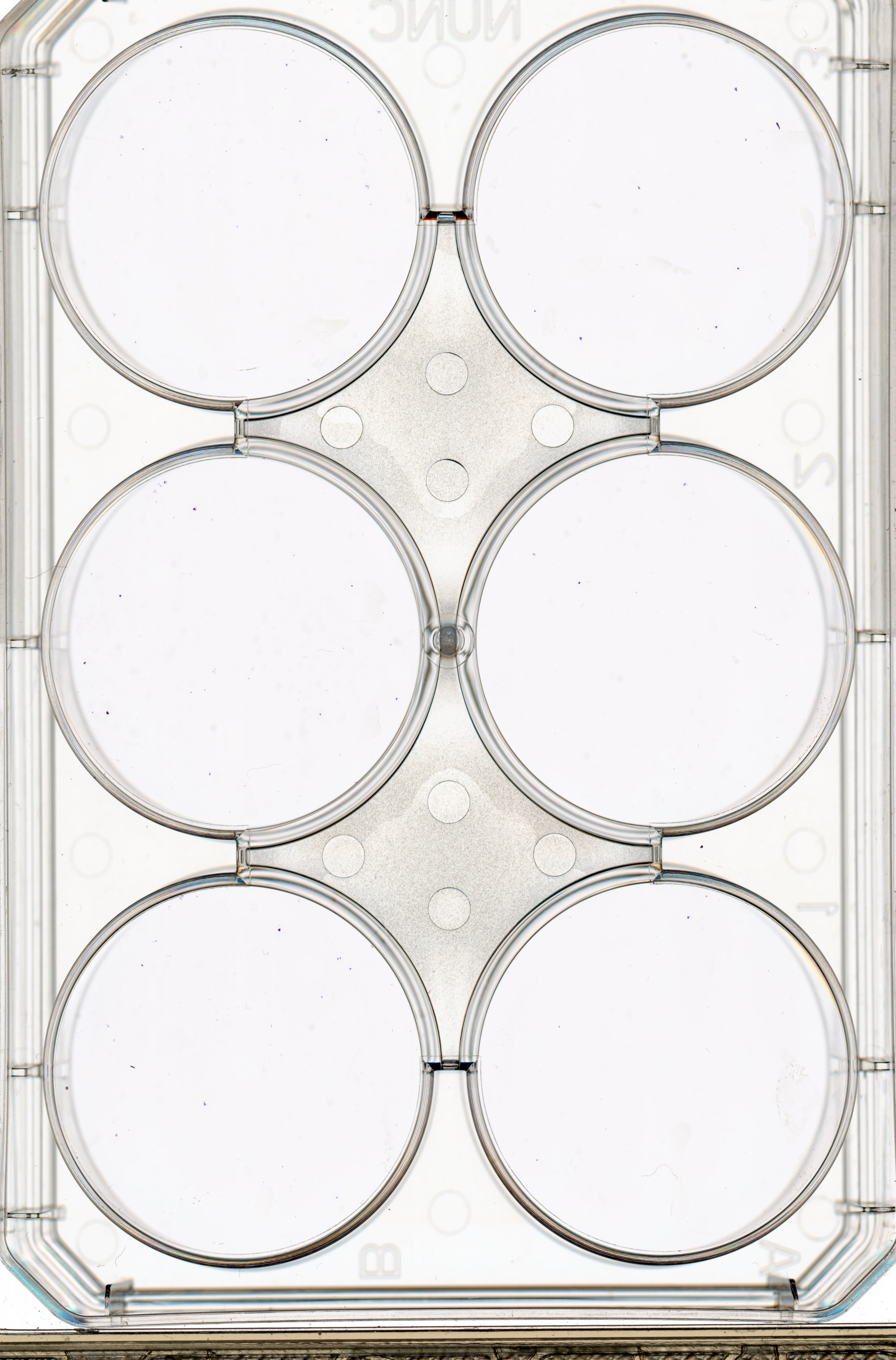

Supplement: Supplementary file 13 — Figure EV5 Source Data [file 44318_2024_108_MOESM13_ESM.zip › EMBOJ-2023-115654_FigEV5_sourcedata/EV5J/E230213 UBE2KsiRNF4 5dC200-300.jpg]

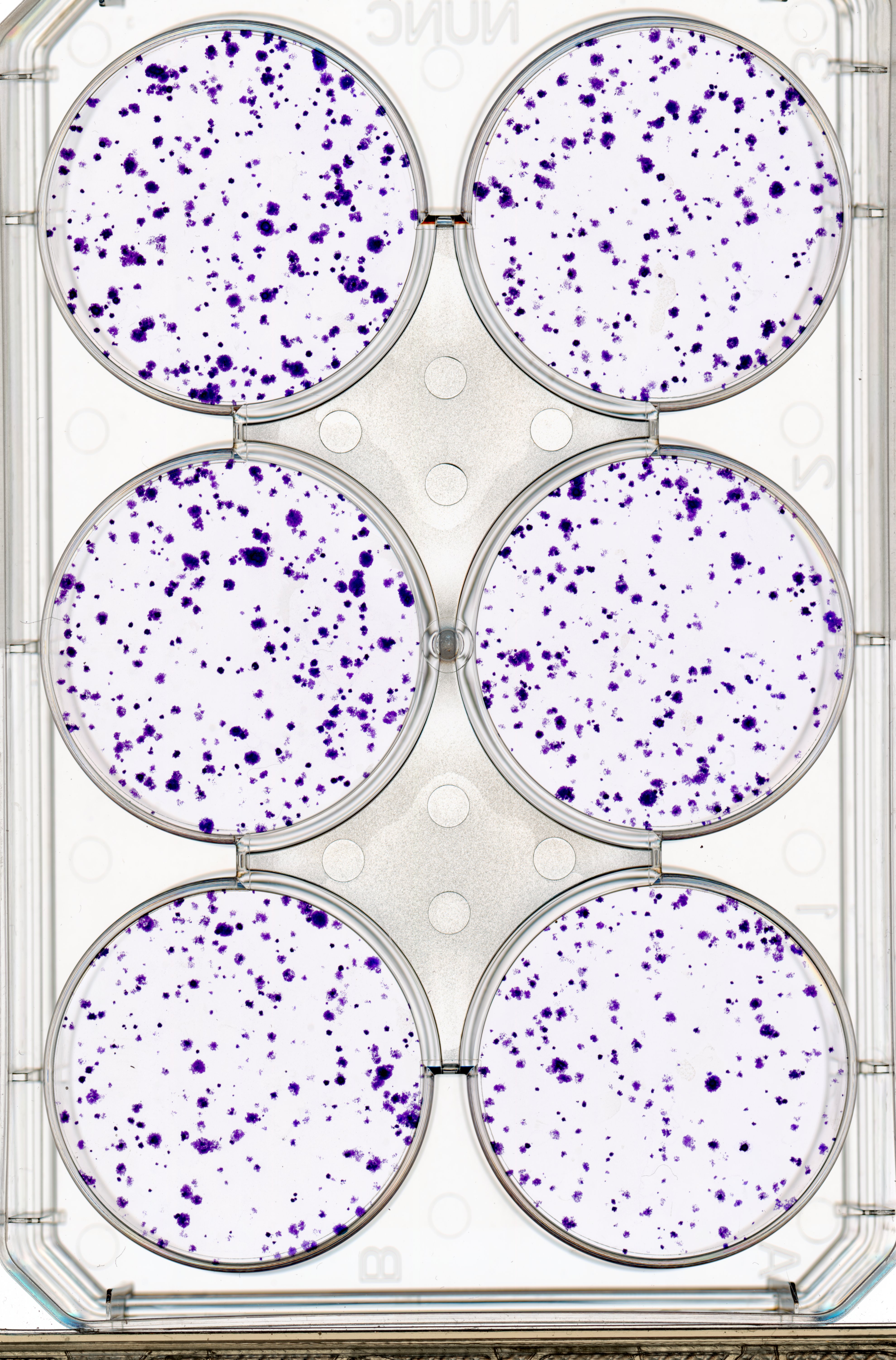

Supplement: Supplementary file 13 — Figure EV5 Source Data [file 44318_2024_108_MOESM13_ESM.zip › EMBOJ-2023-115654_FigEV5_sourcedata/EV5J/E230213 UBE2KsiRNF4 5dC10-20.jpg]

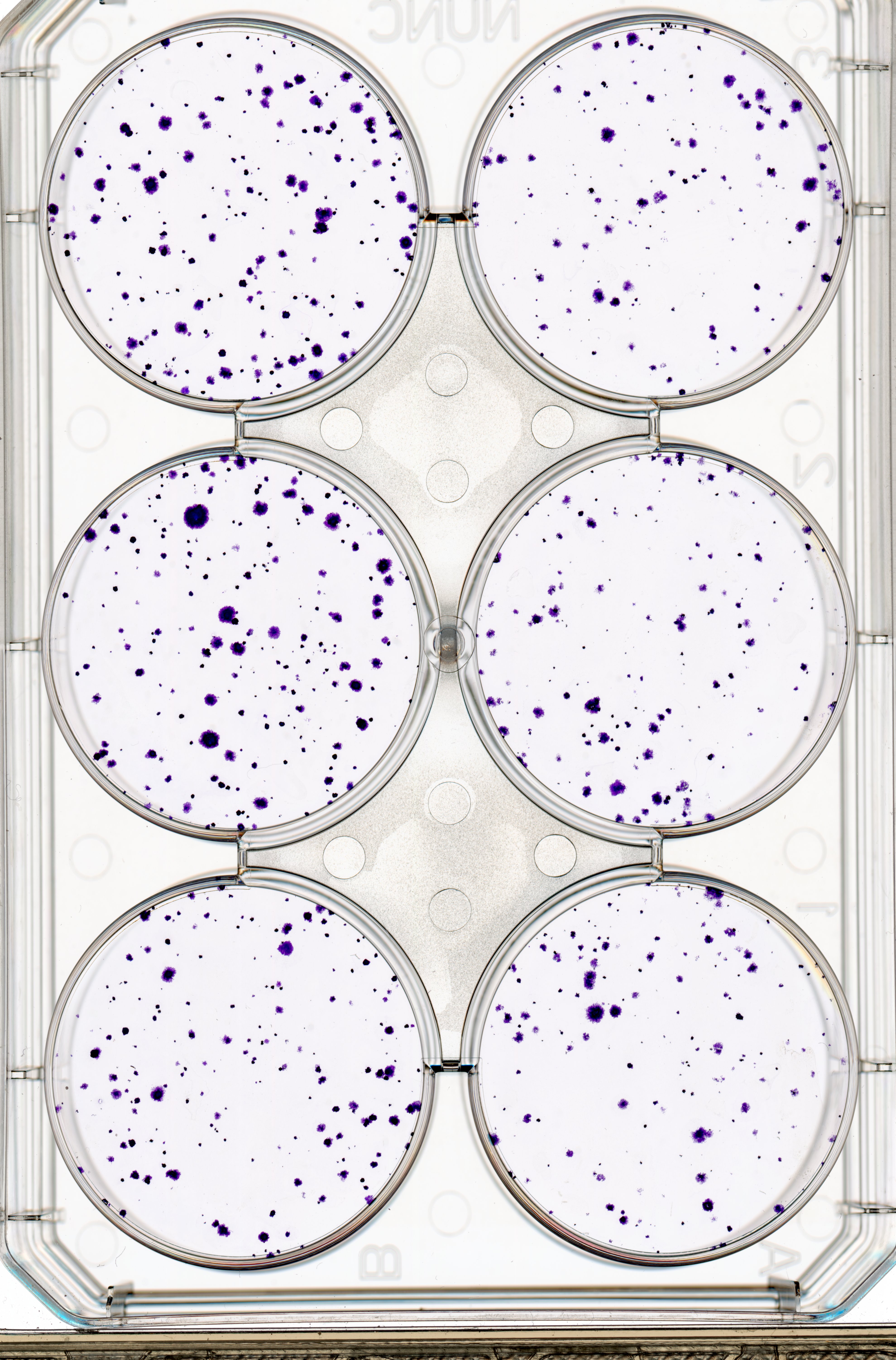

Supplement: Supplementary file 13 — Figure EV5 Source Data [file 44318_2024_108_MOESM13_ESM.zip › EMBOJ-2023-115654_FigEV5_sourcedata/EV5J/E230213 WTsiRNF4 5dC50-100.jpg]

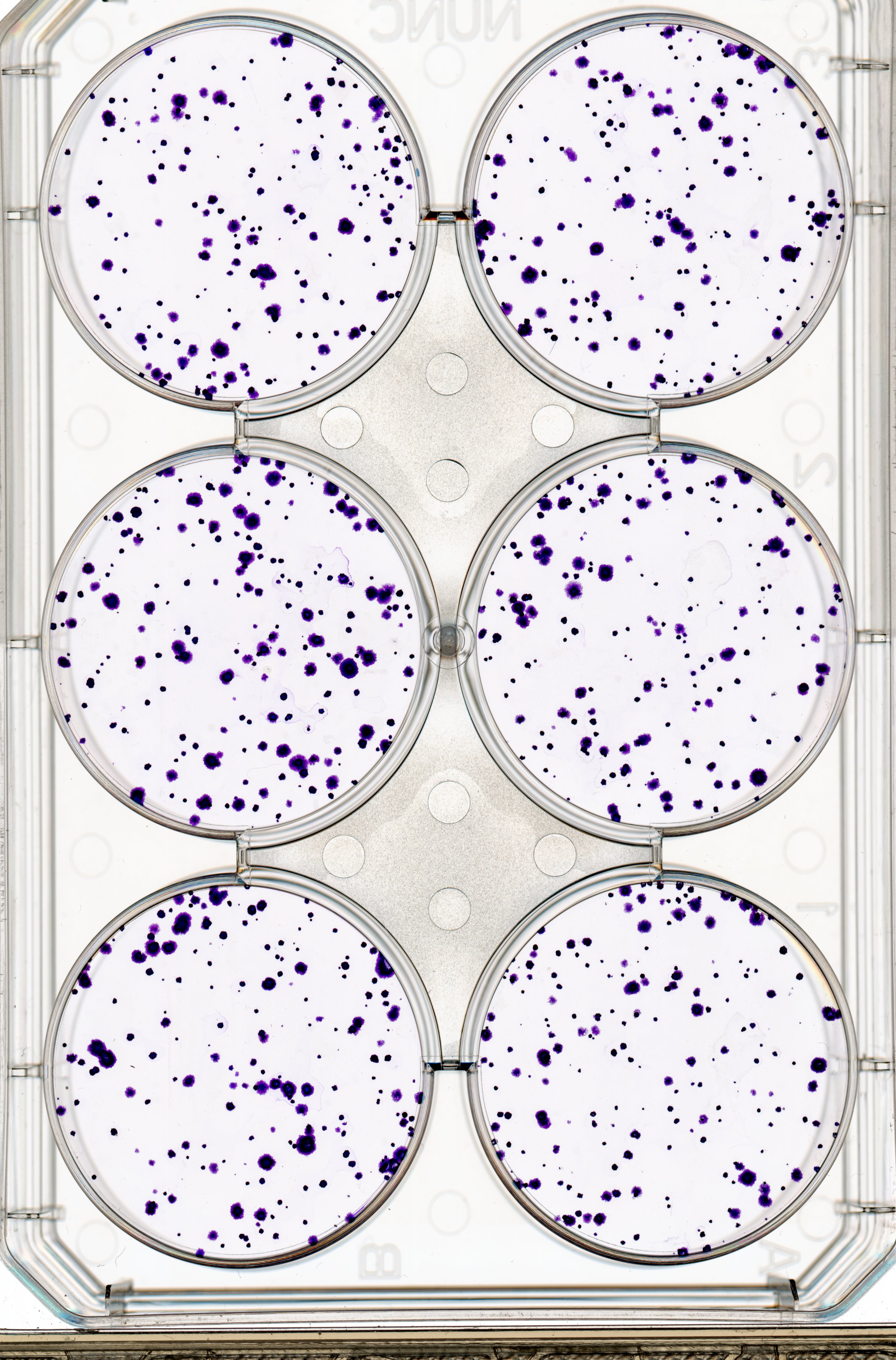

Supplement: Supplementary file 13 — Figure EV5 Source Data [file 44318_2024_108_MOESM13_ESM.zip › EMBOJ-2023-115654_FigEV5_sourcedata/EV5J/E230213 WTsiCtrl 5dC10-20.jpg]

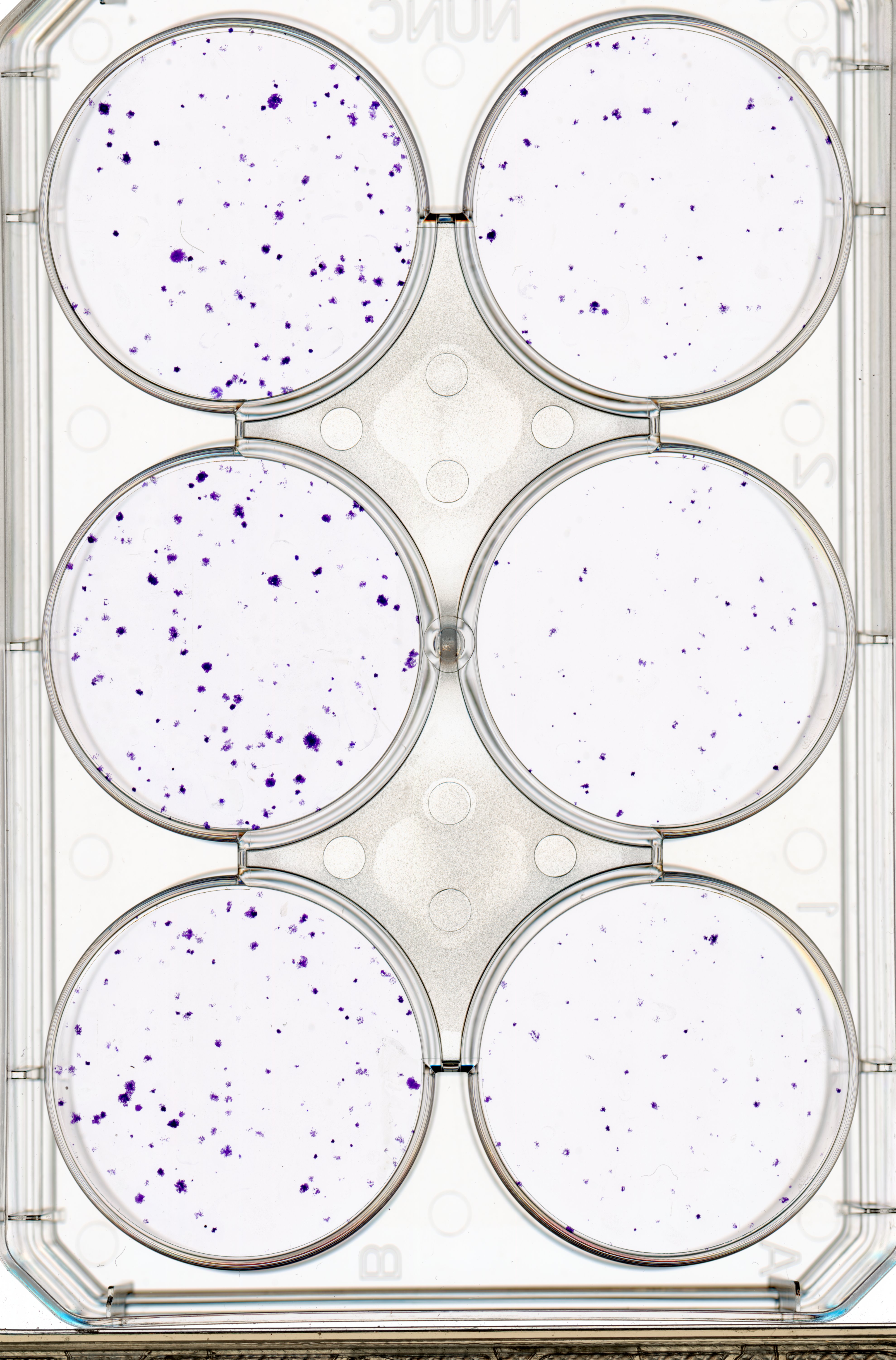

Supplement: Supplementary file 13 — Figure EV5 Source Data [file 44318_2024_108_MOESM13_ESM.zip › EMBOJ-2023-115654_FigEV5_sourcedata/EV5J/E230213 UBE2KsiCtrl 5dC50-100.jpg]

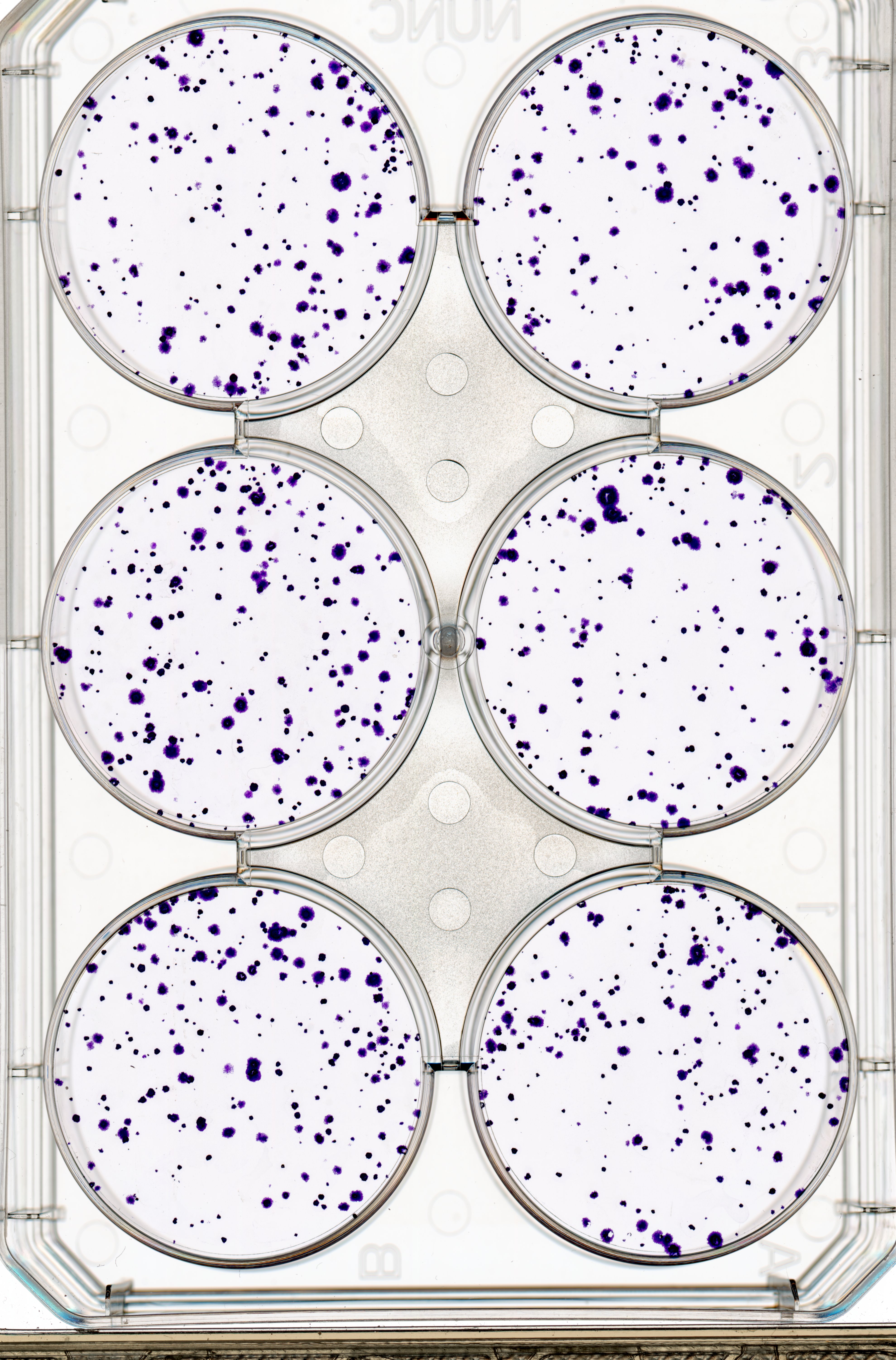

Supplement: Supplementary file 13 — Figure EV5 Source Data [file 44318_2024_108_MOESM13_ESM.zip › EMBOJ-2023-115654_FigEV5_sourcedata/EV5J/E230213 WTsiRNF4 5dC10-20.jpg]

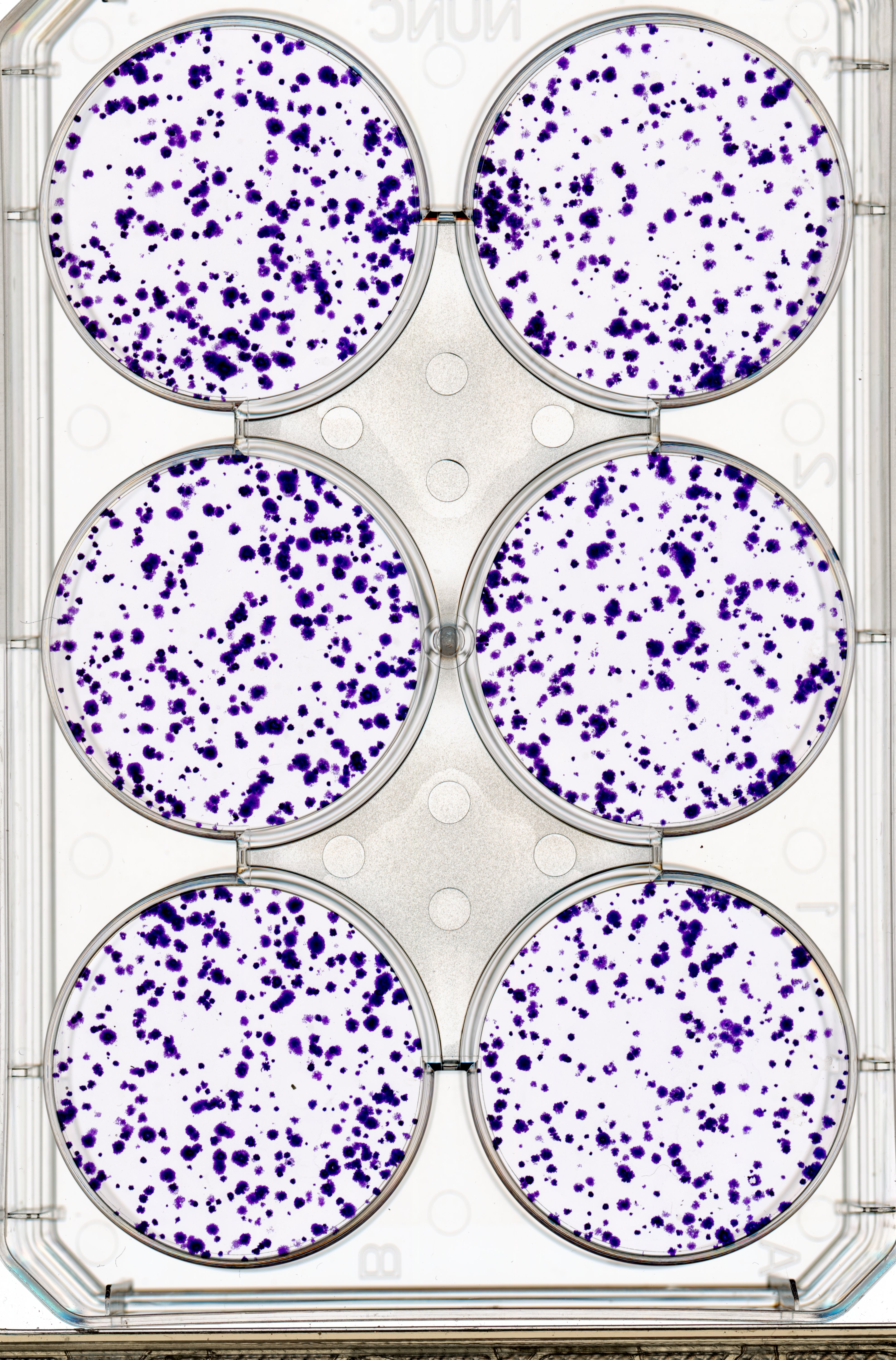

Supplement: Supplementary file 13 — Figure EV5 Source Data [file 44318_2024_108_MOESM13_ESM.zip › EMBOJ-2023-115654_FigEV5_sourcedata/EV5J/E230213 UBE2KsiRNF4 5dC0-5.jpg]

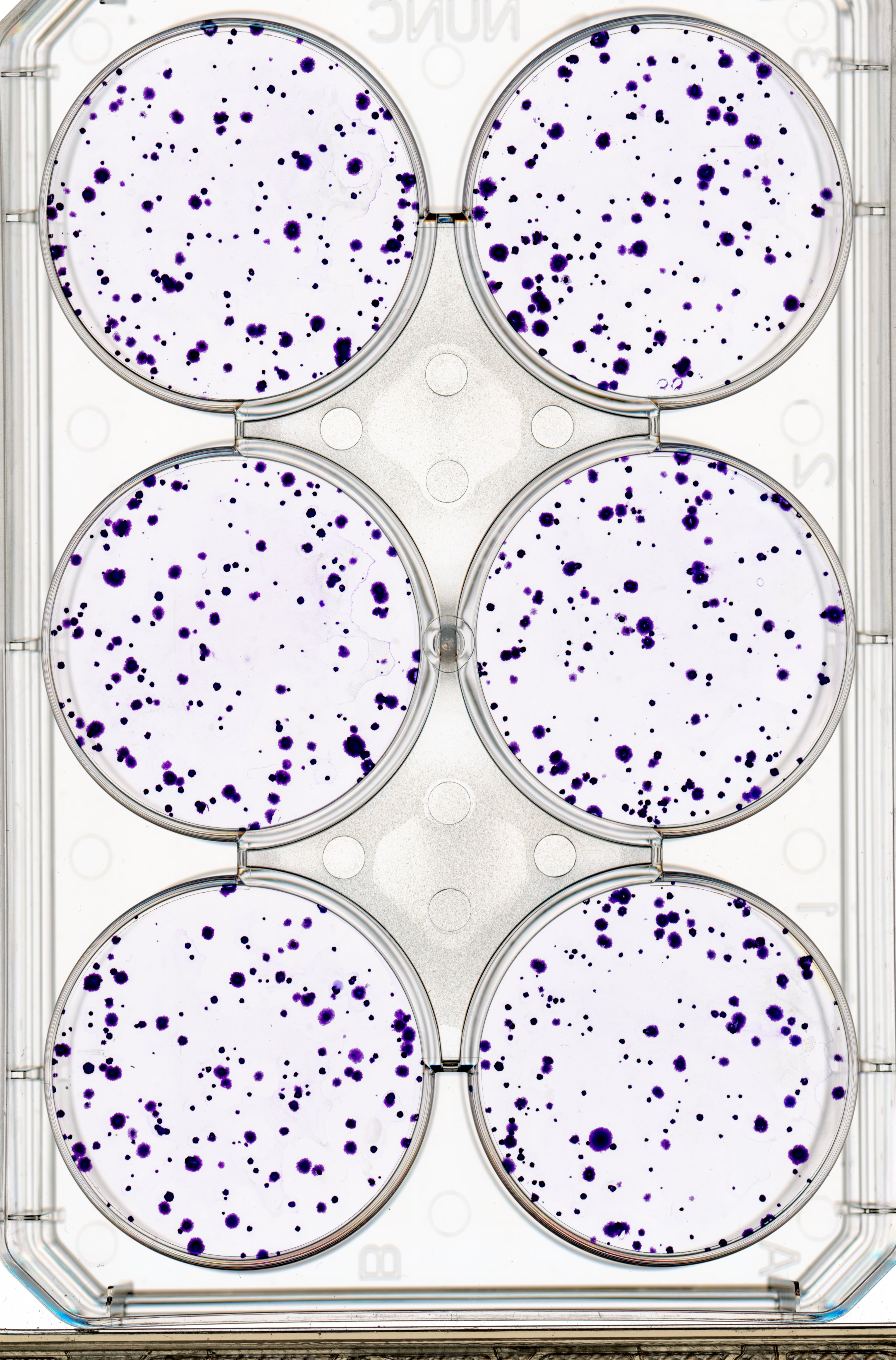

Supplement: Supplementary file 13 — Figure EV5 Source Data [file 44318_2024_108_MOESM13_ESM.zip › EMBOJ-2023-115654_FigEV5_sourcedata/EV5J/E230213 WTsiCtrl 5dC0-5.jpg]

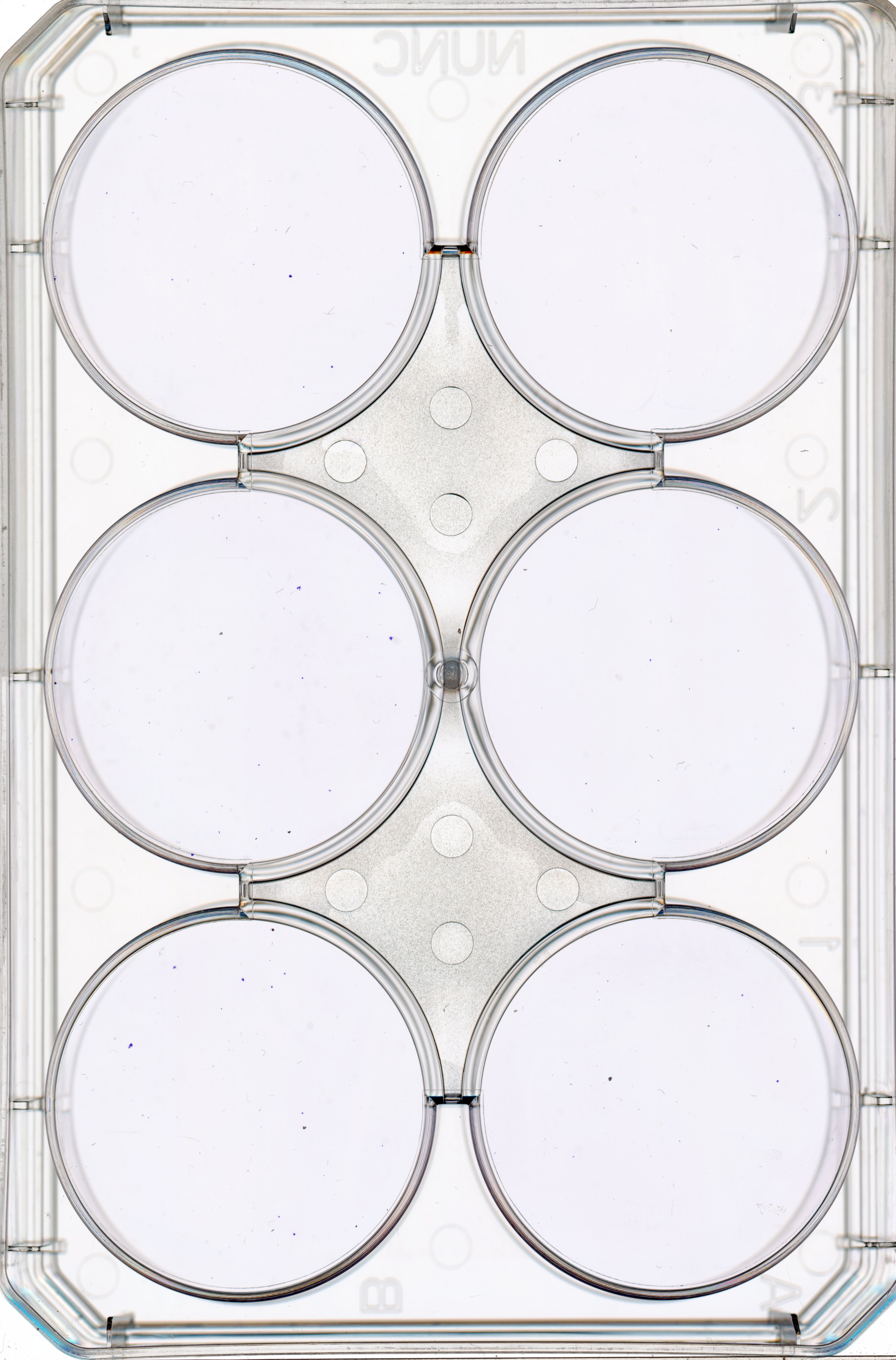

Supplement: Supplementary file 13 — Figure EV5 Source Data [file 44318_2024_108_MOESM13_ESM.zip › EMBOJ-2023-115654_FigEV5_sourcedata/EV5G/E230217 U2KtrsDKO1cl 5dC50-100.jpg]

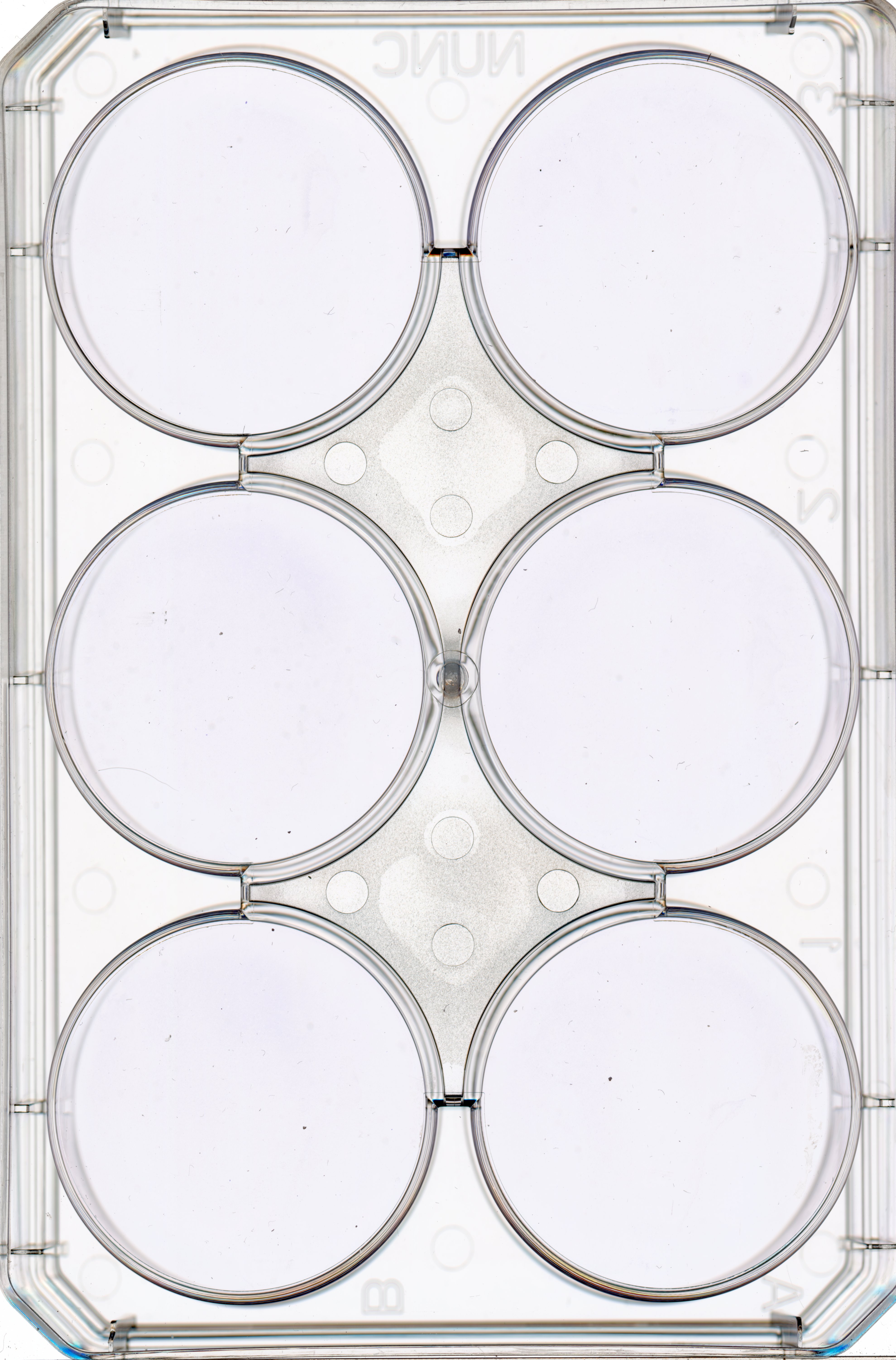

Supplement: Supplementary file 13 — Figure EV5 Source Data [file 44318_2024_108_MOESM13_ESM.zip › EMBOJ-2023-115654_FigEV5_sourcedata/EV5G/E230217 U2KtrsDKO1cl 5dC200-300.jpg]

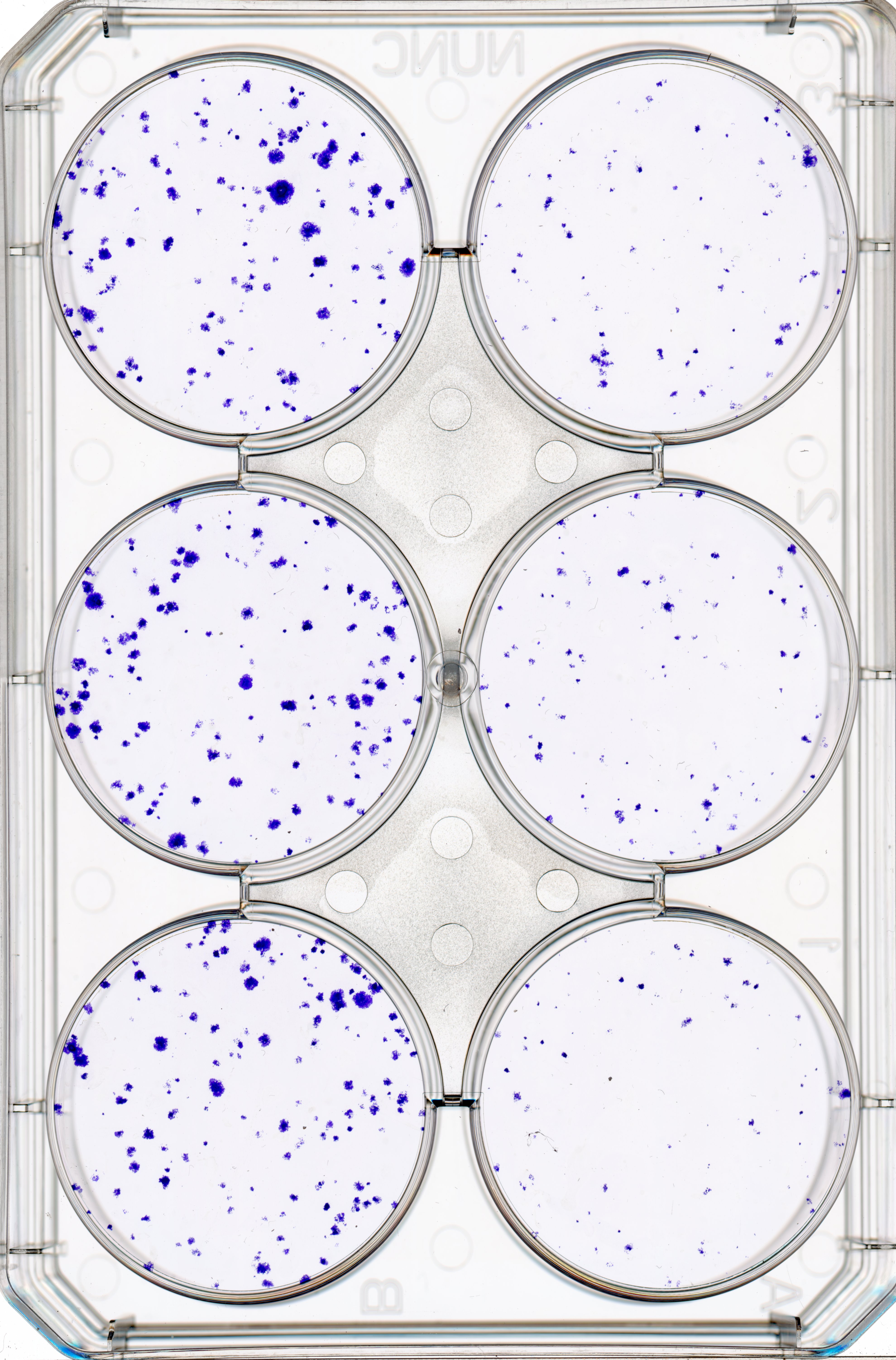

Supplement: Supplementary file 13 — Figure EV5 Source Data [file 44318_2024_108_MOESM13_ESM.zip › EMBOJ-2023-115654_FigEV5_sourcedata/EV5G/E230217 UBE2K 5dC50-100.jpg]

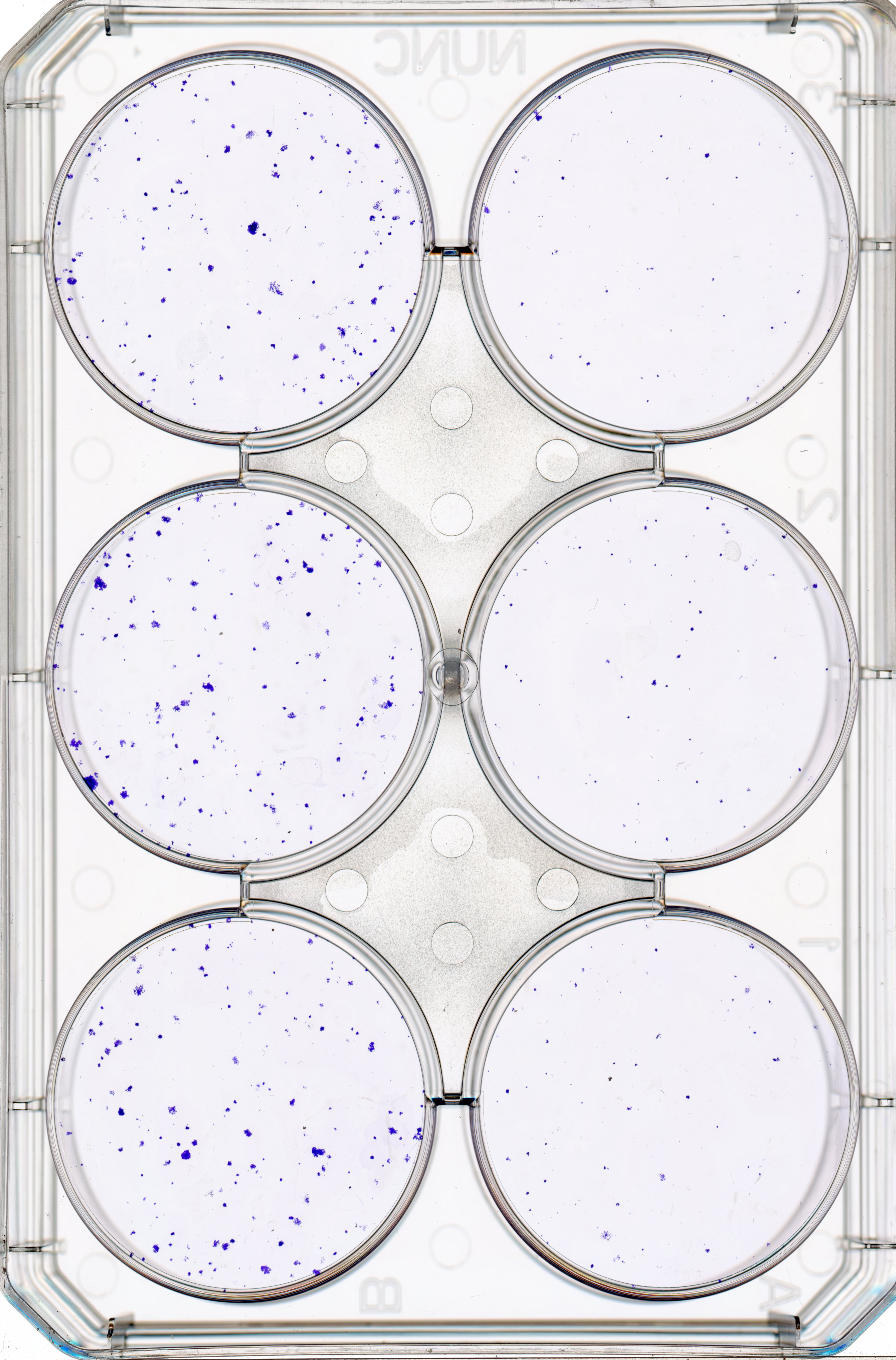

Supplement: Supplementary file 13 — Figure EV5 Source Data [file 44318_2024_108_MOESM13_ESM.zip › EMBOJ-2023-115654_FigEV5_sourcedata/EV5G/E230217 U2KtrsDKO1cl 5dC10-20.jpg]

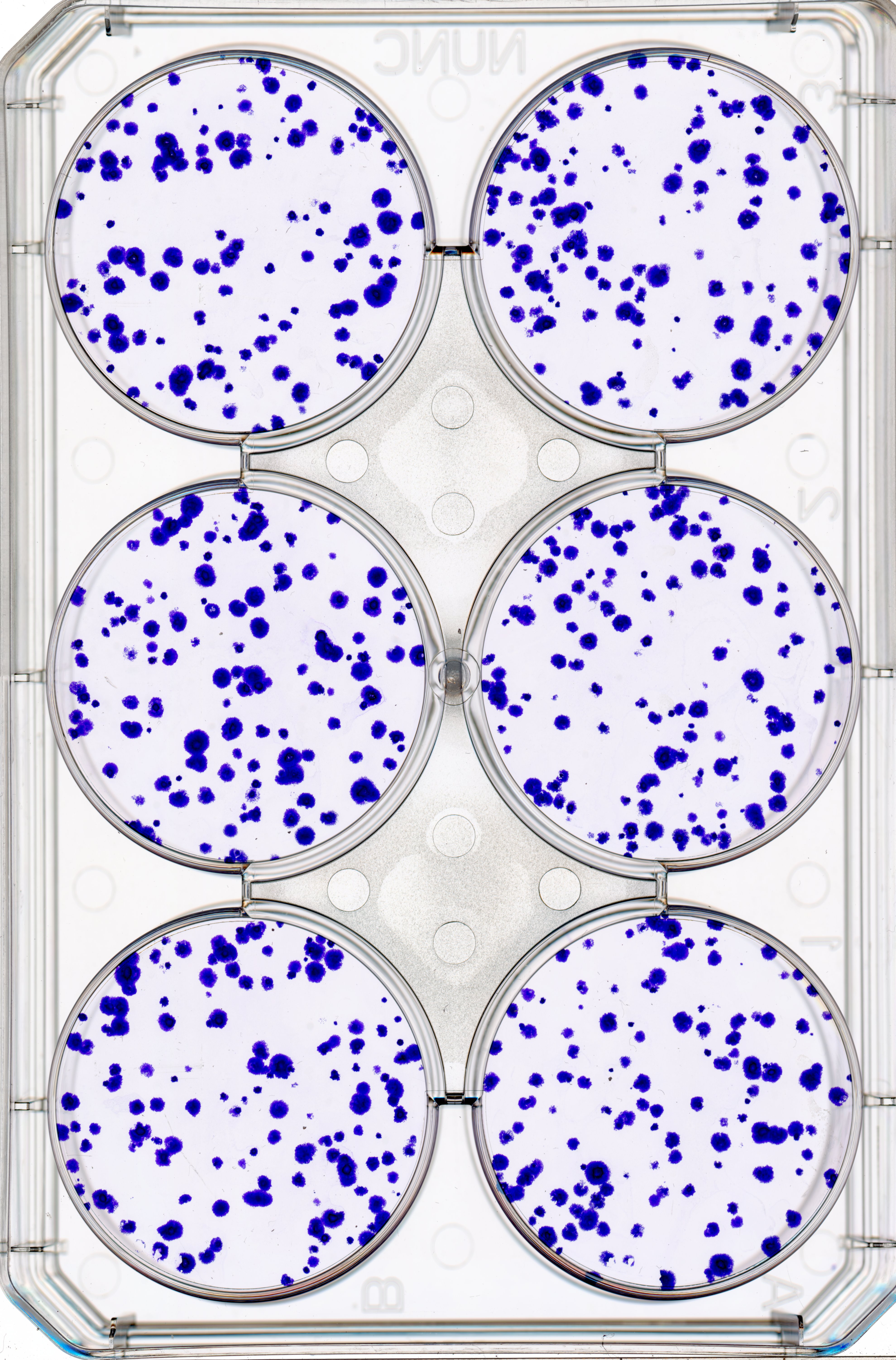

Supplement: Supplementary file 13 — Figure EV5 Source Data [file 44318_2024_108_MOESM13_ESM.zip › EMBOJ-2023-115654_FigEV5_sourcedata/EV5G/E230217 UBE2K 5dC0-5.jpg]

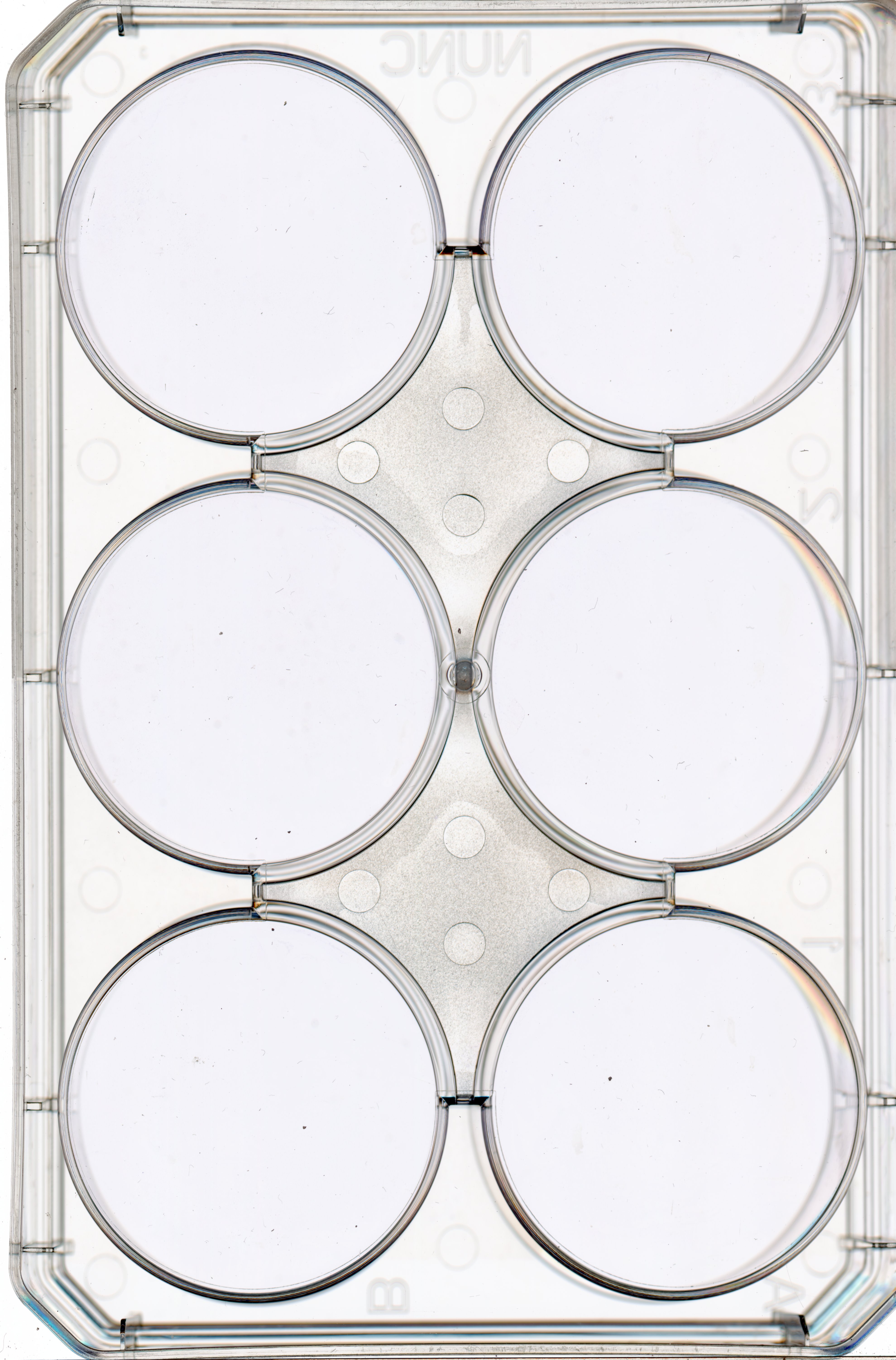

Supplement: Supplementary file 13 — Figure EV5 Source Data [file 44318_2024_108_MOESM13_ESM.zip › EMBOJ-2023-115654_FigEV5_sourcedata/EV5G/E230217 U2KtrsDKOcl10 5dC200-300.jpg]

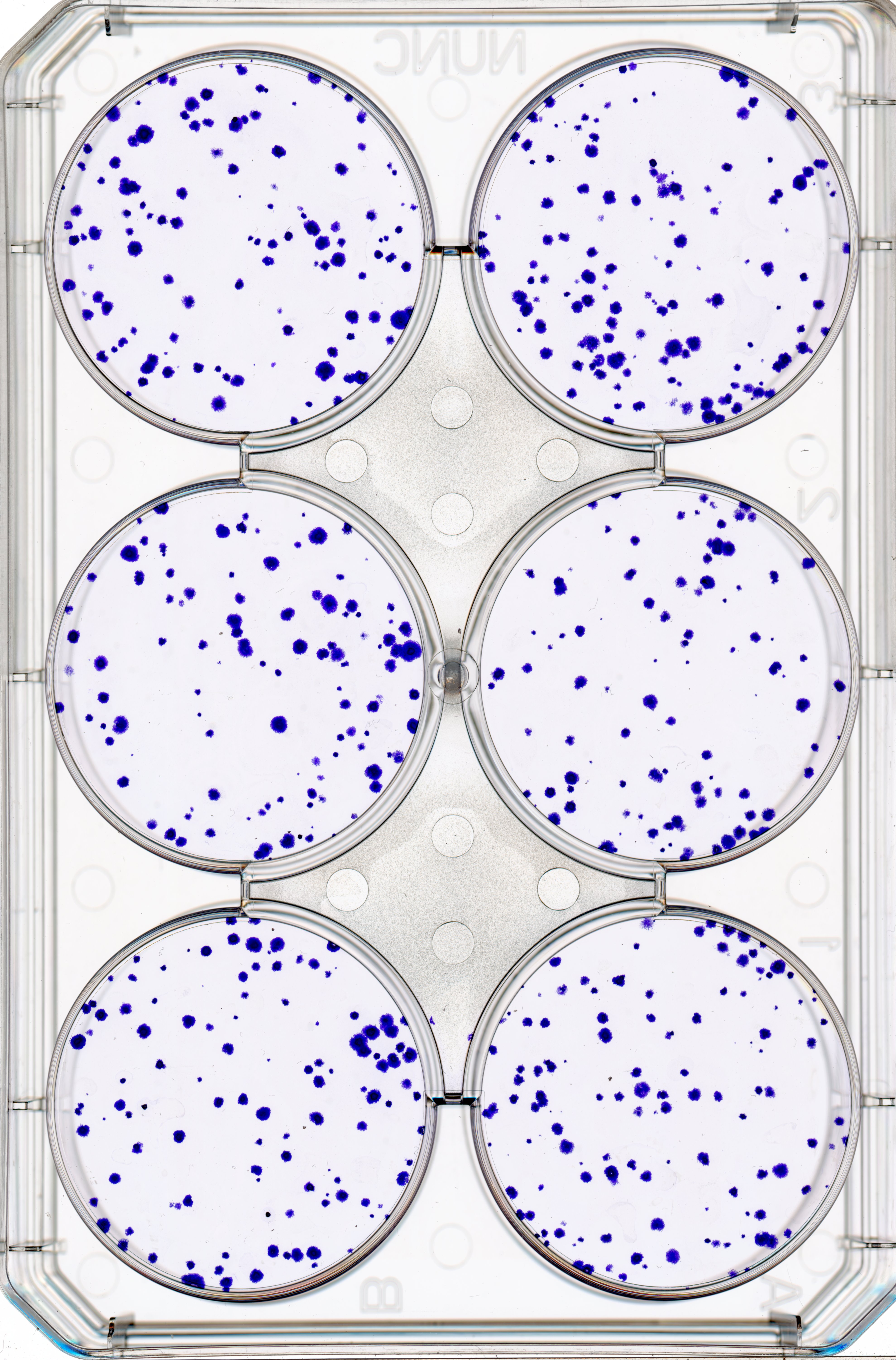

Supplement: Supplementary file 13 — Figure EV5 Source Data [file 44318_2024_108_MOESM13_ESM.zip › EMBOJ-2023-115654_FigEV5_sourcedata/EV5G/E230217 TOPORS 5dC0-5.jpg]

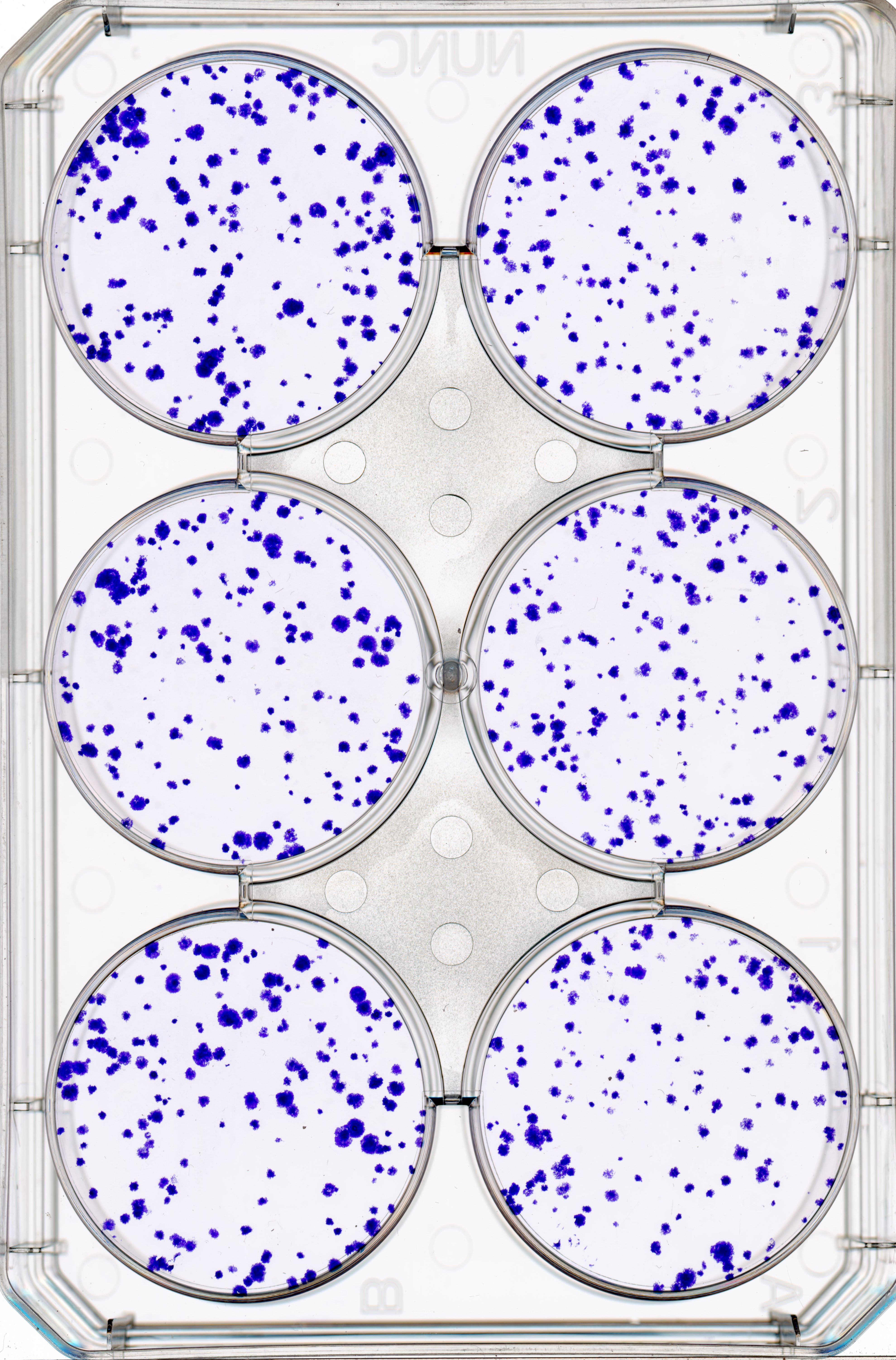

Supplement: Supplementary file 13 — Figure EV5 Source Data [file 44318_2024_108_MOESM13_ESM.zip › EMBOJ-2023-115654_FigEV5_sourcedata/EV5G/E230217 U2KtrsDKOcl10 5dC0-5.jpg]

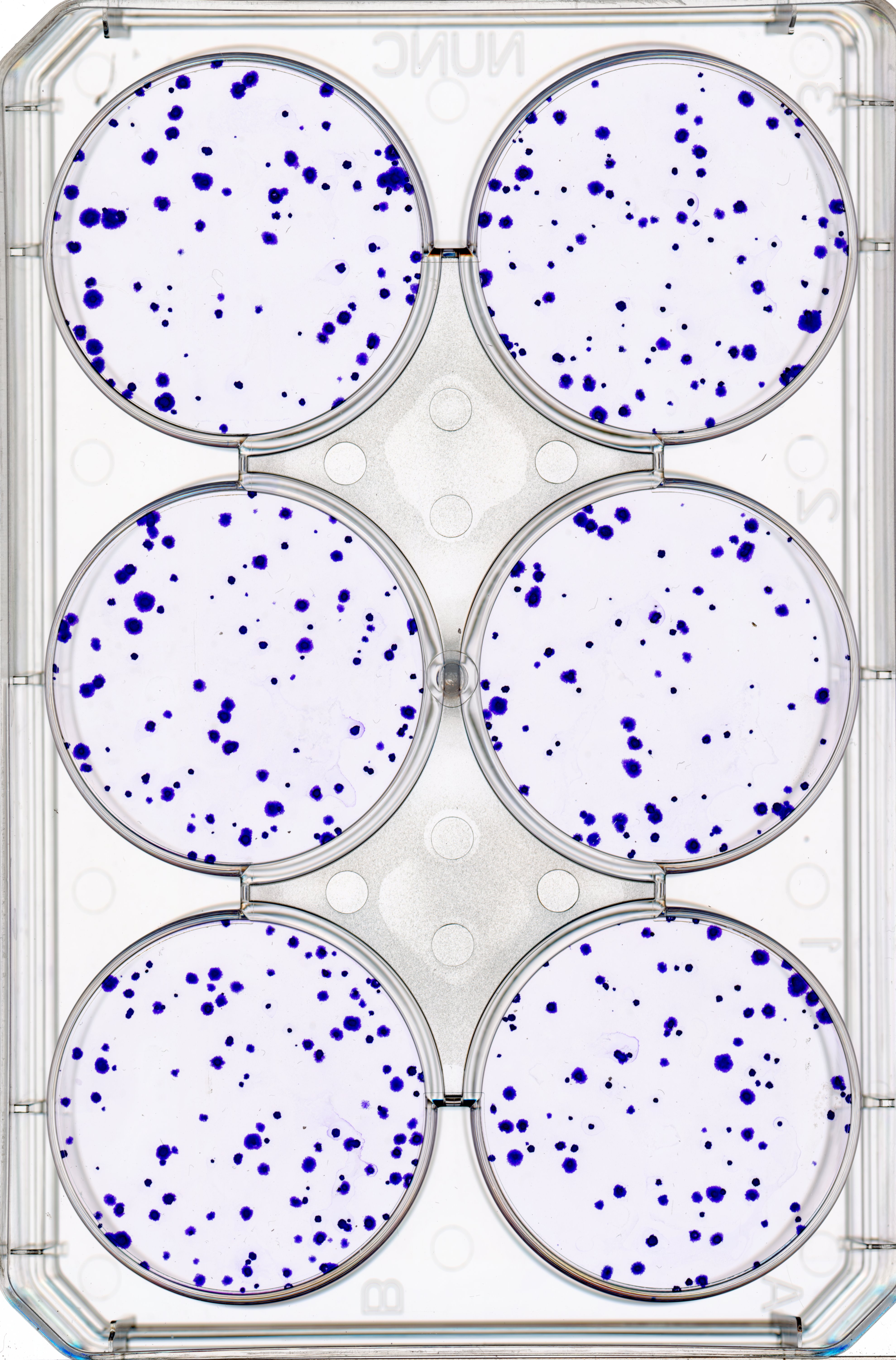

Supplement: Supplementary file 13 — Figure EV5 Source Data [file 44318_2024_108_MOESM13_ESM.zip › EMBOJ-2023-115654_FigEV5_sourcedata/EV5G/E230217 WT 5dC10-20.jpg]

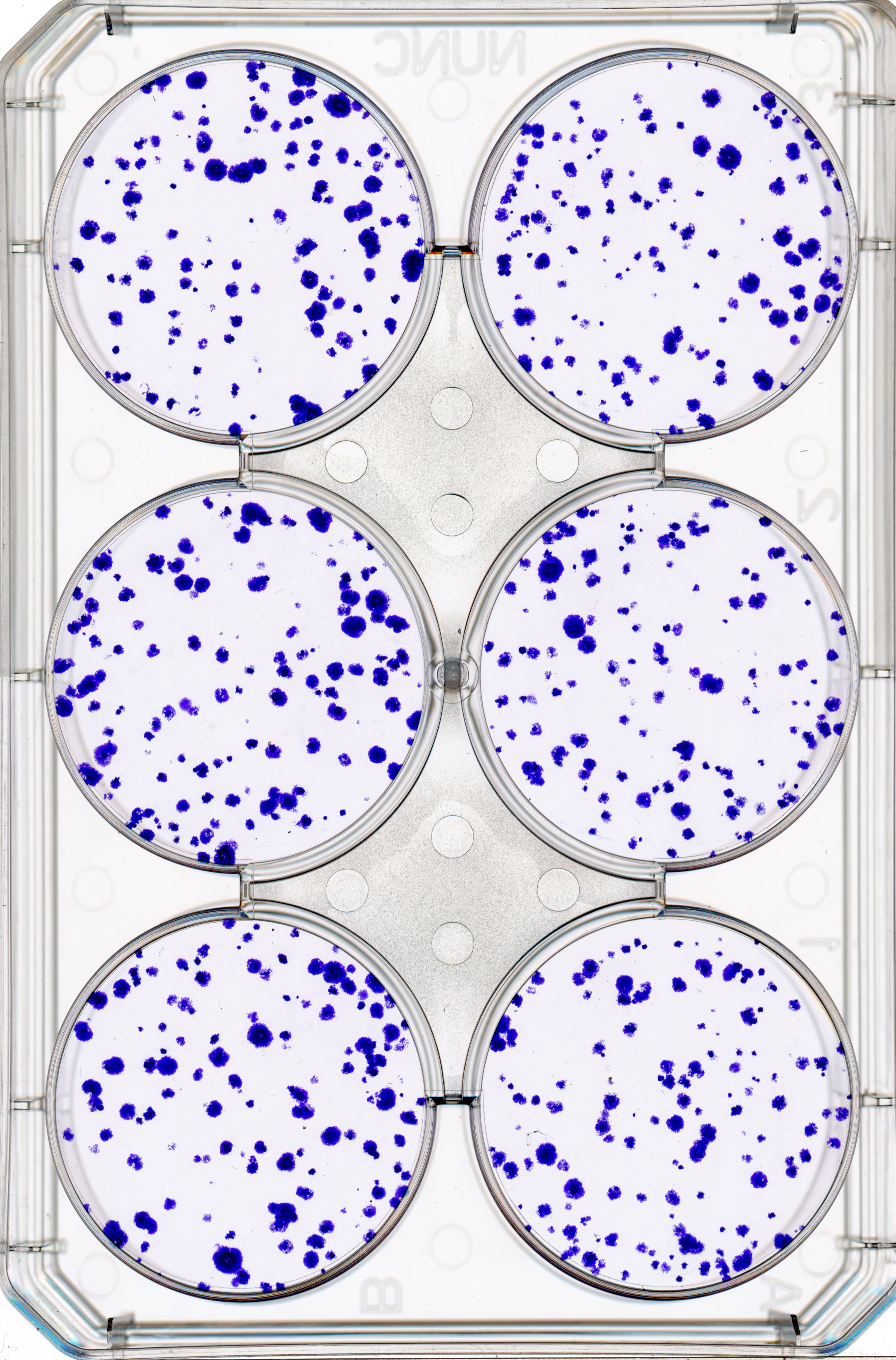

Supplement: Supplementary file 13 — Figure EV5 Source Data [file 44318_2024_108_MOESM13_ESM.zip › EMBOJ-2023-115654_FigEV5_sourcedata/EV5G/E230217 UBE2K 5dC10-20.jpg]

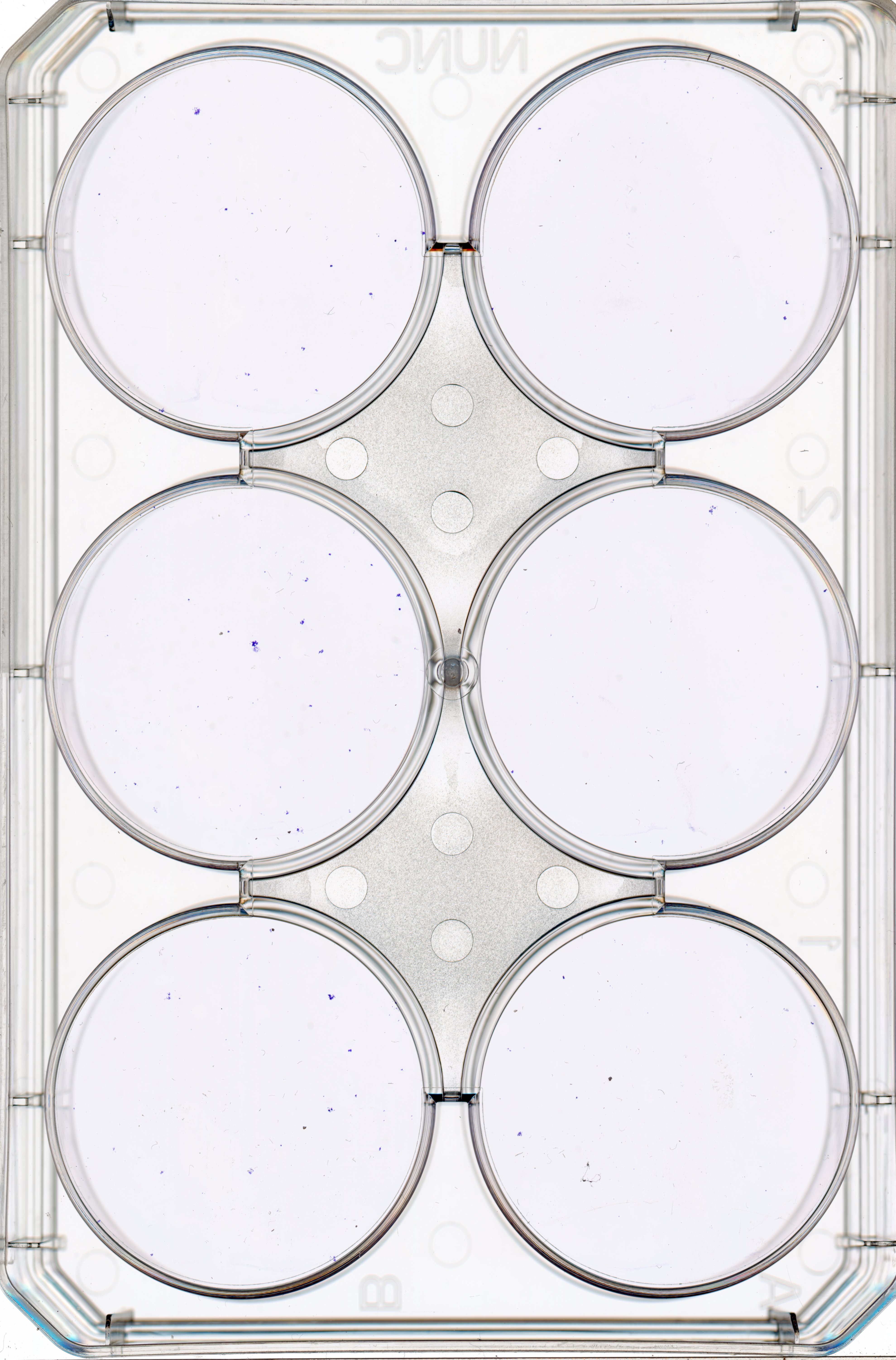

Supplement: Supplementary file 13 — Figure EV5 Source Data [file 44318_2024_108_MOESM13_ESM.zip › EMBOJ-2023-115654_FigEV5_sourcedata/EV5G/E230217 UBE2K 5dC200-300.jpg]

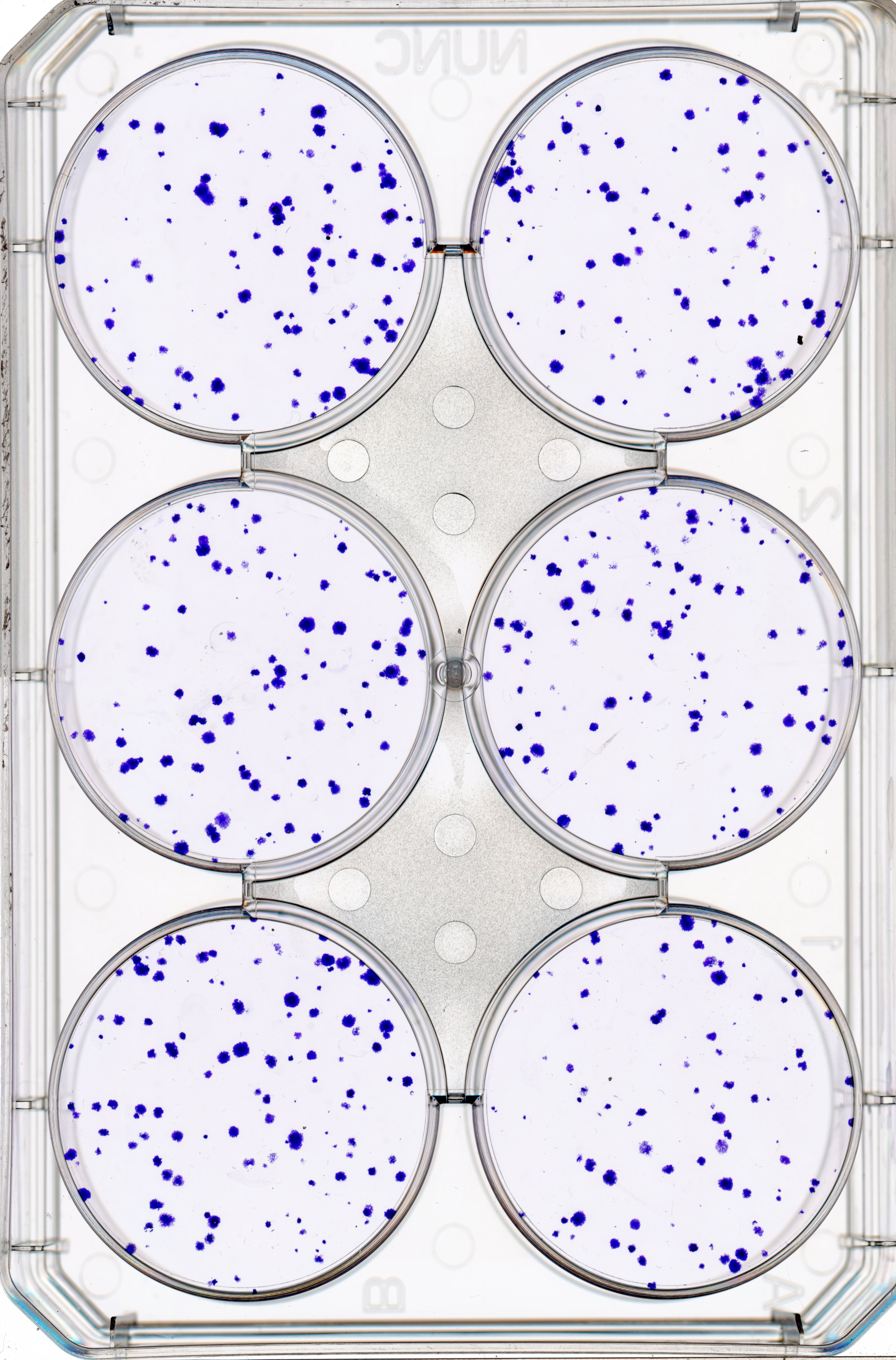

Supplement: Supplementary file 13 — Figure EV5 Source Data [file 44318_2024_108_MOESM13_ESM.zip › EMBOJ-2023-115654_FigEV5_sourcedata/EV5G/E230217 TOPORS 5dC10-20.jpg]

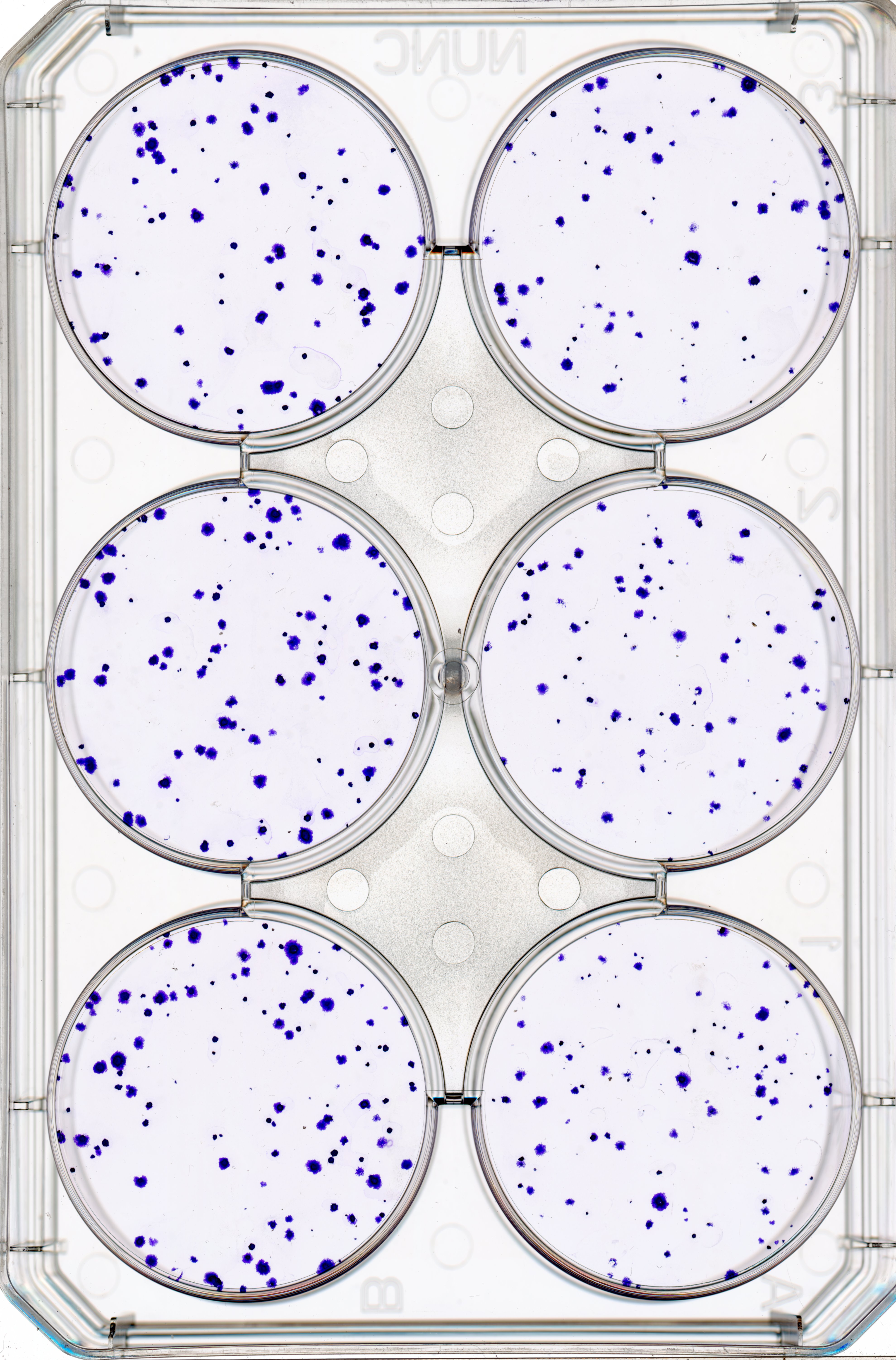

Supplement: Supplementary file 13 — Figure EV5 Source Data [file 44318_2024_108_MOESM13_ESM.zip › EMBOJ-2023-115654_FigEV5_sourcedata/EV5G/E230217 WT 5dC50-100.jpg]

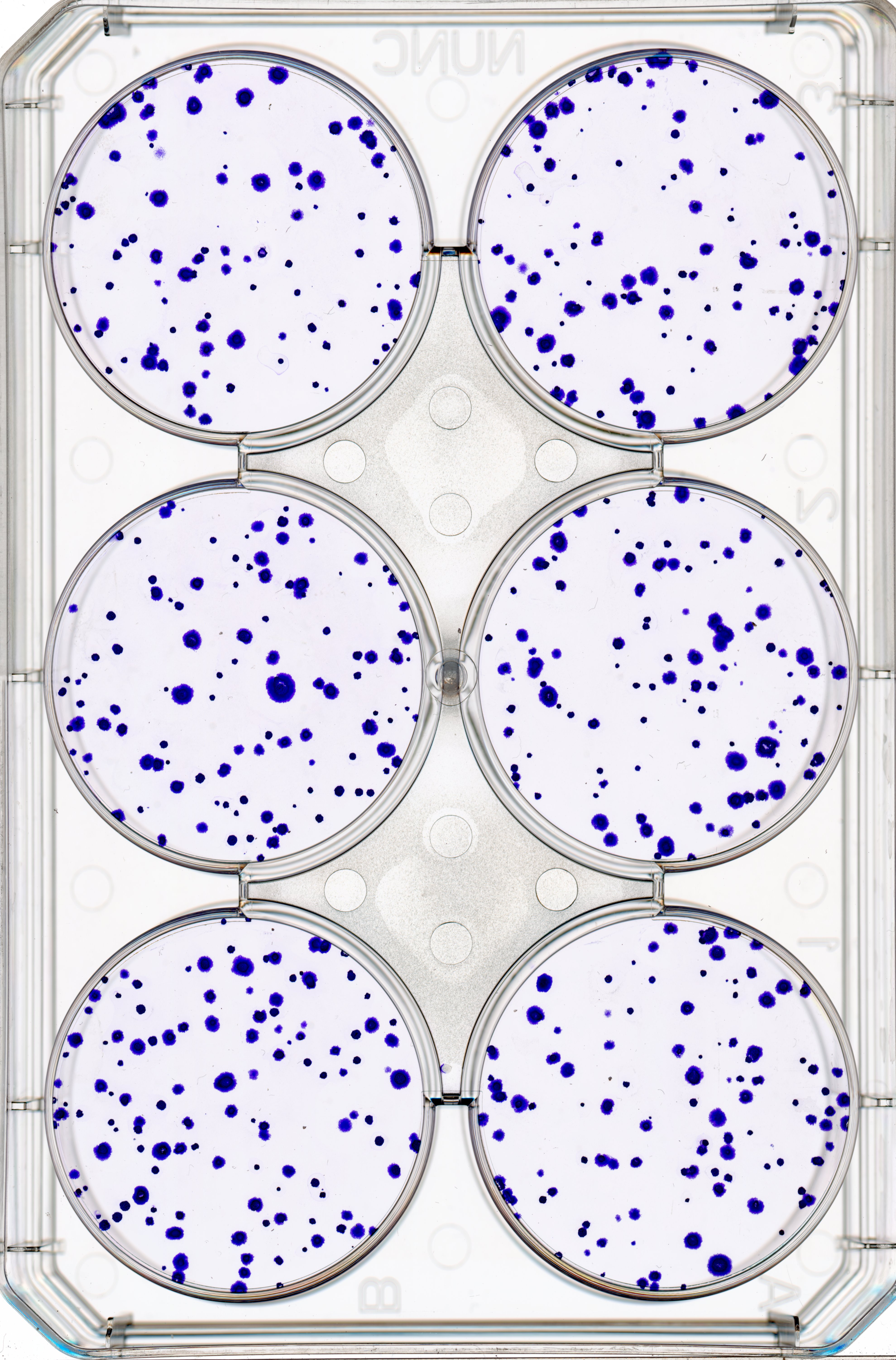

Supplement: Supplementary file 13 — Figure EV5 Source Data [file 44318_2024_108_MOESM13_ESM.zip › EMBOJ-2023-115654_FigEV5_sourcedata/EV5G/E230217 WT 5dC0-5.jpg]

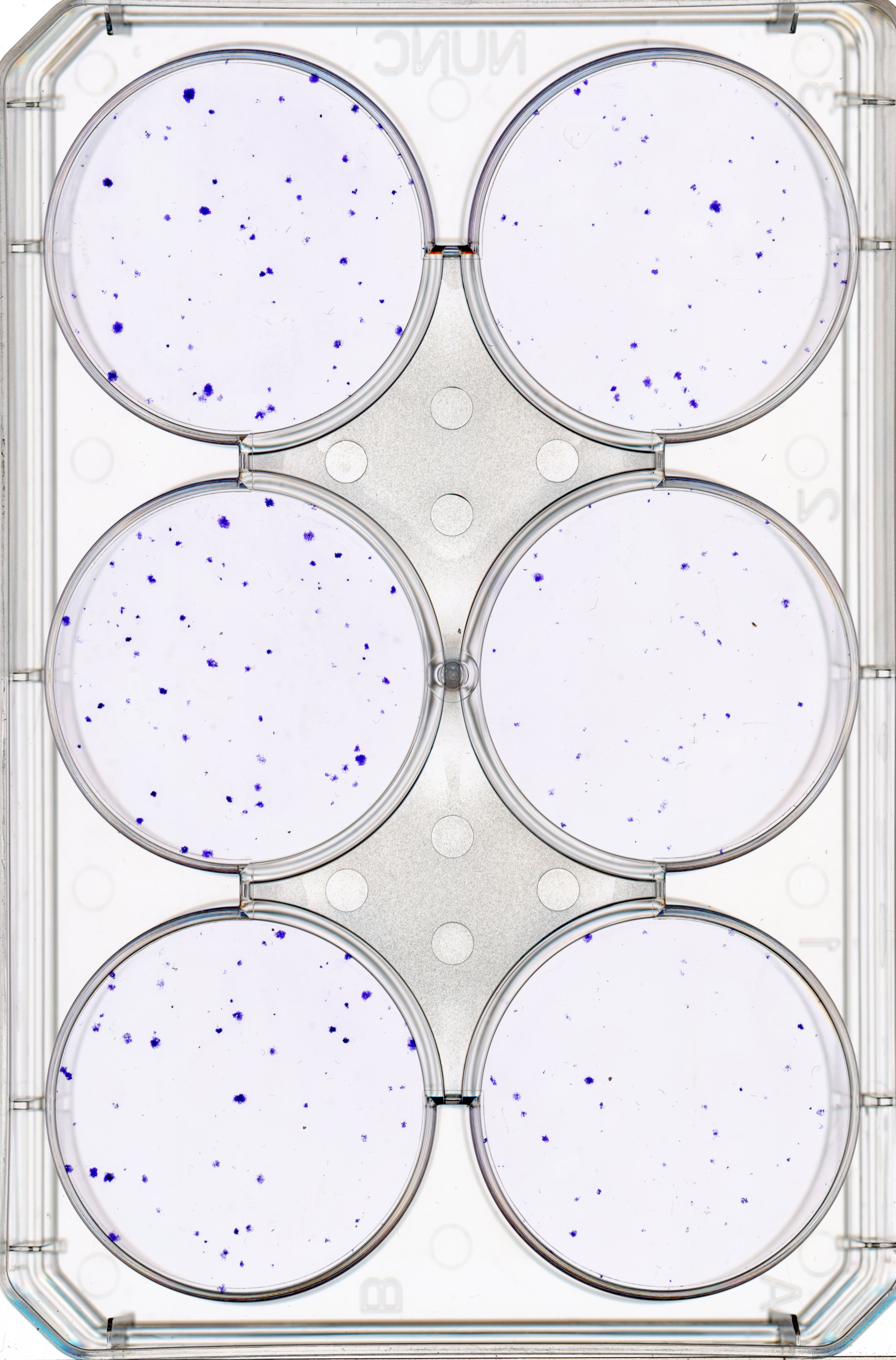

Supplement: Supplementary file 13 — Figure EV5 Source Data [file 44318_2024_108_MOESM13_ESM.zip › EMBOJ-2023-115654_FigEV5_sourcedata/EV5G/E230217 WT 5dC200-300.jpg]

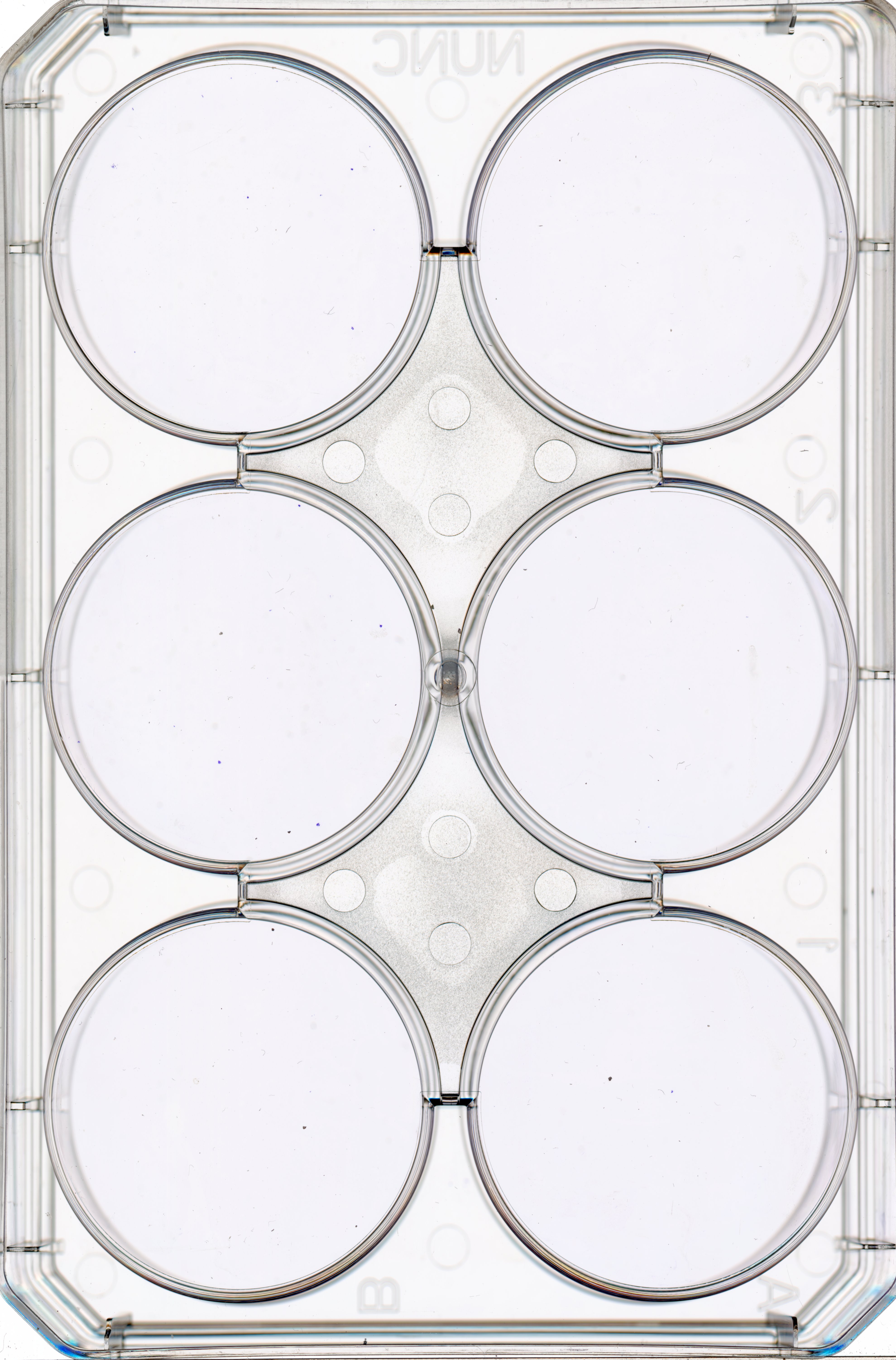

Supplement: Supplementary file 13 — Figure EV5 Source Data [file 44318_2024_108_MOESM13_ESM.zip › EMBOJ-2023-115654_FigEV5_sourcedata/EV5G/E230217 U2KtrsDKOcl10 5dC50-100.jpg]

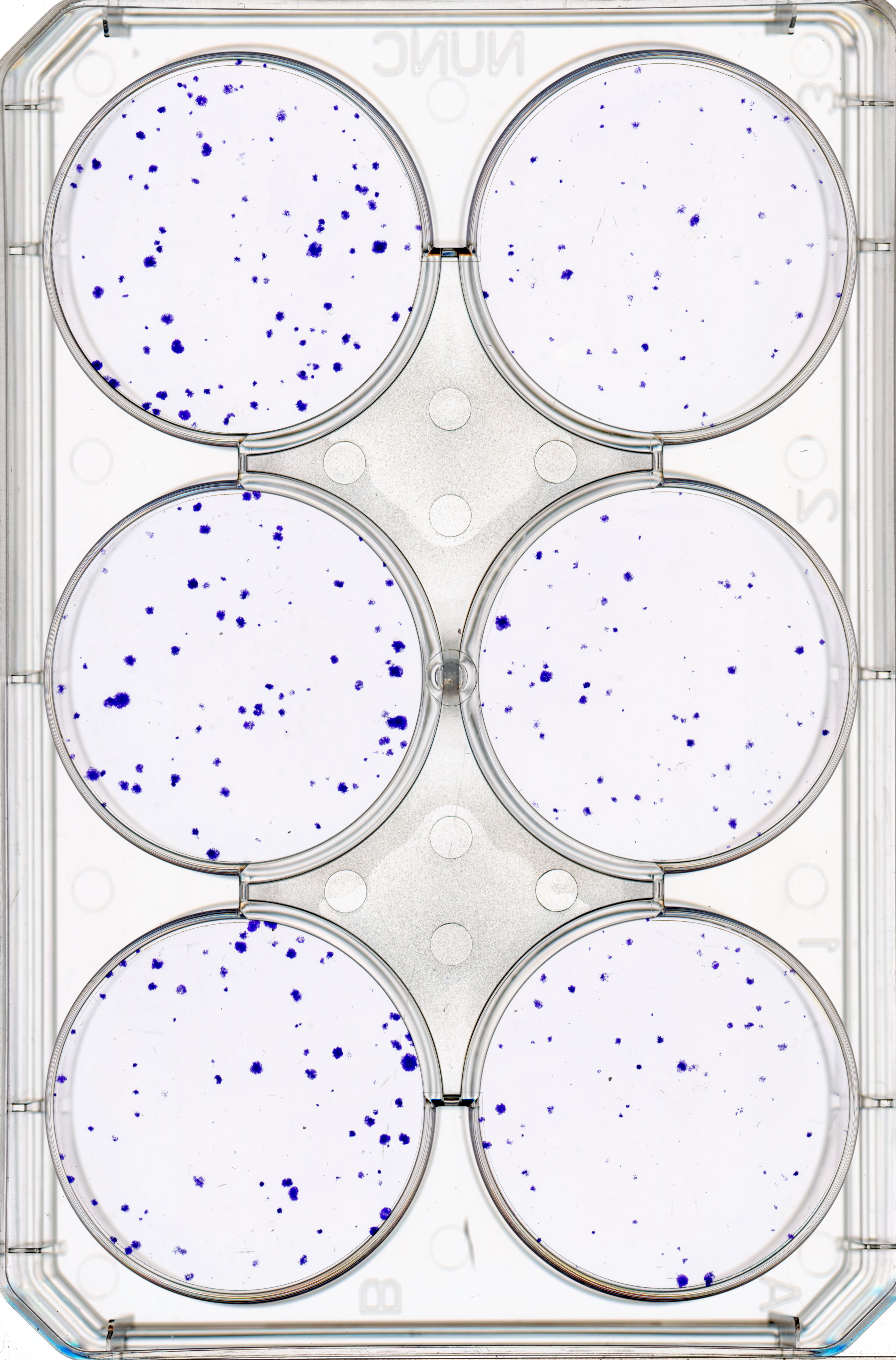

Supplement: Supplementary file 13 — Figure EV5 Source Data [file 44318_2024_108_MOESM13_ESM.zip › EMBOJ-2023-115654_FigEV5_sourcedata/EV5G/E230217 TOPORS 5dC50-100.jpg]

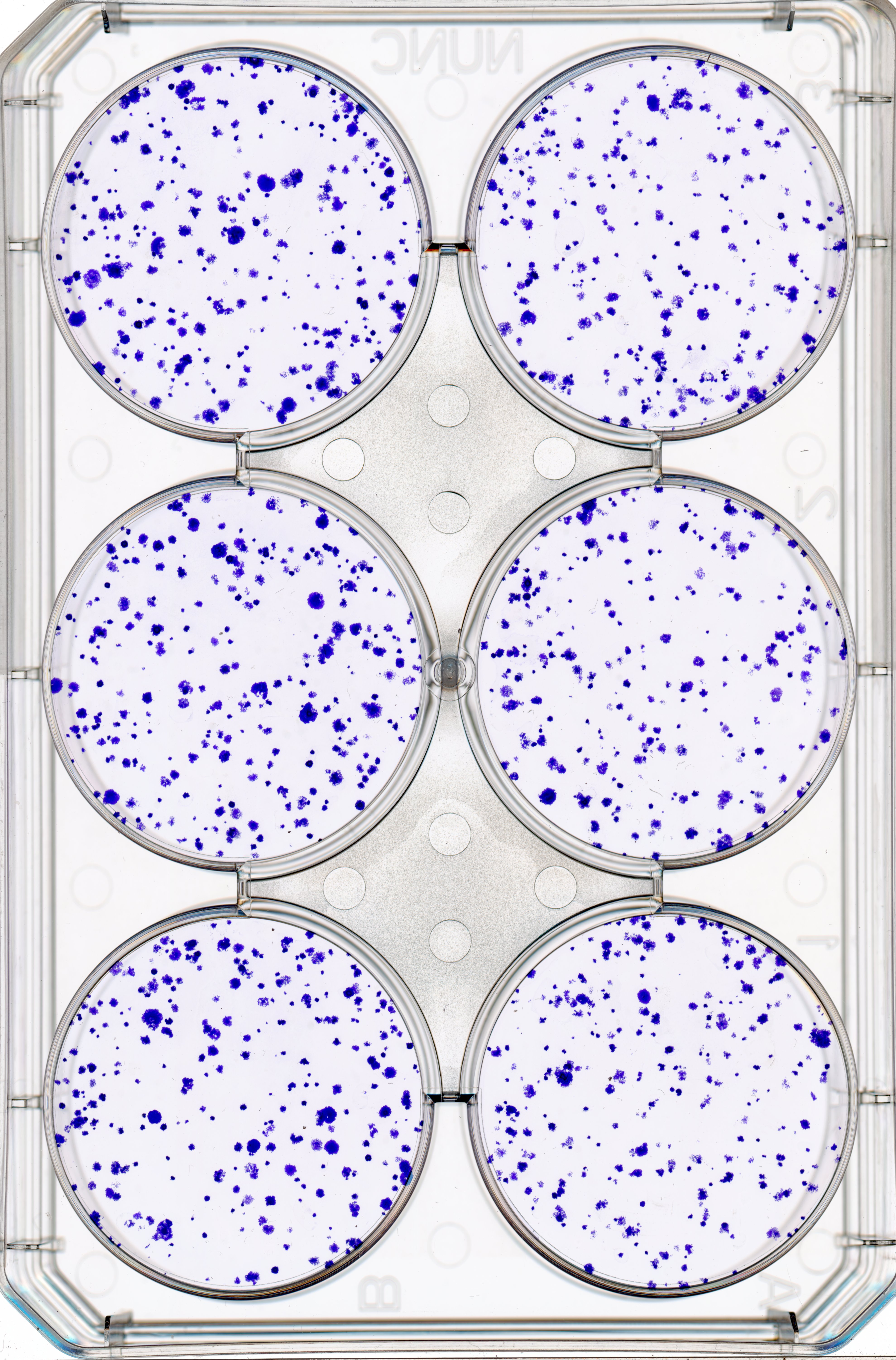

Supplement: Supplementary file 13 — Figure EV5 Source Data [file 44318_2024_108_MOESM13_ESM.zip › EMBOJ-2023-115654_FigEV5_sourcedata/EV5G/E230217 U2KtrsDKO1cl 5dC0-5.jpg]

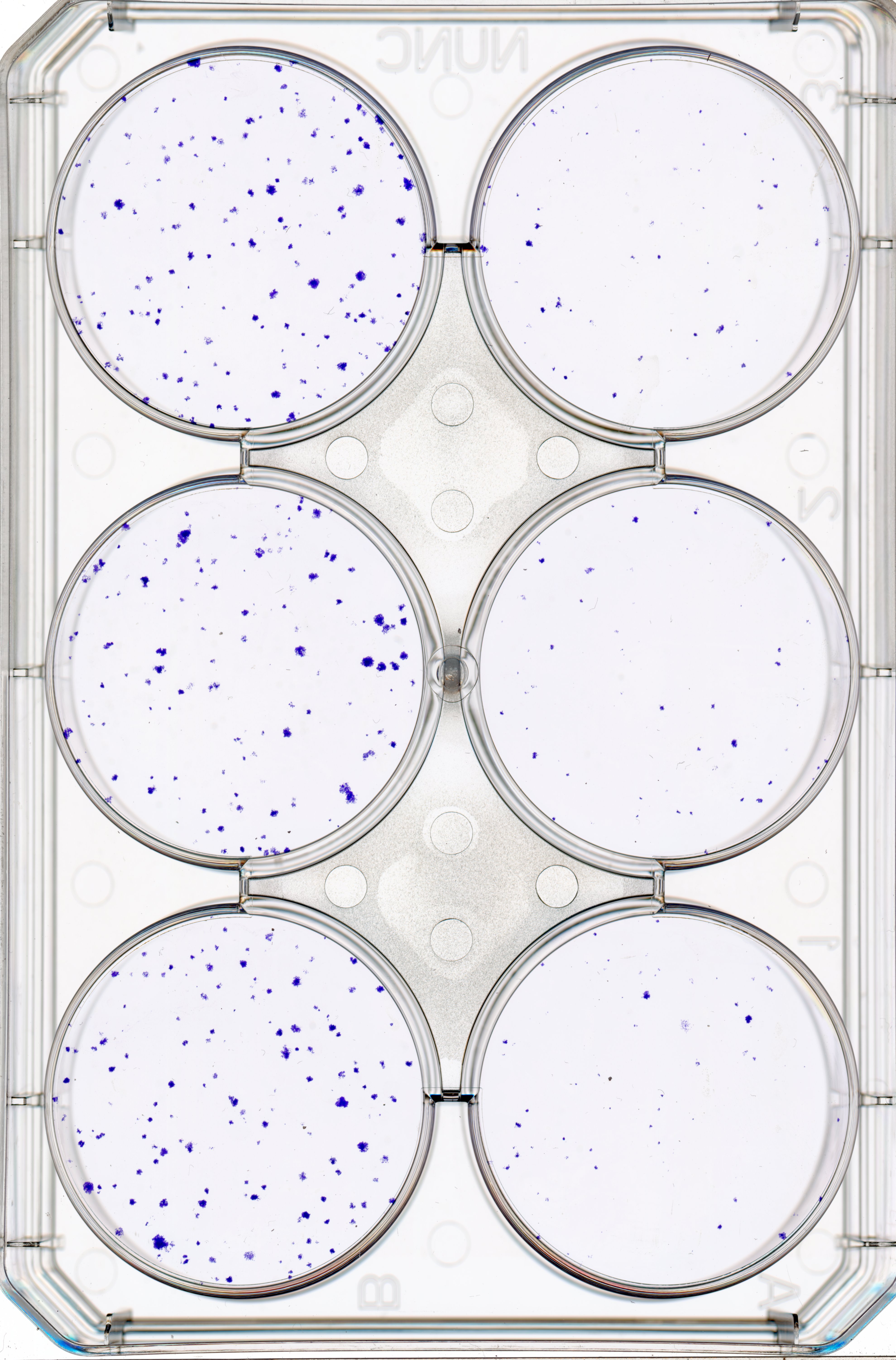

Supplement: Supplementary file 13 — Figure EV5 Source Data [file 44318_2024_108_MOESM13_ESM.zip › EMBOJ-2023-115654_FigEV5_sourcedata/EV5G/E230217 U2KtrsDKOcl10 5dC10-20.jpg]

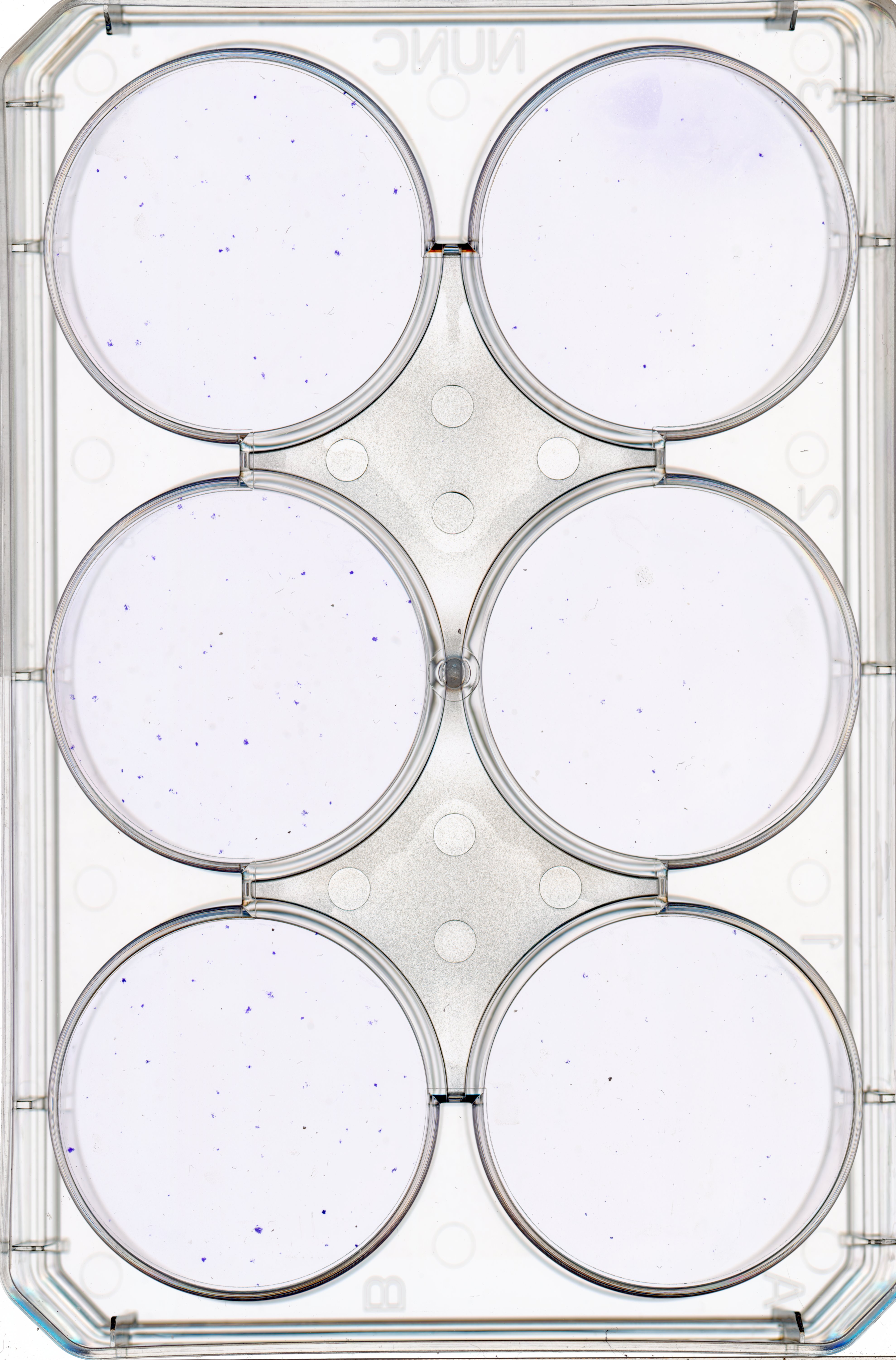

Supplement: Supplementary file 13 — Figure EV5 Source Data [file 44318_2024_108_MOESM13_ESM.zip › EMBOJ-2023-115654_FigEV5_sourcedata/EV5G/E230217 TOPORS 5dC200-300.jpg]
