# Supplementary material for: Decitabine cytotoxicity is promoted by dCMP deaminase DCTD and mitigated by SUMO-dependent E3 ligase TOPORS
Source: EMBO J. 2024 May 17;43(12):6. doi: 10.1038/s44318-024-00108-2 (PMC11183266; doi:10.1038/s44318-024-00108-2)
Supplement: Supplementary file 16 — Expanded View Figures [file 44318_2024_108_MOESM16_ESM.pdf]

Expanded View Figures

Figure EV1. DCTD promotes DNMT1-independent 5-aza-dC cytotoxicity.

(**A**, **B**) Western blot in WT and *DCTD* KO (**A**) or *DCK* KO (**B**) HAP1 cells with the indicated antibodies; representative of 3 (**A**) and 2 (**B**) independent experiments. The DCTD-specific band in (**A**) is marked with a red asterisk (*). (**C**) Speculative model for DCK-independent incorporation of 5-aza-dC into DNA and subsequent DNMT1 trapping. Briefly, upon cellular uptake, 5-aza-dC can be deaminated by CDA, followed by triphosphorylation involving the activity of TK1, followed by possible conversion of 5-aza-dUTP to 5-aza-dCTP by CTPS1 and subsequent DNA incorporation and DNMT1 trapping. (**D**) Western blot for the indicated antibodies in WT and *DCTD* KO HAP1 cells transfected with the indicated siRNAs; representative of three independent experiments. (**E**) Clonogenic survival assays with siRNA-transfected WT and *DCTD* KO cells from (**D**) treated with 5-aza-dC; $n = 3$ biological replicates, error bars ± SEM. (**F**) Representative images from (**D**) at selected 5-aza-dC doses. (**G**) Percentage of EdU-positive cells determined by flow cytometry of HAP1 WT, *DCTD* KO and *DCK* KO cells either untreated or treated with 1 µM 5-aza-dC for 3 h or 6 h; $n = 3$ biological replicates, error bars show mean ± SD. (**H**) Western blot with the indicated antibodies of polyclonal cell populations of WT and *DCTD* KO cells following CRISPR/Cas9-mediated depletion of PARP1 with the indicated sgRNAs; representative of two independent experiments. (**I**) Clonogenic survival assays on cells from (**G**) treated with 5-aza-dC; $n = 3$ biological replicates, error bars ± SEM. (**J**) Representative images from (**I**) at selected 5-aza-dC doses. Source data are available online for this figure.

 © The Author(s)

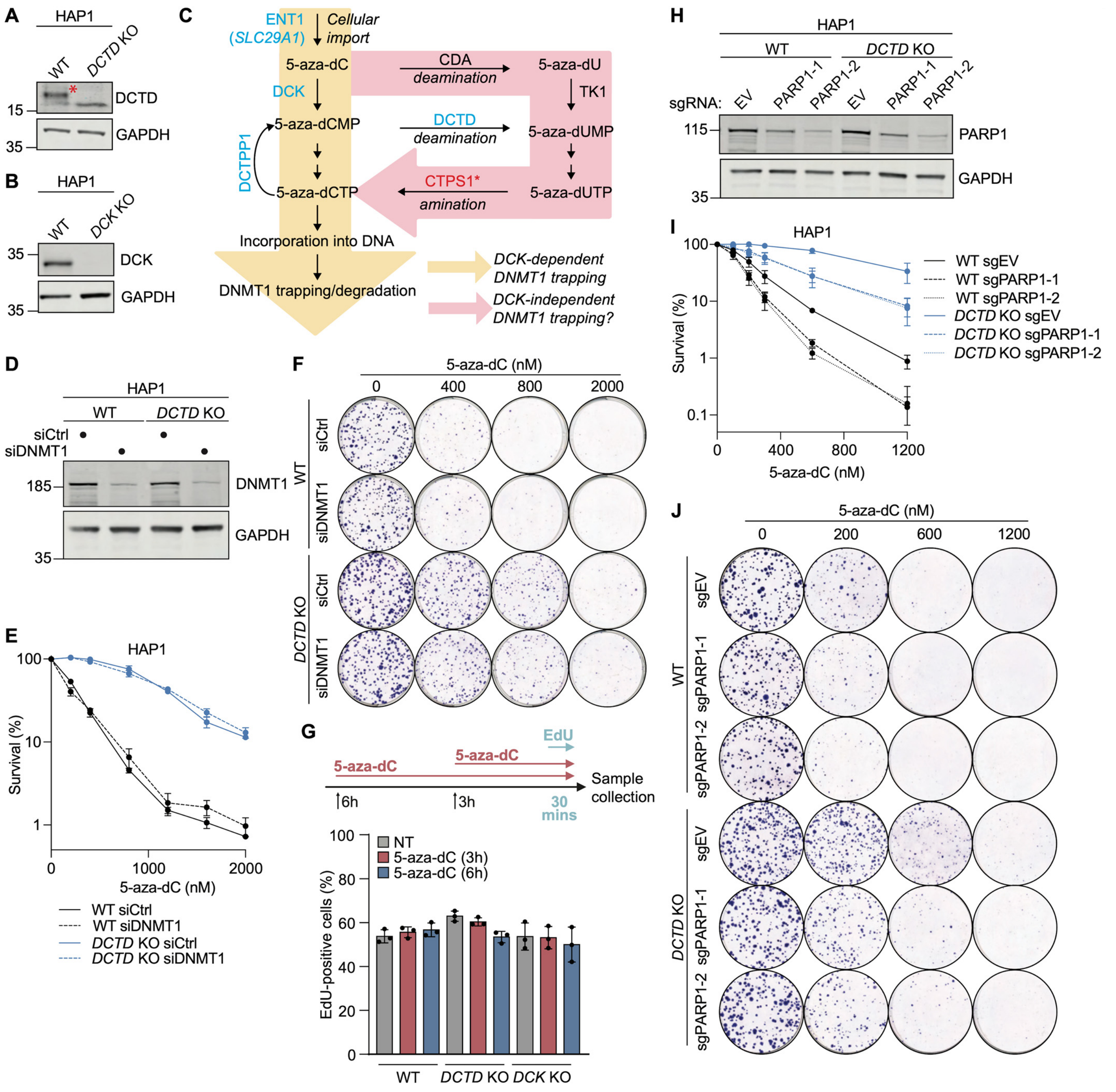
A
HAP1
WT
DCTD KO
DCTD
GAPDH
15
35
B
HAP1
WT
DCK KO
DCK
GAPDH
35
35
C
ENT1
(SLC29A1)
Cellular import
5-aza-dC
DCK
5-aza-dCMP
DCTPP1
5-aza-dCTP
Incorporation into DNA
DNMT1 trapping/degradation
CDA
deamination
5-aza-dU
TK1
DCTD
deamination
5-aza-dUMP
CTPS1*
amination
5-aza-dUTP
DCK-dependent DNMT1 trapping
DCK-independent DNMT1 trapping?
D
HAP1
WT
DCTD KO
siCtrl
siDNMT1
185
DNMT1
GAPDH
35
E
HAP1
Survival (%)
100
10
1
0
1000
2000
5-aza-dC (nM)
WT siCtrl
WT siDNMT1
DCTD KO siCtrl
DCTD KO siDNMT1
F
5-aza-dC (nM)
0
400
800
2000
WT
siCtrl
siDNMT1
DCTD KO
siCtrl
siDNMT1
G
5-aza-dC
5-aza-dC
EdU
6h
3h
30 mins
Sample collection
NT
5-aza-dC (3h)
5-aza-dC (6h)
EdU-positive cells (%)
100
80
60
40
20
0
WT
DCTD KO
DCK KO
H
HAP1
WT
DCTD KO
sgRNA:
EV
PARP1-1
PARP1-2
EV
PARP1-1
PARP1-2
115
PARP1
GAPDH
35
I
HAP1
Survival (%)
100
10
1
0.1
0
400
800
1200
5-aza-dC (nM)
WT sgEV
WT sgPARP1-1
WT sgPARP1-2
DCTD KO sgEV
DCTD KO sgPARP1-1
DCTD KO sgPARP1-2
J
5-aza-dC (nM)
0
200
600
1200
WT
sgEV
sgPARP1-1
sgPARP1-2
DCTD KO
sgEV
sgPARP1-1
sgPARP1-2

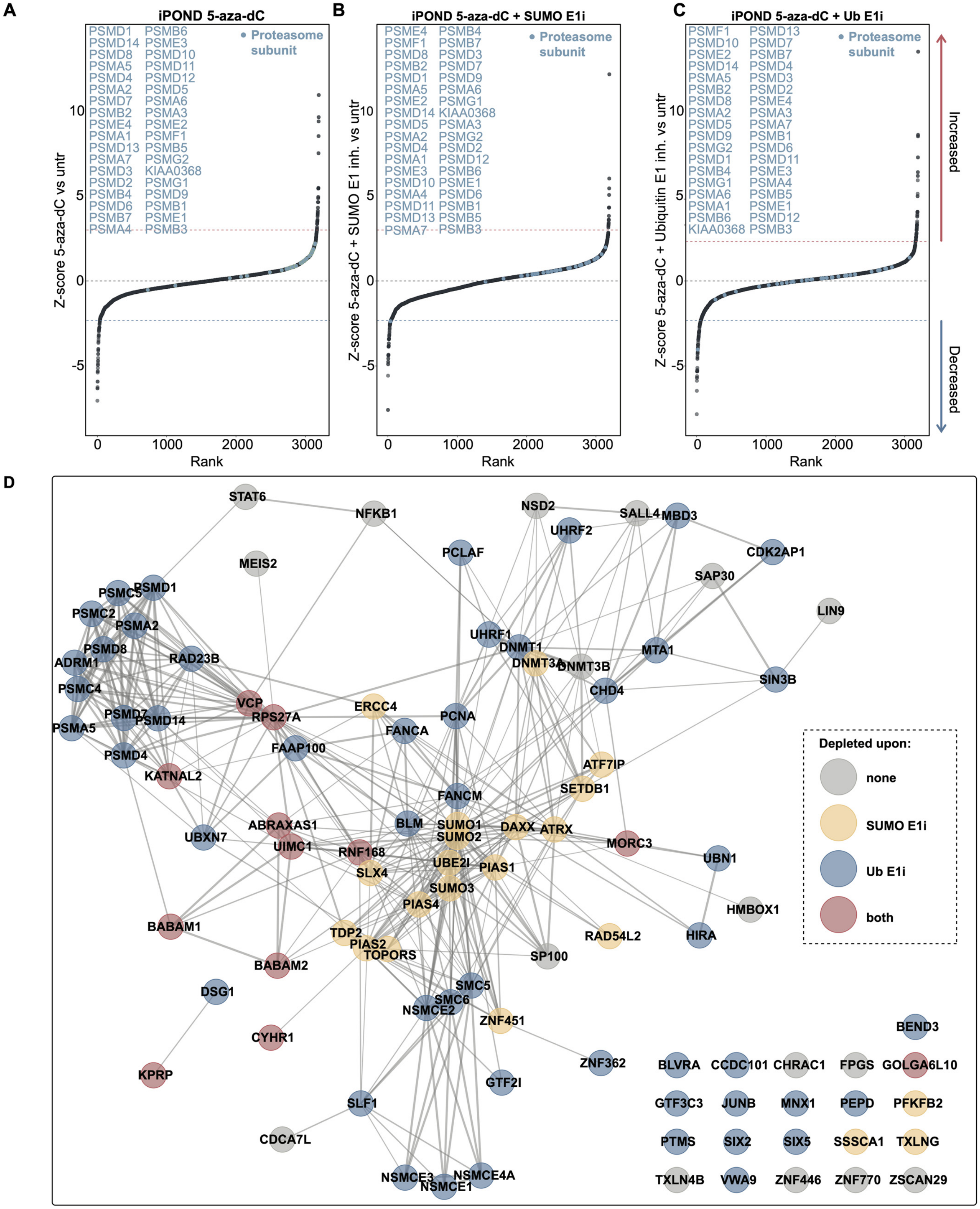
A
iPOND 5-aza-dC
PSMD1 PSMB6
PSMD14 PSME3
PSMD8 PSMD10
PSMA5 PSMD11
PSMD4 PSMD12
PSMA2 PSMD5
PSMD7 PSMA6
PSMB2 PSMA3
PSME4 PSME2
PSMA1 PSMF1
PSMD13 PSMB5
PSMA7 PSMG2
PSMD3 KIAA0368
PSMD2 PSMG1
PSMB4 PSMD9
PSMD6 PSMB1
PSMB7 PSME1
PSMA4 PSMB3
• Proteasome subunit
Z-score 5-aza-dC vs untr
Rank
B
iPOND 5-aza-dC + SUMO E1i
PSME4 PSMB4
PSMF1 PSMB7
PSMD8 PSMD3
PSMB2 PSMD7
PSMD1 PSMD9
PSMA5 PSMA6
PSME2 PSMG1
PSMD14 KIAA0368
PSMD5 PSMA3
PSMA2 PSMG2
PSMD4 PSMD2
PSMA1 PSMD12
PSME3 PSMB6
PSMD10 PSME1
PSMA4 PSMD6
PSMD11 PSMB1
PSMD13 PSMB5
PSMA7 PSMB3
• Proteasome subunit
Z-score 5-aza-dC + SUMO E1 inh. vs untr
Rank
C
iPOND 5-aza-dC + Ub E1i
PSMF1 PSMD13
PSMD10 PSMD7
PSME2 PSMB7
PSMD14 PSMD4
PSMA5 PSMD3
PSMB2 PSMD2
PSMD8 PSME4
PSMA2 PSMA3
PSMD5 PSMA7
PSMD9 PSMB1
PSMG2 PSMD6
PSMD1 PSMD11
PSMB4 PSME3
PSMG1 PSMA4
PSMA6 PSMB5
PSMA1 PSME1
PSMB6 PSMD12
KIAA0368 PSMB3
• Proteasome subunit
Z-score 5-aza-dC + Ubiquitin E1 inh. vs untr
Rank
Increased
Decreased
D
STAT6 NFKB1 NSD2 SALL4 MBD3 UHRF2 PCLAF CDK2AP1 MEIS2 SAP30 LIN9
PSMC5 PSMD1 PSMC2 PSMA2 PSMD8 ADRM1 RAD23B PSMC4 VCP RPS27A PSMD7 PSMD14 PSMA5 PSMD4 KATNAL2 FAAP100 ERCC4 FANCA PCNA
UHRF1 DNMT1 DNMT3A DNMT3B MTA1 CHD4 SIN3B
ATF7IP SETDB1 FANCM BLM SUMO1 SUMO2 DAXX ATRX MORC3 UBN1
UBXN7 ABRAXAS1 UIMC1 RNF168 SLX4 UBE2I PIAS1 SUMO3 PIAS4 HMBOX1
BABAM1 TDP2 PIAS2 TOPORS SP100 RAD54L2 HIRA BABAM2
DSG1 SMC5 SMC6 NSMCE2 ZNF451 CYHR1 ZNF362 KPRP GTF2I SLF1 CDCA7L NSMCE3 NSMCE4A NSMCE1
BEND3
BLVRA CCDC101 CHRAC1 FPGS GOLGA6L10
GTF3C3 JUNB MNX1 PEPD PFKFB2
PTMS SIX2 SIX5 SSSCA1 TXLNG
TXLN4B VWA9 ZNF446 ZNF770 ZSCAN29
Depleted upon:
none
SUMO E1i
Ub E1i
both

 © The Author(s)

Figure EV2. iPOND identifies the SUMO- and ubiquitin-dependent DNMT1-DPC-proximal proteome.

(**A–C**) Ranked standardised enrichment of proteasomal subunits detected by iPOND-MS from 5-aza-dC-treated (**A**), 5-aza-dC- and SUMO E1i-co-treated (**B**), and 5-aza-dC- and Ub E1i-co-treated (**C**) over untreated cells. (**D**) STRING analysis of proteins enriched on nascent DNA after 5-aza-dC treatment with SUMO/ubiquitin dependencies indicated, as assessed by iPOND-MS.

© The Author(s)

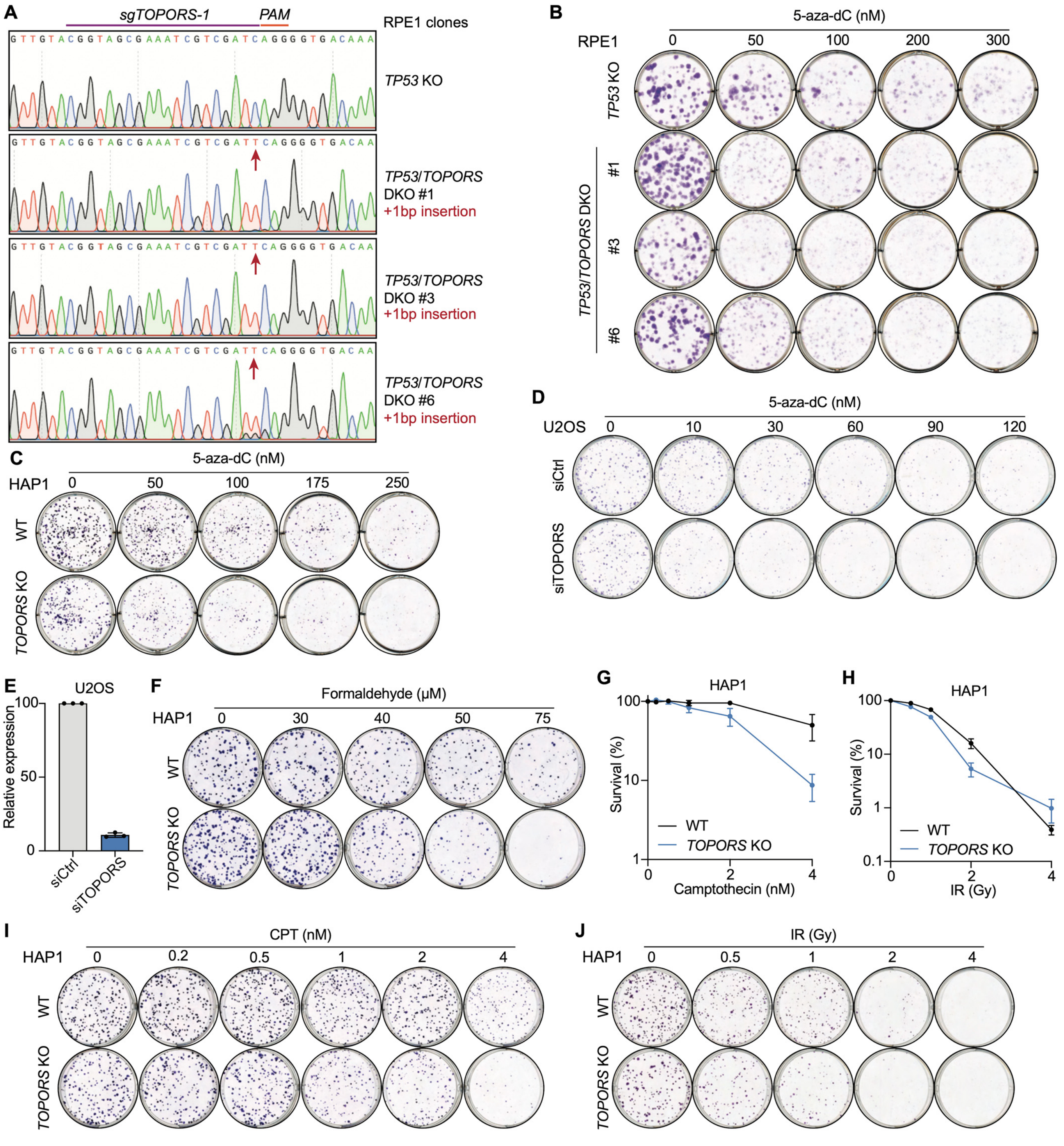

Figure EV3. TOPORS loss sensitises cells to DPC-inducing agents.

(**A**) Validation by Sanger sequencing of *TP53/TOPORS* DKO RPE1 clones. (**B–D**) Representative images of selected 5-aza-dC doses from clonogenic survival assays in Fig. 4A (**B**), Fig. 4B (**C**) and Fig. 4C (**D**). (**E**) Relative expression levels of TOPORS from U2OS cells 72 h after siRNA-mediated depletion of TOPORS measured by qPCR, relative to GAPDH expression and normalised to TOPORS expression level in siCtrl cells; $n = 3$ replicates, error bars ± SEM. (**F**) Representative images from clonogenic survival assays in Fig. 4D. (**G**, **H**) Clonogenic survival assays in WT and *TOPORS* KO HAP1 cells treated with camptothecin (**G**) and ionising radiation (IR; **H**); $n = 3$ biological replicates, error bars ± SEM. (**I**, **J**) Representative images from clonogenic survival assays in (**G**, **H**), respectively. Source data are available online for this figure.

 © The Author(s)

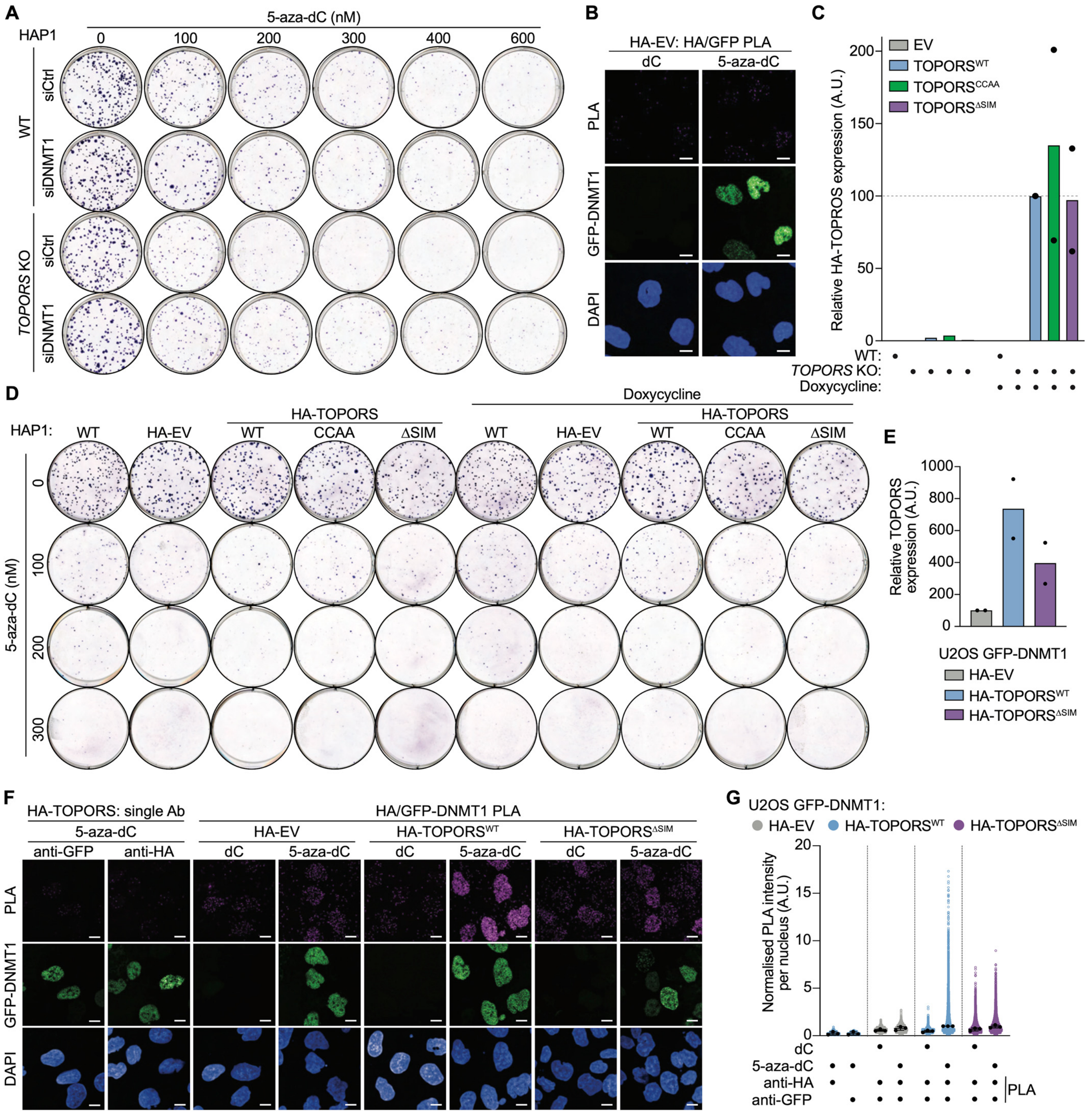

Figure EV4. TOPORS promotes 5-aza-dC resistance through its RING domain and SUMO-interaction motifs.

(**A**) Representative images from clonogenic survival assays in Fig. 4E. (**B**) Proximity ligation assay in U2OS cells expressing GFP-DNMT1 and HA-EV, treated with dC or 5-aza-dC; quantification in Fig. 4H. (**C**) Expression levels of HA-TOPORS after doxycycline induction measured by qPCR, relative to GAPDH expression and normalised to doxycycline-induced HA-TOPORS^{WT}; $n = 2$ biological replicates performed in technical triplicate, error bars ± SEM. (**D**) Representative images from clonogenic survival assays in Fig. 5B,C. (**E**) Expression levels of TOPORS in U2OS GFP-DNMT1 cells measured by qPCR relative to GAPDH and normalised to U2OS GFP-DNMT1 cells expressing HA-EV; $n = 2$ biological replicates performed in technical triplicate. (**F**, **G**) Representative images (**F**) and quantification (**G**) of PLA in U2OS GFP-DNMT1 cells expressing HA-EV, HA-TOPORS^{WT} or HA-TOPORS$^{\Delta SIM}$ treated with dC or 5-aza-dC; scale bars = 10 µm. In (**G**), black dots display the median normalised PLA intensity of each biological replicate for each condition; $n = 3$ independent biological replicates, error bars ± SEM. Source data are available online for this figure.

© The Author(s)

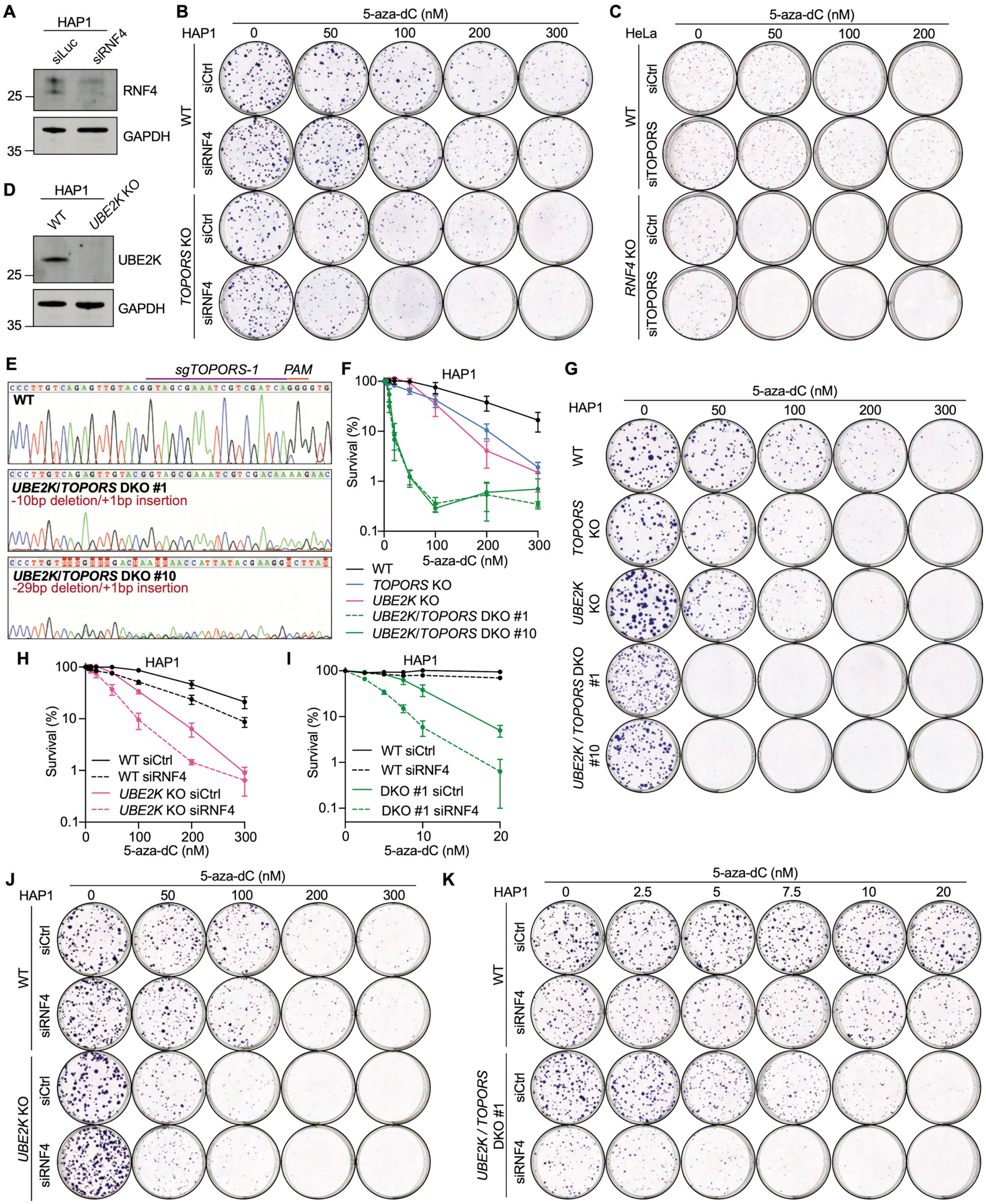
A
HAP1
siLuc
siRNF4
25
35
RNF4
GAPDH
B
5-aza-dC (nM)
HAP1
0
50
100
200
300
WT
siCtrl
siRNF4
TOPORS KO
siCtrl
siRNF4
C
5-aza-dC (nM)
HeLa
0
50
100
200
WT
siCtrl
siTOPORS
RNF4 KO
siCtrl
siTOPORS
D
HAP1
WT
UBE2K KO
UBE2K
GAPDH
25
35
E
sgTOPORS-1
PAM
WT
UBE2K/TOPORS DKO #1
-10bp deletion/+1bp insertion
UBE2K/TOPORS DKO #10
-29bp deletion/+1bp insertion
F
HAP1
Survival (%)
100
10
1
0.1
0
100
200
300
5-aza-dC (nM)
WT
TOPORS KO
UBE2K KO
UBE2K/TOPORS DKO #1
UBE2K/TOPORS DKO #10
G
5-aza-dC (nM)
HAP1
0
50
100
200
300
WT
TOPORS KO
UBE2K KO
UBE2K / TOPORS DKO #1
#10
H
HAP1
Survival (%)
100
10
1
0.1
0
100
200
300
5-aza-dC (nM)
WT siCtrl
WT siRNF4
UBE2K KO siCtrl
UBE2K KO siRNF4
I
HAP1
Survival (%)
100
10
1
0.1
0
10
20
5-aza-dC (nM)
WT siCtrl
WT siRNF4
DKO #1 siCtrl
DKO #1 siRNF4
J
5-aza-dC (nM)
HAP1
0
50
100
200
300
WT
siCtrl
siRNF4
UBE2K KO
siCtrl
siRNF4
K
5-aza-dC (nM)
HAP1
0
2.5
5
7.5
10
20
WT
siCtrl
siRNF4
UBE2K / TOPORS DKO #1
siCtrl
siRNF4

 © The Author(s)

Figure EV5. TOPORS functions in parallel to RNF4 and UBE2K to promote cellular 5-aza-dC tolerance.

(**A**) Western blot of RNF4 from HAP1 cells after siRNA-mediated depletion of RNF4; representative of two independent replicates. (**B**, **C**) Representative images at selected 5-aza-dC doses from clonogenic survival assays in Fig. 6B (**B**) and Fig. 6C (**C**). (**D**) Western blot of UBE2K in WT and *UBE2K* KO HAP1 cells; representative of two independent replicates. (**E**) Validation by Sanger sequencing of *UBE2K/TOPORS* DKO HAP1 clones. (**F**) Clonogenic survival assays in WT, *UBE2K* KO, *TOPORS* KO and *UBE2K/TOPORS* DKO HAP1 cells treated with 5-aza-dC; $n = 3$ biological replicates, error bars ± SEM. (**G**) Representative images of selected doses from (**F**). (**H**, **I**) Clonogenic survival assays with 5-aza-dC in WT and *UBE2K* KO (**H**; $n = 4$ biological replicates. Note that two replicates are shared with data shown in Fig. 6B) or *UBE2K/TOPORS* DKO #1 (**I**; $n = 3$ biological replicates) cells transfected with siCtrl or siRNF4; error bars ± SEM. (**J**, **K**) Representative images at selected 5-aza-dC doses from (**H**) and (**I**), respectively. Source data are available online for this figure.
